# Proceedings of the 31st European Paediatric Rheumatology Congress: part 2

**DOI:** 10.1186/s12969-024-01005-y

**Published:** 2024-09-09

## PReS 2024 Abstract Submission

### JIA (oligo, poly, psoriatic)

#### P177 Investigation of functionality, participation, and biopsychosocial status of individuals with JIA according to disease activity

##### Orkun Tüfekçi^1^, Sinan Buran^2^, Nur B. Karaca^1^, Emil Aliyev^3^, Yağmur Bayındır^3^, Yelda Bilginer^3^, Edibe Ünal^2^, Seza Özen^3^

###### ^1^Department of Basic Physiotherapy and Rehabilitation, Hacettepe University Institute of Health Sciences; ^2^Department of Heart and Respiratory Physiotherapy and Rehabilitation, Hacettepe University Faculty of Physical Therapy and Rehabilitation; ^3^Department of Pediatrics, Division of Rheumatology, Hacettepe University Faculty of Medicine, Ankara, Türkiye

####### **Correspondence:** Orkun Tüfekçi

*Pediatric Rheumatology 2024*, **22(2)**: PReS24-ABS-1330

**Introduction:** The relationship between disease activity status in JIA and the functionality, participation, and psychosocial status of these individuals has been emphasized. However, upon reviewing the literature, the need for evaluation of disease activity status in JIA in these aspects is reported (1, 2).

**Objectives:** This study aimed to investigate the functionality, participation, and biopsychosocial status of individuals with JIA according to disease activity.

**Methods:** Our study included fifty individuals (31 girls, 19 boys) diagnosed with JIA, of whom 35 had oligoarticular and 15 had polyarticular JIA, and who were followed up with routine controls. Demographic information of the participants was documented, and disease activity status was assessed using the Juvenile Arthritis Disease Activity Score in 71 joints (JADAS-71). Functionality was measured using the Childhood Health Assessment Questionnaire (CHAQ), participation was evaluated using the Child and Adolescent Scale of Participation (CASP), and biopsychosocial status was examined using the Juvenile Arthritis Biopsychosocial Questionnaire (JAB-Q). Disease activity was categorized based on the JADAS-71 score: ≤1 indicated inactive disease, while >10.5 indicated high disease activity (3). Group characteristics were compared using the Mann-Whitney U test.

**Results:** Demographic characteristics of inactive and high disease activity JIA patients were similar (p>0.05). CHAQ pain, general well-being, and total score, CASP home participation and total score, JAB-Q-child disease activity, joint, functionality, fatigue, and total scores were significantly better in favor of the inactive group; ESR value and number of affected active joints were higher in individuals with high disease activity (p<0.05).

**Conclusion:** This study showed that individuals with JIA exhibiting high disease activity were more affected in functionality, participation, and biopsychosocial status compared to those with inactive disease. The findings of this study suggest that these factors should be considered in the disease management of individuals with JIA starting from high disease activity.

**Date of birth::** novembre 0

**Patient Consent**

Not applicable (there are no patient data)

**Disclosure of Interest**

None Declared

**References**

1. Taxter AJ, Wileyto EP, Behrens EM, Weiss PF. Patient-reported Outcomes across Categories of Juvenile Idiopathic Arthritis. The Journal of Rheumatology. 2015;42(10):1914-21.
2. Unal E, Batu ED, Sonmez HE, Arici ZS, Arin G, Karaca NB, et al. A new biopsychosocial and clinical questionnaire to assess juvenile idiopathic arthritis: JAB-Q. Rheumatology International. 2018;38(8):1557-64.
3. Weiss PF, Beukelman T, Schanberg LE, Kimura Y, Colbert RA. Enthesitis-related Arthritis Is Associated with Higher Pain Intensity and Poorer Health Status in Comparison with Other Categories of Juvenile Idiopathic Arthritis: The Childhood Arthritis and Rheumatology Research Alliance Registry. The Journal of Rheumatology. 2012;39(12):2341-51.

### JIA (oligo, poly, psoriatic)

#### P178 Methotrexate polyglutamate concentrations in pediatric patients with juvenile idiopathic arthritis and association with clinical response

##### Marta Klanjscek^1^, Paolo Dalla Zuanna^1, 2^, Martina Franzin^3^, Andrea Taddio^1, 3^, Serena Pastore^3^, Giuliano Ponis^3^, Gilda Paternuosto^3^, Riccardo Addobbati^3^, Gabriele Stocco^1, 3^

###### ^1^Department of Medical, Surgical and Health Sciences, University of Trieste, Trieste; ^2^Clinical and Experimental Pharmacology, Centro di Riferimento Oncologico di Aviano, Aviano; ^3^IRCCS Burlo Garofolo, Trieste, Italy

####### **Correspondence:** Paolo Dalla Zuanna

*Pediatric Rheumatology 2024*, **22(2)**: PReS24-ABS-1580

**Introduction:** Methotrexate (MTX) is one of the first-line medications for the treatment of juvenile idiopathic arthritis (JIA). Unfortunately, about 35% of patients does not respond to MTX. To personalize MTX therapy it would be important to carry out therapeutic drug monitoring, quantifying the erythrocyte levels of MTX and its polyglutamate (MTX-PG1-7) active metabolites. It appears that levels of long-chain polyglutamates (MTX-PG3-5) correlates with lower disease activity.

**Objectives:** This study aims to determine the concentrations of MTX-PG1-7 and their association with clinical response in a cohort of pediatric JIA patients.

**Methods:** The study was conducted at IRCCS Burlo Garofolo (Trieste, Italy). Pediatric JIA patients, treated with MTX since more than 3 months, not suspected of poor compliance (total polyglutamate levels (MTX-PGTOT) > 2 nM), both in cotreatment with biological drug or not, were recruited. MTX-PG1-7 quantification was carried out by LC-MS/MS. Disease activity and remission were calculated using JADAS27 score and Wallace criteria, respectively. Association between MTX-PG levels and demographic/clinical variables was assessed by non-parametric tests (Kruskal-Wallis test for categorical variables and Spearman’s test for continuous variables). For diseases remission, association was evaluated by generalized linear mixed effect models of the binomial family, with remission as dependent variable, MTX-PG levels as independent variables (fixed effect) and the patient as the random effect.

**Results:** Thirty-four patients, for a total of 85 samples (median age: 11 y, 24 female, median dose: 0.34 mg/kg, median therapy duration: 13 months, 51 on biological cotreatment (22 adalimumab, 11 infliximab and 18 other) were enrolled. MTX-PG3-5 levels were significantly higher in MTX monotherapy patients (kruskal-wallis test, p=0.0069) while a trend show that MTX-PG1 levels were higher in patients cotreated with biologic (kruskal-wallis test, p=0.061). Patients receiving subcutaneous MTX showed significantly higher MTX-PGTOT levels than patients taking it orally (Kruskal-wallis test, p=0.0015). No association was observed between MTX-PG levels and the JADAS27 score. A trend for an association between MTX-PG3-5 levels with clinical remission was seen (OR: 1.01, 95% CI: 1-1.02, p=0.090).

**Conclusion:** MTX-PG3-5 levels were significantly higher in MTX monotherapy patients while MTX-PG1 levels tended to be higher in patients cotreated with biologics: since it was previously shown that MTX-PG3-5 levels correlate with response, our hypothesis is that low MTX-PG3-5 levels require biologic therapy. Then, we found that MTX-PGTOT levels are significantly higher in patients taking subcutaneous MTX than oral. Finally, we found a trend for an association between MTX-PG3-5 levels with clinical remission, that must be further confirmed.

**Patient Consent**

Yes, I received consent

**Disclosure of Interest**

None Declared

### JIA (oligo, poly, psoriatic)

#### P179 Immunoprofiling of oligoarticular jia reveals upregulation of cell chemotaxis and migration, suggests biomarkers correlated with disease activity and pain and with the potential to predict disease outcome

##### Xingzhao Wen^1^, Cecilia Aulin^1^, Erik Sundberg^2^, Heshuang Qu^1^, André Struglics^3^, Erik Melén^4^, Maria Altman^2^, Helena Erlandsson Harris^1, 5^

###### ^1^Department of medicine, Solna; ^2^Department of Women’s and Children’s Health, Karolinska Institutet, Stockholm; ^3^Department of Clinical Sciences Lund, Lund university, Lund; ^4^Department of Clinical Sciences and Education, Karolinska Institutet, Stockholm, Sweden; ^5^University of Bergen, Bergen, Norway

####### **Correspondence:** Xingzhao Wen

*Pediatric Rheumatology 2024*, **22(2)**: PReS24-ABS-1279

**Introduction:** Oligoarticular juvenile idiopathic arthritis (oJIA) is the most common subtype of JIA. Despite advances in the past decades, the pathogenesis of oJIA remains incompletely understood.

**Objectives:** In this study, we set out to map the immunoprofile of oJIA using plasma and synovial fluid (SF) samples to provide new molecular insights into the pathogenesis of oJIA and to identify potential novel diagnostic and prognostic biomarkers for oJIA.

**Methods:** 38 plasma and 63 synovial fluid (SF) samples from oJIA patients, 38 plasma samples from age- and sex-matched healthy controls (HC), 12 SF samples from adolescent patients undergoing arthroscopic examination resulting in no abnormal clinical features (CTRL), and 26 SF samples from adolescent knee injury patients were analysed by Olink proteomics assay (panel of 92 inflammatory markers). Clinical data for oJIA patients were retrieved from the Swedish pediatric rheumatology register and from medical records. Differentially expressed proteins (fold change > 2, p-value < 0.05, DEPs) were identified for further STRING and gene ontology (GO) biological process analyses. Receiver-operating characteristic (ROC) analysis was performed to identify the diagnostic or predictive ability of potential biomarkers.

**Results:** Plasma immunoprofiles were largely overlapping between oJIA and HC. In contrast, a clear separation was evident comparing SF immunoprofiles of oJIA and CTRL. In SF, 48 DEPs were recorded in oJIA compared to HCs, which identified leukocyte migration and cell chemotaxis as top significantly activated pathways. Comparative analysis of DEPs in oJIA versus controls and DEPs in knee injury patients versus CTRL revealed 13 proteins (ADA, CD8A, CD5, CD6, CD244, CXCL9, IFNg, IL-12B, IL-17A, PD-L1, TNF, TNFB, TNFRSF9) being specific for oJIA. Comparison of immunoprofiles with available clinical data for the oJIA patients identified proteins in plasma (IL6, MMP-1, VEGFA) and in SF (IL6, MMP-1, CCL20, OSM) that correlate with cJADAS-71 and pain. Finally, by comparing immunoprofiles between an oJIA subgroup with “remission-at-5-years and a “non-remission subgroup” we identified CXCL9 and CXCL10 as potential predictive biomarkers for remission.

**Conclusion:** Immunoprofiling SF samples from oJIA defined a set of biomarkers with altered expression in oJIA, where chemotactic and migration related pathways were activated. The inflammation pattern in oJIA suggests adaptive immune reactions, as opposed to the pattern seen after knee injuries. Plasma IL6 and MMP-1 can serve as biomarkers for disease activity and pain separately, while plasma VEGFA offers an alternative biomarker option. In SF, IL6, MMP-1, CCL20 and OSM associate with disease activity assessment. Notably, increased SF levels of CXCL9 and CXCL10 early in disease are associated with an increased likelihood of chronic disease. The plasma immunoprofiles were highly overlapping between oJIA and the control group, which supports the notion that oJIA is a local rather than a systemic disease. Thus, SF is more suitable than plasma for immunopathogenesis studies.

**Patient Consent**

Yes, I received consent

**Disclosure of Interest**

None Declared

### JIA (oligo, poly, psoriatic)

#### P180 Interferon pathways are associated with the response to methotrexate treatment in juvenile idiopathic arthritis

##### Melissa Kartawinata^1, 2^, Wei-Yu Lin^3^, Beth Jebson^1, 2^, Kathryn O’Brien^2^, Elizabeth Ralph^1, 2^, Restuadi Restuadi^1, 2, 4^, George T. Hall^5^, Sergi Castellano^5^, Chris Wallace^3, 6^, Lucy R. Wedderburn^1, 2, 4^ and on behalf of the CLUSTER Consortium

###### ^1^UCL Great Ormond Street Institute of Child Health; ^2^Centre for Adolescent Rheumatology Versus Arthritis at UCL, University College London Hospital (UCLH) and Great Ormond Street Hospital (GOSH), London; ^3^MRC Biostatistics Unit, University of Cambridge, Cambridge; ^4^National Institute for Health Research (NIHR) GOSH Biomedical Research Centre; ^5^Genetics and Genomic Medicine Research & Teaching Department, UCL Great Ormond Street Institute of Child Health, London; ^6^Cambridge Institute of Therapeutic Immunology and Infectious Disease (CITIID), Jeffrey Cheah Biomedical Centre, University of Cambridge, Cambridge, United Kingdom

####### **Correspondence:** Melissa Kartawinata

*Pediatric Rheumatology 2024*, **22(2)**: PReS24-ABS-1280

**Introduction:** Juvenile idiopathic arthritis (JIA) is the most common autoimmune rheumatic disease in children with methotrexate (MTX) as the first line treatment. However, about 50% of JIA patients will not respond well to MTX yet still experience drug side effects.

**Objectives:** To establish biomarkers that predict response to methotrexate treatment in patients with JIA.

**Methods:** Transcriptional analysis was performed on peripheral blood mononuclear cells (PBMC) from pre-MTX-treated JIA patients (n = 97) of all ILAR JIA subtypes but excluding systemic JIA. RNAseq was performed on total PBMC and sorted immune cell populations: CD4+ T cells, CD8+ T cells, CD19+ B cells, and CD14+ monocytes. Clinical data collected at the time of sampling (baseline) and at follow-up (between 3-12 months) were used to define outcomes, measured as change in active joint count (AJC) and change in Physician VAS (PhysVAS). After batch normalisation with ComBat-seq, differential gene expression (DGE) analysis was performed using limma-voom (R version) with age, sex, ethnicity, and steroid status included as covariates in each cell type. MTX outcome measures as continuous variables were used in the design matrix of DGE analysis. Gene set enrichment analysis (GSEA) was performed using gene-based permutation (fgsea^1^) and phenotype-based permutation (Broad Institute GSEA^2^) with MSigDb Hallmark pathways.

**Results:** DGE analysis showed minimal significant differentially expressed (DE) genes that passed 5% false discovery rate (FDR < 0.05) across different cell types. The greatest number of significant DE genes were observed in CD14+ monocytes, where baseline expression of 13 genes were significantly associated with change in PhysVAS. As alterations of gene expression for a heterogeneous disease such as JIA can be subtle and correlated between genes, GSEA was performed to investigate expression changes at pathway level. Both GSEA methods showed significant association of interferon-alpha and interferon gamma pathways with MTX response in all cell types including total PBMC (FDR < 0.05). Specifically in B cells, IL6-JAK/STAT3 signaling pathway also showed association to MTX response (FDR < 0.25). Pathway divergence between T cell and non-T cell lineages was also observed in the overall blood signature of pre-MTX JIA in association with their response outcomes.

**Conclusion:** Interferon pathways are associated with response to MTX and significantly enriched across all mononuclear cell lineages, suggesting genes in these pathways could be predictive biomarkers for response to MTX. Different directionality of pathways that might be relevant to JIA response to treatment with MTX is also observed in different cell lineages. This could potentially explain the difficulties of finding biomarkers which correlate with response to treatment from whole blood or PBMC.

**Patient Consent**

Not applicable (there are no patient data)

**Disclosure of Interest**

None Declared

**References**

1. bioRxiv 060012; doi: 10.1101/060012
2. Proc Natl Acad Sci. 2005 Oct 25;102(43):15545-50. doi: 10.1073/pnas.0506580102

### JIA (oligo, poly, psoriatic)

#### P181 Juvenile idiopathic arthritis: what promise do the printo classification criteria hold?

##### Mehmet Yıldız, Elif K. Kilic, Esma Aslan, Hakan Demir, Aybüke Günalp, Fatih Haslak, Ümit Gül^1^, Nergis Akay, Sezgin Şahin, Amra Adrovic, Kenan Barut, Ozgur Kasapcopur

###### Pediatric Rheumatology, Istanbul University-Cerrahpasa, Istanbul, Türkiye

####### **Correspondence:** Mehmet Yıldız

*Pediatric Rheumatology 2024*, **22(2)**: PReS24-ABS-1338

**Introduction:** The currently generally accepted classification criteria for Juvenile Idiopathic Arthritis (JIA) are the ILAR criteria, and a new draft classification criteria set was proposed by the PRINTO group in 2018.

**Objectives:** This study aims to evaluate the efficacy of preliminary PRINTO criteria, particularly in categorizing patients who were previously under undifferentiated JIA group according to ILAR classification criteria, thereby assessing the improvement in diagnostic inclusivity and potentially enhancing patient management and treatment strategies.

**Methods:** In a study conducted at the Department of Pediatric Rheumatology, Istanbul University-Cerrahpasa Cerrahpasa Medical School, in-person interviews were conducted with individuals diagnosed with JIA and attending the outpatient clinics. The study included patients who met the ILAR and/or PRINTO classification criteria, provided informed consent, and had a minimum six-month follow-up period. An experienced pediatric rheumatologist (MY) who was unaware of the patients' diagnoses used the ILAR and PRINTO systems to assign each patient to a specific JIA subtype based on their clinical, laboratory, and imaging data.

**Results:** The study included 364 patients (female: 200, male: 164). The mean age of the patients at the time of the study was 167.9 ± 59.5 months. In the patient group, the mean age at onset of symptoms, mean age at diagnosis and mean follow-up period were 78.7 ± 54.7 months, 88.3 ± 57.4 months and 71 ± 53.4 months, respectively. When ILAR diagnostic criteria were applied to the patient group, 155 (42.6%) patients could be classified as oligoarticular JIA, 78 (21.4%) as enthesitis-related arthritis, 33 (9.1%) as RF negative polyarticular JIA, 20 (5.5%) as systemic JIA, 15 (4.1%) as psoriatic arthritis, and 7 (1.9%) as RF positive polyarticular JIA. The number of patients in the undifferentiated JIA group was 56 (15.4%). According to the classification criteria proposed by PRINTO, 113 (31%) patients were classified as early-onset ANA-positive JIA, 112 (30.8%) as other JIA, 89 (24.5%) as enthesitis/spondylitis-related arthritis, 28 (7.7%) as systemic JIA, 16 (4.4%) as RF-positive JIA and 6 (1.6%) as unclassified JIA. Upon evaluating the 56 patients within the undifferentiated JIA group according to the ILAR classification using the PRINTO classification, it was observed that 14 (25%) were classified within the enthesitis/spondylitis-related arthritis group, 13 (23.2%) within the other JIA group, 11 (19.6%) within the early-onset ANA positive group, 9 (16.1%) within the RF positive JIA group, 8 (14.3%) within the systemic JIA group, and 1 (1.8%) within the unclassified JIA group.

**Conclusion:** This study suggests that the use of PRINTO criteria may be effective especially in the classification of patients who remain in the undifferentiated group in the ILAR classification criteria. Prospective studies are needed to make definitive conclusions.

**Date of birth::** décembre 0

**Patient Consent**

Yes, I received consent

**Disclosure of Interest**

None Declared

**References**

1. Martini A, Ravelli A, Avcin T, Beresford MW, Burgos-Vargas R, Cuttica R, et al. Toward new classification criteria for juvenile idiopathic arthritis: first steps, pediatric rheumatology international trials organization international consensus. J Rheumatol 2018;46:190–7.
2. Petty RE, Southwood TR, Manners P, Baum J, Glass DN, Goldenberg J, et al. International League of Associations for Rheumatology classification of juvenile idiopathic arthritis: second revision, Edmonton, 2001. J Rheumatol 2004;31:390.

### JIA (oligo, poly, psoriatic)

#### P182 ITPA activity to predict remission with methotrexate in juvenile idiopathic arthritis

##### Sofia Sindici Forgiarini^1^, Marianna Lucafò^2^, Debora Curci^3^, Davide Selvestrel^1^, Valentina Moressa^3^, Sofia Pagarin^1^, Alberto Tommasini^1, 3^, Mara Becker^4^, Laura Ramsey^5, 6^, Susan D. Thompson^7, 8^, Carl Langfeld^9^, Edoardo Marrani^10^, Gabriele Simonini^10^, Gabriele Stocco^1, 3^, Giuliana Decorti^1^, Andrea Taddio^1, 3^, Serena Pastore^3^

###### ^1^Department of Medicine, Surgery and Health Sciences; ^2^Department of life Science, University of Trieste; ^3^IRCCS Burlo Garofolo, Trieste, Italy; ^4^Department of Pediatrics, Duke University School of Medicine, Durham; ^5^Department of Pediatrics, Children's Mercy Kansas City, Kansas City; ^6^Department of Pediatrics, University of Missouri-Kansas City, Kansas City; ^7^Division and Center for Autoimmune Genomics and Etiology, Cincinnati Children's Hospital Medical Center; ^8^University of Cincinnati College of Medicine, Department of Pediatrics, Cincinnati; ^9^Department of Biostatistics and Data Science, Wake Forest School of Medicine, Winston-Salem, United States; ^10^I.R.C.C.S Meyer Children’s University Hospital, Florence, Italy

####### **Correspondence:** Serena Pastore

*Pediatric Rheumatology 2024*, **22(2)**: PReS24-ABS-1457

**Introduction:** Methotrexate (MTX) is the first-line therapy for juvenile idiopathic arthritis (JIA), although up to 45% of patients do not respond. Recent studies suggest that JIA patients with low inosine-triphosphate-pyrophosphatase (ITPA) activity are less likely to achieve clinical remission (1-2). Notably, *ITPA rs1127354* has been linked to decreased ITPA activity.

**Objectives:** To investigate the role of ITPA in modulating MTX response in JIA.

**Methods:** Pediatric patients from Trieste, Florence (Italy), and Kansas City (USA) receiving weekly MTX (10-15 mg/m^2^) for at least 12 months (204 patients, average age 7 ± 5 years, 73.6% females) were enrolled. Data were collected at baseline, 6 months, and every 3 months thereafter, with a follow-up of 12 months. ITPA activity was measured for 204 patients using erythrocyte lysates collected during clinical routine care (3). Moreover, DNAs of 131 patients were genotyped for *ITPA rs1127354*. In vitro investigations, conducted on human hepatocytes (IHH) transfected with an ITPA-containing plasmid and MOCK control, employed MTT cytotoxicity assay, real-time PCR for ADORA expression, quantification of released adenosine and western blot to quantify NF-kB p65 and its active phosphorylated form. Statistical analysis of remission and ITPA activity association was done by logistic regression analysis and non-parametric tests, adjusting for clinical center by multivariate logistic regression or meta-analysis combining results in each center. For the in vitro part, two-way ANOVA and Tukey's multiple comparisons test were used.

**Results:** Patients with JIA who reach remission had higher ITPA activity than those who did not reach remission (all measurements: 209.6±91.0 vs 169.7±80.5 nmol IMP/h, p = 0.00099; onset: 230.3±96.3 vs 193.5±84.9 nmol IMP/h, p = 0.025). Predictive cutoffs of 164.72 and 222.58 nmol IMP/h were established for overall and onset measurements, respectively. A trend towards reduced remission was observed for patients with variant *rs1127354* (p = 0.06). After 72h of treatment, IHH MOCK cells exhibited lower sensitivity to MTX compared to IHH ITPA cells at concentrations of 25, 200, and 600 nM (p<0.001, p<0.05, p<0.01, respectively). In both lines, the ADORA2B expression is significantly higher than ADORA1 and ADORA2A (p<0.01). Notably, preliminary results suggest that IHH ITPA cells treated with MTX 25 nM for 24 and 48 hours released more adenosine compared to IHH MOCK cells. Finally, IHH MOCK treated with 25 nM and 200 nM MTX showed increased activation of p65 compared with IHH ITPA (p<0.01, p<0.0001, respectively).

**Conclusion:** Patients with JIA with higher ITPA activity have increased probability of remission during therapy with methotrexate, both when measured at different times during therapy and at the therapy start. In this way it would be possible to predict the probability of remission at disease onset. Increased ITPA activity causes increased adenosine release, which may underlie the improved response to therapy observed in JIA patients.

**Patient Consent**

Not applicable (there are no patient data)

**Disclosure of Interest**

None Declared

**References**

1. Pastore S. et al. 2015; 35(4):619-27.
2. Wallace CA. et al. 2004; 31(11):2290-4.
3. Shipkova M. et al.; 2006; 52(2):240-7.

### JIA (oligo, poly, psoriatic)

#### P183 Relation between fatigue a other parent reported outcomes in a chort of children with JIA

#####  Cecilia Contratto^1^, Vanessa Fraga^2^, Carolina Quintela^3^, Marco Burrone^1^, Francesca Ridella^1^, Roberta Naddei^4^, Silvia Orsi^1^, Marco Gattorno^5^, Angelo Ravelli^6^, Alessandro Consolaro^1^

###### ^1^Pediatric Rheumatology, Istituto Giannina Gaslini, Genova, Italy; ^2^ Rheumatology Department, Unidade Local de Saúde Almada- Seixal, Almada; ^3^ Pediatrics Department, Unidade Local de Saúde Trás-os-Montes e Alto Douro, Vila Real, Portugal; ^4^Dipartimento di Scienze Mediche Traslazionali, Università degli Studi di Napoli Federico II, Napoli; ^5^ Rheumatology Department; ^6^Istituto Giannina Gaslini, Genova, Italy

####### **Correspondence:** Francesca Ridella

*Pediatric Rheumatology 2024*, **22(2)**: PReS24-ABS-1509

**Introduction:** Although fatigue is considered to be common in children and young people with juvenile idiopathic arthritis (JIA), the relevance of this symptom within the burden of the disease was poorly studied.

**Objectives:** To assess correlation of fatigue with physician centered measures and other patient reported outcomes (PROs) in a cohort of JIA patients

**Methods:** We enrolled in the study all JIA patient attending the outpatient clinic at the Study Unit in April 2024. Patients were asked to complete the Juvenile Arthritis Multidimensional Assessment Report (JAMAR) and the PROMIS® Item Bank v1.0 – Fatigue – Short Form 13a (FACIT-Fatigue), measuring the level of fatigue in 13 items with a 5-points Likert scales yielding a score of 0 to 52, with higher scores indicating more sever fatigue. Physician centered measures included the rheumatological examination form and the physician global assessment of disease activity (PhGA). We calculated Spearman rank correlation between fatigue score and quantitative measures included in the JAMAR, active joints count, and PhGA. Finally, we compared the levels of fatigue in patients considering themselves to be satisfied or not satisfied with current disease outcome.

**Results:** The questionnaires were proposed for completion before clinical examination to 51 JIA patients; 49 (87.2% females) completed both questionnaires and were included in the study. Patients had a median disease duration of 9.6 years (IQR 5.3-13.5) and were predominantly affected with oligoarticular JIA (72.3%). All JIA categories were represented. Median PhGA was 0 (0-2). Median fatigue score was 47 (38-51.5). Fatigue correlations were higher with JAMAR HRQoL tool (r = -0.67) and with JAMAR functional ability tool (r = -0.60). Correlation coefficients were between -0.4 and -0.6 with well-being VAS, patient disease activity VAS, pain VAS, morning stiffness duration, and cJADAS10. Correlations were poor (r < -0.4) with PhGA and active joint count. Correlations with HRQoL items in the JAMAR that were suggested to explore the domain of fatigue were the highest (r = -0.70 between fatigue score and JAMAR HRQoL items 3 and 9). Fatigue score was 37 (35-44) in 21 patients who were not satisfied with current disease outcome and was 51 (47-52) in 26 patients who considered their disease status as satisfactory (p < 0.001).

**Conclusion:** Fatigue seems to have a relevant weigh in the disease perception of children with JIA in a cohort of children with a generally well controlled disease. It is strongly correlated with HRQoL score and functional ability score. Patients not satisfied with disease outcome had significantly higher level of fatigue.

**Date of birth::** août 19, Y

**Patient Consent**

Yes, I received consent

**Disclosure of Interest**

None Declared

### JIA (oligo, poly, psoriatic)

#### P184 Development of a transitional therapeutic education programme for adolescents with Juvenile idiopathic arthritis

##### Sophie Hecquet^1^, Marion Thomas^1^, Alice Combier^1^, Julien Wipff^1^, Gertrude Touanga^1^, Pierre Quartier^2^, Yannick Allanore^1^

###### ^1^Rheumatology, Cochin Hospital; ^2^Rheumatology, (Hôpital Necker, Paris, France

####### **Correspondence:** Sophie Hecquet

*Pediatric Rheumatology 2024*, **22(2)**: PReS24-ABS-1571

**Introduction:** The transition period is a key moment in the management of patients with juvenile idiopathic arthritis (JIA). However, despite the advances that have been made to facilitate this crucial period, too many patients are still being lost. Therapeutic education is one of the tools we have to help patients and carers during this transition from paediatric to adult rheumatology.

**Objectives:** The aim of this study was to explore the expectations of adolescents with JIA and their caregivers, with a view to developing a patient education (PE) workshop tailored to their needs when they arrive on the adult rheumatology ward.

**Methods:** A questionnaire was emailed to patients from a single-centre retrospective cohort of JIA patients at the time of transition to adult rheumatology and to their parents.

**Results:** A total of 70 people responded to the survey, 46 patients and 24 caregivers. 65% of the patient respondents were aged between 18 and 25 years, 9% were aged between 15 and 18 years, and 26% were aged over 25 years. The main difficulties experienced by patients and caregivers during the transition were changing doctors (44%-29%) and lack of information (36%-29%). Over 28% of respondents reported no difficulties. 15% of patients had already attended an PE transition workshop. Among the topics that patients would like to discuss in PE, the evolution of the disease was mentioned by 91% of patients, 67% would like to talk about the psychological impact of the disease, 64% about treatments and 69% about the origin of the disease, 56% about diet, 47% about sports and over 30% about the professional/academic world and sexuality. Among parents, 82% would like to discuss the course of the disease and treatments, 52% the psychological impact of the disease and less than 30% other topics.

For both patients and parents, the most popular PE programme methods were: a dedicated application or website (56%-61%), one-to-one interviews (36%-39%), face-to-face group meetings (36%-26%), virtual group meetings (29%-39%) and escape games (29%-0%). The presence of a patient expert was considered important by almost 66% of patients and 55% of parents. Finally, 48% of patients would like to have a moment alone and a moment with their parent(s) during the PE workshop.

**Conclusion:** This study, which is still ongoing, has highlighted the various topics of interest to be addressed, as well as the PE workshop methods desired by patients and carers. The progression of the disease, its psychological impact and the presence of a patient expert seem to be essential elements to be included in the transitional PE workshops.

**Patient Consent**

Yes, I received consent

**Disclosure of Interest**

None Declared

### JIA (oligo, poly, psoriatic)

#### P185 Is it worth including the posterior scanning approach in the ultrasound assessment of the tibiotalar joint in Juvenile idiopathic arthritis?

##### Fabiana Di Stasio ^1^, Federica Plebani^2^, Chiara Provini^3^, Ivan Taietti^4^, Elisabetta Lo Iudice^3^, Federica Vianello^1^, Martina Rossano^1^, Sara Farhanghi^3^, Giovanni Filocamo^1, 3^, Stefano Lanni^1^

###### ^1^Fondazione IRCCS Ca' Granda Ospedale Maggiore Policlinico, Milano; ^2^Università degli Studi dell’Insubria, Varese; ^3^Università degli Studi di Milano, Milano; ^4^Università degli Studi di Pavia, Pavia, Italy

####### **Correspondence:** Fabiana Di Stasio

*Pediatric Rheumatology 2024*, **22(2)**: PReS24-ABS-1670

**Introduction:** The ankle is a complex anatomical structure owing to the multiple joint recesses and surrounding tendons. The tibiotalar joint (TTJ) is one of the main articular compartments of the ankle region. Ultrasound (US) is a valuable tool to confirm the presence of synovitis in the TTJ in children with juvenile idiopathic arthritis (JIA). It has been suggested to evaluate the TTJ on US using both an anterior and a posterior scanning approach.

**Objectives:** The aims of the study were: 1) to assess the frequency of synovitis on US in the anterior and posterior recesses of the TTJ; 2) to determine the most informative scanning approach to assess the TTJ on US in patients with JIA.

**Methods:** Fifty ankles of 38 patients with JIA with clinical arthritis were included in the study. The TTJs were scanned by a physician (SL) with experience in the US assessment of children with JIA, using an anterior and a posterior scanning approach. For both the joint recesses of the TTJ the detection of joint effusion (JE), synovial hypertrophy (SH) and power Doppler (PD) signal inside the area of SH were recorded on US evaluation. For the purpose of scoring, JE and SH were combined into a grey-scale (GS) US-score, which was representative of the joint cavity widening. The GS-US score and PD-US score were graded on a 4-point semiquantitative scale. An overall US severity score was calculated as the sum of the GS and PD scores for the anterior and the posterior recess of the TTJ.

**Results:** US-detected synovitis in 31/50 (62.0%) TTJs. All 31 ankles with US-detected TTJ involvement showed US findings on the anterior scanning approach. Only eight (25.8%) of them also had synovitis on the posterior scanning approach to the TTJ. Overall, JE and PD signals were recorded more frequently in the anterior recess of the TTJ than in the posterior (96.8% and 35.5% *versus* 77.8% and 44.4%, respectively). The frequency of SH as detected on US was equal for the anterior and the posterior side of the joint (100%). In the 31 ankles with US-determined TTJ involvement, the overall US severity score resulted as higher in the anterior aspect of the joint (median 3.0, IQR 2.0-4.0) compared to the posterior side (median 0.0, IQR 0.0-2.0).

**Conclusion:** Inflammation is frequently detected on US in the TTJ of patients with JIA and clinical ankle arthritis. The anterior scanning approach to the joint seems to be more appropriate for US evaluation of the TTJ. In this perspective, the possibility to scan only the more representative aspect of the TTJ may help to shorten the length of the US session, especially in the younger and poorly cooperative patients.

**Patient Consent**

Yes, I received consent

**Disclosure of Interest**

None Declared

### JIA (oligo, poly, psoriatic)

#### P186 The relationship of fatigue, pain interference and physical disability in children newly diagnosed with juvenile idiopathic arthritis: results from the Capri registry

##### Naomi Choong^1^, Michelle Batthish^2^, Roberta A. Berard^3^, Gaëlle Chédeville^4^, Brian M. Feldman^5^, Kristin M. Houghton^1^, Adam M. Huber^6^, Sarah James^1^, Jean-Philippe Proulx-Gauthier^7^, Dax G. Rumsey^8^, Heinrike Schmeling^9^, Karine Toupin-April^10^, Jaime Guzman^1^ and CAPRI Registry Investigators

###### ^1^Pediatrics, UBC, Vancouver; ^2^Pediatrics, McMaster University, Hamilton; ^3^Pediatrics, Western University, London; ^4^Pediatrics, McGill University, Montreal, ^5^Pediatrics, University of Toronto, Toronto; ^6^Pediatrics, Dalhousie University, Halifax; ^7^Pediatrics, Universite Laval , Quebec; ^8^Pediatrics, University of Alberta, Edmonton; ^9^Pediatrics, University of Calgary, Calgary; ^10^Pediatrics, University of Ottawa, Ottawa, Canada

####### **Correspondence:** Jaime Guzman

*Pediatric Rheumatology 2024*, **22(2)**: PReS24-ABS-1050

**Introduction:** Fatigue, pain interference and physical disability impact quality of life in children with juvenile idiopathic arthritis (JIA), but their extent and relationships have not been fully described in a population-based inception cohort representative of all JIA disease categories.

**Objectives:** 1) to quantify the relationships between fatigue, pain interference, and physical disability in children newly diagnosed with JIA and 2) to test whether fatigue mediates the relationship between pain interference and physical disability.

**Methods:** Patients enrolled within 3 months of JIA diagnosis in the Canadian Alliance of Pediatric Rheumatology Investigators (CAPRI) Registry between February 2017 and May 2023 were included. Their parents completed the Patient-Reported Outcomes Measurement Information System (PROMIS) fatigue and pain interference parent proxy short questionnaires (where 50 is the mean of the general pediatric population and 10 is the SD) and the Childhood Health Assessment Questionnaire (CHAQ) disability index (from 0= no disability to 3=severe disability) at registry enrollment. Associations were assessed using Pearson’s correlations and multiple linear regression. Structural equation modeling (SEM) was used to test if fatigue mediates the relationship between pain interference and physical disability.

**Results:** Among 855 patients (61.4% female, 44.1% with oligoarthritis), most reported fatigue and pain interference scores similar to the reference population, but 15.6% reported severe fatigue (score>65) and 7.3% reported severe pain interference (score>65) at the time of enrollment with wide variation across JIA categories. Children with polyarthritis RF-positive JIA had the highest frequency of severe fatigue and pain interference while children with oligoarthritis had the lowest. Fatigue was strongly correlated with pain interference (r = 0.72, *p* < 0.001) and with physical disability (*r* = 0.60, *p* < 0.001). Pain interference (*b* = 0.027, *p* < 0.001) and fatigue (*b* = 0.013, *p* < 0.001) were both associated with physical disability after controlling for each other and potential confounders. SEM analysis supported that fatigue partially mediates the relationship between pain interference and physical disability.

**Conclusion:** Our findings suggest both fatigue and pain interference independently influence physical disability in children shortly after the diagnosis of JIA, and the effect of pain interference is partly mediated by fatigue. Addressing fatigue and pain interference in the early stages of JIA may mitigate physical disability.

The CAPRI Registry was supported by grants from The Arthritis Society Canada [CAPRI-15-001 SOG 18-0352] and the Canadian Institutes of Health Research [PJT-175235].

**Trial registration identifying number:** Not applicable

**Patient Consent**

Not applicable (there are no patient data)

**Disclosure of Interest**

None Declared

### JIA (oligo, poly, psoriatic)

#### P187 Risk factors for cardiovascular events are increased in adults with juvenile idiopathic arthritis-results from the south-swedish juvenile idiopathic arthritis cohort

##### Helena Tydén^1, 2^, Alma Dahlberg^2, 3, 4^, Elisabet Berthold^1, 2^, Tobias Schmidt^1, 3, 4^, Morteza S. Najibi^1^, Fredrik Kahn^2, 5^, Anki Mossberg^3, 4^, Robin Kahn^2, 3, 4^

###### ^1^Department of Rheumatology, Clinical Sciences Lund, Lund University, Lund; ^2^Skåne University Hospital , Lund and Malmö; ^3^Department of Pediatrics, Clinical Sciences Lund, Lund University; ^4^Wallenberg Center for Molecular Medicine; ^5^Section of Infectious Medicine, Clinical Sciences Lund, Lund University, Lund, Sweden

####### **Correspondence:** Helena Tydén

*Pediatric Rheumatology 2024*, **22(2)**: PReS24-ABS-1295

**Introduction:** Arterial stiffness is a predictor of cardiovascular disease (CVD)(1)_._ Altered arterial properties have been found in adults with juvenile idiopathic arthritis (JIA), but data are limited and conflicting(2-4).

**Objectives:** To investigate the cardiovascular risk profile in adult JIA patients with up to 44 years disease duration from a validated JIA cohort from southern Sweden.

**Methods:** Patients diagnosed with JIA between 1980-1993 were recruited from a pre-existing population-based JIA cohort in southern Sweden. Augmentation index (AI), a measure of arterial stiffness and reactive hyperemia index (RHI), a measure of endothelial function, were assessed with Endopat(5). Non-parametric Mann-Whitney U-test and rank biserial correlation as effect size (r) were used to compare groups.

**Results:** Adult individuals with JIA (n=103, female 77.7%, oligoarthritis 49.5%, ANA positive 48.5%, mean age 42 years, mean disease duration 35 years), as well as age- and sex-matched healthy controls (n=56) were included. Individuals with JIA exhibited higher BMI (p=0.009, r=0.250), elevated diastolic blood pressure (p<0.001, r=0.328), augmentation index (p=0.009, r=0.252), and systolic blood pressure (p=0.057, r=0.183) compared to age- and sex-matched healthy controls, all showing small to medium effect sizes. No significant difference between the groups was seen for the reactive hyperemia index (p=0.640, r=0.046).

**Conclusion:** Elevated augmentation index, a predictor of cardiovascular disease, was found in adult individuals with JIA, compared to healthy controls. Furthermore, the JIA patients had higher BMI and blood pressure. This indicates that adult individuals with JIA have an increased risk of cardiovascular disease compared to their healthy peers. Early predictors of cardiovascular disease in individuals with JIA need to be identified and managed to prevent future morbidity.

**Patient Consent**

Not applicable (there are no patient data)

**Disclosure of Interest**

None Declared

**References**

1. Mitchell GF, et al. Circulation. 2010;121(4):505-11.
2. Aulie HA, et al. Ann Rheum Dis. 2015;74(8):1515-21.
3. Aulie HA, et al. Pediatr Rheumatol Online J. 2018;16(1):85.
4. Evensen K, et al. J Rheumatol. 2016;43(4):810-5.
5. Tyden H, et al. RMD Open. 2017;3(2):e000508.

### JIA (oligo, poly, psoriatic)

#### P188 The efficiency of educational programs for parents of children with Juvenile idiopathic arthritis: first results

##### Polina Lototskaya^1^, Vladislav Sevostyanov^2^, Saniya Valieva^3^, Anastasiya Glazyrina^4^, Seda Kurbanova^3^, Elena Zholobova^1^

###### ^1^Department of Childhood Diseases; ^2^Department of Propaedeutics of Childhood Diseases, I.M. Sechenov First Moscow State Medical University; ^3^Department of Rheumatology, Moscow City Center of Pediatric Rheumatology, Morozov Children's City Clinical Hospital; ^4^Federal Children's Rehabilitation Center "Korablik", Russian Children's Clinical Hospital of the Ministry of Health of Russia, Moscow, Russian Federation

####### **Correspondence:** Polina Lototskaya

*Pediatric Rheumatology 2024*, **22(2)**: PReS24-ABS-1244

**Introduction:** Awareness of patients about their chronic disease significantly increases adherence to therapy, improving the course and prognosis of diseases. This research describes the first results of conducting schools for parents of children with juvenile idiopathic arthritis (JIA) in Moscow.

**Objectives:** Development and evaluation of the effectiveness of educational programs for parents of children with JIA in the school format.

**Methods:** Development of a school program for parents of children with JIA based on the expert opinion of leading pediatric rheumatologists and the wishes of listeners; development of a questionnaire and conducting a survey of parents to assess the level of knowledge about the disease before and after school; organization of regular classes; analysis of the results obtained.

**Results:** In the implementation of this project, meetings are held between rheumatologists and parents on a monthly basis, regular meetings with a psychologist are held in face-to-face and online formats, specialized specialists are invited. The following topics are discussed in the lesson: what is JIA, causes of development, variants of the course of the disease; diagnosis, rheumatoid eye disease, complications, prognosis of JIA; methods of treatment of JIA; the importance of adherence to therapy; features of vaccination, the basics of physical therapy and rehabilitation of children with JIA and other issues. By the present moment, 9 full-time schools have been held. 95 parents of children with JIA have been trained. The most exciting topics for parents were:

- What is the prognosis of the disease?
- The value of physical therapy. What rehabilitation methods can help with the diagnosis of JIA?
- Are there any nutritional features that affect the course of JIA?
- How to inject drugs? How to prevent side effects of therapy?
- Is it possible to relax by the sea? How does the climate affect the course of the JIA?
- How to maintain remission, what factors provoke an exacerbation?

Before school, 84-75% of parents wanted to know the answers to these questions. After school, the vast majority of parents (91-94%) understood these questions (p < 0.001). According to the results of the questionnaire, the general awareness of parents about the disease before attending school was 22%. After attending school, 94% of the same questions were clear to parents. Also, an online information channel has been created for the constant support of parents, where posts on topical issues about the disease are published, training webinars are held, and answers to parents' questions are regularly published.

**Conclusion:** Education of parents of children with JIA has important medical and socio-economic importance. The results of the parent’s survey before and after school training differ significantly in the level of awareness about the basics of JIA. Participation in school can significantly increase children's adherence to therapy and improve the prognosis of the disease, reducing the burden on medical staff.

**Patient Consent**

Yes, I received consent

**Disclosure of Interest**

None Declared

### JIA (oligo, poly, psoriatic)

#### P189 Retinal microvasculature in pediatric patients with juvenile idiopathic arthritis using optical coherence tomography angiography

##### Elena Nedealcova^1, 2^, Olga Gaidarji^1^, Rodica Eremciuc^1, 2^, Valeriu Cusnir^3^, Ninel Revenco^1, 2^

###### ^1^Pediatrics, SUMPh "Nicolae Testemitanu"; ^2^Pediatric Clinic N1, Mother and Child Healthcare Institute; ^3^Ophtalmology and Optometry, SUMPh "Nicolae Testemitanu", Chisinau, Moldova, Republic of

####### **Correspondence:** Olga Gaidarji

*Pediatric Rheumatology 2024*, **22(2)**: PReS24-ABS-1283

**Introduction:** Juvenile idiopathic arthritis (JIA) is a chronic autoimmune disease affecting children, often leading to ocular complications such as uveitis. Optical coherence tomography angiography (OCTA) has emerged as a valuable tool for the non-invasive assessment of retinal microvasculature alterations in various ocular diseases. However, its utility in evaluating retinal microvasculature changes in pediatric JIA patients remains understudied.

**Objectives:** This study aims to investigate the relationship between disease activity and retinal microvasculature alterations detected through OCTA in pediatric patients with JIA.

**Methods:** A prospective study was conducted involving 60 children diagnosed with JIA who underwent comprehensive rheumatologic and ophthalmologic evaluations, including OCTA imaging. Disease activity was assessed using the Juvenile Arthritis Disease Activity Score (JADAS10), with patients categorized into minimal disease activity (n = 31) and moderate/severe activity (n = 29) groups. OCTA images were acquired using a device from Optopol, Poland. Vascular density of the superficial and deep capillary plexuses (SCP/DCP), foveal avascular zone (FAZ) area, and central macular thickness (CMT) were quantitatively analyzed and compared between the two groups.

**Results:** The mean age of the participants was 10.78 ± 4.2 years, with a mean onset age of JIA at 7.26 ± 4.3 years. The majority of patients were girls (65%). Oligoarthritis was the most common subtype (60%). Vascular density of SCP/DCP in the fovea was lower in patients with moderate/severe disease activity compared to those with minimal activity (13.8 ± 4.1% vs. 17.3 ± 3.7% for SCP, and 28.8 ± 3.7% vs. 31.7 ± 3.03% for DCP). The FAZ area was larger in patients with moderate/severe activity compared to those with minimal activity (0.39 ± 0.14 mm² vs. 0.23 ± 0.05 mm²). Additionally, CMT was increased in patients with moderate/severe disease activity compared to those with minimal activity (301.4 ± 169.1 μm vs. 237.5 ± 25.8 μm).

**Conclusion:** Our findings suggest that OCTA is a valuable tool for evaluating retinal microvascular changes in pediatric patients with JIA. Patients with moderate/severe disease activity exhibit significant alterations in retinal microvasculature, including decreased vascular density and increased FAZ area and CMT, highlighting the potential of OCTA in monitoring ocular manifestations of JIA.

**Date of birth::** juin 20, Y

**Patient Consent**

Yes, I received consent

**Disclosure of Interest**

None Declared

**References**

1. Sheila T. Angeles-Han,Sarah Ringold,Timothy Beukelman 2019 American College of Rheumatology/Arthritis Foundation Guideline for the Screening, Monitoring, and Treatment of Juvenile Idiopathic Arthritis–Associated Uveitis, 25 April 2019, 10.1002/acr.23871
2. Ayman G. Elnahry, Lameece M. Hassan, Walaa Abdelrahman, Mai N. Abd Elmohsen, Optical coherence tomography angiography (OCTA) findings in juvenile idiopathic arthritis, The Egyptian Rheumatologist,Volume 45, Issue 1, 2023, Pages 105-110, ISSN 1110-1164. 10.1016/j.ejr.2022.11.010. (https://www.sciencedirect.com/science/article/pii/S1110116422001284)

### JIA (oligo, poly, psoriatic)

#### P190 The relationship between femoral cartilage thickness, disease activity and quality of life in juvenile idiopathic arthritis patients

##### Hatice Melisa Kaçmaz^1^, Mazlum S. Akaltun^2^, Ayşe Balat^1^

###### ^1^Paediatric Rheumatology; ^2^Department of Physical Medicine and Rehabilitation, Gaziantep University Faculty of Medicine, Gaziantep, Türkiye

####### **Correspondence:** Hatice Melisa Kaçmaz

*Pediatric Rheumatology 2024*, **22(2)**: PReS24-ABS-1725

**Introduction:** Juvenile idiopathic arthritis (JIA) is a chronic autoimmune disease characterized by joint involvement in children under the age of 16, which is heterogeneous and multifactorial. If left untreated, JIA can lead to irreversible joint and tendon damage. Although the etiology is unknown, triggered immune response can cause enzymatic proteolysis leading to cartilage damage. This damage is associated with pain, functional limitations, and a worse quality of life.

**Objectives:** The aim of this study is to investigate the relationship between the thickness of femoral cartilage measured by ultrasound method and clinical and sociodemographic findings in patients with JIA

**Methods:** This study was planned as a cross-sectional case-control study. A total of 44 patients diagnosed with Juvenile Idiopathic Arthritis (JIA) according to the *International League of Associations for Rheumatology (ILAR 2001) classification* criteria who applied to the Gaziantep University Pediatric Rheumatology outpatient clinic were included, along with a healthy control group matched for age and gender. Sociodemographic information such as age, gender, body mass index (BMI) was queried and recorded for the patients and control group included in the study. In the patient group, the subgrups of JİA, *Juvenile Arthritis Disease Activity Score (JADAS-27)* for disease activity assessment,*Childhood Health Assessment Questionnaire (CHAQ)* scores for quality of life assessment, physician and family*Visual Analog Scale (VAS) for* pain assessment were queried and recorded. In addition, erythrocyte sedimentation rate (ESR), C-reactive protein (CRP), anti-citrullinated peptide (CCP), rheumatoid factor (RF) values of the patients were recorded from their files. Measurement of femoral cartilage thickness using ultrasound method was performed on the same day by the same pediatric rheumatologist in a blinded manner.

**Results:** Of the 44 JIA patients included in the study, 13 (30.2%) were male, 30 (69.8%) were female, with a mean age at diagnosis of 10.35 ± 4.27 years and a mean disease duration of 3.49 ± 2.72 years. When the control and patient groups included in the study were compared, no significant differences were observed in parameters such as age, gender, body mass index (p>0.05). Twenty-three patients (53.5%) in the patient group had oligoarticular type, while 20 (46.5%) had polyarticular type. 31 out of 44 patients were receiving biological treatment. Femoral cartilage thickness was significantly lower in the patient group compared to the control group (p<0.05). When the patient group was compared according to disease activity, femoral cartilage thickness was significantly lower in the group with high disease activity (p<0.05). There was a significant correlation between femoral cartilage thickness and BMI, JADAS, CHAQ score, family VAS, physician VAS. A negative correlation was observed between the duration of the disease and femoral cartilage thickness (p<0.05). There was no significant relationship between femoral cartilage thickness and gender (p>0.05). No significant difference was observed in terms of cartilage thickness between the right and left extremities (p>0.05).

**Conclusion:** To our knowledge, our study is the first to evaluate femoral cartilage thickness in JIA patients. Femoral cartilage thickness was found to be lower in JIA patients compared to the healthy group. When compared by disease activity, femoral cartilage thickness was found to be lower in the group with higher activity. The results of this study indicate that distal femoral cartilage thickness in JIA patients may be associated with disease activity.

**Date of birth::** 01.01.1984

**Patient Consent**

Not applicable (there are no patient data)

**Disclosure of Interest**

None Declared

**References**

1. Naredo E, Acebes C, Möller I, Canillas F, de Agustín JJ, de Miguel E et al (2009) Ultrasound validity in the measurement of knee cartilage thickness. Ann Rheum Dis 68(8):1322–1327
2. Yildirim A, Önder ME, Özkan D. Ultrasonographic evaluation of distal femoral and talar cartilage thicknesses in patients with early rheumatoid arthritis and their relationship with disease activity. Clin Rheumatol 41(7):2001-2007.

### JIA (oligo, poly, psoriatic)

#### P191 Evaluation of bone health in patients diagnosed with juvenile idiopathic arthritis

##### Ümran Çakıroğlu^1^, Özlem Akgün^2^, Selen Duygu Arik^2^, Beyza Eliuz Tipici^3^, Firdevs Baş^4^, Nuray Aktay Ayaz^2^

###### ^1^Division of Pediatrics; ^2^Division of Pediatric Rheumatology; ^3^Nutrition and Dietetics; ^4^Division of Pediatric endocrinology, Istanbul University Istanbul Faculty of Medicine, Istanbul, Türkiye

####### **Correspondence:** Selen Duygu Arik

*Pediatric Rheumatology 2024*, **22(2)**: PReS24-ABS-1730

**Introduction:** Children with chronic illnesses often face challenges that compromise their bone health, including reduced physical activity, nutritional deficiencies, malabsorption, inadequate calcium and vitamin D intake, systemic inflammation, elevated cytokine levels, and certain medications.

**Objectives:** The aim of our retrospective study was to evaluate bone health in patients with Juvenile Idiopathic Arthritis (JIA). We evaluated clinical, biochemical and radiologic findings related to bone health and examined the effects of treatments on bone health while identifying associated problems.

**Methods:** This study was designed retrospectively. In this study, 53 JIA patients under 18 years of age diagnosed according to ILAR criteria were analyzed. Patient records from the Department of Pediatrics, Department of Rheumatology, IU Medical Faculty were analyzed for various parameters including demographic characteristics, clinical history, disease activation score, biochemical markers, imaging results, treatments and bone mineral density (BMD) measured by dual-energy X-ray absorptiometry (DEXA). A nutritional questionnaire was also administered. Patients were categorized according to JIA subgroups, age groups, gender, BMD measurements, disease activation score and medications.

**Results:** Among the 53 patients (26 boys, 27 girls), the average age at JIA diagnosis was 152.7 ± 42.7 months. Oligoarticular JIA was the most common subtype (69.8%), followed by polyarticular JIA (18.9%), systemic JIA (5.7%), and enthesitis-related arthritis (5.7%). Pain was the predominant complaint, with a small percentage reporting fractures (9.4%) or back pain (5.7%). Consanguineous marriage was noted in 15.1% of cases, and a family history of rheumatic disease was present in 35.8%.In terms of treatment, 58.5% had previously received steroid therapy, while most received conventional disease-modifying drugs after diagnosis (79.2%). DEXA scans showed that 45.3% of patients had osteopenia and 15.1% had osteoporosis. There was no significant difference in BMD between children who received steroid treatment and those who did not. Vitamin D deficiency (≤12 ng/ml) was detected in 20.8% of patients. Treatment protocols did not show a significant relationship with BMD Z score. However, a moderate positive correlation was found between BMD Z score and 25 (OH) D3 levels (r=0.317; p<0.05).

**Conclusion:** Our study shows that children with JIA have a significant decrease in bone mass and a significant rate of osteopenia and osteoporosis. Disease severity did not correlate with decreased BMD, but a positive correlation was observed between BMD Z score and 25(OH)vitamin D3 levels. Adequate vitamin D and calcium intake is crucial for maintaining bone health in these patients.

**Date of birth::** octobre 30

**Patient Consent**

Not applicable (there are no patient data)

**Disclosure of Interest**

None Declared

### JIA (oligo, poly, psoriatic)

#### P192 Is the Juvenile arthritis damage index suitable for predicting the effectiveness and monitoring of biological therapy in non-systemic variants of JIA?

##### Zarina Kolkhidova, Irina Nikishina, Svetlana Glukhova

###### V. A. Nasonova Research Institute of Rheumatology, Moscow, Russian Federation

####### **Correspondence:** Irina Nikishina

*Pediatric Rheumatology 2024*, **22(2)**: PReS24-ABS-1402

**Introduction:** Juvenile idiopathic arthritis (JIA) has a high risk of joint damage, extra-articular effects, and disability progression. The CHAQ and X-ray are common methods to evaluate functional impairment, but The Juvenile Arthritis Damage Index (JADI) could be a better tool for predicting and monitoring JIA therapy outcomes in clinical settings.

**Objectives:** To assess how the JADI corresponds with clinical signs in JIA patients (pts), who didn`t receive biologics (B) previously and to determine if JADI values can predict response to B.

**Methods:** This open prospective study included 150 in-patient children with non-systemic JIA variants with no history of B. The choice of the drug was determined in the usual manner, as is customary in real clinical practice. The mean age was 12.2±4.6 years, 60% were girls. A total of 112 pts were examined at 6 or 12 months following the onset of B. All pts underwent clinical evaluations, including detailed joint assessments and scoring with JADI-A and JADI-E.

**Results:** The study discovered that half of the biological-naive pts had damage according to the JADI score, with 43% showing JADI-A«+» and 23% showing JADI-E«+». Both JADI-A and JADI-E were present in 15% (23) of pts. Among those with JADI-A, the most common issues were flexion contractures in the knee joints (39%), elbow joints (28%), and limited movement in the cervical spine (26%). Extra-articular damage were prevalent such as avascular necrosis of bones (41%) and significant differences in limb length (35%). Articular changes were linked to positive results for ANA, RF, and ACCP, as well as the absence of enthesitis, high ESR and CRP levels, high clinical activity, JADAS71, CHAQ values, history of glucocorticoid therapy, and poor adherence to treatment. The onset of polyarthritis affecting small hand joints and a tendency for rapid contracture formation were also associated with JADI-A positivity.

Under the B as part of routine clinical practice, most pts were able to achieve a stable condition with no signs of damage, or their damage index score didn’t change. Out of the pts, 26 (23%) was improvements in their JADI-A scores, with contractures resolving completely or joint movement increasing. In 10 (9%) cases, there was progression of lesions, but this was not associated with a specific drug choice, including 6 (5%) children who had new damage despite targeted therapy. At the second assessment point, a significant correlation between activity and damage indices was established: JADAS71 of low grade in 4.9% corresponded to JADI«+», and JADAS71 of high grade in 46.3% corresponded to JADI«+» (p<0.01). This proves the potential for using JADI as a tool to assess the efficacy of targeted therapy.

**Conclusion:** The JADI is a useful and accessible tool for clinical assessment of articular and extra-articular lesions, reflecting the prognosis of JIA and being an objective indicator of therapy efficacy. A large proportion of pts with damage require simultaneous, rather than sequential, administration of biological and synthetic DMARD, especially in patients with poor prognosis factors.

**Patient Consent**

Yes, I received consent

**Disclosure of Interest**

None Declared

### JIA (oligo, poly, psoriatic)

#### P193 Incidence of Juvenile idiopathic arthritis in Finland, 2000–2020

##### Erika Uusitupa^1, 2^, Heidi Rahikkala^2, 3^, Sirja Sard^3, 4, 5^, Tytti Pokka^3, 6^, Henri Salo^7^, Johanna Kärki^8^, Tuulikki Sokka-Isler^9^, Maria Backström^3, 10^, Paula Vähäsalo^3, 4, 5^

###### ^1^Pediatrics, University of Turku; ^2^Pediatrics, Turku University Hospital, Turku; ^3^Research Unit of Clinical Medicine, University of Oulu; ^4^Children and Adolescents, Oulu University Hospital; ^5^Medical Research Center Oulu, Oulu University Hospital and University of Oulu; ^6^Research Service Unit, Oulu University Hospital, Oulu; ^7^Knowledge Brokers Department, Finnish Institute for Health and Welfare, Helsinki; ^8^Children and Adolescents, Wellbeing Services County of Kanta-Häme, Hämeenlinna; ^9^Hospital Nova of Central Finland, Wellbeing Services County of Central Finland, Jyväskylä; ^10^Pediatrics, Wellbeing Services County of Ostrobothnia, Vaasa, Finland

####### **Correspondence:** Erika Uusitupa

*Pediatric Rheumatology 2024*, **22(2)**: PReS24-ABS-1044

**Introduction:** The last epidemiological data of JIA in Finland are from the turn of the millennium, including restricted study periods and geographical coverage (1-4).

**Objectives:** The aim of this study was to assess the recent annual incidence of JIA in several consecutive years in Finland across different patient groups, subtypes and hospital districts.

**Methods:** All children <16 years of age who met the ILAR classification criteria for JIA were analyzed. Cases from 2000-2020 were identified from two national registers: the Care Register for Health Care of the Finnish Institute for Health and Welfare and the Reimbursement Register containing medication data from the Social Insurance Institution of Finland; cases from 2016-2020 were identified from the Finnish Rheumatology Quality Register. Joinpoint regression analysis on a logarithmic scale was used to detect increasing or decreasing trends in incidence over follow-up time segments. The annual percentage change (APC) was used to characterize trends in the incidence rate of JIA.

**Results:** The incidence of JIA was 31.7 per 100,000 (95% CI 30.2, 33.1), according to the Care Register in 2000-2020. Around the year 2012, the incidence peaked to 37.6 per 100,000 (95% CI 33.8, 41.7), but the annual incidence was similar at the end (28.7 per 100,000; 95% CI 25.3, 32.3) of the study period compared to the beginning (29.3 per 100,000; 95% CI 26.0, 32.8). No considerable differences in incidence rates were observed among registers. In all age groups, incidence in girls was predominant compared to boys. The incidence in girls peaked at the ages of 2 years (78.8 per 100,000; 95% CI 71.8, 86.3) and 14 years (50.7 per 100,000; 95% CI 45.4, 56.6). Decreasing incidence was observed among boys 0-3 years old during the entire study period (APC -2.5%; 95% CI -4.5%, -0.5%; p=0.016), whereas increasing incidence was observed in 2000-2013 among teenage girls (APC 5.7%; 95% CI 2.8%, 8.6%; p=0.001) and 4-7 years old boys (APC 3.8%; 95% CI 1.3%, 6.4%; p=0.006).

**Conclusion:** The incidence of JIA in Finland is globally high and higher than previously reported. Regional and annual variations in incidence of JIA were observed, yet the overall incidence remained stable at the end of the study period.

**Patient Consent**

Not applicable (there are no patient data)

**Disclosure of Interest**

None Declared

**References**

1. Berntson L, Andersson Gäre B, Fasth A, Herlin T, Kristinsson J, Lahdenne P, et al. Incidence of juvenile idiopathic arthritis in the Nordic countries. A population based study with special reference to the validity of the ILAR and EULAR criteria. J Rheumatol. 2003;30:2275–82.
2. Kaipiainen-Seppänen O, Savolainen A. Incidence of chronic juvenile rheumatic diseases in Finland during 1980-1990. Clin Exp Rheumatol. 1996;14:441–4.
3. Kaipiainen-Seppänen O, Savolainen A. Changes in the incidence of juvenile rheumatoid arthritis in Finland. Rheumatology (Oxford). 2001;40:928–32.
4. Savolainen E, Kaipiainen-Seppänen O, Kröger L, Luosujärvi R. Total incidence and distribution of inflammatory joint diseases in a defined population: results from the Kuopio 2000 arthritis survey. J Rheumatol. 2003;30:2460–8.

### JIA (oligo, poly, psoriatic)

#### P194 The role of childhood infectious diseases in the exacerbation of Juvenile idiopathic arthritis in patients in Moscow

##### Denis Rassokha^1^, Vladislav Sevostyanov^1^, Nikita Babich^1^, Elena Zholobova^2^

###### ^1^Department of Propaedeutics of Childhood Diseases; ^2^Department of Childhood Diseases, I.M. Sechenov First Moscow State Medical University, Moscow, Russian Federation

####### **Correspondence:** Denis Rassokha

*Pediatric Rheumatology 2024*, **22(2)**: PReS24-ABS-1176

**Introduction:** Patients with juvenile idiopathic arthritis (JIA) are at high risk for mass infections, and in the case of acute infection, basic antirheumatic drugs are usually canceled, which leads to the exacerbation of the underlying disease and the progression of the pathological process.

**Objectives:** To investigate the correlation between childhood infections and disease exacerbations in patients aged 0-17 with JIA in Moscow.

**Methods:** We conducted an observational analytical cross-sectional study that included 395 patients with juvenile idiopathic arthritis. We performed statistical data processing using IBM SPSS Statistics 26 and Microsoft Excel 2019. We presented qualitative features as absolute numbers (n) with corresponding percentages (%). We assessed the normality of data distribution using the Kolmogorov-Smirnov test, Shapiro-Wilk test, kurtosis, and skewness indicators, as well as histogram analysis. We used the Pearson Chi-squared test to compare three or more groups of categorical variables. We set the statistical significance at a p-value of 0.05.

**Results:** The analysis of childhood infections in patients after the onset of JIA has been performed. Chickenpox accounts for the majority of cases, comprising 20.5% of the total number (n=81). Other diseases represent a smaller proportion of the overall structure, with whooping cough and measles accounting for 0.8% each (n = 3). One child had rubella, accounting for 0.3%. In 77.7% of patients (n= 307), no childhood infections were recorded during the follow-up period following the onset of JIA. In total, 22.3% (n=88) of the subjects had childhood infections. When assessing the relationship between childhood infectious diseases and the occurrence of JIA exacerbations, it was found that the patients who had suffered from an infection after the onset of JIA were twice as likely to have a recurrence of the underlying condition. In other words, children who had not suffered from any childhood infectious diseases worsened 2 times less often. We found that an exacerbation of the underlying disease was detected in 37.2% (n=147) of patients. Of these, 37.4% had a childhood infection. Statistically significant differences were found between the two groups (p < 0.001). The chances of exacerbation were 4,035 times higher in the group with a history of childhood infection compared to those without infections (95% confidence interval: 2,449–6,649).

**Conclusion:** According to the results of the study, vaccination significantly reduces the frequency of exacerbations of the underlying chronic disease. Patients who did not suffer an infection in childhood are 2 times less likely to experience an exacerbation of juvenile idiopathic arthritis. Thus, vaccination plays a crucial role in the treatment of rheumatoid arthritis, as almost a quarter of JIA patients experience one or two childhood infections before reaching adulthood.

**Patient Consent**

Yes, I received consent

**Disclosure of Interest**

None Declared

### JIA (oligo, poly, psoriatic)

#### P195 Clinical insights into heterogeneity of rheumatoid factor negative polyarticular juvenile idiopathic arthritis across the world

##### Roberta Naddei^1^, Marco Burrone^2, 3^, Francesca Ridella^2, 3^, Yosef Uziel^4^, Maria Trachana^5^, Pavla Dolezalova^6^, Ingrida Rumba-Rozenfelde^7^, Nicolino Ruperto^3^, Marco Gattorno^3^, Angelo Ravelli^2, 3^, Alessandro Consolaro^2, 3^

###### ^1^Dipartimento di Scienze Mediche Traslazionali, Università degli Studi di Napoli Federico II, Naples; ^2^Dipartimento di Neuroscienze, Riabilitazione, Oftalmologia, Genetica e Scienze Materno Infantili (DINOGMI), Università degli Studi di Genova; ^3^IRCCS Istituto Giannina Gaslini, Genoa, Italy; ^4^Meir Medical Centre and Kfar Saba and Sackler School of Medicine, Tel Aviv University, Tel Aviv, Israel; ^5^Paediatric Immunology and Rheumatology Referral Centre, Hippokration General Hospital, School of Medicine, Aristotle University of Thessaloniki, Thessaloniki, Greece; ^6^Centre for Paediatric Rheumatology and Autoinflammatory Diseases, Charles University and General University Hospital in Prague, Prague, Czech Republic; ^7^University of Latvia, Pediatric Rheumatology, Riga, Latvia

####### **Correspondence:** Marco Burrone

*Pediatric Rheumatology 2024*, **22(2)**: PReS24-ABS-1209

**Introduction:** Limited information is available about the differences in the characteristics of rheumatoid factor (RF)-negative polyarticular juvenile idiopathic arthritis (JIA) throughout the world.

**Objectives:** To compare the demographic and clinical features of patients with RF-negative polyarthritis across the world, in order to gain further insights into the geographic variability of this JIA subtype.

**Methods:** Data were extracted from a dataset of 9,081 subjects with JIA from 49 countries enrolled in the Epidemiology, treatment and Outcome of Childhood Arthritis study (1). All patients underwent a retrospective data collection and a cross-sectional assessment. Demographic and clinical data were compared across 8 geographic areas (northern Europe, western Europe, southern Europe, eastern Europe, North America, Latin America, Africa and Middle East, and southeast Asia). Dunn’s test and Bonferroni correction were used for post-hoc analysis.

**Results:** 2,141 patients (23.6%) with RF-negative polyarthritis were included in the analysis. The prevalence of RF-negative polyarthritis was highest in North America (31.5%) and lowest in southeast Asia (12.7%). Age of onset showed a biphasic distribution in all areas. Northern and southern Europe presented the highest prevalence of patients with a disease onset before 6 years of age. Those two areas presented a significantly higher prevalence of uveitis (21.1% and 14.2%), as compared to western and eastern Europe (9.6% and 8%) and North America (9.1%), and to Southeast Asia (4.2%), Africa & Middle East (4.1%) and Latin America (1.8%). ANA positivity was more frequent in western and southern Europe (51% and 62.7%). The proportion of patients treated with conventional disease-modifying antirheumatic drugs (DMARDs) ranged from 75.8% (North America) to 93.8% (Southeast Asia). Biological DMARDs were used mostly in northern Europe and North America and less frequently in southeast Asia. The prevalence of active joints at the cross-sectional visit resulted significantly lower in northern and southern Europe, and higher in eastern Europe. Subjects from southern Europe presented less frequently pain, morning stiffness and impairment of overall well-being, quality of life and function ability. The frequency of inactive disease resulted higher in patients from southern Europe. Subjects from Africa and Middle East and eastern Europe showed the highest scores of composite disease activity measures. Patients with early disease onset and ANA positivity, whose association resulted higher in southern Europe, presented a higher prevalence of uveitis but better outcomes in terms of joint count, physician and parent global assessments, physical function, quality of life, and disease activity.

**Conclusion:** Our results confirm the wide heterogeneity of the clinical presentation and outcome of children with RF-negative polyarticular JIA throughout the world. Patients with early disease onset and ANA positivity, predominant in southern Europe, showed a higher prevalence of uveitis but better outcomes in terms of joint disease. Further studies are needed to assess genetic and environmental factors underlying these findings.

**Patient Consent**

Not applicable (there are no patient data)

**Disclosure of Interest**

None Declared

**Reference**

1. Consolaro A, Giancane G, Alongi A, van Dijkhuizen EHP, Aggarwal A, Al-Mayouf SM, et al. Phenotypic variability and disparities in treatment and outcomes of childhood arthritis throughout the world: an observational cohort study. Lancet Child Adolesc Health. 2019;3:255-63.

### JIA (oligo, poly, psoriatic)

#### P196 Screening for comorbid autoimmune disease should be considered in children with ANA positive juvenile idiopathic arthritis – results from the South-Swedish Juvenile idiopathic arthritis cohort

##### Alma Dahlberg^1, 2, 3^, Helena Tydén^2, 4^, Anna Jöud^5, 6^, Fredrik Kahn^2, 7^, Elisabet Berthold^2, 4^

###### ^1^Department of Pediatrics, Clinical Sciences Lund, Lund University, Lund; ^2^Skåne University Hospital, Lund and Malmö; ^3^Wallenberg Center for Molecular Medicine; ^4^Department of Rheumatology, Clinical Sciences Lund; ^5^Department of Laboratory Medicine, Division of Occupational and Environmental Medicine, Lund University; ^6^Department of Research and Education, Skåne University Hospital; ^7^Section of Infectious Medicine, Clinical Sciences Lund, Lund University, Lund, Sweden

####### **Correspondence:** Alma Dahlberg

*Pediatric Rheumatology 2024*, **22(2)**: PReS24-ABS-1274

**Introduction:** There are no consensus or clinical guidelines for screening routines of autoimmune diseases in patients with juvenile idiopathic arthritis (JIA), since results are conflicting whether the risk for such conditions is increased (1-5).

**Objectives:** To investigate the occurrence- and the need for screening routines for comorbid autoimmune conditions in JIA by using a validated population-based JIA cohort from southern Sweden.

**Methods:** Autoimmune comorbidities were evaluated in a pre-existing population-based JIA cohort of 302 participants, constituting of individuals diagnosed with a validated JIA diagnosis 2000–2010 in southern Sweden. The comorbidities were determined through analysis of diagnosis codes registered after JIA diagnosis until 2019. Two registered diagnosis codes at separate outpatient healthcare visits or one registered inpatient diagnosis code was considered as a verified comorbidity. With the use of a reference population of 1510 age- and sex matched individuals, hazard ratios (HR) were calculated with Cox proportional models, and to further explore potential predictors for comorbid conditions, subgroup analyses of patient characteristics were performed.

**Results:** During the study period, 7.7% of the JIA cohort received an autoimmune diagnosis after their JIA diagnosis. JIA patients had an increased risk of autoimmune diseases in general (HR 2.01, 95% CI 1.16-3.51), as well as separately for celiac disease (HR 3.98, 95% CI 1.44-11.01) compared to the reference population. Antinuclear antibody (ANA) positivity as well as treatment with disease-modifying anti-rheumatic drugs (DMARD) was associated with a significantly increased risk of comorbid autoimmune disease in the JIA cohort.

**Conclusion:** JIA patients have a significantly increased risk of acquiring an autoimmune disease after receiving their JIA diagnosis compared to matched references. ANA positivity and treatment with DMARD are associated with a further increased risk. Our results emphasize awareness in physicians of additional autoimmune disorders in JIA patients and advocate serological screening of autoimmune conditions during follow-up, potentially preventing morbidity and the adverse effects of untreated, undiagnosed disease.

**Patient Consent**

Not applicable (there are no patient data)

**Disclosure of Interest**

None Declared

**References**

1. Öman A, et al. Acta Paediatr. 2019;108(4):688-93.
2. Schulz C, et al. Pediatr Rheumatol Online J. 2022;20(1):8.
3. Simon TA, et al. Pediatr Rheumatol Online J. 2020;18(1):43.
4. Tronconi E, et al. Ital J Pediatr. 2017;43(1):56.
5. Pohjankoski H, et al. Scand J Rheumatol. 2010;39(5):435-6.

### JIA (oligo, poly, psoriatic)

#### P197 Glucose metabolism in Juvenile idiopathic arthritis

##### Maria Francesca Gicchino, Marica Esposito, Annalisa Barlabà, Margherita Luciano, Alma N. Olivieri, Emanuele Miraglia del Giudice, Anna Di Sessa

###### University of the Study of Campania L. Vanvitelli, Naples, Italy

####### **Correspondence:** Maria Francesca Gicchino

*Pediatric Rheumatology 2024*, **22(2)**: PReS24-ABS-1321

**Introduction:** Juvenile idiopathic arthritis (JIA) represents a chronic inflammatory disease in childhood. From a pathophysiological point of view, the underlying immune dysregulation and inflammatory cytokines overproduction in JIA resembled those observed in adult rheumatoid arthritis (RA). As a consequence, chronic inflammation leads to insulin-resistance (IR), in turn resulting in glucose metabolism abnormalities. Although there is evidence in adults with RA for an increased risk of type 2 diabetes (T2D) and metabolic syndrome, similar data in children with JIA are still scarce.

**Objectives:** To investigate glucose metabolism in a cohort of children with JIA.

**Methods:** One-hundred thirty-nine children diagnosed with JIA classified according to the International League of Association for Rheumatology (ILAR) criteria attending our Rheumatology Clinic were retrospectively examined. Patients with acute conditions (e.g. severe infections, trauma, exacerbation of chronic disease) or diagnosed with type 1 diabetes or T2D prior to the study enrollment were excluded.

A detailed anthropometric and biochemical evaluation including homeostasis model assessment of insulin resistance (HOMA-IR) and *hemoglobin* A1c (HbA1c) assessment was performed in all the enrolled subjects. IR was defined according to HOMA-IR cut-offs for sex and pubertal stage. Disease activity was calculated by using the Juvenile Arthritis Disease Activity Score 10 (JADAS-10) joint reduced count, and cut-offs for disease states were applied. Based on JADAS-10 score, four categories of disease status such as “inactive disease”, “low disease activity”, “moderate disease activity”, and “high disease activity” were identified.

According to BMI-SDS quartiles, patients were divided into four groups. At the examination of the study population at the time of JIA diagnosis, none of patients received any pharmacological treatment.

**Results:** No differences for sex, age at disease onset, Tanner stage, and disease duration across the groups were found (all p>0.05). A trend for JADAS-10 score across BMI-SDS quartiles was observed (p=0.06), but disease activity did not significantly differ (p=0.09).

Compared to patients in the lower quartiles, those belonging to the highest quartile showed increased systolic and diastolic blood pressure, alanine transaminase (ALT), fasting insulin, and low-density lipoprotein (LDL) levels (p=0.01, p=0.01, p=0.01, p=0.008, and p=0.04, respectively). As inflammation markers, ferritin, C-reactive protein, and erythrocyte sedimentation rate levels did not significantly differ across BMI-SDS quartiles (all p >0.05).

Out of 139, 7 patients (5%) showed a HbA1c value between 5.7-6.5% and 3 (2.1%) had impaired fasting glucose as prediabetes phenotypes.

As cardiometabolic risk markers, they also presented with increased uric acid and tryglycerides (TG)/high-density lipoprotein (HDL) ratio values (p=0.03 and p=0.04, respectively). Both HOMA-IR, and HbA1c values significantly increased across BMI-SDS quartiles (p=0.028 and p=0.026, respectively). A higher percentage of subjects with IR was found in the highest quartile compared to others (p=0.03). Patients belonging to the highest quartile showed an adjusted odds ratio (OR) to show IR of 1.78 (95% CI 1.07-2.94, p=0.025).

**Conclusion:** Overweight/obese children diagnosed with JIA showed an overall unfavorable cardiometabolic risk profile. These patients also presented with an increased risk of IR and prediabetes. Given the higher risk of developing T2D, cardiovascular disease, and metabolic syndrome overtime, a careful monitoring of glucose metabolism should be warranted in children with JIA. Further longitudinal studies in the field are required to confirm these important findings for pediatric cardiometabolic health in children with JIA.

**Patient Consent**

Yes, I received consent

**Disclosure of Interest**

None Declared

### JIA (oligo, poly, psoriatic)

#### P198 Evaluation of ilar and printo classifications for juvenile idiopathic arthritis: oligoarticular JIA vs early-onset ANA positive JIA

##### Batuhan Küçükali^1^, Çisem Yıldız^1^, Buğra T. Gülle^2^, Deniz Gezgin Yıldırım^1^, Sevcan A. Bakkaloğlu^1^

###### ^1^Pediatric Rheumatology, Gazi University, Faculty of Medicine, Ankara; ^2^Public Health, Division of Epidemiology, Dokuz Eylül University, Faculty of Medicine, İzmir, Türkiye

####### **Correspondence:** Batuhan Küçükali

*Pediatric Rheumatology 2024*, **22(2)**: PReS24-ABS-1467

**Introduction:** International League of Associations for Rheumatology (ILAR) juvenile idiopathic arthritis (JIA) classification revisited by Pediatric Rheumatology International Trials Organization (PRINTO) in 2018. Classifications should provide uniform groups to assist physicians to provide optimal care.

**Objectives:** We evaluated changes proposed by PRINTO to highlight their impact on forming consistent groups regarding uveitis and treatment responses, particularly focusing on early-onset anti-nuclear antibody (ANA) positive JIA.

**Methods:** Pediatric patients diagnosed with JIA according to ILAR and PRINTO classification, with a minimum one-year follow-up, were enrolled, excluding those meeting both oligoarticular JIA and early-onset ANA positive JIA groups’ exclusion criteria.

**Results:** Among 139 enrolled patients, 110 (79.1%) had oligoarticular JIA, while 15 (10.8%) had early-onset ANA-positive JIA. Below age 5 criteria showed the strongest association with uveitis, while below age 7 provided similar associations without substantial exclusions (Odds ratio 8.62 [2.50-29.81] vs 7.45 [2.37-26.66]). Patients with single ANA positivity at a titer ≥ 1/160 and below age 7 had a notably higher risk of new-onset uveitis and biologic DMARDs requirement (Odds ratio 7.95 [2.37-26.66] and 3.6 [1.42-9.09], respectively).

**Conclusion:** Inclusion of age of disease onset and ANA positivity with a titer ≥ 1/160 has enhanced uniformity in uveitis risk and treatment response, including conventional synthetic DMARDs failure. A single ANA positivity at a ≥ 1/160 titer yields similar or better results, while the involved joint count criteria failed to form consistent groups. PRINTO's classification places a significant portion of patients into the “other JIA” group, necessitating further classification for improved clinical utility.

**Date of birth::** mars 17, Y

**Patient Consent**

Not applicable (there are no patient data)

**Disclosure of Interest**

None Declared

### JIA (oligo, poly, psoriatic)

#### P199 Unravelling juvenile idiopathic arthritis subtypes: insights from synovial fluid proteomic signatures

##### Narendra K. Bagri^1^, Saumya Srivastava^1^, Yogendra Singh^2^, Thirumurthy Velpandian^2^, Ashish Upadhyay^2^, Venkatesan S. Kumar^2^, Subhradip Karmakar^2^, Sushil K. Kabra^2^

###### ^1^Division of Pediatric Rheumatology, Department of Pediatrics; ^2^All India Institute of Medical Sciences , New Delhi , India

####### **Correspondence:** Narendra K. Bagri

*Pediatric Rheumatology 2024*, **22(2)**: PReS24-ABS-1623

**Introduction:** Synovial fluid (SF) seems an ideal fluid for studying the pathogenic mechanisms of Juvenile Idiopathic Arthritis (JIA). Employing an Isobaric tag for relative and absolute quantitation (iTRAQ) coupled with Liquid chromatography-mass spectrometry (LC-MS/MS), we conducted a proteomic analysis of SF from 48 participants with JIA and healthy controls to delineate the proteomic signatures in various subtypes of JIA.

**Objectives:** To investigate subtype-specific protein expression patterns in JIA through proteomic analysis of SF.

**Methods:** SF samples from 48 participants (3 healthy controls and 45 JIA patients with various subtypes) were analysed. Proteins were extracted, labelled with iTRAQ for quantification, and analysed via LC-MS/MS. Differential protein expression analysis identified significant variations between control and JIA subtype samples^1^. A more than 5-fold change (FC) in protein expression compared to controls was considered upregulated. Gene Ontology (GO) and Kyoto Encyclopedia of Genes and Genomes (KEGG) pathway enrichment analysis elucidated associated biological pathways upregulated proteins^2^.

**Results:** Our study identified over 282 SF proteins exhibiting differential expression in various subtypes of JIA. Polyarticular JIA displayed 14 SF proteins, with four unique proteins (including Pyruvate dehydrogenase phosphate). Enthesitis related arthritis revealed 18 upregulated proteins, eight unique (including Haptoglobin-related and Fibrinogen proteins). Systemic juvenile idiopathic arthritis exhibited 19 upregulated proteins, eleven unique (including FH1/FH2 domain, Mitochondrial potassium channel, Apolipoprotein and Nck-associated protein etc.), and Oligoarticular JIA showed 4 upregulated proteins, three unique (including Leucine-rich alpha-2-glycoprotein). Proteins S-100, Septin 7 MMP-16, etc, were commonly upregulated in all subtypes of JIA except Oligo. However, haptoglobin was found to be upregulated in all except sJIA. GO pathway analysis of these upregulated proteins showed enriched patterns largely to the immune response and complement activation-related biological processes. The KEGG pathway enrichment suggested that upregulated proteins may play an important role in the complement activation and regulation of the actin cytoskeleton.

**Conclusion:** Our study revealed significant protein profile changes in JIA, highlighting its diversity. We identified subtype-specific and shared upregulated proteins. The enriched immune, complement, and actin cytoskeleton pathways underscored their relevance in the pathogenesis of JIA.

**Patient Consent**

Yes, I received consent

**Disclosure of Interest**

None Declared

**References**

1. Balakrishnan L, Bhattacharjee M, Ahmad S, et al. Differential proteomic analysis of synovial fluid from rheumatoid arthritis and osteoarthritis patients. Clin Proteomics. 2014;11(1):1. 2014 Jan 6. doi: 10.1186/1559-0275-11-1
2. Tang D, Chen M, Huang X, Zhang G, Zeng L, Zhang G, Wu S, Wang Y. SRplot: A free online platform for data visualization and graphing. PLoS One. 2023 Nov 9;18(11) doi: 10.1371/journal.pone.0294236

### JIA (oligo, poly, psoriatic)

#### P200 Laboratory predictors of jia disease course: a pilot study

##### Ilaria Maccora^1, 2^, Maria Moriondo^3, 4^, Edoardo Marrani^1^, Maria Vincenza Mastrolia^1, 2^, Ilaria Pagnini^1^, Chiara Azzari^3, 4^, Gabriele Simonini^1, 2^

###### ^1^Rheumatology Unit, Meyer Children's Hospital IRCCS; ^2^NeuroFARBA Department, University of Florence; ^3^Pediatric Immunology Unit, Meyer Children's Hospital IRCCS; ^4^Department of Health Sciences,, University of Florence, Florence, Italy

####### **Correspondence:** Gabriele Simonini

*Pediatric Rheumatology 2024*, **22(2)**: PReS24-ABS-1369

**Introduction:** Juvenile Idiopathic Arthritis (JIA) is the most common chronic rheumatic disease in childhood. Thanks to the steps forward in understanding the pathogenesis, several drugs have been developed and successfully used. However, it is not clear yet, how to identify patients who can achieve disease inactivity and might benefit of a therapy rather than another and can timely stop the treatment.

**Objectives:** To identify specific standard lymphocyte subpopulations (LS) predictors of disease course.

**Methods:** In a monocentric prospective observational study at the Rheumatology and Immunology Unit of our Hospital, we involved children with a diagnosis of JIA according to ILAR, with a follow-up of at least 6 months, who underwent to LS and/or immunoglobulin dosage. We collected demographics, clinical (JADAS10), laboratory and treatment data. We performed immunophenotyping by flow cytometry for standard LS(CD3+, CD3+CD4+, CD3+CD8+, CD19+, CD16+CD56+).

**Results:** We enrolled 40 patients (pts)(29 female 72.5%, 35 ANA + 87.5%), with a median age at onset of 4.5 years (Range 0.75-15.16), and median follow-up of 42 months (R 6-175). Nineteen pts had oligoarticular JIA(47.5%), 15 polyarticular JIA(37.5%), 4 psoriatic arthritis(10%), 2 ERA(5%), and 7 uveitis(17.5%). At the last available follow-up 4 pts were on remission off-therapy(10%), 4 active(A) off-therapy(10%), 6 A-on therapy(15%), 26 inactive(I) on-therapy(65%).

The median of IgG at onset was 1074 mg/dl (R661-2361), of IgM 107.5 mg/dl (R40-283) and IgA 98.2 mg/dl (R0-554).

LS were performed at onset in 7 (16.3%) and during disease in 36 (83.7%) after a median time of 28 months (R0-140).

At LS test, 21 pts were I (48.8%, 10 on therapy (23.3%), 7 pre-withdrawal (16.3%), 2 after withdrawal (4.7%)) and 22 A (51.2%, 7 at onset (16.3%), 12 on therapy (27.9%), 3 off-therapy(7%)). LS showed a median of % and absolute value of CD3+ of 73 (46-83) and 1770(421-3567), of CD3+CD4+ of 42(27-61) and 1057(255-2249), of CD3+CD8+ of 21(12-36) and 574(116-1069), of CD4/CD8 1.9(1-3.6), of CD19+ 19(9-44) and 444(147-1804), and of CD3-CD16+CD56+ 8(4-19) and 207(77-867).

We observed significant differences in CD4+% (p0.032, I 43% vs A 39.5) and CD4+/CD8+ (p 0.023, 2.2 vs1.75) and the inflammatory status at the time of LS. Pts who achieved/maintain I after LS showed significant differences in the distribution of CD8% (p0.036, A 27 vs I 20) and CD4/CD8 (p0.012, A 1.3 vs I 2). Moreover, in the sub-analysis of the 22 pts persistently A at the time of LS, we observed significant differences if they later achieved or not I, in the distribution of CD8% (p0.02, 20 vs 28), CD8 absolute value (p0.032, 584 vs 838) and CD4+/CD8+ (p0.02, 1.95 vs 1.3). Considering the 22 pts still A at LS, 10 children who then achieved I disease on anti-TNF had lower value of CD8+ (574 vs 838 p 0.028), and higher value of CD19% (22.5 vs 11, p 0.042) compared to 3 children who did not achieve I.

Overall, no significant differences were observed regarding the dosage of IgG, IgA and IgM performed at onset and the inflammatory status at the last available follow-up.

**Conclusion:** Our pilot study reported specific LS that might help to identify JIA children on treatment who will achieve than disease inactivity. Conversely, due to the sample size of pts who stop the treatment, and overall the short follow-up, no specific LS have been identified to predict disease course after treatment withdrawal.

**Patient Consent**

Yes, I received consent

**Disclosure of Interest**

None Declared

### JIA (oligo, poly, psoriatic)

#### P201 Exploring children's perspectives on the Juvenile arthritis support program (Jasp-1) **-** insights from qualitative interviews

##### Karina Mördrup^1, 2^, Cecilia Bartholdson^1, 3^, Eva W. Broström^1, 3^, Johanna G. Jungner^1^

###### ^1^Women's and children's health, Karolinska Institutet; ^2^Astrid Lindgrens Childrens Hospital, Rheumatology Unit; ^3^Astrid Lindgrens Childrens Hospital, Karolinska University Hospital, Stockholm, Sweden

####### **Correspondence:** Karina Mördrup

*Pediatric Rheumatology 2024*, **22(2)**: PReS24-ABS-1388

**Introduction:** In Sweden, 200 children are being diagnosed with Juvenile Idiopathic Arthritis (JIA) every year and approximately 2000 children are living with JIA^1^. Information given in connection to the time of diagnose can for many families be difficult to remember^2^ and many families’ experiences shortage of information^3^. Therefore, families require support and repeated information to feel secure and guided^4^. A one-year Juvenile Arthritis Support Program (JASP-1) was therefore developed, consisting of seven structured patient- and family-centered visits during the child´s first year with JIA.

**Objectives:** The aim of this study was to explore children’s experiences of participating in JASP-1.

**Methods:** Children 8-17 years old who had participated in JASP-1 were invited to participate in semi-structured telephone interviews. An interview guide with open questions was used letting the children tell their story and express their experiences with participating in JASP-1. The interviews were, after consent from the child, sound recorded and later transcribed verbatim. Data was analyzed using qualitative content analyses^5^.

**Results:** Interviews were conducted between September 2020 and November 2022 with nine girls and five boys, aged between 12-17 years old. The interviews lasted between 8-20 minutes. After preliminary analyses of the interviews three main categories emerged: 1) Good balance between contact, visits, and school, 2) Safety through professional support and information and 3) Safety through treatment and pain relief.

**Conclusion:** The children’s experiences with JASP-1 exposed that they experienced that the program was well planned with visits, not interfering with school and that JASP-1 gave them a sense of safety by providing support, information, treatment, and pain relief.

**Patient Consent**

Not applicable (there are no patient data)

**Disclosure of Interest**

None Declared

**References**

1. https://barnreumaregistret.se/for-patienter/information-om-barnreumatism/
2. on S and Sule S. Living with Juvenile Idiopathic Arthritis: Parent and Physician Perspectives. *Rheumatology and therapy*. 2018; 5: 1-4.
3. Yuwen, W., Lewis, F. M., Walker, A. J., & Ward, T. M. (2017). Struggling in the Dark to Help My Child: Parents' Experience in Caring for a Young Child with Juvenile Idiopathic Arthritis. *J Pediatr Nurs*, *37*, e23-e29. 10.1016/j.pedn.2017.07.007
4. izen EHP, Egert T, Egert Y, et al. Patient's experiences with the care for juvenile idiopathic arthritis across Europe. *Pediatr Rheumatol Online J*. 2018; 16: 10.
5. Lindgren BM, Lundman B, Graneheim UH. Abstraction and interpretation during the qualitative content analysis process. Int J Nurs Stud. 2020;108:103632.

### JIA (oligo, poly, psoriatic)

#### P202 Selection of the second biologic agent for Juvenile idiopathic arthritis and its effectiveness: a retrospective cohort study

##### Emilio Amleto Conti^1, 2^, Maria Rosa Pellico^1, 3^, Stefania Costi^3^, Francesco Baldo^3^, Sabino Germinario^1, 3^, Claudia Iannone^1, 3^, Andrea Amati^1, 3^, Marco Pandolfi^1, 3^, Maurizio Gattinara^3^, Cecilia Chighizola^1, 3^, Cecilia Eboli^1, 2^, Sofia Mazzitelli^1, 2^, Giovanni Filocamo^2^, Francesca Minoia^2^, Roberto Caporali^1, 3^, Achille Marino^3^

###### ^1^University of Milan; ^2^Pediatric Rheumatology, Fondazione IRCCS Ca' Granda Ospedale Maggiore Policlinico; ^3^Pediatric Rheumatology, ASST G. Pini-CTO, Milan, Italy

####### **Correspondence:** Emilio Amleto Conti

*Pediatric Rheumatology 2024*, **22(2)**: PReS24-ABS-1303

**Introduction:** TNF-α inhibitors (TNFi) are frequently used as first-line treatment in juvenile idiopathic arthritis (JIA), while there are no clear recommendations on the choice of the second biologic agent (bDMARD).

**Objectives:** to describe the prescription pattern and effectiveness of the second bDMARD in a cohort of non-systemic JIA patients.

**Methods:** This retrospective cohort study included non-systemic JIA patients treated with at least two bDMARDs followed in two Rheumatology Pediatric tertiary centers in Milan, Italy. Analyses were performed with SPSS statistics.

**Results:** The study cohort included 118 (91 F) patients: 59 oligoarticular, 48 polyarticular JIA, and the remaining were psoriatic JIA and ERA (11 together). The median age at onset and at the 1^st^ bDMARD was 4.78 [interquartile range (IQR) 6.43] and 9.12 (IQR 9.88) years, respectively.

Etanercept and adalimumab were the most frequently prescribed 1^st^ bDMARD (63 and 39 times, respectively). The causes of the first bDMARD discontinuation were articular flare (54.2%), uveitis flare (21.1%), and adverse events (19.4%). The median age at the 2^nd^ bDMARD was 13.79 (IQR 9.02) years. Most of the patients in the cohort (73.9%) received a TNFi as the second bDMARD. Adalimumab was the most prescribed 2^nd^ bDMARD (49.1%), followed by etanercept (14.4%). A non-TNFi was prescribed as the 2^nd^ bDMARD in 30 patients (25%): 8 subjects received abatacept, and 17 subjects were treated with tocilizumab.

94%, 78.8% and 46.6% of the patients received metothrexate respectively before, at 1^st^ bDMARD and 2^nd^ bDMARD onset.

51 patients (43.2%) discontinued the 2^nd^ bDMARD: 66.6% due to articular flare, 17.6% due to uveitis flare, and 15.6% due to adverse events.

At 6 months, 81.3% and 69.4% of patients achieved clinical inactive disease (CID) on 1^st^ and 2^nd^ line TNFi (p value=0.406).

Active arthritis at 6 months was seen in 11 (19.6%) and 17 (30.3%) patients on 1^st^ and 2^nd^ TNFi, respectively (p value= 0.0208).

There was no statistically significant difference in the rates of active uveitis at 6 months between the 1st and 2nd TNFi treatments (8 patients (14%) vs 4 (7%), p value=0.161).

The retention time of the 2^nd^ bDMARD was longer than the 1^st^ bDMARD (2.36 vs. 1.74 years; p=0.221).

**Conclusion:** The majority of patients received a TNFi as their first and second bDMARD. There was no statistical difference in the retention time between the first and second bDMARD. No significant differences were observed in achieving CID, active arthritis, and uveitis at 6 months between the 1^st^ and 2^nd^ TNFi.

**Patient Consent**

Yes, I received consent

**Disclosure of Interest**

None Declare

### JIA (oligo, poly, psoriatic)

#### P203 Ultrasound patterns of ankle tenosynovitis in patients with Juvenile idiopathic arthritis

##### Federica A. Vianello^1^, Federica Plebani ^1^, Chiara Provini ^1^, Fabiana Di Stasio^1^, Elisabetta Lo Iudice ^1^, Martina Rossano^1^, Sofia Mazzitelli ^1^, Giovanni Filocamo^1, 2^, Stefano Lanni ^1^

###### ^1^Fondazione IRCCS Ca' Granda Ospedale Maggiore Policlinico; ^2^Università degli Studi di Milano, Milan, Italy

####### **Correspondence:** Federica A. Vianello

*Pediatric Rheumatology 2024*, **22(2)**: PReS24-ABS-1743

**Introduction:** The ankle is frequently affected in juvenile idiopathic arthritis (JIA). This region has a complex anatomical structure because of the presence of multiple joint recesses and adjacent tendon compartments. Both joints and tendons may become inflamed in JIA. The increasing use of ultrasound (US) as a complement tool to clinical evaluation of ankles with clinical arthritis has shown that in several cases ankle swelling is related to tendon inflammation.

**Objectives:** To evaluate US features of tenosynovitis in ankles with clinical arthritis of patients with JIA

**Methods:** Ankles with evidence on clinical examination of arthritis were scanned by a physician (SL) with experience in the US assessment of children with JIA. The US protocol included the evaluation of the tibiotalar joint (TTJ), the subtalar joint (STJ), the intertarsal joint (ITJ)(talonavicular and navicular–first cuneiform joints assessed together), the anterior tendon compartment (ATC), the medial tendon compartment (MTC), and the lateral tendon compartment (LTC) of the ankle. Descriptive statistics were performed.

**Results:** Fifty clinically active ankles of 38 patients with JIA were included in the study. Seventeen ankles (34.0%) showed on US synovitis in a unique joint compartment. This finding was concomitant to the detection on US of tenosynovitis in 7/17 (41.2**%**) ankles: in 6 cases tendon inflammation affected only a single tendon compartment (4 in the MTC and 2 in the LTC); in the remaining ankle US documented tenosynovitis in 2 tendon compartments (MTC and LTC). US-detected synovitis was found in 2 joint compartments in 11/50 (22.0%) ankles: 8 of them (72.7**%**) had on US also tendon inflammation which affected a single tendon compartment in 5 cases (2 in the MTC and 3 in the LTC, respectively); inflammation involving both the MTC and LTC was found on US in the remaining 3 ankles with tenosynovitis on US. The 15/50 (30.0%) ankles with US-determined synovitis in all the 3 different joint compartments of the ankle (TTJ, STJ, and TNJ) showed tenosynovitis on US in around two third of cases: in the majority of them (6 ankles) tendon inflammation was documented on US as affecting more than one tendon compartment. Seven ankles did not show synovitis on US, but in 6 of them tenosynoivitis was documented on US.

**Conclusion:** Tenosynovitis is a frequent finding on US in ankles with clinical arthritis of patients with JIA. Detection on US of inflammation in multiple tendon compartments seems to be more common in ankles with US-detected synovitis affecting concomitantly different joint recesses of the ankle region.

**Patient Consent**

Yes, I received consent

**Disclosure of Interest**

None Declared

### JIA (oligo, poly, psoriatic)

#### P204 Characterization of fibroblast-like synoviocytes isolated from the synovial fluid of patients affected by Juvenile idiopathic arthritis

##### Federica Briasco^1^, Federica Raggi^1^, Chiara Rossi^1^, Chiara Artale^1^, Marco Gattorno^1^, Alessandro Consolaro^1^, Angelo Ravelli^2^, Maria Carla Bosco^1^, Simone Pelassa^1^

###### ^1^UOC Rheumatology and Autoinflammatory Diseases; ^2^Scientific Direction, IRCCS Istituto Giannina Gaslini, Genova, Italy

####### **Correspondence:** Federica Briasco

*Pediatric Rheumatology 2024*, **22(2)**: PReS24-ABS-1547

**Introduction:** Although the role of immune cells in Juvenile Idiopathic Arthritis (JIA) pathogenesis has been extensively characterized, the understanding of synovial cells remains limited. Fibroblast-like Synoviocytes (FLS) have been investigated in the synovial membrane (SM) or fluid (SF) of adults affected by arthritides and in murine arthritis models. Two subsets of FLS, lining (LL) and sublining (SL), were identified: the former involved in cartilage degradation and the latter in immune response regulation. Furthermore, FLS demonstrated multipotent properties and chondrocyte-like differentiation potential. To date, the characterization of different FLS subtypes and their multipotent properties in JIA remains unexplored.

**Objectives:** This study was aimed at characterizing FLS isolated from the SF of JIA patients with active disease, assessing their membrane antigen profile, gene expression patterns, and chondrogenic potential.

**Methods:** FLS were isolated from the SF of 10 JIA patients with active disease. Skin fibroblasts (sFB) from 4 healthy donors were used as control. Cytofluorimetric analysis was performed after staining with anti-CD45, CD34, PDPN, and THY antibodies. Gene expression was evaluated using RT-PCR under both basal and pro-inflammatory conditions (treatment with pro-inflammatory stimuli, LPS or TNFα). Chondrogenic differentiation was induced by culturing pelleted cells in differentiation medium for 28 days, followed by fixation and staining with Alcian Blue.

**Results:** Cytofluorimetric analysis revealed CD45-CD90+PDPN+ phenotype in FLS from SF of active JIA patients, similarly to what reported for FLS in the SM sublining regions of adult arthritic patients. RT-qPCR showed that, upon LPS and TNF stimulation, FLSs displayed increased expression of the pro-inflammatory cytokines (IL1β, IL6, IL8, CCL5, CXCL5, MCP-1), indicating their potential involvement in inflammatory responses. Similar or lower levels of metalloproteinase expression was observed in SF-derived FLS compared to sFBs, suggesting low tissue degradation ability. In addition, SF-derived FLS exhibited higher levels of expression of chondrocyte-associated genes (BMP-4, Aggrecan) compared to sFBs and were able to differentiate into chondrocyte-like cells, as confirmed by Alcian-Blue staining.

**Conclusion:** Our study demonstrated that FLS from active JIA patients resemble SL FLS identified in SM of adult arthritis patients, potentially contributing to joint inflammation. Furthermore, SF-derived FLSs can differentiate into chondrocyte-like cells, suggesting their value as a model for studying JIA chondrocytes and predicting disease severity, as recently suggested in the literature.

**Patient Consent**

Not applicable (there are no patient data)

**Disclosure of Interest**

None Declared

### JIA (oligo, poly, psoriatic)

#### P205 The prevalence and risk factors for methotrexate intolerance in patients with Juvenile idiopathic arthritis

##### Stefan A. Djordjevic ^1, 2^, Gordana Susic^1^, Dragana Lazarevic^3, 4^, Dusica Novakovic^5^, Smiljka Kovacevic^2^, Predrag Ostojic^5^

###### ^1^Division of Pediatric Rheumatology, University Children’s Hospital; ^2^Faculty of Medicine, University of Belgrade, Belgrade; ^3^Department of Pediatric Rheumatology, Clinic of Pediatrics, University Clinical Center Nis; ^4^Faculty of Medicine, University of Nis, Nis; ^5^Institute of Rheumatology, Belgrade, Serbia

####### **Correspondence:** Stefan A. Djordjevic

*Pediatric Rheumatology 2024*, **22(2)**: PReS24-ABS-1650

**Introduction:** Methotrexate (MTX) intolerance can have a negative impact on treatment adherence, treatment outcome and quality of life.

**Objectives:** To estimate the prevalence and identify potential risk factors for MTX intolerance in patients with juvenile idiopathic arthritis (JIA).

**Methods:** We conducted a cross-sectional study at a large pediatric rheumatology referral center between July 2019 and July 2021. Pediatric patients aged up to 19 years with JIA receiving chronic MTX therapy (at least 3 months) were eligible for the study. Exclusion criteria were a diagnosis of systemic JIA and MTX toxicity. Demographic and clinical data as well as data on MTX use were collected. MTX intolerance was assessed using the Methotrexate Intolerance Severity Score (MISS).^1^ Patients with and without MTX intolerance were compared using the Mann-Whitney U test, the chi-square test or the Fisher exact test. The Pearson correlation coefficient was used to examine the relationship between age at disease onset and age at initiation of MTX treatment.

**Results:** A total of 94 patients were enrolled in the study. The median age of all participants was 9.9 years (range 2.3–18.5 years), and 69 (73.4%) were female. The median age at disease onset was 3.7 years (range 0.8–16.4 years) and the median duration of disease was 3.6 years (range 0.6–14 years). The prevalence of MTX intolerance was 24.5%. The most common symptom of MTX intolerance was nausea after taking MTX (38.3% of all patients), while 48.9% of patients had some of the behavioral problems. There was a statistically significant difference in age at disease onset (U=577.0, p=0.04) and at initiation of MTX treatment (U=555.5, p=0.02) and in the use of MTX as first-line treatment (X^2^=5.78, p=0.02) between MTX-tolerant and MTX-intolerant patients. Age at disease onset and age at initiation of MTX treatment were strongly correlated (r=0.9, p<0.001). Patients receiving subcutaneous MTX and those receiving higher MTX doses had an increased risk of MTX intolerance, although no statistical significance was reached (p=0.09 and U=532.0, p=0.09, respectively).

**Conclusion:** The prevalence of MTX intolerance in patients with JIA is relatively high (24.5%). Nausea after taking MTX is the most common complaint, but behavioral symptoms are also common. Older age at the onset of the disease and at initiation of MTX therapy as well as MTX as first-line therapy are potential risk factors for MTX intolerance.

**Patient Consent**

Not applicable (there are no patient data)

**Disclosure of Interest**

None Declared

**Reference**

1. Bulatović M, Heijstek MW, Verkaaik M, Van Dijkhuizen EHP, Armbrust W, Hoppenreijs EPA, et al. High prevalence of methotrexate intolerance in juvenile idiopathic arthritis: Development and validation of a methotrexate intolerance severity score. Arthritis Rheum. 2011;63(7):2007–2013.

### JIA (oligo, poly, psoriatic)

#### P206 Epidemiology of juvenile idiopathic arthritis in central russia

##### Vladislav Sevostyanov^1^, Denis Rassokha^2^, Nikita Babich^2^, Polina Lototskaya^3^, Elena Zholobova^3^

###### ^1^Department of Propaedeutics of Children's Diseases; ^2^Department of Propaedeutics of Childhood Diseases; ^3^Department of Childhood Diseases, I.M. Sechenov First Moscow State Medical University, Moscow, Russian Federation

####### **Correspondence:** Vladislav Sevostyanov

*Pediatric Rheumatology 2024*, **22(2)**: PReS24-ABS-1130

**Introduction:** Juvenile idiopathic arthritis (JIA) is a chronic autoimmune disorder that is characterized by joint inflammation of unclear etiology. Assessing the epidemiological data of JIA is one of the important areas of pediatrics; information about the prevalence allows to plan the necessary resources for the diagnosis and treatment of patients.

**Objectives:** To establish the main trends and features of the prevalence and incidence of juvenile arthritis for the period from 2012 to 2021 years and determine the influence of organizational factors on epidemiological indicators.

**Methods:** We analyzed official statistic data of the prevalence and incidence of JIA from 18 regions of Central Russia over 10-year follow-up period (2012-2021). The influence of the staffing of pediatricians and rheumatologists on epidemiological indicators was assessed. Statistical analyses was performed using application packages IBM SPSS Statistics 26. To describe quantitative data with a distribution other than normal, the median and quartiles were calculated [Q1; Q3]. Correlation analysis was carried out according to Spearman.

**Results:** In 2021 year 7 218 858 children (0-17 years old) lived in 18 regions of Central Russia, the median for 10-year period was 6 867 006 (Q1-Q3: 6 372 192-7 102 226), a positive trend of 124.508.2. During the follow up period (2012–2021), a slight increase in JIA incidence was established with the median of 13 cases per 100 000 children (Q1–Q3: 13–15) and the positive trend (+0.26). According to monitoring data of the prevalence of JIA, an increase was noted from 53.1 to 83.3 cases per 100 000 children with a trend of +3.4. Since a primary diagnosis, as well as further monitoring of patients with rheumatic diseases is carried out by pediatricians, a relationship between the organization of pediatric care and the level of general and primary JIA morbidity was evaluated. Weak correlation has been established. If the indicator “Number of pediatricians per 10,000 children (0–17 years)” increases by 1, we should expect a decrease in the indicator “Prevalence of JIA per 100 000 children” by 4.997 (p=0.006). A correlation analysis of the relationship between the prevalence and incidence of JIA and the indicator “Number of visits to a rheumatologist per 100 000 children (0–17 years)” was carried out. Weak correlation has been noted as well. With an increase in the indicator “Number of visits to a rheumatologist per 100 000 children (0–17 years)” by 1000, a decrease in the “Incidence of JIA per 100 000 children (1–17 years)” by 2.0 should be expected, which indicates over diagnosis of JIA at the primary care level (p=0.008).

**Conclusion:** JIA epidemiology data evaluation and identifying factors influencing epidemiological indicators is of great interest for further research in different countries and regions.

**Patient Consent**

Not applicable (there are no patient data)

**Disclosure of Interest**

None Declared

### JIA (oligo, poly, psoriatic)

#### P207 Using osmic acid as a treatment for patients with recurrent monoarthropathy of the knee in juvenile idiopathic arthritis: the Northern Ireland paediatric rheumatology experience

##### Diarmuid McLaughlin^1^, Dearbhla McKenna^2^, Cathy Campbell^2^, Michael D. Shields^3^, Madeleine Rooney ^2^

###### ^1^Paediatric Rheumatology, Great Ormond Street Hospital, London; ^2^Paediatric Rheumatology, Musgrave Park Hospital; ^3^Centre for Experimental Medicine, Queens University Belfast, Belfast, United Kingdom

####### **Correspondence:** Diarmuid McLaughlin

*Pediatric Rheumatology 2024*, **22(2)**: PReS24-ABS-1534

**Introduction:** The role of chemical synovectomy or Osmic Acid in treatment resistant joint disease in Juvenile Idiopathic Arthritis (JIA) despite repeated Intra Articular Corticosteroid Injections (IACIs) is not well-recognised or an established treatment in this patient cohort.

**Objectives:** To describe the outcome of Osmic Acid knee injections in patients with JIA who required repeated IACIs.

**Methods:** A retrospective review was undertaken of patients with JIA who received Osmic Acid (osmium tetroxide) knee injections, between December 2006 and August 2020, at the tertiary paediatric rheumatology unit in Northern Ireland. Patients were identified by analysing the local patient electronic database and their clinical notes subsequently reviewed. Data recorded was anonymised and stored securely. Each patient had serial joint ultrasounds performed before, during and after each Osmic Acid injection. Post procedure, patients were assessed at regular intervals in clinic to determine clinical response. A positive clinical response was determined as resolution of the patient’s pain, absence of swelling on clinical examination along with improvement of ultrasonic evaluation in joint effusion and synovial hypertrophy. The requirement for further IACIs was calculated before and after the first administration of Osmic Acid.

**Results:** A total of 11 patients with recurrent monoarthropathy (7 male and 4 female, mean age 13.9y (range 2 to 22y)) had a Osmic Acid joint injection. Following first administration of Osmic Acid 5 patients (46%) had no further relapses and a further 2 (18%) had a single isolated recurrence. 4 patients (36%) required a single further administration of Osmic Acid with no further recurrences following this. Prior to the use of Osmic Acid the recurrence rate (requirement for further IACIs) was 0.87 events per patient year and this reduced to 0.31 events per patient year following first administration of Osmic Acid. No complications or side effects were found following injection of Osmic Acid in any knee joint.

**Conclusion:** The role of chemical synovectomy in the modern paediatric biologic era is not well described and further study is warranted. Our initial review suggests that Osmic Acid appears to prolong or obviate the need for IACIs in JIA patients who had required frequent IACIs in the same knee joint. The current UK practice for an ongoing active monoarthopathy, despite IACIs is escalation to DMARD therapy. The use of Osmic Acid could negate the requirement for DMARDs in some patients. Furthermore, as the shortage of triamcinolone hexacetonide across many countries persists, the use of Osmic Acid could be considered.

**Patient Consent**

Not applicable (there are no patient data)

**Disclosure of Interest**

None Declared

**References**

1. Knut L. Radiosynovectomy in the therapeutic management of arthritis. World J Nucl Med. 2015; Jan-Apr;14(1):10-5. doi: 10.4103/1450-1147.150509.
2. Courtney P, Doherty M. Joint Aspiration and Injection. Best Practice & Research Clinical Rheumatology. 2005;19(3):345–369. doi: 10.1016/j.berh.2005.01.009.
3. Bessant R, Steuer A, Rigby S, Gumpel M. Osmic acid revisited: factors that predict a favourable response. Rheumatology. 2003;42:1036–1043.
4. Slobodin G, Rosner I, Boulman N, Rozenbaum M. Osmic acid synovectomy in the era of biologics. Rheumatol Int. 2008;28:303–304.

### JIA (oligo, poly, psoriatic)

#### P208 JIA T cells dispose mechanism to keep ferroptosis in check

##### Christopher Neullens^1^, Sudheendra Hebbar Subramanyam^1^;Jorg van Loosdregt^2^, Bas Vastert^2^, Kim K. Ohl^1^, Klaus Tenbrock^1^ and Translational Pediatric Rheumatology and Immunology, RWTH Aachen University Hospital, Aachen, Germany

###### ^1^Translational Pediatric Rheumatology and Immunology, Uniklinik RWTH Aachen, Aachen, Germany; ^2^Pediatric Rheumatology, UMC Utrecht, Utrecht, Netherlands

####### **Correspondence:** Klaus Tenbrock

*Pediatric Rheumatology 2024*, **22(2)**: PReS24-ABS-1173

**Introduction:** Ferroptosis has recently been identified as a novel type of regulated cell death that is driven by lipid peroxidation. Cells have evolved various defense mechanisms to detoxify these toxic lipid peroxides. Especially T cells established a fine-tuned redox-balance as reactive oxygen species are required to activate T cells, while too high amounts are deleterious for their function. We hypothesized that T cells in inflamed tissue have significantly increased needs in antioxidant defense and therefore are much more dependent on mechanism to counteract ferroptosis.

**Objectives:** The central role of inflammatory T cells in Juvenile idiopathic arthritis (JIA) is undoubtable. We therefore analyzed this mechanism in CD4+ T cells from peripheral blood and from inflamed joints of these patients.

**Methods:** Ferroptosis and ferroptosis-relevant proteins were analyzed in peripheral blood mononuclear cells (PBMC) and synovial fluid mononuclear cells (SFMC) by flow cytometry. In vitro T cell stimulation assays were performed for metabolic and functional studies.

**Results:** We could prove that Ferroptosis in CD4+ T cells is regulated by the canonical SLC7A11/Cysteine/Glutathione pathway. Sulfasalazine (SAS), which is currently used for treatment of inflammatory bowel disease and enthesitis-associated arthritis, is a SCL7A11 and Cysteine uptake inhibitor. Despite of its long-lasting therapeutic use, the immune-modulating effects are not clear. Interestingly, inhibition of SLC7A11 by SAS not only induced ferroptosis in CD4+ T cells but also dampened their proinflammatory function (IFN-g and IL-17 production), while regulatory T cell expression was enhanced. Moreover, JIA CD4+ T cells reveal a higher SLC7A11 expression and higher cystine uptake compared to peripheral blood T cells.

**Conclusion:** Our data thereby suggest that JIA T cells dispose mechanism to keep ferroptosis in check, which uncovers a new Achilles heel to treat exaggerated CD4+ T cell responses in JIA.

**Patient Consent**

Not applicable (there are no patient data)

**Disclosure of Interest**

None Declared

### JIA (oligo, poly, psoriatic)

#### P209 Paediatric rheumatology vaccination rates at the children’s hospital in Zürich – where can we improve?

##### Seraina Palmer Sarott^1^, Tatjana Welzel^2^, Elvira Cannizzaro^1^

###### ^1^Department of Paediatric Rheumatology, University Children's Hospital Zürich, Zürich; ^2^Department of Paediatric Rheumatology, University Children's Hospital Basel, Basel, Switzerland

####### **Correspondence:** Seraina Palmer Sarott

*Pediatric Rheumatology 2024*, **22(2)**: PReS24-ABS-1123

**Introduction:** Patients with rheumatic disease (PRD) might have an increased risk of infection, therefore we want to provide optimal prevention by ensuring an up to date vaccination status.^1,2^

**Objectives:** To assess the rate of complete basic vaccinations (as recommended by the Swiss Federal Office of Public Health) and flu vaccinations (season 2021) in our PRD patients. To explore factors associated with incomplete vaccination status in order to address these specifically in our everyday practice.

**Methods:** This single center observational study included children aged 1 to 18 diagnosed with PRD seen at our tertiary Pediatric Rheumatology Department between 01/2022 and 06/2022. During routine clinical visits the vaccine card was checked for baseline vaccinations and flu for our immunosuppressed (IM) and non-immunosuppressed (NIM) patients. Patient’s demographics and immunosuppressive treatment were recorded. All families were asked about factors that influenced their decision to vaccinate.

**Results:** 54 Patients were included with a mean age of 10.25 years (+/-4.06 SD). 63% were female, 66% were IM. Overall, 88.8% (48 Patients) had a complete basic vaccination status, 46.9% had received a flu vaccination. All IM had a complete basic vaccination status compared to 85% NIM (p=0.0516). More IM (61.3%) received a flu vaccination compared to NIM (27.8%; p=0.0378). Most families (IM: 96.7%, NIM: 84.2%) reported that they had discussed vaccinations with a at least one health professional (80.4% paediatrician, 30.4% paediatric rheumatologist). However, 15.9% of all patients (IM 25%) would have wished for more information regarding vaccinations. The reason for not completing the basic vaccinations was: worry of side effects (N=3). Reasons for not completing the flu vaccination were: not knowing that vaccination was recommended (N=3), worry of side effects (N=2), child was ill (N=2), vaccination not deemed necessary (N=2), no time (N=1) and general skepticism regarding vaccinations (N=2).

**Conclusion:** In this study, the majority of PRD patients (88%) had a complete basic vaccination status and particularly the IM-Group had an updated basic vaccination status (100%). However, only 2/3 of IM were vaccinated against the flu. Listening to our patients there is room for improvement: 25% of families with IM wished for more information regarding vaccinations in general. This highlights the importance of implementing regular open discussions about vaccinations in paediatric rheumatology routine care, a task for the treating rheumatologist.^2^ Information regarding safety and necessity of vaccinations must regularly be discussed, and then passed on to the paediatrician, who plays a crucial role in vaccinating children in the Swiss health system.

**Patient Consent**

Not applicable (there are no patient data)

**Disclosure of Interest**

None Declared

**References**

1. Thiele F, Klein A, Windschall D, Hospach A, Foeldvari I, Minden K, Weller-Heinemann F, Horneff G. Comparative risk of infections among real-world users of biologics for juvenile idiopathic arthritis: data from the German BIKER registry. Rheumatol Int. 2021 Apr;41(4):751-762.
2. Jansen MHA, Rondaan C, Legger GE, Minden K, Uziel Y, Toplak N, Maritsi D, van den Berg L, Berbers GAM, Bruijning P, Egert Y, Normand C, Bijl M, Foster HE, Koné-Paut I, Wouters C, Ravelli A, Elkayam O, Wulffraat NM, Heijstek MW. EULAR/PRES recommendations for vaccination of paediatric patients with autoimmune inflammatory rheumatic diseases: update 2021. Ann Rheum Dis. 2023 Jan;82(1):35-47

### JIA (oligo, poly, psoriatic)

#### P210 Infection rate as an ideal indicator of the effectiveness of vaccination in patients with juvenile idiopathic arthritis in Moscow

##### Denis Rassokha^1^, Vladislav Sevostyanov^1^, Nikita Babich^1^, Elena Zholobova^2^

###### ^1^Department of Propaedeutics of Childhood Diseases; ^2^Department of Childhood Diseases, I.M. Sechenov First Moscow State Medical University, Moscow, Russian Federation

####### **Correspondence:** Denis Rassokha

*Pediatric Rheumatology 2024*, **22(2)**: PReS24-ABS-1175

**Introduction:** Evaluating the effectiveness of vaccination in patients with juvenile idiopathic arthritis (JIA) is challenging. The ideal indicator for effectiveness, the frequency of infections, is seldom studied due to the requirement for large sample sizes.

**Objectives:** To evaluate the effectiveness of vaccination against childhood infectious diseases in patients aged 0-17 with JIA in Moscow.

**Methods:** We conducted an observational analytical cross-sectional study that included 395 patients with juvenile idiopathic arthritis. We performed statistical data processing using IBM SPSS Statistics 26 and Microsoft Excel 2019. We presented qualitative features as absolute numbers (n) with corresponding percentages (%). We assessed the normality of data distribution using the Kolmogorov-Smirnov test, Shapiro-Wilk test, kurtosis, and skewness indicators, as well as histogram analysis. We used the Pearson Chi-squared test to compare three or more groups of categorical variables. We set the statistical significance at a p-value of 0.05.

**Results:** To assess the effectiveness of vaccine prophylaxis, we compared the incidence rates among children who had received at least one vaccine for the studied childhood infections with those who had no vaccination history. Additionally, we evaluated the incidence rates between children fully vaccinated according to the preventive vaccination schedule for a specific childhood disease and those without immunization. We found that patients who had received a single vaccination against chickenpox and measles were statistically significantly less likely to contract the respective diseases compared to unvaccinated children (p < 0.047 and p < 0.006, respectively). Similarly, those adhering to the whooping cough vaccination schedule were significantly less likely to contract the disease compared to those without complete immunization (p < 0.031). However, a complete vaccination schedule for rubella, measles, and chickenpox showed no significant differences in disease incidence between vaccinated and unvaccinated children, possibly due to a low rate of complete immunization.

**Conclusion:** The study demonstrated that both partial and full immunization are effective in children with juvenile idiopathic arthritis.

**Patient Consent**

Yes, I received consent

**Disclosure of Interest**

None Declared

### JIA (oligo, poly, psoriatic)

#### P211 Comparison of immunization coverage rates among children with Juvenile idiopathic arthritis in moscow with data from the who

##### Denis Rassokha^1^, Nikita Babich^1^, Vladislav Sevostyanov^1^, Elena Zholobova^2^

###### ^1^Department of Propaedeutics of Childhood Diseases; ^2^Department of Childhood Diseases, I.M. Sechenov First Moscow State Medical University, Moscow, Russian Federation

####### **Correspondence:** Denis Rassokha

*Pediatric Rheumatology 2024*, **22(2)**: PReS24-ABS-1178

**Introduction:** Patients with juvenile idiopathic arthritis (JIA) are at an increased risk for infections due to the use of immunosuppressive medications. For this reason, it is essential for this group of patients to be prioritized for vaccination. However, vaccination rates among this vulnerable population remain low.

**Objectives:** To assess the level of immunization coverage among children aged 0-17 with JIA in Moscow and compare the data with indicators set by the World Health Organization.

**Methods:** The study included 753 children with juvenile idiopathic arthritis (JIA) living in Moscow. Immunization coverage was assessed by determining the proportion of participants who had received at least one dose of vaccine for tuberculosis, polio, diphtheria, measles, mumps, rubella, three doses of pertussis, and three doses of hepatitis B. We conducted an observational analytical cross-sectional study that included 395 patients with juvenile idiopathic arthritis. We performed statistical data processing using IBM SPSS Statistics 26 and Microsoft Excel 2019. We presented qualitative features as absolute numbers (n) with corresponding percentages (%).To describe quantitative indicators using data that follows a normal distribution, the average value is used along with the calculation of the standard error and the 95% confidence interval (95% CI). We set the statistical significance at a p-value of 0.05.

**Results:** We found that BCG vaccination occupies the leading position in terms of coverage at 91.8% (n=691). However, this level does not correspond to the high global coverage indicators set by the WHO (95%). The vaccination coverage for hepatitis B (three doses) stands at 73,7% (n=555), while that for diphtheria is- 84,9 (n=639), which also falls short of the global coverage levels set by WHO (85%). Measles vaccination coverage is - 81,0% (n=610), rubella- 78,2% (n=589), and mumps- 80,3% (n=605), all of which are higher than the WHO coverage level (70%). Polio vaccination coverage according to our study was- 88,3% (n=665), slightly higher than that recommended by WHO (85%). Pertussis immunization coverage (three doses), on the other hand, was lower at 76,9% (n=579), falling short of WHO's global coverage target of 85%.

**Conclusion:** The data from the conducted study may indicate a possible risk of outbreaks of vaccine-preventable infections among children with juvenile idiopathic arthritis. This finding indicates the need to address this emerging issue and to optimize approaches and methods for vaccinating these children.

**Patient Consent**

Yes, I received consent

**Disclosure of Interest**

None Declared

### JIA (oligo, poly, psoriatic)

#### P212 Comorbidity of juvenile idiopathic arthritis and type i diabetes: single center experience

##### Gayane Torosyan^1^, Svetlana Rodionovskaya^1, 2^, Inna Livishina^1^, Darya Grishina^3^, Ilya Zyabkin^1, 4^, Irina Nikishina^1, 5^

###### ^1^Federal Scientific and Clinical Center for Children and Adolescents of the Federal Medical and Biological Agency of Russia, ^2^State Scientific Center - Federal Medical Biophysical Center named after. A.I. Burnazyan of the Federal Medical and Biological Agency of Russia, ^3^Federal State Budgetary Institution, Federal Scientific and Clinical Center for Children and Adolescents of the Federal Medical and Biological Agency of Russia, ^4^Russian Medical Academy of Continuous Professional Education, ^5^ V.A.Nasonova Research Institute of Rheumatology, Moscow, Russian Federation

####### **Correspondence:** Irina Nikishina

*Pediatric Rheumatology 2024*, **22(2)**: PReS24-ABS-1188

**Introduction:** Juvenile idiopathic arthritis (JIA) and type I diabetes (T1D) are the most common comorbid conditions in patients with combined autoimmune pathology causing a high risk of adverse prognosis with the development of microvascular and functional disorders.

**Objectives:** To determine the prevalence of T1D in patients with JIA registered between 2018 and 2024 in the database of the our hospital, as well as to evaluate the clinical and demographic characteristics of patients with JIA and T1D, therapeutic options, mutual influence of this conditions.

**Methods:** Among of 524 patients in our database with JIA, there were 8 (1.52%) who has having T1D. A retrospective analysis of the clinical and demographic parameters, the dynamics of JADAS10 indices of JIA activity, the level of glycated hemoglobin (HbA1c), the results of therapy with disease-modifying antirheumatic drugs (DMARDs) – methotrexate (MT), leflunomide (LF), biologic DMARDs (bDMARDs, that is, adalimumab (ADA), infliximab (INF), etanercept (ETN), secukinumab (SEC)), insulin pump therapy (IPT) for the T1D control.

**Results:** The study included 8 patients with the median (Me) age of JIA onset of 9.5 years [interquartile range IQR 6.55; 14.57], T1D onset of 7.6 [6.7; 9.45], 3 boys, 5 girls. In 7 patients the development of T1D preceded juvenile arthritis by 3.0 [1.3; 4.7] years; in one patient with new-onset JIA T1D joined 3.7 years later. The duration of JIA before the first visit to a rheumatologist was 6 [3.0; 19.25] months. According to the ILAR classification, 3 patients - RF-negative polyarticular variant of JIA, 2 patients - oligoarticular JIA (one with uveitis development), 1 patient - enthesitis-related JIA (ERA), 1 patient - juvenile psoriatic arthritis. The antinuclear factor positivity was detected in 4 cases, HLA B27 antigen was detected in one case (ERA). The activity of JIA at onset according to the JADAS10 scale was of a moderate and high degree of 17.5 [9; 20.5] points for everyone. Intra-articular injections of glucocorticoids (GC) were administered to 4 patients with the development of adverse effects requiring the correction of the insulin therapy, 7 patients received MT at a dose of 15mg/m2, 1 patient - MT switched by LF, 5 patients were treated by bDMARDs - ADA, INF, ETN, and SEC. All patients were on IPT. According to the results of the observation, 6 patients reached the inactive status of the JIA disease, the HbA1c level constituted 7.25 [6.7; 8.0] %, the need for insulin ranged from 0.75 to 1.2 units/kg/day. With regard to the complications of T1D, lipodystrophies were recorded in 2 children (diabetes duration 14/8 and 18/13) and diabetic polyneuropathy in 1 (18/13).

**Conclusion:** The prevalence of T1D in patients with JIA seems to be significantly higher than in the overall population. In the majority of patients JIA started after the development of T1D, was characterized by high activity, requiring early prescription of DMARDs, biological therapy. A significant problem of the pediatric patients with JIA and T1D is the achievement of T1D compensation, while avoiding the use of any form of GC. Biological therapy for JIA, IPT, regular dynamic monitoring by specialized professionals, the compliance of the patients and the parents are the factors of an efficient control that significantly improve the prognosis in this group of patients.

**Patient Consent**

Yes, I received consent

**Disclosure of Interest**

None Declared

### JIA (oligo, poly, psoriatic)

#### P213 Outcome of oligoarticular patients in the German pro-kind cohort

##### Klaus Tenbrock^1, 2^, Gerd Horneff^3^, Dirk Föll^4^, Kristina Vollbach^5^, Ralf Trauzeddel^6^, Hermann Girschick^7^, Frank Dressler^8^, Prasad Oommen^9^, Daniel Windschall^10^, Fabian Speth^11^, Julia Pagel^11^, Ivan Foeldvari^12^, Tilman Kallinich^13^, Markus Hufnagel^14^, Kirsten Mönkemöller^15^, Johannes P. Haas^16^, Rainer Berendes^17^, Ariane Klein^3^, Catharina Schütz^18^, Normi Brück^18^, Jasmin Kümmerle-Deschner^19^, Tatjana Welzel^19^, Anton Hospach^20^, Sonja Mrusek^21^, Frank Weller-Heinemann^22^, Jürgen Brunner^23^, Henner Morbach^24^, Annette Holl-Wieden^24^, Michael Rühlmann^25^, Jens Klotsche^26^, Martina Niewerth^26^, Kirsten Minden^26^ and ProKind Rheuma Germany Studygroup

###### ^1^Translational Pediatric Rheumatology, RWTH Aachen, Aachen, Germany; ^2^University of Bern, Bern, Switzerland; ^3^Asklepios Kliniken, Sankt Augustin; ^4^University of Münster, Münster; ^5^RWTH Aachen, Aachen; ^6^Helios Kliniken Buch; ^7^Vivantes Klinikum Friedrichshain, Berlin; ^8^Kinderklinik der Medizinischen Hochschule Hannover, Hannover; ^9^Universität Düsseldorf, Düsseldorf; ^10^St. Josef-Stift Sendenhorst, Sendenhorst; ^11^UKE; ^12^Hamburger Zentrum für Kinder- und Jugendrheumatologie am Klinikum Eilbek, Hamburg; ^13^Charite, Berlin; ^14^Universität Freiburg, Freiburg; ^15^Kliniken der Stadt Köln gGmbH, Köln; ^16^Deutsches Zentrum für Kinder- und Jugendrheumatologie, Garmisch-Partenkirchen; ^17^Kinderkrankenhaus St. Marien gGmbH, Landshut, ^18^Universität Dresden, Dresden; ^19^Universität Tübingen, Tübingen; ^20^Klinikum Stuttgart, Stuttgart; ^21^Kinderarztpraxis Baden-Baden, Badeb-Badeb; ^22^Klinikum Bremen-Mitte, Bremen, Germany; ^23^Universität Innsbruck, Innsbruck, Austria; ^24^Universität Würzburg, Würzburg; ^25^Kinderarztpraxis Göttingen, Göttingen; ^26^DRFZ, Berlin, Germany

####### **Correspondence:** Klaus Tenbrock

*Pediatric Rheumatology 2024*, **22(2)**: PReS24-ABS-1248

**Introduction:** Improvement of outcome and prognosis in childhood arthritis are the aim of the PROKIND study. It aims to achive this goal by harmonisation of diagnosis, monitoring and treatment decision defining PROKIND protocols, which are based on the treat to target concept.

**Objectives:** To determine the outcomes of patients with newly diagnosed oligoarticular JIA during the first year of treatment.

**Methods:** Outcomes from a prospective observational study of patients with newly diagnosed oligoarticular JIA during the first year of treatment were analysed. Disease activity was assessed with the cJADAS-10. Treat to target was defined as DMARD escalation or change if remission after intraarticular injection or initial DMARD treatment was not achieved

**Results:** 272 oJIA patients from 23 paediatric rheumatology institutions in Germany and Austria were recruited, of these data from 110 patients were available at 6 and 12 months. Most patients received intraarticular corticosteroid therapy as initial therapy, and 49% were in remission at 6 months, while 51% were not. 13% received DMARD escalation / change according to T2T approach, while 37% did not. At 12 months 52% with T2T approach had inactive disease while only 32% of the group that did not follow the T2T approach (p=0,032). Compared to data from the German Kinder Kerndokumentation, DMARDs were used more often in the Pro Kind cohort in persistent oligoarthritis (56% vs 36%).

**Conclusion:** A treat-to-target approach achieves improvement in disease activity of oligoarticular juvenile idiopathic arthritis patients. However, after 12 months, only 50% are in complete remission, which suggest that better treatment approaches are necessary.

**Patient Consent**

Not applicable (there are no patient data)

**Disclosure of Interest**

None Declared

### JIA (oligo, poly, psoriatic)

#### Withdrawn from programme Age at onset of diagnosis as a prognostic factor in Juvenile idiopathic arthritis

##### Anna-Kaisa Tuomi^1^, Katariina Rebane^1^, Hannu Kautiainen^2^, Mia Glerup^3^, Kristiina Aalto^1^ and Nordic Study Group of Pediatric Rheumatology (NoSPeR)

###### ^1^New Children's Hospital, University of Helsinki and Pediatric Research Center; ^2^Folkhälsan Research Centre, Helsinki, Finland; ^3^Aarhus University Hospital, Department of Pediatrics, Aarhus, Denmark

####### **Correspondence:** Anna-Kaisa Tuomi

*Pediatric Rheumatology 2024*, **22(2)**: PReS24-ABS-1285

**Introduction:** Age at onset of a chronic disease may have a negative impact on wellbeing. There are only few studies concerning age at onset as a prognostic factor for juvenile idiopathic arthritis (JIA).

**Objectives:** The aim of this study was to clarify how age at onset of JIA affects the outcome of the disease. We focused on the primary outcome parameters such as remission and Health-Related Quality of Life (HRQoL). In addition, functional ability after 18 years of the diagnosis was evaluated.

**Methods:** This study is part of the population-based Nordic JIA cohort study (1). Newly diagnosed patients with JIA were recruited consecutively between 1997-2000 in specific regions in Finland, Sweden, Norway, and Denmark. Initially, 510 patients were recruited and 358 (70%) of them attended this study. Patients were divided into three age groups: <3 years, 3-5 years and ≥6 years. They were also categorized into three groups according to their International League of Associations for Rheumatology (ILAR) JIA subtypes (2) at six months after the onset of the disease: oligoarthritis, seronegative polyarthritis and others (enthesitis-related arthritis, psoriatic arthritis, and undifferentiated arthritis). Few seropositive and systemic onset patients were excluded. Clinical data were collected six months and 18 years after the disease onset.

**Results:** Mean age was 5.8 years (N=201, 72% female), 5.9 years (N=85, 74% female) and 8.7 years (N=72, 51% female) for oligoarthritis, seronegative polyarthritis and in the group of others, respectively. Remission rates off medication were 62% (95% CI: 54-68), 49% (95% CI: 38-60) and 51% (95% CI: 39-66), respectively, p=0.012.

Related factors predicting remission at the time point of 18 years in the three JIA subgroups are shown in the Table 1. Related factors predicting remission in the oligoarthritis group included age of onset, male gender, JADAS71 score, and uveitis. In the seronegative polyarthritis group, onset age and JADAS71 score predicted remission.

By using logistic regression models, estimated probability of achieving remission 18 years after the diagnosis shows that both genders achieve remission earlier when diagnosed at an earlier age in the oligoarthritis group. In seronegative polyarthritis group, achieving remission is the opposite compared to oligoarthritis group in females. In the group of others, patients in remission were similar (U-shaped) in both genders. Onset age had no significant effect on HRQoL and functional ability. ROC-analyses shows optimal different age cut-off points between JIA subgroups and gender.

**Conclusion:** The current study shows a relationship between continuous age at the diagnosis of the oligoarthritis and seronegative polyarthritis and remission outcome.

**Patient Consent**

Yes, I received consent

**Disclosure of Interest**

None Declared

**References**

1. Glerup M et al. Long-Term Outcomes in Juvenile Idiopathic Arthritis: Eighteen Years of Follow-Up in the Population-Based Nordic Juvenile Idiopathic Arthritis Cohort. Arthritis Care Res (Hoboken). 2020;72(4).
2. Petty RE et al. International League of Associations for Rheumatology. International League of Associations for Rheumatology classification of juvenile idiopathic arthritis: second revision, Edmonton, 2001. J Rheumatol. 2004; 31:390–2.

### JIA (oligo, poly, psoriatic)

#### P215 Methotrexate -induced vomiting syndrome in children with Juvenile idiopathic arthritis

##### Natalia Shevchenko, Olga Pavlova

###### Pediatrics, V. N. Karazin Kharkiv National University, Kharkiv, Ukraine

####### **Correspondence:** Olga Pavlova

*Pediatric Rheumatology 2024*, **22(2)**: PReS24-ABS-1332

**Introduction:** Monitoring of adverse effects of long-term methotrexate (MTX) treatment in children with juvenile idiopathic arthritis (JIA) is ongoing and continues to this day. Controversial results are still available on the liver disorders in patients with JIA.

**Objectives:** To study MTX-induced vomiting syndrome in children with JIA on the background of taking MTX.

**Methods:** 104 patients with polyarthritis (50,96%) and oligoarthritis (40,38%) variants JIA (mean age 13.3 yrs, 59,62 % female, mean age of JIA onset 7.2 yrs, mean disease duration 5.06 yrs) were included in this 4-years prospective study. In 104 children with JIA were treated with MTX 75.96 % (vs. 24.04 % not treated with MTX, 16,35 % were prescribed MTX, but they didn’t receive any dose yet on investigation day). Among patients treated with MTX 4.81 % had dose less than 10 mg/m^2^/week, 32.69 % 10-12.5 mg/m^2^/week, 31.73 % 12.6 - 15 mg/m^2^/week, 6.73 % over 15 mg/m^2^/week. In this study we compared patients who had vomiting and didn`t have it. Also, were analyzed depending on patients’ gender, age, and age of JIA onset, its variant, duration, activity, and presence of MTX in treatment and its dose. Levels of bilirubin, AST, ALT, GGT, LDG, erythrocyte sedimentation rate (ESR), C-reactive protein (C-RP), circulating immune complex (CIC) and antistreptolysin-O (ASL-O) were analyzed in this study

**Results:** In children who received MTX: the average level of age of JIA onset (p = 0.005) significantly increased in patients without vomiting. There were more patients with oligoarthritis without vomiting (p = 0.005). It was also found higher disease duration in patients with vomiting (p = 0.015). Also, patients with vomiting had significantly lower levels of general bilirubin (p = 0.017), direct bilirubin (p = 0.007), undirect bilirubin (p = 0.025) and GGT (p = 0.017). AST level was higher in patients with vomiting (p = 0.018).

In children who had no MTX: the average level of age of JIA onset (p = 0.010) significantly increased in patients with vomiting. There was higher disease duration in patients without vomiting (p = 0.010). Also, patients with vomiting had significantly higher levels of GGT (p = 0.041). ESR level was higher in patients with vomiting (p = 0.0001).

**Conclusion:** Children with JIA had a lower bilirubin and GGT level in patients with vomiting receiving MTX treatment. Patients with vomiting and no MTX treatment were corresponded with high GGT and ESR levels. Presence of lower bilirubin levels may have prognose impact on vomiting syndrome formation in children with JIA.

**Date of birth::** février 18

**Patient Consent**

Yes, I received consent

**Disclosure of Interest**

None Declared

### JIA (oligo, poly, psoriatic)

#### P216 Diagnostic role of synovial calprotectin analysis in juvenile idiopathic arthritis and pigmented villonodular synovitis. pre results of monocentric study

##### Aleksei Kozhevnikov^1, 2^, Elena Derkach^1^, Sergei Vissarionov^1^

###### ^1^H. Turner National Medical Research Center for Children’s Orthopedics and Trauma Surgery, ^2^St. Petersburg State Pediatric Medical University, Saint-Petersburg, Russian Federation

####### **Correspondence:** Aleksei Kozhevnikov

*Pediatric Rheumatology 2024*, **22(2)**: PReS24-ABS-1347

**Introduction:** Juvenile idiopathic arthritis (JIA) is one of the most severe inflammatory joint diseases. Oligoarticular subtype (oligo-JA) most common form of JIA. Pigmented villonodular synovitis (PVNS) is a locally aggressive neoplastic synovial disease characterized by joint effusions, expansion of the synovium and bony erosions which mimic oligo-JA. Intraarticular steroids used in JIA may negatively affect on course of PVNS. Therefore exclusion of neoplastic disease is an important aspect of differential diagnostic of arthritis. Arthroscopy and synovial biopsy used to exclude PVNS often onerous for children with JIA. Majority of laboratory inflammatory tests neither rule in nor rule out JIA. Serum calprotectin in several studies has been demonstrated as a rheumatic disease-specific biomarker.

**Objectives:** The purpose of this study was examine of diagnostic role of synovial calprotectin in JIA and PVNS.

**Methods:** A total of 42 children (all girls; aged median (IQR) 4,2 ± 2,6 years) with oligoarticular onset JIA and 12 with PVNS (66,7% girls; aged median (IQR) 14,2 ± 2,6 years) were enrolled. PVNS – all diffuse form of knee which diagnosed after histological futures. Oligo-JA – at onset all children with ANA-positive active knee arthritis. Synovial fluid was obtained by arthrocentesis. The synovial fluid calprotectin (synCal), level of IL6 and TNF-alpha was tested by an enzyme-linked immunosorbent assay (ELISA). Synovial levels were presented as a median [5th; 95th percentile]. Serum calprotectin (serCal) was also analyzed too.

**Results:** The median of synCal level was 108 μg/ml [28,2; 237] in the oligo-JA group and 1,53 μg/ml [1,26; 1,69] in children with PVNS (p < 0.01). The increase of synCal in children with JIA was more than 20 times. The median level synovial IL6 was 4200 pg/ml [330; 14400] in the JIA group and 4300 pg/ml [1612; 6580] in children with PVNS (p > 0.05). Synovial level of TNF-alpha were not correlated with active oligo-JA and PVNS (2,14 [0,68; 4,22] vs 1,96 [0,34; 3,18] pg/ml, p>0,05). Level of serCal in active oligo-JA were 2,61 μg/ml [1,25; 3,95], PVNS - 1,258 μg/ml [0,78; 2,35].

**Conclusion:** Despite the criteria for JIA diagnostic often associated with some difficulties. Elevated synovial levels of interleukin-6 cannot serve as criteria for identifying JIA. Determine of calprotectin in synovial fluid may be the biomarker which will be helpful for differentiate JIA and PVNS.

**Patient Consent**

Not applicable (there are no patient data)

**Disclosure of Interest**

None Declared

**References**

1. Romano M, Gerloni V, De Lucia O, Piskin D, Giani T, Gattinara M, Borghi MO, Bodio C, Mahler M, Meroni PL, Cimaz R. Serum calprotectin (S100A8/9), clinical and ultrasound assessment in patients with juvenile idiopathic arthritis. Clin Exp Rheumatol. 2021 Sep-Oct;39(5):1132-1140.
2. Marushko T, Holubovska Y, Kulchytska YE. Evaluation of serum calprotectin (MRP-8/MRP-14) levels in patients with juvenile idiopathic arthritis. Journal of Pediatric and Neonatal Individualized Medicine. Journal of Pediatric and Neonatal Individualized Medicine 2021;10(1):e100140
3. Altobelli E, Angeletti PM, Petrocelli R, Lapergola G, Farello G, Cannataro G, Breda L. Serum Calprotectin a Potential Biomarker in Juvenile Idiopathic Arthritis: A Meta-Analysis. J Clin Med. 2021 Oct 22;10(21):4861.

### JIA (oligo, poly, psoriatic)

#### P217 Kidney lesions in children with Juvenile idiopathic arthritis

##### Natalia S. Shevchenko^1, 2^, LIudmyla F. Bogmat^2^, Tetiana O. Holovko^1, 2^

###### ^1^Department of Pediatrics, V.N.Karazin Kharkiv National University; ^2^Department of Rheumatology and comorbidities, SI "Institute for Children and Adolescents Health Care of the NAMS of Ukraine", Kharkiv, Ukraine

####### **Correspondence:** Natalia S. Shevchenko

*Pediatric Rheumatology 2024*, **22(2)**: PReS24-ABS-1348

**Introduction:** Kidney lesions in adult patients with rheumatoid arthritis are found with a very high frequency (from 36 to 73%), the prevalence of chronic kidney disease is much higher than in the general population [1, 2]. Nephropathy can lead to nephrosclerosis development, a decrease in the number of functioning nephrons with chronic renal failure formation [3, 4]. Early markers of kidney failure are a decrease in urine specific gravity, glomerular filtration rate (GFR), and increased urinary protein excretion

**Objectives:** To determine kidneys’ functional state in children with juvenile idiopathic arthritis (JIA) considering due to disease activity.

**Methods:** 152 children aged 10-18 (13.42 ± 0.22) with JIA were examined, 68 boys and 80 girls. Control group consisted of 38 healthy children (9 boys, 29 girls), their peers (14.72 ± 0.28 years old). JADAS 27 disease activity was assessed. All patients received methotrexate treatment for at least 12 months.

Analysis of clinical indicators of urine, creatinine and urea concentration, glomerular filtration rate (GFR), albuminuria per day was carried out.

**Results:** Among patients with JIA, polyarticular variant was in 105 patients, oligoarticular variant in 47 children. Disease duration was 72.14 ± 3.69 months (polyarticular - 72.44 ± 4.63 months, oligoarticular - 71.48 ± 6.06 months); JADAS27 activity in children with oligoarticular variant was 8.15 ± 0.93 units, with polyarticular - 12.83 ± 0.63 units. High activity (III stage) was observed in 83 (54.6%) children, moderate (II stage) - in 38 (25.0%) and low (0 - I stage) - in 31 (20.4%)).

In children with JIA, the level of albumin in urine was higher in children of the main group (37.47 mg/day vs. 13.30 mg/day, p < 0.01) and increased according to the degree of JIA activity (14.36 mg/day; 20.52 mg/day; 47.47 mg/day, respectively). Also, patients with high activity had a lower urine density, both the minimum and maximum values during the day (p < 0.05).

**Conclusion:** In children with JIA, when the disease lasts more than one year, markers of kidney damage are determined, namely: a decrease in the specific gravity of urine and an increase in the excretion of protein in the urine, the intensity of which depends on the activity of the disease.

**Patient Consent**

Yes, I received consent

**Disclosure of Interest**

None Declared

**References**

1. Hironari Hanaoka, et al. *Clinical Kidney Journal*. 15(7), 2022. P. 1373–1378, 10.1093/ckj/sfac036
2. Ashraf O. Oweis et al. Renal dysfunction among rheumatoid arthritis patients: A retrospective cohort study. *Annals of Medicine and Surgery*. 2020. 60. P. 280-284. 10.1016/j.amsu.2020.11.011
3. Karie, et al. Kidney disease in RA patients: prevalence and implication on RA-related drugs management: the MATRIX study, *Rheumatology*. 47 (3), 2008, P. 350–354.10.1093/rheumatology/kem370
4. Mori, S*et al.* Prevalence of and factors associated with renal dysfunction in rheumatoid arthritis patients: a cross-sectional study in community hospitals. *Clin Rheumatol.* 36, 2673–2682 (2017). 10.1007/s10067-017-3804-5.

### JIA (oligo, poly, psoriatic)

#### P218 Correlation of platelet indices with disease activity in Indian children with Juvenile idiopathic arthritis: a cross sectional study

##### Akash Singhal^1^, Aarushi Singla^1^, Saloni Goyal^1^, Anu Maheshwari^1^, Sangeeta P. Sindhwani^2^, Deonath Mahto^1^

###### ^1^Paediatrics; ^2^Pathology , Lady Hardinge Medical College and associated Kalawati Saran Children's Hospital, New delhi, India

####### **Correspondence:** Saloni Goyal

*Pediatric Rheumatology 2024*, **22(2)**: PReS24-ABS-1459

**Introduction:** Inflammation in Juvenile Idiopathic Arthritis (JIA) can alter the platelet indices. With the widespread and easy availability of five-part and seven-part coulter machines, platelet indices are available at point of care. Inflammatory mediators may influence platelet indices; therefore, platelet indices can be used as a marker for disease activity.

**Objectives:** To study the correlation of platelet indices with disease activity score in children with JIA.

**Methods:** A cross-sectional study conducted at Kalawati Saran Children's Hospital in New Delhi between November 2018 and March 2020, which included 86 children, aged 1 to 18 years, diagnosed with JIA according to ILAR criteria. Exclusions comprised cases of inherited platelet disorder, chronic ITP, aplastic anemia, hemoglobinopathies, hypothyroidism, chronic liver disease, and chronic kidney disease. Disease activity was assessed using juvenile arthritis disease activity score (JADAS–27)^1^, and platelet indices^2^ (platelet count, Mean Platelet Volume (MPV), Plateletcrit (PCT), Platelet distribution width (PDW) and Platelet-large cell ratio (PLCR) were measured with a SYSMEX XN-1000 automated blood cell counter. Quantitative data were expressed as mean with standard deviation or median with interquartile range, with differences between groups analyzed using appropriate statistical tests (t-test or Mann Whitney ‘U’ test for two groups, ANOVA or Kruskal Wallis H test for more than two groups, followed by post-hoc tests). Qualitative data were expressed as percentages. Statistical differences were assessed using chi-square test or Fisher’s exact test. Correlation between quantitative variables was determined using Spearman correlation coefficient (r<0.4 weak correlation; r = 0.40-0.59 moderate correlation; r ≥ 0.60 strong correlation).

**Results:** Amongst 86 patients with JIA, 50 were males and the median age of presentation was 9.5 years (IQR 5.75-13). Mean age of onset of symptoms was 7.75 years (4.39-11). Median JADAS-27 score was 13.05 (IQR 4-21.5). Among JIA subtypes, Rheumatoid factor (RF) negative polyarticular JIA was the most common (45.3%), followed by oligoarticular JIA (24.4%), Enthesitis Related Arthritis (ERA) was 17.4%, systemic onset JIA (SOJIA) was 10.5% and rheumatoid factor positive polyarticular JIA was 2.3%, respectively. Platelet count (x 10^9/L) exhibited a positive correlation with JADAS- 27 score (r=0.37, p<0.001) with a mean value of 319 ± 103 (range: 113 - 658), while plateletcrit (%) also demonstrated a positive correlation with JADAS-27 score (r=0.38, p<0.001) with a mean value of 0.37 ± 0.10 (range: 0.16 - 0.70). However, mean platelet volume (MPV, fL), platelet distribution width (PDW, fL), and platelet large cell ratio (PLCR, %) did not show significant correlations with JADAS-27 score in children with JIA.

**Conclusion:** Platelet count and plateletcrit showed positive correlation with disease activity. As these indices are cost effective and easily available, they can be used as a point of care biomarkers to monitor disease activity in patients with JIA and can be used to tailor treatment. Further studies are needed to study the correlation between the two.

**Date of birth::** septembre

**Patient Consent**

Yes, I received consent

**Disclosure of Interest**

None Declared

**References**

1. Consolaro A, Giancane G, Schiappapietra B, Davì S, Calandra S, Lanni S, *et al.* Clinical outcome measures in juvenile idiopathic arthritis. Pediatr Rheumatol. 2016 Dec;14(1):1–8.
2. Gawlita M, Wasilewski J, Osadnik T, Reguła R, Bujak K, Gonera M. Mean platelet volume and platelet-large cell ratio as prognostic factors for coronary artery disease and myocardial infarction. Folia Cardiol. 2015;10(6):418–22

### JIA (oligo, poly, psoriatic)

#### P219 Overweight and obesity are not associated to a higher disease activity in an Italian cohort of patients with Juvenile idiopathic arthritis

##### Simona Di Gennaro, Maria Sacco, Gianmarco Fiorenza, Caterina De Simone, Roberta Naddei, Maria Alessio

###### Department of Translational Medical Sciences, Section of Pediatrics, University Federico II, Naples, Italy

####### **Correspondence:** Simona Di Gennaro

*Pediatric Rheumatology 2024*, **22(2)**: PReS24-ABS-1471

**Introduction:** Contrasting data are available on the impact of obesity on the clinical course of juvenile idiopathic arthritis (JIA) (1-2).

**Objectives:** To assess the prevalence of overweight and obesity in our JIA cohort and to evaluate their impact on the disease activity.

**Methods:** A single-center retrospective study was conducted by reviewing the medical records of patients with JIA consecutively seen at our Unit from January to June 2020. Demographic and clinical data, including treatment regimens, were collected and compared across 5 groups of patients distinguished by body mass index (BMI) z-scores (3).

**Results:** Our JIA cohort was constituted mainly by female (78%) and the Oligoarticular subtype was the most common (73%). Median age at onset was 4.08 years (IQR 2.16-7.04) while the median disease duration was 5.16 years (IQR 2.60-8.66). Among our 5 groups of patients, 5.6% have been classified as underweight, 54.8% as normal weight, 25.1% as overweight, 11.7% as obese, and 2.8% as severely obese. Obese patients were more frequently males (p=0.027). There was no association between overweight or obesity and JIA subtypes. The swollen, tender, limited and active joint resulted similar in all 5 groups, as well as the physician and the parent global assessments. The ESR was higher in the severe obesity group, even though not significantly (p=0.095). No significant differences in the levels of cJADAS10 and JADAS10 among the 5 categories of subjects. The prevalence of active disease according to the JADAS10 in the 5 groups ranged from 33.3% to 46.7% with no significant differences. The time spent on biological medications resulted similar among the 5 categories of patients, as well as the age of starting those drugs.

**Conclusion:** Our cohort was one of the largest cohorts aimed to investigate the prevalence and the impact of obesity in JIA. Obesity prevalence resulted comparable to previous reports in some European JIA cohorts and resulted higher in males. Our findings suggest that obesity and overweight are not associated to higher disease activity in JIA. However, obesity should still be viewed as a risk factor for children's well-being and as an additional comorbidity.

**Patient Consent**

Not applicable (there are no patient data)

**Disclosure of Interest**

None Declared

**References**

1. Giani T et al.The Influence of Overweight and Obesity on Treatment Response in Juvenile Idiopathic Arthritis. Front Pharmacol. 2019 Jun 12; 10:637.
2. Pelajo CF et al. Obesity and disease activity in juvenile idiopathic arthritis. Pediatr Rheumatol Online J. 2012 Jan 12;10(1):3.
3. Miranda-Alatriste et al. Association between BMI z-score and body composition indexes with blood pressure and grip strength in school-age children: a cross-sectional study. Scientific Reports 14.1. 2024: 5477.

### JIA (oligo, poly, psoriatic)

#### P220 Epidemiology of juvenile idiopathic arthritis in Mexico

##### Fernando García-Rodríguez, Jimena García-Silva, Alondra Correa-Galván, Raul O. Guerra-Espiricueta, Humberto Ochoa-Alderete, Ana K. Leos-Leija, Ana V. Villarreal-Treviño, Nadina Rubio-Pérez and Colaborativa de Investigación en Beneficio de la Reumatología Infantil (COLIBRI)

###### Pediatrics, Universidad Autónoma de Nuevo León, Monterrey, Mexico

####### **Correspondence:** Fernando García-Rodríguez

*Pediatric Rheumatology 2024*, **22(2)**: PReS24-ABS-1559

**Introduction:** Epidemiology of Juvenile Idiopathic Arthritis (JIA) differs among regions; characteristics of this disease have been well described in Europe and the USA, but there is limited information about the incidence, prevalence, and clinical features of these patients in Latin America.

**Objectives:** To describe demographics, clinical features, treatment, and long-term outcomes of patients with JIA from a single center in Mexico.

**Methods:** Observational study of patients diagnosed with JIA after 2000 in a referral center for pediatric rheumatology in Northeast Mexico. Descriptive statistics of demographic and clinical data were carried out using SPSS v.27.

**Results:** A total of 130 patients with a diagnosis of JIA were included, 91 girls (70%), with an age at diagnosis of 118.5 (IQR 80.7-143) months. Seventy-two patients presented with polyarthritis (55.4%), 46 of them had positive rheumatoid factor (35.4%). Enthesitis-related (ERA), systemic, and oligoarticular JIA presented in similar frequencies (19 [14.6%], 18 [13.8%], and 15 [11.5%], respectively). The median time between the onset of symptoms and diagnosis was 143.5 (IQR 66-291) days and the time to diagnosis after the patient's first visit to our center was 0 (IQR 0-15.5) days. The longest times to diagnosis were observed in ERA (164 [IQR 63-418] months) and the shortest in oligoarticular (92 [IQR 59-336]).

At diagnosis 43 presented with positive ESR (18, IQR 9-33mm/h), 29 with positive CRP, and 1 with uveitis. During the first visit, the median number of active joints was 5 (IQR 2-13), the global medical VAS 4 (IQR 3-6), the patient VAS 5 (IQR 3-7), and the JADAS 10 score was 17.1 (IQR 9.1-22.9). The knees were the most frequently active joints at diagnosis (63.7%) followed by the ankles (38.2%) and hips (19.6%). The highest JADAS 10 scores were observed in systemic (21 [IQR 16.5-27.8]) and the lowest in oligoarticular (6.6 [IQR 5.5.-10.2]).

After their first visit, 65 patients (49.6%) received methotrexate as treatment. Seventy-one adverse events were identified during the follow-up, 17 (24%) were classified as serious, and 14 (20%) as related to the treatment, based on the MedDRA terminology.

The median time of follow-up was 39 (IQR 14.7-72) months, longer for oligoarticular JIA patients (56 [21-128] months). Based on the Wallace criteria, 60 patients (46.2%) achieved remission on their last visit.

**Conclusion:** This study highlights the epidemiology of JIA found in our population. Compared with other reports, the polyarticular course of JIA was the most frequent subtype in our population. Further studies, in bigger cohorts, are needed to continue to define the characteristics of JIA patients in the Mexican population, and how could these factors affect the JIA phenotype expression and treatment response.

**Date of birth::** mars 27, Y

**Patient Consent**

Not applicable (there are no patient data)

**Disclosure of Interest**

None Declared

### JIA (oligo, poly, psoriatic)

#### P221 Juvenile idiopathic arthritis in adulthood: long-term follow up and outcome of the disease

##### Ioanna Saougkou, Theodora E. Markatseli, Alexandros A. Drosos, Paraskevi V. Voulgari

###### Department of Internal Medicine, Rheumatology Clinic, University Hospital , Ioannina, Greece

####### **Correspondence:** Ioanna Saougkou

*Pediatric Rheumatology 2024*, **22(2)**: PReS24-ABS-1635

**Introduction:** Many patients with JIA are followed into adulthood, experiencing detrimental effects due to prolonged synovitis and progressive joint damage. The course of the disease is unpredictable and active arthritis creates through the years cumulative negative effect on functional status and social outcomes.

**Objectives:** The aim of this study was to explore and determine the long term burden of the disease in adult patients with JIA, evaluate their disease activity, functional and social outcomes.

**Methods:** Patients with JIA referred to a tertiary university hospital in Greece were identified. Patients older than 18 years old, with more than 5 years of disease duration were enrolled. Data regarding ILAR category at onset, age at disease onset, disease duration (years), presence of rheumatoid factor (RF), anticitrullinated protein antibodies (ACPA), antinuclear antibodies (ANAs considered positive if titres ≥160), acute phase reactant levels, pain levels, sociodemographic features, Health Assessment Questionnaire (HAQ), 10-joint Juvenile Arthritis Disease Activity Score (JADAS-10), comorbidities and participation in society (educational and vocational status) were analysed.

**Results:** Complete data were available for 56 out of 78 patients identified. Not all JIA subtypes were represented (there were no cases of psoriatic arthritis documented). The highest mean disease duration was observed in systemic arthritis (sJIA) subtype (25.00±12.00) while the lowest in enthesitis related arthritis (ERA) subtype (7.2±5.07) years. 62.5% of patients had active disease based on clinical examination. In RF+ Poly and sJIA subtypes we documented the worst mean HAQ and JADAS-10 score, demonstrating the joint damage in these subtypes. The most common complication of the disease in sJIA subtype was cushing disease and opthalmological complications mainly cataract; both of them developed due to the frequent and intense use of corticosteroids in the era prior to biological treatments. Uveitis was observed in 11 out of 56 patients overall (19.6%) predominantly in the oligoarthritis group and 7 of the 56 patients had at some point in the disease course joint replacement surgery. 21 out of 56 patients managed to obtain university degree; nevertheless 10 of 56 patients were unemployed reflecting the social consequences of JIA.

**Conclusion:** The study illustrates clinical and functional information on adult patients with longstanding JIA. Although severe disease outcomes in JIA are more rare than they were decades ago, we demonstrated in this analysis that morbidity is still evident in adults with JIA requiring continuous rheumatology care.

**Patient Consent**

Not applicable (there are no patient data)

**Disclosure of Interest**

None Declared

### JIA (oligo, poly, psoriatic)

#### P222 Analysis of immunoglobulin repertoire reveals altered peripheral b cell tolerance checkpoints in patients with Juvenile idiopathic arthritis

##### Emiliano Marasco, Angela Aquilani, Rebecca Nicolai, Guisyda Tarantino, Silvia Magni Manzoni, Fabrizio De Benedetti

###### Rheumatology, Bambino Gesù Children Hospital, Rome, Italy

####### **Correspondence:** Emiliano Marasco

*Pediatric Rheumatology 2024*, **22(2)**: PReS24-ABS-1710

**Introduction:** Juvenile Idiopathic Arthritis (JIA) is the most common form of chronic arthritis in childhood. The adaptive branch of the immune system plays a crucial role in the development of JIA. We have showed that switched memory B cells are expanded in patients with oligoarticular- and polyarticular-JIA.

**Objectives:** To characterize the antigen-experienced immunoglobulin repertoire, we performed deep sequencing of the switched repertoire in peripheral blood (PB) of oligo-/ploy-JIA patients(n=14) and age matched controls(n=4), and in matched synovial fluid (SF) of oligo-/ploy-JIA patients(n=7).

**Methods:** We employed a cDNA-based-5′RACE approach with unique molecular identifiers. De-multiplexing, UMI extraction, and UMI-based consensus assembling were performed using the MIGEC software. Clonotype assembly were performed using MiXCR. SHM analysis was performed with SHazaM. B cell tolerance checkpoints were analyzed with a validated flow cytometry-based system (Malkiel S, Arthritis Rheumatol. 2016).

**Results:** Immunoglobulin heavy chain variable gene segment usage frequency was similar between patients and controls, and PB and SF. Thus, we did not observe clustering of patients from controls. Analysis of averaged complementarity-determining region 3 (CDR3) repertoire characteristics revealed that CDR3 length was similar among the three groups, whereas physico-chemical characteristics of amino acid residues were significantly different in the IgG repertoire, but not in the IgA repertoire. IgG from SF showed higher charge and polarity and an increased hydrophobicity index compared to PB of controls and patients. We then analyzed the frequency of somatic hypermutation (SHM) and the selection pressure with SHazaM. We found a significant reduction in the frequency of SHM and a higher frequency of sequences with less than 10 mutations per Ig molecule in patients (in both PB and SF) than controls. The IgG repertoire in SF exhibited decreased positive selection in CDRs. To further characterize the immunoglobulin repertoire, we performed a flow cytometry-based staining to assess the frequency of autoreactive B cells in the transitional, naïve and memory B cell subsets in PB and also of memory B cells in SF. We found that central tolerance checkpoints are effective in both JIA patients and controls: the frequency of autoreactive B cells decreased from transitional, to naïve B cells in both JIA patients and controls. However, the frequency of autoreactive B cells was higher in memory B cells of JIA patients compared to controls, with the highest frequency observed in memory B cells of SF.

**Conclusion:** Altogether, the observed changes in the IgG repertoire in PB and SF establish an altered peripheral selection process of IgG+ B cells in oligo-/ploy-JIA with a decreased load of SHM. Our data show for the first time that an altered GC response, leading to the accumulation of autoreactive B cells in the memory compartment, is a feature of oligo-/ploy-JIA.

**Patient Consent**

Yes, I received consent

**Disclosure of Interest**

None Declared

### JIA (oligo, poly, psoriatic)

#### P223 Exploring the outcomes of patients with JIA during the transition period

##### Kübra Uçak, Nihal Şahin, Hafize E. Sönmez

###### Department of Pediatric Rheumatology, Kocaeli University, Kocaeli, Türkiye

####### **Correspondence:** Kübra Uçak

*Pediatric Rheumatology 2024*, **22(2)**: PReS24-ABS-1795

**Introduction:** Juvenile idiopathic arthritis (JIA) stands as the most prevalent form of chronic arthritis during childhood, boasting an incidence rate of 1:10,000. Thanks to advancements in treating pediatric rheumatic diseases, a growing cohort of children now advances into adulthood, necessitating their transition from pediatric to adult healthcare systems. There is limited data on the outcomes of patients with JIA during the transition period.

**Objectives:** The study aims to evaluate the outcomes and presence of damage in patients with JIA during the transition period.

**Methods:** This is a retrospective cohort study evaluating children aged between 16 and 18 years who were diagnosed with JIA. Diagnosis of JIA was made according to the International League of Associations for Rheumatology (ILAR) criteria. Patients with concomitant diseases were excluded. Patients were assessed using the Juvenile Arthritis Damage Index for arthritis (JADI-A) and extra-articular involvement (JADI-E).

**Results:** A total of 83 patients were included in the study. Of them, 43 were male (51.8%), 40 were female (48.2%). The male-female ratio was 1.07. The median age at the diagnosis was 14 years. JIA subtypes of patients were ERA in 33, persistent oligo JIA in 27, extended oligo JIA in 8, RF negative poly JIA in 6, psoriatic arthritis in 6, systemic JIA in 6 and RF positive JIA in one. At the last transition visit, 39 patients (47.6%) were in remission with medication, 29 patients (35.4%) had active disease, and 14 patients (17.1%) were in remission without medication. Among patients, 33 (39.3%) were receiving biologics, and 20 (23.8%) were receiving DMARDs. When we evaluated damage, there were sequelae in 6 (7.7%) patients. Of these 6 patients, 2 had articular damage (ankylosis in 2) with a median JADI-A score of 4, and 4 had extra-articular damage (cataract in 2, glaucoma in 1, and avascular necrosis in 1) with a median JADI-E score of 2.

**Conclusion:** Comprehensively assessing the outcomes and presence of patients with JIA during the transition period may facilitate the optimal transition process. Although there have been many advances in diagnosis and treatment of JIA, active disease continues into adulthood in a significant portion of children with JIA. We believe that providing data on the current outcomes of patients before transition will contribute to the development of transition preparation policies at healthcare centers.

**Date of birth::** avril 09,

**Patient Consent**

Yes, I received consent

**Disclosure of Interest**

None Declared

### JIA (oligo, poly, psoriatic)

#### P224 Different profiles of autoantibodies in juvenile idiopathic arthritis (JIA) and Rheumatoid Arthritis (RA)

##### Nadine Fischer^1^, Christian Klemann^2^, Fiona Engelke^3^, Boris Huegle^1^, Torsten Witte^3^, Johannes-Peter Haas^1^

###### ^1^German Centre for pediatric and adolescent rheumatology, Garmisch-Partenkirchen; ^2^Dep. of Rheumatology and Immunology, Childrens´hospital, University of Leipzig, Leipzig; ^3^Dep. for Rheumatology and Immunology, MH-Hannover, Hannover, Germany

####### **Correspondence:** Nadine Fischer

*Pediatric Rheumatology 2024*, **22(2)**: PReS24-ABS-1126

**Introduction:** Autoantibodies (aAB) are a frequent phenomenon in patients with Juvenile Idiopathic Arthritis (JIA) as well as those with Rheumatoid Arthritis (RA). While antinuclear antibodies are present in many JIA patients, rheumatoid factors are predominantly found in patients with RA. We conducted samples from these populations considering both age groups to get a more precise insight into their aAB-profiles.

**Objectives:** Comparing profiles of autoantibodies in different categories of JIA- and RA-patients.

**Methods:** Presence of IgG aAB against 67 antigens were analyzed in patients with JIA (acc. ILAR classification) and RA (acc. ACR-criteria) using Luminex method. The cut off value was determined using the ≥98 quantile of the fluorescence intensity values in healthy controls (n=123).

**Results:** In JIA, 290 patients (78% female) have been classified as: persisting oligoarthritis n=67 (PO-JIA), extended oligoarthritis n=114 (EO-JIA), rheumatoid factor-negative polyarthritis n=66 (SNP-JIA), rheumatoid factor-positive polyarthritis n= 43 (RFP-JIA). As a comparison, sera samples from 50 adult RA patients were tested, including rheumatoid factor-negative RA n=15 (SNP-RA) and rheumatoid factor-positive RA n=35 (RFP-RA) patients.

Any aAB was detected in 65% of the JIA patients independent from gender, presence of ANA or duration of the disease. Most of JIA as well as RA patients were positive for aAB, although the frequency is significantly higher in adults with RFP-RA (see table 1). Some aAB were preferentially found in adults (e.g. CTSL, IL37), while some were exclusively present in JIA (e.g. FAF1). The frequency of anti-Ro aAB (TRIM21 und TROVE2) was significantly higher in RA patients than reported in the literature.

**Conclusion:** Both populations demonstrated complex patterns of aAB with marked differences in the presence and frequencies when comparing certain categories of JIA and RA. Clinical and functional relevance of the autoimmune reactions resulting in these aAB profiles have to be further analyzed.

**Patient Consent**

Not applicable (there are no patient data)

**Disclosure of Interest**

None Declared

**Reference**

1. This project was supported by a non-restricted grant of the „Verein Hilfe für das rheumakranke Kind eV“.

**Table 1 (abstract PReS24-ABS-1126).** Clinical parameters and results from selected aAB in patients with JIA (*n*=290) and RA (*n*=50)

	PO-JIA (67)	EO-JIA (114)	SNP-JIA (66)	RFP-JIA (43)	JIA-total (290)	SNP-RA (15)	RFP-RA (35)	RA total (50)
**Mean number of aABs positive**	1,2	1,2	1,3	1,3	1,3	2,9	**7,0**	5,8
*CTSL (%)*	*1,5*	*2,6*	*3,0*	*4,7*	*2,8*	***13,3***	***48,6***	*38,0*
*FAF1 (%)*	***9,0***	***6,1***	***9,1***	***4,7***	*7,2*	*0*	*0*	*0*
*HNRNPBA1 (%)*	***7,5***	*3,5*	*0*	*0*	*3,1*	*6,7*	***31,4***	*24,0*
*IL37 (%)*	*0*	*0*	*0*	*0*	*0*	***6,7***	***11,0***	*10,0*
*TRIM21 (Ro52) (%)*	*1,5*	*0,9*	*1,5*	*4,7*	*1,7*	***13,3***	***14,3***	*14,0*
*TROVE2 (Ro60) (%)*	*1,5*	*0,9*	*0*	***7,0***	*1,7*	***13,3***	***28,6***	*24,0*
*CDR2 (%)*	*1,5*	*2,6*	***6,1***	*0*	*2,8*	***13,3***	*8,6*	*10,0*
*PPL (%)*	*6,0*	*3,5*	*0*	***11,6***	*4,5*	*0*	***20,0***	*14,0*

### JIA (oligo, poly, psoriatic)

#### P225 Transitional care experience in Florence

##### Laura Gatti^1^, Ilaria Pagnini^1^, Jelena Blagojevic^2^, Ilaria Maccora^1, 3^, Edoardo Marrani^1^, Vincenza Mastrolia^1^, Serena Guiducci^2^, Gabriele Simoninini^1, 3^

###### ^1^Rheumatology Unit, ERN-ReCONNET center, Meyer Children's Hospital IRCCS; ^2^Experimental and Clinical Medicine, Division of Rheumatology, AOUC - University of Florence, ^3^NEUROFARBA, University of Florence, Florence, Italy

####### **Correspondence:** Maria Vincenza Mastrolia

*Pediatric Rheumatology 2024*, **22(2)**: PReS24-ABS-1145

**Introduction:** In 2016, the EULAR/PReS provided the transitional care shared recommendations for young people with rheumatic diseases (1), highlighting the importance of a direct and clear process in order to facilitate the transfer into the adult world.

**Objectives:** We describe our experience in the transitional process of young people affected by Juvenile Idiopatic Arthritis (JIA) followed at Meyer Children’s Hospital IRCSS and Careggi Adult Rheumatology Center in Florence, Italy.

**Methods:** We performed a retrospective observational study including 43 patients with JIA, classified according to ILAR criteria (2), who underwent the transitional process from 2017 up to 2024. We collected data on the JIA sub-type, disease activity (JADAS-10, CHAQ, EQ-5D) (3), treatment during disease course and overall condition at time of transitional care. Our transitional care process foresees at least 2 joined outpatient clinic visits for each patient.

**Results:** We enrolled 43 patients (27 female) affected by JIA: 15 patients had oligo-arthritis, 14 polyarthritis, 9 enthesitis-related arthritis, 4 psoriasic arthritis and 1 systemic JIA. At disease onset, 14 patients had JADAS-10 score ≥ 30, CHAQ score ≥2,3 and EQ-5D score ≤ 20, and were then stratified as severe disease activity; 11 patients had JADAS-10 score ≤15, CHAQ score ≤ 1,3 and EQ-D5 score ≥ 80, and entered as with mild disease activity; 18 patients had intermediate scores (JADAS-10 score 15-30, CHAQ score 1,4-2,2 and EQ-5D score 40-70) and then considered with moderate disease activity. 36 patients started a treatment with Disease Modifying Antirheumatic Drugs (DMARDS), 23 of which also added a biological DMARDS (bDMARD), while 3 patients started directly with bDMARD for a severe disease activity. At transitional care, 28 patients were in complete remission on medications (14 with bDMARDS, 8 with DMARDS and 6 with a combination therapy of bDMARDS plus DMARDS), 12 were in complete remission off-therapy, and 3 had still active disease in treatment with bDMARDS. All the patients, except the 3 young adults with active disease, had a JADAS score and a CHAQ score of 0 and a EQ-5D score of 100, highlighting the overall wellness. In 39 patients, we performed 2 joined sessions with pediatric and adult rheumatologists, while 4 patients required 3 joined sessions. At the successive adult follow-up, 38 patients maintained the same therapy at transition, while 5 patients switched therapy due to flare of disease activity. All the patients regularly continued the follow-up with the adult rheumatologists and no drop-out events have been then entered.

**Conclusion:** This is the first retrospective study to analyze a relevant cohort of patient according to the EULAR/PReS standards for the transitional care. Our transitional care setting succeeded in efficiently passing 43 patients from the pediatric care to the adult care, without any drop-out in the follow-up. More study will be necessary to enhance the transitional care.

**Patient Consent**

Yes, I received consent

**Disclosure of Interest**

None Declared

**References**

1. Foster HE et al. EULAR/PReS standards and recommendations for the transitional care of young people with juvenile-onset rheumatic diseases. Ann Rheum Dis. 2017 Apr;76(4):639-646.
2. Petty RE et al. ILAR classification of juvenile idiopathic arthritis: second revision, Edmonton, 2001. J Rheumatol. 2004 Feb;31(2):390-2.
3. Consolaro A et al. Development and validation of a composite disease activity score for juvenile idiopathic arthritis. Arthritis Rheum. 2009 May 15;61(5):658-66.

### JIA (oligo, poly, psoriatic)

JIA (oligo, poly, psoriatic)

#### P226 Adherence to low-dose methotrexate in children with Juvenile idiopathic arthritis using a sensitive methotrexate assay

##### Julia Möhlmann^1^, Sytze de Roock^2^, Annelies Egas^3^, Evelien ter Weijden^1^, Martijn Doeleman^2^, Alwin Huitema^1, 4, 5^, Matthijs van Luin^1^, Joost Swart^2^

###### ^1^Clinical Pharmacy; ^2^Pediatric Rheumatology and Immunology; ^3^Clinical Diagnostics, University Medical Centre Utrecht, Utrecht University, Utrecht; ^4^Clinical Pharmacy and Pharmacology, Antoni van Leeuwenhoek, Netherlands Cancer Centre, Amsterdam; ^5^Pharmacology, Princess Máxima Centre for Pediatric Oncology, Utrecht, Netherlands

####### **Correspondence:** Julia Möhlmann

*Pediatric Rheumatology 2024*, **22(2)**: PReS24-ABS-1277

**Introduction:** Low-dose weekly methotrexate (MTX) is the mainstay of treatment in juvenile idiopathic arthritis (JIA) [1]. Unfortunately, a substantial part of patients has insufficient efficacy of MTX [2]. A potential cause of this inadequate response is suboptimal drug adherence [3].

**Objectives:** The aim of this study was to assess MTX adherence in JIA patients by quantification of MTX concentrations in plasma at start of the treatment and after one year of MTX use. Secondly, the association between MTX concentrations and either self-reported adherence issues, or concomitant use of biologics was examined.

**Methods:** This was a retrospective, observational study using plasma samples from JIA patients. An ultrasensitive liquid chromatography-tandem mass spectrometry method was developed for quantification of MTX and its metabolite 7-hydroxy-MTX in plasma. The determined MTX plasma concentrations in JIA patients were compared with adherence limits, corresponding with the dose and time after administration, categorising them as either adherent or possibly non-adherent to MTX therapy.

**Results:** Plasma samples of 43 patients with JIA were analysed. The median age was 11 years (1 to 17 years) and 25 (58%) patients were female. At start of MTX treatment, 5 (12%) patients had concomitant treatment with a biological. This increased to 17 (40%) patients after one year. Adherence to MTX in our JIA population was 88% shortly after initiation of MTX therapy and decreased to 77% after one year of treatment. In a sub-analysis performed in the 40% of patients with a known day of administration, MTX adherence percentages were 83% at baseline, decreasing to 59% after one year of use. There were 3 patients with MTX concentrations below the adherence limit at both sampling points. Possibly non-adherent patients had a higher age (p=0.002). Such negative association between age and MTX adherence was observed before [4]. We could not find an association between MTX adherence with either self-reported adherence issues, nor with the use of concomitant biological treatment (p=1.00 and p=0.27, respectively; Fisher’s Exact).

**Conclusion:** Quantification of MTX in plasma is a feasible and objective method to assess adherence in patients using low-dose weekly MTX. In clinical practice, the use of this method could be a helpful tool for physicians to refute or support suspicion of non-adherence to MTX therapy.

**Patient Consent**

Not applicable (there are no patient data)

**Disclosure of Interest**

None Declared

**References**

1. Ferrara G, et al. Methotrexate in juvenile idiopathic arthritis: Advice and recommendations from the MARAJIA expert consensus meeting. Vol. 16, Pediatric Rheumatology. BioMed Central Ltd.; 2018
2. Bulatović M, et al. Prediction of clinical non-response to methotrexate treatment in juvenile idiopathic arthritis. Ann Rheum Dis. 2012 Sep;71(9):1484–9
3. Hope HF, et al. Psychological factors predict adherence to methotrexate in rheumatoid arthritis; findings from a systematic review of rates, predictors and associations with patient-reported and clinical outcomes. RMD Open. 2016;2(1):e000171
4. Pelajo CF, Sgarlat CM, Lopez-Benitez JM, Oliveira SKF, Rodrigues MCF, Sztajnbok FR, et al. Adherence to methotrexate in juvenile idiopathic arthritis. Rheumatol Int. 2012 Feb;32(2):497–500;

### JIA (oligo, poly, psoriatic)

#### P227 Intricate two-way relationship between the heart and kidneys in children with Juvenile idiopathic arthritis

##### LIudmyla F. Bogmat^1^, Natalia S. Shevchenko^1, 2, 2, 3^, Tetiana O. Holovko^1, 2^

###### ^1^Department of Rheumatology and comorbidities, SI "Institute for Children and Adolescents Health Care of the NAMS of Ukraine"; ^2^Department of Pediatrics, V.N.Karazin Kharkiv National University; ^3^Department of Rheumatology and comorbidities, SI "Institute for Children and Adolescents Health Care of the NAMN of Ukraine", Kharkiv, Ukraine

####### **Correspondence:** Natalia S. Shevchenko

*Pediatric Rheumatology 2024*, **22(2)**: PReS24-ABS-1350

**Introduction:** Inflammation and endothelium dysfunction in patients with rheumatic diseases are crucial for pathological changes in many organs and systems as well as secondary cardiorenal syndrome development [1].

**Objectives:** To evaluate two-way relationship between the heart and kidneys in children with juvenile idiopathic arthritis,

**Methods:** 152 children aged 10-18 (13.42 ± 0.22) with JIA were examined, 68 boys and 80 girls. The control group consisted of 38 healthy children (9 boys, 29 girls), their peers (14.72 ± 0.28 years old). JADAS 27 disease activity was assessed. All patients received methotrexate treatment for at least 12 months. Kidney function were evaluated based on: creatinine, urea, albuminuria, density, glomerular filtration rate. Groups were formed based on glomerular filtration rate (GFR) levels and were divided by quartiles (lower scores that were below the 25th percentile, average scores between the 25th and 75th percentiles, and upper scores above the 75th percentile). Heart parameters (left and right ventricles) were determined using ultrasound. All parameters were statistically significant (р < 0,05).

**Results:** End-diastolic size, end-diastolic volume and stroke volume of the left ventricle were significantly lower in patients with lower and average scores of GFR compared with upper one. All patients with JIA had increased right ventricular end-systolic size, end-systolic and end-diastolic volumes and the highest levels was in lower scores group. Significant increase in stroke volume and minute volume of the right ventricle was also observed in lower scores group.

A decrease in the ejection fraction of the right ventricle was determined in all patients with JIA, it was the lowest in upper scores group.

**Conclusion:** The interplay between cardiorenal connectors and hemodynamic ultrasound parameters changes based on kidney work, precisely their long-term preservation, is crucial to future cardiorenal syndrome development in patients with JIA.

**Patient Consent**

Yes, I received consent

**Disclosure of Interest**

None Declared

### JIA (oligo, poly, psoriatic)

#### P228 Translation and validation in italian of the methotrexate intolerance severity score for children and adults with arthritis

##### Giusyda Tarantino^1^, Rebecca Nicolai^1^, Angela Aquilani^1^, Andrea Tommasini^2^, Antonella Celano^2^, Aurora Pucacco^1^, Silvia Magni Manzoni^1^, Fabrizio De Benedetti^1^, Emiliano Marasco^1^

###### ^1^Rheumatology Division, IRCCS Bambino Gesù Children's Hospital, Rome; ^2^APMARR, Lecce, Italy

####### **Correspondence:** Giusyda Tarantino

*Pediatric Rheumatology 2024*, **22(2)**: PReS24-ABS-1588

**Introduction:** Methotrexate (MTX) is the most used drug for the treatment of children and adults suffering from arthritis although its intake is burdened by well-known side effects. The Methotrexate Intolerance Severity Score (MISS) was originally developed in English [1] to identify patients intolerant to MTX.

**Objectives:** Methotrexate (MTX) is the most used drug to treat children and adults with arthritis and its use is burdened by adverse effects. The MTX intolerance severity score (MISS) was developed in English to identify patients who are intolerant to MTX. The aim of this study was to translate and validate the MISS in Italian.

**Methods:** The Italian version of the MISS was developed following the “guidelines for process of cross-cultural adaptation of self-reported measures”. The Italian version of the MISS was validated in 125 patients with juvenile idiopathic arthritis (JIA) followed at the Rheumatology Unit of Bambino Gesù Children Hospital. We assessed the construct validity and calculated the internal consistency of the Italian MISS. We performed ROC analysis to assess the overall performance of the Italian MISS.

**Results:** Of the study cohort (83% female), 71 patients had oligoarthritis, 43 had RF-negative polyarthritis, 4 had enthesitis-related arthritis (ERA), and 3 had psoriatic arthritis. The mean age (±IQR) at diagnosis was approximately 4 years, with a "disease duration" on the MISS questionnaire of 6 years (IQR 3.3-10.5). The mean (± SD) VAS and c-JADAS scores were 0.7 and 2.4 respectively, with 65% of patients having inactive disease. The majority (98%) of patients received MTX subcutaneously and the average duration of use of the drug was approximately 5 years. We translated and adapted the MISS to the Italian language. The Italian MISS showed a very good internal consistency as shown by a Cronbach a of 0.87 (95% CI, 0.84-0.90) and a composite reliability of 0.89 (95% CI, 0.83-0.91). A threshold of 6 to define intolerant patients, showed a sensitivity of 98.3% and specificity of 81.2%.

**Conclusion:** We developed the Italian version of the MISS and showed its validity and reliability to identify patients intolerant to MTX in clinical practice and in a research setting.

**Patient Consent**

Yes, I received consent

**Disclosure of Interest**

None Declared

**References**

1. M. Bulatović et al., “High prevalence of methotrexate intolerance in juvenile idiopathic arthritis: development and validation of a methotrexate intolerance severity score.” Arthritis Rheum 63, 2007-2013 (2011).
2. D. Wild et al., “Principles of Good Practice for the Translation and Cultural Adaptation Process for Patient-Reported Outcomes (PRO) Measures: report of the ISPOR Task Force for Translation and Cultural Adaptation.” Value Health 8, 94-104 (2005).

### JIA (oligo, poly, psoriatic)

#### P229 Systemic treatment does not increase success rate of talonavicular corticosteroid injection with ultrasound guidance

##### Marco Burrone^1, 2^, Francesco Pasetti^3^, Clara Malattia^1, 2^, Sara Perissi^3^, Silvia Rosina^2^, Stefania Viola^2^, Roberta Caorsi^2^, Caterina Matucci-Cerinic^1, 2^, Valentina Natoli^1, 2^, Claudio Lavarello^1, 2^, Francesca Ridella^1^, Silvia M. Orsi^1^, Stefano Volpi^1, 2^, Riccardo Papa^2^, Angelo Ravelli^1, 4^, Marco Gattorno^2^, Alessandro Consolaro^1, 2^

###### ^1^Dipartimento di Neuroscienze, Riabilitazione, Oftalmologia, Genetica e Scienze Materno Infantili (DINOGMI), Università degli Studi di Genova; ^2^UOC Reumatologia e Malattie Autoinfiammatorie; ^3^UOC Radiologia; ^4^Direzione Scientifica, IRCCS Istituto Giannina Gaslini, Genoa, Italy

####### **Correspondence:** Marco Burrone

*Pediatric Rheumatology 2024*, **22(2)**: PReS24-ABS-1366

**Introduction:** Intraarticular corticosteroid injections (IACI) are widely used in the management of juvenile idiopathic arthritis (JIA) to induce rapid relief of symptoms of active synovitis and to obtain resolution of functional impairment. In recent years, ultrasound (US) has been increasingly used as guidance to IACI in JIA patients. Ankle joints (as well as wrist joints) are known to present lower success rate after IACI than other joints, possibly due to the relative difficulty in assessing this joint only clinically. US guidance, therefore, is of particular help in guiding talonavicular joint injection.

**Objectives:** To evaluate the response of talonavicular synovitis to local steroid therapy driven by US in children with JIA.

**Methods:** This is a retrospective study of 29 consecutive patients with 37 talonavicular synovitis confirmed by US examination. Five patients had two affected feet. All 29 patients enrolled in the study had a diagnosis of JIA and were treated and followed up at the Department of Pediatric Rheumatology, IRCCS Gaslini, Genoa. Joint injection was performed by the interventional radiologist with US guidance and methylprednisolone acetate (MPA) was injected by the pediatric rheumatologist. The dose of MPA injected was 20-40 mg based on patient’s body weight. All patients and their parents were instructed to avoid activity or weight bearing for a period of 24 hours. Patients were stratified into two groups according to the therapy at the time of IACI: Group 1 = none/unchanged therapy; Group 2 = upgraded therapy. Response to treatment was evaluated at the following visit 2 to 6 months after IACI. A successful treatment response was defined as absence of swelling in the injected joint, and absence of pain/tenderness on motion and a normal range of motion. The rate of successful IACI in the two groups was compared with Fisher’s exact test.

**Results:** A successful treatment response was observed in 23/37 (62%) cases. Twenty-six IACI were inserted in Group 1 and 11 IACI in Group 2. The successful treatment response rate was similar in the two groups (p = 0.99). No side effects such as subcutaneous tissue atrophy were observed in tarsal region of any patients during the follow-up time.

**Conclusion:** Talonavicular corticosteroid injection with US guidance was safe and moderately effective in our cohort. The concomitant modification of systemic treatment did not significantly affect success rate at first follow up visit

**Patient Consent**

Yes, I received consent

**Disclosure of Interest**

None Declared

### JIA (oligo, poly, psoriatic)

#### P230 Familial autoimmune diseases in Juvenile idiopathic arthritis

##### Brigita Koren, Vojko Berce

###### Pediatric clinic, University Medical Centre , Maribor, Slovenia

####### **Correspondence:** Brigita Koren

*Pediatric Rheumatology 2024*, **22(2)**: PReS24-ABS-1085

**Introduction:** Autoimmune diseases are relatively common, estimated to affect 5 to 10% of the population. There is evidence for clustering of autoimmunity in individuals and families.

**Objectives:** To evaluate the prevalence of familial autoimmune diseases in children with different subtypes of juvenile idiopathic arthritis (JIA).

**Methods:** A retrospective, single small center study was performed. Patients diagnosed with JIA who were examined at our pediatric clinic in the last 10 years were enrolled. JIA was classified according to International League Against Rheumatism (ILAR) criteria. Data was collected from patients’ medical records.

**Results:** A total of 115 patients were included. The mean age of children with JIA was 8.1 years. 74 patients (64.3%) were female.

Altogether, 29 patients with JIA (25.2%) had familial autoimmunity. Among them 2 children had more than 1 family member with autoimmune disease. Familial autoimmunity was most commonly present in the group of children with rheumatoid factor positive polyarthritis (all 3 patients, 100%), then in the psoriatic arthritis (8 patients, 53.3%), enthesitis related arthritis (8 patients, 30.7%), rheumatoid factor negative polyarthritis (13 patients, 23%), systemic arthritis (1 patient, 20%) and oligoarthritis (8 patients, 16%). 50% of children with oligoarthritis who had familial autoimmune diseases were antinuclear antibody (ANA) positive. 3 patients were diagnosed with undifferentiated arthritis and they had no familial autoimmunity.

The most common familial autoimmune diseases were psoriasis, rheumatoid arthritis and psoriatic arthritis. Other familial autoimmune diseases were JIA, autoimmune thyroid disease and ankylosing spondylitis.

**Conclusion:** Our study demonstrated that family members of children with JIA have a higher prevalence of autoimmune diseases. A family history of autoimmune diseases is associated with JIA subtype, as well as with ANA and RF positivity.

**Patient Consent**

Not applicable (there are no patient data)

**Disclosure of Interest**

None Declared

**References**

1. Van Straalen JW, de Roock S, Giancane G et al. Prevalence of familial autoimmune diseases in juvenile idiopathic arthritis: results from the international Pharmachild registry. Pediatric Rheumatology 2022; 20:103.
2. Prahalad S, McCracken CE, Ponder LA et al. Familial autoimmunity in the childhood arthritis and rheumatology research alliance registry. Pediatric Rheumatology 2016; 14:14.

### JIA (oligo, poly, psoriatic)

#### P231 Clinical and anamnestic determinants of treatment and prognosis in children with Juvenile idiopathic arthritis

##### Maria Francesca Gicchino, Alessia Amodio, Dario Piatto, Emanuele Miraglia del Giudice, Alma N. Olivieri, Anna Di Sessa

###### University of the Study of Campania L.Vanvitelli, Naples, Italy

####### **Correspondence:** Maria Francesca Gicchino

*Pediatric Rheumatology 2024*, **22(2)**: PReS24-ABS-1325

**Introduction:** Juvenile idiopathic arthritis (JIA) is the most common pediatric rheumatic disease. Significant improvements have been reported in the therapeutic JIA landscape with the advent of different medications (e.g. methotrexate (MTX), biologic drugs).

Since its relevant health burden and its marked variability also across the seven different subtypes, an appropriate treatment is crucial. Young age at the disease onset (≤ 6 years), high level of disease activity at onset, presence of uveitis, polyarticular course, wrist, hip, and ankles involvement at onset have been considered as predictors of poor prognosis among patient with JIA, but potential factors influencing JIA treatment are still lacking.

**Objectives:** To investigate potential factors associated with JIA treatment in young patients.

**Methods:** We revaluated 113 children with JIA classified according to ILAR criteria attending our Rheumatology Clinic. At the time of the first visit, anamnestic , anthropometric and laboratory data were recorded. Age at disease onset, JIA subtype, disease duration, joint involvements, presence of comorbidities, and type of medications were also collected. Disease activity was assessed by JADAS-10. Disease flare was defined according to Wallace criteria.

**Results:** Patients showed a mean age of 7.57±4.20. JIA subtypes of our cohort were as follows: 41% persistent oligoarticular, 9.1% extended oligoarticular, 23.6% RF- polyarticular, 8.2% Rheumatoid Factor (RF)+ polyarticular, 7.3% systemic, 8.2% enthesitis-related arthritis, and 2.7% psoriatic arthritis. MTX was administered in 62.4 % of patients, biological drugs in 40%. Patients receiving MTX showed a significant lower sacroiliac, hip, midfoot, and knees involvement (p=0.001, p=0.04, p=0.01, and p=0.002, respectively) and a higher bilateral ankle involvement (p=0.002). These patients also had a lower wrist, temporomandibular, and elbow joint involvement (p=0.0008, p=0.02, and p=0.04). Children with JIA receiving biological drugs presented with reduced sacroiliac and hip involvement (p=0.03 and p=0.004, respectively) and an increased bilateral ankle involvement (p<0.0001). These patients also had a lower wrist joint involvement (p<0.0001), while a trend for temporomandibular joint was found (p=0.06).

Among anamnestic factors, family history for autoimmune diseases showed a significant association with wrist joint involvement (p=0.04).

Relapses was also positively correlated to wrist (p=0.04), ankle (p=0.01), and temporomandibular (p=0.002) involvement. Shorter intervals free from JIA relapses were also found in patients with hip involvement (p=0.02). An increased disease activity in JIA patients with knee and hip involvement was also found (p=0.04 and p=0.03, respectively).

**Conclusion:** Children with JIA receiving MTX or biological drugs presented with a defined joint involvement pattern. While a reduced sacroiliac, hip, and wrist and a higher bilateral ankle involvement were shared between the two groups, patients treated with MTX showed a significant reduced involvement of temporomandibular, knee, and midfoot joints. Family history for autoimmune disease was associated with wrist involvement. Noteworthy, certain joints also showed a prognostic value on JIA course.

Further longitudinal studies are needed to better define predictors of JIA treatment and prognosis.

**Patient Consent**

Yes, I received consent

**Disclosure of Interest**

None Declared

### JIA (oligo, poly, psoriatic)

#### P232 Efficacy and safety of different corticosteroid regimens used in the management of children and young people with newly diagnosed Juvenile idiopathic arthritis before and during the covid-19 pandemic

##### Maysoon Elfadil^1^, Darcy Horler^2^, Tania Amin^3^, Daniel Hawley^1^

###### ^1^Paediatric Rheumatology , Sheffield Children's Hospital NHS Foundation Trust; ^2^Medical school, University of Sheffield , Sheffield; ^3^Paediatric Rheumatology , Leeds Children's Hospital , Leeds, United Kingdom

####### **Correspondence:** Maysoon Elfadil

*Pediatric Rheumatology 2024*, **22(2)**: PReS24-ABS-1543

**Introduction:** Juvenile Idiopathic Arthritis (JIA) is a chronic autoimmune disease affecting children and young people (CYP). Corticosteroids (CS) are commonly used to control inflammation in JIA, yet optimal regimens remain uncertain.

**Objectives:** To describe and compare the efficacy and safety of various CS regimens used in the management of newly diagnosed JIA in Yorkshire and the Humber region, UK, before and during the COVID-19 pandemic.

**Methods:** A retrospective observational study was conducted at Leeds Children's Hospital (LCH) and Sheffield Children's Hospital (SCH). Data were collected for two, one-year periods, before and after the pandemic. All CYP with a new diagnosis of JIA in the region were included. Patients with systemic JIA or other active inflammatory conditions were excluded. Differing CS modalities including intra-articular corticosteroid injection (IACI), oral prednisolone and intravenous methylprednisolone (IVMP) were analysed for efficacy, in reducing active joint counts (AJC) and the need for further treatment, and the rate of associated side-effects including adrenal suppression, weight gain and severe infections, in relation to the type of CS used.

**Results:** A total of 115 CYP newly diagnosed with JIA were included in this study with 58 cases identified pre-pandemic. No significant changes in demographics and baseline disease characteristics were observed during the COVID-19 pandemic compared to the pre-pandemic period. The female-to-male ratio was 2.1:1 pre-pandemic and 1.4:1 during the pandemic. Two prominent age groups identified at diagnosis were children under 5 years and those over 10 years of age. Oligoarticular JIA was the predominant subtype, representing 55% of cases pre-pandemic and 58% of cases during the pandemic.

There was no significant change in the choice of CS modality during the pandemic, though a slight increase in oral CS use was observed in polyarticular-pattern JIA patients (34% vs 19%). The preferred CS therapy for oligoarticular JIA was IACI, resulting in a 60.5% average reduction in active joint count. In polyarticular-pattern JIA, all CS modalities demonstrated efficacy in reducing active joint count. Notably, oral CS alone showed the highest average reduction in active joint count (76%), while IACI had the lowest efficacy (36%), although not statistically significant *(p=0.505).*

No severe systemic infections requiring hospitalization were reported within the first six months of CS therapy. There were no cases of psychosis, but two CYP exhibited mood changes or drowsiness on oral CS. Weight gain was observed but did not significantly differ between IVMP and oral CS groups (mean=28.9g/kg & 28.1g/kg respectively). Adrenal suppression was seen in one patient.

**Conclusion:** Baseline characteristics and CS regimen choice remained stable before and during the pandemic. All CS modalities effectively reduced active joint counts in polyarticular-pattern JIA, with oral CS demonstrating the highest efficacy, while IACI had the lowest efficacy, supporting the use of systemic CS in the treatment of CYP with newly diagnosed polyarticular-pattern JIA. Further research is needed to explore the efficacy of oral CS at varying doses to reduce the burden associated with systemic CS side-effects.

**Patient Consent**

Not applicable (there are no patient data)

**Disclosure of Interest**

None Declared

### JIA (oligo, poly, psoriatic)

#### P223 Identification of antigen targets of recombinant monoclonal antibodies from JIA patients’ synovial B-Cells

##### Tobias Schmidt^1, 2, 3^, Claudia Vollmuth^4^, Johannes Dirks^4^, Henner Morbach^4^, Anki Mossberg^1, 2^, Robin Kahn^1, 2^

###### ^1^Division of Pediatrics, Department of Clinical Sciences Lund; ^2^Wallenberg Center for Molecular Medicine; ^3^Division of Rheumatology, Department of Clinical Sciences Lund, Lund University, Lund, Sweden; ^4^Department of Pediatrics, University Hospital Würzburg, Würzburg, Germany

####### **Correspondence:** Anki Mossberg

*Pediatric Rheumatology 2024*, **22(2)**: PReS24-ABS-1691

**Introduction:** Autoantibodies, such as antinuclear autoantibodies (ANAs) are important drivers in autoimmune diseases, as they target endogenous antigens, resulting in an immune reaction to the host. However, the role of autoantibodies in the pathogenesis of juvenile idiopathic arthritis (JIA), the most common rheumatic disease in children, is still not clear.

**Objectives:** The aim of this study is to identify specific antigens targeted by combining the findings from 1) recombinant monoclonal antibodies generated from single synovial B-cells and 2) serum from JIA patients.

**Methods:** Recombinant monoclonal antibodies (n=9), generated from single synovial B cells of four different JIA patients, were produced as previously described (1). Roughly half of the isolated antibodies were confirmed for autoreactivity when tested using a commercial ANA test. The recombinant antibodies were crosslinked to Protein G coated magnetic beads and used for immunoprecipitation (IP) with whole cell protein extracts. Bound proteins were identified using Mass Spectrometry (MS). In parallel, serum from JIA patients (n=11) were used for IP using similar protein extracts. Proteins identified in both experimental setups were subjected to further validation with specific ELISAs.

**Results:** In all experimental conditions, ribosomal proteins, histones, and proteins of the glycolytic pathways were identified by MS among the precipitated proteins. Of these possible antigens, several have previously been identified in other autoimmune diseases, such as Fibronectin, Vitronectin, snRNPs and heat shock proteins. Importantly, only four proteins were identified in all settings and were therefore selected for validation experiments. A previously collected cohort of JIA sera (n=103) will be screened using specific ELISAs to the four identified proteins, which is now under development.

**Conclusion:** In conclusion, we identified several potential autoantigens in JIA using recombinant monoclonal antibodies and serum from JIA patients. The consistent identification of certain proteins across the experimental setups shows promise for further research. Future steps include additional identification of antigens, validation experiments with specific ELISAs and screening of a larger JIA cohort.

**Patient Consent**

Not applicable (there are no patient data)

**Disclosure of Interest**

None Declared

**Reference**

1. Smith, K., Garman, L., Wrammert, J. *et al.* Rapid generation of fully human monoclonal antibodies specific to a vaccinating antigen. *Nat Protoc* 4, 372–384 (2009). 10.1038/nprot.2009.3.

### JIA (oligo, poly, psoriatic)

#### P234 A clinico-epidemiological profile and assessment of disease status of patients with Juvenile idiopathic arthritis: a cross-sectional study

##### Piyush Chauhan, Avinash Sharma, Sandesh Guleria

###### Pediatrics , Dr Rajendra Prasad Government Medical College , Kangra, India

####### **Correspondence:** Piyush Chauhan

*Pediatric Rheumatology 2024*, **22(2)**: PReS24-ABS-1443

**Introduction:** Arthritis is one of the common manifestations that brings children to pediatric clinics. Juvenile Idiopathic Arthritis (JIA) is the most common among all chronic childhood arthritis. The term JIA includes several different subtypes, with unifying feature being arthritis for more than 6 weeks in one or more joints. It is challenging to assess outcomes in condition like JIA where there is no gold standard laboratory parameter. So various outcome measures have been designed for this purpose. The outcome measure for evaluating the disease activity in JIA includes the scoring system Juvenile Arthritis Disease Activity Score (JADAS) and for damage, the score used is Juvenile Arthritis Damage Index (JADI). JADI has 2 components with include JADI-A (articular) and JADI-E (extra-articular).

**Objectives:** To assess short-term outcome in children with JIA from Sub-Himalayan region in North-West India with limited exposure to biologics.

**Methods:** An observational cross-sectional study was performed in the Pediatric Rheumatology Clinic in department of pediatrics at Dr Rajendra Prasad government medical college, Himachal-Pradesh, India. All patients diagnosed with JIA according to International League of Associations for Rheumatology (ILAR) criteria were enrolled. Study duration was 1 year from May 2023 to April 2024. All the newly diagnosed and previously diagnosed patients were enrolled and disease activity and damage were assessed using JADAS and JADI respectively.

**Results:** A total of 50 patients (37 boys) with disease duration of less than 5 years were enrolled with the mean age of 10.7 **±** 4.82 years. Patients who came for a routine follow-up visit were evaluated and those who were not coming for regular follow-up were called telephonically as per their convenience. Commonest diagnosis was enthesitis related arthritis (ERA) in 34 (68%) patients followed by oligoarthritis 10 (20%). Mean disease duration was 2.52 **±** 1.57 years. 26 patients (52%) were in remission, out of which 12 patients (24%) were on medications. Twenty-four (48%) had active disease with mean JADAS of 5.57. Ten patients (20%) were on NSAIDs and 13 (26%) were on disease modifying anti rheumatic drugs, 4 patients (8%) were receiving biologicals. Highest frequency of patients who achieved remission off medication were with ERA (34%) and lowest had polyarthritis. Out of 10 patients of oligoarthritis 5 patients had uveitis and all were ANA positive. Two patients (4%) had articular damage (multiple joint contractures and valgus deformity of knee) and 6 patients (12%) had extraarticular damage. Most common extra articular damage was damage to eyes, other were growth failure and pubertal delay.

**Conclusion:** ERA was the commonest form of JIA seen in this study. Only a handful of patients were on biologicals. After a disease duration ranging from 6 months to 5 years, 24 patients had ongoing disease activity as assessed using JADAS. Forty eight percent had active disease despite being on treatment.

Disease activity and damage scores must be used to evaluate ongoing course of disease in JIA, which may help make treatment decisions, esp. where biologicals are not readily available and are often not the initial treatment of choice.

**Date of birth::** juillet 15

**Patient Consent**

Yes, I received consent

**Disclosure of Interest**

None Declared

### JIA (oligo, poly, psoriatic)

#### P235 The childhood arthritis southwest sweden inception cohort (classic): a new population-based cohort study of Juvenile idiopathic arthritis (JIA)

##### Michael Damgaard^1^, Elisabet Berthold^2^, Jenny Lingman Framme^1^, Susanne Lindgren^1, 3^, Robin Kahn^4^

###### ^1^Department of Pediatrics, Institute of Clinical Sciences, The Sahlgrenska Academy, University of Gothenburg, Gothenburg; ^2^Department of Rheumatology, Clinical Sciences Lund, Lund University, Lund; ^3^Department of Rheumatology and Inflammation Research, The Sahlgrenska Academy, University of Gothenburg, Gothenburg; ^4^Department of Pediatrics, Clinical Sciences Lund, Lund University, Lund, Sweden

####### **Correspondence:** Michael Damgaard

*Pediatric Rheumatology 2024*, **22(2)**: PReS24-ABS-1101

**Introduction:** The treatment of JIA has changed dramatically in the last three decades as new, effective immunosuppressive drugs have been developed. Treatment is now introduced earlier in the course of the disease, to reduce inflammation and preserve joint function. Children diagnosed with JIA today are believed to have a better prognosis than those diagnosed previously, but scientific support is scarce.

The Childhood Arthritis Southwest Sweden Inception Cohort (CLASSIC) study is a population-based observational study of newly diagnosed JIA patients which is started as an effort to create a solid ground for evidence-based knowledge about the early phases of JIA from a clinical and immunological viewpoint.

**Objectives:** Primary aims are to study the incidence of JIA and to describe disease manifestations, including uveitis and articular damage, given the contemporary treatment regimens. Secondary aims are to study if clinical variables or immunological biomarkers assessed at diagnosis can be used to predict treatment response and outcome.

**Methods:** All patients who are diagnosed with JIA in a well-defined region, during a 3-year period, will be asked to participate. The region consists of 4 counties in the southwest of Sweden, with a total population of 3.7 million inhabitants, corresponding to 35% of the Swedish population. Inclusion should preferably be within 1 month from diagnosis and before any intra-articular or systemic steroids or DMARDs are started. Patient demographics and clinical data including treatment regimens, disease activity scores and patient/parent reported outcome measures will be recorded in the Swedish Pediatric Rheumatology Quality Registry at all visits for 3 years. The data from the visits that fall closest to 3, 6, 12, 24 and 36 months will be extracted for analysis. A subset of patients will have samples from peripheral blood and synovial fluid for analysis of cytokines, cell populations using flow cytometry as well as RNA/DNA sequencing.

**Results:** An estimated 300 patients will be included, starting from May 2024. 1 year data will be available in 2028 and final 3 year data in 2030.

**Conclusion:** This new prospective, population-based cohort of approximately 300 patients with newly diagnosed JIA is being created to add important insight in the early phases of JIA.

**Patient Consent**

Not applicable (there are no patient data)

**Disclosure of Interest**

None Declared

### JIA (oligo, poly, psoriatic)

#### P236 Evaluation of fear of falling in patients with Juvenile idiopathic arthritis

##### Pelin Esmeray Şenol^1^, Emre Leventoğlu^2^, Batuhan Küçükali^1^, Çisem Yıldız^1^, Nihal Karaçayır^1^, Merve Kutlar Tanıdır^1^, Nuran Belder^1^, Deniz Gezgin Yıldırım^1^, Sevcan Bakkaloğlu^1^

###### ^1^Pediatric Rheumatology, Gazi University, Ankara; ^2^Pediatric Nephrology, Konya City Hospital, Konya, Türkiye

####### **Correspondence:** Pelin Esmeray Şenol

*Pediatric Rheumatology 2024*, **22(2)**: PReS24-ABS-1190

**Introduction:** Juvenile idiopathic arthritis (JIA) is a chronic rheumatic disease characterized by arthritis. The fear of falling (FOF) negatively impacts the quality of life of JIA patients through restriction of patients' ability to maintain their daily and physical activity levels. Causes of FOF may include joint pain, imbalance, muscle weakness, side effects of medications, the presence of uveitis, and restrictions on physical activities. There are many studies evaluating the FOF in adults with chronic diseases (1,2), while pediatric data are limited (3,4) and no study regarding JIA. In our study, we aimed to evaluate the FOF in JIA.

**Objectives:** 34 patients with JIA and 15 patients with recurrent urinary tract infections (UTI) between September and December 2023 were included in the study. A questionnaire consisting of 34 questions, lasting approximately 15 minutes to assess FOF were completed by parents or children over 6 years old. The FOF assessment scale developed by İpek et al. in children with neuromuscular disease was used in the study (4).

**Results:** The study included 34 patients with JIA and 15 patients with recurrent UTIs as the control group. Nineteen (55.9%) of the JIA patients and six (40%) of the control group were female. The mean age was 12.5 (±1.8) years in the JIA group and 15.2 (±3.8) years in the control group (p>0.05). All JIA patients had lower extremity involvement during their disease process. The mean disease duration was 75.5 (±45.9) months. There were no differences in any domain of FOF between JIA and UTI patients. Only 2 (5.9%) JIA patients indicated an increase in falls after diagnosis.

**Conclusion:** This study is important because it is the first study evaluating the FOF in patients with JIA, and established a significant FOF. This study is the initial step of the development of a specific questionnaire to assess the FOF in JIA patients. With these evaluations, it can be aimed to improve the quality of life of JIA patients by providing appropriate psychological support.

**Date of birth::** octobre 22

**Patient Consent**

Yes, I received consent

**Disclosure of Interest**

None Declared

**References**

1. Oliveira CC, McGinley J, Lee AL, Irving LB, Denehy L. Fear of falling in people with chronic obstructive pulmonary disease. Respiratory medicine. 2015;109(4):483-9.
2. Mehdizadeh M, Martinez-Martin P, Habibi SA, Nikbakht N, Alvandi F, Bazipoor P, et al. The association of balance, fear of falling, and daily activities with drug phases and severity of disease in patients with Parkinson. Basic and clinical neuroscience. 2019;10(4):355.
3. Fong SS, Ng SS, Chung LM, Ki W, Chow LP, Macfarlane DJ. Direction-specific impairment of stability limits and falls in children with developmental coordination disorder: implications for rehabilitation. Gait & posture. 2016;43:60-4.
4. İpek C, Yilmaz Ö, Karaduman A, Alemdaroğlu-Gürbüz İ. Development of a questionnaire to assess fear of falling in children with neuromuscular diseases. Journal of Pediatric Orthopaedics B. 2021;30(5):494-9.

### JIA (oligo, poly, psoriatic)

#### P237 The pain and behavioral traits of early Juvenile arthritis induced by collagen ii in rat models

##### Nihal Sahin^1^, Gülşen Çelebi^2^, Yunus E. Bayrak^1^, Gözde Tüfekçi^2^, Semil S. Göçmez^2^, Hafize E. Sönmez^1^, Tijen Utkan^2^

###### ^1^Pediatric Rheumatology; ^2^Pharmacology, Kocaeli University, Kocaeli, Türkiye

####### **Correspondence:** Nihal Sahin

*Pediatric Rheumatology 2024*, **22(2)**: PReS24-ABS-1442

**Introduction:** Chronic rheumatic diseases, including juvenile idiopathic arthritis (JIA), can be associated with anxiety-depression-like behavior and cognitive decline. Collagen II-induced arthritis is the most commonly used rat model of rheumatoid arthritis. However, the characteristics of the collagen II-induced early juvenile arthritis model, which occurs in rats younger than five weeks before puberty, have not been clearly defined yet.

**Objectives:** The aim is to evaluate the relationship between JIA and pain, depressive behaviors, and cognitive changes by examining the characteristics of an early juvenile arthritis rat model.

**Methods:** Juvenile male Wistar albino rats were divided into three groups: control, arthritis, and sham. Arthritis was induced with collagen II emulsified in Freund's adjuvant in the arthritis group. Arthritis severity and pain were analyzed weekly. After six weeks, depression-like behavior and cognitive functions were evaluated.

**Results:** Initial joint diameters were similar across groups (p>0.05). Over weekly follow-ups, the arthritis group showed significantly increased joint diameters compared to others (p<0.05). The mechanical withdrawal threshold was consistently lower in the arthritis group for six weeks (p<0.05). The passive avoidance test had no significant difference (p>0.05). The arthritis group exhibited longer immobilization time in the modified forced swimming test (arthritis: 34.97±4.38 sec, control: 16.52±3.19 sec, sham: 21.35±3.79 sec; p=0.005).

**Conclusion:** Our investigation employing the early juvenile arthritis rat model has uncovered a dichotomy: in arthritis occurring at a remarkably young age, depression-like behaviors become conspicuous, while cognitive functions remain resilient.

**Funding:** This work has been supported by Kocaeli University Scientific Research Projects Coordination Unit under grant number TSA-2023-3336.

**Date of birth::** 21.09.1987

**Patient Consent**

Not applicable (there are no patient data)

**Disclosure of Interest**

None Declared

### JIA (oligo, poly, psoriatic)

#### P238 Evaluation of capillaroscopy findings in patients with Juvenile psoriatic arthritis

##### Bengisu Menentoğlu^1^, Ayşenur Doğru^1^, Gülşah Kavrul Kayaalp^1^, Selen D. Arık^1^, Fatma G. Demirkan^2^, Özlem Akgün^1^, Figen Çakmak^3^, Nuray Aktay Ayaz^1^

###### ^1^Pediatric rheumatology, Istanbul University Istanbul Faculty of Medicine; ^2^Kanuni Sultan Suleiman Training and Research Hospital; ^3^Çam and Sakura city hospital, İstanbul, Türkiye

####### **Correspondence:** Bengisu Menentoğlu

*Pediatric Rheumatology 2024*, **22(2)**: PReS24-ABS-1561

**Introduction:** Juvenile Psoriatic Arthritis (jPsA) represents about 7-10% of cases within the spectrum of Juvenile Idiopathic Arthritis. It can lead to joint deterioration and enduring deformities by causing peripheral arthritis, enthesitis, dactylitis, and sacroiliitis. Moreover, nail involvement manifests in roughly half of jPsA patients. Nailfold capillaroscopy is a valuable diagnostic tool that offers important prognostic information in the early phases of connective tissue diseases. Despite its prevalent use among individuals with Raynaud's phenomenon and systemic sclerosis, capillaroscopy is a well-established method for evaluating microcirculation, and is increasingly used for other childhood rheumatic diseases.

**Objectives:** In this study, we aimed to evaluate capillaroscopic findings of patients diagnosed with jPsA, and compare them with those of healthy peers.

**Methods:** Sixteen patients with jPsA followed up at the pediatric rheumatology outpatient clinic and sixteen age and gender-matched healthy children were included in the study. All fingers except the thumbs were examined with a digital videocapillaroscope, and 16 capillaroscopic images for each patient were evaluated in accordance with the standardization recommendations of the EULAR Study Group on Microcirculation in Rheumatic Diseases. Capillary morphology, capillary density, presence of microhemorrhages, avascular areas and capillary dilations were assessed and compared with healthy controls.

**Results:** Compared to healthy controls, higher rates of capillary tortuosity (p=0.02) and crossing capillaries (p=0.007) were observed in patients with jPsA. Apical loop width was found to be lower in jPsA patients compared to healthy controls (p=0.04). There was no significant difference between the two groups in terms of capillary morphology and capillary density. The presence of microhemorrhage, avascular area, neoangiogenesis, or abnormal capillary were similar in both gruops.

**Conclusion:** In this study, minor differences in capillaroscopic findings among patients with jPsA were identified. However, our analysis revealed that the overall capillaroscopy pattern remains unchanged in this condition. Further extensive research is required to ascertain any alterations in microcirculation in jPsA.

**Date of birth::** novembre 0

**Patient Consent**

Yes, I received consent

**Disclosure of Interest**

None Declared

**References**

1. Zaripova LN, Midgley A, Christmas SE, Beresford MW, Baildam EM, Oldershaw RA. Juvenile idiopathic arthritis: from aetiopathogenesis to therapeutic approaches. Pediatr Rheumatol Online J. 2021 Aug 23;19(1):135. doi: 10.1186/s12969-021-00629-8.
2. Gerhold K, Becker MO. Nailfold capillaroscopy in juvenile rheumatic diseases: known measures, patterns and indications. Clin Exp Rheumatol. 2014 Nov-Dec;32(6 Suppl 86):S-183-8.
3. Piotto DG, Len CA, Hilário MO, Terreri MT. Nailfold capillaroscopy in children and adolescents with rheumatic diseases. Rev Bras Reumatol. 2012 Oct;52(5):722-32.

### JIA (oligo, poly, psoriatic)

#### P239 The prevalence of juvenile idiopathic arthritis in Republic of Sakha (Yakutia)

##### Sargylana Boeskorova^1^, Marina Afonskaia^1^, Ludmila Leonteva^2^, Polina Sleptsova^1^, Vera Argunova^1^, Tatiana Burtseva^2, 3^, Mikhail Kostik^4^

###### ^1^Republican Hospital №1 - National Center of Medicine; ^2^Yakut science center of complex medical problems; ^3^North-Eastern Federal University, Yakutsk; ^4^Saint-Petersburg State Pediatric Medical University, Saint-Petersburg, Russian Federation

####### **Correspondence:** Mikhail Kostik

*Pediatric Rheumatology 2024*, **22(2)**: PReS24-ABS-1790

**Introduction:** Sakha (Yakutia) Republic has the biggest Asian population in Russia with indigenous peoples - Sakha, living in the Far North. Previous studies shown the high incidence of HLA B27 distribution in healthy Sakha people - 25%, which is similar to Native Americans from Alaska – the closest nationality to Sakha.

**Objectives:** Our study aimed to evaluate the prevalence of juvenile idiopathic arthritis (JIA) and distribution of its categories in people, living in Sakha Republic.

**Methods:** In the retrospective cohort study were included the data about all patients (n=225) with different JIA categories (<18 years) who were admitted to the single tertial pediatric rheumatology center in Yakutsk – the capital of Sakha Republic in 2018-2023 years.

**Results:** The prevalence of JIA in Sakha Republic is 86.9 per 100 000 children which is higher than in whole Russia 62.3 per 100 000 children. We observed the increasing incidence of new JIA patients since 9.1 per 100,000 children in 2016 to 11.7 per 100,000 children in 2021 and 15.5 per 100,000 children in 2023. The increase in incidence grows up +67% during the last five years.

The ethical distribution was 168 (74.7%)-Sakha, 49 (21.8%)-White/Caucasians and 8 (3.5%)-other nationalities. The Asians were 176 (78.2%) children.

The incidence of JIA is higher in children living in rural areas than in the city 55.5 vs 39.8 per 100,000 children.

The significantly higher incidence of JIA in Sakha-110.1 compared to White/Caucasians-69.4 per 100,000 children was observed. The highest incidence was in Sakha children, living in cities 139.4 than in rural territories 89.8 per 100,000 children.

Boys were-107 (47.6%) and girls were 118 (52.4%) with median onset age 9 (5; 12) years. The JIA categories distribution was systemic JIA-7 (3.1%), oligoarthritis-76 (33.8%), RF-negative polyarthritis-33 (14.7%), RF (+) polyarthritis-1 (0.4%), enthesitis-related arthritis-99 (44.0%) and psoriatic arthritis-7 (3.1%). The frequency of HLA B27 in whole JIA cohort was 87/220 (39.6%).

**Conclusion:** The prevalence of JIA in Sakha republic is higher than in whole Russia with predominant enthesitis-related arthritis. Sakha peoples predisposed to JIA higher than White/Caucasians.

**Patient Consent**

Not applicable (there are no patient data)

**Disclosure of Interest**

None Declared

### JIA (oligo, poly, psoriatic)

#### P240 Profile of anemia in patients with Juvenile idiopathic arthritis: a cross-sectional study

##### Jahnavi Shrivastava, Anu Maheshwari, Deonath Mahto, Lakhinana Kalicharan

###### Pediatrics, Lady Hardinge Medical College, Delhi, India

####### **Correspondence:** Jahnavi Shrivastava

*Pediatric Rheumatology 2024*, **22(2)**: PReS24-ABS-1362

**Introduction:** Anemia is a significant comorbidity in Juvenile Idiopathic Arthritis (JIA), yet the underlying causes remain underexplored [1]. In low and middle-income countries like India, micronutrient deficiencies (iron, folate and Vitamin B12 deficiencies) contribute substantially to anemia in JIA patients, potentially leading to inappropriate therapeutic interventions [2]. This study aimed to ascertain the prevalence and type of anemia in newly diagnosed JIA patients and investigate the correlation between hemoglobin levels and disease activity scores.

**Objectives:** To assess the type of anemia in newly diagnosed JIA patients and correlate hemoglobin levels with disease activity scores.

**Methods:** This cross-sectional study included thirty four newly diagnosed JIA patients based on ILAR criteria [3] and was conducted between 2018 and 2020 in a tertiary care center in North India. Children who had received blood component transfusions in the previous three months, had any other known chronic illness (renal disease, liver disease, endocrine disorders or transfusion dependent anemias) or had received treatment with methotrexate over the past three months were excluded. After taking consent of the parent/guardian, demographic data and JADAS-27 scores were recorded for each patient. Anemia was assessed and quantified according to the WHO guidelines, followed by further investigations including serum ferritin, serum transferrin receptors, direct Coomb’s test, iron studies, serum folate, and Vitamin B12 levels. Spearman correlation coefficient was calculated to evaluate the relationship between hemoglobin levels and JADAS-27 scores.

**Results:** This cross-sectional study included thirty four newly diagnosed JIA patients based on ILAR criteria and was conducted between 2018 and 2020 in a tertiary care center in North India. Children who had received blood component transfusions in the previous three months, had any other known chronic illness (renal disease, liver disease, endocrine disorders or transfusion dependent anemias) or had received treatment with methotrexate over the past three months were excluded. After taking consent of the parent/guardian, demographic data and JADAS-27 scores were recorded for each patient. Anemia was assessed and quantified according to the WHO guidelines, followed by further investigations including serum ferritin, serum transferrin receptors, direct Coomb’s test, iron studies, serum folate, and Vitamin B12 levels. Spearman correlation coefficient was calculated to evaluate the relationship between hemoglobin levels and JADAS-27 scores.

**Conclusion:** Given the prevalence of Iron Deficiency Anemia in JIA patients, hemoglobin levels should not be relied upon as a sole biomarker for therapy titration. Routine iron supplementation may be warranted in JIA patients to alleviate the burden of anemia and associated morbidity. Further research is needed to explore optimal therapeutic strategies for managing anemia in JIA patients effectively.

**Date of birth::** juin 09, Y

**Patient Consent**

Yes, I received consent

**Disclosure of Interest**

None Declared

**References**

1. Egorov A, Chasnyk V, Kostik M, Snegireva L, Kalashnikova O, Dubko M, *et al*. Anemia in children with JIA: is it really driven by hepcidin level, or by a set of factors of a chronic disease. Pediatric Rheumatology. 2014;12:1-2.
2. Agrawal S, Misra R, Aggarwal A. Anemia in rheumatoid arthritis: high prevalence of iron-deficiency anemia in Indian patients. Rheumatology international. 2006;26:1091-1095.
3. Petty RE, Southwood TR, Manners P, Baum J, Glass DN, Goldenberg J, et al. International League of Associations for Rheumatology. International League of Associations for Rheumatology classification of juvenile idiopathic arthritis: second revision, Edmonton, 2001. J Rheumatol. 2004;31:390-392.

### JIA (oligo, poly, psoriatic)

JIA (oligo, poly, psoriatic)

#### P241 The first analisys of the moscow registry of children with Juvenile idiopathic arthritis

##### Vladislav Sevostyanov^1^, Seda Kurbanova^2^, Saniya Valieva^2^, Denis Rassokha^1^, Nikita Babich^1^, Elena Zholobova^3^

###### ^1^Department of Propaedeutics of Childhood Diseases, I.M. Sechenov First Moscow State Medical University, ^2^Department of Rheumatology, Moscow City Center of Pediatric Rheumatology, Morozov Children's City Clinical Hospital, ^3^Department of Childhood Diseases, I.M. Sechenov First Moscow State Medical University, Moscow, Russian Federation

####### **Correspondence:** Vladislav Sevostyanov

*Pediatric Rheumatology 2024*, **22(2)**: PReS24-ABS-1109

**Introduction:** Juvenile idiopathic arthritis (JIA) is one of the most common rheumatic diseases in children. JIA is characterized by a variety of clinical manifestations and subsets, which often complicates diagnosis and a timely treatment.

**Objectives:** Using data from the Moscow JIA Registry (ARTEC) the general characteristics of the population of patients with JIA were assessed to obtain initial demographic and clinical data.

**Methods:** ARTEC is an ongoing observational registry of patients with JIA as ILAR criteria receiving biologics or synthetic disease-modifying anti-rheumatic drugs (cDMARDs). An observational descriptive cross-sectional study was conducted. For qualitative data, frequencies (percentage) were reported, while quantitative data were expressed in terms of medians with first and third quartiles.

**Results:** The study included 1350 patients with JIA aged 0 to 17 years, of which 866 were girls (64.2%) and 484 (35.8%) were boys. The JIA category distribution differed among registries, but the most frequent JIA category was rheumatoid factor (RF) – negative polyarthritis (range of 24.6%–29.9%). The frequency of oligoarticular JIA was higher in the Swedish registry (49.6% versus about 30.5%–37.1% in the other two registries), while in BiKeR the frequencies of oligo- and poly-articular JIA RF-negative were similar (about 30%). In the structure of JIA, the most frequent JIA category was oligoarticular variant (range 52.4%), 32.1% was (RF)-negative polyarthritis, 9.7% was systemic variant, enthesit-related, polyarticular (RF)-positive and psoriatic JIA were 3.8%, 1.1% and 0.9% respectively. It was revealed that the earliest onset of JIA was observed in systemic, oligoarticular, are polyarticular seronegative variants; the median onset was at the age of 4 years (Q1–Q3: 2–9; 2–7; 2–7, respectively). The median onset of JIA in the psoriatic and enthesitis variants was 12 years (Q1–Q3: 11–13; 10–13, respectively), and in the (RF)-positive polyarthritis – 13 years (Q1–Q3: 11–13). The median interval from the onset of the disease to verification of the diagnosis for all variants was about 2 months. Uveitis was diagnosed in 11.9% of patients with JIA (n=160). The incidence of uveitis among boys was 8.5% (n=41), among girls – 13.7% (n=119), while the risk of uveitis in girls are 1.7 times higher than in boys: OR=1.730 95 % CI [1.167–2.563] (p=0.006). Basic anti-inflammatory therapy was used in 79.0% of patients with JIA (n=1067). Among them, 86.9% patients received methotrexate, 10.7% - sulfasalazine. 680 (50.4%) patients with JIA receive biological therapy, of them etanercept – 46.3%, adalimumab – 28.1%, tocilizumab – 11.3%, golimumab – 5.2%, secukinumab – 4.4%, abatacept – 2.8%, canakinumab – 1.9%. Tofacitinib, an oral Janus kinase inhibitor, was administered in 42 patients with JIA in Moscow.

**Conclusion:** The analysis demonstrated that diagnosis of JIA in Moscow for all variants is early - the median interval from the onset of the disease to verification of the diagnosis was 2 months. Experience in optimizing the process of management the children with JIA has shown the effectiveness of such organizational measures as using a centralized patients registry with analysis of the structure and course of JIA and planning of the necessary drug therapy.

**Patient Consent**

Yes, I received consent

**Disclosure of Interest**

V. Sevostyanov Grant / Research Support with: Research was supported by an independent grant from Pfizer, S. Kurbanova: None Declared, S. Valieva: None Declared, D. Rassokha: None Declared, N. Babich: None Declared, E. Zholobova: None Declared

### JIA (oligo, poly, psoriatic)

#### P242 Proteinuria and hematuria as early signs of renal involvement in Juvenile idiopathic arthritis

##### Emanuela Del Giudice on behalf of Emanuela Del Giudice*, Alessia Marcellino, Sara Hoxha, Vanessa Martucci, Mariateresa Sanseviero, Silvia Bloise, Sara Giovanna De Maria, Maria Rita Leone, Flavia Ventriglia, Riccardo Lubrano

###### Department of Maternal Infantile and Urological Sciences, Sapienza University of Rome, Polo Pontino, Latina, Italy

####### **Correspondence:** Emanuela Del Giudice

*Pediatric Rheumatology 2024*, **22(2)**: PReS24-ABS-1302

**Introduction:** Juvenile idiopathic arthritis (JIA) is the most common rheumatic disease in childhood and according to ILAR classification different JIA categories can be identified, with distinct clinical symptoms and disease outcomes.

In addition to pathognomonic joint disease, JIA is also burdened by extra-articular involvement and comorbidities , the most frequent of which are uveitis and cardiovascular complications. However, the pathogenesis of renal involvement in JIA is poorly understood, it may develop because of subclinical endothelial damage due to the inflammatory systemic process itself, leading to kidney disease, cardiovascular disease up to hypertension in children with JIA.

Despite the known correlation between chronic inflammation and risk of early endothelial dysfunction and renal damage described in adults with RA (rheumatoid arthritis), there are only few published data assessing the renal involvement risk in children with JIA.

In fact, there is no evidence in literature about an extensive evaluation of renal function and 24-h ambulatory blood pressure monitoring (ABPM) in children affected by JIA.

**Objectives:** To evaluate glomerular and tubular renal functions and analyze blood pressure in a cohort of pediatric patients with juvenile idiopathic arthritis (JIA) compared to healthy controls and to establish the possible relationship with disease activity in JIA patients.

**Methods:** A total of 40 pediatric patients, 20 (50%) with JIA and 20 (50%) healthy control subjects, were studied and performed the renal function on 24-hour collection and the 24-h ambulatory blood pressure monitoring (ABPM). Moreover, we compared renal function and blood pressure trends between the groups of JIA patients with different disease activities.

**Results:** No statistically significant differences were observed between patients with JIA and healthy children in terms of glomerular filtration rate (GFR), fractional excretion of sodium (FENa), tubular reabsorption of phosphate (TRP), and calcium-creatinine urine ratio (CaU/CrU). In contrast, we observed significantly higher values in JIA patients than in controls for the presence of hematuria (p<0.0001) and proteinuria (p<0.0001). Compared to the control group there were significantly higher values of hematuria and proteinuria/day in both groups of JIA patients with low disease activity (respectively, p=0.0001 and p=0.0002) and moderate disease activity (respectively p=0.0001 and p=0.0012). Systolic and diastolic dipping were significantly reduced in patients with JIA compared with healthy controls (p<0.0001 and p<0.0001, respectively).

**Conclusion:** Our study showed that children with JIA, already in the early stages of the disease, have higher values of hematuria and proteinuria, which are early warning signs of nephropathy. Therefore, detailed screening of renal function and pressure monitoring in patients are necessary to monitor their evolution over time.

**Patient Consent**

Yes, I received consent

**Disclosure of Interest**

None Declared

**References**

1. Ponticelli C, Doria A, Moroni G. Renal disorders in rheumatologic diseases: the spectrum is changing (part 2. Arthridides). J Nephrol. 2021;34(4):1081–90.
2. Cafarotti A, Marcovecchio ML, Lapergola G, Di Battista C, Marsili M, Basilico R, et al.Kidney function and renal resistive index in children with juvenile idiopathic arthritis. Clin Exp Med. 21**;**2022

### JIA (oligo, poly, psoriatic)

#### P243 Study of correlation of serum vitamin d levels with disease activity in children with juvenile idiopathic arthritis

##### Jahnavi Shrivastava, Anu Maheshwari, Deonath Mahto, Barkha Dhanjani

###### Pediatrics, Lady Hardinge Medical College, Delhi, India

####### **Correspondence:** Jahnavi Shrivastava

*Pediatric Rheumatology 2024*, **22(2)**: PReS24-ABS-1304

**Introduction:** Juvenile Idiopathic Arthritis (JIA) is a multifactorial chronic arthritis with immune dysregulation. Various studies in literature suggest a negative correlation between vitamin D levels and JIA disease activity, using Vitamin D as an immunomodulator [1,2,3]. Our study examines the relationship between Vitamin D levels and disease activity scores in treatment naive children with JIA.

**Objectives:** To investigate the correlation between serum vitamin D levels and disease activity in treatment naïve children with JIA.

**Methods:** This was prospective observational study conducted over two years from 2020 to 2022 after approval of the institutional ethics committee. Thirty five children diagnosed with JIA according to the ILAR criteria [4] were enrolled after taking consent from parents. Children with subtypes of JIA other than oligoarticular and polyarticular JIA, patients of chronic disease (liver disease, diabetes mellitus, malabsorption syndromes), or on antiepileptics, steroids or Vitamin D supplements were excluded. Disease activity was assessed using the JADAS-27 score along with assessment of serum Vitamin D levels via Electrochemiluminiscence protein binding immunoassay. Spearman correlation coefficients between vitamin D and JADAS-27 were calculated at presentation and after 3 months of treatment in vitamin D-deficient patients.

**Results:** Of the 35 patients (18 females, 17 males), median age was 6 years (IQR 4-11 years). Rheumatoid factor (RF) positive polyarticular JIA was the commonest subtype (51.4%), followed by oligoarticular JIA (34.3%) and RF negative polyarthritis (14.3%). Median JADAS-27 scores at diagnosis were 24.2 in patients with vitamin D levels <20ng/ml (labelled as group A) and 36.45 in those with sufficient vitamin D levels (>20ng/ml) (labelled group B) (p = 0.38). Median change in JADAS-27 score after vitamin D therapy was 3.90 (IQR: 1-14.90) in group A and 5.60 (IQR: 0-14) in group B (p=0.70). The delta change in JADAS 27 score after 3 months was higher in group A, though statistically insignificant, indicated a trend towards better response with vitamin D supplementation. Spearman correlation coefficient showed a weak negative correlation (r = -0.07; p=0.65) between vitamin D levels and JADAS-27 scores.

**Conclusion:** While no correlation was found between disease activity and serum 25(OH)D3 at diagnosis, a trend suggests a better response to DMARD treatment in patients with sufficient vitamin D levels. Further investigation is warranted to elucidate the role of vitamin D in JIA management.

**Date of birth::** juin 09, Y

**Patient Consent**

Yes, I received consent

**Disclosure of Interest**

None Declared

**References**

1. Pelajo CF, Lopez-Benitez JM, Kent DM, Price LL, Miller LC, Dawson-Hughes B. 25-hydroxyvitamin D levels and Juvenile idiopathic arthritis: is there an association with disease activity? Rheumatol Int. 2012;32:3923–3929.
2. Çomak E, Doğan ÇS, Uslu-Gökçeoğlu A, Akbaş H, Özdem S, Koyun M, et al. Association between vitamin D deficiency and disease activity in Juvenile idiopathic arthritis. Turk J Pediatr. 2014;56:626–631.
3. Sengler C, Zink J, Klotsche J, Niewerth M, Liedmann I, Horneff G, et al. Vitamin D deficiency is associated with higher disease activity and the risk for uveitis in Juvenile idiopathic arthritis - data from a German inception cohort. Arthritis Res Ther. 2018;20:276.
4. European League Against Rheumatism. EULAR Bulletin No. 4: Nomenclature and Classification of arthritis in children. Natl Ztg AG Basel. 1977.

### JIA (oligo, poly, psoriatic)

#### P244 Arthritic enigma: when a simple solution is provided, is it final and enough? - a case report

##### Ante Vidović, Ivana Radoš, Barbara Perše, Orjena Žaja, Mandica Vidović

###### Department of Pediatrics, Sestre milosrdnice University Hospital Centre, Zagreb, Croatia

####### **Correspondence:** Mandica Vidović

*Pediatric Rheumatology 2024*, **22(2)**: PReS24-ABS-1316

**Introduction:** Joint manifestations are common finding across a spectrum of medical conditions, ranging from autoimmune disorders like juvenile idiopathic arthritis (JIA) or systemic lupus eritematosus, infectious diseases such as Lyme disease or as a reactive arthritis. These manifestations encompass a diverse array of symptoms, including arthritis, arthralgia and joint deformities, often posing diagnostic challenges due to overlapping clinical presentations. Understanding the joint involvement in these diseases is crucial for precise diagnosis and efficient treatment. Thus, investigating patient epidemiology, clinical presentations, underlying pathophysiological mechanisms and ensuring regular follow-up are essential.

**Objectives:** The aim of this case report is to illustrate instances where joint abnormalities may serve as a manifestation of non-rheumatological conditions. On the other hand, they may also signify a concurrent rheumatological disorder alongside another disease capable of causing arthralgia or arthritis.

**Methods:** An 8-year-old male presented with a six-month history of arthralgia affecting the proximal interphalangeal joint of the third finger of the left hand and both knees, accompanied by limited mobility of the left hand fingers. No history of trauma was reported. Ultrasound examination revealed effusion and synovitis in the aforementioned joints. Nonsteroidal anti-inflammatory drug (NSAID) therapy was initiated, and following comprehensive rheumatological assessment, elevated levels of anti-tissue transglutaminase antibodies (anti-tTG) were detected. After further gastrointestinal evaluation (repeated anti-tTG measurement and biopsy of duodenum), diagnosis of coeliac disease was confirmed, prompting initiation of an exclusive gluten-free diet along with NSAIDs to mitigate inflammation. The arthralgia and arthritis were attributed to extraintestinal manifestations of immune-mediated mechanisms triggered by gluten exposure. Despite reduction in anti-tTG levels on the gluten-free regimen, joint symptoms persisted, with effusions in both knees, prompting further diagnostic evaluation. Magnetic resonance imaging of the knees revealed characteristic changes indicative of chronic arthritis and intra-articular corticosteroid therapy was administered, resulting in improvement of knee symptoms and NSAID therapy continued; rheumatological check-up is pending.

**Conclusion:** In summary, this case highlights the significance of considering coeliac disease as a potential underlying cause of arthritis, especially in the absence or subtlety of gastrointestinal symptoms. It emphasizes the necessity of including serological screening for coeliac disease (measurements of anti-tTG and total immunoglobulin A concentration) in the evaluation of patients presenting with new-onset arthritis. Timely recognition and management of coeliac-related arthritis are critical for alleviating patient discomfort and mitigating long-term joint complications. However, in cases where joint symptoms persist despite strict adherence to a gluten-free diet, clinicians should consider the possibility of concurrent conditions such as juvenile idiopathic arthritis, which is why long-term follow-up is recommended.

**Patient Consent**

Yes, I received consent

**Disclosure of Interest**

None Declared

**References**

1. Bourne JT, Kumar P, Huskisson EC, Mageed R, Unsworth DJ, Wojtulewski JA. Arthritis and coeliac disease. Ann Rheum Dis. 1985 Sep;44(9):592-8. doi: 10.1136/ard.44.9.592. PMID: 3876079; PMCID: PMC1001716.
2. Kim KH, Kim DS. Juvenile idiopathic arthritis: Diagnosis and differential diagnosis. Korean J Pediatr. 2010 Nov;53(11):931-5. doi: 10.3345/kjp.2010.53.11.931. Epub 2010 Nov 30. PMID: 21218014; PMCID: PMC3012272.

### JIA (oligo, poly, psoriatic)

#### P245 Is assessment of cytokine levels good tool for diagnostics of Juvenile idiopathic arthritis: the preliminary data?

##### Lubov Sorokina^1^, Daria Kozlova^2, 3^, Vitaly Khizha^3^, Karina Yureva^3, 4^, Mikhail Kostik^1^

###### ^1^Saint-Petersburg State Pediatric Medical University; ^2^Saint-Petersburg Clinical Hospital of the Russian Academy of Sciences; ^3^Sechenov Institute of Evolutionary Physiology and Biochemistry of the Russian Academy of Sciences (IEPhB RAS) ; ^4^Saint-Petersburg State University, Saint-Petersburg, Russian Federation

####### **Correspondence:** Mikhail Kostik

*Pediatric Rheumatology 2024*, **22(2)**: PReS24-ABS-1726

**Introduction:** Plasma cytokine levels consider a possible tool for assessment of the disease activity and might be used for diagnostics.

**Objectives:** The aim of our study was to determine the cytokines spectrum the most sensitive and specific for the JIA diagnosis.

**Methods:** Thirty plasma samples from JIA patients (14 boys and 16 girls with a mean age of 12.2±4.1 years) and 20 samples from healthy individuals (10 boys and 10 girls; mean age 10.2±5.9 years) were collected and then analyzed using the MILLIPLEX® kit to determine a wide pool of cytokines content. In addition, the content of 14-3-3 eta protein and calprotectin were determined using solid-phase ELISA. Statistical processing was carried out using the GraphPad Prism 8 (GraphPad Software, Inc., Boston, MA, USA) software package. For determination of the most sensitive and specific markers, “multivariate analysis of variance” was used. Eight blinded blood samples were used for validation of the results. Informed consent was obtained from all subjects involved in this study.

**Results:** The most specific cytokines for JIA patients were: IL-17F, IL-27, protein 14-3-3 eta and calprotectin. The levels of IL-17F (158.0±55.1 pg/mL) and IL-27 (12,2±1,4 pg/mL) were 5 times higher in JIA patients, compared to healthy controls, and the levels of protein 14-3-3 eta (62.7±11.4 pg/mL) and calprotectin (5.1±3.9 pg/mL) were increased 4 and 6 times, respectively. Based on the obtained markers, blind testing of 8 unknown diagnosis samples was performed. According to the results of the analysis, 5 patients from the analyzed sample were diagnosed with JIA, which was subsequently confirmed when the data was verified with protocols received from rheumatologists.

**Conclusion:** The complex assessment of these four markers could be used to expand the laboratory confirmation of the JIA diagnosis.

**Patient Consent**

Not applicable (there are no patient data)

**Funding**

The research was carried out with the support of state assignment No. 075-00264-24-00.

**Disclosure of Interest**

None Declared

### JIA (oligo, poly, psoriatic)

#### P246 Macrophage activation syndrome (mas) and Juvenile idiopathic arthritis (JIA): is it always a systemic subtype?

##### Maria Teresa Riccio, Andrea Lo Vecchio, Eugenia Bruzzese, Vincenza Pontillo, Maria Alessio

###### Traslational Medical Science, Section of Pediatrics, University of Naples Federico II, Naples, Italy

####### **Correspondence:** Maria Teresa Riccio

*Pediatric Rheumatology 2024*, **22(2)**: PReS24-ABS-1776

**Introduction:** Juvenile idiopathic arthritis (JIA) is a pediatric condition involving chronic arthritis and potential complications in the eyes, skin, and internal organs, sometimes leading to severe disability or fatality. Subtypes include oligoarticular, polyarticular, systemic, psoriatic arthritis, and enthesitis-related arthritis. Systemic JIA (s JIA) may trigger life-threatening macrophage activation syndrome (MAS). Diagnosis relies on clinical evaluation, family history, laboratory tests, and imaging. Early treatment is crucial for better outcomes.

**Objectives:** To demonstrate the importance of biomarkers in distinguishing different forms of JIA.

**Methods:** D., a 3-year-old girl diagnosed with ANA-positive oligoarticular juvenile idiopathic arthritis (JIA) and treated with methotrexate, was admitted with fever and arthritis of the right knee. Laboratory findings indicated elevated ferritin levels (>50,000), AST at 117, and triglycerides at 201, alongside lymphadenopathy and polyarthritis. Based on Ravelli's criteria, she was diagnosed with systemic juvenile idiopathic arthritis (sJIA) complicated by macrophage activation syndrome (MAS) and treated with three boluses of methylprednisolone (30 mg/kg) and subcutaneous Anakinra (8 mg/kg), resulting in rapid improvement. Due to persistent arthritis and new joint involvement, such as the wrists and left knee, tocilizumab therapy (12 mg/kg) was initiated after a month, discontinuing Anakinra and starting corticosteroid tapering with partial benefit. Subsequent worsening of articular symptoms led to the introduction of cyclosporine two months later. A new articular flare-up occurred two months thereafter, involving multiple joints as confirmed by ultrasound. This necessitated increased corticosteroid dosage with subsequent improvement. However, attempts at corticosteroid tapering resulted in symptom exacerbation, prompting administration of three boluses of methylprednisolone (30 mg/kg) followed by maintenance therapy with prednisone.

**Results:** Considering the polyarticular involvement and absence of systemic symptoms, tocilizumab therapy was discontinued in favor of initiating treatment with methotrexate and etanercept, which led to significant improvement and resolution of symptoms. This prompted a reevaluation of the initial diagnosis, confirming polyarticular juvenile idiopathic arthritis.

**Conclusion:** This clinical case aims to highlight the need of biomarkers in distinguishing different forms of JIA. The absence of a specific biomarker made it difficult to identify the correct diagnosis due to the subtle clinical presentation. Moreover, MAS is a known complication of sJIA. In this instance, we reported MAS secondary to polyarticular JIA.

**Date of birth::** août 04, Y

**Patient Consent**

Yes, I received consent

**Disclosure of Interest**

None Declared

### JIA (oligo, poly, psoriatic)

#### P247 A case of malignancy in a patient with Juvenile idiopathic arthritis

##### Eka Nakhutsrishvili, Tinatin Kutubidze

###### Tbilisi State Medical University, Tbilisi, Georgia

####### **Correspondence:** Eka Nakhutsrishvili

*Pediatric Rheumatology 2024*, **22(2)**: PReS24-ABS-1385

**Introduction:** Juvenile idiopathic arthritis (JIA) comprises a group of heterogenous disorders characterized by childhood-onset chronic joint inflammation. It is the most common rheumatologic disease in the pediatric population and an important cause of chronic illness in children. Without treatment, juvenile idiopathic arthritis (JIA) may have devastating consequences.The long-term impact of active disease in JIA on cancer risks is only partly understood. The few available studies suggest that patients with JIA may be at increased risk for lymphoproliferative malignancies in childhood and early adulthood.

**Objectives:** In recent years, concern has been raised about Juvenile Idiopathic Arthritis (JIA) that it could be associated with an increased risk for malignancies.

**Methods:** Here we present case of 15 years old girl with JIA and osteosarcoma.

**Results:** The disease started at the age of 5 with arthritis of the left knee joint (swelling, pain). She was consulted by a paediatric rheumatologist, complex studies were performed, and she was diagnosed with juvenile idiopathic arthritis, oligoarticular form, seropositive (ANA-1:2560, RF-pos, synovitis by ultrasound of the knee joint). At that stage, the treatment was NSAID, an intra-articular corticosteroid. According to the parent's report, the condition improved due to the treatment, but since then, the visit to the rheumatologist has been unsystematic, and despite the complaints for 10 years, no DMARD treatment has been performed. It should be noted that the patient's younger sister was also diagnosed with juvenile idiopathic arthritis at the age of 1 and was under the supervision of a rheumatologist. Currently, the patient came to our clinic with complaints: pain in the left knee joint, asymmetric disfigurement, limitation of walking movement, morning stiffness for 20–25 minutes. During the examination, attention was drawn to crepitation during passive movement and asymmetric deformity of the left knee and joint. The patient immediately underwent an MRI of the joint, where changes characteristic of osteosarcoma were observed.

**Conclusion:** The risk of cancer, including any secular trends in risk, in patients with juvenile idiopathic arthritis (JIA) is incompletely understood. Children and adolescents with JIA are at a slightly increased risk of lymphoproliferative malignancies. At the group level, there is no sign that this risk has increased further after the introduction of bDMARDs.

**Date of birth::** août 15, Y

**Patient Consent**

Yes, I received consent

**Disclosure of Interest**

None Declared

**References**

1. Stoll ML, Cron RQ.Treatment of juvenile idiopathic arthritis: a revolution in care. Pediatr Rheumatol Online J2014;12.
2. Shoop-Worrall SJW, Verstappen SMM,Baildam E, et al. How common is clinically inactive disease in a prospective cohort of patients with juvenile idiopathic arthritis? the importance of definition. Ann Rheum Dis2017;76:1381–8.
3. Nordstrom BL, Mines D, Gu Y, et al. Risk of malignancy in children with juvenile idiopathic arthritis not treated with biologic agents. Arthritis Care Res2012;64:1357–64.

### JIA (oligo, poly, psoriatic)

#### P248 A decade of pediatric rheumatology: tracking Juvenile idiopathic arthritis cases at faro hospital

##### Ana Chícharo^1^, Graça Sequeira^1, 2, 3^

###### ^1^Rheumatology, Faro Hospital, Faro, ^2^Faculdade de Medicina, Universidade Católica de Lisboa, Lisboa, ^3^Mestrado integrado de Medicina, Universidade do Algarve, Faro, Portugal

####### **Correspondence:** Graça Sequeira

*Pediatric Rheumatology 2024*, **22(2)**: PReS24-ABS-1412

**Introduction:** Juvenile idiopathic arthritis (JIA) encompasses a heterogeneous group of inflammatory disorders mainly affecting the joints. According to the ILAR classification, JIA can be subdivided into systemic JIA, oligoarticular JIA, polyarticular JIA positive for rheumatoid factor (RF), polyarticular JIA negative for RF, psoriatic JIA, enthesitis-related arthritis, and undifferentiated arthritis. Each of these conditions requires early referral to a pediatric rheumatologist for proper monitoring and treatment. JIA represents the most common rheumatologic diagnosis in pediatric age, with an estimated annual incidence of 0.008–0.226 per 1000 children.

**Objectives:** The authors aim to characterize the population of children diagnosed with JIA followed at Faro Hospital between 2011 and 2021.

**Methods:** An observational study with a retrospective analysis was conducted. Data were obtained from medical records and Portuguese national registry *Reuma.pt*.

**Results:** Thirty-five children with a JIA diagnosis were included, 20 girls (57.1%) and 15 boys (42.9%). Oligoarticular JIA was the most frequent subtype observed (19, 54.3%), followed by enthesitis-related JIA (8, 22.9%), systemic JIA (4, 11.4%), seronegative polyarticular JIA (3, 8.6%), and seropositive polyarticular JIA (1, 2.9%). No cases of psoriatic or undifferentiated JIA were identified. The mean age of symptom onset was 8.0 years, while the mean age at diagnosis was 8.7 years. Extra-articular manifestations, such as fever, rash, uveitis and colitis, were collected. Antibody profile (antinuclear antibodies, RF, anti-CCP) and HLA-B27 status were also reported. Most children were referred to Pediatric Rheumatology by their general practitioner (11, 31.4%) or the Pediatrics Department at Faro Hospital (7, 20.0%). Disease activity was recorded at the beginning and the end of follow-up of these children. In the last available follow-up visit, 12 children were in remission while on treatment (57%), 6 were in remission without treatment, keeping clinical surveillance (29%), and 3 continued to experience disease activity, necessitating medication adjustments.

Regarding therapy, only a minority of children (4, 11.4%) received biologic therapy. By the end of 2021, 2 children remained under bDMARD therapy, both under Infliximab. Other administered therapies were also identified, including cDMARDs such as methotrexate (19, 54.3%).

In 2021, 13 (37.1%) patients continued follow-up in Pediatric Rheumatology, 8 (22.9%) were transferred to General Rheumatology due to reaching 18 years of age, the others were transferred to other hospitals or lost their follow-up.

**Conclusion:** This study provides the initial characterization of patients followed with a JIA diagnosis at Faro Hospital, contributing to a better understanding of the most important rheumatic disease in pediatric age.

**Patient Consent**

Not applicable (there are no patient data)

**Disclosure of Interest**

None Declared

### JIA (oligo, poly, psoriatic)

#### P249 Examining anthropometric measures and functional capacity in children with JIA undergoing bdmard treatment

##### Ante Vidović, Ivana Rados, Karla Rubelj, Mandica Vidović

###### Department of Pediatrics, Sestre milosrdnice University Hospital Center, Zagreb, Croatia

####### **Correspondence:** Mandica Vidović

*Pediatric Rheumatology 2024*, **22(2)**: PReS24-ABS-1341

**Introduction:** Juvenile idiopathic arthritis (JIA) encompasses a diverse array of rheumatological conditions marked by arthritis onset before the age of 16, constituting the most prevalent autoinflammatory disorder in children. An effective treatment aims to quell active joint inflammation, alleviate clinical symptoms thus allowing normal growth and development.

**Objectives:** The objective of this study was to assess anthropometric features and appraise the functional capacity of pediatric and adolescent patients diagnosed with JIA undergoing treatment with biological disease-modifying antirheumatic drugs (bDMARDs).

**Methods:** In this prospective open study, we enrolled 21 participants, comprising 3 boys and 18 girls. Data were extracted from their medical records to assess JIA disease activity using Juvenile Arthritis Disease Activity Score (JADAS). Additionally, we measured anthropometric parameters including body mass, height and skinfold measurements (determined using the 7-site Jackson & Pollock formula) which were assessed and percentiles were calculated. Furthermore, a physiotherapist evaluated participants using standardized objective functional diagnostic tests (handgrip strength, Y balance test and step test).

**Results:** One patient included in this study exhibited underweight status while undergoing tocilizumab therapy as the sole agent, with a recorded JADAS of 6. Despite her underweight status, she demonstrated normal performance on the hand grip, step and Y balance tests. Fourteen subjects exhibited BMI within the normal range, with eleven demonstrating normative results in functional assessments. Among the five overweight patients, distinct patterns of functional impairment were observed. All five of them underperformed on the step test.

**Conclusion:** Pediatric patients with well-managed JIA under bDMARD therapy did not exhibit statistically significant deviations in anthropometric measures or notable underperformance on functional assessments. Instances of functional underperformance were primarily attributed to disease relapse and the consequent impact of arthritis on the extremities, as well as higher BMI. Satisfactory disease control ensures adequate growth and functional development, also reducing the risk of injury. Following negative nutritional trends and rise of the obesity in children, patients with JIA should also be monitored for this developmental aspect.

**Patient Consent**

Yes, I received consent

**Disclosure of Interest**

None Declared

**References**

1. Zaripova LN, Midgley A, Christmas SE, Beresford MW, Baildam EM, Oldershaw RA. Juvenile idiopathic arthritis: from aetiopathogenesis to therapeutic approaches. Pediatr Rheumatol Online J. 2021 Aug 23;19(1):135. doi: 10.1186/s12969-021-00629-8. PMID: 34425842; PMCID: PMC8383464.
2. Schwiertz G, Brueckner D, Beurskens R, Muehlbauer T. Lower Quarter Y Balance Test performance: Reference values for healthy youth aged 10 to 17 years. Gait Posture. 2020 Jul;80:148-154. doi:10.1016/j.gaitpost.2020.05.041. Epub 2020 Jun 1. PMID: 32505979.
3. Nieczuja-Dwojacka J, Marchewka J, Siniarska A, Budnik A, Popielarz K, Tabak I. Influence of body build on hand grip strength, simple reaction time and strength of the abdominal muscles in prepubertal children. Anthropol Anz. 2023 Mar 16;80(2):151-158. doi: 10.1127/anthranz/2023/1591. PMID: 36752666.
4. Jacks, Dean & Moore, Justin & Topp, Robert & Bibeau, Wendy. (2008). Prediction of VO2 Peak Using a Sub-maximal Bench Step Test in Children: 2268. Medicine and Science in Sports and Exercise – MED SCI SPORT EXERCISE. 40. 10.1249/01.mss.0000322780.29703.13.

### JIA (oligo, poly, psoriatic)

#### P250 Patterns and predictors of juvenile idiopathic arthritis in the masovian region: a retrospective analysis from 2018 to 2022

##### Lidia Rutkowska-Sak, Iryna Naishtetik, Joanna Wojtowicz, Piotr Gietka

###### Pediatric Rheumatology Department, National Institute of Geriatrics, Rheumatology and Rehabilitation (NIGRIR), Warsaw, Poland

####### **Correspondence:** Joanna Wojtowicz

*Pediatric Rheumatology 2024*, **22(2)**: PReS24-ABS-1663

**Introduction:** Juvenile Idiopathic Arthritis (JIA) is a heterogeneous group of diseases characterized by joint inflammation and onset prior to 16 years of age [1]. Estimates suggest that the number of children with JIA in Poland ranges from 6,996 to 8,523, with an incidence rate between 5-9.5 per 100,000 children annually [2,3].

**Objectives:** The aim of this study was to analyze the incidence of JIA in the Masovian region, focusing on variations by age and sex, the duration between disease onset and diagnosis, and the prevalence of a family history of rheumatic diseases among children with JIA.

**Methods:** This retrospective study reviewed medical records from 780 children evaluated at the Outpatient Pediatric Clinic of the Institute of Rheumatology between 2018 and 2022.

**Results:** The overall incidence of JIA in this cohort was determined to be 5.09 cases per 100,000 children per year. Age-specific incidence rates showed the highest frequency in children aged 6-12 years at 5.42/100,000, followed by those under 5 years at 5.94/100,000, and the lowest in teenagers (13-18 years) at 3.96/100,000. Diagnostic timelines indicated that 56.5% of the cases were identified within the first year following onset, with an additional 38.5% diagnosed by the second year. Only about 5% of cases experienced diagnostic delays exceeding two years. The incidence of JIA was notably higher in girls than in boys, with rates of 5.90 versus 2.91 per 100,000 per year, respectively. A familial history of rheumatic diseases was reported in 7.17% of the cohort, with rheumatoid arthritis (26 cases), ankylosing spondylitis (8 cases), and psoriatic arthritis (9 cases) being the most common.

**Conclusion:** The findings from this study underscore the importance of early diagnosis and intervention in managing JIA. The higher incidence in girls and children between 6-12 years suggests a targeted need for preventative strategies in these groups. Furthermore, the occurrence of rheumatic diseases in family histories suggests more research into the genetic and environmental elements influencing the development of JIA.This study highlights critical epidemiological insights that could inform public health strategies and clinical practices in the region.

**Patient Consent**

Yes, I received consent

**Disclosure of Interest**

None Declared

**References**

1. Petty RE, Southwood TR, Manners P, Baum J, Glass DN, Goldenberg J, et al. International League of Associations for Rheumatology classification of juvenile idiopathic arthritis: second revision, Edmonton, 2001. J Rheumatol 2004; 31: 390–2.
2. Wolny-Niedzielska A. Choroby układu ruchu u dzieci kierowanych do Poradni Reumatologicznej w Kielcach w latach 1999–2003, Reumatologia 2005; 43: 265-273.
3. Żuber Z, Kania U, Król-Zdechlikiewicz A, et al. Analysis of clinical symptoms and laboratory profiles in children with juvenile idiopathic arthritis in Malopolska region (Poland) in the Years 2007-2010. Mac J Med Scien 2014; 7: 56-61.

### Juvenile dermatomyositis

#### P253 A single centre experience of switching from intravenous to subcutaneous immunoglobin as treatment for Juvenile dermatomyositis (JDM)

##### Neil Martin, Chris King, Andrew Fell

###### Royal Hospital for Children, Glasgow, United Kingdom

####### **Correspondence:** Chris King

*Pediatric Rheumatology 2024*, **22(2)**: PReS24-ABS-1768

**Introduction:** Intravenous Immunoglobulin(IVIg) is recommended as a treatment for refractory JDM, but side effects are common, particularly headache, which can be severe.

**Objectives:** We report our experience of using Subcutaneous Immunoglobulin(SCIg) as an alternative treatment for patients with JDM, particularly those who developed severe headaches associated with Intravenous Immunoglobulin(IVIg).

**Methods:** All patients who had received SCIG as treatment for JDM at our hospital since 2012 were identified. Clinical details were outlined by retrospective case review. Patients and their families were asked for their opinion on SCIG, including tolerability, side effects, ease of administration and overall impression.

**Results:** 3 patients were included. All had typical JDM rash, raised muscle enzymes and MRI findings in keeping with myositis. 2 were female. 2 patients had skin disease refractory to standard first line treatment using steroids and methotrexate with time from diagnosis to starting IVIg of 22 and 48 months respectively. A third patient started IVIg during his initial admission at diagnosis alongside steroids and Methotrexate as treatment for severe, ulcerative skin disease in the context of MDA5 positive antibody and hyperferritinaemia. All patients received IVIg 2g/kg monthly for a minimum of two months (2-12 months) prior to SCIg. All reported debilitating headaches following IVIg. Starting dose of SCIg was 0.2-0.3g/kg/wk. Families were trained to give SCIg by a nurse specialist and equipment for home administration was obtained. After switching to SCIg all patients reported improved tolerability and significant reduction or cessation of headache following treatment. No loss of efficacy was seen and all subsequently achieved clinically inactive disease. All reported improved tolerability and satisfaction with SCIg. One patient stopped SCIg on 2 separate occasions due to inactive disease state but on both occasions JDM rash subsequently flared and SCIg was restarted with positive clinical effect. The other 2 patients both achieved clinically inactive disease and have successfully stopped SCIg.

**Conclusion:** SCIg may provide an effective alternative to IVIg as treatment for severe or refractory JDM with less adverse effects, particularly severe headache. Moving from parenteral hospital administration to home administration requires training and supply of medication and equipment. Due to safety action notices, infusion devices required special exemption to allow their use in this situation. Despite this complex process, families appreciated the effort was worth the resulting improvement in health and family life. In our patients, no apparent reduction in efficacy was seen, with significant improvements in tolerability and patient satisfaction as well as reduced time missed from school to attend infusions. Further research, such as a non inferiority trial comparing SCIg with IVIg, would be helpful to assess efficacy, safety and tolerability of SCIg as a treatment for refractory JDM.

**Patient Consent**

Yes, I received consent

**Disclosure of Interest**

None Declared

### Juvenile dermatomyositis

#### P254 Should capillaroscopy be trusted in the management of patients with Juvenile dermatomyositis?

##### Natalia Yudkina, Maria Kaleda, Irina Nikishina, Alexander Volkov

###### V.A. Nasonova Research Institute of Rheumatology , Moscow, Russian Federation

####### **Correspondence:** Irina Nikishina

*Pediatric Rheumatology 2024*, **22(2)**: PReS24-ABS-1773

**Introduction:** Considering the often early disability of patients with juvenile dermatomyositis (jDM), a search is being carried out, among other things, for instrumental diagnostic methods that make it possible to early suspect the disease and predict the response to therapy. Nailfold videocapillaroscopy (NVC), an accessible and non-invasive method for assessing microangiopathy, has shown promise, but should it be trusted?

**Objectives:** To determine the role of NVC in the management of patients with jDM.

**Methods:** The study included 30 children with a definite diagnosis of jDM according to the EULAR/ACR 2017 criteria. All patients had typical skin changes, weakness of the muscles of the shoulder and pelvic girdle of varying degrees of severity, increased serum levels of CPK, and a primary muscle pattern according to needle electromyography. All patients underwent NVC using a stereomicroscope with 200x magnification and displaying images on a widescreen monitor. The study was performed on 2-5 fingers of both hands, assessing the density, size, shape of capillaries and the presence of hemorrhages. NVC was performed at the time of diagnosis of dermatomyositis and after 6, 12, 18, 24 months against the background of pathogenetic therapy.

**Results:** Of the 30 examined patients with jDM, pronounced changes in the vessels of the microvasculature were detected, often visualized with the naked eye. In the vast majority of patients they were similar to systemic sclerosis (SSc). In children, a relationship was identified between the severity of the skin process and the pathology of the capillaries, while the correlation between myopathy and changes in the periungual bed was not established. Mixed vascular disorders (abnormally shaped capillaries, giant capillaries, formation of avascular areas, hemorrhages) were observed in patients with a disease duration of more than 6 months and no therapy. Against the background of active treatment, after 6, 12, 18 and 24 months, all children achieved a decrease in disease activity and/or remission, which affected the condition of the periungual bed in the form of transformation into nonspecific changes in the capillaries.

**Conclusion:** NVC is a useful tool to complement the diagnosis of jDM. But the method cannot be considered as a reliable assistant, since the type of changes in the capillaries is similar to SSc or is of a nonspecific nature, disguised as other nosologies, reflects the severity of an already easily distinguishable skin process, and changes against the background of active therapy for dermatomyositis.

**Patient Consent**

Not applicable (there are no patient data)

**Disclosure of Interest**

None Declared

### Juvenile dermatomyositis

#### P255 Capillaroscopic findings and disease activity in Juvenile dermatomyositis: a year-long prospective study

##### Kadir Ulu^1^, Rana İşgüder^2^, Şeyma Türkmen^1^, Şengül Çağlayan^1^, Balahan Makay^2^, Erbil Ünsal^2^, Betül Sözeri^1^

###### ^1^Department of Pediatric Rheumatology, Umraniye Training and Research Hospital, University of Health Sciences, İstanbul; ^2^Department of Pediatric Rheumatology, Dokuz Eylül University, Faculty of Medicine, İzmir, Türkiye

####### **Correspondence:** Kadir Ulu

*Pediatric Rheumatology 2024*, **22(2)**: PReS24-ABS-1792

**Introduction:** Capillaroscopy is a non-invasive imaging technique used for the evaluation and follow-up of juvenile dermatomyositis (JDM) patients. It provides valuable insights into the microcirculation of the nailfold capillaries, aiding in the diagnosis and monitoring of disease activity. Serial capillaroscopy examinations can track changes in capillary morphology, indicating disease flares or remission. Data on the use of capillaroscopy in children and adolescents with JDM are limited.

**Objectives:** This study aims to prospectively assess the relationship between capillaroscopic changes and disease activity in juvenile dermatomyositis patients.

**Methods:** Capillaroscopy images of 19 JDM patients followed in two tertiary paediatric rheumatology centres were assessed in a total of 72 visits at 3-month intervals. Thirty-two images were captured with 200x magnification, four images from each of the eight fingers of each patient. The area of one mm was evaluated for each image. Visits in which eight clear images could not be obtained due to technical reasons were excluded from the study.

**Results:** Fifteen (78%) of the 19 patients were female. The mean age at diagnosis for the patients was 7.2 (±3.3) years. 2304 images were analyzed. Major abnormalities were found on capillaroscopic images in 76% of visits. The mean capillary density was calculated to be 4.89 (±1.9) and the mean apical loop width was calculated to be 30 (±21) μm in all images obtained. The results indicate a moderate positive correlation between CMAS and capillary density mean (p=0.05, 95% CI (-0.0002, 0.4805)). Correlations between CMAS and capillary, arterial part, venous part, and apical loop widths were generally low or very weak, and no strong correlations were observed. Capillary density showed a moderate negative correlation with both the physician VAS and the patient/parent VAS (p=0.012, p=0.021). The CMAS scores were found to be significantly lower in the presence of dilated capillaries, bushy capillaries, neoangiogenesis and avascular areas. (p=0.013, p=0.001, p=0,002, p=0.005). In the follow-up visits, a significant increase in capillary density was found between the first and last visit (p=0.005).

**Conclusion:** The study highlights the value of capillaroscopy as a non-invasive tool for monitoring disease activity in JDM. The observed correlations between capillary density and disease activity scores indicate that capillaroscopic findings can provide valuable insights into disease progression and response to therapy. The significant increase in capillary density over the follow-up period provides further support for the utility of capillaroscopy in evaluating therapeutic outcomes in JDM patients.

**Date of birth::** janvier 01

**Patient Consent**

Yes, I received consent

**Disclosure of Interest**

None Declared

### Juvenile dermatomyositis

#### P256 Positive effect of additional upfront ivig on the outcome of Juvenile dermatomyositis

##### J. Merlijn Van Den Berg^1^, Daniek H. Schinkel^1^, Renske G. Kamperman^2^, Dieneke Schonenberg-Meinema^1^, Mariken P. Gruppen^1^, Amara Nassar-Sheikh Rashid^1^, Giske Biesbroek^1^, Taco W. Kuijpers^1^, Joost Raaphorst^2^, Anneke J. van der Kooi^2^

###### ^1^Department of Paediatric Immunology, Rheumatology and Infectious Diseases, Emma Children’s Hospital, Amsterdam University Medical Centres (AUMC), University of Amsterdam; ^2^Department of Neurology and Clinical Neurophysiology, Amsterdam University Medical Centres (AUMC), University of Amsterdam, Amsterdam, Netherlands

####### **Correspondence:** J. Merlijn Van Den Berg

*Pediatric Rheumatology 2024*, **22(2)**: PReS24-ABS-1479

**Introduction:** Juvenile dermatomyositis (JDM) is an extremely rare and severe autoimmune disease of childhood characterized by inflammation of skin and muscles. Historically, chronic disease and bad outcomes were reported in the majority of cases, but implementation of standardized treatment guidelines based on expert-consensus have improved this significantly. Currently, most JDM patients are treated with the combination of corticosteroids and methotrexate, adding further immunomodulatory therapy such as intravenous immunoglobulin (IVIG) later when deemed necessary (1). Historical evidence for adding upfront IVIG is limited but positive(2). Based on this, at the Emma Children’s Hospital/ Amsterdam University medical Centers all JDM patients received IVIG immediately after diagnosis, next to corticosteroids and methotrexate.

**Objectives:** To describe the outcome of IVIG treatment in JDM from the literature and from a longstanding cohort of JDM patients.

**Methods:** The PubMed database was searched for eligible articles. A retrospective chart review of all JDM patients diagnosed between 2003 and 2023 and treated in Amsterdam was performed. This is a non-biased group, as all children with JDM in our region with an ethnically diverse population of 3000.000 people are referred to our center. Primary outcomes were disease course, attainment of complete remission, cumulative dose of steroids and long-term side effects.

**Results:** Seventeen eligible articles were reviewed.No RCT were found. In total, 88 JDM patients treated with IVIG therapy had been described, demonstrating favorable results in 92%, with limited side effects. Next, a retrospective chart review of 24 JDM patients treated in our center was performed. All patients received 2g/kg/4 weeks (max 60g) IVIG starting at diagnosis, normally for 6-12 months. Disease remission was reached in 92% patients after a median time of one year. A medication-free complete recovery with no apparent sequelae was achieved in 19/24 (79%) patients, 5/24 (21%) have complaints of subjective long term fatigue, and just 2/24 (8%) developed mild calcinosis.

**Conclusion:** Upfront additional IVIG therapy next to corticosteroids and weekly methotrexate in JDM patients is effective and outcomes are excellent with few adverse effects. With the lack of RCTs, further research is needed to determine the exact efficacy of this therapy.

**Patient Consent**

Yes, I received consent

**Disclosure of Interest**

None Declared

**References**

1. Bellutti Enders F, Bader-Meunier B, Baildam E, Constantin T, Dolezalova P, Feldman BM, et al. Consensus-based recommendations for the management of juvenile dermatomyositis. Ann Rheum Dis. 2017;76(2):329-40
2. Al-Mayouf SM, Laxer RM, Schneider R, Silverman ED, Feldman BM. Intravenous immunoglobulin therapy for juvenile dermatomyositis: efficacy and safety. J Rheumatol. 2000 Oct;27(10):2498-503.

### Juvenile dermatomyositis

#### P257 Urinary beta2-microglobulin as a predictive marker for clinical aggravation in progressive interstitial lung diseases complicated anti-mda5 antibody-positive idiopathic inflammatory myopathies

##### Kazuko Yamazaki, Etsushi Toyofuku, Kohei Yoshioka, Seido Ooka, Kimito Kawahata

###### Rheumatology and Allergology, St.mariannna University School of Medicine, Kawasaki, Japan

####### **Correspondence:** Kazuko Yamazaki

*Pediatric Rheumatology 2024*, **22(2)**: PReS24-ABS-1556

**Introduction:** Anti-Melanoma differentiation associated gene 5 (MDA5) autoantibody (Ab) was reported to be related to rapidly progressive (RP)- interstitial lung disease (ILD) and a poor prognosis in East-Asian patients with juvenile dermatomyositis (JDM). A standard treatment for DM/JDM complicated with progressive -ILD (anti-MDA5-ILD) has not yet been established. The treatment with a combined immunosuppressive regimen of high dose PSL, calcineurin inhibitor (CNI), and intravenous pulse administration of cyclophosphamide (IVCY) has been proposed and administered beginning in the early phase of this disease in Japan. This regimen brought a significant improvement in the mortality of anti-MDA5-ILD. Plasma exchange and JAK inhibitors are useful as an additional treatment to a combined immunosuppressive regimen in the condition of the disease exacerbation for patients with anti-MDA5-RP-ILD. Predictive markers for clinical aggravation in PM/DM-ILD have been discussed through clinical and serological evaluations, including IFNs and IFNs-related chemokines. In this study, we focus on the urinary β2-microglobulin (U-β2MG), one of the candidates for markers predicting aggravation, which reflects increased HLA class I expression induced by IFN-γ.

**Objectives:** We investigated whether U-β2MG could be a predictive marker for clinical aggravation in patients with anti-MDA5-ILD.

**Methods:** We conducted a retrospective study on pediatric and adult patients with anti-MDA5-ILD who were admitted from 2016 to August 2023. These patients were classified into three groups: death with plasma exchange (PEX), survival with PEX, and survival with no PEX (nPEX). We then analyzed their U-β2MG (urinary creatinine-corrected), anti-MDA5 antibody levels, ferritin, and KL-6.

**Results:** Of the 41 admitted patients (32 alive, nine dead), 16 measured U-β2MG prior to treatment, including 11 females, three deaths with PEX, 5 with PEX, and 8 with nPEX. All patients received triple therapy (PSL+IVCY+CNI), and 6 received Tofacitinib. The pretreatment median U-β2MG was 21,706mg/gCr, 23,957mg/gCr, and 1,065 mg/gCr in the death, PEX, and nPEX groups, respectively. The median U-β2MG was predominantly higher in the death and PEX groups than in the nPEX group (P<0.05). Although the post-treatment U-β2MG was increased in the death group, the post-treatment U-β2MG was decreased in the PEX group. Serum ferritin, KL-6, and anti-MDA5 antibody levels were not significantly different among the three groups.

**Conclusion:** U-β2MG is a useful marker for predicting aggravation in patients with anti-MDA5-ILD. A persistently elevated level of U-β2MG indicates a progressive and potentially fatal clinical course.

**Patient Consent**

Yes, I received consent

**Disclosure of Interest**

None Declared

### Juvenile dermatomyositis

#### P258 Calcinosis prevalence and treatment response in Juvenile dermatomyositis: a retrospective observational study

##### Martina Rossano, Anna Malfitano, Chiara Provini, Federica Vianello, Fabiana Di Stasio, Stefano Lanni, Francesca Minoia, Giovanni Filocamo

###### SC Pediatria Immunoreumatologia, Fondazione IRCCS Ca' Granda Ospedale Maggiore Policlinico, Milan, Italy

####### **Correspondence:** Martina Rossano

*Pediatric Rheumatology 2024*, **22(2)**: PReS24-ABS-1392

**Introduction:** Calcinosis is one of the most typical complications of Juvenile dermatomyositis (JDM), which is difficult to treat and may cause long-term morbidity. It is defined as the intracellular deposition of insoluble calcium salts in affected tissues. [1] Different types of antibodies are associated with specific phenotypes; in the literature, anti-NXP2 is reported to be more frequently associated with the development of calcinosis. [2]

**Objectives:** We aimed to investigate the prevalence of calcinosis, its association with MSA and MAA patterns, and the response to treatment in JDM patients.

**Methods:** Patients with JDM followed from 2015 to 2024 in our pediatric tertiary care center were enrolled in this retrospective observational study. Patients with no available data on MSA and MAA were excluded. Descriptive analyses were reported as median and IQR for continuous variables and absolute frequencies and percentages for categorical. The T-student test and Z-test were used to analyze the two groups.

**Results:** Twenty-four patients (75% female) were enrolled. The median age at disease presentation was 6.9 y (IQR 3.3-9.6), and the median diagnosis delay was 3.5 months (IQR 0.9–3.1 months). MSA was positive in 15 patients (62%) and MAA in 3 (12.5%). Anti-NXP2 was the most frequently reported antibody in our cohort (53%). 29.1% patients presented calcinosis during the follow-up; none had calcinosis at diagnosis. Four patients were NXP2+ (57%), 1 patient was anti-SAE+, and 1 anti-Ku+. Patients were divided into 2 groups based on its presence. The median time from diagnosis to the development of calcinosis was 4 y (IQR 2.5-3.3). There was no difference in the age at onset, the delay between onset and therapy, and the CPK levels at onset between the groups with and without calcinosis. The difference in the prevalence of NXP2+ patients between the two groups was not statistically significant. Seventy-one percent of patients in the calcinosis group had disease relapse versus 29% in the group without calcinosis; (P-value 0.04). Treatments used for patients with calcinosis included MTX and GCS and IVIg for all patients. Two patients underwent therapy with bisphosphonates. Three patients underwent anti-TNF, one of which halted progression and completely dissolved the calcinosis, one showed partial resolution (treatment ongoing), and one did not respond. One patient received Jak-inhibitor for one year, showing no further progression but no improvement in calcinosis.

**Conclusion:** Calcinosis is a significant complication in JDM, affecting 29.1% of our cohort. In our cohort patients with calcinosis exhibited a higher relapse rate. Furthermore, no significant association was found between anti-NXP2 antibodies and calcinosis. Treatment outcomes for calcinosis varied, indicating the need for further research to identify risk factors and develop standardized therapeutic approaches for this debilitating complication.

**Patient Consent**

Yes, I received consent

**Disclosure of Interest**

None Declared

**References**

1. Kul Cinar O, Papadopoulou C, Pilkington CA. Treatment of Calcinosis in Juvenile Dermatomyositis. Curr Rheumatol Rep. 2021 Feb 8;23(2):13.
2. Tansley SL, Betteridge ZE, Shaddick G et al. Juvenile Dermatomyositis Research Group. Calcinosis in juvenile dermatomyositis is influenced by both anti-NXP2 autoantibody status and age at disease onset. Rheumatology (Oxford). 2014 Dec;53(12):2204-8

### Juvenile dermatomyositis

#### P259 Sleep disturbances and fatigue in children with Juvenile dermatomyositis (JDM)

##### Senne Cuyx^1^, Helena Codes-Méndez^2^, Afroditi Barmpakou^1^, Meredyth Wilkinson^3^, Dario Cancemi^3^, Polly Livermore^1, 3^, Yvonne Glackin^1^, Muthana Al-Obaidi^1, 3^, Charalampia Papadopoulou^1, 3^ and UK Juvenile Dermatomyositis Cohort and Biomarker Study (JDCBS)

###### ^1^Paediatric Rheumatology, Great Ormond Street Hospital for Children NHS Foundation Trust, London, United Kingdom; ^2^Rheumatology, Hospital de la Santa Creu i Sant Pau, Barcelona, Spain; ^3^Infection, Immunity and Inflammation Research and Teaching, UCL Great Ormond Street Institute of Child Health, University College London, London, United Kingdom

####### **Correspondence:** Senne Cuyx

*Pediatric Rheumatology 2024*, **22(2)**: PReS24-ABS-1470

**Introduction:** Sleep disturbances and fatigue are common in juvenile dermatomyositis (JDM), with a significant impact on psychosocial wellbeing. The exact prevalence and contributing factors remain poorly understood. Patient reported outcome measures (PROMIS) are an efficient tool with preliminary evidence of validity, reliability, and responsiveness to change.

**Objectives:** To assess the prevalence of fatigue and sleep disturbances in JDM and to investigate correlations with disease parameters.

**Methods:** Patients were recruited from the Juvenile Dermatomyositis Cohort Biomarker Study & Repository (JDCBS). Sleep and fatigue PROMIS and SDQ questionnaires were completed. Clinical data and routine clinical and research blood tests were collected. A cross-sectional study examined differences in active versus inactive JDM. Data were prospectively collected at 6 and 12 months.

**Results:** A preliminary 22 patients were included in the study. All of them were studied cross-sectionally and 4 patients longitudinally.

Of the 22 patients, 17 were female (77%). Median age was 14 years (11-15 years, IQR) with a median time to recruitment since diagnosis of 6 years (4-8 years, IQR). Eight patients were Caucasian, 6 Asian, and 5 Afro-Caribbean, while ethnicity was not documented in 3 patients. At the time of recruitment, 10 (45%) patients had active and 12 (55%) had inactive disease. Four (18%) were not on any treatment, and 2 (9%) were on corticosteroids. The rest (73%) were on different immunosuppressive and biological treatments. The main types of myositis specific antibodies (MSA) identified were: 6 TIF1γ positive, 4 MDA5, 2 Mi2, 1 NXP2. No MSA were detected in 3 patients, and rare MSA in 6.

Parents reported higher fatigue scores than patients (p<0.001), while patients reported higher total difficulty scores. A significant difference was seen in parent reported scores between patients with and without active disease for fatigue score (p=0.02), total difficulty score (p=0.028), hyperactivity score (p=0.029), peer problem score (p=0.019), and prosocial score (p=0.018). In the patient reported scores, the only statistically significant difference was seen in the sleep impairment score (p=0.029). There was a strong correlation between parent reported fatigue and sleep impairment scores (r^2^=0.75, p<0.05) and in parent and patient reported fatigue scores (r^2^=0.799, p<0.0001).

**Conclusion:** PROMIS and SDQ scores show feasibility to identify fatigue and sleep problems in patients with JDM. Significant differences were shown between parent versus patient reported scores, as well as between those with and without active disease. The next part of the research will focus on further collection of clinical data and blood samples (including interferon scores), which in turn will be correlated with the reported scores. Further collaborations will explore sleep and fatigue in children with JDM in association with newly proposed pathogenesis mechanisms such as mitochondrial dysfunction.

**Patient Consent**

Not applicable (there are no patient data)

**Disclosure of Interest**

None Declared

### Juvenile dermatomyositis

#### P260 Successful treatment of an anti-mda5 antibody-positive Juvenile dermatomyositis patient with refractory interstitial lung disease using tofacitinib

##### Valentina Natoli^1, 2^, Serena Palmeri^1^, Ana Isabel Rebollo-Giménez^2^, Caterina Matucci-Cerinic^1, 2^, Roberta Caorsi^2^, Stefano Volpi^1, 2^, Riccardo Papa^2^, Alessandro Consolaro^1, 2^, Marco Gattorno^2^, Silvia Rosina^2^, Angelo Ravelli^1, 3^

###### ^1^Dipartimento di Neuroscienze, Riabilitazione, Oftalmologia, Genetica e Scienze Materno-Infantili (DiNOGMI), Università degli Studi di Genova; ^2^Paediatric Rheumatology and Autoinflammatory Diseases Unit; ^3^Direzione Scientifica, IRCCS Istituto Giannina Gaslini, Genoa, Italy

####### **Correspondence:** Valentina Natoli

*Pediatric Rheumatology 2024*, **22(2)**: PReS24-ABS-1154

**Introduction:** Juvenile dermatomyositis (JDM) is a rare systemic autoimmune disorder affecting children, mainly characterized by skin and muscle vasculopathy. Some patients also manifest life-threating complications of the disease, such as interstitial lung disease (ILD). Anti-MDA5 antibody-positive JDM represents a distinct phenotype of disease with a high risk of developing rapidly progressive or refractory ILD. A large body of evidence suggests the pivotal role of type I interferon (IFN) pathway in the pathogenesis of JDM.

**Objectives:** To report the efficacy and safety of tofacitinib, a Janus kinase (JAK) 1/3 inhibitor, in treating an anti-MDA5-positive JDM young girl with refractory ILD, highlighting the potential of JAK inhibitors as targeted therapy in these patients.

**Methods:** A retrospective review of patient’s clinical records, including laboratory and radiological findings, was performed. Laboratory evaluations focused on muscle enzymes, inflammatory markers, myositis-specific autoantibodies and type I IFN signature. Radiological assessments used chest HRCT to track ILD progression, and total body short tau inversion recovery (STIR) magnetic resonance imaging (MRI) to evaluate potential sub-clinical muscle involvement.

**Results:** A previously healthy 7-year-old female patient of Caucasian ethnicity, presenting with a 6-month history of fatigue, weight loss, skin lesions and mildly impaired muscle strength, was diagnosed with anti-MDA5 positive JDM. During the diagnostic work-up performed at disease diagnosis, JDM-associated ILD was also detected. Initial treatment with methylprednisolone pulses followed by high-dose intravenous glucocorticoids, oral cyclophosphamide and monthly intravenous immunoglobulin (IVIG) infusions led to skin and muscle disease remission (normal muscle strength and negative total body STIR MRI). Nevertheless, lung disease showed no significant signs of improvement, exhibiting both clinical and radiological deterioration over time. Supported by reports on adult-onset DM and the presence of the elevated type I-IFN signature in the patient, tofacitinib in combination with subcutaneous methotrexate and IVIG were initiated. This treatment led to a stable remission, near-complete resolution of the pulmonary involvement, and discontinuation of glucocorticoids. No adverse effects were observed during 2-year follow-up evaluations.

**Conclusion:** This case underscores the promising efficacy and safety of tofacitinib in managing anti-MDA5-positive JDM with ILD non-responsive to aggressive conventional immunosuppressive treatments. It also emphasizes the importance of early suppression of disease activity and the potential benefits of employing targeted therapies based on specific biomarkers, such as type I IFN signatures. Future research should focus on the design of international randomized controlled trials aimed to assess the efficacy and safety of JAK inhibitors in JDM, as to foster personalised treatment approaches and improve patient outcomes.

**Patient Consent**

Yes, I received consent

**Disclosure of Interest**

None Declared

### Juvenile dermatomyositis

#### P261 Effectiveness of 3-month biopsychosocial based exercise model-bety on biopsychosocial status, pain, quality of life, participation and functionality in a boy with juvenile dermatomyositis: a case study

##### Orkun Tüfekçi^1^, Sinan Buran^2^, Nur B. Karaca^1^, Erdal Sağ^3^, Yelda Bilginer^3^, Edibe Ünal^2^, Seza Özen^3^

###### ^1^Department of Basic Physiotherapy and Rehabilitation, Hacettepe University Institute of Health Sciences; ^2^Department of Heart and Respiratory Physiotherapy and Rehabilitation, Hacettepe University Faculty of Physical Therapy and Rehabilitation; ^3^Department of Pediatrics, Division of Rheumatology, Hacettepe University Faculty of Medicine, Ankara, Türkiye

####### **Correspondence:** Orkun Tüfekçi

*Pediatric Rheumatology 2024*, **22(2)**: PReS24-ABS-1518

**Introduction:** Juvenile dermatomyositis (JDM) is the most common inflammatory myopathy in children characterized by muscle weakness with disease-specific skin findings and organ involvement (1). Individuals with JDM have poorer quality of life and symptoms such as anxiety, depression, and fatigue compared to healthy individuals. However, since JDM is a rare connective tissue disease, biopsychosocial model-based exercise intervention studies conducted with these individuals are limited (2).

**Objectives:** The study aimed to examine the outcomes of a case with JDM who was followed up with the Cognitive Exercise Therapy Approach (*Bilişsel Egzersiz Terapi Yaklaşımı*-BETY) intervention, a biopsychosocial-based exercise model.

**Methods:** A 9-year-old boy with a diagnosis of JDM was included in the study. Biopsychosocial status was assessed with the Juvenile Arthritis Biopsychosocial-Questionnaire (JAB-Q), pain catastrophizing with the Pain Catastrophizing Scale (PCS), quality of life with the Juvenile Arthritis Quality of Life Questionnaire (JAQQ), participation with the Child and Adolescent Scale of Participation (CASP) and functionality with the Childhood Health Assessment Questionnaire (CHAQ); Time Up and Go Test (TUG) to assess mobility and fall risk, Stair Climb Test (SCT) and Chair Stand Test (CST) to assess lower extremity strength and dynamic balance were used as functional tests. The patient was followed up with 24 sessions of BETY which includes function-oriented core stabilization exercises, chronic pain, and mood management 2 days a week for 3 months. All evaluations were performed before and after the 3-month BETY intervention.

**Results:** After 3 months of BETY training, JAB-Q total score, pain severity, disease activity, joint status, functional status, psychosocial status, performance in school, fatigue (from 45 to 20, 55.56%; from 4 to 0, completely; from 5 to 1, 80%; from 3 to 2, 33.33%; from 8 to 4, 50%; from 12 to 7, 41.67%; from 5 to 1, 80%; from 8 to 2, 75%, respectively); PCS total score, rumination, magnification, helplessness (from 17 to 9, 47.06%; from 17 to 9, completely; from 10 to 4, 60%; from 4 to 3, 25%; from 3 to 2, 33.33%, respectively), CHAQ total score, pain, general well being (from 0.375 to 0.25, 33.33%; from 4.3 to 0, completely; from 3.9 to 1.2, 69.23%, respectively), JAQQ total score (9 to 1.3, 88.33%) decreased and improved, while CASP total score (97.5 to 98.33, 0.85%) remained similar. TUG (8.79 to 7.22, 17.86%), and SCT (6.03 to 5.58, 7.46%) decreased, while CST score (17 to 21, 23.53%) increased.

**Conclusion:** The findings in the case with JDM showed improvements in all parameters including biopsychosocial status, pain, quality of life, participation, and functionality with biopsychosocial model-based exercise-BETY. The promising findings of this study may form the basis for future studies in individuals diagnosed with JDM in which more individuals are included and supported by a control group.

**Date of birth::** novembre 0

**Patient Consent**

Yes, I received consent

**Disclosure of Interest**

None Declar

**References**

1. Lundberg IE, Tjärnlund A, Bottai M, Werth VP, Pilkington C, Visser MD, et al. 2017 European League Against Rheumatism/ American College of Rheumatology classification criteria for adult and juvenile idiopathic inflammatory myopathies and their major subgroups. Annals of the Rheumatic Diseases. 2017;76 (12):1955-64.
2. Kelly AH, Singh-Grewal D, Sumpton D, Hasset G, Manera KE, Tong A. Range and consistency of outcome measures reported in randomised trials in dermatomyositis: a systematic review. Clinical and Experimental Rheumatology. 2022;40 (2):358-65.

### Juvenile dermatomyositis

#### P262 The use of artificial intelligence software compared with recommended classical software for the quantitative assessment of patients with Juvenile dermatomyositis

##### Ana Victoria Villarreal Treviño, Fernando Garcia, Nadina Rubio Perez on behalf of Colibri, Yolanda Macias^1^ and Colibri Colaborativa de Investigación en Beneficio de la Reumatología Infantil

###### Pediatrics, Hospital Universitario , Mty, Mexico

####### **Correspondence:** Ana Victoria Villarreal Treviño

*Pediatric Rheumatology 2024*, **22(2)**: PReS24-ABS-1815

**Introduction:** Introduction: Juvenile Dermatomyositis (JDM) is the most common of the pediatric inflammatory myopathies characterized by inflammatory microvasculopathy. Clasical software of Nailfold videocapillaroscopy (NVCP) is an in-vivo, rapid, and inexpensive imaging technique that allows quantitative assessment of microcirculation and storage of images for clinical and research. The SHARE guidelines for JDM recommends realization NVCP at diagnosis.(1) The resent uses of Artificial intelligence (AI) in software for analysis of capillaries was documented for adults(2) and demonstrates could differentiate between a healthy child and a JDM patient (3). But there is a lack of information regarding the reliability and concordance between the use of the validates software recommended by Microvasculatory Study Group of EULAR (Optipix) and Artificial Intelligence software (Capillary.iO

**Objectives:** Objectives: To compare the quantitative assessment of microvasculature of patients with JDM using the software Optipix by manual measure with the IA software to asses reliability between both systems

**Methods:** Methods: The present study was a cross-sectional observational NVCP was performed by the same examiner (AVT) Using the Software Optipix using an Optilia Videocapilaroscope at 200x optical prove, 4 (A-B-C-D) images were obtained from all fingers except thumbs of both hands using a videocapillaroscope equipped with a 200x optical probe. The images were collected, coded, and stored using OptiPix software (version 1.7.16), 2015 Optilia Instruments. Same images at the same resolution were analyzed by de (AI) software. The statistical analysis were performed by SPSS 21.0, frecuecnces, percentages, mean and SD was made.

**Results:** A of 832 images were analyzed from 15 patients with JDM, 85% of patients were female and all have cutaneous activity during the assessment Mi2 was founded in 5.3%, and NXP2 8%. The qualitative assessment (Capillary Density, Apical Diameter, arterial and venous diameter) were performed by the two software, the analysis shows differences between the two softwares regarding the reliability in 96% of the images regarding the quantitative. Non statistical diference were found. Kappa 0.39 (p> 0.05).

**Conclusion:** Conclusion: NVCP is an useful tool to asses and diagnosis of the JDM patients, we didn’t found correlation between the use of IA in patients with disease activity, and better correlation with active disease using the recommended software Optipix.

**Date of birth::** décembre 0

**Patient Consent**

Yes, I received consent

**Disclosure of Interest**

None Declared

**References**

1. Bellutti Enders F, Bader-Meunier B, Baildam E, et al. Consensus-based recommendations for the management of juvenile dermatomyositis. *Ann Rheum Dis*. 2017;76(2):329-340. doi: 10.1136/annrheumdis-2016-209247
2. Garaiman A, Nooralahzadeh F, Mihai C, et al. Vision transformer assisting rheumatologists in screening for capillaroscopy changes in systemic sclerosis: an artificial intelligence model [published correction appears in Rheumatology (Oxford). 2023 Apr 11;:]. *Rheumatology (Oxford)*. 2023;62(7):2492-2500. doi: 10.1093/rheumatology/keac541
3. Kassani, P. H., Ehwerhemuepha, L., Martin-King, C., Kassab, R., Gibbs, E., Morgan, G., & Pachman, L. M. (2024). Artificial intelligence for nailfold capillaroscopy analyses - a proof of concept application in juvenile dermatomyositis. *Pediatric research*, *95*(4), 981–987. 10.1038/s41390-023-02894-7

### Juvenile dermatomyositis

#### P263 Analysis of treatment approaches in Juvenile dermatomyositis patients in the jir-cohort database

##### Amy-Jayne Hutchings^1^, Claas Hinze^1^, Francois Hofer^2^, Kenza Bouayed^3^, Aurelie Chausset^4^, Nazia Maldar^5^, Helmut Wittkowski^1^

###### ^1^Klinik für Paediatrische Rheumatologie und Immunologie, Universitaetsklinikum Muenster, Muenster, Germany; ^2^Fondation Rhumatismes-Enfants-Suisse, Lausanne, Switzerland; ^3^Pediatric Rheumatology, Hopital D’enfants Ibn Rochd Casablanca, Casablanca, Morocco; ^4^Pediatric Rheumatology, Centre-Hospitalier Universitaire de Clermont-Ferrand, Clermont-Ferrand, France; ^5^Pediatric Rheumatology, SRCC Children’s Hospital, Mumbai, India

####### **Correspondence:** Amy-Jayne Hutchings

*Pediatric Rheumatology 2024*, **22(2)**: PReS24-ABS-1122

**Introduction:** Juvenile Dermatomyositis (JDM) is extremely rare, yet the most common idiopathic inflammatory myopathy in childhood^i^, inflicting vasculopathic damage to muscle, skin and visceral organs. Treatment approaches vary widely globally, hindering standardisation and optimisation^ii^. The JIR-Cohort, currently comprising data from aproximately 13,000 rheumatic disease patients across 10 countries, serves as an international network, a research tool and an electronic health record^iii^

**Objectives:** We conducted an observational patient cohort study involving 250 JDM patients from 6 countries between January 2022 and June 2023, with retrospective data accepted from January 1990, aiming to compare clinical care, treatment approaches and disease activity, while focusing on the use of immunosuppressive agents and IgG-formulations in this cohort.

**Methods:** Disease progression and activity were assessed up to 24-months post-diagnosis, with initial response categorised based upon steroid response. Disease activity was evaluated using established assesment tools (Visual Analogue Scale (VAS), childhood myositis assessment scale (CMAS) and physician assessment). We initially examined a smaller cohort of patients (n=57), hencefore referred to as ‘test cohort’, from four pediatric rheumatology centres with comprehensive datasets.

**Results:** The average age of the test cohort was 7.2 ± 3.6 years; upon diagnosis 87.7% presented with muscle involvement, 98.3% with skin involvement. 68.4% of patients tested positive for autoantibodies, with the most common being anti-nxp2 (15.8%), anti-mda5 (10.5%), anti-mi2 (7.0%) and anti-pmscl (7.0%). Additionally 17.5% of the cohort received IgG-formulation (intravenous or subcutan) therapy.

**Conclusion:** Preliminary findings indicate demographic comparability with prior JDM cohorts^iv^. Statistical analysis of the test cohort is ongoing, with the goal of developing an algorithm that can be applied to the complete project cohort. Particularly the international nuances in steroid and IgG therapy are challenging to unravel, further underscoring the need for an international standardisation of treatment plans.

**Patient Consent**

Not applicable (there are no patient data)

**Disclosure of Interest**

None Declared

**References**

1. Swafford C *et. al.,* Semin Neurol. 2020 Jun;40(3):342-348. doi: 10.1055/s-0040-1705120. Epub 2020 Apr 6. PMID: 32252099.
2. Hinze CH *et. al.,* Pediatr Rheumatol Online J. 2018 Jun 20;16(1):38. doi: 10.1186/s12969-018-0256-7. PMID: 29925381; PMCID: PMC6011340
3. Bellutti E *et. al.,* Ann Rheum Dis. 2017 Feb;76(2):329-340. doi: 10.1136/annrheumdis-2016-209247. Epub 2016 Aug 11. PMID: 27515057; PMCID: PMC5284351.
4. https://www.jircohorte.org/jircohort, accessed on 23.04.2024
5. Lam CG *et. al.,* Ann Rheum Dis. 2011 Dec;70(12):2089-94. doi: 10.1136/ard.2011.153718. Epub 2011 Oct 6. PMID: 21978999
6. Ruperto N *et. al.,* Lancet. 2016 Feb 13;387(10019):671-678. doi: 10.1016/S0140-6736(15)01021-1. Epub 2015 Nov 30. PMID: 26645190.

### Juvenile dermatomyositis

#### P264 Single center retrospective study of juvenile idiopathic inflammatory myopathies: focus on the specifics of treatment

##### Olga Kostareva, Maria Kaleda, Irina Nikishina, Svetlana Arsenyeva, Valeria Matkava, Aliya Arefieva, Anna Shapovalenko

###### Paediatric, V.A. Nasonova Research Institute of Rheumatology, Moscow, Russian Federation

####### **Correspondence:** Irina Nikishina

*Pediatric Rheumatology 2024*, **22(2)**: PReS24-ABS-1732

**Introduction:** Juvenile idiopathic inflammatory myopathies (JIIM) is a group of rare autoimmune disease affecting children. It presented by Juvenile Dermatomyositis (JDM) and different kinds of overlap syndromes. Treatment strategies for JDM are multifaceted, aiming to suppress inflammation, alleviate symptoms and prevent disease-related complications. Biologics and targeted agents become promising alternatives as opposed to glucocorticoids (GC), especially in refractory cases.

**Objectives:** To describe our cohort of pts with JIIM, clinical and laboratory features, treatment options including Biologics and JAK-inhibitors.

**Methods:** We analyzed data of 52 pts with JIIM (clinical features and treatment options) who were observed in our clinic since 2020 to 2024 years. Routine rheumatological assessment was performed in all pts.

**Results:** Our cohort includes 52 pts (18/35% male,34/65% female), among them in 41 pts JDM was diagnosed and 11 pts with overlap syndromes were recognized. The median age at onset was 5.25 y.o. (min-7 mo; max -15 y.o.). The median disease duration before establishing diagnosis – 8 mo (min – 1 mo;max – 88 mo). 47/90% pts had myopathic syndrome, 44/85% pts - typical skin lesions, 39/75% pts suffered from arthritis. Calcinosis was observed in 14/27% pts, polyneuropathy - in 6/12% pts, the lung involvement - in 7/13% pts. Laboratory findings included positive ANA in 38/73% pts, anti-Jo-1 in 2/4% pts, anti-RNP in 2/4%, anti-Scl-70 in 2/4% pts, anti-PM in 1/2% pts, level of CK was elevated in 21/40% pts. 49/94% pts received GC. Most of pts (47/90%) received methotrexate (MTX), 7 of them combined with hydroxychloroquine, 6/12% pts - mycophenolate mofetil, IVIG - 38/73% pts. Due to refractory course of JIIM 22/52/42% were treated by Biologics and JAK-inhibitors (among them 8/11/73% pts with overlap syndromes). This group had more severe course of JIIM before therapy escalation. We noted that lung involvement was significantly higher in group with Biologics (5/22/23% versus 2/30/7%), calcinosis was detected in 8/22/36% versus 6/30/20% pts, positive ANA was fixed in 20/22/91% pts versus 18/30/60% pts. Spectrum of therapy included abatacept (11 pts – in 1-st line, 2 – in 2-nd), rituximab (5 pts – in 1-st line), tofacitinib (4 pts – in 1-st line, 2 – in 2-nd), tocilizumab (1 pt – in 2-nd line after inefficacy of abatacept), upadacitinib (1 pt – in 3-rd line after inefficacy of abatacept and tofacitinib). Under the therapy we observed improvement of muscle force, relieve of myopathic syndrome, decrease of calcinosis. We detected that patients with poor adherence to therapy had a worse prognosis. On the contrary, in 5/12% pts with good compliance and early administration of therapy including CS, MTX, IVIG the inactive status of the disease without steroids was achieved.

**Conclusion:** Despite the fact that none of Biologics ang JAK-inhibitors officially registered for JIIM (JDM including), we rather often resort to the appointment of a targeted therapy «off-label» and are quite satisfied with their good effectiveness and favorable safety profile.

**Patient Consent**

Not applicable (there are no patient data)

**Disclosure of Interest**

None Declared

### Juvenile dermatomyositis

#### P265 Local injection of infliximab into calcinosis lesions in patients with juvenile dermatomyositis (JDM): a clinical trial

##### Reza Shiari^1^, Forughsadat Emami^2^, Azade Z. Mirzaee^3^, Khosro Rahmani^1^, Vadood J. Parvaneh^1^, Niloofar Shashaani^1^

###### ^1^Pediatrics, Shahid Beheshti University of Medical Sciences, Tehran; ^2^Pediatrics, Guilan University of Medical Sciences, Rasht; ^3^Pediatrics, Pediatric Respiratory Disease Research Center, Masih daneshvari Hospital, Tehran, Iran, Islamic Republic Of

####### **Correspondence:** Reza Shiari

*Pediatric Rheumatology 2024*, **22(2)**: PReS24-ABS-1562

**Introduction:** Juvenile Dermatomyositis (JDM) is a rare autoimmune disorder that primarily affects muscles and skin. One of the severe complications associated with JDM is calcinosis, and treating this condition presents significant challenges.

**Objectives:** This study aimed to evaluate the efficacy and safety of local injection of infliximab into calcinosis lesions in patients with JDM.

**Methods:** In this clinical trial, five patients diagnosed with JDM and calcinosis lesions were enrolled. The primary treatment consisted of weekly infliximab injections for 16 weeks, targeting all four sides of each lesion. Lesion dimensions, including length and width, were documented and monitored weekly. Before the intervention, patients underwent radiographic imaging. After the final injection in week 16, a follow-up radiographic assessment was performed. Data were analyzed using the Generalized Estimating Equation (GEE) method.

**Results:** The lesions’ size significantly decreased in both length and width during each visit. On average, the lesion length reduced by 2.66%, and the width shrank by 3.32% per visit. Based on radiographic findings, the average length and width of lesions at the initial visit were 12.09 ± 5.05 mm (range: 6.00-25.50 mm) and 6.35 ± 3.00 mm (range: 2.00–16.00 mm), respectively. The average length and width at the last visit were 5.59 ± 7.05 mm (range: 0–23.00 mm) and 3.41 ± 4.05 mm (range: 0–13.00 mm), respectively. No specific side effects related to the treatment were reported.

**Conclusion:** The results suggest that the direct administration of infliximab into the calcinosis lesions of patients with JDM could be a safe and effective treatment approach.

**Trial registration identifying number:** Name of the registry: The effect of infliximab injection into calcinosis lesions on patients with juvenile dermatomyositis (JDM), Trial registration number: IRCT20210808052107N1, Registration date: 2022-07-22, URL of trial registry record: https://en.irct.ir/trial/58329.

**Date of birth::** décembre 3

**Patient Consent**

Yes, I received consent

**Disclosure of Interest**

None Declared

### Juvenile dermatomyositis

#### P266 An unusual association between Juvenile dermatomyositis and parotid involvement

##### Federica Casabona^1^, Agnese Feliciello^1^, Silvia Rosina^2^, Claudio Lavarello^1, 2^, Francesca Alessi^2^, Daiana Mariano^1^, Clara Malattia^1, 2^, Marco Gattorno^2^, Angelo Ravelli^1, 3^

###### ^1^Department of Neurosciences, Rehabilitations, Ophthalmology, Genetic and Maternal Infantile Sciences (DINOGMI), University of Genoa; ^2^Rheumatology and autoinflammatory diseases Unit; ^3^Scientific Direction, IRCCS Istituto Giannina Gaslini, Genoa, Italy

####### **Correspondence:** Federica Casabona

*Pediatric Rheumatology 2024*, **22(2)**: PReS24-ABS-1182

**Introduction:** Juvenile dermatomyositis (JDM) is the most common juvenile idiopathic inflammatory myopathy, and it mainly involves muscles and skin. Multiple other organs can be affected, but the presence of parotid involvement is not reported.

**Objectives:** To describe salivary gland involvement in four patients affected by JDM.

**Methods:** We describe the cases of four patients diagnosed with JDM and followed at our center between 2013 and 2024 who developed salivary gland involvement. Parotid involvement was defined as the presence of parotid enlargement or inhomogeneous gland structure on imaging studies (US/MRI), with or without clinical evidence of parotid pain or swelling.

**Results:** Patients were three males and one female. When JDM was diagnosed, all patients presented with both muscle and skin manifestations. Median age at JDM diagnosis was 5.5 years (range 4-6). ANA were positive in one patient, while myositis specific autoantibodies (MSA) were positive in all patients, in particular Anti-TIF1-Y-Ab in three patients and Anti-NPX2-Ab in one patient. Median age at parotid involvement detection was 10 years (range 6-13), when JDM was in remission in two patients and mildly active in the other two. At that time, all patients were receiving methotrexate, in association with oral steroids in three patients, with intravenous immunoglobulins in one patient, and with topic tacrolimus in another. In 3 out of 4 patients parotid gland alterations were discovered accidentally on a routinely whole-body MRI. Only one patient presented with episodes of recurrent parotitis with painful parotid swelling, while the other three patients remained persistently asymptomatic. US parotid scans showed: increased size, altered echo-structure, increased vascularization, lymphoid hyperplasia and intra-glandular and cervical lymph nodes enlargement. Two patients also presented US signs of submandibular involvement. In the first US evaluation, parotid involvement was unilateral in three patients and bilateral in one. During disease follow-up, parotid involvement became bilateral in two patients, whereas it remained unilateral in one. No patient complained of xerostomia or xerophthalmia. Moreover, a negative salivary flow test and Schirmer’s test were performed in three out four patients.

**Conclusion:** At our knowledge, this is the first report of salivary gland involvement in JDM. Clinical and serological data allowed us to exclude both Sjogren syndrome and overlapping mixed connective tissue disease. Histological data may be useful to better understand the pathologic mechanism responsible for this unusual association between JDM and salivary gland involvement. The collection of further cases may also help to clarify its frequency, possibly underestimated, and its prognostic value, if any.

**Date of birth::** mars 08, Y

**Patient Consent**

Yes, I received consent

**Disclosure of Interest**

None Declared

### Juvenile dermatomyositis

#### P267 First case of anti‑mda5 rapidly progressive interstitial lung disease treated with eculizumab as adjunctive therapy

##### Greta Mastrangelo^1^, Elizaveta Limenis^1^, Andrea Knight^1^, Molly Dushnicky^1^, Caroline Malcolmson^2^, Fiona Kritzinger^3^, Briseida Mema^4^, Christoph Licht^5^, Brian Feldman^1^

###### ^1^Division of Rheumatology; ^2^Division of Hematology/Oncology; ^3^Division of Respiratory Medicine; ^4^Department of Critical Care Medicine; ^5^Division of Nephrology, The Hospital for Sick Children, Toronto, Canada

####### **Correspondence:** Greta Mastrangelo

*Pediatric Rheumatology 2024*, **22(2)**: PReS24-ABS-1232

**Introduction:** Rapidly progressive interstitial lung disease ILD (RP-ILD) is a rare, life-threatening complication of Juvenile Dermatomyositis (JDM), often associated with the anti-melanoma differentiation-associated gene 5 (MDA5) antibody and resistant to conventional treatment. Despite aggressive immunosuppressive therapy, the prognosis for anti-MDA5 RP-ILD remains poor, with no established evidence-based treatment (1). Recent research suggests that enhanced immune complex formation in the lungs may contribute to the pathogenesis of anti-MDA5 RP-ILD (2). To date, eculizumab, a humanized monoclonal antibody targeting complement C5, has not been utilized in the treatment of anti-MDA5 RP-ILD.

**Objectives:** To describe the first report of the use of eculizumab in treating anti‑MDA5 RP-ILD.

**Methods:** A previously healthy 7-year-old girl presented with six weeks of fever, weight loss, urticarial rash, and hepatosplenomegaly. Bloodwork showed features of macrophage activation syndrome (MAS) including cytopenia, elevated ferritin, triglycerides, transaminases, lactate dehydrogenase, soluble IL-2 receptor, CXCL9, CD 163. Creatine kinase was transiently mildly elevated and subsequently normalized. Initial chest radiograph showed bilateral perihilar peribranchial thickening with patchy airspace opacities. She was started on high-dose Dexamethasone as per the HLH 2004 protocol (3). On day two of admission, she developed acute respiratory failure, despite improved MAS markers, requiring intubation. Chest CT revealed diffuse alveolar ground-glass densities suggesting interstitial lung disease, with mild pneumomediastinum. There were no pathognomonic features of JDM except for a vague history of generalized weakness, mildly reduced nailfold capillaries (4/mm), and erythematous areas on the right knee, medial malleoli bilaterally and I metacarpophalangeal joints bilaterally on examination. Further work-up revealed positive antinuclear and anti-Ro52 antibodies at high titers. Pulse IV methylprednisolone was initiated. Unfortunately, her respiratory status continued to deteriorate, prompting escalation to extracorporeal membrane oxygenation (ECMO) and ruxolitinib (4). The diagnosis of anti‑MDA5 RP-ILD (EULAR/ACR score 6.2, probable idiopathic inflammatory myopathies) was confirmed on day 9 through an extended antibody assay that revealed a very high titer of anti-MDA5. She was subsequently treated with IV cyclophosphamide, tacrolimus, five days of therapeutic plasma exchange, two doses of rituximab and anakinra for recurring MAS, with poor response. Therefore, considering clinical and laboratory indicators of endothelial activation, along with elevated terminal complement activity (CH50 >60 kU/L) and complement staining on lung biopsy, we opted to target the complement pathway with eculizumab with the therapeutic end point of improving ventilation through reduced lung inflammation.

**Results:** The timing of the eculizumab doses was adjusted based on the CH50 level, which decreased from >60 kU/L to <10 kU/L after the first dose. She received three doses (30 mg/kg/dose) in total. While her inflammatory markers improved, she had a resolution of laboratory signs of MAS and anti-MDA5 antibodies became negative 13 days after treatment intensification, there was no improvement in her respiratory status. No specific side effects related to the medication were reported. The patient developed signs of multi-organ failure and was deemed ineligible for lung transplantation. She died 32 days post admission. Eculizumab was initiated at an advanced stage of lung damage, possibly explaining the ultimately poor outcome, despite its potential role in controlling immune-complex-mediated lung injury.

**Conclusion:** This is the first report describing the use of eculizumab as part of the treatment of anti‑MDA5 RP-ILD. Despite the poor outcome in this case, eculizumab might be considered earlier in the course of RP-ILD, given our emerging understanding of the role of complement and some of the signs of laboratory reversal that we saw.

**Patient Consent**

Yes, I received consent

**Disclosure of Interest**

None Declared

**References**

1. Selva-O'Callaghan A, Romero-Bueno F, Trallero-Araguás E, et al. Pharmacologic Treatment of Anti-MDA5 Rapidly Progressive Interstitial Lung Disease. Curr Treatm Opt Rheumatol. 2021;7(4):319-333.
2. Zaizen Y, Okamoto M, Azuma K, et al. Enhanced immune complex formation in the lungs of patients with dermatomyositis. Respir Res. 2023 Mar 19;24(1):86.
3. Henter JI, Horne A, Aricó M, et al. HLH-2004: Diagnostic and therapeutic guidelines for hemophagocytic lymphohistiocytosis. Pediatr Blood Cancer. 2007 Feb;48(2):124-31.
4. Chan Ng PLP, Mopur A, Goh DYT, et al. Janus kinase inhibition in induction treatment of anti-MDA5 juvenile dermatomyositis-associated rapidly progressive interstitial lung disease. Int J Rheum Dis. 2022 Feb;25(2):228-231.

### Juvenile dermatomyositis

#### P268 Potential predictive factors of disease course in a monocentric cohort of Juvenile myositis

##### Ilaria Maccora ^1, 2^, Laura Gatti^1^, Edoardo Marrani^1^, Maria Vincenza Mastrolia^1, 2^, Ilaria Pagnini^1^, Gabriele Simonini^1, 2^

###### ^1^Rheumatology Unit, Meyer Children's Hospital IRCCS; ^2^Neurofarba department, University of Florence, Florence, Italy

####### **Correspondence:** Ilaria Maccora

*Pediatric Rheumatology 2024*, **22(2)**: PReS24-ABS-1485

**Introduction:** Juvenile myositis (JM) is an umbrella term that includes Juvenile Dermatomyositis (JDM) and necrotising myositis (NM). The achievement of remission on treatment (RT) and medication free remission (MFR) is extremely challenging and potential predictors are quite unknown.

**Objectives:** To describe a monocentric cohort of children with JM and identify predictors of RT and MFR.

**Methods:** In a retrospective cohort study, we enrolled all patients(pts) followed in our Rheumatology Unit with JM according to Bohan and Peter criteria. Demographics, clinical, laboratory, radiological and treatment (T) data were collected. The main outcomes were the achievement of RT and MFR according to the PRINTO criteria.

**Results:** We identified 23 children (13 male 56.5%) with JM (21 JDM (91.3%), 2 NM (8.7%)) with a median follow-up of 89 months (IQR36.3-129). The median ages at first symptoms and diagnosis were respectively 8 years (IQR 4-10) and 9 (IQR 4-12). The main clinical symptoms were proximal muscle weakness in 21(91.3%), heliotropic rash in 18(78.3%), Gottron papules in 18(78.3%), dysphagia in 5(21.7%), calcinosis in 4(17.4%).

At onset, pts showed a median of CK 952 (IQR 302-1845), AST 101(IQR70-163), Aldolase 21.5 (IQR 15.5-38.5), LDH 554(IQR 392-758), CMAS 32(IQR15.25-44),MMT 80(IQR 80-80), n of muscle involved(in whole body MRI (WB-MRI) performed in 20 (87%)) of 39/42(IQR 18-40.5), and grade of inflammation of 60/82(IQR 28.75-73.5).

ANA were positive in 13(56.5%), anti-TIF1 in 1(4.3%), anti-NXP2 in 3(13%), anti-HMGCR in 2(8.6%).

The first T was administered after a median time from onset of 1 months(IQR 1-5.25). The median duration of T was 90 months(32.6-126.9), with a median number of T excluding corticosteroids of 3(IQR 2-4). All pts received corticosteroids(100%, median duration 8 months (IQR 4.5-14.5)), 17 IVIG(73.9%), 18 MTX(78.3%), 8 MMF (34.8%), 1 Cyclophosphamide(4.3%), 15 HCQ(65.2%), 9 RTX(39.1%), 1 IFX(4.3%) and 3 baricitinib(13%). MTX was considered effective in 10(43.5%), RTX in 5(21.7%), MMF in 2(8.7%), baricitinib in 2(8.7%).

At last follow-up we observed a significant improvement in CMAS (52vs32, p<0.001), MMT (80vs80, p<0.001), CK (107vs952, p0.007), n of muscle involved in WB-MRI (0/42vs39/42,p<0.001), and grade of inflammation (0/82vs60/82, p<0.001).

17 pts (73.9%) achieved RT after a median time of 18 months(IQR 6-30), while 7 MFR(30.4%). Pts who achieved MFR were younger at diagnosis (5vs10 years, p0.038), have longer follow-up(156vs46 months p0.004), longer duration of corticosteroids T(12vs6 p0.039), less frequently received RTX(χ_2_ 4.105, p 0.043). While pts who achieved RT had more frequently JDM(χ_2_9.39 p0.002), were male(χ_2_8.02 p0.005), longer follow-up(94 vs 25 months, p0.022), younger at diagnosis(6 vs 13, p0.017), longer duration of T(95vs23 months p0.039), lower number of IVIG doses (0vs7, p 0.038).

Additionally, pts with anti-NXP2 received a higher number of T (5 vs 3, p0.022) and have better chance to achieve RT with baricitinib (χ_2_ 12.24 p 0.007)

**Conclusion:** Several predictor factors of diseases course have been identified: a) children with JM have better chance of RT and MFR if younger at diagnosis, b) NM have less chance of RT than JDM, c)children with anti-NXP2 have better chance of RT with Baricitinib.

**Patient Consent**

Yes, I received consent

**Disclosure of Interest**

None Declared

### Juvenile dermatomyositis

#### P269 Early onset versus late onset Juvenile dermatomyositis: phenotype disparities and novel genetic findings

##### Ali Alasmari ^1^, Abdullah Alsonbul^1^, Sulaiman Al-Mayouf^1^, Alhanouf Alsaleem^1^

###### ^1^KFSHRC, Riyadh, Saudi Arabia

####### **Correspondence:** Ali Alasmari

*Pediatric Rheumatology 2024*, **22(2)**: PReS24-ABS-1525

**Introduction:** Juvenile dermatomyositis (JDM) is a chronic multisystem inflammatory disease of unclear origin. There are few studies described in the literature comparing the clinical features, treatment agents and outcomes of early onset JDM versus late onset JDM. To date, no published data comprising the genetic background on patients with JDM, specifically early onset group.

**Objectives:** To determine whether early onset JDM (≤5years) differ from late onset patients in terms of clinical features and disease outcome.To report the genotype findings in early onset group.

**Methods:** An observational, cross-sectional study was conducted. All patients fulfilled Bohan and peter criteria were categorized into early onset (age ≤5years) and late onset (> 5 years) JDM groups based on the age of disease onset. Data were retrospectively collected at last follow up visit. Medical records were reviewed for demographic, family history, clinical and genetic data. In addition, assessment of diseases damage measured by myositis damage index (MDI).

**Results:** Total of 15 patients with early onset JDM and 14 patients with late onset JDM with a median of age of disease onset of 4.0 months (IQR 2.0-3.0) and 10.5 months (IQR 6.0- 8.25), respectively were included. Constitutional symptoms, lipodystrophy, cutaneous ulceration, and calcinosis were more frequent in the early onset group, whereas Raynaud’s phenomenon was seen more often in the late onset JDM. Facial swelling, heliotropic rash, and gottron papules were commonly observed in both groups. There were no differences between the two groups regarding frequency of the myositis specific/associated autoantibodies. The rates of treatment with corticosteroids, IVIg and methotrexate were comparable in both groups, with an increased use of biological agents in the early onset group. Nine patients with early onset disease completed genetic testing and multiple candidate variants of uncertain significance were identified. Early onset JDM had higher mean MDI (*p*<0.05). There was no death reported in both groups.

**Conclusion:** Our study showed that early onset JDM are more likely to develop a more aggressive disease with constitutional features, lipodystrophy, and calcinosis as the most frequent manifestations. We have identified novel genetic findings with secondary candidate variants which probably plays a role regarding the phenotypic disparity.

**Patient Consent**

Yes, I received consent

**Disclosure of Interest**

None Declared

### Juvenile dermatomyositis

#### P270 Pre and post treatment changes in nail fold capillaroscopy in juvenile dermatomyositis

##### Jitendra S. Oswal, Sri Lakshmi S

###### Bharati Vidyapeeth Medical College, Pune, India

####### **Correspondence:** Jitendra S. Oswal

*Pediatric Rheumatology 2024*, **22(2)**: PReS24-ABS-1545

**Introduction:** Juvenile dermatomyositis (JDM) is a systemic inflammatory disease affecting the skin and the muscular system. The etiopathogenesis of JDM is a complex interplay of environmental triggers, immune dysfunction, and specific tissue responses.

**Objectives:** To study nailfold capillaroscopy (NFC) changes in the course of JDM.

**Methods:** We did an observational study of 9 children with JDM who attended the outpatient department for one year. The children received various immunomodulator and immunosuppressive therapy depending upon their disease severity. Their clinical features, duration of illness, treatment details and nail fold capillaroscopy changes pre and post treatment (12 to 24 weeks) were noted. The capillary density, dimensions, morphology and abnormal findings were studied. NFC changes were observed in relation to clinical and laboratory disease activity.

**Results:** The age at presentation of children with JDM was 1 to 9 years (mean age- 4). Duration between symptoms onset and diagnosis was 4 months. Among them, 7 were males and 2 females. In 5 children, the NFC changes had significant improvement in the form of decrease in number of giant capillaries, bushy capillaries and increase in capillary density. In one child who had improvement in myopathic symptoms but persistent skin changes revealed no improvement in NFC findings. In 3 children, the NFC changes (giant capillaries and bushy capillaries) worsened along with clinical flare. In one child, we noticed them ahead of clinical worsening. NFC changes were noted and found to be dynamic with giant capillaries size and CPK score correlating with Pearson correlation coefficient of 0.23 (P- 0.012). We tried eliminating the two outliers (CPK) values and the Pearson correlation coefficient was 0.83. The CMAS score and giant capillary score correlated negatively with pearson correlation coefficient of -0.02 (P-0.199).

**Conclusion:** Juvenile dermatomyositis in children has a very indolent course with many having resistant disease. NFC changes such as giant capillaries and bushy capillaries are found in JDM. Our study shows that the size of the giant capillaries correlated with clinical disease activity score and CPK values. NFC can be used as a tool monitor disease activity.

**Patient Consent**

Yes, I received consent

**Disclosure of Interest**

None Declared

### Juvenile dermatomyositis

#### P271 Intravenous immunoglobulin use in patients with Juvenile dermatomyositis

##### Selen Duygu Arik^1^, Özlem Akgün^1^, Gülşah Kavrul Kayaalp^1^, Şeyma Türkmen^2^, Mustafa Çakan^3^, Nihal Şahin^4^, Betül Sözeri^2^, Nuray Aktay Ayaz^1^

###### ^1^Division of Pediatric Rheumatology, Istanbul University Istanbul Faculty of Medicine; ^2^Division of Pediatric Rheumatology, Umraniye Training and Research Hospital; ^3^Division of Pediatric Rheumatology, Göztepe Zeynep Kamil Training and Research Hospital, Istanbul; ^4^Division of Pediatric Rheumatology, Kocaeli University Faculty of Medicine, Kocaeli, Türkiye

####### **Correspondence:** Selen Duygu Arik

*Pediatric Rheumatology 2024*, **22(2)**: PReS24-ABS-1803

**Introduction:** Juvenile dermatomyositis (JDM), the most common form of juvenile idiopathic inflammatory myositis, is an autoimmune myopathy characterized by capillary vasculopathy affecting muscle and skin tissue. The efficacy of IVIG was first demonstrated in a controlled study in adults with dermatomyositis, but, there have been no formal clinical trials of the use of intravenous immunoglobulin (IVIG) in JDM in children. However, it is generally accepted to be an effective drug in the treatment of JDM. The efficacy of IVIG in children with JDM has been demonstrated in case series resistant to treatment with prednisone, azathioprine and cyclosporine.

**Objectives:** In this multicenter study, we aimed to share our experience with IVIG in JDM.

**Methods:** 28 JDM patients receiving IVIG from 4 Pediatric Rheumatology centers were retrospectively reviewed. All patients fulfilled the Bohan-Peter classification criteria. Patients younger than 18 years of age who started intravenous immunoglobulin therapy and had at least 6 months of follow-up data were included in the study. Indications for and response to IVIG therapy, physician and patient VAS before and after treatment, disease at the start of IVIG therapy and steroid dose were assessed. Muscle strength was assessed with the Childhood Myositis Assessment Scale (CMAS, range 0-52) and Manual Muscle Testing (MMT) scale (range 0-80) if previously recorded. These scales were re-evaluated at the beginning and 6 months of IVIG treatment. Muscle enzyme levels and IVIG side effects at baseline and last visit were also questioned. Organ involvement such as lung, cardiac and gastrointestinal system (GIS) was evaluated as remission-stable disease progression.

**Results:** The study included 28 patients, 18 (64.3%) of whom were female. All patients had a history of glucocorticoid use, 8 patients were on biologic therapy for refractory disease. 5 patients were on tocilizumab, 4 on rituximab, 3 on tofacitinib and 2 on infliximab. In terms of IVIG side effects, headache was observed in 2 patients and vomiting in 1 patient. No fever, hypertension, lethargy, abdominal pain or thromboembolic events were reported in any patient. The median CMAS scores were 31.5 (IQR:14.5-43.25) at the onset of the disease, 37 (IQR:21.25-43.5) at the beginning of IVIG treatment and 50 (IQR:38-52) at the 6th month of IVIG treatment.

**Conclusion:** In conclusion, our findings suggest that IVIG is a safe alternative for the treatment of JDM. Future studies should aim to define the group of patients who may benefit from the use of IVIG in JDM.

**Date of birth::** octobre 30

**Patient Consent**

Not applicable (there are no patient data)

**Disclosure of Interest**

None Declared

### Juvenile dermatomyositis

#### P272 Juvenile Idiopathic Inflammatory Myopathies (JIIM) in a 14-year-old male patient: a case report

##### Awatif A. Abushhaiwia ^1^, Nairuz B. M. Abushhiwa^2^

###### ^1^Paediatric Rheumatology , Tripoli Children's Hospital, Faculty Of Medicine, University Of Tripoli, ^2^Pathology , Faculty Of Medicine, University Of Tripoli, Tripoli , Libya

####### **Correspondence:** Awatif A. Abushhaiwia

*Pediatric Rheumatology 2024*, **22(2)**: PReS24-ABS-1036

**Introduction:** JIIMs are rare and serious autoimmune diseases of children and young people that predominantly affect the muscles and skin but can also involve other organs.The presence of myositis-specific autoantibodies has been associated with certain clinical phenotypes, organ involvement, and disease outcomes.Here .We report an adolescent boy with severe muscle disease without skin involvement that was anti-Mi2 antibody-positive

**Objectives:** highlighting the importance of muscle biopsy and the myositis-specific autoantibody and myositis-associated autoantibody profile are the key to correct diagnosis and early treatment.

**Methods:** We describe the clinical, laboratory, imging characteristics, and treatment response of JIIM in adolescent patient.The diagnosis was confirmed by histopathology findings; parasitic infections, malignancy-associated myopathy, and congenital myopathies or dystrophies were excluded.

**Results: A 14-year-old Libyan boy**, born of non-consanguineous, healthy parents, he’s known to have bronchial asthma since, at age 4 years old, his maternal aunt has SLE.Presented to our pediatric rheumatology with anorexia and weight loss of about 6 kg.

He denied having difficulty swallowing associated with progressive loss of muscle strength and severe muscle weakness of both upper and lower extremities, but mainly in LL, that’s made him bedridden for 2 months.

On physical examination, he was unable to elevate his head, stand, or walk with a grading for motor strength of upper 3\5 and lower 2\5, decreased tone, absent deep reflexes of the upper and lower extremities, more prominent on the proximal muscle with severe muscle wasting, **s**limness, and normal texture with no thickness or skin tightness. He weighed 24 kg (under the 3rd percentile).

Other systemic examinations revealed no pathological signs.Barium studies showed hypomotility of the oesophagus;HRCT scans of the chest & PFT were also normal.ECG and echocardiography were unremarkable. CBC, ESR, and CRP were within the normal range.

Blood for ANA was positive with a high titter of 1:1280, and anticentromere B antibodies (ACA) were positive.Anti-DNA, Scl-70, and anti-RNP were negative.The myositis-specific autoantibody and myositis-associated autoantibody profile all of them were negative except anti-Mi2 antibody was weakly positive.EMG and NCS confirmed a diffuse myopathic process, despite a normal CK.Muscle biopsy revealed severe inflammatory myopathy.The diagnosis of JIIM was made. Oral prednisone 1 mg/kg\day,then 0.3mg\kg,IVIG 2 g/kg for 10 months, and MMF 500mg twice per day at 5 months stopped, then added methotraxte 15 mg/m2 wk SC.

During the 5th dose of IVIG, the patient showed marked clinical improvement and regained his ability to walk with assistance. Gaining weight (which became 34 kg).Currently he’s in complete remission

**Conclusion:** This case clarifies the importance of a muscle biopsy. The myositis-specific autoantibody and myositis-associated autoantibody profiles are not only to confirm IIMs but also helpful to identify the phenotypes of IIMs.Which was successfully treated with the combination of steroids, immunosuppressants (MTX), and IVIG.

**Patient Consent**

Yes, I received consent

**Disclosure of Interest**

None Declared

### Macrophage activation syndrome

#### P273 Investigating nlrc4 mutations in children with recurrent macrophage activation syndrome

##### Seher Sener^1^, Ezgi D. Batu^1^, Erdal Sag^2^, Ozge Basaran^1^, Yelda Bilginer^1^, Seza Ozen^1^

###### ^1^Pediatric Rheumatology, Hacettepe University; ^2^Pediatric Rheumatology, Ankara Research and Training Hospital, Ankara, Türkiye

####### **Correspondence:** Seher Sener

*Pediatric Rheumatology 2024*, **22(2)**: PReS24-ABS-1133

**Introduction:** The *NLRC4* gene mutations are associated with infantile enterocolitis and recurrent macrophage activation syndrome (MAS).

**Objectives:** We aimed to search for *NLRC4* mutations in pediatric patients with recurrent MAS.

**Methods:** Seven pediatric patients with recurrent MAS were included. All exons and 20 base-pair exon-intron boundary regions of the *NLRC4* gene were sequenced with Sanger sequencing.

**Results:** The median age at diagnosis was 2.1 (1.7-4.8) years (F/M=3/4). Three patients also had gastrointestinal system (GIS) symptoms. *NLRC4* GOF mutation was detected in only one male patient (14.3%). A heterozygous mutation in the exon 8 of *NLRC4* gene was identified in this patient *(c.2728G>A, p.Val910lle).* He had recurrent MAS episodes (six times over nine years) and GIS symptoms. Interleukin-18 level measured simultaneously was also found to be slightly high (1384 pg/mL). The patient received various treatments during the follow-up (glucocorticoid, cyclosporine A, etoposide, anakinra, and canakinumab). When the genetic analysis was performed, anakinra treatment had last been discontinued four years ago and he was under follow-up without treatment. At last visit, the patient was asymptomatic, and all laboratory values associated with MAS were within normal ranges. Consequently, a decision was made to closely monitor the patient at regular intervals without initiating any treatment.

**Conclusion:** Genetic analysis for *NLRC4* GOF mutations should be performed in patients with recurrent MAS, especially if they also have GIS symptoms. Interestingly, our disease was symptom-free, and off-treatment in the last few years. Future multicenter studies may help better define genotype-phenotype relationships and provide effective treatments in these patients.

**Date of birth::** 09.10.1989

**Patient Consent**

Yes, I received consent

**Disclosure of Interest**

None Declared

### Macrophage activation syndrome

#### P274 Clinical usefulness of serum cxcl9/stnf-rii levels for monitoring of disease activity in a patient with systemic Juvenile idiopathic arthritis associated macrophage activation syndrome receiving canakinumab

##### Mao Mizuta^1^, Shuya Kaneko^2^, Masaki Shimizu^2^, Yasuo Nakagishi^1^

###### ^1^Department of Pediatric Rheumatology, Hyogo Prefectural Kobe children's Hospital, Kobe; ^2^Department of Pediatrics and Developmental Biology, , Graduate School of Medical and Dental Sciences, Tokyo Medical and Dental University, Tokyo, Japan

####### **Correspondence:** Mao Mizuta

*Pediatric Rheumatology 2024*, **22(2)**: PReS24-ABS-1614

**Introduction:** Canakinumab (CAN), a monoclonal antibody targeting interleukin (IL)-1β, has shown remarkable therapeutic efficacy in patients with systemic juvenile idiopathic arthritis (s-JIA). However, macrophage activation syndrome (MAS) can still occur in s-JIA patients receiving CAN therapy. Furthermore, CAN can modify the clinical features and laboratory findings of MAS. Therefore, new biomarkers for predicting the development of MAS and for monitoring disease activity of MAS in s-JIA patients receiving CAN are desired.

**Objectives:** We present the case of s-JIA-associated MAS receiving CAN in whom serum levels of CXCL9 and sTNFR-II levels were useful for predicting the development of MAS and for monitoring disease activity of MAS.

**Methods:** Serum levels of IL-18, IL-6, CXCL9, and soluble TNFR-II were measured using ELISA. These results were compared with clinical and laboratory characteristics.

**Results:** This patient was diagnosed with s-JIA at 10 years of age. Methylprednisolone pulse and oral prednisolone therapy induced remission at onset. One year after onset, the first flare occurred, and tocilizumab (TCZ) was initiated. However, the flare and MAS were repeated during TCZ therapy, and CAN has been started 2.5 years after onset. After the introduction of CAN therapy, this patient was in remission without glucocorticoid administration. However, six months later, a sudden high-grade fever, hyperferritinemia, and a decreasing platelet count were observed, and the laboratory data fulfilled Ravelli’s MAS criteria. At the time of the diagnosis of MAS, serum CXCL9 and sTNFR-II levels were extremely elevated. Fourteen days before the onset of MAS, no fever or other clinical symptoms were observed, and serum ferritin level was 122.1 ng/mL. However, serum CXCL9 and sTNFR-II levels were already elevated as much as those in MAS phase, and serum IL-18 levels were extremely high in the clinical remission phase. Serum IL-6 levels were not elevated compared between the pre- and post-MAS development.

**Conclusion:** CAN can mask clinical features and laboratory findings in patients with s-JIA. New criteria for MAS during CAN therapy are necessary. Serum CXCL9 and sTNFR-II levels may be predictive markers for MAS development in s-JIA patients receiving CAN.

**Patient Consent**

Yes, I received consent

**Disclosure of Interest**

None Declared

### Macrophage activation syndrome

#### P275 Extracervical kikuchi-fujimoto pediatric disease complicated with hemophagocytic lymphohistiocytosis

##### Céline Lafay, Delphine Michel, Lena Andral, Raphaelle Sarton, Mahé RUIN

###### Saint Pierre University Hospital, Saint Pierre, France

####### **Correspondence:** Céline Lafay

*Pediatric Rheumatology 2024*, **22(2)**: PReS24-ABS-1058

**Introduction:** The Kikuchi-Fujimoto disease is a rare disease which occurs mainly in adult patients. However, a few cases have been reported in children. They are usually characterized by benign cervical lymphadenopathy with fever, although extracervical locations have been reported.

The pathophysiology of the disease remains unknown to this day. Its course is usually benign and self-limited. It has special histopathological features that allow the differential diagnosis with other entities, which from a clinical point of view can be very complicated.

**Objectives:** Here we present 2 case reports of Kikuchi Fujimoto pediatric diseases. The first was a 12-year-old male and the second a 17-year-old female.

**Methods:**

 1st case report

A 12-year-old caucasian male with a history of heart failure due to a genetic cathecolinergic ventricular tachycardia (variant in the gene RYR2) requiring an external defibrillator, presented with an isolated fever and a CRP of 300mg/l which was first treated as a staphylococcus aureus sepsis with antibiotics and a removal of the defibrillator.  2 weeks later, he developped a dermohypodermatitis with fasciitis of the left hand and 4 weeks later an arthritis of the left ankle. The ankle puncture showed a sterile synovial fluid and a negative ARN16S. He stayed febrile during 2 months. HIV, EBV, CMV, Hepatitis B and C and HIV markers were negative. ANA, ASMA, anti ds-DNA and complement fraction were normal. Chest X-ray, abdominal echography, heart echography, bone scan and myelogram were normal. A PET scan showed numerous hypermetabolic lymph nodes above and below the diaphragm with diffuse osteomedullary and periarticular hypermetabolism of the left ankle and right calf muscles.  An inguinal lymphnode biopsy revealed histiocytic necrotizing lymphadenitis, confirming the diagnosis of KFD.

After 2 months, he was admitted in intensive care unit because of a hypovolemic shock due to sudden vomitting and needed non-invasive ventilation. His laboratory results showed that he had a hemophagocytic lymphohistiocytosis (HLH) which lead to corticosteroids treatment (1g/1.73m2) for 5 days with a good response.

At the end of the treatment, he relapsed showing new features of HLH, he was then treated with anti-IL1. Since then he has shown no sign of any inflammation.

 2nd case report

A 17-year-old female presented with fever, deterioration in general health and hepatomegaly. She had no medical history.

Her blood test showed moderate inflammation CRP = 55mg/l, leukopenia 2210/mm3 and anemia Hb 9g/dL.

Her viral markers and antibodies were negative as well.

She had a PET TDM which showed multiple hypermetabolic lymph nodes above an below the diaphragm as well as an heterogenous splenic hypermetabolism.

An axillary lymph node biopsy revealed numerous histiocytes and macrophages with a few large immunoblastic looking cells an early necrosis, signing the diagnosis of KFD.

She had normal cardiac and abdominal ultrasounds and a TAP CT. She was first treated by IV antibiotics during a month. 3 weeks after the beginning of the fever, she showed biological signs of hemophagocytic lymphohistiocytosis, which lead to treatment by corticosteroids (1mg/kg), with a good response and early remission. She hasn’t relapsed since.

**Results:** Most studies have shown that the Kikuchi-Fujimoto disease is mainly located in the neck and the outcome is usually good, with frequent resolution of symptoms without treatment and recurrence in 3%–4% of cases. However, there are extracervical locations and the disease can lead to complications such as hemophagocytic lymphohistiocytosis.

**Conclusion:** The Kikuchi-Fujimoto disease is rare amongst children. Awareness should be raised amongst physicians and especially pediatricians. They must consider it in the differential diagnosis of fever of unknown origin in children.

**Patient Consent**

Yes, I received consent

**Disclosure of Interest**

None Declared

### Macrophage activation syndrome

#### P276 Visceral leishmaniosis, an insidious yet important cause of secondary hemophagocytic lymphohistiocytosis: a case series of five paediatric patients

##### Maria Isabella Petrone, Arianna De Matteis, Matteo Trevisan, Manuela Pardeo, Ivan Caiello, Valentina Matteo, Elena Loricchio, Giusi Prencipe, Fabrizio De Benedetti, Claudia Bracaglia

###### Division of Rheumatology, IRCCS Ospedale Pediatrico Bambino Gesù, Rome, Italy

####### **Correspondence:** Maria Isabella Petrone

*Pediatric Rheumatology 2024*, **22(2)**: PReS24-ABS-1801

**Introduction:** HLH is a severe life-threatening disorder, caused by hyperactivation of lymphocytes and macrophages, with hypercytokinemia leading to multiorgan failure. Secondary-HLH (sHLH) can be caused by infections, including Visceral Leishmaniosis (VL). Ruling out VL in sHLH is paramount, as treating the infection can induce remission without immunosuppressive treatment.

**Objectives:** To report 5 pediatric cases of VL-sHLH.

**Methods:** Plasma CXCL9 and IL-18 levels were measured by ELISA (n.v. <150 and <1000 pg/ml).

**Results:** Mean age at onset was 17 months (IQR 13;50). All were White and lived in Italy, where canine leishmaniosis is endemic.

- Pt 1:1-year old (y.o.) male presented with sHLH. Dexamethasone and cyclosporine (CYA) were started with good response. Relapse occurred with weaning of glucocorticoids (GCs). Amastigotes on bone marrow aspirate (BMA) and positive PCR on peripheral blood (PB) confirmed VL. Amphotericin B (L-AMB) was started with rapid improvement. CXCL9 and IL-18 levels were not available (N.A.).
- Pt 2: 13-month-old (m.o.) male presented with severe unresponsive HLH. Primary HLH was initially suspected. High-dose IVGCs and CYA were administered, with poor response. Positive serology and PCR on BMA for Leishmania were positive. L-AMB was started, with rapid improvement. CXCL9 and IL-18 levels were 77874 and 4517 pg/ml.
- Pt 3: 4-y.o. female presented with HLH. Positive PCR on PB and serology for Leishmania were found. Start of L-AMB resulted in rapid remission without immunosuppressive therapy. CXCL9 and IL-18 levels were 2020 and 9299 pg/ml.
- Pt 4: 19-m.o. female was admitted with HLH. BMA showed hemophagocytosis. Treatment with IL-1 inhibitor (IL-1i) was started with incomplete response. PCR for Leishmania was positive on BMA and negative on PB, with positive serology. LAM-B was started, combined to IVGCs for persistent HLH features. GCs and IL-1i were gradually withdrawn with persistent remission. CXCL9 and IL-18 were 12060 and 17128 pg/ml.
- Pt 5: 14-y.o. boy was transferred to our centre with suspicion of HLH. Serology and PCR for VL on PB were positive. LAM-B was started with prompt normalization of WBC, and no need for immunosuppressive therapy. CXCL9 and IL-18 were 1581 and 10543 pg/ml.

All patients met the HLH-2004 diagnostic criteria at onset. All had hyperferritinemia (mean value 3737 mg/l, IQR 1671;3737). In 4 patients CXCL9, an IFNγ-induced chemokine known to be elevated in sHLH, was markedly elevated during active disease, and decreased during remission (respectively N.A., 16665, <300, 507, N.A.). IL-18 was increased during active disease and normalized during remission. None had symptoms suggestive for Still’s disease.

**Conclusion:** VL-sHLH is common, not only in endemic regions and should always be excluded in cases of sHLH. CXCL9 confirms to be a valuable biomarker for disease activity in HLH even in this form of sHLH, and IL-18 is proven to be more specific for MAS than other causes of sHLH.

**Patient Consent**

Yes, I received consent

**Disclosure of Interest**

None Declared

### Macrophage activation syndrome

#### P277 Macrophage activation syndrome as a threatening complication of rheumatic diseases - a retrospective study from a tertiary centre

##### Ana Sofia Figueiredo^1^, Sara Araújo^2^, Inês P. Ferreira^3^, Inês Santos^4^, Sara Ganhão^5^, Francisca Aguiar^6, 7^, Mariana Rodrigues^6, 7, 8^, Iva Brito^6, 7^

###### ^1^Pediatrics, ULS Trás-os-Montes e Alto Douro, Vila Real; ^2^Pediatrics, ULS Entre Douro e Vouga, Santa Maria da Feira; ^3^Pediatrics, ULS Tâmega e Sousa, Penafiel; ^4^Rheumatology Unit, ULS de Viseu Dão-Lafões, Viseu; ^5^Pediatric and Young Adult Rheumatology Unit, ULS São João; ^6^Pediatric and Young Adult Rheumatology Unit, ULS São João; ^7^Faculty of Medicine, University of Porto; ^8^Pediatrics, ULS São João, Porto, Portugal

####### **Correspondence:** Ana Sofia Figueiredo

*Pediatric Rheumatology 2024*, **22(2)**: PReS24-ABS-1462

**Introduction:** Macrophage activation syndrome (MAS) is a potentially life-threatening complication of systemic inflammatory diseases and is most commonly associated with systemic juvenile idiopathic arthritis (sJIA). Nevertheless, it can also complicate other systemic inflammatory diseases.

**Objectives:** This study aimed to characterise the clinical presentation, laboratory findings and outcomes in pediatric MAS.

**Methods:** We conducted a monocentric retrospective study that included 12 patients with MAS diagnosis, admitted between May 2011 and May 2023 into a Portuguese tertiary pediatric centre. Patients without a primary diagnosis were excluded. We collected data regarding demographic characteristics, clinical and laboratory variables, therapeutic options, adverse effects, and prognosis. Statistical analysis was performed with SPSS (*p-value*<0.05 for statistical significance).

**Results:** Of the 12 patients with MAS diagnosis, only 4 had a previous diagnosis of a rheumatic disease. It was secondary to sJIA in 8 (67%), Kawasaki disease in 3 (25%) and systemic lupus erythematosus in 1 (8%). The mean age at presentation was 5 years and 6 months, and the female:male ratio was 2:1. Fever and hyperferritinemia were present in all patients. Additionally, 75% had a rash, 58% had hepatomegaly, and 50% had splenomegaly, lymphadenopathy or cardiac involvement. The laboratory features showed a mean ferritin level of 10933ng/mL (1339-26128ng/ml), mean fibrinogen of 314mg/dL (61-720mg/dL), and mean platelet count of 187x109/uL (16-489 x109/uL). High levels of aspartate aminotransferase (median 130 U/L) and triglycerides (median 212 mg/dL) were also described. All cases of MAS fulfilled the criteria proposed by *Ravelli et al.,* and the case in the context of SLE also fulfilled *Parodi et al*. diagnostic criteria. When comparing the 2016 *Ravelli et al.* criteria with the HLH-2004 criteria, only 6/12 patients fulfilled the MAS criteria. We also calculated MS scores for the eight sJIA patients. Eight patients scored higher than -2.1, defined as the cut-off that provided the best discrimination between MAS and active sJIA. All patients were treated with high-dose IV methylprednisolone, and most also received intravenous immunoglobulin. Anakinra was used as an add-on drug in 9 patients with good clinical response. Two patients had acute toxic hepatitis as an adverse reaction to anakinra, and one of them also had a skin reaction to canakinumab. During follow-up, one patient died from presumed sepsis, and at present, more than half of the patients (n=7) are in remission without treatment.

**Conclusion:** As described in the literature, MAS occurred predominantly in the context of sJIA, correlating with disease activity. Fever and hyperferritinemia were the cardinal signs, present in all cases. MS scoring did not perform better in our study than the 2016 *Ravelli et al.* criteria. Specific treatment with anakinra effectively treated all patients (9/12) in which it was used. MAS/HLH requires early identification and management to prevent organ failure and mortality. A high index of suspicion is required in all levels of care, especially in the absence of a previous diagnosis. Widely applicable diagnostic criteria would help identify patients who require urgent transfer to expert centres.

**Patient Consent**

Yes, I received consent

**Disclosure of Interest**

None Declared

### Macrophage activation syndrome

#### P278 Refractory parvovirus b19 triggered macrophage activation syndrome in SJIA: the crucial role of cyclosporine

##### Camilla Sembenini, Andrea Leo, Maria Laura Cagnato, Francesca Tirelli, Alessandra Meneghel, Francesco Zulian

###### Departement for Women and Child’s Health, University Hospital of Padua, Italy, Pediatric Rheumatology Unit, Padua, Italy

####### **Correspondence:** Camilla Sembenini

*Pediatric Rheumatology 2024*, **22(2)**: PReS24-ABS-1383

**Introduction:** Parvovirus B19 (PVB19) infection is a common benign disease in immunocompetent children, but severe complications may occur. Macrophage activation syndrome (MAS) is a life-threatening condition that can develop secondary to rheumatological diseases, malignancies or infections.

**Objectives:** Case report of PVB19 infection triggering MAS in a patient with systemic Juvenile Idiopathic Arthritis (sJIA).

**Results:** A healthy 12-year-old boy was admitted for acute chest pain and fever. Laboratory test revealed: Troponin I 11000 ng/L, BNP 10000 pg/mL, CRP 310 mg/L, ESR 102 mm/h, WBC 16.370/mmc. EKG and echocardiography confirmed myopericarditis with left ventricular dysfunction (EF 48%, GLS -13.8%) and pericardial effusion. Intravenous immunoglobulin (2 g/kg) was started, along with ACE-inhibitor and ibuprofen, without improvement. Due to persistent fever associated with skin rash, hepatomegaly, myalgias and pleural effusion, sJIA was suspected and i.v. Anakinra (ANK, 4 mg/kg/day) was started on day 5. However, fever persisted and a rapidly growing laterocervical lymphadenomegaly developed. Blood tests showed: WBC 1550/mmc, N 290/mmc, Hb 9 g/dL, PLT 162.000/mmc, hypofibrinogenemia 0.81 g/L, ESR 7 mm/h, CRP 118 mg/L, GOT 217 U/L, GPT 510 U/L, triglycerides 5.68 mmol/L and ferritin > 100000 ug/L. Bone marrow aspirate excluded a malignancy and showed signs of hemophagocytosis. Lymph node biopsy was consistent with necrotizing lymphadenopathy. Real-time PCR for PVB19 was positive in peripheral blood, bone marrow aspiration and lymph node biopsy. Treatment was enhanced with i.v. methylprednisolone pulses (1000 mg/day from day 10 to 12, then 2 mg/kg/day) and ANK increased up to 9 mg/kg/day from day 13. Because of persistent fever, hypofibrinogenemia and increased liver enzymes, cyclosporine (CSA, 4.5 mg/kg/day) was added on day 13. Fever and rash subsided on day 14; myalgias, lymphadenopathy, inflammatory and MAS markers gradually improved allowing steroid tapering and switching to maintenance ANK from day 24 (2 mg/Kg/day). He was discharged on day 26 with full therapy (ANK, CSA, Prednisone), presenting a favorable outcome during follow-up.

**Conclusion:** Myocarditis is a known PVB19 complication, seldom reported as a trigger for MAS. Only one case of PVB19-related MAS in sJIA successfully treated with ANK has been described in children so far. In our case high-dose ANK and steroid pulses were not enough to stop MAS progression therefore cyclosporine was needed. In conclusion, PVB19 should be considered in complicated MAS, particularly when myocarditis is present, and an old drug, like CSA, can make a difference in severe refractory forms.

**Patient Consent**

Yes, I received consent

**Disclosure of Interest**

None Declared

### Macrophage activation syndrome

#### P279 IL-1 blockers: navigating traps and solutions in macrophage activation syndrome - a singular case report

##### Michele Fastiggi, Alessandro De Fanti, Michela Cappella

###### Unit of Pediatric Rheumatology, AUSL-IRCCS di Reggio Emilia, Reggio Emilia, Italy

####### **Correspondence:** Michele Fastiggi

*Pediatric Rheumatology 2024*, **22(2)**: PReS24-ABS-1061

**Introduction:** Macrophage activation syndrome (MAS) is a rare but potentially fatal complication observed in various paediatric rheumatologic conditions, most notably in systemic juvenile idiopathic arthritis (sJIA) characterized by a cytokine storm and subsequent multiorgan dysfunction. The introduction of interleukin-1 (IL-1) blockers has revolutionized the management of the disease. However the use of these immunomodulatory therapies may obscure traditional inflammatory clinical and laboratory markers, further complicating the diagnostic process.

**Objectives:** To describe a case of sJIA-MAS on IL-1 blocker therapy with atypical inflammatory marker presentation, fully responsive to the use of high-dose intravenous Anakinra.

**Results:** A 12-year-old healthy female was diagnosed as having Still disease based on an unexplained spiking fever for 2 weeks, maculopapular-salmon rash, knee arthralgia, neutrophilic leukocytosis, elevated acute phase reactants (C-reactive protein 21.45 mg/dL), hyperferritinemia (4170 ng/mL), consistent with Yamaguchi diagnostic criteria. Treatment initiation with intravenous high-dose corticosteroids and subcutaneous anakinra resulted in rapid clinical-laboratory remission. However, few weeks post-discharge, the patient experienced recurrent continuous fever. Hematologic parameters demonstrated thrombocytopenia (Platelets 113,000/μL), hyperferritinemia (ferritin 3608 ng/ml) in absence of inflammatory markers elevation (ESR 15 mm/h and CRP 0,05 mg/dl), hypertransaminasemia (AST 161 IU/L, ALT 236 IU/L), increased lactate dehydrogenase (LDH 729 U/L), fasting hypertriglyceridemia and hypo-fibrinogenemia (fibrinogen 160 mg/dl) with elevated d-dimer (1447 ng/ml). Microbiological tests yielded negative results. Considering the clinical suspicion of macrophage activation syndrome (MAS), anakinra therapy was escalated to 8 mg/kg intravenously while maintaining steroid therapy. A progressive and complete normalization of MAS parameters was observed on day 18 of hospitalization, subsequently allowing a normal transition to the subcutaneous formulation.

**Conclusion:** MAS is a life-threatening complications of Still disease that clinically resembles multiorgan dysfunction and shock. Remarkably, our patient met MAS criteria despite normal inflammatory markers, mirroring findings in retrospective studies of MAS patients undergoing IL-6 blocker therapy^1^. Notably, escalating therapy with intravenous anakinra led to complete resolution of MAS, highlighting the potential of upstream IL-1 pathway blockade in modulating the cytokine cascade during MAS.

**Patient Consent**

Yes, I received consent

**Disclosure of Interest**

None Declared

**Reference**

1. Shimizu M et al. Tocilizumab modifies clinical and laboratory features of macrophage activation syndrome complicating systemic juvenile idiopathic arthritis. Pediatr Rheumatol Online J. 2020 Jan 10;18(1):2.

### Macrophage activation syndrome

#### P280 Large vessel vasculitis with macrophage activation syndrome as a complication of newly diagnosed childhood systemic lupus erythematosus

##### Paivi Miettunen ^1, 2^, Vijay Moorjani^1, 3^, Seamus L. Stephenson^2^

###### ^1^Pediatrics, University of Calgary; ^2^Rheumatology; ^3^Diagnostic Imaging, Alberta Children's Hospital, Calgary, Canada

####### **Correspondence:** Paivi Miettunen

*Pediatric Rheumatology 2024*, **22(2)**: PReS24-ABS-1305

**Introduction:** Aortic vasculitis is a rare but severe manifestation of childhood systemic lupus erythematosus (SLE) with up to 31.4% mortality^1^. Macrophage activation syndrome (MAS) is a severe complication of SLE with increased mortality^2^ (up to 49%). It has not been previously reported with large vessel vasculitis in childhood SLE.

**Objectives:** We report a pediatric patient who developed aortic vasculitis with SLE, followed by MAS.

**Methods:** This 15-year-old girl of East Indian background was admitted to hospital with new SLE (oral ulcers, arthritis, proteinuria, elevated anti-ds DNA (6609 (normal < 9 KIU/L), elevated chromatin, ribosomal P, Smith and Sm/RNP antibodies, low C3 and C4, and hemolytic anemia). Her neurologic manifestations included cognitive impairment, tremor, and peripheral neuropathy. She received oral prednisone (60 mg/day), Plaquenil, amitriptyline and gabapentin while waiting for a renal biopsy (eventually Class II). Her neuropathic pain worsened on day 4 of steroids and she developed tachycardia (140 bpm) and abdominal pain. Fecal occult blood was negative. ECHO revealed enlarged ascending aorta *(*z-score *+2.4)* with no pericarditis. She received IV Methylprednisolone (IVMP) pulses (1 gram/day x 3), then 50 mg/day of oral prednisone. Within 24 hours of oral prednisone, she developed high fever, malaise, new severe malar rash, worsening arthritis and neuropathic pain, vasculitis of all digits, and a hard palate ulcer. She received IVIG (2g/kg) and broad-spectrum antibiotics, further IVMP ( 1 gram/day x2), followed by oral prednisone 60 mg/day. Within 12 hours of re-starting oral prednisone, she developed fever and hypotension, requiring fluid resuscitation. Blood work was consistent with macrophage activation syndrome (MAS) ( ferritin 4562 ug/L, normal (N) 15-100 ug/L), LDH 556 U/L (N 120-250 U/L), ALT 108 U/L (N < 40 U/L), D-Dimer > 10 mg/L FEU (N < 0.5 mg/L FEU), fibrinogen 1.6g/L (N 2.0-4.0 g/L), Hgb 104 g/L (N 120-160 g/L, Plt 149 10^9^g /L (N 140-400 10^9^g /L), WBC 9.2 10^9^g /L (N 4.5-13.0 10^9^g /L ). Infectious workup was negative. She was restarted on IVMP (250 mg/day), IVIG (2g/kg), followed by IV cyclophosphamide (490 mg/dose). QID anakinra (100 mg/dose) and cyclosporin ( 2 mg/kg/day) were added for MAS. She developed severe shoulder and knee pain, suspicious for avasculart necrosis of the bone, and her IVMP dose was held at 250 mg/day. She had progressive chest pain, tachycardia, severe neuropathic pain and worsening MAS with fever. ECG, a repeat ECHO, troponin, NT-proBNP, a factor 8 related antigen, brain MRI and MR-angiogram (MRA) were ordered.

**Results:**

ECG revealed elevated T-waves. Enlargening ascending aorta ( *Z-score + 2.73*) and a pericardial effusion were present on ECHO. Troponin and NT-proBNP were normal.

Factor 8 related antigen: Elevated at 5.13 U/mL (N 0.5-1.50 U/mL). MRI Brain: unremarkable.

**MRA: Dilated ascending aorta (z-score + 2.73)**. Increased T2 signal and wall enhancement of distal common carotid and proximal extracranial ICA segments bilaterally. Enhancement of nondilated infrarenal abdominal aorta, consistent with large vessel vasculitis.

Because of ongoing severe SLE and non-responsive MAS, Rituximab 500 mg x 2 (2 weeks apart) was added. Her MAS resolved within 2.5 weeks (ferritin 59 ug/L, ALT 34 U/L, AST 24 U/L, D-dimer 0.43 mg/L, fibrinogen 2.3 g/L, Hgb 124 g/L, Plt 360 10^9^g/L, LDH 252 U/L); and anakinra and cyclosporine were discontinued. She received 2nd monthly IV cyclophosphamide (490 mg/dose) and tapering oral prednisone along with Plaquenil and prophylactic Septra. Within 4 weeks, her chest pain and all SLE manifestations, apart from peripheral neuropathic pain, resolved, Anti-ds DNA decreased to 83 KIU/L; C3, C4 and urinalysis normalized. Factor 8 related antigen was measured serially as a marker of vasculitis. It remained elevated at 6 weeks (3.27 U/mL). Her shoulder and knee pain resolved. She remains on Plaquenil, tapering oral prednisone, once monthly IV cyclophosphamide and gabapentin and amitriptyline for the neuropathic pain which is gradually improving at 9 weeks of followup.

**Conclusion:** This pediatric patient developed rare aortic ( large vessel) vasculitis with her acute SLE. This case highlights the importance of early recognition and treatment of co-existing severe SLE and MAS. Because of strong immunosuppressive treatment, prophylactic Septra and intensive surveillance for infection is necessary during treatment for SLE and MAS.

**Patient Consent**

Yes, I received consent

**Disclosure of Interest**

None Declared

**References**

1. E.M.D. Smith, H Lythgoe and CM Hedrich. *Vasculitis in Juvenile-Onset Systemic Lupus Erythematosus.* Frontiers in Pediatrics, May 9, 2019 (7): 1-9.
2. S.H. Nam, S.M. Ahn, J.S. Oh et al.*Macrophage activation syndrome in rheumatic disease: Clinical Characteristics and prognosis of 20 adult patients.* PLoS Online. 2022;17(5): e026771

### Miscellaneous rheumatic diseases

#### P281 Migratory arthritis in children: what besides rheumatic fever?

##### Bar Goldberg, Gil Amarilio, Oded Scheuerman, Tarek Zuabi, Alina Guz, Maayan Ravia, Naiera Assalia, Yoel Levinsky

###### Schneider children medical center of Israel, Petah Tikva, Tel Aviv district, Israel

####### **Correspondence:** Yoel Levinsky

*Pediatric Rheumatology 2024*, **22(2)**: PReS24-ABS-1098

**Introduction:** Migratory Arthritis, a not uncommon presentation in pediatrics, is classically associated with rheumatic fever (RF), although can be the first presentation of other medical conditions as well. The literature focusing on this phenomenon is sparse.

**Objectives:** Our aim was to review the cases presented at our institution, describe the range of conditions associated with it, and propose a workup plan for migratory arthritis in children.

**Methods:** A review of all cases of migratory arthritis in children (aged 0-18 years) presented between 2003-2023 within the emergency department and the pediatric rheumatology clinic. We extracted primary diagnoses along with epidemiological, clinical, and laboratory parameters. Next, we compared cases related to RF with other cases

**Results:** A total of 165 cases of migratory arthritis were identified. Among them 77 (41%) were diagnosed with RF, while the others had various conditions, including post-streptococcal reactive arthritis, post-viral arthritis, reactive arthritis, inflammatory bowel disease, systemic lupus erythematosus, and, rarely, malignancy. High population density areas were a significant predictors for RF (P = 0.001), as well as a family history of RF (P = 0.03) and fever (P = 0.002). Conversely, rash was more common in the non-RF group, as well as arthralgia compared to arthritis.

**Conclusion:** Migratory arthritis is not rare with a mean presentation of at least one case per month. Moreover, only 41% of cases of migratory arthritis were associated with rheumatic fever. A significant proportion of diagnoses belong to post-infectious conditions. Clinically important, malignancies are include in the differential diagnosis. There were only few cases of JIA. Migratory arthritis in children poses a diagnostic challenge, and this study proposes a diagnostic algorithm based on the collected data.

**Patient Consent**

Not applicable (there are no patient data)

**Disclosure of Interest**

None Declared

### Miscellaneous rheumatic diseases

#### P282 Large cohort of patients with rare genetic disease with rheumatological manifestations

##### Valeria Matkava^1^, Irina Nikishina^1^, Svetlana Arsenyeva^1^, Aliya Arefieva^1^, Svetlana Salugina^1^, Tatiana Markova^2^, Anna Shapovalenko^1^

###### ^1^Paediatric, V.A. Nasonova Research Institute of Rheumatology; ^2^Research Centre for Medical Genetics, Moscow, Russian Federation

####### **Correspondence:** Valeria Matkava

*Pediatric Rheumatology 2024*, **22(2)**: PReS24-ABS-1512

**Introduction:** Pediatric rheumatologists often meet patients with rare genetic syndromes associated with different rheumatological symptoms e.g. arthropathies and it’s very difficult to differentiate it is a symptom of genetic disorder or separate rheumatic disease (RD).

**Objectives:** To describe cohort of patients with rare genetic syndromes associated with inflammatory patterns of musculoskeletal abnormalities and evaluate therapy approaches.

**Methods:** We collected data of 78 patients (pts) who were examined in our clinic since 2018 to 2024. The data includes pts with rare genetic syndromes such as fibrodysplasia ossificans progressive (FOP), STING-Associated Vasculopathy with onset in Infancy (SAVI), Chronic Atypical Neutrophilic Dermatosis with Lipodystrophy and Elevated Temperature (CANDLE), Blau syndrome (BS), camptodactyly-arthropathy-coxa vara-pericarditis syndrome (CACP), Stickler syndrome (SS), Down’s syndrome (DS), Congenital insensitivity to pain (CIP), Robinow syndrome (RS) and progressive pseudorheumatoid dysplasia (PPD).

**Results:** The prospective analysis includes data of 78 pts (43/55% boys; 35/45% girls). The largest number of pts had FOP – 54 pts, 8 – BS, 4 – PPD, 3 – DS, 2 – SAVI, 2 – CACP, 2 – CIP, 1 – CANDLE, 1 – SS, 1 – RS. In all patients during routine assessment, we found signs of classical RD and verified alternative/additional rheumatic diagnosis depending on clinical manifestations, imaging findings. All pts had some rheumatological signs such as arthritis – 76/97%, deformities of digital joints – 7/9%. Also imaging (US, X-Ray, MRI, CT) detected joints damage such as synovitis of large and small joints, lesions of axial structures with sacroiliitis and ankyloses, osteitis, erosions. 15 pts with FOP were fulfilled to the diagnosis of Juvenile Ankylosing Spondylitis (JAS). One pt with DS had definite systemic JIA (sJIA) and 2 pts with SAVI had sJIA like alternative diagnosis. Other pts had the RF-negative polyarticular subtype of JIA. All pts received antirheumatic therapy including biologics and target therapy in some pts. 78/100% pts received NSAIDS, 13/16% - methotrexate, 34/43% - oral glucocorticoids (GC), 18/23% - intravenous GC, 5/7% - immunoglobuline, 11/14% - bisphosphonates, 1/1% - cyclophosphan. Due to severe manifestations of joints disorder and progression of functional impairment we decided to intensify the therapy. Most pts received tofacitinib – 44/78 (56%). Biologic therapy: 8/10% - etanercept, 6/8% - adalimumab, 2/3% - golimumab, 2/3% - infliximab, 3/4% - abatacept, 1/1% - rituximab, 6/8% - tocilizumab, 1/1% - secukinumab. Most of pts had good response with decreasing of arthritis and significant improvement of joint functions and quality of life. Some pts received more than 1 line of biologics due to secondary inefficacy – 19/24%, adverse events (uveitis de novo – 1 pt, staphylococcus sepsis – 1 pt). One pt with CACP had 3 lines of biologics and now he receives combination therapy tofacitinib and abatacept. Two pts with BS had 5 lines of biologics with refractory course of the disease.

**Conclusion:** Rheumatological manifestations in rare genetic diseases are characterized by inflammatory processes associated with the clinic, imaging and response to targeted therapy. Indeed, in such cases it is difficult to draw a clear line between “non-idiopathic” and idiopathic arthritis with severe comorbidity.

**Date of birth::** juin 08, Y

**Patient Consent**

Not applicable (there are no patient data)

**Disclosure of Interest**

None Declared

### Miscellaneous rheumatic diseases

#### P283 Clinical course of overlap syndromes in children

##### Olena A. Oshlianska^1^, Kateryna A. Yats^2^

###### ^1^Department of pediatrics, pediatric infectious diseases, immunology and allergology, Shupic National Medical Academy of postgraduate education; ^2^pediatric department for older children, Institute of Pediatrics, Obstetrics and Gynecology of the National Academy of Medical Sciences of Ukraine, Kyiv, Ukraine

####### **Correspondence:** Olena A. Oshlianska

*Pediatric Rheumatology 2024*, **22(2)**: PReS24-ABS-1147

**Introduction:** Many connective tissue diseases exhibit similar signs and symptoms, posing challenges in diagnosing specific rheumatic conditions. Overlap syndrome, characterized by clinical features of at least two other conditions, presents a diagnostic conundrum. Currently, there is no evidence-based approach or definitive treatment recommendations. BMJ Best Practice (2022) suggests adopting a syndromic approach to therapy.

**Objectives:** To analyze cases of Overlap Syndrome diagnosis and explore issues in the differential diagnosis.

**Methods:** A prospective observational study was conducted at the pediatric rheumatology clinic from 2012 to 2023. All rheumatological cases were diagnosed using ACR criteria. Out of the total 3565 children with rheumatological diseases, 16 were diagnosed with overlap syndrome.

**Results:** Among those examined, girls predominated: 15 (93,75%). The average age of the patients at the time of debut was 10,8 years (range: 5 to 16 years). The diagnosis was established through 17,5 months on average (from 3 to 57 months) from the first manifestations of the disease. In 10 cases (62,5%) the initial manifestations were skin disorders (plaque-like rash over the metacarpophalangeal joints, skin tightening, discoid rash on the face, hemorrhages), and edema was noted in 3 cases (18,75 %). Arthritis was observed in 10 patients (62,5%), primarily affecting the hand joints, with half developing contractures. Sclerodactyly was observed in 1 case, and Raynaud's syndrome in 50% of cases. EMG-confirmed myositis was registered in 5 patients, while MRI changes without increased CPK and EMG changes were observed in 1 case. Lung damage was detected in 6 patients based on functional research methods and CT scans, with severity in 1 child. Two patients exhibited nephritis without CKD. Pericarditis was observed in 4 cases, and myocardial disorders in 2. Subcutaneous calcinosis occurred in 4 cases, and salivary gland lesions in 2. Hypertransaminasemia was noted in 1 child. ANA+ was detected in 15 cases, with aRNP being the most frequent (7 (43,75%)), followed by Pm-Scl – 1, myositis-associated – 1, and anticentromeric antibodies – 1. Clinically, symptoms combined with SSc were most frequent: JDM-SSc – 4 cases, SLE-SSc – 2 cases, SV-SSc – 1 case, JIA-SSc – 3 cases. Five patients met the criteria for Sharpe syndrome, and 1 showed manifestations of SV+IBD. Therapy included systemic CS in 14 patients, MTX in 8, MMF in 1, AZA in 1, hydroxychloroquine in 4, and CP in 3. Four patients required repeated replacement of DMARDs. Calcium channel blockers were used in 10 cases, ibandronates in 3, and denosumab in 1. The majority (15 patients) achieved medical remission within 6 months, though 2 had high ANA titers, and 1 case was fatal.

**Conclusion:** Overlap syndromes, among other rheumatic diseases, are characterized by delayed diagnosis due to the non-simultaneous appearance of clinical manifestations. Vigilant screening for organ involvement in atypical rheumatic disease courses is crucial. The absence of clear management recommendations for overlap syndromes results in polypharmacy and impedes timely therapy escalation in severe cases.

**Patient Consent**

Not applicable (there are no patient data)

**Disclosure of Interest**

None Declared

### Miscellaneous rheumatic diseases

#### P284 Will it be possible to do without capillaroscopy in the diagnosis of rheumatic diseases in children?

##### Natalia Yudkina, Irina Nikishina, Alexander Volkov

###### V.A.Nasonova Research Institute of Rheumatology, Moscow, Russian Federation

####### **Correspondence:** Irina Nikishina

*Pediatric Rheumatology 2024*, **22(2)**: PReS24-ABS-1770

**Introduction:** Despite the currently developed classification criteria for rheumatic diseases (RMD), the diagnosis of many of them is often difficult. This is especially true among children. Therefore, scientists are introducing new methods for diagnosing RMD. One of these tools is nailfold videocapillaroscopy (NVC) - an accessible, fast and simple way to detect microcirculation pathology in patients of different age groups.

**Objectives:** To determine the place of NVC in the diagnosis of RMD in children.

**Methods:** The study included 146 people aged 1 to 18 years. 7 with systemic sclerosis (SSc), 30 with dermatomyositis (DM), 5 with mixed connective tissue disease (MCTD), 23 with systemic lupus erythematosus (SLE), 10 with rheumatoid arthritis (RA), 25 with primary Raynaud's phenomenon (pRP) and 46 with localized scleroderma (LoSD). All patients underwent NVC using stereomicroscopy with 200x magnification and displaying images on a widescreen monitor. The doctor examined the nailfold from fingers 2 to 5 of two hands in turn. A quantitative assessment of capillary shape, density, size and the presence of hemorrhages was carried out.

**Results:** Among 25 children with pRP, 46 with LoSD and 10 with RA, microscopy of the nailfold revealed no pathological changes in shape, density, size or the presence of hemorrhages; the capillaroscopic picture was regarded as normal, found in healthy individuals. In 7 patients with juvenile SSc, typical scleroderma patterns were found, which made it possible to confirm the difficult diagnosis of SSc. In a group of 30 children with DM, severe microangiopathy was visualized, even without microscopy. In the overwhelming majority of patients with DM, changes in the NVC were mixed vascular disorders (abnormally shaped, giant capillaries, the formation of avascular areas, hemorrhages). In NVC, non-specific changes were found in 16 patients with SLE and 3 patients with MCTD, and in 7 and 2 patients, respectively, an unchanged pattern of NVC, which also occurs in healthy people.

**Conclusion:** NVC can be widely used in the search for RMD in patients under 18 years of age due to its convenience, non-invasiveness, painlessness, patient trust in the procedure, and low cost of the study. However, the informativeness and value of the method for differential diagnosis among RMDs remains a subject of debate. In our study, for pRP, LoSD and RA, capillaroscopy turned out to be normal and did not determine the diagnosis. Exactly the same as in groups with SLE, DM, MCTD, where the type was defined as similar to SSc or non-specific abnormalities, and in some even as normal. Only in the group of children with SSc did changes in the microvasculature correspond to scleroderma patterns, which explains the inclusion of NVC in the 2013 ACR/EULAR criteria.

**Patient Consent**

Not applicable (there are no patient data)

**Disclosure of Interest**

None Declared

**Reference**

1. Melsens K, Cutolo M, Schonenberg-Meinema D, Foeldvari I, Leone MC, Mostmans Y, Badot V, Cimaz R, Dehoorne J, Deschepper E, Frech T, Hernandez-Zapata J, Ingegnoli F, Khan A, Krasowska D, Lehmann H, Makol A, Mesa-Navas MA, Michalska-Jakubus M, Müller-Ladner U, Nuño-Nuño L, Overbury R, Pizzorni C, Radic M, Ramadoss D, Ravelli A, Rosina S, Udaondo C, van den Berg MJ, Herrick AL, Sulli A, Smith V; EULAR Study Group on Microcirculation in Rheumatic Diseases. Standardized nailfold capillaroscopy in children with rheumatic diseases: a worldwide study. Rheumatology (Oxford). 2023 Apr 3;62(4):1605-1615. doi: 10.1093/rheumatology/keac487. Erratum in: Rheumatology (Oxford). 2023 Mar 16;: PMID: 36005889; PMCID: PMC10070071.

### Miscellaneous rheumatic diseases

#### P285 Childhood-onset sjögren disease in current practice: an international survey study

##### Sonia Melo Gomes^1^, Edoardo Marrani^2^, Sezgin Sahin^3^, Marija Jelusic^4^, Nathanael Bourns^5^, Francesco Baldo^6^, Marijan Frkovic^4^, Geertje Elizabeth Legger^7^, Inga Tursevich^1^, Coziana Ciurtin^8^, Muthana Al-Obaidi^1, 9^ and Childhood-onset Sjögren Disease Interest Group

###### ^1^Rheumatology, Great Ormond Street Hospital, London, United Kingdom; ^2^Rheumatology, Meyer Children's Hospital, Florence, Italy; ^3^Rheumatology, Istanbul University Cerrahpasa, Istanbul, Türkiye; ^4^Rheumatology, University of Zagreb School of Medicine, Zagreb, Croatia; ^5^patient/parent representative, London, United Kingdom, ^6^Rheumatology, ASST Gaetano Pini CTO, Milan, Italy; ^7^Rheumatology, University of Groningen, Groningen, Netherlands; ^8^Centre for Adolescent Rheumatology, University College London; ^9^Rheumatology, GOS UCL Institute of Child health, London, United Kingdom

####### **Correspondence:** Sonia Melo Gomes

*Pediatric Rheumatology 2024*, **22(2)**: PReS24-ABS-1435

**Introduction:** Chilhood-onset Sjögren disease (cSjD) is a rare, possibly underdiagnosed and under-recognised autoimmune rheumatic disease. Lack of validated classification criteria and differences in clinical presentation with adult-onset SD contribute to a delayed diagnosis. The natural history and long-term outcomes are poorly understood.

**Objectives:** To investigate current views and practices regarding the diagnosis, assessment, treatment and outcomes of cSjD.

**Methods:** An online survey with 31 questions was distributed through PReS/EMERGE social media and mailing lists (Dec23-Apr24). Descriptive statistics were performed using MS forms software and excel.

**Results:** 224 practitioners of different specialties completed the survey, although 56 (25%) did not see young people with cSjD and were therefore excluded; of the remainder, 163 (73%, from 42 countries) looked after at least 1 cSjD case in the last year (152; 68%) or before. Most participants have only 1-2 young people with cSjD in their practice (80/152; 52.6%) with 7 (4.6%) following up>10; 87 participants viewed cSjD as a spectrum of adult disease (57.3%) but 69 (42.4%) considered it to be a distinct entity. Modified ACR/EULAR 2016 adult criteria (61; 37.4%) or clinical judgement (54; 33.1%) were used for diagnosis. The clinical features identified as most important for diagnosis were recurrent/persistent parotitis (154; 94.5%), dry eye/mouth (150; 92%) and constitutional symptoms (97; 59.5%). The most used diagnostic investigations were ANA (158; 96.9%), anti-Ro (157; 96.3%) and anti-La (152; 93.2%) testing, as well as Schirmer test (95; 58.3%) and salivary gland ultrasound (89; 54.6%). The systemic medications most frequently prescribed were steroids (137; 84%), hydroxychloroquine (146; 89.6%) and methotrexate (101; 62%). Persistent/recurrent parotitis was identified as a risk factor for difficult to treat disease by 29 participants (17.8%), but many were unsure (119; 73%). Clinical manifestations (42; 25.6%) and clinical expertise (73; 44.8%) were most frequently used for assessment of disease activity and damage, respectively. For assessment of response to treatment, practitioners were mainly guided by their clinical opinion (139; 85.3%). Malignancy was reported by 11 responders, including 1 death.

**Conclusion:** Most clinicians see very few young people with cSjD and there was a significant response heterogeneity in their approach to testing, diagnosis, assessment and treatment. Many children with cSjD do not fulfill ACR/EULAR 2016 criteria and the reliance on clinician opinion and expertise for disease assessment and management highlights the need for improved education to facilitate early disease recognition and better research to support evidence-based management strategies. Future work should focus on understanding of natural course of cSjD, including malignancy risk and development of validated paediatric classification criteria.

**Patient Consent**

Not applicable (there are no patient data)

**Disclosure of Interest**

None Declared

### Miscellaneous rheumatic diseases

#### P286 Biomarkers of endothelial injury in patients with raynaud phenomenon

##### Olga Gaidarji^1^, Rodica Eremciuc^1, 2^, Elena Nedealcova^1^, Ninel Revenco^1, 2^

###### ^1^Pediatrics, State University of Medicine and Pharmacy "Nicolae Testemitanu"; ^2^Rheumatology, Mother and Child Institute, Chisinau, Moldova, Republic of

####### **Correspondence:** Olga Gaidarji

*Pediatric Rheumatology 2024*, **22(2)**: PReS24-ABS-1785

**Introduction:** Raynaud’s phenomenon (RP) is a transient color change of the extremities as a result of exposure to cold and emotional stress. At the same time, RP is the clinical manifestation of peripheral microcirculation disturbances, that can precede or accompany autoimmune connective tissue diseases.Biomarkers of endothelial damage (von Willebrand factor, tissue-type plasminogen activator) show promising results from the adults’ studies, increased levels being associated with subsequent development of the overt disease even in the absence of capillaroscopic changes.

**Objectives:** To estimate the levels of the endothelial dysfunction biomarkers (von Willebrand factor, tissue-type plasminogen activator) in children with Raynaud phenomenon.

**Methods:** A prospective cohort study was conducted. 70 patients were recruited from the in-patient and outpatient Rheumatology department of the Institute of Mother and Childcare. Patients with Raynaud’s phenomenon (RP) were selected according to pre-established inclusion and exclusion criteria. All patients were assessed for vWF and tPA levels. Definitions, nomenclature related to RP, and the assignment to primary and secondary RP groups were based on the current guidelines.

**Results:** The median age of the participants was 15.2 years, with an interquartile range (IQR) between 13.5 and 16.7 years. Among the 70 patients recruited with Raynaud phenomenon, 50% were diagnosed with primary Raynaud phenomenon, while the other 50% were diagnosed with secondary Raynauds. Girls represented 71.4% of the cohort, while boys represented 28.6%. Median vWF levels were 104% [IQR: 96,117] in the primary RP group and 127% [IQR 96-208] in the secondary RP group. Median tPA levels in the primary RP group was 93 [IQR 85-97] and 96 [IQR 84-103] in the second group.

**Conclusion:** Raynaud phenomenon and its potential transition to a definite or undifferentiated CTD is a topic of great interest, given the high burden and increased severity of rheumatic conditions with onset in childhood. Early detection of patients at risk for the development of connective tissue diseases, and timely therapeutic intervention could lead to a huge improvement in the lives of patients with RMDs.

**Date of birth::** juin 20, Y

**Patient Consent**

Yes, I received consent

**Disclosure of Interest**

None Declared

**References**

1. Belch, J., A. Carlizza, P. H. Carpentier, J. Constans, F. Khan, J.-C. Wautrecht, A. Visona, C. Heiss, M. Brodeman, Z. Pécsvárady, K. Roztocil, M.-P. Colgan, D. Vasic, A. Gottsäter, B. Amann-Vesti, A. Chraim, P. Poredoš, D.-M. Olinic, J. Madaric, S. Nikol, A. L. Herrick, M. Sprynger, P. Klein-Weigel, F. Hafner, D. Staub and Z. Zeman (2017). "ESVM guidelines – the diagnosis and management of Raynaud’s phenomenon." Vasa 46(6): 413-423.
2. Gorski, S., M. Bartnicka, A. Citko, B. Żelazowska-Rutkowska, K. Jablonski and A. Gorska (2019). "Microangiopathy in Naifold Videocapillaroscopy and Its Relations to sE- Selectin, Endothelin-1, and hsCRP as Putative Endothelium Dysfunction Markers among Adolescents with Raynaud’s Phenomenon." Journal of Clinical Medicine 8(5): 567.
3. Gualtierotti, R., F. Ingegnoli, S. Griffini, E. Grovetti, M. O. Borghi, P. Bucciarelli, P. L. Meroni and M. Cugno (2017). "Detection of early endothelial damage in patients with Raynaud's phenomenon." Microvascular Research 113: 22-28.
  Herrick, A. L. and F. M. Wigley (2020). "Raynaud's phenomenon." Best Practice & Research Clinical Rheumatology 34(1): 101474.

### Miscellaneous rheumatic diseases

#### P287 Primary juvenile sjogren syndrome: case series in a tertiary center

##### Inês Madureira, M Inês Marques, Cristina Henriques, Marta Conde, Margarida P. Ramos

###### Pediatric Rheumatology Unit , Hospital Dona Estefânia, ULS São José, Lisbon, Portugal

####### **Correspondence:** M Inês Marques

*Pediatric Rheumatology 2024*, **22(2)**: PReS24-ABS-1809

**Introduction:** Primary Sjögren Syndrome (pSS) is a chronic multisystem autoimmune disorder characterized by inflammation of the exocrine glands. Childhood-onset disease is rare and its most common feature is recurrent parotid swelling/parotitis, while extraglandular manifestations are less common.

In 1999, Bartunkova proposed a set of criteria for the diagnosis of pSS in children, such as exists for adults, but these have yet to be validated.

**Objectives:** Characterization of demographics, clinical and complementary findings, treatment and outcomes in children with pSS, followed in a tertiary reference center for pediatric rheumatic diseases.

**Methods:** A single center retrospective analysis of children diagnosed with childhood-onset pSS using the classification criteria proposed by Bartunkova from 2000 to April 2024.

**Results:** Nine patients were identified (7 female), median age at diagnosis of 14 years (9-16y) and median time to diagnosis of 4.2y (0.6-5.9y).

The most common clinical manifestations at diagnosis were recurrent parotitis (6/9), sicca symptoms (6/9: 3 with dry mouth and 3 with dry eye - one with keratoconjunctivitis sicca). During follow-up, three additional patients developed xeropthalmia (3/6 with a diminished Shirmer test), and two with xerostomia (2/9 with dental cavities and 3/9 with recurrent oral ulcers).

Other manifestations present at diagnosis include constitutional symptoms (4/9), arthralgias/arthritis (4/9), and Raynaud Phenomenon (2/9). All patients had evaluation of their Diffusing Capacity for Carbon Monoxide, 2/9 showed reduced capacity and one also had an abnormal thoracic CT with ground-glass opacities. One patient had a pericardial effusion and another had a Central Nervous System venous thrombosis.

All patients showed a positive ANA (³1:160); 8/9 had a positive anti-SSA/Ro antibody, 5/9 anti-SSB/La, 6/9 positive RF, and none had positive cryoglobulins. All patients underwent salivary gland ultrasound, which was abnormal in 8/9, and minor salivary gland biopsy, which revealed lymphoepithelial sialadenitis.

Symptomatic treatment included the use of non-steroidal anti-inflammatories and topical artificial tears. Further pSS treatment included hydroxychloroquine (8/9), azathioprine (2/9), methotrexate (1/9) and oral steroids (2/9). During follow-up, the patients showed optimum symptomatic control and no occurrence of complications to date.

**Conclusion:** Childhood-onset pSS manifestations can be under-recognized which might account for the delay to diagnosis. Additionally, the absence of current validated diagnostic criteria render this a challenge. A high index of suspicion of pSS should be applied to children with recurrent parotitis, even in the absence of sicca symptoms, given that the latter are less common in pediatric age. In our case series, extraglandular involvement was frequent and, in most cases, present at diagnosis. Salivary gland ultrasoundwas a reliable diagnostic tool for sialadenitis, is readily available and non-invasive.

**Patient Consent**

Not applicable (there are no patient data)

**Disclosure of Interest**

None Declared

### Miscellaneous rheumatic diseases

#### P288 Lubricin deficiency: a single center experience

##### Serena Palmeri^1, 2^, Atena Stoian ^1, 2^, Marco Burrone^1, 2^, Claudio Lavarello^1, 2^, Caterina Matucci-Cerinic^1, 2^, Valentina Natoli^1, 2^, Stefano Volpi^1, 2^, Roberta Caorsi^1, 2^, Silvia Rosina^1^, Alessandro Consolaro^1, 2^, Clara Malattia^1, 2^, Marco Gattorno^1^, Riccardo Papa^1^

###### ^1^IRCCS Istituto Giannina Gaslini, Paediatric Rheumatology and Autoinflammatory Diseases Unit; ^2^Università degli Studi di Genova, Dipartimento di Neuroscienze, Riabilitazione, Oftalmologia, Genetica e Scienze Materno-Infantili (DiNOGMI), Genova, Italy

####### **Correspondence:** Atena Stoian

*Pediatric Rheumatology 2024*, **22(2)**: PReS24-ABS-1384

**Introduction:** Lubricin deficiency (LD), also known as camptodactyly, arthropathy, coxa vara, pericarditis (CACP) syndrome, is a rare monogenic disorder caused by homozygous mutations of *PRG4*. Patients usually present with early-onset, non-inflammatory polyarthropathy, causing progressive and symmetric joint contractures, skeletal abnormalities, and non-inflammatory pericardial and pleural effusions. The incomplete penetrance may cause misdiagnosis with idiopathic inflammatory polyarthritis especially in childhood.

**Objectives:** To describe a cohort of LD patients followed in our Center.

**Methods:** We conducted a retrospective analysis of medical records of LD patients, comparing clinical manifestations, lab tests, imaging, and genetic analysis. Management strategies, including non-pharmacological interventions, were assessed.

**Results:** We followed-up 6 LD patients from 5 families, with a median age of 19 years (range 12- 31). Only one patient had consanguineous parents. The vast majority were male (5/6 patients, 83%). The median age at the disease onset was 7.6 months (range 0 – 63.6) and the median diagnostic delay was 6 years (range 0 - 25). Symptoms prompting genetic analysis were symmetric camptodactyly requiring surgery (4/6 patients, 66%), arthropathy (6/6 patients, 100%), coxa-vara (2/6 patients, 33%) and pericarditis (2/6 patients, 33%). During the follow-up, the most affected joints were interphalangeal joints of the hands (6/6 patients, 100%), knees (6/6, 100%), elbows (4/6, 67%) and wrists (4/6, 67%). In all the episodes of joint swelling, musculoskeletal ultrasound showed joint effusion without Doppler signals or a significant synovial thickening. Inflammatory markers were always within the normal range and the common serum autoantibodies were absent. In 5/6 patients (83%) arthrocentesis was carried out with the analysis of synovial fluid showing amorphous material with a dense gelatinous liquid. A patient was treated with methotrexate for 2 years, etanercept for 1 year, adalimumab for 1 year and tocilizumab for 6 months prior to diagnosis, without benefit. One patient underwent a synovial biopsy. Only two patients exhibited coxa vara throughout the entire follow-up period.

Two patients displayed pericarditis, complicated by restrictive cardiomyopathy in one case and with a recurrent course in a patient. Interestingly, cardiac valve involvement was present in 3/6 patients (50%). Specifically, one patient exhibited moderate tricuspid valve insufficiency, while a patient showed mild aortic and mitral valve insufficiency, and the other patient displayed mild pulmonary valve insufficiency.

**Conclusion:** Despite the camptodactyly is the most frequent cause of clinical suspicion in our cohort, we show that a recurrent, drug-resistant joint involvement causing fix contractures without bone erosions or imaging signs, such as synovial hypertrophy with Doppler signal, should prompt a larger differential diagnosis, including genetic analysis of *PRG4*. Furthermore, our experience suggests a cardiac valvulopathy in LD to be confirmed in larger cohort.

**Patient Consent**

Not applicable (there are no patient data)

**Disclosure of Interest**

S. Palmeri: None Declared, A. Stoian: None Declared, M. Burrone: None Declared, C. Lavarello: None Declared, C. Matucci-Cerinic: None Declared, V. Natoli: None Declared, S. Volpi Speaker Bureau with: Sobi, R. Caorsi Speaker Bureau with: Novartis, Sobi, S. Rosina: None Declared, A. Consolaro: None Declared, C. Malattia: None Declared, M. Gattorno Speaker Bureau with: Novartis, Sobi, Fresenius Kabi, Kiniksa, R. Papa: None Declared

### Miscellaneous rheumatic diseases

#### P289 Extra-intestinal manifestations in pediatric inflammatory bowel diseases

##### Kenza Bouayed^1, 2^, Asmaa Boullouz^1^, Ghita Benbrahim Ansari^1, 2^, Hanan Aboufaris^1^, Asmaa Sakhi^1, 2^, Abdelhak Abkari^2, 3^

###### ^1^Pediatric Rheumatology, Hôpital Mère Enfant A. Harouchi, CHU Ibn Rochd; ^2^Faculty of Medicine and Pharmacy, Hassan 2 University; ^3^Peditric Gastro-Enterology, Hôpital Mère Enfant A. Harouchi, CHU Ibn Rochd, Casablanca, Morocco

####### **Correspondence:** Kenza Bouayed

*Pediatric Rheumatology 2024*, **22(2)**: PReS24-ABS-1646

**Introduction:** Inflammatory bowel disease (IBD) is a multisystem disorder whose main manifestation is the digestive tract. Extra-digestive symptoms mainly concern joints, skin and eyes. They can be revelatory, occur at the same time as digestive symptoms, or during the course.

**Objectives:** Explore the extra-digestive manifestations of IBD in children, focusing on their clinical diversity, and therapeutic management.

**Methods:** This retrospective study involved 15 children followed up for extra-intestinal manifestations of IBD, including 14 Crohn's disease (CD) and one ulcerative colitis (UC), over an 11-year period from January 2011 to December 2023.

**Results:** The mean age at diagnosis was 10.27 years. The mean age of onset was 9.6 years, with a sex ratio of 4. Constitutional manifestations were: fever 26.66%, weight loss 33.33%. Digestive manifestations were present in all patients, with abdominal pain 86.66%, chronic diarrhea 86.66%, vomiting 26.66%, digestive hemorrhage 33.33% and one case of intestinal occlusion with ascites. Joint involvement was reported in 80%, with arthralgia 3 cases and arthritis localized mainly in the ankles 9 cases, while 6 patients with CD had both axial and peripheral involvement. Mucocutaneous manifestations were present in 26.66% (erythema nodosum 4 cases, oral aphthosis 3 cases and bipolar aphthosis one case), while bilateral panuveitis was present in one UC case. They were inaugural 66.6% and concomitant with digestive manifestations 33.3%. Inflammatory syndrome was present 86,6%. Auto-immune tests revealed a positive anti-Saccharomyces cerevisiae antibody in 2 of 10 cases. Fecal calprotectin was increased 93.33%. Regarding malabsorption, hypoalbuminemia, hypocholesterolemia and hypoferritinemia were found respectively in 20, 33.33 and 46.66%. Colonoscopy confirmed the diagnosis of IBD in all patients. Treatment was based on corticosteroid and azathioprine in all CD patients, and corticosteroid combined with 5-aminosalicylic acid in the UC patient. Five patients required treatment with anti-TNF monoclonal antibodies (Adalimumab 2, Infliximab 3). The indication was refractory joint involvement 2 cases, severe digestive involvement one case and both refractory joint and digestive involvement 2 cases. The course was marked by complete remission on treatment 6 cases, relapses 7 cases and still active disease 2 cases.

**Conclusion:** Extra-intestinal manifestations of IBD are polymorphous and more frequent in children. They may precede digestive involvement and delay diagnosis. They may occur during the course, raising the question of whether their origin is related to the disease or to the paradoxical effect of anti-TNF agents. They are dominated by joint involvement. They have a poor prognosis and may require aggressive treatment.

**Patient Consent**

Not applicable (there are no patient data)

**Disclosure of Interest**

None Declared

**Reference**

1. Rahmani P. Extraintestinal Manifestations of inflammatory bowel disease and associated factors in pediatric patients. Ann Med Surg (Lond). 2022 Feb 15;75:103363

### Miscellaneous rheumatic diseases

#### P290 Evaluation of heart rate variability in childhood diagnosed with raynaud's phenomenon

##### Samet Ulusoy^1^, Eviç Z. Başar Akgün^2^, Yunus E. Bayrak^3^, Betül Öksel ^3^, Nihal Şahin^3^, Hafize E. Sönmez^3^

###### ^1^Department of Pediatrics, ^2^Kocaeli University, Faculty of Medicine, Department of Pediatric Cardiology, ^3^Department of Pediatric Rheumatology, Kocaeli University, Faculty of Medicine, KOCAELİ, Türkiye

####### **Correspondence:** Betül Öksel

*Pediatric Rheumatology 2024*, **22(2)**: PReS24-ABS-1093

**Introduction:** Raynaud's phenomenon(RP) is a vasoconstrictive condition characterized by pallor, cyanosis, and erythema in the extremities triggered by cold and emotional stress.Although the etiopathogenesis of RP remains incompletely understood, it is believed to be associated with vascular hyperactivity.Patients without underlying structural vascular disease or collagen tissue disease are classified as primary RP, while cases associated with vascular or collagen tissue diseases are classified as secondary RP.Primary Raynaud's phenomenon typically begins at an earlier age and presents with symmetric involvement.Typically, these patients exhibit normal capillaroscopic findings, and their levels of anti-nuclear antibodies (ANA) are usually negative.It is believed that autonomic nervous system dysfunction is an important factor causing vascular hyperactivity in cases of RP.Heart rate variability (HRV) is one of the most reliable parameters for demonstrating autonomic dysfunction.However, the studies conducted on this topic in children are insufficient.

**Objectives:** Our study aims to investigate heart rate and heart rate variability in cases diagnosed with primary RP.

**Methods:** Patients aged 0-18 years who were followed up with a diagnosis of primary RP were included in the study.The files of patients were scanned, and the demographic characteristics, date of diagnosis, symptoms, comorbidities, treatments, clinical courses, laboratory data, and capillaroscopic findings were recorded.Rhythm analysis was conducted on all patient individuals using Holter monitors.Time-dependent heart rate analysis was performed using the 24-hour ECG recordings, resulting in obtaining average heart rate, maximum and minimum heart rates, average RR intervals, and 24-hour SDNN, SDSD, and RMSSD values.

**Results:** The study included 36 patients,of whom 11 (30.4%) were male and 25 (69.4%) were female.The median ages for symptom onset, diagnosis, and inclusion in the study were 14 years (range 5-16),15 years (range 7-17),and 15 years (range 8-18), respectively.Three patients (8.3%) had a family history of RP, and the consanguinity rate was 13.9% (n=5).Thirteen patients (36.1%) showed biphasic symptoms,11 (30.6%) had triphasic symptoms, and 12 (33.3%) had monophasic findings.Bruising was observed in 33 patients (91.7%), pallor in 22 patients (61.1%), and redness in 21 patients (58.3%).Symptoms worsened during winter in 31 patients (86.1%), were present throughout all seasons in 5 patients (13.9%), and stress exacerbated symptoms in 16 patients (44.4%).It was found that 6 patients (16.6%) tested positive for ANA. Nevertheless, none of the patients fulfilled the diagnostic criteria for autoimmune diseases.Initially, all patients were advised on preventive measures.In Holter examinations, when heart rate variability was analyzed, SDNN(ms)(standard deviation of RR intervals) was significantly different in patients diagnosed with RP compared to healthy controls (132 ms *vs* 145 ms,p=0.04).However, no significant differences were observed in terms of SDSD (standard deviation of successive NN interval differences) and RMSSD (square root of the mean of the squares of successive NN interval differences) values.

**Conclusion:** Raynaud's phenomenon is a disease with an unclear cause,and it may involve a dysfunction in the autonomic nervous system favoring increased sympathetic activity and decreased parasympathetic activity in affected individuals.It is also important to examine patients from this perspective.

**Date of birth::** mars 29, Y

**Patient Consent**

Not applicable (there are no patient data)

**Disclosure of Interest**

None Declared

### Miscellaneous rheumatic diseases

#### P291 The effect of cytokine blocking agents on the long-term immunogenicity of tetanus toxoid vaccine in children with rheumatic diseases

##### Tjaša Šinkovec Savšek ^1, 2^, Mojca Zajc Avramovič^1^, Nina Emeršič^1^, Tadej Avčin^1, 2^, Nataša Toplak^1, 2^

###### ^1^Department of Allergology, Rheumatology and Clinical Immunology, University Children's Hospital, University Medical Centre Ljubljana, ^2^Faculty of Medicine, University of Ljubljana, Ljubljana, Slovenia

####### **Correspondence:** Tjaša Šinkovec Savšek

*Pediatric Rheumatology 2024*, **22(2)**: PReS24-ABS-1358

**Introduction:** Rheumatic diseases (RD) belong to a large group of immune-mediated diseases where patients are often immunocompromised – because of the disease and the treatment they receive. Therefore, after vaccination against infectious diseases, long-term immunogenicity and efficacy can be compromised. Data on long-term immunogenicity after tetanus vaccination in children with RD is limited, and the optimal time for tetanus booster vaccination in those treated with anti-cytokine therapy (ACT) is not known.

**Objectives:** The main objective was to determine long-term immunogenicity after the fifth dose of the tetanus vaccine, given according to the National Immunization Program (NIP), in adolescents with RD, and to determine if the timing of the sixth booster dose in the NIP is appropriate also for adolescents with RD treated with ACT.

**Methods:** We conducted a retrospective study and collected data from adolescents with RD at regular visits at the rheumatology outpatient clinic at the University Children's Hospital in Ljubljana, Slovenia. All patients received the fifth dose of the tetanus vaccine according to the NIP and were vaccinated at least two years before their inclusion in the study. We collected demographic data and data regarding diagnosis and therapy. The anti-tetanus toxoid IgG levels were measured using ELISA, and levels above 0.1 IE/ml were considered protective. We compared levels between two groups, those treated with ACT and those never treated with systemic medications. For statistical analysis, we used a student's t-test for two independent samples.

**Results:** We collected data for 44 adolescents with RD; 33 (75%) were female. The most prevalent diagnosis was juvenile idiopathic arthritis (93%). Of the enrolled patients, at some point during their disease, 22 (50%) were treated with ACT, and of those, 20 (91%) were receiving anti-TNFα. Twelve patients (27%) were treated with immunomodulatory therapy only, and ten (23%) never received any of these medications. At the time of data collection, all patients were vaccinated with five doses of the tetanus vaccine. We determined the IgG levels at 5.6±1.9 years after the fifth dose. On average, the IgG levels were lower in 22 patients treated with ACT compared to the 10 untreated patients, with IgG 0.64±0.51 IE/ml and 0.71±0.46 IE/ml, respectively. However, there was no statistically significant difference in IgG levels between the two groups, t(30) = -0.32 and *p*-value = 0.75.

Three (9%) patients (two treated with anti-TNFα and one with anti-IL-1) had IgG levels below the threshold and were considered unprotected. We measured their IgG levels in the period of 5.3 to 6.2 years after the fifth dose of the vaccine. After receiving these results, all three were vaccinated with the sixth dose of the vaccine ahead of the vaccination planned in the NIP. They developed adequate responses, with IgG levels of 2.22, 0.62, and 1.52 IE/ml, respectively.

**Conclusion:** Our preliminary results suggest that patients with RD treated with ACT tend to have lower anti-tetanus toxoid IgG levels compared to the untreated patients and could benefit from an earlier booster tetanus vaccination than indicated in the NIP.

**Trial registration identifying number:** The study was approved by the Medical Ethics Committee of the Republic of Slovenia (document number: 0120-532/2023-2711-6).

**Patient Consent**

Yes, I received consent

**Disclosure of Interest**

None Declared

### Miscellaneous rheumatic diseases

#### P292 Retrospective single-center study of the characteristics, management, and outcomes of childhood-onset undifferentiated connective tissue disease

##### Flavia Riccio^1^, Francesco Mori ^2^, Angela Pistorio^3^, Francesca Alessi^2^, Stefania Viola^2^, Silvia Rosina^2^, Alessandro Consolaro^2^, Marco Gattorno^2^, Clara Malattia^2, 4^

###### ^1^University of Campania Luigi Vanvitelli, Naples, ^2^Rheumatology and Autoinflammatory diseases Unit, ^3^Biostatistics Unit, IRCCS Istituto Giannina Gaslini, ^4^Department of Neurosciences, Rehabilitation, Ophthalmology, Genetic and Maternal Infantile Sciences (DINOGMI), University of Genoa, Genoa, Italy

####### **Correspondence:** Francesco Mori

*Pediatric Rheumatology 2024*, **22(2)**: PReS24-ABS-1542

**Introduction:** Undifferentiated Connective Tissue Disease (UCTD) is a clinical condition characterized by serologic and clinical manifestations of systemic autoimmune diseases, without meeting, however, the diagnostic criteria of a specific connective tissue disease (CTD). The prognosis of UCTD usually depends on the degree of organ involvement. In adults, up to 30% of patients have an evolving course, with the majority developing systemic lupus erythematosus (SLE) or rheumatoid arthritis (RA) within 5-6 years of UCTD diagnosis. To date, there are no data on the clinical and serologic phenotype, disease course, and outcomes of pediatric-onset UCTD.

**Objectives:** To describe the first cohort of pediatric patients with UCTD and to identify predictive factors for progression to CTD.

**Methods:** This is a retrospective cohort study of patients with pediatric UCTD seen at the study center over the past 10 years. Patients were categorized as evolving (eUCTD) or stable UCTD (sUCTD) based on their evolution toward a definable autoimmune syndrome during follow-up. Fisher’s Exact test and Mann-Whitney U test were applied to compare clinical and laboratory data between eUCTD and sUCTD groups, and to identify potential variables associated with progression to CTD. P values < 0.05 were considered statistically significant.

**Results:** Twenty-six patients (24 female) were included with a median age at diagnosis of 11.8 years (2.8-17) and a median follow-up of 3.6 years (1.0-5.0). The median age at onset was 9.4 years (6.6- 11.9), and the most common manifestations were arthralgia (53.8%), Raynaud's phenomenon (42.3%), morning stiffness (30.8%), skin lesions (30.8%), and fatigue (19.2%). Laboratory tests revealed a positive interferon-gamma signature in 75% of patients, an elevated erythrocyte sedimentation rate (ESR) in 58%, hypergammaglobulinemia in 53.8%, and elevated CPK levels in 40%. Twenty-four patients (92%) were ANA positive with variable titers and patterns. ENA was positive in 67% of patients, and the most common autoantibody specificities detected were anti-SSA/Ro (20.8%) and anti-CENP B (20.8%). Elevated RF was detected in 20.8% of patients, and the presence of lupus anticoagulant and anti-beta2 microglobulin I IgM was detected in 30% and 26.1% of patients, respectively. 38.5% of patients were treated with hydroxychloroquine and 30.8% received steroids during the course of the disease. During follow-up, 7 patients (26.9%) evolved to a differentiated CTD: four patients developed SLE, one was diagnosed with VEODOSS, one with juvenile dermatomyositis (MDA2 positive) and one with Sjögren's syndrome. Baseline ESR levels were significantly higher in the eUCTD patients (median ESR 28.9 mm/h) compared to the sUCTD patients (9 mm/h; p=0.0003). Skin manifestations and photosensitivity were also associated with the progression into a defined CTD. There were no deaths or chronic organ failure.

**Conclusion:** This is the first description of a cohort of pediatric patients with UCTD. The clinical and laboratory manifestations were similar to those described in adults. The ESR is a valuable biomarker to identify patients at higher risk of developing a defined CTD. Larger multicenter longitudinal studies are needed to develop consensus guidelines for the monitoring and management of pediatric UCTD.

**Patient Consent**

Yes, I received consent

**Disclosure of Interest**

None Declared

### Miscellaneous rheumatic diseases

#### P293 Recurrent pericarditis as presenting sign of Juvenile sjogren's syndrome: a case report

##### Andrea Leo, Camilla Sembenini, Maria Laura Cagnato, Francesca Tirelli, Alessandra Meneghel, Francesco Zulian

###### Department for Woman’s and Child’s Health, University Hospital of Padua, Pediatric Rheumatology Unit, Padua, Italy

####### **Correspondence:** Andrea Leo

*Pediatric Rheumatology 2024*, **22(2)**: PReS24-ABS-1382

**Introduction:** Juvenile Sjögren’s syndrome (jSS) is a multisystem autoimmune disease characterized by hypofunction of salivary and lacrimal glands and possible systemic manifestations. Classical symptoms, such as dry eye and dry mouth, may initially lack and develop later in children.

**Objectives:** Case report of recurrent pericarditis in otherwise asymptomatic jSS.

**Results:** A previously healthy 15-year-old boy was admitted to our Unit for acute chest pain without fever. EKG and echocardiography showed acute pericarditis with non-hemodynamically significant effusion. Blood tests showed: CRP 145 mg/L, ESR 80 mm/h, troponin I 50.8 ng/L (nv <34). He was treated with NSAIDs and maintenance therapy with colchicine was started. During NSAIDs tapering, he presented recurrence of acute pericarditis confirmed by EKG, echocardiography and laboratory tests. Oral prednisone was then started and, considering pericarditis recurrence, further rheumatological evaluations were performed. Fine speckled high titer (1:640) antinuclear antibodies (ANA) were found, along with positive anti-SSA/Ro (>240 kU/L), anti-SSB/La (158 kU/L) and rheumatoid factor (RF). Nailfold videocapillaroscopy showed an unspecific pattern. Pulmonary function tests with DLCO were normal. Parotid ultrasound showed hypoechogenic glands with a non-homogeneous structure, focal ductal ectasias and slightly increased vascularization, with enlargement of submandibular lymph nodes. These findings were also confirmed on MRI. Schirmer’s test showed lacrimal glands hyposecretion. Despite the complete absence of typical ocular and oral dryness symptoms, specific autoantibody positivity, altered salivary glands imaging and ocular tests lead us to confirm the diagnosis of primary jSS, according to EULAR/ACR classification criteria and ESSDAI questionnaire. Hydroxychloroquine was started while prednisone and colchicine were tapered.

**Conclusion:** Diagnosis of jSS is challenging in children because of the lack of typical organ involvement and the wide range of clinical manifestations. Given the rarity of the disease, there are no validated diagnostic criteria, therefore, actually, diagnosis is based on expert opinion. Cardiovascular involvement is rarely reported in SS both in children and in adults.

To our knowledge this is the first case of pediatric recurrent pericarditis as presenting sign for jSS. We suggest considering jSS in the differential diagnosis of serositis, even in the absence of typical organ involvement.

**Patient Consent**

Yes, I received consent

**Disclosure of Interest**

None Declared

### Miscellaneous rheumatic diseases

#### P294 Autoantibody profiles in primary and Secondary Juvenile Sjögren’s Syndrome (JSS)

##### Katharina Friedinger ^1^, Fiona Engelke^2^, Manuela Krumrey-Langkammerer^1^, Nadine Fischer^1^, Christian Klemann^3^, Torsten Witte^2^, Johannes-Peter Haas^1^

###### ^1^German Centre for Pediatric and Adolescent Rheumatology, Garmisch-Partenkirchen; ^2^Dep. for Rheumatology and Immunology, MH Hannover, Hannover; ^3^Dep. of Rheumatology and Immunology, Childrens´Hospital, University of Leipzig, Leipzig, Germany

####### **Correspondence:** Katharina Friedinger

*Pediatric Rheumatology 2024*, **22(2)**: PReS24-ABS-1125

**Introduction:** Juvenile Sjögren’s Syndrome (jSS) is a rare, chronic autoinflammatory disease involving the exocrine glands, predominantly salivary und lacrimal glands, and may also affect other organs (e.g. kidney, lung). JSS can manifest either as a primary autoimmune disease or secondary in the context of other inflammatory systemic diseases (e.g. systemic lupus erythematosus (SLE), mixed connective tissue disease (MCTD). Infiltration of the glandular parenchyma by memory B-cells and typical autoantibodies (anti-Ro/SSA and anti-La/SSB) are pathognomonic. Moreover, patients with jSS have an increased risk to develop B-cell lymphomas.

**Objectives:** Analyzing autoantibodies in juvenile Sjögren´s Syndrome to characterize typical profiles.

**Methods:** Presence of IgG autoantibodies (aAB) against 67 antigens already tested in an adult SS-population were analysed in 29 patients with confirmed jSS using Luminex method. The cut off value was determined using the ≥98 quantile of the fluorescence intensity values in healthy controls (n=123).

**Results:** The cohort (female n= 26; 89.7%) included patients with primary jSS (n=15; 51.7%) and secondary jSS (n=14; 48.3%), the latter diagnosed as MCTD(n=10), SLE(n=3) and systemic sclerosis (n=1). The mean age of onset in jSS patients was 11.2 years. The mean values were 48.2 months for disease duration and 9.2 points for the ESSDAI at the time of blood sampling. Maximum ESSDAI within the complete course of disease was on average 15.4 points. Identical to adults with SS, we found predominantly anti-Ro/SSA und anti-La/SSB antibodies in the jSS group. Here, 44.8% (n=13) were positive for Ro52+ and Ro 60+ and 24.1% (n=7) were positive for Ro52+ only, where as the combination of Ro52- and Ro60+ was not found in any jSS patient. In addition to the autoantibodies already known in adult SS, SNRPB2 was observed with increased frequency of 41.4% in patients with jSS.

**Conclusion:** Autoantibody profiles in patients with jSS appear to be comparable to the adult SS-cohorts with regard to Ro52, while autoantibodies against Ro60 were less frequently detected. The presence of autoantibodies against SNRPB2 in children with jSS requires further investigation in terms of its clinical and pathophysiological relevance.

**Patient Consent**

Not applicable (there are no patient data)

**Disclosure of Interest**

None Declared

### Miscellaneous rheumatic diseases

#### P295 Determination of the relationship between kobayashi,sano,egami criteria and prevalance of intravenous immunoglobulin resistance and coronary artery aneurysm in Iranian children with kawasaki disease

##### Reza Shiari^1^, Niloofar shashaani ^1^, khosro rahmani^1^, Azadeh Zeinab mirzaee^2^, vadood javadi parvaneh^1^

###### ^1^Pediatric Rheumatology, Mofid Children Hospital; ^2^Pediatric Rheumatology, Masih Daneshvari Hospital, tehran, Iran, Islamic Republic Of

####### **Correspondence:** Niloofar Shashaani

*Pediatric Rheumatology 2024*, **22(2)**: PReS24-ABS-1757

**Introduction:** Introduction: Kawasaki disease (KD) is a systemic vasculitis that occurs mostly in children under five years old. Kawasaki affects the middle-size arteries, especially the coronary arteries. Therefore, without adequate treatment, it may cause coronary artery aneurysm in 25% of patients. The purpose of this study was to investigate the relationship between Kobayashi, Sano, and Egami criterions with coronary artery aneurysm in KD patients during the last ten years and to identify risk factors in patients with intravenous immunoglobulin (IVIG)-resistant and coronary artery aneurysms.

**Objectives:** Statistical Analysis Data were analyzed with SPSS software and MedCalc version 12.7.1.0. To analyze the continuous data, we used the Student’s t-test for normally distributed variables and Kruskal-Wallis for non-parametric variables. One-way analysis of variance (ANOVA) was used to compare multiple group averages. A linear logistic regression analysis was performed in an attempt to determine predictors for IVIG resistance. In all the analyses, a two-sided p-value of <0.05 was considered statistically significant. Means were accompanied by standard deviations and medians by ranges.

**Methods:** Methodology: Medical records of 363 Kawasaki patients referred during 2008–2017 were reviewed. Patients’ demographic data and Kobayashi, Sano, and Egami scores of each patient were calculated. Based on echocardiographic findings, cases of coronary artery aneurysm were determined. Sensitivity, specificity, positive and negative predictive value, and the accuracy of each criterion were determined to predicting IVIG resistance and detect coronary artery aneurysm.

**Results:** Results: There was a slight relationship between IVIG-resistance in Kawasaki children and its prediction based on the Kobayashi risk score, but no relationship was found between the Egami and Sano criteria. Sixty-three patients (17.4%) had coronary artery lesions (CALs) on time of diagnosis. There were no statistically significant differences between gender and mean age of children with and without CALs. Also, there was no significant relationship between coronary artery aneurysm in Kawasaki children and its prediction based on the above three risk factors. The area under the ROC-curve of all three risk measures of Kobayashi, Egami, and Sano indicated that all three criteria were not useful in predicting CALs.

**Conclusion:** Conclusion: Despite the low accuracy of the three above criteria to predictive of patients with IVIG resistance, it seems that the variables of age, duration of fever, and C-reactive protein (CRP) are more useful than other variables and may be utilized to evaluate patients by establishing a more appropriate cut-off point.

**Date of birth::** décembre 3

**Patient Consent**

Not applicable (there are no patient data)

**Disclosure of Interest**

None Declared

**References**

1. Shiari R. Kawasaki disease; A review article. Arch Pediatr Infect Dis. 2014;2(1):154–159.
2. Kawasaki TJA. Acute febrile mucocutaneous syndrome with lymphoid involvement with specific desquamation of the fingers and toes in children. Arerugi. 1967;16:178–222.
3. Newburger JW, Takahashi M, Gerber MA, et al. Diagnosis, treatment, and long-term management of Kawasaki disease: a statement for health professionals from the committee on Rheumatic Fever, Endocarditis and Kawasaki Disease, council on Cardiovascular Disease in the young, American Heart Association. Circulation. 2004;110(17):2747–2771.

### Miscellaneous rheumatic diseases

#### P296 Quality of life in pediatric patients with rheumatological diseases and disorders of the brain-gut axis

##### Camilo A. Vargas-Rincon, Carlos Velasco-Benítez, Diana Castilla-Bolaños

###### Pediatrics, Universidad del Valle, Cali, Colombia

####### **Correspondence:** Camilo A. Vargas-Rincon

*Pediatric Rheumatology 2024*, **22(2)**: PReS24-ABS-1548

**Introduction:** The prevalence of rheumatological diseases (RD) in the pediatric population ranges between 5.0-7.0% and worldwide a prevalence of disorders of the brain-gut axis (DGBI) has been determined to be 23.0%. Previous studies have found increased gastrointestinal involvement in patients with (RD), which in turn leads to poorer quality of life. However, currently there are few and limited studies in this population.

**Objectives:** Describe the prevalence of DGBI and its effects on quality of life in children diagnosed with RD.

**Methods:** Prospective descriptive observational study. Children between 5 and 18 years old who attended the pediatric rheumatology outpatient clinic at the Hospital Universitario del Valle “Evaristo García” in Cali, Colombia between May and November 2023 were included. The validated Roma IV questionnaire for pediatric digestive symptoms was applied to them. Spanish (QPGS-IV) to identify the presence of DGBI and the KIDSCREEN questionnaire to assess quality of life. Sociodemographic variables, clinical variables, family variables and quality of life variables were included.

**Results:** 58 children were included (13.4+/-3.7 years, 70.7% female, 69.0% adolescents, 46.6% mixed race and 78.9% from public school). 48.3% were born by cesarean section; 22.4% were premature and 3.4% had some comorbidity. The prevalence of DGBI was 39.7%, with the main being functional dyspepsia (17.2%; n=10) and functional constipation (17.2%; n=10), followed by functional abdominal pain. not otherwise specified (3.5%; n=2). 12.1% had 2 or more DGBI, the most frequent coexistence being dyspepsia associated with functional constipation (3.5%). A statistically significant lower KIDSCREEN score was found in children with DGBI compared to those without (p=0.0043), mainly in children with a diagnosis of systemic lupus erythematosus (SLE) (p=0.0209).

**Conclusion:** The prevalence of DGBI in children with RD is almost double compared to the general population, with dyspepsia and functional constipation being the most prevalent, drawing attention to the fact that dyspepsia is more prevalent in juvenile idiopathic arthritis and constipation in SLE. There was a worse quality of life in children with DGBI and RD; This more than anything for children with autoimmune diseases, and of them SLE.

**Patient Consent**

Yes, I received consent

**Disclosure of Interest**

None Declared

### Miscellaneous rheumatic diseases

#### P297 Vaccination frequency in pediatric patients with rheumatological diseases

##### Luz Del C. Reynoso Medina, María M. Rodríguez Reyes, María De L. Aldana Galván, Ana V. Villarreal Treviño, Fernando García Rodríguez, Nadina E. Rubio Pérez

###### Pediatric Rheumatology, Hospital Universitario "Dr. José Eleuterio González", Monterrey, Mexico

####### **Correspondence:** Ana Victoria Villarreal Treviño

*Pediatric Rheumatology 2024*, **22(2)**: PReS24-ABS-1352

**Introduction:** Pediatric patients with rheumatologic diseases are considered immunosuppressed, due to the alterations in humoral and cellular immunity that characterize them and due to the immunosuppressive therapy used, leading to higher susceptibility to infections. Protection against infectious diseases is vital in management. Vaccines can prevent some of these infections; however, several factors, including disease activity, immunosuppressive treatment, infection risk, and vaccine safety and efficacy, must be considered during vaccination. Current guidelines recommend immunization in patients with rheumatologic diseases and with use of immunosuppressants and biological treatment. Despite this, the frequency of immunization in our population is unknown.

**Objectives:** To determine the frequency of vaccination among patients with pediatric rheumatic diseases in a single center in Mexico.

**Methods:** Descriptive, cross-sectional study. Non-random, consecutive sampling. Pediatric patients diagnosed with rheumatologic diseases attending to the Pediatric Rheumatology Service at the University Hospital "Dr. José Eleuterio González" were included if their vaccination records were available in the month of April 2024.

**Results:** Twenty patients were included, with a median of 11.5 years (Maximum 16, minimum 3, IQR 6), 50% were female. The diagnoses were: Juvenile Idiopathic Arthritis (13; 65%), Juvenile Dermatomyositis (3; 15%), Juvenile Systemic Lupus Erythematosus (2; 10%), Juvenile Scleroderma (1; 5%), and IgG4-Related Disease (1; 5%). The majority of patients (18; 90%) receiving immunosuppressive therapy. More than half of the patients (13; 65%) did not have a complete vaccination schedule for their age according to national regulations; of these, 13 (100%) did not have the influenza vaccine booster, and only 1 patient did not have the MMR vaccine. 11 (84.6%) reported not having get vaccinated because they did not attend the established vaccination campaigns, 1 (7.6%) because they had a respiratory tract disease and 1 (7.6%) did not want to be vaccinated. Some patients (12; 60%) received vaccines that are not included in the National Vaccination Schedule: COVID-19 (11; 91.6%), Varicella (1; 7.6%), and Hepatitis A (1; 7.6%). The patient's guardian was asked if they had received any specific vaccination recommendations from the pediatric rheumatologist, and a minority (6; 30%) responded affirmatively.

**Conclusion:** The majority of our pediatric rheumatology patients (90%) are on immunosuppressive treatment, and more than half do not have a complete vaccination schedule for their age (65%). Additionally, only a minority (30%) reported receiving vaccination recommendations from the pediatric rheumatologist. This highlights the importance of providing information and raising awareness about proper vaccination in these patients, as this action reduces the risk of serious infectious processes that may result in hospitalization.

**Date of birth::** août 03, Y

**Patient Consent**

Not applicable (there are no patient data)

**Disclosure of Interest**

None Declared

### Miscellaneous rheumatic diseases

#### P298 A clinical case of polyarthritis in a patient with two rare genetic syndromes (Robinow and Phelan-Mcdermid): is it Juvenile idiopathic or non-idiopathic arthritis?

##### Irina Nikishina, Valeria Matkava, Svetlana Arsenyeva, Aliya Arefieva

###### Paediatric, V.A. Nasonova Research Institute of Rheumatology, Moscow, Russian Federation

####### **Correspondence:** Valeria Matkava

*Pediatric Rheumatology 2024*, **22(2)**: PReS24-ABS-1731

**Introduction:** Some genetic disorders has very different manifestations which may «hide» other important signs such as musculoskeletal involvement. Robinow syndrome (RS) is a rare autosomal dominant genetic disease characterized by distinctive facial features, short stature, and skeletal abnormalities. Phelan-McDermid syndrome (PMS), also known as 22q13.3 deletion syndrome presented with severe intellectual disability motor delay and autistic traits. Cognitive impairment in these patients makes it difficult to diagnose somatic disorders.

**Objectives:** To describe a clinical case of rare comorbidity RS, PMS associated with JIA.

**Methods: Case report:** Girl 17 y.o. admitted to our clinic with two confirmed genetic conditions. RS was presented by skeletal involvement (short stature, mesomelic limb shortening, brachydactyly), dysmorphic facial features (widely spaced eyes, midface hypoplasia, epicant), dental abnormalities (prognathia, oligodentia, fibrous gingival dysplasia) and established by the identification of heterozygous pathogenic variant in WNT5A gene through molecular genetic testing. PMS was presented by developmental delay, lack of expressive speech, specific awkward gait with frequent falls and diagnosed by detection of a de novo heterozygous deletion of chromosome 22q13.3.

**Results:** Since 2019 patient had suffered from hips, knees arthralgias with progression of pain and functional impairment which were flared after severe course of coronavirus disease and measles vaccination. Patient was repeatedly examined by various medical specialists and none of them found signs of inflammation despite the fact that patient had synovitis in the TMJs and knee joints by imaging. She was examined in our paediatric department and clinical evidences of polyarthritis with swelling and joints contractures (wrists, hips, knees, ankles, metacarpophalangeal, tarsophalangeal joints) were found. Cervical spine and TMJs were also involved. Imaging detected signs of symmetric coxitis, synovitis in wrists, knees, ankles, intercarpal, talofemoral joints, multiple tenosynovitis. Patient was treated by NSAIDs with mild positive effect. Considering presence of active polyarthritis, insufficient response to NSAIDs, we decided to start therapy with the JAK-inhibitor tofacitinib. We received prompt positive response to tofacitinib therapy: reduction of pain syndrome, increasing functional activity and unexpected improvement in mental status. There is no adverse event.

**Conclusion:** Despite the presence of two verified genetic diseases and musculoskeletal symptoms, it is not clear whether the arthritis is a manifestation of classic immunoinflammatory disease, such as JIA or it is mediated by genetic conditions. Regardless, reliable inflammatory changes allowed us to extrapolate from rheumatologic experience targeted drug therapy with successful outcome.

**Date of birth::** 08.06.1995

**Patient Consent**

Yes, I received consent

**Disclosure of Interest**

None Declared

### Miscellaneous rheumatic diseases

#### P299 Progressive pseudorheumatoid dysplasia: case presentation of two pediatric patients

##### Meraj A. Siddiqui^1^, Esra Baskın^2^, Halil I. Aydın^3^

###### ^1^Department of Pediatrics; ^2^Department of Pediatric Rheumatology; ^3^Department of Pediatric Metabolic Diseases, Başkent University Faculty of Medicine, Ankara, Türkiye

####### **Correspondence:** Meraj A. Siddiqui

*Pediatric Rheumatology 2024*, **22(2)**: PReS24-ABS-1418

**Introduction:** Progressive pseudorheumatoid dysplasia (PPD) is a rare autosomal recessive disorder characterized by non-inflammatory progressive arthropathy. A genetic defect causes progressive degeneration in joint cartilage (1) . Typically starting with symmetric joint swelling and pain in childhood, PPD is often confused with more common pediatric rheumatic diseases such as juvenile idiopathic arthritis due to clinical similarities (2). Radiographic findings typically display characteristic changes like vertebral flattening and peripheral metaphyseal widening, which are important for distinguishing PPD from other conditions. This disease leads to progressive cartilage loss, joint stiffness, joint enlargement, and skeletal abnormalities, without the systemic inflammatory features typical of JIA.

**Objectives:** Misdiagnosis of PPD can lead to inappropriate treatments. Here, we present two siblings diagnosed with PPD to highlight the need for a genetic approach in cases of arthropathy with atypical courses.

**Methods:** *Case 1 (17-year-old male):* The patient first presented at the age of 7-8 with swelling in the fingers, leg deformities, later immobility, and a history of corrective orthopedic surgery. He had previously received treatments for JIA, which had not been beneficial. It was noted that his parents were related. Physical examination revealed bilateral metacarpophalangeal and interphalangeal joint swelling, pronounced kyphosis, deformities in the lower extremities, muscle atrophy, and swelling in both knees and toes, resulting in the inability to walk and reliance on a wheelchair. Despite these findings, he reported no significant pain. The patient's growth metrics were significantly below the standard percentile for his age. Acute phase reactants were normal, and radiographic examinations showed epiphyseal widening in the hand joints. MRI also displayed similar changes, indicating a non-inflammatory polyarticular process. *Case 2 (9-year-old male):* The younger brother of Case 1 presented with swelling in the fingers, wrists, and knees, and difficulty walking. Despite sharing similar clinical features, he did not have the advanced orthopedic complications seen in his older brother. Family history, radiological, and clinical findings prompted genetic testing of the patients.

**Results:** *Genetic Analysis:* The results revealed a homozygous mutation in the CCN6 gene, confirming the diagnosis of PPD. These findings played a critical role in differentiating the patients from juvenile idiopathic arthritis and other musculoskeletal disorders.

**Conclusion:** These two cases highlight the complexities in identifying rare genetic musculoskeletal disorders. In pediatric rheumatology, evaluating clinical, radiological, and genetic analyses together is crucial in approaching patients. Timely and accurate recognition of PPD allows for avoiding unnecessary treatments and implementing effective management strategies.

**Patient Consent**

Yes, I received consent

**Disclosure of Interest**

None Declared

**References**

1. Pulsatelli L, Manferdini C, Gabusi E, et al. Mesenchymal stromal cells from a progressive pseudorheumatoid dysplasia patient show altered osteogenic differentiation. Eur J Med Res. 2022;27(1):57.
2. Riaz M, Khoso Z, Rai VR, et al. A Rare Skeletal Dysplasia-Close Mimicker Of Juvenile Idiopathic Arthritis-Progressive Pseudorheumatoid Dysplasia. J Ayub Med Coll Abbottabad. 2022;34(Suppl 1)(4):S1050-S1052.

### Miscellaneous rheumatic diseases

#### P300 Tafro syndrome: the first report of two pediatric cases successfully treated with interleukin-1 inhibition

##### Serena Palmeri^1, 2^, Riccardo Papa^1^, Silvia Rosina^1^, Stefano Volpi^1, 2^, Valentina Natoli^1, 2^, Caterina Matucci-cerinic^1, 2^, Marco Gattorno^1^, Roberta Caorsi^1, 2^

###### ^1^IRCCS Istituto Giannina Gaslini, Paediatric Rheumatology and Autoinflammatory Diseases Unit; ^2^Università degli Studi di Genova, Dipartimento di Neuroscienze, Riabilitazione, Oftalmologia, Genetica e Scienze Materno-Infantili (DiNOGMI), Genova, Italy

####### **Correspondence:** Serena Palmeri

*Pediatric Rheumatology 2024*, **22(2)**: PReS24-ABS-1454

**Introduction:** TAFRO syndrome (Thrombocytopenia, anasarca, fever, reticulin fibrosis, renal insufficiency, and organomegaly) is a potentially life-threatening inflammatory condition with sudden onset, that can occur in the context of idiopathic multicentric Castleman disease (iMCD), infectious diseases, neoplasms, and connective tissue disorders. By now, most cases have been treated with interleukin-6 (IL-6) inhibitors. Here, we present two paediatric cases of TAFRO successfully treated with anakinra.

**Objectives:** To report two pediatric cases of TAFRO syndrome successfully treated with interleukin-1 (IL-1) inhibitor anakinra.

**Methods:** We described the initial presentation, clinical course, laboratory and imaging results, treatment and outcome of two pediatric patients with TAFRO syndrome presentation, followed at our tertiary referral center.

**Results: Case 1.** A 14-year-old Caucasian male presented with persistent fever, headache, vomiting, and positive Helicobacter pylori antigen. Symptoms progressed to an inflammatory syndrome with fever, and generalized anasarca, necessitating ICU admission. A lymph node biopsy confirmed Castleman's Disease suspicion. Acute renal failure required hemodialysis; renal biopsy revealed tubular necrosis. Central venous catheter removal led to dyspnoea and hemoptysis from superior vena cava thrombosis, treated with heparin. For the onset of seizures a brain MRI was performed, showing leptomeningeal involvement. **Case 2.** An 8-year-old North African female was transferred to our institute due to persistent fever associated with vomiting, hepatosplenomegaly, hypoalbuminemia, coagulopathy, and progressive development of anasarca. A change in behaviour with aggressiveness prompted a brain MRI, revealing a small frontal ischemic focus requiring heparin therapy. Blood and platelet transfusions were required. Total body MRI showed multiple lymphadenopathies; axillary lymph node biopsy confirmed histology compatible with Castleman's disease. Interestingly, the patient tested positive for anti-SSA/Ro and SSB/Ra autoantibodies on multiple occasions, despite never experiencing xerostomia or xerophthalmia and having normal salivary gland ultrasound at onset. Salivary gland biopsy, however, revealed lymphocytic infiltration. In both cases, given the poor response to antibiotics, high-dose intravenous steroid therapy, combined with interleukin-1 inhibitor (anakinra) and supportive treatment, allowed to control the clinical picture. The two patients are still in treatment with IL-1-inhibitor for 6 years and 6 months respectively with complete control of the disease.

**Conclusion:** We describe the first pediatric cases of TAFRO treated with anakinra. Due to its short half-life and good tolerability, anakinra represents an excellent therapeutic option for patients with inflammatory life-threatening conditions, such as TAFRO syndrome.

**Patient Consent**

Yes, I received consent

**Disclosure of Interest**

S. Palmeri: None Declared, R. Papa: None Declared, S. Rosina: None Declared, S. Volpi Speaker Bureau with: Sobi, V. Natoli: None Declared, C. Matucci-cerinic: None Declared, M. Gattorno Speaker Bureau with: Novartis, Sobi, Fresenius Kabi, Kiniksa, R. Caorsi Speaker Bureau with: Novartis, Sobi

### Miscellaneous rheumatic diseases

#### P301 Evaluation of clinical and laboratory features of pediatric patients with behçet's disease

##### Mesut Kara^1^, M. Hakan Poyrazoğlu ^1^, Esra Esen^1^, Ayşenur Paç Kısaarslan^1^, Duygu Gülmez Sevim^2^, Demet Kartal^3^, Sümeyra Çiçek^1^

###### ^1^Pediatric Rheumatology; ^2^Ophtalmology; ^3^Dermatology, Erciyes University, Kayseri, Türkiye

####### **Correspondence:** M. Hakan Poyrazoğlu

*Pediatric Rheumatology 2024*, **22(2)**: PReS24-ABS-1472

**Introduction:** Behçet's disease (BD) is a chronic, systemic vasculitis with periods of exacerbation and remission caused by environmental and genetic factors. Behçet's disease is mostly seen in the second and fourth decades and its incidence in children is less common. The clinical manifestation pattern in pediatric BD is heterogeneous and varies in different genders, ethnicities, and geographic regions. There are also some differences in clinical presentations and prognosis between pediatric and adult BD. A limited number of studies reported issues about pediatric BD.

**Objectives:** In this study, It was objected to determine the demographic, clinic, laboratory and system involvement characteristics of Behçet's disease patients with pediatric onset, to contribute to the literature with the data obtained from the study.

**Methods:** The data of the patients who were followed up in Erciyes University Faculty of Medicine, Division of Pediatric Rheumatology was retrospectively analyzed. The study included 52 patients. Gender, age at the time of diagnosis, time between the onset of symptoms and diagnosis, follow-up period, presence of Behçet's disease in first-degree relatives, system involvement, HLA-B51 positivity, skin pathergy test positivity, laboratory tests and treatment options were evaluated. In terms of prognosis, variables such as response to treatment at 6 months, response to treatment at 12 months, complete remission with drug treatment, partial remission, treatment-resistant disease and mortality were analyzed.

**Results:** 55.8% of the patients were female and the average age of onset of the disease was 11 years. The median time from disease onset to diagnosis was 11 months. 26.9% of the patients had a history of Behçet's disease in their first-degree relatives. HLA-B51 test was positive in 50% of the patients, and pathergy test was positive in 18.1%. While pseudotumor cerebri was observed in 24% of patients with a positive HLA-B51 test, pseudotumor cerebri was not observed in any of the HLA-B51 negative patients. It was determined that all patients used colchicine, and the most commonly used immunosuppressive drugs were corticosteroids and azathioprine. The average follow-up period of the study group was 40.5 months. It was determined that 71.2% of the patients responded to the treatment completely or partially in the 6th month of treatment and 80.8% in the 12th month. At the end of the follow-up, it was determined that 26.1% of the patients were in complete remission and drug-free, and 73.9% were in complete or partial remission but continued drug treatment. It was determined that those who responded to treatment in the first six months were more likely to achieve complete remission.

**Conclusion:** In our study group, the disease appeared in the second decade of life, consistent with the literature. The higher rate of pseudotumor cerebri in HLA-B51 positive patients suggested that HLA-B51 positivity may predispose to neurological involvement. The fact that patients in complete remission respond better to treatment at the 6th month suggests that early response to treatment may be an important indicator of better prognosis and that early diagnosis and treatment are important.

**Patient Consent**

Yes, I received consent

**Disclosure of Interest**

None Declared

**References**

1. Ozen S, Eroglu FK. Pediatric-onset Behçet disease. Curr Opin Rheumatol. 2013;25(5):636–42.
2. Yildiz M, Koker O, Adrovic A, Sahin S, Barut K, Kasapcopur O. Pediatric Behçet’s disease - clinical aspects and current concepts. Eur J Rheumatol. 2020;7(1):38–47.
3. Yildiz M, Haslak F, Adrovic A, Sahin S, Koker O, Barut K, et al. Pediatric Behçet’s Disease. Front Med. 2021;8(February):1–10.

### Miscellaneous rheumatic diseases

#### P302 Childhood-onset sjögren's disease: a descriptive analysis of a single-center longitudinal retrospective cohort

##### Francesco Baldo^1^, Achille Marino^1^, Maurizio V. Gattinara^1^, Stefania Costi^1^, Veronica Gerli^2^, Cecilia B. Chighinzola^1, 2^, Roberto F. Caporali^1, 2^

###### ^1^ASST G.Pini-CTO; ^2^University of Milano, Milano, Italy

####### **Correspondence:** Francesco Baldo

*Pediatric Rheumatology 2024*, **22(2)**: PReS24-ABS-1540

**Introduction:** Childhood onset Sjögren’s disease (CoSD) is a rare and often underdiagnosed condition that significantly impacts children’s quality of life. It is a distinct entity from the adult counterpart, and evidence regarding the management and long-term evolution of the disease in pediatric patients is limited

**Objectives:** To describe a monocentric cohort of patients diagnosed with CoSD between 1991 and 2024, focusing on clinical manifestationsand therapeutic interventions.

**Methods:** A retrospective analysis of available medical records at our center between 1991 and 2024 was performed. All patients with diagnosis of CoSD were included.

**Results:** We identified 14 pSS patients, with a male-to-female ratio of 3:11. The median age at disease onset was 10.8 years (IQR: 7.9–14.5), and the median age at diagnosis was 14.9 years (IQR: 11.3–16.5).Family history was positive for rheumatologic conditions in 5/14 patients (1SLE, 1SpA,1AR, 1Raynaud’s phenomenon, 1 autoimmune thyroid disease). Most common manifestations at first evaluation were salivary gland swelling (6/14) and recurrent parotits (5/14) s, followed by cutaneous manifestations (3 purpura, 2 erythema nodosum, 3 other skin rashes). 8/14 patients complained of sicca symptoms at diagnosis but only 3/14 had positive Schirmer’s test. Nine/14 patients had hypergammaglobulinemia at diagnosis, 6/14 were RF positive while ANA were invariably positive. Twelve/14 were anti-SSA-Ro positive, and of the two remaining patients one was anti SSB-La positive (in total 7/14 were SSB-La positive). Whenever performed (8 cases), salivary gland US documented sialadenitis. 7/14 subjects a median age of 13,5 y(11,1-16,3), underwent a salivary gland biopsy,which showed lymphocytic sialadenitis in all cases. Over a mean follow up of 7,8y(SD8.1), 13/14 were treated with hydroxychloroquine (HCQ), 5/14 received oral steroid therapy, 5 were administered rituximab (RTX), 4 were prescribed methotrexate (MTX) and 1 azathioprine (AZA); belimumab was prescribed in 1 case. Main side effects due to therapy were hypogammaglobulinemia following RTX requiring substitutive treatment (1 case), gastric intolerance to MTX (1 case), telogen effluvium attributed to HCQ (1 case). No malignancies were observed during the follow-up period. The mean European League Against Rheumatism Sjögren's Syndrome Disease Activity Index (ESSDAI) score decreased from 5 to 2.9, indicating disease activity reduction over time.

**Conclusion:** Key findings of this study include the prominence of anti-SSA and anti-SSB antibodies, positive ANA, hypergammaglobulinemia, and recurrent salivary gland manifestations at disease onset. Salivary gland US resulted being a useful tool supporting the diagnosis of CoSD. Between the onset of the disease and the diagnosed, a ndiagnostic delay was observed. Treatment with HCQ, oral steroids, and RTX seem to show efficacy and acceptable safety profiles within our cohort, with regular monitoring recommended, particularly for potential side effects of RTX

**Patient Consent**

Not applicable (there are no patient data)

**Disclosure of Interest**

None Declared

### Miscellaneous rheumatic diseases

#### P303 Childhood-onset primary sjogren’s syndrome: clinical features, immunological profile, and clinical management of an Irish cohort

##### Marah Shaikh Yousef, Emma J. MacDermott, Orla G. Killeen

###### National Centre for Paediatric Rheumatology, Children’s Health Ireland (CHI) @ Crumlin, Dublin, Ireland

####### **Correspondence:** Marah Shaikh Yousef

*Pediatric Rheumatology 2024*, **22(2)**: PReS24-ABS-1589

**Introduction:** Primary Sjogren’s Syndrome (pSS) is a systemic autoimmune disease that is characterized by inflammation of the exocrine glands and primarily affects middle-aged women.^1^ Childhood onset pSS is rarely reported and underdiagnosed due to the limited data available.^1^ Published reports have identified distinct features of paediatric cases and neither the American-European Consensus Group nor the American college of Rheumatology/EULAR criteria are validated in the Paediatric population.^1^ We conducted this study to identify and report our recent clinical experience with childhood-onset pSS and to expand on the current knowledge repertoire published as well as evaluating the diagnostic utility of proposed criteria to date. We also conducted a literature review for comparison with published cases to date.

**Objectives:** This study aims to characterize the clinical presentation, immunological profile, and management of patients diagnosed with pSS in our tertiary paediatric centre.

**Methods:** We conducted a retrospective chart review of patients diagnosed with pSS in the last decade (2013-2023) at the National Centre for Paediatric Rheumatology. Data was collated anonymously and analysed using SPSS. Descriptive statistics were used to summarise the data. Ethical approval was obtained (REC-403-24).

**Results:** Nine patients in total identified; 77.8% (n=7/9) female; mean age at the time of first symptom onset was 10.5 years (± 3.71) and the mean age at diagnosis was 11.2 years (± 3.89). Commonest presenting complaint was dry mouth (66.7% n=6/9), followed by dry eyes (55.5% n=5/9), and recurrent parotid swelling (55.5% (n=5/9). Other features included: fatigue (n=4), rash (n=3), mouth ulcers (n=3), and ranula (n=1). Autoantibody profile identified a positive ANA titre>1/160 (55.5%, n=5/9), positive anti-Ro antibody (66.7% (n=6/9), and positive anti-La antibody (55.5%, n=5/9). Only 2 patients had raised ESR 33.3%, n=2/9). Eight of the nine patients had a parotid ultrasound; 77.8% (n=7/9) of the ultrasound scans identified heterogenous parotid glands. One patient underwent a sublabial biopsy which identified lymphocytic sialadenitis. Management included: hydroxychloroquine (7/9), methotrexate (2/9), and mycophenolate mofetil (1/9).

**Conclusion:** Clinical features of paediatric pSS is variable with a predominance of extra glandular manifestation as compared to the adult cohort. Validated diagnostic criteria are required for paediatric cases. Given the rarity of this condition, it will require collaboration and the consensus of international working groups to define this complex autoimmune disorder in greater detail.

**Patient Consent**

Not applicable (there are no patient data)

**Disclosure of Interest**

None Declared

**Reference**

1. Randell RL, Stern SM, Van Mater H, Schanberg LE, Lieberman SM, Basiaga ML, CARRA Sjögren Workgroup, CARRA Investigators. Pediatric rheumatologists’ perspectives on diagnosis, treatment, and outcomes of Sjögren disease in children and adolescents. Pediatric Rheumatology. 2022 Sep 5;20(1):79.

### Miscellaneous rheumatic diseases

#### P304 A case of lipoatrophic panniculitis with systemic inflammation occurring soon after hpv vaccine which responded well to tocilizumab

##### Lucy Paterson-Brown^1^, Jo Walsh^1^, Thomas Savage^2^, Neil Martin^1^

###### ^1^Paediatric Rheumatology; ^2^Radiology, Royal Hospital for Children, Glasgow, United Kingdom

####### **Correspondence:** Lucy Paterson-Brown

*Pediatric Rheumatology 2024*, **22(2)**: PReS24-ABS-1638

**Introduction:** There are documented cases of localised lipodystrophy, erythema nodosum or new onset SLE occurring soon after HPV vaccine. To our knowledge no cases of Lipoatrophic Panniculitis or Weber-Christian Disease have been described as occurring after HPV vaccine.

**Objectives:** Describe a case of Lipoatrophic panniculitis with systemic inflammation after HPV vaccination with complete resolution & no recurrence since treatment with Tocilizumab.

**Methods:** Case report

**Results:** A previously well 12 year old boy developed progressive, symmetrical erythema, pain, reduced mobility and ankle swelling 10 days following HPV vaccination. Erythema progressed from feet to mid-thigh with painful palpable popliteal fossa lymph nodes and fever. He was unable to walk more than a few steps due to pain. Thermal imaging showed increased heat in the affected areas which felt woody with an erythematous leading edge.

Investigations: ANA 1/640, ESR 40, Coombs positive, CRP 74, LDH 363. Later he developed low C3 and C4. Alpha1-antitrypsin was normal. MRI of legs showed bilateral extensive signal abnormality of the subcutaneous tissues and fascia, affecting proximal tissues more than distal with relative muscle sparing. Biopsy showed acute on chronic lobar panniculitis of the skin with fat necrosis and lipoatrophy, a reactive bone marrow, and a reactive picture on lymph node biopsy. Pulmonary function tests detailed a restrictive lung defect. Whole Body MRI showed widespread abnormal marrow signal, splenomegaly, widespread lymphadenopathy and soft tissue oedema affecting upper and lower limbs.

He initially received 3 doses of IV Methylprednisolone followed by oral Prednisolone. Methotrexate SC was commenced. 3 weeks later IV Tocilizumab was added when panniculitis extended clinically to involve his arms. He responded well to this and had inactive disease within 3 months. He is now in remission off treatment, complement normalised and he has a normal skin examination with some residual lipoatrophy of his feet.

**Conclusion:** A rapid and debilitating case of Lipoatrophic Panniculitis occurred soon after HPV vaccine. Despite Steroids and Methotrexate this continued to extend but resolved with Tocilizumab and has not recurred. Tocilizumab may be a useful treatment option for Lipoatrophic Panniculitis or Weber-Christian disease.

**Patient Consent**

Yes, I received consent

**Disclosure of Interest**

None Declared

### Miscellaneous rheumatic diseases

#### P305 A first case of kikuchi-fujimoto’s disease, and salivary and parotid gland involvement in children

##### Abdallah Aloteiby^1^, Samer Ali^1^, Buthaina Al-Adba^2^, Sharon Bout-Tabaku^2^

###### ^1^General Pediatric; ^2^Pediatric Rheumatology, Sidra Medicine, Doha, Qatar

####### **Correspondence:** Samer Ali

*Pediatric Rheumatology 2024*, **22(2)**: PReS24-ABS-1409

**Introduction:** Kikuchi-Fujimoto disease (KFD), also known as histiocytic necrotizing lymphadenopathy, is a rare benign disorder primarily affecting young female adults^.^ Characterized by necrotizing lymphadenitis, its etiology remains largely unknown, with theories suggesting infectious and/or autoimmune processes. While KFD typically presents with non-specific symptoms such as fever, lymphadenopathy, fatigue and body rash are the frequently encountered signs and symptoms in adults, as well as in children.

**Objectives:** We present a unique case of KFD in a 14-year-old female with concomitant salivary and parotid gland involvement, a rare occurrence in the pediatric age group. We emphasize the importance of considering KFD in the differential diagnosis of pediatric patients presenting with fever and lymphadenopathy, especially within cervical chain involvement.

**Results:** A 14-year-old female, previously healthy, presented with an acute history of fever and left-sided neck swelling, associated with erythematous-popular rash over the face, neck, and chest. Initial laboratory investigations revealed leukopenia of 2.6x10^3/uL and elevated inflammatory markers, CRP 33 mg/L, ESR 64 mm/hr, ferritin of 415 ug/L and D-Dimer 4429 ng/mL. The peripheral smear showed no blast cells. Infectious workup came back negative, including Mycobacterium tuberculosis. Serial labs showed persistent leukopenia and elevated inflammatory markers despite a course of empirical antibiotics.

An MRI of the neck was performed and showed multiple abnormal glands and cervical lymph nodes involvement.

Excisional lymph node biopsy was performed and showed a partial preservation of the follicular architecture and several areas of paracortical necrosis. with a diffuse mixed inflammatory cell infiltrate. There was abundant single cell apoptosis. There were no viral cytopathic features and no evidence of pathogenic organisms. EBV in situ hybridization was negative. Submandibular salivary gland biopsy was performed and showed a similar process with infiltration of activated lymphocytes. The interstitium was involved by immunoblasts and prominent karyorrhectic activity. The apoptosis and inflammation within the salivary gland represent features of KFD.

Taken together, the findings are of necrotizing lymphadenitis with a marked paucity of granulocytes. a pattern keeping with KFD in the lymph node and the salivary gland. The relative paucity of plasma cells and the absence of vasculitis with DNA encrustation (Azzopardi phenomenon) make KFD more likely than a SLE.

**Conclusion:** This rare case report highlights the importance of considering KFD as part of the differential diagnosis in patients presenting with fever and lymphadenopathy, particularly in the presence of uncommon features of KFD such as salivary and parotid glands involvement. To the best of our knowledge, this is the first case of pediatric KFD with salivary and parotid gland involvement.

**Patient Consent**

Yes, I received consent

**Disclosure of Interest**

None Declared

### Miscellaneous rheumatic diseases

#### P306 Chikungunya fever presenting as lupus erythematosus-like syndrome : a case report

##### Najat Hijazi^1^, Judith Mura^2^, Araceli Centurion^1^, Cynthia Vega^3, 4^, Lorena Grau^5^, Jorge Lopez-Benitez^6, 7^, Claudia Flecha^1^, Natalia Cabrera^1, 4^ on behalf of Paediatric RheumatologY Study Group (PY Study Group)

###### ^1^Paediatric Department, Instituto de Prevision Social; ^2^Sociedad Cientifica de Estudiantes de Medicina, Universidad Catolica 'Nuestra Señora de la Asunciòn"; ^3^Paediatric Rheumatology, Hospital General Pediatrico 'Niños de Acosta Ñu' - Ministerio de Salud Publica y Bienestar Social; ^4^Sociedad Paraguaya de Reumatologia; ^5^Epidemiology, Instituto de Prevision Social; ^6^Paediatric, Centro Médico La Costa; ^7^Hemato Oncología Pediátrica (HOPe), Facultad de Ciencias Médicas - Universidad Nacional de Asunción, Asunción , Paraguay

####### **Correspondence:** Natalia Cabrera

*Pediatric Rheumatology 2024*, **22(2)**: PReS24-ABS-1089

**Introduction:** Juvenile Systemic Lupus Erythematosus (jSLE) poses a diagnosis challenge and should be excluded also in the infectious context of epidemics, such as chikungunya fever (CHIK fever).

**Objectives:** Report the case of CHIK fever in a teenager with multiorgan affection, mimicking the clinical presentation of SLE syndrome

**Methods:** Case report

**Results:** In March 2023, a previously healthy 12-year-old girl in Paraguay was admitted to the ER during a CHIKV epidemic. She presented with a 7-day history of fever, generalized pain, tactile hyperesthesia, myalgia, and swelling in her lower extremities. Upon admission, her swelling had worsened, and she complained of abdominal pain.

Physical examination revealed tachycardia, elevated blood pressure (95th percentile), significant weight gain (dry weight: 58kg vs 61kg) in 24 h), a skin rash, and joint swelling, particularly in the hips and knees. Abdominal evaluation showed symmetrical distention with a small inflammation (4cc inthe right iliac region). Laboratory analysis showed hypoalbuminemia, proteinuria, leucocytosis, anemia, elevated CRP, and muscle enzyme levels. She received symptomatic treatment including parenteral albumin.

After two weeks, due to ongoing symptoms (fever, oedema, prostration with eschars on both heels, myalgia) and renal involvement, further tests were conducted revealing a biological inflammatory syndrome (with CRP and VSG positive), C4 consumed and a positive ANA test (1/160) with negative anti-DNA. The MRI showed mild diffuse symmetrical muscular edema involving all thigh muscle compartments, more pronounced distally and slightly favoring the left adductor magnus, with a partial subfascial pattern. Additionally, myocarditis was diagnosed based on elevated cardiac enzymes, minimal pericardial effusion, and suggestive EKG findings including regular sinus rhythm, narrow QRS, QS pattern in lead D2, and peaked T wave in precordial leads.

On the 14th day of illness, a positive CHIKV PCR result prompted the administration of immunoglobulin at a dosage of 1g/kg per day for two days due to the persistent significant inflammatory state. After one week of treatment, the patient was discharged. Presently, she remains in good general condition, with 12 months having passed since the onset of symptoms. Subsequent laboratory tests have shown normalization of C3 and C4 values, along with negative anti-DNA and ANA results.

**Conclusion:** This case posed challenges considering that, initially, the patient displayed pronounced symptoms, necessitating the exploration of multiple differential diagnoses including jSLE. Show the significance of carefully considering various diagnoses in patients presenting with symptoms resembling those of SLE in arbovirus outbreaks (1) and highlights a long-term follow up to ensure the patient does not indeed develop SLE (2).

**Date of birth::** janvier 16

**Patient Consent**

Yes, I received consent

**Disclosure of Interest**

None Declared

**Reference**

1. Torales M, CDC-MMWR, 2023, 2Rajadhyaksha, A. et al. Lupus, 2012

### Miscellaneous rheumatic diseases

#### P307 Lysinuric protein intolerance associated with sjogren’s syndrome

##### Miika Arvonen^1^, Terhi Remes-Pakarinen^1^, Eija Piippo-Savolainen^1, 2^, Liisa Kröger^1^, Harri Niinikoski^3, 4^

###### ^1^Department of Pediatrics, Kuopio University Hospital; ^2^Department of Pediatrics, University of Eastern Finland, Kuopio; ^3^Department of Pediatrics, Turku University Hospital, ^4^Institute of Biomedicine, University of Turku , Turku, Finland

####### **Correspondence:** Miika Arvonen

*Pediatric Rheumatology 2024*, **22(2)**: PReS24-ABS-1278

**Introduction:** A 6-year-old girl from North Karelia, Finland, presented at the pediatric rheumatology outpatient department in May 2021 with joint pains and morning stiffness. Clinical examination revealed mild wrist synovitis and hypoechoic areas in the salivary glands. ENA, ANA, and anti-SS-A (Ro) were positive. She had mild (stage 1) caries in primary molar teeth but no hyposalivation or hypolacrimia. Due to delayed growth, a thorough medical history was conducted, revealing an aversion to meat products and nausea after consuming cheese.

**Objectives:** Diagnostics, treatment, and follow-up.

**Results:** Abdominal ultrasound showed an echogenic liver, and liver biopsy revealed mild hepatitis and microvesicular steatosis affecting 60% of hepatocytes. In 24-hour urine amino acid analysis, elevated excretions of lysine, arginine, and ornithine were observed, along with low lysine and increased levels of glutamine and alanine in plasma, suggesting lysinuric protein intolerance and potential association with hyperammonaemia. Exome sequencing revealed homozygous Finnish founder mutation in SLC7A7c.895-2A>T, confirming the diagnosis of lysinuric protein intolerance (LPI). Due to LPI, she was initiated on citrulline to enhance urea cycle function and alleviate symptoms. Additionally, sodium benzoate and sodium phenylbutyrate were prescribed to reduce residual nitrogen load. Moderate protein restriction (1–1.5 g/kg/day in children and 0.8–1 g/kg/day in adults) was also started along with supplemental lysine as well as vitamin and mineral supplements. Because she suffered from joint pain drug treatment with hydroxychloroquine (5 mg/ kg/day) was initiated in May 2023. A year later she developed right parotid inflammation. In an ultrasound the salivary glands appeared structurally abnormal throughout.

**Conclusion:** Lysinuric protein intolerance (LPI) arises from mutations in the SLC7A7 gene, manifesting as a rare autosomal recessive metabolic disorder and is associated with autoantibodies (Chen 2022, Contreas 2021). As demonstrated in this patient, LPI may be linked with salivary gland inflammation and Sjögren’s syndrome during childhood.

**Patient Consent**

Yes, I received consent

**Disclosure of Interest**

None Declared

**References**

1. Chen, Yating, and Xiangrong Zheng. "Lysinuric protein intolerance associated systemic lupus erythematosus with new mutations in the SLC7A7 gene." Authorea Preprints (2022)
2. Contreras, Josefina Longeri, et al. "Immune dysregulation mimicking systemic lupus erythematosus in a patient with lysinuric protein intolerance: case report and review of the literature." Frontiers in Pediatrics 9 (2021): 673957.

### Miscellaneous rheumatic diseases

#### P308 An atypical presentation of sjögren's syndrome: hypokalemia and distal renal tubular acidosis – a clinical case

##### João Pedro Valente, André Salvada, Andreia F. Ribeiro, Andreia Martins, Vanda Bento, Marta Cabral

###### Serviço de Pediatria (Direção: Drª Helena Cristina Loureiro), Departamento da Criança e do Jovem, Unidade Local de Saúde Amadora/Sintra - Hospital Professor Doutor Fernando Fonseca, E.P.E, Lisbon, Portugal

####### **Correspondence:** Marta Cabral

*Pediatric Rheumatology 2024*, **22(2)**: PReS24-ABS-1446

**Introduction:** Sjögren's syndrome (SS) is rare in paediatric age, especially due to the difficulty in fully meeting the classification criteria of the ACR and EULAR, established for adulthood.

**Objectives:** Underscore the importance of considering SS in the differential diagnosis of paediatric patients presenting with atypical symptoms.

**Results:** A 13-year-old female, native of Guinea-Bissau, previously healthy with no relevant family history. Two weeks before admission, developed polydipsia, polyuria, sensation of dry mouth, anorexia, 13% weight loss and easy fatigue. She denied fever, dysuria, suprapubic pain, arthralgia, xerophthalmia, oral/genital ulcers or skin changes.

On complementary evaluation: hyperchloremic metabolic acidosis and hypokalemia on arterial blood gas (ABG) analysis: pH 7.243, HCO3 15.9mmol/L, K 2.59mmol/L, Cl 115mmol/L. Urine analysis: density 1.003, pH 7, K 7mmol/L, Potassium Ejection Fraction 30% (upper normal limit), UOsm/POsm 0.3 (markedly decreased). The diagnostic hypothesis of distal renal tubular acidosis type I was considered. Potassium replacement therapy was initiated intravenously and later with potassium citrate.

Etiological investigation identified: ANA 1/160, anti-SSA, SSB, and Ro-52 antibodies +++. ESR 142mm/h. Investigation for possible SS was initiated: Schirmer Test was negative, without keratoconjunctivitis sicca. Salivary glands ultrasound revealed slight bilateral parotid atrophy with heterogeneous echotexture. Discharged on day 18 with unaltered ABG analysis, normalization of diuresis and medicated with potassium citrate.

After discharge, a sublingual gland biopsy was performed: “extensive lymphocytic infiltrate with at least 10 lymphoid aggregates, some with germinal centers - Grade 4 in Chisholm-Mason classification”.

The patient met the classification criteria that allow the formal diagnosis of SS and started follow-up in Paediatric Nephrology and Rheumatology consultation. Started treatment with hydroxychloroquine and received prednisolone in the first 6 months.

By the end of the first year underwent a gradual weaning off potassium citrate, symptomatic improvement. Analytically: ESR 19mm and a balanced ABG.

**Conclusion:** Due to the rarity of diagnosing SS in paediatric patients, diagnosis and follow-up present a challenge. Faced with the initial presentation of extraglandular manifestations and electrolyte disturbances, a high index of suspicion is essential. Moving forward, continued research and awareness efforts are essential to improve early recognition, diagnosis, and treatment of SS in paediatric patients, ultimately leading to better outcomes and quality of life for affected individuals.

**Patient Consent**

Yes, I received consent

**Disclosure of Interest**

None Declared

### Miscellaneous rheumatic diseases

#### P309 Rare case of stickler syndrome associated with severe Juvenile arthritis and non-bacterial osteomyelitis

##### Valeria Matkava^1^, Irina Nikishina^1^, Aliya Arefieva^1^, Svetlana Arsenyeva^1^, Tatiana Markova^2^

###### ^1^Paediatric, V.A. Nasonova Research Institute of Rheumatology; ^2^Research Centre for Medical Genetics, Moscow, Russian Federation

####### **Correspondence:** Valeria Matkava

*Pediatric Rheumatology 2024*, **22(2)**: PReS24-ABS-1507

**Introduction:** Stickler syndrome (SS) is a genetic disorder of type II collagenopathies which connected with pathogenic variants in 6 different genes and may lead to severe disability due to multiple system involvement progressive. skeletal/joints disorderes, ophtalmological complications, hearing loss.

**Objectives:** To describe a clinical case of newly diagnosed SS which was recognized because of unusual course of Juvenile Idiopathic Arthritis (JIA).

**Methods: Case report:** Boy, 9 y.o. was admitted to our clinic with polyarticular course of JIA. Since 6 y.o. he had suffered from progressive disability due to limitation of motion in hip joints, knees and pain in lumbar spine. Instrumental findings revealed kyphoscoliosis, hypoplasia of the iliac bones, signs of thoracic and lumbar vertebrae destruction (platyspondylia and wedge-shaped deformation). Infectious and oncologic diseases were excluded. His mother has the diagnosis of ankylosis spondylitis with total hips replacements. HLA-B27 was negative in both.

**Results:** According to detailed examination we observed definite signs of multiple synovitis, active bilateral sacroiliitis and foci of bone destruction, regarded as Chronic non-bacterial osteomyelitis (CNO), osteonecrosis of acetabulum. But there were also many features unusual for JIA: valgus deformity of lower extremities, waddling type walking, short stature, strabismus, progressive myopia and conductive hearing loss., lytic lesions of thoracic and lumbar vertebrae. Genetic disorder suggested. Target sequencing confirmed heterozygous pathogenic variants in COL2A1 gene. Patient was treated by NSAIDs with mild positive effect. Given the ambiguous situation, we decided to prescribe Tofacitinib, avoiding the use of methotrexate and received very good response - relief of pain, increasing of motion in hips and knees.

**Conclusion:** In this case we came across with definite diagnosis of SS (COL2A1+) on the one hand and all the grounds to make a diagnosis of JIA associated with CNO on the other hand. We couldn`t say yet whether this is a case of a special inflammatory phenotype of SS or its combination with JIA. The universal anti-inflammatory effect of Janus kinase inhibitors Tofacitinib seems to be the preferred alternative. It allows us to hope for the prevention of joint destruction.

**Date of birth::** juin 08, Y

**Patient Consent**

Yes, I received consent

**Disclosure of Interest**

None Declared

### Miscellaneous rheumatic diseases

#### P310 Autoimmune thyroid disease in pediatric patients with rheumatological diseases from Colombia

##### Camilo A. Vargas-Rincon^1^, Clara Malagón^2^, Catalina Mosquera^3^

###### ^1^Pediatrics, Universidad del Valle, Cali; ^2^Reumaped; ^3^Pediatrics, Colsubsidio Clinic Childrens, Bogotá, Colombia

####### **Correspondence:** Camilo Andres Vargas-Rincon

*Pediatric Rheumatology 2024*, **22(2)**: PReS24-ABS-1532

**Introduction:** Autoimmune thyroid disease (ATD) is the most common organ-specific autoimmune pathology in the population. It is now the most common cause of goiter, with an estimated prevalence of 5%. The presence of ATD can be identified as a premonitory pathology for the development of other autoimmune diseases (AID), and its search is important, even in the absence of clinical symptoms. Studies in the pediatric population of the coexistence of ATD with other rheumatological diseases report a prevalence of 5-15%.

**Objectives:** The aim of this study was to establish the prevalence of ATD and thyroid function in a group of pediatric patients with rheumatic diseases. Establish the prevalence of familial autoimmunity in this group.

**Methods:** A prospective multicenter study in which patients were included from three pediatric rheumatology centers in Bogotá, Colombia was performed. Antithyroid antibodies were carried out, and TSH and FT4 levels were determined. A record was made with clinical, paraclinical data and family history.

**Results:** We included 120 pediatric patients. ATD was identified in nine participants who had at least one positive antithyroid antibody, six had two antithyroid antibody positive. Two patients had hypothyroidism and subclinical hypothyroidism in 13. 100% of patients with autoimmune thyroiditis were women. Pathologies associated with ATD were UCTD, noninflammatory musculoskeletal pain and JDM each with two cases, SLE, Sjögren and JIA each with a case. The family history of AID was reported positive in 56.6% of patients and 16.6% had a second family member with AID. ATD was reported by 25.8% of patients, followed by rheumatoid arthritis 14.1%, SLE 8.3% and psoriasis 3.3%.

Anti-TG antibodies were positive at a level equal to or greater than 50IU/ml in 5.8% of patients, with a range of 58.6-2,535IU/ml. Anti-TPO antibodies, at levels of 100IU/ml or more were present in 6.6%, with a range of 115.4-771.5IU/ml. The highest titers of anti-TG were presented by a female patient with Sjögren's syndrome without a history of previous thyroid disease.

**Conclusion:** Monitoring of thyroid autoantibodies and thyroid function in patients with pediatric rheumatic disease, especially in women should be done periodically, even in the absence of symptoms of thyroid dysfunction. Autoimmune pathologies should be evaluated in symptomatic relatives of patients with ATD due to the frequency of familial autoimmunity. Generalized non-inflammatory musculoskeletal pain and fatigue during follow-up in pediatric rheumatology should be evaluated for ATD, as autoantibody positivity may be associated.

**Date of birth::** mai 09, YY

**Patient Consent**

Yes, I received consent

**Disclosure of Interest**

None Declared

**References**

1. Alvarez Madrid C, González Fernandez A, Lisbona Muñoz M, Molina Rodriguez MA, Merino Muñoz R, García-Consuegra Molina J. Thyroid disorders and childhood rheumatic diseases. An Pediatr (Barc) 2009;70:53-56.
2. PohjankoskiH, KautiainenH, KotaniemiK, KorppiM, SavolainenA. Autoimmune diseases in children with juvenile idiopathic arthritis. Scand. J Rheumatol 2010;39:435-436.

### Miscellaneous rheumatic diseases

#### P311 Atypical case of camptodactyly-arthropathy-coxa vara-pericarditis syndrome with high-titer anti-nuclear antibodies and elevated inflammatory markers

##### Ivanna Romankevych^1^, Jennifer Huggins^1, 2^, Ekemini Ogbu^1, 2, 3^

###### ^1^Rheumatology, Cincinnati Children's Hospital Medical Center; ^2^University of Cincinnati College of Medicine, Cincinnati; ^3^Johns Hopkins University, Maryland, United States

####### **Correspondence:** Ivanna Romankevych

*Pediatric Rheumatology 2024*, **22(2)**: PReS24-ABS-1591

**Introduction:** Camptodactyly-arthropathy-coxa vara-pericarditis syndrome (CACP) is a rare genetic condition characterized by development of progressive severe arthropathy, coxa vara and noninflammatory serositis from early childhood. It is often initially misdiagnosed as JIA. However, the synovial changes in CACP result from impaired synthesis of lubricin (proteoglycan 4, PRG4) the physiologic function of which is lubrication of the joint. The arthropathy in CACP is considered non-inflammatory and often does not improve with anti-inflammatory treatment or immune modulation.

**Objectives:** To share with pediatric rheumacology community our experience about unique case of CACP in 16-years old girl.

**Methods:** Case presentation

**Results:** We present the case of a 16-year-old Arab female who was referred to our clinic because of abnormal hand MRI suggesting severe chronic inflammatory arthropathy of both hands, and flexion deformities of all fingers. The initial signs of arthropathy were noted during her first 3 months of life. She had minimal joint pain complaints despite significant swelling of multiple joints. She had functional impairment from camptodactyly and chronic arthropathy of her wrists. She underwent tendon release surgery two times for trigger fingers before. There was a family history of consanguinity, autoimmune thyroiditis, Crohn’s disease, and similar arthropathy in her first-degree relatives. Laboratory results showed positive ANA 1:1280, elevated serum IgG 1.61 g/L, elevated ESR 29 mm/hr. However, RF, anti-CCP, HLA B27, dsDNA and ENA, and thyroid antibodies were all negative. Her pelvic radiograph demonstrated coxa vara and she had left sided pleural effusion on chest radiograph. Her left knee synovial biopsy showed synovial hyperplasia with no significant inflammatory changes. Combination of physical finding of severe arthropathy, autoimmune diseases in 1st degree relatives, and consanguinity between parent prompted us for additional genetic testing. Patient was positive for a mutation in the PRG4 gene consistent with CACP.

Due to elevation of inflammatory markers, she was started on a trial of steroids and hydroxychloroquine, and occupational therapy with improvement in her joint swelling, limitation, and her inflammatory markers over a 2-month period.

**Conclusion:** CACP is a rare condition and its diagnosis may be challenging, and it is often misdiagnosed with JIA. Development of progressive arthropathy from the first year of life should urge consideration of broader differential diagnosis and evaluation for monogenic arthropathies. Our patient has all typical presentation of CACP. However, she had elevated ANA and inflammatory markers pointed to possible overlapping autoimmune processes making her case unique. Her positive response to immune modulation raises consideration for individualized treatment and further studies of CACP.

**Patient Consent**

Yes, I received consent

**Disclosure of Interest**

None Declared

### Miscellaneous rheumatic diseases

#### P312 Exploring the connection: gender and vitamin d in adolescents with autoimmune diseases

##### María D. L. Aldana Galván, María M. Rodríguez Reyes, Luz D. C. Reynoso Medina, Ana V. Villarreal Treviño, Fernando García Rodríguez, Nadina E. Rubio Pérez

###### Pediatrics, Hospital Universitario Dr. José Eleuterio González, Monterrey, Mexico

####### **Correspondence:** María D. L. Aldana Galván

*Pediatric Rheumatology 2024*, **22(2)**: PReS24-ABS-1128

**Introduction:** Vitamin D, known for its roles in bone health and calcium regulation, affects the neurological, cardiovascular, insulin sensitivity, and immunological systems. A negative feedback mechanism involving PTH and FGF-23 controls its activity. Gender differences may influence vitamin D gene expression, affecting supplement response and health.^1^

**Objectives:** Determine if there is a relationship between gender and vitamin D insufficiency in patients with autoimmune diseases. Analyze if gender is related to disease activity and vitamin D insufficiency in patients diagnosed with juvenile idiopathic arthritis.

**Methods:** The study, conducted at José E. González University Hospital's Pediatrics Department, employed a retrospective, cross-sectional design. It involved patients aged 10 to 17 years diagnosed with various autoimmune diseases from the pediatric rheumatology clinic, with their vitamin D levels measured within a year of diagnosis. Patients were categorized into groups based on their vitamin D levels: insufficiency (<30 ng/mL) and optimal levels (≥30 ng/mL). Vitamin D deficiency (<20 ng/mL) was evaluated descriptively. Patient data such as age, gender, diagnosis, and juvenile idiopathic arthritis (JIA) activity were assessed. Disease activity was classified using specific cutoffs for the cJADAS index:

- High disease activity: cJADAS value > 8.5 for polyarthritis and > 4 for oligoarthritis.

The study analyzed the association between gender, vitamin D insufficiency, and JIA severity using Odds Ratio and 95% confidence interval. Fisher's exact test and Mann-Whitney U test were employed for statistical analysis, both with a significance level of <0.05. IBM SPSS Statistics 21 was utilized for data analysis.

**Results:** The study included 36 patients, predominantly females (61%) with a median age of 12.5 years. It was observed that 8 patients (22%) had vitamin D deficiency (levels <20 ng/mL). Juvenile idiopathic arthritis was the most common diagnosis (19, 53%). Regarding disease activity in juvenile idiopathic arthritis, there were no significant differences between patients with vitamin D insufficiency and normal levels (p = 0.968, Mann-Whitney U test). Males showed lower odds of vitamin D insufficiency (OR = 0.38, 95% CI: 0.0949, 1.5181), but this trend did not reach statistical significance (p = 0.30). Additionally, no significant association was found between vitamin D insufficiency and disease activity in either gender group (p = 1 for females; p = 0.5 for males, Fisher's test).

**Conclusion:** No significant association was found between gender and vitamin D deficiency in adolescents with autoimmune diseases, although a potential protective role for male gender was suggested. Factors such as age, geography, hormones, sun exposure, diet, and lifestyle may influence vitamin D levels and should be considered in future research.

Limitations of the study include its cross-sectional design, retrospective data collection, and sample size. Future studies, including randomized clinical trials, are recommended to determine the optimal vitamin D supplementation dose and assess the influence of gender. These studies could impact clinical practice and the management of autoimmune diseases.

**Date of birth::** mars 01, Y

**Patient Consent**

Not applicable (there are no patient data)

**Disclosure of Interest**

None Declared

**Reference**

1. Wierzbicka A, Oczkowicz M. Sex differences in vitamin D metabolism, serum levels and action. [Internet]. 2022 [cited 2024 May 4]. Available from: doi: 10.1017/S0007114522000149.

### Miscellaneous rheumatic diseases

#### P313 Advances in pediatric rheumatic diseases and polyautoimmunity: exploring capillaroscopy and optical coherence tomography angiography – clinical case series

##### Rodica Eremciuc^1, 2^, Olga Gaidarji ^1^, Elena Nedealcova^1, 2^, Ninel Revenco^1, 2^

###### ^1^Pediatrics, State University of Medicine and Pharmacy "Nicolae Testemitanu"; ^2^Pediatric Clinic N1, Mother and Child Healthcare Institute, Chisinau, Moldova, Republic of

####### **Correspondence:** Olga Gaidarji

*Pediatric Rheumatology 2024*, **22(2)**: PReS24-ABS-1293

**Introduction:** Patients with pediatric disorders often experience decreased quality of life, heightened disability, and increased mortality when comorbid conditions, such as coexisting autoimmune diseases, are present. Overt polyautoimmunity, characterized by the clinical manifestation of multiple well-defined autoimmune diseases in a single patient, exacerbates these challenges.

**Objectives: The study** aims to examine a series of clinical cases involving various autoimmune disorders in children, among which includes a connective tissue disease.

**Methods:** We compare our personal data of three clinical cases. Additionaly, we compares our data with similar case series, studies and meta-analysis from medical databases (Pubmed, Cochrane, Web of Science, SciELO). The following terms were used to generate a search: autoimmune disorders, polyautoimmunity, pediatric rheumatology, JIA, SLE, DMJ, psoriasis, antinucleary antibodies, children. The inclusion criteria were studies investigating autoimmune comorbidities in children with known pediatric rheumatic disorder.

**Results:** There are differences in the epidemiology and clinical course of ADs among pediatric and adult groups. In pediatric populations, the most frequent ADs are juvenile idiopathic arthritis, SLE, autoimmune myopathies, and scleroderma.

Our cases represent overlap syndromes, where at list 1 disease is a connective tissue disease:

- Case 1 – adolescent girl, 13 y.o., diabetes mellitus type 1, further associated with juvenile dermatomyositis, than confirmed autoimmune thyroiditis.
- Case 2 - adolescent girl, 14 y.o., seronegative polyarthritis ANA positive JIA in overlap with type 1 diabetes, severe form, unbalanced, with hypoglycemia and hyperglycemia; clinical case peculiarity represented by severe ocular involvement confirmed through optical coherence tomography angiography.
- Case 3 – 8 y.o. girl with a known diagnosis of autoimmune thyroiditis manifested clinical picture of juvenile dermatomyositis on an euthyroid state; clinical case peculiarity represented by symmetrical proximal muscle weakness, presence of Gottron's sign, heliotrope rash, increase in muscle degradation enzymes, myopathic-type ENMG changes, ANA 1:100, anti mi-2 positive, scleroderma-type capillaroscopic pattern, all of those corresponding for a high disease activity (CMAS 15 pts out of 52)

It remains a challenging area because, autoimmune diseases develop over a long time, which at least spans over several years.

**Conclusion:** The co-existence of multiple autoimmune diseases in patients with rheumatic disorders may be explained by the involvement of common genes. Additional work in this area is warranted given the opportunity to start detailed genomic studies which will elucidate pathogenic mechanisms in autoimmunity.

**Date of birth::** juin 20, Y

**Patient Consent**

Yes, I received consent

**Disclosure of Interest**

None Declared

### Miscellaneous rheumatic diseases

#### P314 Severe costochondritis and vertigo - a post-infective phenomenon or a rare rheumatological condition: relapsing polychondritis

##### Marah Shaikh Yousef^1^, Karen Kelleher^1^, Sinead O’Riordan^2^, Aisling Lyons^2^, Aaron Stirling^3^, Orla G. Killeen^1^, Emma J. MacDermott^1^

###### ^1^National Centre for Paediatric Rheumatology, CHI-Crumlin, Dublin; ^2^Department of Paediatrics, University Hospital Galway, Galway; ^3^Department of Radiology, CHI-Crumlin, Dublin, Ireland

####### **Correspondence:** Marah Shaikh Yousef

*Pediatric Rheumatology 2024*, **22(2)**: PReS24-ABS-1417

**Introduction:** Costochondritis is a benign cause of chest pain; management is with nonsteroidal anti-inflammatories (NSAIDs).^1^ Severe costochondritis is characterized by severe recurrent chest pain unresponsive to NSAIDs.^1^ We note a case in a paediatric patient post covid- 19 who responded to colchicine.^1^ Other rare causes of severe costochondritis include relapsing Polychondritis (RP) an immune-mediated multisystem condition characterized by recurrent inflammation of cartilaginous structures; diagnosis is made as per McAdam’s criteria.^2,3^

**Objectives:** Here, we report the case of a 13-year-old boy who presented with a 2-month history of severe chest pain, preceded by a covid-19 infection a few months prior. He was afebrile and hemodynamically stable but looked pale, clammy, and unwell with focal parasternal tenderness on a background of previous presentations for which he was treated as pneumonia. During the admission, he developed new-onset vertigo and left-eye keratitis.

**Methods:** Initial laboratory work revealed elevated inflammatory markers. Blood cultures were negative. He was treated with regular analgesia and intravenous antibiotics. His chest x-ray was unremarkable, prompting further imaging. Computerized tomography of his chest revealed severe advanced costochondritis. Magnetic Resonance Imaging of the brain revealed bilateral mastoid effusions.

**Results:** Given his persistent symptoms and raised inflammatory markers, he was discussed with and transferred under the care of the Rheumatology team in a tertiary paediatric centre. Histological samples were considered but deferred given his clinical condition. Upon consideration and exclusion of multiple differential diagnoses, he was ultimately diagnosed with probable RP given the severe costochondritis, vestibular dysfunction, and ocular involvement and was managed with steroids and colchicine. His symptoms and inflammatory markers resolved promptly thereafter, except for his vestibular symptoms, which were slower to subside.

**Conclusion:** Severe costochondritis is rare and has been described recently with post-COVID-19 infection and with RP, both of which respond to corticosteroids.^1^ Long-term patient follow-up is required to assess for long-term complications and disease progression.

**Patient Consent**

Yes, I received consent

**Disclosure of Interest**

None Declared

**References**

1. Collins RA, Ray N, Ratheal K, Colon A. Severe post-COVID-19 costochondritis in children. InBaylor University Medical Center Proceedings 2022 Jan 2 (Vol. 35, No. 1, pp. 56-57). Taylor & Francis.
2. Herrera I, Mannoni A, Altman RD. Relapsing polychondritis: commentary. Reumatismo. 2002;54(4):301-6.
3. Belot A, Duquesne A, Job-Deslandre C, Costedoat-Chalumeau N, Boudjemaa S, Wechsler B, Cochat P, Piette JC, Cimaz R. Pediatric-onset relapsing polychondritis: case series and systematic review. The Journal of pediatrics. 2010 Mar 1;156(3):484-9.

### Miscellaneous rheumatic diseases

#### P315 Early onset of multiple sclerosis in pediatric age: a case report

##### Ana Recalde^1, 2^ on behalf of Club de Redacción Científica, Sebastián Martin Etchegaray^3, 4^, Judith Mura^3, 4^, Natalia Cabrera^2, 5^, Cynthia Vega^6^, Alcy Torres^7^, Bruno Domínguez^7^, Natalia Irala^7^, Jorge López-Benítez^7, 8, 9, 10^

###### ^1^Pathophysiology, Catholic University "Nuestra Señora de la Asunción"; ^2^Pediatric Rheumatology, Club de Redacción Científica; ^3^Catholic University "Nuestra Señora de la Asunción"; ^4^Club de Redacción Científica; ^5^Pediatric Rheumatology, Instituto de Previsión Social; ^6^Pediatric Rheumatology, Hospital General Pediátrico "Niños de Acosta Ñú"; ^7^Pediatric Rheumatology, Medical Center La Costa, Asunción, Paraguay; ^8^Neurology, Boston Medical Center, Boston, United States; ^9^Pediatric Rheumatology, Paraguayan Society of Rheumatology; ^10^Pediatric Rheumatology, Pediatric Study Group, Asunción, Paraguay

####### **Correspondence:** Sebastián Martin Etchegaray

*Pediatric Rheumatology 2024*, **22(2)**: PReS24-ABS-1433

**Introduction:** Multiple Sclerosis (MS) is the most common non-traumatic disabling disease that affects young adults. The onset age typically ranges from 20 to 40 years old; however, cases have been reported outside of this range (1).

**Objectives:** Describe an unusual case of early-onset multiple sclerosis in a 2-year old girl, outlining the diagnostic process and discussing the clinical and imaging features that led to the identification of the disease.

**Methods:** Case report

**Results:** A previously healthy two-year old girl first presented to the Pediatric Service in 2014 complaining of hemiparesis and tremors on the left leg that gradually worsened. A hip Magnetic Resonance Imaging (MRI) revealed a small hypersignal lesion of 2.5 mm in diameter, adjacent to the surface cartilage of the femoral head. A cranial MRI revealed multiple lesions with hypersignal on T2 and hyposignal on T1, these lesions were found in various locations: the middle cerebellar peduncles on both sides, white matter of the supratentorial region, white matter of the lenticular nuclei on the left, the white matter adjacent to the left occipital horn and the hypothalamic area; also a lesion on the right side of the thalamus with a faint hypersignal in T2, without translation on T1 and a slightly positive on diffusion mapping, though not on primary sequences. After ruling out other diseases, and with the pattern of the MRI images consistent with MS, pulses of Methylprednisolone were started at 30 mg/kg/dose per day for a total of three days. Esteroides were started with methylprednisolone pulses and wean it down thereafter in 45 days.

In 2015, the patient was no longer on any medication but showed improvement, a subsequent cranial MRI was performed and showed the remaining of some active lesions. By 2016, the parents noticed slurred speaking patterns marked by a tongue draggin, and excessive salivation. A cranial MRI revealed a new lesion on the left temporal region. Three months later another cranial MRI was conducted, this time revealing new lesions in supratentorial white matter, the general aspect of the lesions as well as the distribution, suggesting demyelinating lesions. Other demyelinating diseases were ruled out based on clinical presentation and the absence of antibodies targeting the water-soluble aquaporin (NMO) channel. In response to the relapse symptoms Avonex (interferon beta 0.5 ml or 30 mg subcutaneously every week) was started and she is still with that drug, with a favorable response observed thus far.

**Conclusion:** This case demonstrates the importance of careful consideration of signs and symptoms that indicate MS, even though it is not common among children.

**Date of birth::** octobre 22

**Patient Consent**

Yes, I received consent

**Disclosure of Interest**

None Declared

**Reference**

1. Browne P, Chandraratna D, Angood C, Tremlett H, Baker C, Taylor BV, Thompson AJ. Atlas of multiple sclerosis 2013: a growing global problem with widespread inequity. Neurology. 2014;83(11):1022–1024. doi: 10.1212/WNL.0000000000000768.

### New diseases

#### P316 Spectrum of chaple disease in children

##### Sushma Shree B C ^1^, Lavenya R. padmanaban^2^, Prashant Bachina^3^, Chandrika S. Bhat^1^, Ryan S. Kaiser^3^

###### ^1^Paediatric Rheumatology; ^2^Paediatric Gastroenterology, Rainbow Children's Hospital, Bengaluru; ^3^Paediatric Gastroenterology, Rainbow Children's Hospital, Hyderabad, India

####### **Correspondence:** Sushma Shree B C

*Pediatric Rheumatology 2024*, **22(2)**: PReS24-ABS-1387

**Introduction:** CHAPLE (Complement hyperactivation, angiopathic thrombosis and protein losing enteropathy) disease is a recently described condition with pathogenic cross-activation of the complement system, predisposing individuals to enteropathy, severe thrombosis, with novel therapeutic agents involved in the management of this rare condition^1,2^.

**Objectives:** To describe clinical profile of CHAPLE disease with an aim to delineate the symptom onset and spectrum, serological and imaging features, as well as response to therapy in a resource limited setting.

**Methods:** We studied the clinical profile of 3 patients with abdominal pain and diarrhea caused by early-onset protein-losing enteropathy, edema due to hypoproteinemia, malabsorption and 1 had angiopathic thrombotic disease as well; the disorder followed an autosomal recessive pattern of inheritance. Whole-exome sequencing was performed to identify gene variants.

**Results:** History of consanguinity of parents was present in all families, age at manifestation varied from 7 months to 18 months, thrombotic complication was present in 1 child (hepatic venous thrombosis), normal inflammatory markers seen in 2, one had classical granulomatous inflammation of Crohns disease, non-specific inflammation on histopathology in distal colon was noted among the rest. 1 child started on Pozelimab responded well and the other 2 children await therapy. Until definitive therapy was initiated these children were predominantly maintained on low dose steroids and replacement immunoglobulin.

**Conclusion:** Monogenic inflammatory bowel disease must be considered in early onset symptoms of protein losing enteropathy. CHAPLE disease must be a differential especially when there is poor response to treatment or complication of thrombosis.

**Date of birth::** novembre 2

**Patient Consent**

Yes, I received consent

**Disclosure of Interest**

None Declared

**References**

1. Ozen A, Comrie WA, Ardy RC et al, Early-Onset Protein-Losing Enteropathy, and Thrombosis. N Engl J Med. 2017 Jul 6;377(1):52-61.
2. Ozen A. CHAPLE syndrome uncovers the primary role of complement in a familial form of Waldmann's disease. Immunol Rev. 2019 Jan;287(1):20-32.
3. Yu, CY., Ardoin, S.P. Complement inhibitor for therapy of CHAPLE. *Nat Immunol*22, 106–108 (2021).

### New diseases

#### P317 Clinical presentations and management of patients diagnosed with haploinsufficiency of A20: case series

##### Gülşah Kavrul Kayaalp^1^, Seher Şener^2^, Özlem Akgün^1^, Naile Kaya Yıldırım^3^, Metin Kaya Gürgöze^3^, Nuray Aktay Ayaz^1^

###### ^1^Department of Pediatric Rheumatology, Istanbul University, Istanbul Faculty of Medicine, Istanbul, ^2^Department of Pediatric Rheumatology, Adana City Research and Training Hospital, Adana, ^3^Department of Pediatric Rheumatology, Fırat University Faculty of Medicine, Elazığ, Türkiye

####### **Correspondence:** Gülşah Kavrul Kayaalp

*Pediatric Rheumatology 2024*, **22(2)**: PReS24-ABS-1161

**Introduction:** Haploinsufficiency of A20 (HA20), a newly recognized disorder arising from autosomal dominant loss-of-function mutations in the tumor necrosis factor alpha-induced protein 3 (TNFAIP3) gene, can manifest in a spectrum of clinical presentations ranging from recurrent fever, oral and genital ulcers, arthritis, arthralgia, ocular inflammation, autoimmunity, or immunodeficiency.

**Objectives:** We aimed to present four cases diagnosed with HA20 presented with different clinical features.

**Methods:** Medical charts of patients followed up in three centres with a diagnosis of HA20 and who have mutations in the TNFAIP3 gene were retrospectively reviewed.

**Results: Case 1:** A 16-year-old female presented with recurrent oral and genital aphthae to the dermatology clinic. Initially diagnosed with Behçet's disease, treatment with colchicine proved ineffective, prompting referral to pediatric rheumatology. Recurrent fever, abdominal pain, diarrhea, erysipelas-like erythema, arthralgia, and arthritis attacks were reported. Prednisolone and azathioprine were initiated, but new-onset arthritis was detected. Genetic testing revealed a novel TNFAIP3 gene mutation (p.L83F), leading to HA20 diagnosis. Infliximab treatment resulted in significant clinical improvement.

**Case 2:** An 8-year-old male had recurrent fever and abdominal pain since age 2. Initially treated with colchicine for suspected FMF without improvement. At 7, he developed perianal ulcers, and colonoscopy revealed nodular appearance in the terminal ileum, suggestive of lymphoid hyperplasia, along with occasional aphthous ulcers. Genetic testing revealed a TNFAIP3 gene mutation (p.(Val5281lefsTer2)). Azathioprine was initiated with incomplete symptom improvement, prompting adalimumab treatment.

Case 3: A 7.5-year-old female patient presented to the rheumatology clinic with recurrent fever, abdominal pain, diarrhea, oral and genital aphthae, and perianal abscess since age 6, occurring in episodic attacks. Hypogammaglobulinemia and growth retardation were noted. Genetic analysis revealed MEFV gene heterozygosity for the E148Q variant. Due to atypical attacks and new episodes despite colchicine, further genetic analysis identified a TNFAIP3 gene mutation (c.728G>A, p.(Cys243Tyr)). Colchicine treatment continued due to positive response.

Case 4: A 3.5-year-old female patient presented with a history of diarrhea, perianal inflammation, and genital ulcers since birth, along with a previous diagnosis of chronic gastritis and duodenitis. Symptoms of arthralgia and rash emerged at 2.5 years of age, followed by recurrent fever, oral aphthae, and abdominal pain at 3.5 years of age. Growth retardation was evident. Genetic testing revealed a TNFAIP3 mutation. Treatment with colchicine yielded an adequate response, and the current therapy was maintained.

**Conclusion:** As our understanding of HA20 expands, diagnosis of the disease may become more straightforward. Clinical manifestations in HA20 patients vary widely. HA20 should be considered in patients with treatment-resistant or unexplained autoinflammatory symptoms.

**Date of birth::** octobre 27

**Patient Consent**

Yes, I received consent

**Disclosure of Interest**

None Declared

### New diseases

#### P318 Myhre syndrome associated with chronic pericarditis and severe hypogammaglobulinemia treated with inhibitor of interleukin-1

##### Iva Rukavina^1^, Dorotea Bartoniček^1^, Mandica Vidović^2^, Nevenka Cigrovski^1^, Marijan Frkovic^1^, Marija Jelušić^1^

###### ^1^Department of Paediatrics, University of Zagreb School of Medicine, University Hospital Centre Zagreb; ^2^Department of Paediatrics, University Hospital Centre Sestre Milosrdnice, Zagreb, Croatia

####### **Correspondence:** Iva Rukavina

*Pediatric Rheumatology 2024*, **22(2)**: PReS24-ABS-1665

**Introduction:** Myhre syndrome, a rare genetic disorder, results from heterozygous mutations in SMAD4 gene, a crucial mediator in the TGF-beta signaling pathway. It’s pathophysiology involves dysregulated TGF-beta signaling, leading to aberrant extracellular matrix deposition and tissue remodeling. Clinically, it presents with dysmorfic facial features, skeletal anomalies like stiff joints and short stature, arthropathy, muscular-appearing body build, scleroderma-like skin, abnormal wound healing, laryngotracheal stenosis, restrictive and obstructive respiratory disease, psychomotor impairment, delayed puberty, hearing loss and cardiovascular abnormalities including pericardial effusion. Severe constrictive pericarditis requiring pericardectomy has been reported while inflammatory markers are usually normal. Pericarditis and restrictive cardiomyopathy are associated with high mortality. Despite varied clinical phenotypes, all of them had significant cardiac and/or pulmonary pathology.

**Objectives:** Here we describe a 13-year old female with previous genetically confirmed Myhre syndrome.

**Results:** She presents with facial dysmorphism included mid-face hypoplasia, hypertelorism, short palpebral fissures, short philtrum, broad nasal bridge, prognathism, short stature, brachydactily and clinodactily, joint contractures, shortened Achilles tendons, mild intellectual disability, repetitive respiratory infections (obstructive bronhitis), severe hypogammaglobulinemia, hypoproteinemia and deafness. Over her extremities the skin was thickened and inelastic, resulting in a scleroderma-like appearance. She has also developed recurrent pericarditis at the age of 9, requiring long-term steroid and non-steroid treatment what led her to obesity. She has also became hypertensive with slightly increased glicated hemoglobin. Chronic pericarditis was treated with colhicine but she developed diarhea. Despite normal inflammatory markers, the patient has received interleukin-1 receptor antagonist treatment (anakinra). To the best of our knowledge, this is the second report of anakinra therapy trying to treat pericarditis in patients with Myhre syndrome. Due to hypogammaglobulinemmia, she received immunoglobulins as well.

**Conclusion:** Management of Myhre syndrome necessitates a comprehensive, multidisciplinary approach addressing medical, developmental and psychosocial aspects to optimize patient outcomes and quality of life. We should be especially careful with recurrent pericarditis as it can be life-threatening. The pathogenesis of it remains unclear but the good response to anakinra at patients with idiopathic recurrent pericarditis suggests an autoinflammatory nature and corticosteroid spearing treatment choice for Myhre syndrome patients with pericarditis.

**Date of birth::** 11/21/1980

**Patient Consent**

Yes, I received consent

**Disclosure of Interest**

None Declared

### New diseases

#### P319 When it’s not behçet’s: unraveling the mystery of recurrent oral and genital ulcers

##### Daniela Pestana ^1^, Ana Zagalo^1^, Marta Cabral^1^, João F. Neves^1^, Cynthia Pinheiro^1^, Beatriz Martins^1^, Vitória Cadete^1^, Sónia Fernandes^2^

###### ^1^Hospital da Criança e do Adolescente; ^2^Serviço de Dermatologia, Hospital da Luz Lisboa, Lisboa, Portugal

####### **Correspondence:** Marta Cabral

*Pediatric Rheumatology 2024*, **22(2)**: PReS24-ABS-1818

**Introduction:** Both skin and mucosal inflammation can be associated with conditions such as autoimmune diseases (SLE, Behçet’s disease), erythema multiforme (EM), Stevens-Johnson syndrome (SJS) or toxic epidermal necrolysis (TEN), each linked to different triggers. Recently, the recognition of Mycoplasma pneumoniae infections as a distinct cause has led to the identification of a unique clinical entity known as Mycoplasma-induced rash and mucositis (MIRM)/Reactive Infectious Mucocutaneous Eruption (RIME).

**Objectives:** We present a case of recurrent RIME, highlighting the challenges in its diagnosis and treatment options.

**Methods:** 13-year-old female adolescent with multiple episodes of recurrent oral and genital ulcers over the past eighteen months, the first one requiring hospitalization due to extensive oral and genital mucositis, leading to reduced oral intake. A diagnosis of MIRM was assumed (positive Mycoplasma pneumoniae IgG/IgM serology) and she was treated with IV methylprednisolone and azithromycin, with complete recovery after 3 weeks. During the next 6 months she had three mild new episodes of oral aphthosis, 3-5 days duration, with fast improvement with steroids. On the current episode she reported rhinorrhea and a productive cough over the past 7 days; four days after, aphtous lesions in the mouth, lips and peribuccal/labial edema were noted. She was seen by her pediatrician and prescribed oral steroids, without any improvement. Additionally, this time she developed bullous and edematous genital lesions, and she was hospitalized due to progression of the oral and genital lesions with increasing pain and refusal to eat. She reported no fever, eye redness, asthenia, fatigue, exanthema, arthralgia, myalgia, vaginal discharges, dysuria, headache, or other neurological symptoms. Laboratory results showed hemoglobin 15.4g/dl, leukocytes 21,700/ul, neutrophils 61%, ESR 9mm/h, CRP 1.34mg/dl. She was admitted and intravenous methylprednisolone 1mg/kg was started. She had no fever and was hemodynamically stable, yet there was a progressive and gradual worsening of oral and genital mucosal lesions, accompanied by bilateral non-exudative conjunctival hyperemia and nasal skin lesions. Observed by Ophthalmology, which ruled out episcleritis, scleritis or uveitis. A multidisciplinary approach (Dermatology, Gynecology) led to the application of local treatments and further investigation showed normal protein electrophoresis, immunoglobulins A, G, M, and E, C3, C4, CH50, as also negative anti-dsDNA antibody, ANA, anti-Sm and anti-RNP. Bacteriological and mycological exams of oral and genital lesions, and Mycoplasma pneumoniae and herpes virus 6/7 oral and genital polymerase chain reaction were negative. Mycoplasma pneumoniae IgG/IgM serologies were positive. Skin biopsy, HLA-B51, anti-BP180, anti-BP230, anti-desmoglein IgG/IgM are pending. A diagnosis of recurrent RIME was assumed and given the lack of improvement after 4 days of intravenous corticosteroids, it was decided to administer IVIG 2g/kg as an addition to the treatment.

**Results:** There was a clear improvement of the oral lesions, most of which are crusting (and bleeding), but less painful, and also the genital lesions, which are scarring.

**Conclusion:** Due to recurrent episodes of exuberant mucocutaneous inflammation, with oral and genital involvement, MIRM can be easily mistaken with autoimmune diseases, namely Behçet's disease. We aim to raise awareness about this entity and highlight the efficacy of IVIG in cases of treatment failure or lack of clinical improvement after steroids. Nevertheless, further research is required to determine effective prevention and treatment strategies for patients with recurrent or refractory MIRM.

**Patient Consent**

Yes, I received consent

**Disclosure of Interest**

None Declared

### Pain, fatigue, disease experience and quality of life

#### P321 Psychiatric disorders in patients with juvenile fibromyalgia syndrome

##### Elena Pescio^1^, Claudio Lavarello ^2^, Flavia Riccio^3^, Cristina Venturino^1^, Elisa De Grandis^4^, Lino Nobili^4, 5^, Anna B. Ronchetti^6^, Francesca Alessi^2^, Francesco Mori^2^, Daiana Mariano^2^, Marco Gattorno^2^, Clara Malattia^2, 5^

###### ^1^Psychology; ^2^Rheumatology and autoinflammatory diseases, IRCCS G. Gaslini, Genoa; ^3^Rheumatology, University of Campania “Luigi Vanvitelli”, Naples; ^4^Neuropsychiatry, IRCCS G. Gaslini; ^5^Department of Neurosciences, Rehabilitation, Ophthalmology, Genetic and Maternal Infantile Sciences (DINOGMI), University of Genoa; ^6^Physical Medicine and Rehabilitation, IRCCS G. Gaslini, Genoa, Italy

####### **Correspondence:** Claudio Lavarello

*Pediatric Rheumatology 2024*, **22(2)**: PReS24-ABS-1174

**Introduction:** Juvenile fibromyalgia syndrome (JFS) is a disabling condition characterized by musculoskeletal pain, fatigue, sleep disturbances, and cognitive impairment. Psychiatric disorders (PDs) are common in adults with fibromyalgia and are associated with greater physical impairment (1). Very little data are available on PDs in JFS.

**Objectives:** To investigate PDs in JFS and their impact on the global burden of the disease.

**Methods:** We included patients with JFS diagnosed according to the 2010 American College of Rheumatology (ACR) criteria who were visited at our center between June 2021 and June 2023. We collected clinical data, including psychiatric comorbidity, and ongoing treatment. Depressive symptoms were assessed using the Children Depression Inventory 2 (CDI-2) for patients aged 11 to 17 years and the Beck Depression Inventory (BDI-2) for patients older than 17 years. Anxiety symptoms were assessed using the Multidimensional Anxiety Scale for Children 2 (MASC-2). Suicidal ideation risk was assessed by responses to item 8 of the CDI-2 and item 9 of the BDI-2. Severity of symptoms, quality of life (QoL), and functional ability (FA) were assessed using the Juvenile Fibromyalgia Multidimensional Assessment Report.

**Results:** 58 patients (52 females) with a median age of 17.7 years (11.4-19.4) and a median age at onset of 13.6 years were included in this cross-sectional study. 31 of 58 patients (53.4%) had the following isolated or associated psychiatric comorbidities: depressive disorder (35.5%), social anxiety disorder (35.5%), feeding and eating disorder (25.8%), panic disorder (19.3%), generalized anxiety disorder (12.9%), bipolar disorder (0.06%), posttraumatic stress disorder (0.03%), schizophrenia (0.03%), and depersonalization-derealization disorder (0.03%). 17 of 58 JFS patients (29.3%) expressed suicidal ideation. 15/31 (48.4%) patients received psychotherapeutic intervention and 21 patients (36.2%) received pharmacological therapy (10 gabapentinoids, 5 selective serotonin reuptake inhibitors, 4 amitriptyline, and 2 duloxetine). Psychiatric comorbidity in JFS was significantly associated with impaired physical functioning (p=0.031) and fatigue (p=0.027).

**Conclusion:** This study reveals that PDs are common in JFS and provides important insights into the impact of PDs on relevant clinical domains of the disease, such as fatigue and disability. Elucidation of the influence of PDs on disease severity could provide insights for the development of more comprehensive and targeted therapeutic approaches.

**Patient Consent**

Yes, I received consent

**Disclosure of Interest**

None Declared

**Reference**

1. Cunningham NR, Tran ST, Lynch-Jordan AM, Ting TV, Sil S, Strotman D, Noll JG, Powers SW, Arnold LM, Kashikar-Zuck S. Psychiatric Disorders in Young Adults Diagnosed with Juvenile Fibromyalgia in Adolescence. J Rheumatol. 2015.

### Pain, fatigue, disease experience and quality of life

#### P322 A comparison of self-reports and parent-proxy reports of health-related quality of life measured with the disabkids chronic generic measure (DCGM-37) and the disabkids arthritis module (ARM) in icelandic children with JIA

##### Drífa B. Guðmundsdóttir ^1^, Solrun W. Kamban^1^, Zinajda A. Licina^1^, Bjorg W. Gudjonsdottir^2^, Audur Kristjánsdottir^2^, Svanhildur A. Oskarsdottir^2^, Judith A. Gudmundsdottir^1^ and A research team from the Department of Physical Therapy at the University of Iceland and the multidisciplinary team for JIA at the Children´s Clinical Center in the National Hospital of Iceland

###### ^1^Children´s Health Center, National hospital of Iceland; ^2^Department of Physical Therapy, University of Iceland, Reykjavik, Iceland

####### **Correspondence:** Drífa B. Guðmundsdóttir

*Pediatric Rheumatology 2024*, **22(2)**: PReS24-ABS-1615

**Introduction:** The health-related quality of life (HRQoL) of children and adolescents with Juvenile idiopathic arthritis (JIA) can be negatively impacted by the symptoms of the disease, including swollen joints and stiffness, acute and chronic pain, sleep problems, fatigue, and an increased risk for uveitis, as well as negative effects related to the medical treatment. Several measures of HRQoL exist, and consist of self-reports and parent-proxy ratings on the impact of chronic diseases on the child´s functioning in various areas of their lives, including biological, psychological and social domains. Certain discrepancies have been found between children´s self-reports and parent proxy reports of HRQoL, suggesting for example that parents of ill children tend to underestimate the child´s self-reported quality of life, particularly when it comes to emotional issues, rather than physical.

**Objectives:** The aim of the current study was to investigate whether Icelandic children and their parents differed in their evaluation of HRQoL, and if so whether the difference would be in accordance with prior research findings.

**Methods:** The DISABKIDS chronic generic measure (DCGM-37) and the DISABKIDS Arthritis module (ArM) were translated into Icelandic and their psychometrical qualities were investigated in a descriptive cross-sectional study. Participants were 28 parent-child dyads (response rate 58%). The children were ages 8-17 years (Mean 12.43, SD 3,05, Median 12.00) and 57% were girls. The majority (82%) lived in and around the capital of Iceland, while the remaining 18% lived within an hour's radius from the capital area. Participation involved an extra appointment at the clinic for juvenile rheumatic diseases, Landspitali, Reykjavik. The children underwent a medical examination by a pediatric rheumatologist, following which they and their parents were asked to rate the child´s HRQOL separately, by filling out DCGM-37 and ArM, on an office computer. The research was approved by the Scientific Board of Landspitali, the Data Protection Authority and the National Bioethics Committee (VSN-19-141).

**Results:** There was a high concordance between children´s and parent-proxy reports of HRQoL within all domains on the DCGM-37. The correlations were strongest within the physical domain and weakest within the emotional domain. On ArM, the concordance between children´s self-reports and parent-proxy reports regarding „Impact“ of the disease was strong (p < .01), whereas the correlation between the child-reported and parent-reported „Understanding“ did not reach significance (p = .057). Both the children and the parents reported that the child experienced only moderate understanding from their environment. A paired sample t-test showed no significant differences between children´s self-reports and parent-proxy reports on either the DCGM-37 nor the ArM.

**Conclusion:** The study did not find significant discrepancies between child and parent proxy ratings of HRQOL in Icelandic children with JIA and their parents regarding the impact of chronic disease in different domains on DCGM-37, nor the ArM.

**Patient Consent**

Yes, I received consent

**Disclosure of Interest**

None Declared

### Pain, fatigue, disease experience and quality of life

#### P323 Retrospective single-centre study of the characteristics, management and outcomes of pediatric complex regional pain syndrome

##### Erika Alboreto ^1^, Pasquale Cardellicchio^2^, Francesca Alessi^3^, Francesco Mori^3^, Flavia Riccio^4^, Daiana Mariano^1^, Claudio Lavarello^3^, Svetlana V. Kotzeva^5^, Marco Gattorno^3^, Anna B. Ronchetti^2^, Clara Malattia^1, 3^

###### ^1^Department of Neurosciences, Rehabilitation, Ophthalmology, Genetic and Maternal Infantile Sciences (DINOGMI), University of Genoa; ^2^Physical Medicine and Rehabilitation Unit; ^3^Pediatric Rheumatology and Autoinflammatory Unit, IRCCS Istituto Giannina Gaslini, Genoa; ^4^University of Campania "Luigi Vanvitelli", Naples; ^5^Pediatric Anesthesiology and Acute Pain Unit, IRCCS Istituto Giannina Gaslini, Genoa, Italy

####### **Correspondence:** Erika Alboreto

*Pediatric Rheumatology 2024*, **22(2)**: PReS24-ABS-1214

**Introduction:** Complex regional pain syndrome (CRPS) is a severe pain condition that causes functional impairment and impacts greatly the patients’ quality of life. Despite some progress in research, more studies are required to improve diagnosis, clarify the molecular mechanisms of the disease and identify predictors of outcome so that targeted treatments can be developed.

**Objectives:** To describe demographic, clinical features and management of a single-center cohort of patients with pediatric CRPS and identify potential predictors of disease course.

**Methods:** This is a retrospective cohort study of patients with pediatric CRPS seen at the study center over the past 10 years. Demographic, clinical, radiologic data and therapeutic strategies were compared between patients with a favorable outcome and those who experienced at least one flare during the course of the disease. Pearson χ2 test or Fisher exact test were used to compare continuous and categorical clinical data between groups. P values < 0.05 were considered statistically significant.

**Results:** Fifty-three patients were admitted to our institute with a diagnosis of CRPS. Twenty-nine of 53 patients (54.7%) fulfilled the Budapest criteria for the diagnosis of CRPS and were included in the present study. Twenty-three patients (79.3%) were females. The mean age at disease onset was 11.2 years (5.7-13.6 years), with a mean time from symptom onset to diagnosis of 78 days (30-275 days). Most cases (82.7%) had lower extremity involvement. Sixteen patients (55.1%) identified an inciting physical traumatic event. At disease onset, 26 patients (89,6%) received the following pharmacologic treatments: nonsteroidal anti-inflammatory drugs in 22 (84.6%), acetaminophen in 11 (42.3%), gabapentinoids in 10 (38.5%), neridronate in 6 (23%), and tricyclic antidepressants in 4 (15.3%). Prompt referral to physiotherapy was reported in 20 patients (68.9%). Follow-up data were available for 18 patients (mean follow-up 20 months), of which nine (50%) had a favorable course of the disease and the remaining 9 (50%) had a relapsing disease (5 had a single relapse, one had 2 episodes of relapses and the remaining 3 had >3 relapses). Psychiatric comorbidities including depression, generalized anxiety, eating disorders, bipolar disorders and suicidal ideation were diagnosed in seven patients (38.9%). Patients who relapsed had a mean time to diagnosis of 84.55 days compared to 57.77 days for patients with a favorable disease course. The percentage of patients treated with neridronate was significantly higher in patients experiencing complete and sustained resolution of symptoms compared to patients with relapsing disease (66.7% versus 22.3%). There were no differences between the groups in age, sex, duration of the first episode, pain intensity, radiological features and psychological symptoms.

**Conclusion:** Approximately half of the patients with suspected CRPS do not meet the Budapest criteria thus highlighting the need for diagnostic criteria appropriate for the pediatric age group. Shorter time to diagnosis and bisphosphonate therapy may predispose patients to more favorable outcomes. Longitudinal multicenter clinical trials are required to develop consensus guidelines for treatment, which should be based on an interdisciplinary approach due to the high frequency of psychiatric comorbidities.

**Patient Consent**

Yes, I received consent

**Disclosure of Interest**

None Declared

### Pain, fatigue, disease experience and quality of life

#### P324 Expanding the toolbox: a comprehensive review of questionnaire utilization in pediatric rheumatology

##### Aysenur Namli Seker^1^, Nilay Arman^2^

###### ^1^Department of Podology, Aydin Adnan Menderes University, Nazilli Health Services Vocational School, Aydin; ^2^Department of Physiotherapy and Rehabilitation, Istanbul University-Cerrahpasa, Faculty of Health Science, Istanbul, Türkiye

####### **Correspondence:** Aysenur Namli Seker

*Pediatric Rheumatology 2024*, **22(2)**: PReS24-ABS-1535

**Introduction:** Pediatric rheumatologic diseases can be assessed using various methods, including questionnaires that measure parameters like fatigue, pain, quality of life, functional capacity, and joint damage. Among these, the Childhood Health Assessment Questionnaire (CHAQ) and the Child Health Questionnaire (CHQ) are commonly used. However, a wider range of questionnaires also exists (1,2).

**Objectives:** The aim of the this review was to evaluate the use of less common questionnaires in pediatric rheumatologic diseases, beyond the frequently utilized CHAQ and CHQ.

**Methods:** The eligibility criteria for the studies included in this study were that they had to 1) be conducted on children and adolescents with a rheumatic disease (aged 0–18 years); 2) be randomized controlled trials (RCTs), a controlled study, or pre-post studies, or trial, or review, or clinical answers and 3) be published in the English language. An internet-based search of three databases- PubMed, PEDro, and CENTRAL-was conducted for studies. The following search terms were used Childhood Myositis Assessment Scale (CMAS), Juvenile Arthritis Damage Index (JADI), Juvenile Arthritis Functional Status Index (JAFSI), Juvenile Arthritis Functional Assessment Report (JAFAR), Pediatric Quality of Life Inventory- Multidimensional Fatigue Scale (PedsQL-MFS), Pediatric Quality-of-Life Inventory Arthritis Module (PedsQL) and Pediatric Gait Arms Legs and Spine (pGALS) for Juvenile Idiopathic Arthritis (JIA), Juvenile dermatomyositis/Juvenile systemic lupus/juvenile fibromyalgia/Juvenile scleroderma/Juvenile spondyloarthritis/inflammatory myopathy.

**Results:** Our search yielded numerous studies: 64 on CMAS, 139 on JADI, 89 on JAFSI, 565 on JAFAR, 40 on PedsQl-MFS, 15 on PedsQl, and 30 on pGALS. Notably, for CMAS, 21 studies were on patients with JIA and 5 with juvenile inflammatory myopathy. JADI was featured in 19 JIA studies, JAFSI and JAFAR each in 5 JIA studies, PedsQl-MFS in 4 studies (3 on JIA, 1 on juvenile dermatomyositis), and PedsQL in 8 studies (7 on JIA, 1 on juvenile fibromyalgia).

**Conclusion:** Questionnaires are extensively used in pediatric rheumatology, particularly among JIA patients. The review highlights the diverse array of questionnaires deployed and underscores the need for incorporating a broader spectrum of tools to enhance diagnostic and therapeutic strategies.

**Date of birth::** septembre

**Patient Consent**

Yes, I received consent

**Disclosure of Interest**

None Declared

**References**

1. Luca, N. J., & Feldman, B. M. (2014). Health outcomes of pediatric rheumatic diseases. *Best practice & research Clinical rheumatology*, *28*(2), 331-350.
2. Tucker, L. B. (2000). Outcome measures in childhood rheumatic diseases. *Current rheumatology reports*, *2*(4), 349-354.

### Pain, fatigue, disease experience and quality of life

#### P325 Efficacy and safety of acupuncture in juvenile fibromyalgia syndrome: results of a pilot study

##### Francesca Alessi^1^, Claudio Lavarello^1^, Brunella Gily^2^, Francesco Mori^1^, Erika Alboreto^3^, Flavia Riccio^4^, Daiana Mariano^3^, Federica Casabona^3^, Marco Gattorno^1^, Clara Malattia^1, 3^

###### ^1^Rheumatology and Autoinflammatory diseases Unit; ^2^IRCCS Giannina Gaslini, Anesthesia and Pain Therapy Unit; ^3^Department of Neurosciences, Rehabilitation, Ophthalmology, Genetic and Maternal Infantile Sciences (DINOGMI), University of Genoa, Genoa; ^4^University of Campania "Luigi Vanvitelli", Naples, Italy

####### **Correspondence:** Francesca Alessi

*Pediatric Rheumatology 2024*, **22(2)**: PReS24-ABS-1393

**Introduction:** Juvenile fibromyalgia syndrome (JFS) is a chronic disabling condition characterized by widespread non-inflammatory musculoskeletal pain, fatigue, sleep and mood disorders. Management of JFS requires a multidisciplinary approach with a combination of non-pharmacological and pharmacological treatment modalities. Systematic reviews suggest that acupuncture may reduce pain and improve well-being in adults with fibromyalgia (1). No data are available for JFS.

**Objectives:** To investigate the efficacy and safety of acupuncture in patients with JFS.

**Methods:** We retrospectively analysed data of patients with JFS diagnosed according to the 2010 American College of Rheumatology (ACR) criteria, who underwent a course of six weekly sessions of acupuncture treatment in our institute during the past 2 years, due to significant refractoriness/intolerance to pharmacological therapy. Clinimetric assessment was based on multiple patient-reported outcomes (PROs), including: 1) 0-10 numeric rating scales (0=no symptoms, 10= great deal of symptoms) to measure the severity of musculoskeletal pain, fatigue, sleep quality, wake-up tiredness, headache, anxiety and depression, cognitive disturbances,abdominal pain and overall patient well-being); 2) Physical Function and Health-Related Quality of Life Questionnaire. Significant improvement, defined as a reduction of at least 30% in the PROs, was assessed at the end of the six-week treatment period.

**Results:** Ten female patients (median age 16.9 years, median disease duration 3.4 years) were included in this pilot study. Five patients were receiving pharmacological therapy (1 on pregabalin, 3 on amitriptyline, 1 on duloxetine) at stable doses for three months prior to treatment and during the study period. One patient discontinued treatment during the first session due to needle phobia. At the end of 6 weeks of acupuncture treatment, a significant reduction in the severity of musculoskeletal pain and headache was observed in 88.9% and 55.6% of patients, respectively. The median pain score was 8 (6-9) before treatment and significantly decreased to 2 (0-6.5), p=0.007, after treatment. Similarly, headache severity decreased from 6.5 (2-9.5) to 4 (0-7), p=0.01. Improvement in cognitive impairment was also seen in 33.3% of patients, with the median cognitive impairment score decreasing from 8 (7.5-9.5) to 6.5 (4-9), p=0.02. There was no significant improvement in other JFS-related symptoms, physical function, or health-related quality of life. Acupuncture was not associated with serious adverse events and had a reliable safety profile.

**Conclusion:** Acupuncture may be considered a potentially effective and safe therapeutic option for reducing musculoskeletal pain and headache in JFS. Our results suggest its use as part of a multimodal approach to the treatment of JFS. Further research is likely to provide data on effective regimens and combination therapies.

**Patient Consent**

Yes, I received consent

**Disclosure of Interest**

None Declared

**Reference**

1. Zheng C, Zhou T. Effect of Acupuncture on Pain, Fatigue, Sleep, Physical Function, Stiffness, Well-Being, and Safety in Fibromyalgia: A Systematic Review and Meta-Analysis. J Pain Res. 2022.

### Pain, fatigue, disease experience and quality of life

#### P326 Investigation of the relationship between disease activity, physical performance assessments, biopsychosocial characteristics, quality of life measurements in patients with juvenile idiopathic arthritis-pilot study

##### Sinan Buran ^1^, Orkun Tüfekçi^2^, Nur B. Karaca^2^, Emil Aliyev^3^, Yağmur Bayındır^3^, Yelda Bilginer^3^, Edibe Ünal^1^, Seza Özen^3^

###### ^1^Department of Heart and Respiratory Physiotherapy and Rehabilitation, Hacettepe University Faculty of Physical Therapy and Rehabilitation, ^2^Department of Basic Physiotherapy and Rehabilitation, Hacettepe University Institute of Health Sciences, ^3^Department of Pediatrics, Division of Rheumatology, Hacettepe University Faculty of Medicine, Ankara, Türkiye

####### **Correspondence:** Sinan Buran

*Pediatric Rheumatology 2024*, **22(2)**: PReS24-ABS-1558

**Introduction:** Juvenile Idiopathic Arthritis (JIA) is the most common childhood rheumatic disease characterized by disease activity and remission periods. Disease activity has a negative impact on the physical performance and psychosocial status of JIA patients (1,2).

**Objectives:** To investigate the relationship between disease activity, physical performance assessments, biopsychosocial (BPS) characteristics, and quality of life (QoL) measurements in patients with juvenile idiopathic arthritis.

**Methods:** Our study included sixty JIA patients (41 girls, 19 boys). Disease activity (JADAS-71), functionality (Childhood Health Assessment Questionaire (CHAQ)), BPS status (Juvenile Arthritis Biopsychosocial Questionnaire (JAB-Q)), QoL (Juvenile Arthritis Quality of Life Questionnaire(JAQQ)), functional capacity (Six Minute Walk Test (6MWT)), walking speed (10-meter walking test(10MWT)) and functional strength (Stair Climbing Test(SCT)) was assessed.

**Results:** The disease activity score (3.99±5.83) was low-moderate. A moderate level (0.40-0.69) significant correlation was found between disease activity and functionality, BPS status, and QoL in JIA patients (p<0.05*). A low level (0.21-0.40) significant correlation was found between disease activity and 6MWT predicted distance (%), 10MWT score (p<0.05*). There was no significant relationship between disease activity and the remaining measurements. A significant correlation was found between 10MWT score and SCT, 6MWT (rho= 0.587, rho= -0.544 respectively p<0.001*). A significant correlation was found between the 10MWT score and SCT, 6MWT (rho= 0.587, rho= -0.544 respectively, p<0.001*).

**Conclusion:** Disease activity impacted BPS status and QoL. The lack of a relationship between disease activity and functional tests may be due to the low-to-moderate level of disease activity in our study's sample. It was planned to investigate the determinants of these functional tests on disease activity and QoL by making the same assessments in patients with high disease activity.

**Date of birth::** avril 25,

**Patient Consent**

Not applicable (there are no patient data)

**Disclosure of Interest**

None Declared

**References**

1. Vincent HK, et al. Gait parameters, functional performance and physical activity in active and inactive Juvenile Idiopathic Arthritis. *Gait Posture*. 2022;98:226-232.
2. Şener S, et al. Comparison of Biopsychosocial Characteristics of Children with Juvenile Idiopathic Arthritis According to Common Disease Subtypes. *Turk Arch Pediatr*. 2023;58(6):625-630.

### Pain, fatigue, disease experience and quality of life

#### P327 Association of fatigue and disease activity in Juvenile idiopathic arthritis

##### María M. Rodríguez Reyes, Luz D. C. Reynoso Medina, María D. L. Aldana Galván, Ana V. Villareal Treviño, Fernando García Rodríguez, Nadina E. Rubio Pérez

###### Pediatric Rheumatology, Hospital Universitario "Dr José Eleuterio González", Monterrey, Mexico

####### **Correspondence:** Ana Victoria Villareal Treviño

*Pediatric Rheumatology 2024*, **22(2)**: PReS24-ABS-1351

**Introduction:** Juvenile idiopathic arthritis (JIA) is an idiopathic and chronic disorder that mainly affects the joints in patients under 16 years of age. On the other hand, fatigue is experienced as a chronic state of tiredness with fluctuations within the same day or between days. Processes such as inflammation and joint damage have been described to be related to fatigue; this has been described as a frequent symptom in rheumatic diseases, which may have association with disease activity in adults, however, this has not been described in pediatric patients.

**Objectives:** To evaluate the association of the scores obtained in the pediatric fatigue questionnaire in patients with juvenile idiopathic arthritis and the disease activity score.

**Methods:** Cross-sectional study. Patients under 16 years of age diagnosed with JIA who attended the outpatient clinic at the "Dr. José Eleuterio González" University Hospital during April 2024 were included. It considered scores on the fatigue scale greater than 60 points according to the standard deviation of the assessment instrument, and disease activity was classified using the JADAS score, and the Fisher's test was used to determine the significance of the study.

**Results:** Fifteen patients were included in the study, with a mean age of 10 years (minimum 3, maximum 15, IQR 5.5); of these, 60% were female. The patients were classified with polyarticular JIA (73.3%), systemic JIA (20%), and oligoarticular JIA (6.6%). Thirty-three percent (5) of patients presented both fatigue and an active disease, 6.6% (1) had no fatigue nor disease activity, 13% (2) did not report fatigue but had disease activity, and 46.6% (7) were in reimission and did not report fatigue. This differences were statistical significant (p = 0.026).

**Conclusion:** Fatigue was associated with disease activity in patients with juvenile idiopathic arthritis, and this association was statistically significant. Furthermore, it was observed that the majority of patients without fatigue also had disease inactivity. These findings underscore the importance of studying this association in a larger sample of patients. Additionally, they suggest the need to implement measures to reduce fatigue symptoms even during periods of disease inactivity, as fatigue seems to persist to some extent even when the disease is inactive.

**Date of birth::** septembre

**Patient Consent**

Not applicable (there are no patient data)

**Disclosure of Interest**

None Declared

### Pain, fatigue, disease experience and quality of life

#### P328 Narrative medicine intervention for mental wellbeing in Juvenile myositis and juvenile idiopathic arthritis

##### Aviya Lanis^1^, Emily Steelquist^2^, Christian Lood^3^, Susan Shenoi^1, 3^

###### ^1^Seattle Children's Hospital, Seattle; ^2^Oregon Health and Science University, Portland; ^3^University of Washington, Seattle, United States

####### **Correspondence:** Susan Shenoi

*Pediatric Rheumatology 2024*, **22(2)**: PReS24-ABS-1354

**Introduction:** Children with juvenile dermatomyositis (JDM) and juvenile idiopathic arthritis (JIA) have impaired quality of life and increased rates of anxiety and depression (15-65%), even in disease remission, as compared to healthy counterparts. Narrative medicine is a patient-targeted group-based intervention that allows patients to reconstruct their medical experiences through written or oral portrayals of emotions and self-reflective perspectives. It has demonstrated improved patient-reported outcomes with reduced rates of depression in adults.

**Objectives:** Recognizing there is limited data to date investigating utility of narrative medicine interventions for patients with rheumatologic conditions, this explorative study aims to assess feasibility of patient-targeted narrative medicine intervention and to understand the potential impact of the intervention on mental health burden.

**Methods:** This longitudinal cohort study prospectively recruited patients with established diagnoses of JDM from 6 to 21 years of age and age-matched patients with JIA from 6 to 21 years of age. Participants were divided by diagnosis into four groups, with 4-6 patients per session. Six narrative medicine-based sessions were held over 3 months, with each session including two narrative medicine-trained facilitators. Age-appropriate interventions including poetry, photography, and art were used to engage participants and prompt discussions around medical experiences. Demographic and medical information were collected by chart review, and patients completed pre- and post-participation questionnaires. Collected questionnaires included the Patient-Reported Outcomes Measurement Information System (PROMIS) Depression Scale, Generalized Anxiety Disorder-7 (GAD7), Childhood Attitude Towards Illness Scale (CATIS), Patient Health Questionnaire-8 (PHQ-8) and CoVID Stress Scale (CSSQ).

**Results:** Twelve patients with JDM and nine age-matched patients with JIA participated in the narrative medicine sessions. Forty-eight percent of patients participated in 6 sessions, 19 percent of patients participated in 5 sessions, 24 percent of patients participated in 4 sessions, and 9 percent of patients participated in 3 sessions. Wilcoxon signed-rank test revealed no statistically-significant difference between pre-participation and post-participation questionnaires for GAD-7, PHQ-8, PROMIS Depression, CSSQ, and CATIS scales.

**Conclusion:** It is feasible to implement a narrative medicine intervention for pediatric patients with JIA and JDM. Future studies should target larger populations to understand the impact of narrative medicine interventions on anxiety, depression and attitude towards illness.

**Patient Consent**

Yes, I received consent

**Disclosure of Interest**

None Declared

### Pain, fatigue, disease experience and quality of life

#### P329 Challenges and strategies in transitioning adolescents with systemic lupus erythematosus and juvenile idiopathic arthritis to adult healthcare: a cross-sectional study

##### Ana Sofia Figueiredo^1^, Sara G. Paulino^2^, Joana B. Monteiro^3^, Sara Ganhão^4^, Mariana Rodrigues^2, 3, 5^, Francisca Aguiar^3, 5^, Iva Brito^3, 5^

###### ^1^Pediatrics, ULS Trás-os-Montes e Alto Douro, Vila Real; ^2^Pediatrics, ULS São João; ^3^Faculty of Medicine, University of Porto; ^4^Pediatric and Young Adult Rheumatology Unit, ULS São João; ^5^Pediatric and Young Adult Rheumatology Unit, ULS São João, Porto, Portugal

####### **Correspondence:** Ana Sofia Figueiredo

*Pediatric Rheumatology 2024*, **22(2)**: PReS24-ABS-1189

**Introduction:** The shift from pediatric to adult healthcare is a critical phase for adolescents and young adults, characterized by various challenges, including the lack of established tools to evaluate patients' readiness for transition.

**Objectives:** Our study focused on assessing the transition readiness in adolescents and young adults (AYAs) with Systemic Lupus Erythematosus (SLE) and Juvenile idiopathic arthritis (JIA).

**Methods:** We conducted a monocentric cross-sectional study that included 33 patients with SLE and JIA between 14 and 26 years of age who attended outpatient pediatric and young adult rheumatology appointments in a Portuguese tertiary center, between October and December 2023. The patients were all diagnosed before reaching 18 years of age and had at least 1 year of follow-up. These patients completed surveys on demographics, medical history, and TRACS (Questionário de Preparação da Transição para a Autonomia nos Cuidados de Saúde) questionnaire – a validated Portuguese version of the Transition Readiness Assessment Questionnaire (TRAQ), with 4 sets of questions (managing medication, appointment keeping, tracking health issues, and talking with providers) and Hospital Anxiety and Depression Scale (HADS) questionnaire. Descriptive statistics, statistical comparisons and correlation tests were performed with SPSS version 29.0.0. A significance level of p < 0.05 was considered for statistical significance.

**Results:** Among the participants, 10 had SLE, mean age 20,3 ±3,4 years and mean disease duration of 5,8 ± 3,01 years. Twenty-three patients had JIA mean age 19 ±2,9 years and mean disease duration of 11,3 ± 5,3 years. The median TRACS score in SLE group was 4.62 [3.41-4.94] and 4.29 [2.82-4.82] in JIA. Notably, the TRACS score showed no significant correlation with age, socioeconomic status, education, or disease activity score. However, managing medication median score was significantly different between groups (p=0,009). JIA group had higher percentage of patients with active disease, but there was no difference between TRACs score and Hospital Anxiety and Depression Scale (HADS) among SLE and JIA groups. The JIA group had a significant moderate positive correlation between older age at diagnosis and higher tracking health issues score (p=0,033). Higher anxiety and depression questionnaires score had a significant moderate negative correlation with tracking health issues score (p=0,027 and p=0,045, respectively) and talking with providers (p=0,021 and p<0,001). In the SLE group, older age patients had significantly higher performance in talking with providers (p=0,026).

**Conclusion:** Our study sheds light on the challenges and differences in transition readiness between AYAs with SLE and JIA. Despite similar global TRACS scores, managing medication skills varied significantly between groups. Interestingly, older age at diagnosis in JIA and older age at the present in SLE patients were correlated with better tracking health issues and better communication skills, respectively. Additionally, anxiety and depression negatively impacted the ability to track health issues and communicate with providers, underscoring the importance of addressing mental health needs during transition planning. Our findings highlight the importance of tailored strategies addressing medication management and mental health support to facilitate successful transition for young rheumatology patients.

**Patient Consent**

Yes, I received consent

**Disclosure of Interest**

None Declared

### Pain, fatigue, disease experience and quality of life

#### P330 Impact of behçet's disease on the quality of life of pediatric patients and their parents

##### Nihal Sahin^1^, Yunus E. Bayrak^1^, Hafize E. Sönmez^1^, Betül Sözeri^2^

###### ^1^Pediatric Rheumatology , Kocaeli University, Kocaeli; ^2^Pediatric Rheumatology , Umraniye Research and Training Hospitale, İstanbul, Türkiye

####### **Correspondence:** Nihal Sahin

*Pediatric Rheumatology 2024*, **22(2)**: PReS24-ABS-1029

**Introduction:** Chronic diseases significantly affect the quality of life (QoL) of individuals. Behçet's Disease (BD), characterized by multisystemic vasculitis, particularly affects various vessels.

**Objectives:** This study aims to evaluate the QoL of pediatric BD patients and its correlation with their parents.

**Methods:** A cross-sectional study was conducted from June to December 2022, including pediatric BD patients meeting classification criteria. Clinical characteristics were recorded, and QoL was assessed using the Pediatric Quality of Life Inventory (PedsQL). Parents' QoL was evaluated using the World Health Organization Quality of Life-Bref (WHOQOL-Bref).

**Results:** The study included 38 patients (60.5% girls, 39.5% boys) with a median age of 15.5 years. All patients presented with oral aphthae, and additional mucocutaneous findings were observed. Patients were in remission under treatment. PedsQL scores for total, physical health, and psychosocial health revealed a median of 74.5, 76.5, and 75, respectively. Girls exhibited lower physical health scores than boys (p=0.004), and a negative correlation was found between disease duration and the total PedsQL score (r=-0.648, p=0.001). Parents' median WHOQOL score was 50, with 20 scoring below the cut-off value.

**Conclusion:** One-third of pediatric BD patients and over half of their parents experienced low QoL. Similar to other chronic illnesses, factors such as disease duration and gender were associated with QoL in pediatric BD. This research emphasizes the need for comprehensive care strategies to address the impact of BD on both pediatric patients and their parents.

**Date of birth::** 21.09.1987

**Patient Consent**

Yes, I received consent

**Disclosure of Interest**

None Declared

### Pain, fatigue, disease experience and quality of life

#### P331 Clinical profile and management of chronic musculoskeletal pain in children

##### Enas Alyaldin^1^, Junaid Ahmad^2^, Aashish Gupta^1^, Anurag Bharadwaj^3^

###### ^1^Paediatric Department, Mid and South Essex NHS Foundation Trust, Basildon; ^2^Paediatric Department, Mid and South Essex NHS Foundation Trust, Southend; ^3^Rheumatology Department, Mid and South Essex NHS Foundation Trust, Basildon, United Kingdom

####### **Correspondence:** Enas Alyaldin

*Pediatric Rheumatology 2024*, **22(2)**: PReS24-ABS-1136

**Introduction:** Chronic musculoskeletal pain in children is a common problem that impacts their physical, emotional, and social well-being. There is limited research and a lack of clear management pathways for chronic pain in children, which results in variation in management by health professionals. Multidisciplinary management, integrating pharmacological, physical, and psychosocial interventions, is crucial for optimal outcomes.

**Objectives:** To investigate the demographic profile, clinical features, and management strategies employed for children presenting with chronic musculoskeletal pain without any evidence of inflammatory arthritis.

**Methods:** The study involved a retrospective review of clinical notes for patients seen in paediatric rheumatology clinics, between January and December 2023 at Basildon University Hospital, Essex, UK. Our inclusion criteria were musculoskeletal pain lasting more than 3 months and/or musculoskeletal pain described as longstanding. Exclusion criteria were pain associated with inflammatory arthritis or other autoimmune conditions. In addition, the patients with a duration of pain less than 3 months were excluded. Our sample size was 51 patients selected as per our criteria after reviewing the case notes of 115 patients.

**Results:** The most common age of presentation was 15 and 16 years, constituting 47% of the 51 patients studied, with a notable female predominance (75%). Unspecified longstanding duration was reported in 49% of cases, while 31.3% had symptoms persisting for 1-2 years. Localised pain was noted in 41% of patients, whereas 56.8% presented with generalised pain. Clinic diagnoses given included terms like hypermobility spectrum disorder (29%), mechanical back pain (24%), chronic musculoskeletal pain (8%), and generalized pain (6%). No comorbidities were noted in 50.9% of patients, while 13.7% had Autism Spectrum Disorder (ASD) and Attention Deficit Hyperactivity Disorder (ADHD) and 1.9% suffered with anxiety/depression. 33% reported a family history of musculoskeletal pain conditions. Laboratory investigations revealed positive antibodies in 21.5% of cases: ANA (14%), RF (4%), ENA (4%), HLA B27 (6%), and Anti CCP (2%). Radiological investigations were conducted in 56.8% of patients. Treatment modalities included NSAIDs (21.5%), Amitriptyline (5.8%), and physiotherapy referral (84.3%). 51% were given a follow-up appointment.

**Conclusion:** In our study, children and young people with chronic musculoskeletal pain commonly presented around 15 to 16 years of age, with female predominance. A notable number had neurodevelopmental conditions such as ASD and ADHD, and mental health issues including anxiety and depression. One fifth of the patients had positive antibodies without any clinical evidence of inflammatory arthritis. Physiotherapy was the mainstay of management. Further research is warranted to explore underlying mechanisms and refine management strategies.

**Patient Consent**

Not applicable (there are no patient data)

**Disclosure of Interest**

None Declared

### Pain, fatigue, disease experience and quality of life

#### P332

##### **Patterns of disharmony; can the traditional tibetan system of the medical humours (nyespa) offer a systems approach to the delivery of non-pharmaceutical management of paediatric chronic pain?**

###### Jonathan Riemer

####### Paediatric & Adolescent Rheumatology, Royal Manchester Children's Hospital , Manchester, United Kingdom

*Pediatric Rheumatology 2024*, **22(2)**: PReS24-ABS-1124

**Introduction:** Medicine is increasingly complex, involving a highly connected system of people, resources, processes, and institutions [1]. A systems approach, a framework for seeing interrelationships rather than things, for seeing patterns rather than static snapshots [2] offers an opportunity for more dynamic delivery of healthcare, clinical efficacy and efficient use of healthcare resources.Traditional healthcare such as Tibetan medicine, that take systems approaches to ill health may offer an understanding for improving delivery of non-pharmaceutical management of paediatric chronic pain.Tibetan medicine has potential advantages of treating pain in how it is seen as an ontological reality [3]. Prevalence of paediatric chronic pain varies [4], uncertainty limits the appropriate allocation of clinical services [5] contributing to it's economic burden. A systems approach that directs non-pharmaceutical intervention to paediatric chronic pain, utilising understandings from Tibetan medicine, can both protect resources and establish clinical efficacy.

**Objectives:** This paper aims to expose opportunity of developing effective and efficient delivery of non-pharmaceutical management of paediatric chronic pain by presenting the traditional Tibetan medical system of *nyespa* in the context of paediatric chronic pain.

**Conclusion:** The Tibetan medical ontological understanding of pain and it's systems approach of *nyespa* imbalance is a framework for seeing interrelationships rather than things. The complexity of paediatric chronic pain with its multifactoral components demands multi-disciplinary non-pharmaceutical approaches. This leads to variable healthcare outcomes and costs. A systems approach developed from Tibetan medicine may offer an opportunity to improve clinical effectiveness and economic efficiencies, directing resources and interventions appropriately.

**Patient Consent**

Not applicable (there are no patient data)

**Disclosure of Interest**

None Declared

**References**

1. Clarkson J, Dean J, Ward J, Komashie A, Bashford T. A systems approach to healthcare: from thinking to -practice. Future Healthc J. 2018 Oct;5(3):151-155
2. INCOSEUK What is systems engineering? Creating successful systems. Z Guides, Issue 3.0 March; 2009. Available at http://incoseonline.org.uk/Documents/zGuides/Z1_What_is_SE.pdf [Accessed 13 September 2018]
3. Luo H, Zhong G, Yue L, Wang Q, Ma L, Luobu Z. Traditional Tibetan medicine in China: A systematic overview of randomized clinical trials. European Journal of Integrative Medicine. 2015 Oct 1;7(5):450-9
4. Tutelman PR, Langley CL, Chambers CT, Parker JA, Finley GA, Chapman D, Jones GT, Macfarlane GJ, Marianayagam J. Epidemiology of chronic pain in children and adolescents: a protocol for a systematic review update. BMJ open. 2021 Feb 1;11(2)
5. Murray CB, de la Vega R, Murphy LK, Kashikar-Zuck S, Palermo TM. The prevalence of chronic pain in young adults: a systematic review and meta-analysis. Pain. 2022 Sep 1

### Psycho-social aspects and rehabilitation

#### P333 Parental ratings of the general well-being of icelandic children with juvenile idiopathic arthritis compared with matched controls, perceived family support and health-care satisfaction

##### Solrun W. Kamban^1^, Zinajda A. Licina^1^, Judith A. Gudmundsdottir^1^, Erla K. Svavarsdottir^2^, Bjorg W. Gudjonsdottir^3^, Svanhildur A. Oskarsdottir^3^, Audur A. Kristjánsdottir^3^, Drífa Björk Guðmundsdóttir^1^

###### ^1^Children´s health center, National University Hospital of Iceland; ^2^Faculty of nursing; ^3^School of health sciences: Department of Physical Therapy, The University of Iceland, Reykjavik, Iceland

####### **Correspondence:** Solrun W. Kamban

*Pediatric Rheumatology 2024*, **22(2)**: PReS24-ABS-1778

**Introduction:** Families of children with Juvenile Idiopathic Arthritis (JIA) differ in their ability to cope with the daily challenges associated with the disease. International research suggests that the perceptions of health care professionals regarding many aspects of the disease differ from those of the patients and their families. Increased focus has been given to these different aspects by applying a multidisciplinary team approach in clinical practice. The pediatric rheumatology team at the National University Hospital in Iceland consists of a pediatric rheumatologist, pediatric nurse specialist, specialist in clinical psychology, and a social worker. The pediatric nurse specialist plays a central role in providing support in daily living during regular control visits, providing information about the disease, encouraging healthy lifestyle, empowering the child and the family, and attempting to reduce obstacles in daily life. Moreover, she mediates the contact with the pediatric psychologist and social worker based on the information acquired from the patient and parents.

**Objectives:** The aim of the current paper was twofold: (1) to investigate whether parents of children with JIA rated their children´s general well-being differently than parents of a control group of healthy children; (2) to investigate parental satisfaction with the provided health care, and the parents´ perception of family support provided by the multidisciplinary pediatric team for JIA.

**Methods:** The measures used for the study´s purposes were PedsQL^TM^General Well-Being Scale-parent proxy, PedsQL^TM^ -Health Care Satisfaction Generic Module, and The Iceland Family Perceived Support Questionnaire (Ice-PFSQ). Parents of 48 children and adolescents with JIA (8-18 years), registered in the medical record system of the National University Hospital of Iceland in the years 2016 to 2019, were invited to participate. Responses were acquired from parents of 25 children with JIA and 36 healthy controls, who were matched with the JIA sample in terms of gender and age. The study was a case-control study with cross-sectional design.

**Results:** The findings of the study indicate that the general well-being of Icelandic children with JIA is significantly poorer than that of children in the control group (M = 26.94, SD = 3,24 compared to M = 25.71, SD = 3.10 p < 0.05). In general, parents reported being very satisfied with the health care provided in different areas, with mean item scores ranging from 4.46 on the ‘Information’ subscale to 4.81 for the ‘General satisfaction’ subscale (where 5.0 was the maximum score). Parents were not equally satisfied with the perceived affective support provided by the multidisciplinary pediatric team (Item M = 3,30) as they were with the cognitive support (Item M = 4.17).

**Conclusion:** The role of the pediatric nurse specialist in the multidisciplinary team for JIA is very important. All patients and their parents meet the pediatric nurse specialist following the diagnoses. She provides them with support and encouragement, sense of security and information about the disease which lead to increased well- being, more perceived support and greater satisfaction with the health care system. Less satisfaction with affective support than cognitive support might indicate that there is room for improvement in that area and a more structured approach should be taken in evaluating the need for affective support.

**Patient Consent**

Yes, I received consent

**Disclosure of Interest**

None Declared

### Psycho-social aspects and rehabilitation

#### P334 Factors influencing physical activity of children and youth with Juvenile idiopathic arthritis and their peers

##### Marey Jonasdottir^1^, Drífa Björk Guðmundsdóttir^2^, Judith A. Gudmundsdottir^2^, Solrun W. Kamban^2^, Zinajda A. Licina^2^, Svanhildur A. Oskarsdottir^1^, Audur Kristjánsdottir^1^, Bjorg W. Gudjonsdottir^1^

###### ^1^Department of Physical Therapy, University of Iceland; ^2^Children´s Medical Center, National Hospital of Iceland, Reykjavik, Iceland

####### **Correspondence:** Drífa Björk Guðmundsdóttir

*Pediatric Rheumatology 2024*, **22(2)**: PReS24-ABS-1604

**Introduction:** Physical activity is important for normal growth, development and mental health. Research indicates that children and youth are not meeting the recommendations of the World Health Organization (WHO) on physical activity (PA) for children and young people aged 5-17 years. Various factors are believed to influence PA.

**Objectives:** The aim of this study is to examine the influencing factors of PA in children with juvenile idiopathic arthritis (JIA) and healthy peers.

**Methods:** The study was a descriptive cross-sectional study. The study participants were 62 children and youth aged 8-18 years, 27 children with JIA and 35 healthy peers. There was no significant difference in physical activity between the groups so the data of the two groups were pooled. Daily PA was objectively measured over seven consecutive days with an activPAL accelerometer. Dependent variables were daily step count and duration of time in moderate to vigorous physical activity (MVPA) and were used as measurements of PA. The children answered three questions about intensity of pain, prevalence, and location of pain over the past week when they returned the accelerometer. The results were analyzed using hierarchical multiple regression.

**Results:** Of the variables examined, only gender and age had a significant correlation with number of steps R^2^=0.215, (p<0.001). Higher step count was correlated with younger aged and/or male participants. Age was the only variable with a significant correlation with time in MVPA R^2^=0.107, (p<0.009). Younger aged participants spent more time in MVPA. The intensity of pain, prevalence and location of pain did not show significant correlations with either of the dependent variables.

**Conclusion:** Age has significant correlation for PA in the pooled group with respect to both the number of steps and the time spent in MVPA while sex only has significant correlation with the number of steps. Further research with a larger sample size and more independent variables is required.

**Patient Consent**

Yes, I received consent

**Disclosure of Interest**

None Declared

### Psycho-social aspects and rehabilitation

#### P335 Exploring the impact and lived experiences of symptomatic hypermobility in children, adolescents, and their parents

##### Susan Ward^1^, Emma J. MacDermott ^2^, Elizabeth Culleton-Quinn^1^, Jane Simmonds ^3^, Janet Deane^4^, Sara Dockrell^1^

###### ^1^Discipline of Physiotherapy , Trinity College Dublin; ^2^Rheumatology , Children's Health Ireland , Dublin , Ireland; ^3^Great Ormond Street Institute of Child Health, University College London, London; ^4^School of Sports, Exercise and Rehabilitation Sciences, University of Birmingham, Birmingham , United Kingdom

####### **Correspondence:** Susan Ward

*Pediatric Rheumatology 2024*, **22(2)**: PReS24-ABS-1782

**Introduction:** Children and adolescents with symptomatic hypermobility can present with chronic musculoskeletal pain and dysfunction, alongside a wide range of multi-systemic complaints (Castori, 2020). The lived experiences of children and adolescents with symptomatic hypermobility and the impact of symptoms on family life remain unexplored (Tran et al. 2020).

**Objectives:** To investigate the impact of symptomatic hypermobility on children, adolescents, and their families i) at home ii) at school iii) during activities and iv) with their peers. To explore if reported symptoms changed over time, as perceived by children, adolescents, and parents.

**Methods:** A qualitative study, using semi-structured online interviews was conducted. A reflexive process was used within a pragmatic ‘real world’ approach. Interviews were transcribed verbatim, member checked, and analysed using reflexive thematic analysis. The Consolidated Criteria for Reporting Qualitative Research (COREQ) Guidelines were followed (Tong et al. 2007). Participants were purposively sampled from a previous observational study conducted as part of a larger project.

**Results:** A total of n=18 children (n=6) and adolescents (n=12) and n=22 parents including mothers (n=18) and fathers (n=4) were included in the study. Interviews ranged in time from 30 minutes to 68 minutes in duration (mean time 45 minutes). Three themes were identified through thematic analysis 1) the need to make sense of symptoms, 2) the impact of symptoms on daily lives and 3) the challenges in care pathways. Children and adolescents expressed their uncertainty about symptoms. Impacts at home were significant, affecting everyday family activities. Pathways to care were often reported as complicated, with descriptions of challenging health care encounters, or difficulty implementing recommendations within school settings or amongst peers.

**Conclusion:** This study demonstrated for the first time, the impact of the uncertainty of symptomatic hypermobility on families. Participants expressed a need to make sense of their symptoms. Uncertainty of symptoms was further compounded by fluctuating symptoms that were difficult to comprehend and hard to explain to others, including other family members and peers. Wide ranging negative impacts on family life were identified. The impact on parental occupation, and potential loss of earnings due to shifts from full to part time were unique findings. Signposting is severely lacking for those on a symptomatic hypermobility ‘journey’. Development of improved care pathways would provide guidance to health care practitioners, families, and schools.

**Patient Consent**

Not applicable (there are no patient data)

**Disclosure of Interest**

None Declared

**References**

1. Castori M. Joint hypermobility in children: a neglected sign needing more attention. Minerva Pediatrica. 2020;72(2):123-33.
2. Tran ST, Jagpal A, Koven ML, Turek CE, Golden JS, Tinkle BT. Symptom complaints and impact on functioning in youth with hypermobile Ehlers--Danlos syndrome. Journal of Child Health Care. 2020;24(3):444-57.
3. Tong A SP, Craig J. Consolidated criteria for reporting qualitative research (COREQ): a 32-item checklist for interviews and focus groups. International Journal for Quality in Health Care. 2007;19(6):349-57.

### Psycho-social aspects and rehabilitation

#### P336 Sedentary behavior of children with Juvenile idiopathic arthritis

##### Birta Hafthorsdottir^1^, Drífa Björk Guðmundsdóttir^2^, Judith A. Gudmundsdottir^2^, Svanhildur A. Oskarsdottir^1^, Audur Kristjánsdottir^1^, Solrun W. Kamban^2^, Zinajda A. Licina^2^, Bjorg W. Gudjonsdottir^1^

###### ^1^Department of Physical Therapy, University of Iceland, ^2^Children´s Medical Center, National Hospital of Iceland, Reykjavik, Iceland

####### **Correspondence:** Drífa Björk Guðmundsdóttir

*Pediatric Rheumatology 2024*, **22(2)**: PReS24-ABS-1611

**Introduction:** High level of sedentary behaviour is negatively associated with good mental and physical health. It is also considered an independent risk factor for a range of diseases, especially cardiovascular diseases. In recent years, sedentary behaviour has increased significantly and research from other countries indicate that children with juvenile idiopathic arthritis (JIA) spend more time in sedentary behaviour compared to controls

**Objectives:** The aim of this study was to evaluate sedentary behaviour in children with JIA in Iceland.

**Methods:** A total of 63 children participated in the study, 28 children with JIA and 35 children in a control group. Sedentary behaviour was measured with an ActivPAL accelerometer which was attached on the participant’s thighs for seven days. Dependent variables were time spent in sitting and lying positions. A mixed model ANOVA was used for statistical analysis. Five analyses were performed for different types of sedentary behaviour: “total sedentary time”, “primary lying time” (sleep), “sitting time”, “transport time”, and “total sedentary time and primary lying time” combined.

**Results:** The groups were comparable in gender ratio, age, height**,** weight and BMI. There were no significant differences between groups in sitting time (p = 0.500), transport time (p = 0.054), total sedentary time (p= 0.585), primary lying time (p = 0.063) or combined total sedentary time and primary lying time (p = 0.132). There was a significant difference between weekdays and weekends for primary lying time and combined total sedentary time and primary lying time (p = < 0.001), where children in both groups spent more time sleeping on weekends than on weekdays.owever, no interactions were observed between groups and days of the week regarding sitting time (p = 0.067), transport time (p =0.544), total sedentary time (p = 0.284), primary lying time (p = 0.284) or combined total sedentary time and primary lying time (p = 0.751).

**Conclusion:** The results suggest that children with JIA in Iceland do not spend more time in sedentary behaviour of any kind compared to controls.

**Patient Consent**

Yes, I received consent

**Disclosure of Interest**

None Declared

### Psycho-social aspects and rehabilitation

#### P337 The effectiveness of the cognitive exercise therapy approach combined with biological treatment in a patient with chronic recurrent multifocal osteomyelitis: a case report

##### Sinan Buran ^1^, Orkun Tüfekçi^2^, Yağmur Bayındır^3^, Yelda Bilginer^3^, Edibe Ünal^1^, Seza Özen^3^

###### ^1^Department of Heart and Respiratory Physiotherapy and Rehabilitation, Hacettepe University Faculty of Physical Therapy and Rehabilitation; ^2^Department of Basic Physiotherapy and Rehabilitation, Hacettepe University Institute of Health Sciences; ^3^Department of Pediatrics, Division of Rheumatology, Hacettepe University Faculty of Medicine, Ankara, Türkiye

####### **Correspondence:** Sinan Buran

**Introduction:** Chronic recurrent multifocal osteomyelitis(CRMO) is a chronic inflammatory skeletal disease that predominantly affects children and adolescents (1). The literature has reported synergistic effects of the Cognitive Exercise Therapy Approach(BETY) combined with biological agent treatment in adult rheumatic diseases (2).

**Objectives:** To demonstrate the effectiveness of BETY combined with biological treatment on biopsychosocial (BPS) factors in a CRMO patient.

**Methods:** A case report

**Results:** An 11-year-old boy diagnosed with CRMO was referred to the Rheumatological Rehabilitation Unit from the Pediatric Rheumatology Department in December 2023. While switching from NSAIDs to biological agents as medical treatment, he was simultaneously included in the BETY program. In the first assessment, complaints of intense pain(Visual Analogue Scale(VAS): 7/10), pain catastrophizing(Pain Catastrophizing Scale-Child(PCS-C): 19/52), loss of functional abilities(Childhood Health Assessment Questionnaire(CHAQ): 1.75/3), BPS impairments(Juvenile Arthritis Biopsychosocial-Questionnaire(JAB-Q): 86/358) a decrease in quality of life(QoL)(Juvenile Arthritis Quality of Life Questionnaire(JAQQ): 3.6/7) were detected. Additionally, physical performance tests(Time-Up and Go Test(TUG): 9.54 sec, 10-meter walking test(10MWT): 7.79 sec, Stair Climbing Test(SCT): 6.26 sec, 30-second sit-to-stand test(30CST): 11) were applied. Six BETY-Function Oriented Core Stabilization Exercises specific to the patient's complaints were given after the assessment. Additionally, BETY-Nosiplastic Pain Management training was applied for his pain problem. Since the patient lives outside the city, the exercises were designed as a home program. The exercise compliance rate was evaluated on VAS in the second assessment, which was made in March 2024(VAS: 4/10). The second assessment results include pain(VAS: 0/10), pain catastrophizing(Pain Catastrophizing Scale-Child: 19/52), functional status(CHAQ: 0/3), BPS status(JAB-Q: 20/358), and QoL(JAQQ: 1.4/7). Dramatic improvements were observed in physical performance assessments(TUG: 7.28 sec, 10MWT: 5.33 sec, SCT: 3.43 sec, 30CST: 17).

**Conclusion:** With the positive results obtained in this case study, the effectiveness of individual BETY applied simultaneously with the biological agent on the biopsychosocial factors of the individual with CRMO was demonstrated. It was planned to increase the efficiency of the study by supporting it with a larger number of CRMO patients and control groups.

**Date of birth::** avril 25,

**Patient Consent**

Yes, I received consent

**Disclosure of Interest**

None Declared

**References**

1. Yousaf A et al. Chronic Recurrent Multifocal Osteomyelitis and Its Management. *Cureus*. 2021;13(10):e18872.
2. Karaca NB, et al. Effectiveness of a supervised group exercise therapy based on the biopsychosocial model introduced simultaneously with anti-TNF therapy in anti-TNF-naive patients with active ankylosing spondylitis. *Turk J Med Sci*. 2022;52(3):667-676.

### Psycho-social aspects and rehabilitation

#### P338 Physiotherapy and occupational therapy follow up after intra-articular steroid injection

##### Anna Haavisto Olow, Linn Hoel, Malin Robertsson, Marie-Louise Hilding

###### Paediatric occupational and physioterapy, Queen Silvia Children's Hospital, Gothenburg, Sweden

####### **Correspondence:** Anna Haavisto Olow

*Pediatric Rheumatology 2024*, **22(2)**: PReS24-ABS-1618

**Introduction:** Intra-articular corticosteroid injections (IAS) are an established method of treating active joint inflammation in juvenile idiopathic arthritis (JIA) (1, 2). At our clinic we routinely follow up the effect of the injections within 2-4 weeks post injection. Physiotherapists see the patients receiving injections in all joints except wrist/finger and temporomandibular joints. Occupational therapists follow up injections in wrist/finger joints. Previously we looked at the rate of follow up at the physiotherapy department after receiving an injection (3). We saw a 92% rate of follow up, with 53% on time in 2018. After that we experienced a pandemic which led to many cancelled care appointments due to restrictions.

**Objectives:** To compare the rate of follow ups after an intra-articular corticosteriod injection at the department of Occupational and physiotherapy after the covid-19 pandemic.

**Methods:** A retrospective survey of the patient registry and charts at the rheumatology clinic at Queen Silvia Children's Hospital, during January 1st - December 31st 2023.

**Results:** The survey shows that the need for IAS was lower in 2023 compared to 2018 (47 patients and 72 appointments for IAS vs. 63 patients and 93 appointments for IAS). We followed up the effects of the IAS in 96% of patients. One was not relevant for follow up due to medical conditions, another because they had their PT/OT contact at a local hospital. One received an injection in the TMJ. This year we have increased the rate of follow up to 96%, and of those 70% were followed up within 2-4 weeks.

**Conclusion:** We fulfill our commitment to follow up the effects of intra-articular corticosteroid injections within 2-4 weeks after the injection. Our team sees this as important as we can identify those in need of medical examination shortly to renew the IAS or adjust the medical treatment. We can also encourage our patients to resume physical activity and exercise as well as perscribre early specific exercises to minimise post arthritic sequele such as limited participation in daily activities, muscular hypotrophy or decreased range of motion.

**Patient Consent**

Not applicable (there are no patient data)

**Disclosure of Interest**

None Declared

**References**

1. Hagelberg S, Andersson-Gäre B, Fasth A, Månsson B, Enman Y, editors. Barnreumatologi. Lund: Studentlitteratur; 2007.
2. Gowdie PJ, Tse SM. Juvenile idiopathic arthritis. Pediatr Clin North Am. 2012;59(2):301-27. 10.1016/j.pcl.2012.03.014. PMID: 22560572.
3. Haavisto Olow A, Hoel L. Physiotherapy follow up within four weeks after intra-articular steroid injection. Proceedings of the 25th European Paediatric Rheumatology Congress (PReS 2018). *Pediatr Rheumatol* **16** (Suppl 2), 52 (2018). 10.1186/s12969-018-0265-6

### Psycho-social aspects and rehabilitation

#### P339 Psychiatric perspective on target-to-treatment strategies in rheumatologic diseases

##### Emil Aliyev^1^, Ecem Selin Akbas Aliyev^2^, Yelda Bilginer^1^, Seza Ozen^1^

###### ^1^Pediatric Rheumatology, Hacettepe University School of Medicine; ^2^Child and Adolescent Psychiatry, Republic of Türkiye, Ministry of Health, Etlik City Hospital and Research Center, Ankara, Türkiye

####### **Correspondence:** Emil Aliyev

*Pediatric Rheumatology 2024*, **22(2)**: PReS24-ABS-1018

**Introduction:** In recent years, treat-to-target (T2T) treatment approaches have gained importance in childhood rheumatologic diseases such as lupus and Familial Mediterranean Fever.

**Objectives:** We aim to apply psychiatric evaluations to all patients admitted to our outpatient clinic and provide recommendations by bringing this perspective to T2T.

**Methods:** All children and adolescents aged 8-18 years with a definite rheumatologic diagnosis were diagnosed at least three months before. Their parents, who applied to our hospital's Pediatric Rheumatology Outpatient Clinics, were included in our study. Between November 20, 2023, and December 29, 2023, data from 108 patients and their parents who met the above criteria were evaluated. Measurement tools were given to assess the depression and anxiety levels, quality of life and sleep disorders of the patients, psychiatric symptoms of their parents, as well as the level of family functioning. In addition, data such as the duration of chronic disease and disease activity of the patients were processed, and statistical correlations were analyzed.

**Results:** Of the patients, 63 (58.3%) were female and 65 (60.2%) were inactive/remission. There were significant differences in disease activity and patient's educational status, depression scale in children - generalized anxiety disorder, and family functioning. There were substantial differences between genders in all areas of the Child and Parent scales of the Quality of Life Scale (QoLS). The groups with vasculitis and autoimmune diseases had significantly lower Parent Stress Scale scores and QoLS Parent scores compared to the other groups. There was a strong correlation between the total score of Depression and Anxiety in Children and additional parental stress, anxiety, and depression. There was a significant and positive correlation between the duration of chronic illness and QoLS Parent, Beck Depression, and Illness perception.

**Conclusion:** In the evaluations made, the duration of chronic disease and disease activity status of the patients significantly affect the psychosocial profile of both patients and parents independently of the rheumatologic diagnosis. Therefore, patient and parent psychiatric evaluations and psychosocial interventions should be added to the targeted treatment strategies for rheumatologic diseases at regular intervals regardless of the diagnosis.

**Date of birth::** novembre 2

**Patient Consent**

Not applicable (there are no patient data)

**Disclosure of Interest**

None Declared

**References**

1. Rosina, S., Rebollo-Giménez, A. I., Consolaro, A., & Ravelli, A. (2023). Treat-to-target in pediatric rheumatic diseases. *Current Rheumatology Reports*, *25*(11), 226-235.
2. Parra Sánchez, A. R., Voskuyl, A. E., & van Vollenhoven, R. F. (2022). Treat-to-target in systemic lupus erythematosus: advancing towards its implementation. *Nature Reviews Rheumatology*, *18*(3), 146-157.
3. Maggio, M. C., & Corsello, G. (2020). FMF is not always “fever”: from clinical presentation to “treat to target”. *Italian Journal of Pediatrics*, *46*(1), 1-5.

### Psycho-social aspects and rehabilitation

#### P340 Evaluation of oral hygiene habits between rheumatic diseases patients and healthy children

##### Büşra Başer Taşkın^1^, Ayşenur Doğru^1^, Ahmet Taşkın^2^, Nuray Aktay Ayaz^1^

###### ^1^Pediatric Rheumatology, Istanbul University, Istanbul Facuty of Medicine; ^2^Pediatric Neurology, Istanbul Medipol University, Istanbul, Türkiye

####### **Correspondence:** Büşra Başer Taşkın

*Pediatric Rheumatology 2024*, **22(2)**: PReS24-ABS-1762

**Introduction:** Inflammation of the oral cavity can lead to systemic infections. Rheumatologic diseases and the medications used in their treatment can have adverse effects on oral health. Therefore, individuals with rheumatologic diseases should pay extra attention to regular dental check-ups, appropriate brushing, and oral hygiene routines.

**Objectives:** This study aims to evaluate the oral health and hygiene of patients with rheumatologic diseases

followed up in the pediatric rheumatology outpatient clinic.

**Methods:** A total of 292 children, comprising 144 with rheumatological diseases, and 148 healthy individuals, were included in the study, and a web-based survey study was conducted. The survey was divided into three sections: demographic information, oral hygiene, and education regarding oral hygiene. The questionnaire comprised of two open-ended questions and 17 multiple-choice questions. A comperative analysis was conducted between patients and healthy controls.

**Results:** A survey evaluating the oral hygiene habits of a total of 292 individuals was conducted. Among the respondents, 148 were healthy controls, and 144 were patients. The mean age of patients was 13 (min-max: 9-16), whereas in the healthy group, it was 12 (8.5-14). Eighty patients (55.6%) and 86 individuals (58.1%) in the healthy control group were female. When asked whether they had previously visited a dentist, 116 patients (80.6%) and 122 individuals (82.4%) in the control group answered affirmatively. The frequency of dental visits in both groups was most common between the ages of 5 and 9.

Regarding tooth brushing habits, it was observed that 74 patients (51.4%) and 68 individuals (45.9%) in the control group brushed their teeth twice a day. Although not statistically significant, this rate was higher in the patient group.

The percentage of participants brushing their teeth before bedtime was 75% among patients and 81% among healthy group. The most common duration of tooth brushing was 1-2 minutes (48% in the healthy group, 49% in the patient group). Manual toothbrushes are used by 93% of the patient and 76% of the healthy group, with a significant difference (p<0.001).

When questioned about their knowledge of toothpaste, 85 patients (59%) and 77 individuals (52%) in the healthy group did not know the contents of the toothpaste they used. Seventy-nine patients (55%) and 76 individuals (51.4%) in the healthy group were found to use fluoride-free toothpaste.

The most common source of education about oral hygiene was from their families (74% in the patient group, 61% in the healthy group). A total of 91 patients (63.2%) and 96 individuals (65%) in the control group stated that they had not attended any informative meetings on dental hygiene.

Seventy-three patients (50.7%) and 47 controls (31.8%) expressed a desire to learn more about oral health, with a significant difference between them (p=0.001).

**Conclusion:** In light of these results, it would be accurate to indicate that patients require further education regarding oral health. Organizing regular educational sessions and informative workshops for the patients can enhance awareness of oral hygiene and assist them in establishing a healthier oral care routine. Providing continuous support regarding the importance of daily oral health habits and correct oral hygiene practices can improve the health and quality of life of the patients.

**Date of birth::** juillet 12

**Patient Consent**

Yes, I received consent

**Disclosure of Interest**

None Declared

### Psycho-social aspects and rehabilitation

#### P341 Evaluation of hand hygiene practices in children with rheumatic diseases and comparison with healthy children

##### Bengisu Menentoğlu^1^, Aysenur Doğru^1^, Büşra Başer Taşkın^1^, Ahmet Taşkın^2^, Özlem Akgün^1^, Nuray Aktay Ayaz^1^

###### ^1^Pediatric Rheumatology, Istanbul University İstanbul Faculty of Medicine; ^2^Pediatric neurology, Istanbul Medipol University, İstanbul, Türkiye

####### **Correspondence:** Bengisu Menentoğlu

*Pediatric Rheumatology 2024*, **22(2)**: PReS24-ABS-1714

**Introduction:** Children with rheumatic diseases are mainly treated in outpatient clinics, so focusing on simple preventive measures like hand hygiene is more beneficial before considering complex interventions to prevent the spread of infectious diseases. Effective hand hygiene has long been recognized as crucial in infection prevention. Community hand hygiene initiatives have successfully reduced the incidence of infectious diseases, highlighting its importance in infection control. Factors such as time constraints, lack of training, and environmental conditions can affect adherence to hand hygiene practices. The importance of handwashing has been further emphasized globally following the emergence of SARSCoV2 after 2019, for both healthy individuals and those with chronic illnesses.

**Objectives:** In this study, we aimed to evaluate the handwashing habits of children with pediatric rheumatic diseases, the importance they attach to hand hygiene to prevent infections, and to reveal the alterations in hand hygiene practices after the SARSCov2 period.

**Methods:** The study included 144 patients followed up in the pediatric rheumatology outpatient clinic and 144 healthy children matched for age and gender. The survey included a variety of question types and divided responses into four main groups: demographic data, handwashing practices, self-assessment via 5-point Likert scale ranging from "strongly agree" to "strongly disagree", and the impact of the SARS-CoV-2 pandemic. Children with rheumatic diseases in the Pediatric Rheumatology Clinic voluntarily completed the survey in about 5 minutes. The survey ran for 14 weeks from December 14, 2023, to March 21, 2024, with each participant allowed to participate once.

**Results:** Females constituted 53,5 % (n:144) of the patient group and 59% of the control group. The Median age of the patient group was 13 (IQR 8-14) years, and the median age of the control group was 11 (IQR 8-14) years. In our study, the prevalent diagnoses in the patient group were FMF at 34% (n=49) and JIA at 25% (n=36). Compared to healthy children, it was observed that the number of daily hand washing (p < 0.001) and duration (p = 0.034) were significantly higher in the patient group. It has been determined that there was an increase in the frequency of handwashing in both groups following the COVID-19 pandemic.

**Conclusion:** It is imperative to emphasize the importance of personal hygiene measures in preventing infections. This is especially critical for children with chronic diseases, as infections can exacerbate their condition, and the medications administered for treatment can result in immunosuppression. Within our pediatric rheumatology outpatient clinic, we provide detailed guidance to patients and their parents on preventive hygiene protocols such as proper handwashing techniques. As a consequence, a greater incidence of handwashing was observed within our patient population.

**Date of birth::** novembre 0

**Patient Consent**

Yes, I received consent

**Disclosure of Interest**

None Declared

### Scleroderma and related syndromes

#### P342 What are the indications for juvenile-onset systemic sclerosis and autologous stem cell transplant. result of a multinational survey

##### Ivan Foeldvari^1^, Samantha Branton^2^, Suzanne Li^3^, Franziska Roser^2^, Kathryn Torok^2^

###### ^1^Hamburg Centre for Pediatric and Adolescence Rheumatology, Hamburg, Germany; ^2^University of Pittsburgh and University of Pittsburgh Medical Center Children’s Hospital of Pittsburgh , Pittsburgh; ^3^Hackensack University Medical Center , Hackensack , United States

####### **Correspondence:** Ivan Foeldvari

*Pediatric Rheumatology 2024*, **22(2)**: PReS24-ABS-1377

**Introduction:** Juvenile systemic sclerosis (jSSc) is an orphan disease. It is a life-threatening disease as in the adult form, but a with a lower morbidity. Hematopoietic Autologous Stem Cell Transplant (ASCT) seems to be a promising treatment option in adults. Currently there is no guidance regarding the indication of ASCT for jSSc, although ASCT has been successful as established therapeutic option in adult systemic sclerosis (aSSc) and seems to be promising for jSSc too.

**Objectives:** The purpose of this survey is to better understand if and for what indications and in which pediatric patient healthcare providers consider ASCT as a therapeutic option for jSSc.

**Methods:** An electronic Qualtrics survey with 43 questions regarding the indication for ASCT in jSSc war distributed by email to the members of the PRES and CARRA juvenile scleroderma working groups and for the contributing physicians of the juvenile scleroderma inception cohort. The first 6 questions of the survey assessed the characteristics of the participant and the next 37 questions assessed, in which indication and after which kind of treatment ASCT would be considered

**Results:** Out of the 42 trained pediatric rheumatologists were invited to complete the survey. 29 (69.0%) submitted a completed survey. Twenty-one participants (72%) said that they personally had recommended a patient for ASCT. When considering referral three criterion, severe impairment of rapid progression (26/89%), severe disease status (24/82%) and quality of life (24/82%), were agreed upon to be the most important factors. Majority of the experts agreed that at least 2-3 DMARD (16/55%) regimens should be attempted before consideration of ASCT, but 9/31% would opt for more than 3 DMARDs. A majority also agreed that failure of mycophenolate mofetil (25/86%)), cyclophosphamide (23/79%)), tocilizumab (22/75%), methotrexate (19/66%) and rituximab (18/62%) would be grounds for ASCT consideration. Regarding disease progression thirteen (45%) participants through that the 3-6 months was the most integral time, while 11 (38%) participants thought that the 6-12 months interval. Twenty-nice (100%) felt that pulmonary involvement should be grounds for ASCT, 27/93% indicated cardiac involvement, 21/72% gastrointestinal involvements, 19/66% skin involvement, 18/62% vascular involvement, and 8/27% musculoskeletal involvements

**Conclusion:** Experienced pediatric rheumatologist found that at least 2-3 DMARDs should be tried, to define nonresponse to DMARDs. The time frame of disease progression should be assessed in 3-12 months of the disease and the top 3 organ involvement was listed by importance pulmonary, cardiac and gastrointestinal. There is a need to find the trajectories, which predict, which patients should be assessed for ASCT, as Keret et al showed MMF and rituximab compared with AHSCT in SSc patients eligible for AHSCT resulted in similar skin and lung clinical improvement with a better safety profile at 24 months in adults

**Patient Consent**

Not applicable (there are no patient data)

**Disclosure of Interest**

None Declared

### Scleroderma and related syndromes

#### =343 Outcome measures for the assessment of cutaneous and musculoskeletal manifestations of juvenile systemic sclerosis: an international collaborative scoping review

##### Amanda D. Robinson^1^, Mustafa Çakan^2^, Susan Shenoi^3^, Simone Appenzeller^4^, Meiping Lu^5^, Betul Sozeri^6^, Rongjun Zheng^5^, Priya Bhave^7^, Natalia Vasquez-Canizares^8^, Suzanne C. Li^9^ and International Juvenile Systemic Sclerosis Outcomes Group

###### ^1^Pediatrics, Division of Pediatric Rheumatology, University of Utah, Salt Lake City, UT, United States; ^2^Pediatrics, Clinic of Pediatric Rheumatology, Zeynep Kamil Women and Children's Diseases Training and Research Hospital, İstanbul, Türkiye; ^3^Seattle Children’s Hospital and Research Center, University of Washington, Seattle, WA, United States; ^4^School of Medical Science, University of Campinas, Campinas, Brazil; ^5^Department of Rheumatology, Immunology and Allergy, Children’s Hospital, Zhejiang University School of Medicine, National Clinical Research Center for Child Health, Hangzhou, China; ^6^University of Health Sciences, Umraniye Training and Research Hospital, İstanbul, Türkiye; ^7^Hackensack Meridian School of Medicine, Hackensack, NJ; ^8^Pediatrics, Division of Pediatric Rheumatology, Children’s Hospital at Montefiore, Albert Einstein College of Medicine, Bronx, NY; ^9^Pediatrics, Division of Pediatric Rheumatology, Joseph M. Sanzari Children’s Hospital, Hackensack Meridian School of Medicine, Hackensack, NJ, United States

####### **Correspondence:** Suzanne C. Li

*Pediatric Rheumatology 2024*, **22(2)**: PReS24-ABS-1422

**Introduction:** Juvenile systemic sclerosis (jSSc) is a rare autoimmune and fibrosing disease associated with significant morbidity and mortality. Current data on treatment strategies is limited and primarily based on adult data. As jSSc has many differences from adult SSc, including a higher overlap subtype frequency, jSSc studies are needed to provide optimal care. An international collaboration based on CARRA and PReS was formed to generate consensus jSSc organ-specific outcomes measures for use in future studies. We report on the findings of the skin and musculoskeletal subgroup.

**Objectives:** To identify and evaluate cutaneous and musculoskeletal outcome measures for adult and juvenile SSc utilizing a scoping literature review.

**Methods:** Four databases identified 24,849 articles. Utilizing the inclusion criteria of English language, original peer-reviewed research related to SSc, and study of a measure from 1 of the 6 organs of interest, 3,284 articles were identified for organ-specific review. The skin/musculoskeletal subgroup subsequently identified 135 articles that reported longitudinal data of measures likely to change in a 1-year clinical trial. These articles underwent full text extraction. Collected data included study type, patient characteristics, anatomic domains, and measure findings.

**Results:** The majority of studies identified were single country (80%), single center (61%), and interventional (84%). Randomized controlled trials (31%) and prospective cohort (36%) were the most common study types. Only 6 studies included patients with jSSc (4%). Of the 121 studies where subtype distribution was noted, diffuse cutaneous (99%) was studied more frequently than limited (52%). Only 5 studies (4%) noted inclusion of overlap patients. Of the anatomic domains assessed, skin was unsurprisingly the most common (84%). The musculoskeletal system was investigated less commonly, with relatively few studies assessing the joint (19%), muscle (10%), and tendon (4%). Due to the complexity of the sclerodermatous hand, it was considered an independent domain and was assessed in 30% of studies. The modified Rodnan skin score (77%), joint counts including DAS28 (7%), and measures of grip/pinch strength (10%) were the most commonly studied measures of the cutaneous, musculoskeletal, and hand domains respectively.

**Conclusion:** This scoping review identified measures used to assess the cutaneous and musculoskeletal manifestations of SSc. Our results highlight the vast underrepresentation of jSSc and overlap subtype in the literature. Despite the prevalence of musculoskeletal involvement in SSc, it is an understudied domain, highlighting the need for additional studies to generate evidence-based recommendations.

**Patient Consent**

Not applicable (there are no patient data)

**Disclosure of Interest**

None Declared

### Scleroderma and related syndromes

#### P344 The influence of gene polymorphism on the risk of developing clinical and immunological phenotypes of scleroderma in children

##### Iryna Chyzheuskaya^1^, Tatyana Matsushko^2^, Anastasiya Chyzhevskaya^3^, Lyudmila Belyaeva^1^

###### ^1^4th City Children's Clinical Hospital; ^2^Minsk Scientific and Practical Center for Surgery, Transplantology and Hematology; ^3^Institute of Genetics and Cytology of the National Academy of Sciences of Belarus, Minsk, Belarus

####### **Correspondence:** Iryna Chyzheuskaya

*Pediatric Rheumatology 2024*, **22(2)**: PReS24-ABS-1602

**Introduction:** Scleroderma is one of the most disabling autoimmune diseases, characterized by an extensive fibrotic process that affects most organs and tissues. One of the central events responsible for the development of the disease is dysregulation of the immune system. The altered immune response in scleroderma is characterized by increased activation of T cells and the secretion of proinflammatory mediators, which contribute to the formation of fibrotic changes and impaired endothelial function - the main symptoms of scleroderma.

**Objectives:** to study the associations of polymorphic variants of genes, the products of which are involved in the regulation and implementation of the functions of the immune system, with the risk of developing juvenile systemic sclerosis (JSS) and juvenile limited scleroderma (JLS) in children.

**Methods:** Three groups of children (22 patients with JSS, 38 patients with JLS, 34 children without autoimmune and chronic inflammatory diseases (control)) were genotyped for 8 polymorphic loci of 6 genes: STAT4 (rs7574865), CTLA4 (rs231775), PTPN2 (rs2542151, rs7234029), RUNX1 (rs9979383), FOXP3 (rs3761548, rs2232365), TRAF1/C5 (rs3761847) using real-time PCR and PCR-RFLP.

**Results:** It was found that CC homozygotes at the rs9979383 гена RUNX1 gene are associated with JSS (OR 1.75 [1.18–2.58], p=0.0058), and GG homozygotes at the rs3761847 locus of the TRAF1/C5 gene are associated with JLS (OR 2.79 [1.29–6.06], p=0.01). It was shown that the GG genotype at the rs3761847 locus of the TRAF1/C5 gene (p=0.04; OR 1.66 [1.03–2.68]), CC genotype (p=0.0002; OR 2.26 [1, 47–3.47]) and allele C (p=0.006; OR 2.01 [1.14–3.52]) at the rs9979383 гена RUNX1 gene are associated with the onset of the disease before the age of 7 years. The group of patients with JSS as a whole differs significantly from the control group in the individual gene loci of FOXP3 (rs2232365), TRAF1/C5 (rs3761847), as well as in the frequencies of 33 pairwise combinations of genotypes of the studied polymorphic variants.

**Conclusion:** The obtained data on genetically determined differences in individual variants of JSS and JLS can serve as the basis for the development of additional criteria for the differential diagnosis of these diseases in children.

**Patient Consent**

Yes, I received consent

**Disclosure of Interest**

None Declared

### Scleroderma and related syndromes

#### P345 There is no difference in major organ involvement and antibody pattern between diffuse and limited subtype Juvenile onset systemic scleroderma patients

##### Ivan Foeldvari^1^, Jens Klotsche^2^, Kathryn Torok^3^, Ozgur Kasapcopur^3^, Amra Adrovic^3^, Brian Feldman^3^, Flavio Sztajnbok^3^, Jordi Anton^3^, Sindhu Johnson^3^, Maria Teresa Terreri^3^, Ana Paula Sakamoto^3^, Raju Khubchandani^3^, Valda Stanevicha^3^, Gülcan Özomay Baykal^3^, Dieneke Schonenberg-Meinema^3^, Eslam Al-Abadi^3^, Ekaterina Alexeeva^3^, Maria Katsicas ^3^, Sujata Sawhney^3^, Vanessa Smith^3^, Simone Appenzeller^3^, Tadey Avcin ^3^, Mikhail Kostik^3^, Thomas Lehman^3^, Suzanne Li^3^, Hana Malcova^3^, Edoardo Marrani^3^, Clare Pain^3^, Anjali Patwardhan^3^, W.-Alberto Sifuentes-Giraldo^3^, Natalia Vasquez-Canizares^3^, Sima Abu Al-Saoud^3^, Patricia Costa Reis^3^, Stefanie Hajek^3^, Mahesh Janarthanan ^3^, Monika Moll^3^, Dana Nemcova ^3^, Siri Opsahl Hetlevik^3^, Maria Jose Santos^3^, Cristina Battagliotti^3^, Lillemor Berntson^3^, Bica, Blanca Bica, Blanca^3^, Jürgen Brunner^3^, Despina Eleftheriou^3^, Liora Harel^3^, Gerd Horneff^3^, Daniela Kaiser^3^, Tilmann Kallinich^3^, Dragana Lazarevic^3^, Kirsten Minden^2^, Susan Nielsen^3^, Farzana Nuruzzaman^3^, Mihaela Sparchez^3^, Yosef Uziel ^3^, Nicola Helmus^1^

###### ^1^Hamburg Centre for Pediatric and Adolescence Rheumatology, Hamburg, ^2^Deutsches Rhemaforschungszentrum, Berlin, ^3^jSSc collaborative group, Hamburg, Germany

####### **Correspondence:** Ivan Foeldvari

*Pediatric Rheumatology 2024*, **22(2)**: PReS24-ABS-1194

**Introduction:** In adult systemic sclerosis they are significant differences in clinical presentation of diffuse and limited subtype. In juvenile systemic sclerosis (jSSc) are the differences less prominent as we reviewed last time in a publication for the first 150 patients [1] of the juvenile scleroderma inception cohort (jSScC). The differences are changing as the included number of patients is growing in the cohort.

**Objectives:** To review the differences of clinical characteristics of patients and patient (PRO) and physician (PhRO)reported outcomes with limited jSSc (ljSSc) and diffuse (djSSc) subtype in the jSScC.

**Methods:** We extracted date from the jSScC including patients who were enrolled till 1^st^ April 2024 into the cohort [1]. We compared the clinical characteristics, patient reported outcomes and physician reported outcomes of the two subtypes and calculated statistical significance using chi-square test.

**Results:** 268 patients were included in the study. 70% (n=188) of the patients had diffuse subtype. Around 70% of the patients were Caucasian in both groups. The median age of onset of Raynaud´s were 10.1 in the djSSc and 11.8 years in the ljSSc. The median age at the time of the first non-Raynaud was 10.1 years in the djSSc and 11.8 years in the ljSSc. The antibody profile with number of patients with ANA, anti-Scl70, anti-centromere and anti-PMscl positivity was not different between subtypes either. Modified Rodnan skin score (16 versus 4, p=0.001), more frequently Gottron papules (32% versus 16%, p=0.032), with sclerodactyly (83% versus 53%, p<0.001), with telangiectasia (43% versus 21%, p=0.011), with history of ulceration (61% versus 32%, p<0.015), with decreased Body mass Index <2 standard deviation(20% versus 6%, p=0.004). None of the patients had renal crisis. There was no significant difference in cardiopulmonary, gastrointestinal involvement and musculoskeletal involvement. Looking at PRO and PhRO in all categories, global disease activity, global disease damage and ulceration activity djSSc patients had significantly more severe disease.

**Conclusion:** These results present a different organ involvement pattern form adult patients. Despite more severe disease according to patient and physician reported outcomes, we found no significant differences in the cardiopulmonary, renal, gastrointestinal and musculoskeletal organ involvement between the subtypes. The antibody profile with number of patients with ANA, anti-Scl70, anti-centromere and anti-PMscl positivity was not different between subtypes either. It seems to be that for pediatric patients the subsetting into diffuse and limited does not make so much difference.

**Patient Consent**

Not applicable (there are no patient data)

**Disclosure of Interest**

None Declared

**Reference**

1. Foeldvari I, Klotsche J, Kasapcopur O, et al. Differences Sustained Between Diffuse and Limited Forms of Juvenile Systemic Sclerosis in an Expanded International Cohort. Arthritis Care Res (Hoboken). 2022 Oct;74(10):1575-1584.

### Scleroderma and related syndromes

#### P346 Improvement Across Physician And Patient Reported Outcome Measures Over A 24 Months-Time Period In The Juvenile Systemic Scleroderma Inception Cohort

##### Ivan Foeldvari ^1^, Jens Klotsche^2^, Ozgur Kasapcopur^3^, Amra Adrovic^3^, Kathryn Torok^3^, Maria Teresa Terreri^3^, Ana Paula Sakamoto^3^, Jordi Anton^3^, Brian Feldman^3^, Raju Khubchandani^3^, Tadey Avcin ^3^, Sindhu Johnson^3^, Mikhail Kostik^3^, Edoardo Marrani^3^, Flavio Sztajnbok^3^, Maria Katsicas ^3^, Dana Nemcova ^3^, Maria Jose Santos^3^, Simone Appenzeller^3^, Cristina Battagliotti^3^, Lillemor Berntson^3^, Jürgen Brunner^3^, Liora Harel^3^, Gerd Horneff^3^, Tilmann Kallinich^3^, Kirsten Minden^2^, Monika Moll^3^, Farzana Nuruzzaman^3^, Anjali Patwardhan^3^, Dieneke Schonenberg-Meinema^3^, Nicola Helmus^1^

###### ^1^Hamburg Centre for Pediatric and Adolescence Rheumatology, Hamburg; ^2^Deutsches Rhemaforschungszentrum, Berlin; ^3^jSSc collaborative group, Hamburg, Germany

####### **Correspondence:** Ivan Foeldvari

*Pediatric Rheumatology 2024*, **22(2)**: PReS24-ABS-1226

**Introduction:** Juvenile systemic sclerosis (jSSc) is an orphan disease with a prevalence of 3 in 1 000 000 children. The Juvenile Systemic Scleroderma Inception cohort (jSScC) is the largest cohort of jSSc patients in the world. The jSScC collects longitudinal data prospectively in jSSc, allowing the evaluation of the development of organ involvement and patients and physician reported outcomes in jSSc over time.

**Objectives:** To review changes in clinical characteristics and patient and physician reported outcomes over the 24 months observation period since enrollment in the cohort.

**Methods:** The jSScC enrols jSSc patients who developed the first non-Raynaud ´s symptom before the age of 16 years and are under the age of 18 years at the time of inclusion. We reviewed jSScC patient clinical data and patient and physician reported outcomes of those with 24 months follow up from the time of inclusion until 1st of April 2024.

**Results:** We could enrol 101 patients with 24 months follow up. 76% of them had diffuse subtype and 82% of them were Caucasian. Median age of onset of Raynaud´s was 9.6 years and median age at the first non-Raynaud was 10.1 years. 31% of the patients were anti-scl70 positive and 3% anti-centromere positive. The Modified Rodnan Skin Score decreased from 12 to 7.5 (p=0.023). The number of patients with active ulceration remained in the same range 20% at time point “0 “and 18% after 24 months. The number of patients with FVC<80% increased for 36% to 44% and DLCO<80% from 49% to 50%, both changes were not significant. The cardiac involvement stayed stable at 3% of the patients. The number of patients with pulmonary hypertension increased 5% to 7% (non-significant). The number of patients with 6 Minute Walk Distance (6MWT) less then 10th percentile decreased significantly from 71% to 57% (p=0.047). No patient developed renal crisis and only 1 patient developed hypertension. The gastrointestinal involvement stayed in the same range with 43% at time point “0” and 37% after 24 months. The number of patients with muscle weakness significantly decreased from 16% to 5% (p=0.019) otherwise lo involvement stayed unchanged. Interestingly all patient and physician reported outcomes related to global disease activity and damage, ulceration acitivity and Raynaud activity, improved significantly over 24 months, beside the CHAQ score.

**Conclusion:** The number of patients with decreased 6MWT under the 10 percentile and muscle weakness improved significantly over 24 months. It is reassuring that major internal organ pattern, such as cardiac, pulmonary, renal and gastrointestinal remained stable. No renal crisis occurred over the 24-month time period. All assessed patient and physician-reported outcomes improved significantly over the 24 months, beside the CHAQ score. It seems to be that the applied therapy made measurable improvement for patients with not many significant changes in the organ involvement pattern.

**Patient Consent**

Not applicable (there are no patient data)

**Disclosure of Interest**

None Declared

### Scleroderma and related syndromes

#### P347 Guidance for stem cell therapy for juvenile systemic sclerosis patients

##### Ivan Foeldvari ^1^, Kathryn Torok^2^, Juliana Silva^3^, Paulina Horvei^2^, Christopher Denton^4^, Franziska Roser^2^, Tamas Constantin^5^, Patricia Costa Reis^6^, Megan Curran^7^, Maurizio Cutolo^8^, Jörg Henes^9^, Bernd Hinrichs^10^, Kim Fligelstone^11^, Suzanne Li^12^, Susan Maillard^3^, Pia Moinzadeh^13^, Catherine Orteu^14^, Clare Pain^15^, Clarissa Pilkington^3^, Linda Schraven^16^, Vanessa Smith^17^

###### ^1^Hamburg Centre for Pediatric and Adolescence Rheumatology, Hamburg, Germany; ^2^University of Pittsburgh and University of Pittsburgh Medical Center Children’s Hospital of Pittsburgh , Pittsburgh, United States; ^3^Great Ormond Street Hospital; ^4^UCL Division of Medicine, Royal Free London NHS Foundation Trust , London , United Kingdom; ^5^Unit of Pediatric Rheumatology, Tűzoltó Street Department, Pediatric Centre, Semmelweis University , Budapest , Hungary; ^6^Faculdade de Medicina, Universidade de Lisboa , Lisboa , Portugal; ^7^Children's Hospital Colorado , Colorado, United States; ^8^University of Genoa and IRCCS San Martino Polyclinic Hospital , Genoa , Italy; ^9^University Medical Center Tuebingen , Tuebingen; ^10^Children's pulmonology, Asklepios Klinik Nord – Heidberg , Hamburg, Germany; ^11^FESCA , London , United Kingdom; ^12^Hackensack University Medical Center , Hackensack , United States; ^13^University Hospital Cologne , Cologne, Germany, ^14^Royal Free London NHS Foundation Trust , London; ^15^Alder Hey Children’s Foundation NHS Trust , Liverpool; ^16^Dutch representative of Fesca, London , United Kingdom; ^17^Ghent University , Ghent, Belgium

####### **Correspondence:** Ivan Foeldvari

*Pediatric Rheumatology 2024*, **22(2)**: PReS24-ABS-1229

**Introduction:** Hemopoetic stem cell transplantation(HSTC) and cellular therapies(CT) are a promising therapeutic options for juvenile systemic sclerosis(jSSc) patients. As pediatric patients have a unique potential to recover and remodel, the applicability of the adult stem cell transplant criteria to pediatric patients with jSSc is unclear/unknown and may be too stringent.

**Objectives:** To develop guidance for HSCT and CT for patients with jSSc.

**Methods:** At a multidisciplinary expert workshop conducted in Hamburg Germany in December 2023, adult and pediatric data were reviewed regarding indications and effectiveness of HSCT and CT. We discussed the extent adult data and criteria can be extrapolated to juvenile patients, and its limitations. In the consensus meeting based on nominal group technique,a guidance regarding HSCT and CT in jSSc was formulated.

**Results:** 1.All types of jSSc can be considered for a haematopoietic stem cell transplant or CT regardless of disease duration. 17/17 voted yes.

2. All types of juvenile systemic sclerosis (limited cutaneous, diffuse cutaneous, overlap and sine scleroderma) can be considered. 17/17 voted yes.

3. To be considered for HSCT or CT the patient should have:

a) Progression of disease or lack of improvement despite treatment with ≥2 disease-modifying antirheumatic drugs (DMARDS). 17/17 voted yes.

b) Moderate or severe disease. 17/17 voted yes.

c) Been assessed by a multidisciplinary team with expertise in jSSc. 17/17 voted yes.

4. In addition to above, patients with jSSc should have ≥ 1 of the following:

a) Moderate or severe skin involvement or progression of skin thickening. 17/17 voted yes.

b) Moderate or severe interstitial lung disease, moderate or severe respiratory impairment or progression of interstitial lung disease. 16 voted yes.

c) Moderate or severe myositis/myopathy, or progression of myositis/myopathy. 17/17 voted yes.

d) Moderate or severe cardiac involvement or progression of cardiac involvement. 17/17 voted yes.

5. The following are additional considerations:

a) Moderate or severe gastrointestinal dysfunction or progression of gastrointestinal dysfunction. 16/16 voted yes.

b) Moderate or severe arthritis/arthropathy or progression of arthritis/arthropathy. 14 voted yes,

c) Moderate or severe cutaneous ulceration or progression of cutaneous ulceration. 12 voted yes.

6. When considering stem cell transplant or CT (in regards to 4 and 5), greater importance was attributed to progression rather than degree of severity by the expert panel. 16 voted yes.

**Conclusion:** We established a guidance, which will help worldwide to standardize the inclusion criteria and make the results of the future procedures more comparable. We hope that HSCT and CT will have standardized exclusion criteria and transplant protocols to enable data collection, and improve outcomes and care.

**Patient Consent**

Not applicable (there are no patient data)

**Disclosure of Interest**

None Declared

### Scleroderma and related syndromes

#### P348 Juvenile localised scleroderma: a single centre experience

##### Elif Kılıç Könte^1^, Nergis Akay^1^, Umit Gul^1^, Aybuke Gunalp^1^, Esma Aslan^1^, Fatih Haslak^1^, Ozgur Demir^2^, Zeynep Altan Ferhatoglu^2^, Mehmet Yildiz^1^, Amra Adrovic^1^, Sezgin Sahin^1^, Kenan Barut^1^, Ozgur Kasapcopur^1^

###### ^1^Department of Child Health and Disease, Division of Pediatric Rheumatology; ^2^Department of Dermatology, Istanbul University-Cerrahpasa, Cerrahpasa Faculty of Medicine, Istanbul, Türkiye, Türkiye

####### **Correspondence:** Elif Kılıç Könte

*Pediatric Rheumatology 2024*, **22(2)**: PReS24-ABS-1681

**Introduction:** Localised scleroderma, or morphea, is a connective tissue disease characterized by thickening and inflammation of the skin and subcutaneous tissue due to excessive collagen deposition. It is divided into five groups: plaque, generalized, pansclerotic, linear, and deep morphea, according to the lesions' depth, number, shape, and size. The most common form is linear scleroderma. The pattern of involvement is localized to the skin and subcutaneous tissue, but internal organ involvement may rarely accompany it. Treatment options include local/systemic steroids and traditional disease-modifying agents such as methotrexate.

**Objectives:** This study aimed to determine the demographic and clinical characteristics of children with localized scleroderma, reveal the pathologies that may accompany them, and investigate the response to treatment.

**Methods:** The medical records of 77 pediatric patients with localized scleroderma treated at Cerrahpasa Pediatric Rheumatology Department between 2013 and 2023 were retrospectively reviewed. The study included demographic and clinical characteristics, laboratory data, biopsy diagnoses, medications, and complications during follow-up.

**Results:** Sixty (77.9%) of the patients were female. The mean age at the last visit, age at symptom onset, and age at diagnosis were 12.65(±3.9), 7.1(±3.7), and 9.08(±3.6) years, respectively. The median delay in diagnosis was 15.2(10.1-33) months and was higher in mixed type and pansclerotic morphea. The most common type of involvement was linear scleroderma, seen in 40(51.9%) patients; the diagnosis was confirmed by biopsy in 53(68.8%) patients, and the most common lesion location was the lower extremities (46; 39%). ANA was positive in 39 (58.2%) of 67 patients, and anti-Scl-70 was positive in one (1.6%) of 60 patients. The most common complications were skeletal muscle involvement in 21(27.2%) patients, craniofacial involvement in 11(14.2%) patients, and neuropsychiatric findings in 5(6.4%) patients. Methotrexate was initiated in 70(90.8%) patients, prednisolone in 61(79.2%) patients and combination therapy in 59(76.6%) patients. Prednisolone treatment was continued for six months (2-28).

**Conclusion:** Localised scleroderma, a connective tissue disease, has low complication rates with early diagnosis and treatment. It is essential to monitor and treat accompanying systemic findings.

**Date of birth::** octobre 12

**Patient Consent**

Yes, I received consent

**Disclosure of Interest**

None Declared

**References**

1. Zulian F, Culpo R, Sperotto F, et al. Consensus-based recommendations for the management of juvenile localised scleroderma. Ann Rheum Dis 2019;78(8):1019-1024, doi: 10.1136/annrheumdis-2018-214697
2. Adrovic A, Sahin S, Barut K, et al. Juvenile Scleroderma-What has Changed in the Meantime? Curr Rheumatol Rev 2018;14(3):219-225, doi: 10.2174/1573397114666180423105056

### Scleroderma and related syndromes

#### P349 Gastrointestinal outcome measures in systemic sclerosis: a scoping review

##### Lusine Ambartsumyan ^1^, Sarah Ishaq^2^, Lauren Robinson ^3^, Aybuke Gunalp^4^, Simone Appenzeller^5^, Ozgur Kasapcopur^6^, Emily Willis^7^, Natalia Vasquez-Canizares ^8^, Suzanne C. Li^9^ on behalf of International Juvenile Systemic Sclerosis Outcomes Group (IJOG)

###### ^1^Gastroenterology and Hepatology, Seattle Children's Hospital , Seattle; ^2^Osteopathic Medicine , Touro College of Osteopathic Medicine; ^3^Pediatric Rheumatology , Hospital for Special Surgery New York Presbyterian - Weill Cornell , New York , United States; ^4^Cerrahpasa Medicine School, Pediatric Rheumatology , Istanbul University - Cerrahpasa, Istanbul , Türkiye; ^5^Department of Orthopedics, Rheumatology and Traumatology , University of Campinas Faculdade de Ciencias Medicas, Campinas, Brazil; ^6^Cerrahpasa Medical School, Istanbul University - Cerrahpasa, Istanbul , Türkiye; ^7^Pediatric Rheumatology , Royal Manchester Children's Hospital , Manchester, United Kingdom; ^8^Pediatric Rheumatology , The Children's Hospital of Montefiore , New York; ^9^Pediatric Rheumatology , Joseph M Sanzari Children's Hospital , Hackensack Meridian School of Medicine , New Jersey , United States

####### **Correspondence:** Ozgur Kasapcopur

*Pediatric Rheumatology 2024*, **22(2)**: PReS24-ABS-1357

**Introduction:** The gastrointestinal (GI) tract is significantly affected in children and adults with systemic sclerosis (SSc) leading to significant morbidity and mortality. To date there are no specific predictors of disease progression, and a lack of standards for GI assessment in jSSc.

**Objectives:** To identify measures used to assess GI involvement in SSc as part of an international effort to generate consensus outcome measures for juvenile SSc trials.

**Methods:** We performed a scoping review of measures used to assess GI involvement in SSc to facilitate future therapeutic trials and areas of research in juvenile SSc. From 4 databases, 24,849 studies were initially screened, yielding 262 studies identified by title and abstract of which 124 full text articles were extracted. Twenty-six GI measures were identified. Measures were sorted by GI region studied, or those related to quality of life (QoL) or nutritional assessment.

**Results:** Most studies were observational retrospective or prospective case series, only 4% (5/124) were randomized control trials. Seventy-eight percent (97/124) of studies were single center and only 11% (14/124) were international collaborations. Most of the studied patients were adults, with only 1.4% (109/7801) including patients < 18 years of age. The most commonly studied GI region was the esophagus (82/124, 66%), then colorectum (26/124, 21%), stomach (22/124, 18%), small intestine (16/124, 13%), and oropharynx (10/124, 8%). Nutritional outcomes were reported in 7% (9/124) while QoL measures comprised 25% (31/124) of studies. Most measures were limited by a lack of standardization and variable and inconsistent use between studies. Pediatric data is lacking.

**Conclusion:** This scoping literature review identifies measures used for GI evaluation in SSc. It highlights the need for more pediatric studies, and for standardized and validated GI outcome measures that are amendable to longitudinal and therapeutic studies.

**Patient Consent**

Not applicable (there are no patient data)

**Disclosure of Interest**

None Declared

### Scleroderma and related syndromes

#### P350 Identifying specific criteria for juvenile systemic sclerosis

##### Suzanne C. Li ^1^, Natalia Vasquez-Canizares^2^, Clare Pain^3^, Marinka Twilt^4^, Amra Adrovic^5^, Abdulrahman Alrasheed^6^, Simone Appenzeller^7^, Adele Civino^8^, Muserref K. Cucueoglu^9^, Fatma Dedeoglu^10^, Sam Deepak^11^, Jianghong Deng^12^, Dalia El-Ghoneimy^13^, Fernando Garcia-Rodriguez^14^, Marija Jelusic^15^, Ankur Jindal^16^, Ozgur Kasapcopur^17^, Maria Katsicas^18^, Archana Khan^19^, Raju Khubchandani^19^, Meiping Lu^20^, Hanna Lythgoe^21^, Edoardo Marrani^22^, Giorgia Martini^23^, Takako Miyamae^24^, Tomo Nozawa^25^, Seza Ozen^9^, Lidia Rutkowska-Sak^26^, Sunil Sampath^27^, Dieneke Schonenberg-Meinema^28^, Hongmei Song^29^, Betul Sozeri^30^, Hayakazu Sumida^31^, Maria Terreri^32^, Kathryn Torok^33^, Emily Willis^21^, Brian M. Feldman^34^ and Pediatric International Consortium for Scleroderma

###### ^1^Pediatrics, Joseph M Sanzari Children's Hospital, Hackensack Meridian School of Medicine, Hackensack; ^2^Pediatrics, The Children's Hospital at Montefiore, New York, United States; ^3^Rheumatology, Alder Hey Children's NHS Foundation Trust, Liverpool, United Kingdom; ^4^Pediatrics, University of Calgary, Alberta, Canada; ^5^Pediatric Rheumatology, Koc University Hospital, Istanbul, Türkiye; ^6^Rheumatology, King Abdullah Specialized Children Hospital, Riyadh, Saudi Arabia; ^7^University of Campinas Faculdade de Ciências Médicas, Campinas, Brazil; ^8^Pediatric Rheumatology, Vito Fazzi Hospital, Lecce, Italy; ^9^Hacettepe University, Ankara, Türkiye, ^10^Boston Children's Hospital, Boston, United States; ^11^Nottingham Children's Hospital, Nottingham, United Kingdom; ^12^Rheumatism and Immunity, Beijing Children's Hospital, Beijing, China; ^13^Children’s Hospital, Ain Shams University, Cairo, Egypt; ^14^Universidad Autonoma de Nuevo Leon, Monterrey, Mexico; ^15^University Hospital Centre Zagreb, at the University of Zagreb, Zagreb, Croatia; ^16^Postgraduate Institute of Medical Education and Research, Chandigarh, India; ^17^Istanbul University-Cerrahpasa, Istanbul, Türkiye; ^18^Hospital de Pediatría Prof Dr JP Garrahan, Buenos Aires, Argentina, ^19^The Society for the Rehabilitation of Crippled Children Children’s Hospital, Mumbai, India; ^20^Children’s Hospital, Zhejiang University Medical School, Hangzhou, China; ^21^Royal Manchester Children's Hospital, Manchester, United Kingdom; ^22^AOU Meyer, Firenze; ^23^University of Udine University Hospital Santa Maria della Misericordia, Udine, Italy; ^24^Institute of Rheumatology, Tokyo Women's Medical University, Tokyo; ^25^Yokohama City University, Yokohama, Japan; ^26^National Institute of Geriatrics, Rheumatology and Rehabilitation, Warszawa, Poland; ^27^The Newcastle Upon Tyne Hospitals Nhs Foundation Trust, Newcastle Upon Tyne, United Kingdom; ^28^Emma Children’s Hospital, Amsterdam University Medical Centers, Amsterdam, Netherlands; ^29^Peking Union Medical College, Beijing, China; ^30^University of Health Sciences, Umraniye Training and Research Hospital, Istanbul, Türkiye; ^31^The University of Tokyo, Tokyo, Japan; ^32^Universidade Federal de São Paulo, São Paulo, Brazil, ^33^Children's Hospital of Pittsburgh, U Pittsburgh, Pittsburgh, United States; ^34^The Hospital for Sick Children, University of Toronto, Toronto, Canada

####### **Correspondence:** Suzanne C. Li

*Pediatric Rheumatology 2024*, **22(2)**: PReS24-ABS-1027

**Introduction:** Despite the high morbidity and mortality risk associated with juvenile systemic sclerosis (jSSc), evidence to guide management is limited^1, 2^. No jSSc clinical trials have been conducted, and relying on adult SSc recommendations is suboptimal because of differences between juvenile and adult onset SSc^3^. Current classification criteria (CC) have limited sensitivity for jSSc; 66% for the 2007 PRES/EULAR/ACR jSSc CC^4^, and 81% for the 2013 ACR/EULAR adult SSc CC^4,6^, versus 91-98% for the 2013 adult CC for adult SSc^5, 7^.

Our Pediatric International Consortium for Scleroderma (PICS) group, consisting mainly of pediatric rheumatologists, is working to develop high performing CC for jSSc treatment studies. We conducted a survey of members to identify potential criteria for inclusion.

**Objectives:** To identify criteria for generating high performing classification criteria (CC) for jSSc

**Methods:** A web-based survey was sent asking PICS members to rate the appropriateness of features to serve as a criteria for CC (1 low, 9 high). Queried features included the 23 evaluated by adult experts during development of the 2013 adult CC^8^. Descriptive statistics were used. Features were ranked based upon median appropriateness score, more raters scoring it in the top 3 levels and fewer scoring it in the lowest 3 levels.

**Results:** Thirty-seven PICS members from 16 countries answered the survey (70% response). PICS members rated vasculopathy-related features the most appropriate, while adult experts ranked skin thickening and autoantibodies (anti-topoisomerase, anticentromere) the highest^8^. Other features rated higher by PICS members than adult experts were anti-PM/Scl autoantibody positivity (level 8 vs 5), persistent gastroesophageal reflux disease (7 vs 4), and reduced diffusion coefficient for carbon monoxide (7 vs 4). Renal crisis, tendon friction rubs, and anti-RNA polymerase III autoantibody positivity were rated lower by PICS members than adults. New items rated by PICS members included restricted oral aperture and diffuse skin atrophy, each rated level 7, and anti-U1 RNP positivity, arthritis, and early satiety, all rated level 6.

We compared the appropriateness scores of PICS members stratified by number of jSSc patients seen. Group A had seen 21 to > 60 jSSc patients, B 11-20 patients, and C 1-10 patients. Only small inter-group rating differences were identified for calcinosis, interstitial lung disease, pulmonary arterial hypertension (PAH), and pulmonary function test parameters. Groups reported a similar prevalence of these features in their patients except for PAH and arrythmias, which were more common in group A patients.

**Conclusion:** Irrespective of the number of jSSc patients they follow, PICS members had similar appropriateness ratings for most features. Ratings differed between pediatric and adult rheumatologists for several features, suggesting modifications of the 2013 adult CC could improve its performance for jSSc.

**Patient Consent**

Not applicable (there are no patient data)

**Disclosure of Interest**

None Declared

**References**

1. Zulian F, et al. Expert Rev Clin Immunol 2017;13:361-9.
2. Foeldvari I, et al. Rheumatology (Oxford) 2021;60:1651-8.
3. Li S, McCormick Q. EMJ Rheumatology 2022;9:47-58.
4. Zulian F, et al. Arthritis Rheum 2007;57:203-12.
5. van den Hoogen F, et al. Arthritis Rheum 2013;65:2737-47.
6. Stevens BE, et al. Arthritis Care Res 2018;70:1806-13.
7. Melchor S, et al. Semin Arthritis Rheum 2016;46:350-5.
8. Fransen J, Jet al. Arthritis Care Res 2012;64:351-7

### Scleroderma and related syndromes

#### P351 Presence of nailfold capillary changes correlates with more severe organ involvement in juvenile systemic scleroderma. Results of the Juvenile scleroderma inception cohort

##### Ivan Foeldvari ^1^, Jens Klotsche^2^, Kathryn Torok^3^, Ozgur Kasapcopur^3^, Amra Adrovic^3^, Brian Feldman^3^, Jordi Anton^3^, Sindhu Johnson^3^, Flavio Sztajnbok^3^, Maria Teresa Terreri^3^, Ana Paula Sakamoto^3^, Valda Stanevicha^3^, Dieneke Schonenberg-Meinema^3^, Ekaterina Alexeeva^3^, Maria Katsicas ^3^, Raju Khubchandani^3^, Sujata Sawhney^3^, Vanessa Smith^3^, Eslam Al-Abadi^3^, Simone Appenzeller^3^, Tadey Avcin ^3^, Mikhail Kostik^3^, Thomas Lehman^3^, Hana Malcova^3^, Edoardo Marrani^3^, Clare Pain^3^, Anjali Patwardhan^3^, W.-Alberto Sifuentes-Giraldo^3^, Natalia Vasquez-Canizares^3^, Sima Abu Al-Saoud^3^, Patricia Costa Reis^3^, Mahesh Janarthanan ^3^, Monika Moll^3^, Dana Nemcova ^3^, Maria Jose Santos^3^, Cristina Battagliotti^3^, Lillemor Berntson^3^, Bica, Blanca Bica, Blanca^3^, Jürgen Brunner^3^, Despina Eleftheriou^3^, Liora Harel^3^, Gerd Horneff^3^, Daniela Kaiser^3^, Tilmann Kallinich^3^, Dragana Lazarevic^3^, Kirsten Minden^2^, Susan Nielsen^3^, Farzana Nuruzzaman^3^, Mihaela Sparchez^3^, Yosef Uziel ^3^, Nicola Helmus^1^

###### ^1^Hamburg Centre for Pediatric and Adolescence Rheumatology, Hamburg; ^2^Deutsches Rhemaforschungszentrum, Berlin; ^3^jSSc collaborative group, Hamburg, Germany

####### **Correspondence:** Ivan Foeldvari

*Pediatric Rheumatology 2024*, **22(2)**: PReS24-ABS-1227

**Introduction:** Juvenile systemic scleroderma (jSSc) is an orphan disease with a prevalence in 3 in 1 000 000 children. Abnormal nailfold capillaroscopy(NF+) finding correlate with more severe disease in adult systemic scleroderma[1]. There is currently no data if this correlation does exist in jSSc.

**Objectives:** To assess the difference in patients with jSSc with normal (NF-) and abnormal NF(NF+) findings at the time of inclusion in the cohort.

**Methods:** Baseline data was extracted from patients enrolled in the juvenile scleroderma inception cohort that had nailfold capillaroscopy performed at inclusion [2] until 1^st^ of December 2023. NF was performed by dermatoscope and/or high resolution video nailfold capillaroscopy. We compared patients with NF+ and NF- findings from the baseline visit using chi-square test.

**Results:** 237 patients were included in the analysis, 185 (78%) of them were female. 126 (70%) had diffuse subtype. 183/237 patients (77%) were in the NF+ group. 71% in the NF+ group were Caucasian compared to 85% in the NF- group (p=0.051). Median disease duration was 2.3 years in the NF+ and 3.2 years in the NF- patients. Median age at onset of the first non-Raynaud´s was around 11 years in both groups. More patients in the NF+ group were ANA positive (95% compared to 79%, p <0.001). There was no difference in the anti-Scl70 or anti-centromere distribution.

NF+ patients had significantly more frequent Raynaud phenomenon (96% compared to 78%, p<0.001); history of digital ulcerations (59% compared to 27%, p<0.001); abnormal high resolution CT findings of the lung ( 49% compared to 30%, p=0.034); overall gastrointestinal involvement (49% compared to 20%, p<0.001); oesophageal involvement (47% compared to 19%, p<0.001); musculoskeletal involvement ( 71% compared to 41%, p=0.003); presence of joints with decreased range (63% versus 45%, p=0.022) and presence of muscle weakness (25% compared to 3%, p=0.002). No significant differences were demonstrated in involvement of other organ systems such as skin, cardiac or renal.

**Conclusion:** In a jSSc cohort there were significantly more patients affected within various organ systems in those with abnormal nailfold capillary changes at enrollment compared to those without . Future studies should assess whether these differences persist over time.

**Patient Consent**

Not applicable (there are no patient data)

**Disclosure of Interest**

None Declared

**References**

1. Vanhaecke A, Cutolo M, Distler O, et al. Nailfold capillaroscopy in SSc: innocent bystander or promising biomarker for novel severe organ involvement/progression? Rheumatology (Oxford). 2022 Nov 2;61(11):4384-4396.
2. Foeldvari I, Klotsche J, Kasapcopur O, et al. Differences Sustained Between Diffuse and Limited Forms of Juvenile Systemic Sclerosis in an Expanded International Cohort. Arthritis Care Res (Hoboken). 2022 Oct;74(10):1575-1584.

### Scleroderma and related syndromes

#### P352 Is there a difference in the number of involved organ systems between Juvenile diffuse and limited subtype systemic sclerosis patients?

##### Ivan Foeldvari ^1^, Jens Klotsche^2^, Kathryn Torok^3^, Ozgur Kasapcopur^3^, Amra Adrovic^3^, Brian Feldman^3^, Flavio Sztajnbok^3^, Jordi Anton^3^, Sindhu Johnson^3^, Maria Teresa Terreri^3^, Ana Paula Sakamoto^3^, Raju Khubchandani^3^, Valda Stanevicha^3^, Gülcan Özomay Baykal^3^, Dieneke Schonenberg-Meinema^3^, Eslam Al-Abadi^3^, Ekaterina Alexeeva^3^, Maria Katsicas ^3^, Sujata Sawhney^3^, Vanessa Smith^3^, Simone Appenzeller^3^, Tadey Avcin ^3^, Mikhail Kostik^3^, Thomas Lehman^3^, Suzanne Li^3^, Hana Malcova^3^, Edoardo Marrani^3^, Clare Pain^3^, Anjali Patwardhan^3^, W.-Alberto Sifuentes-Giraldo^3^, Natalia Vasquez-Canizares^3^, Sima Abu Al-Saoud^3^, Patricia Costa Reis^3^, Stefanie Hajek^3^, Mahesh Janarthanan ^3^, Monika Moll^3^, Dana Nemcova ^3^, Siri Opsahl Hetlevik^3^, Maria Jose Santos^3^, Cristina Battagliotti^3^, Lillemor Berntson^3^, Bica, Blanca Bica, Blanca^3^, Jürgen Brunner^3^, Despina Eleftheriou^3^, Liora Harel^3^, Gerd Horneff^3^, Daniela Kaiser^3^, Tilmann Kallinich^3^, Dragana Lazarevic^3^, Kirsten Minden^2^, Susan Nielsen^3^, Farzana Nuruzzaman^3^, Mihaela Sparchez^3^, Yosef Uziel ^3^, Nicola Helmus^1^

###### ^1^Hamburg Centre for Pediatric and Adolescence Rheumatology, Hamburg; ^2^Deutsches Rhemaforschungszentrum, Berlin; ^3^jSSc collaborative group, Hamburg, Germany

####### **Correspondence:** Ivan Foeldvari

*Pediatric Rheumatology 2024*, **22(2)**: PReS24-ABS-1225

**Introduction:** Juvenile systemic sclerosis (jSSc) is an orphan disease with a prevalence of 3 in 1 000 000 children. In adult patients diffuse subtype is associated with higher number of organ systems involvement. In a CARRA North American study, it was noted that 38% of jSSc patients had four or more organ systems involved. This topic has not yet been assessed in the currently largest juvenile scleroderma inception cohort (jSSic) cohort.

**Objectives:** To assess the number of organ systems involved in the diffuse and limited jSSc patients at the time of inclusion in the cohort.

**Methods:** The jSSic is a prospective cohort including patients, who fulfill the adult SSc criteria[1], with first non-Raynaud symptom before the age of 16 years and under 18 years of age at the time of inclusion. We reviewed the number of organ systems involved at the time of inclusion into the cohort in patients, who were included till 1^st^ of April 2024. The categorization of the organ system involvement was skin, vascular, muscular, articular, pulmonary, cardiac, gastrointestinal, renal and nervous system. We compared the number of involved systems between diffuse and limited jSSc subtype.

**Results:** Until 1st of April 2024, 268 patients were enrolled and 188 of them had diffuse subtype. The median age at the onset of Raynaud´s was 10.4 years. The median age of the first non-Raynaud organ involvement was 10.9 years. The median disease duration was 2.5 years. 48% in the diffuse subset and 39% of the limited subset had in involvement of 4 or more organ systems involved. There was no significant difference between the cumulative number of organ systems, number of involved organs 1 to 7, between the jSSc subtypes in the Inception cohort.

**Conclusion:** In this currently largest jSSc cohort in the world, around 43 % the enrolled children have 4 or more organ systems involved, which highlights the overall severity of the disease. There was no significant difference in jSSc children skin subtypes, lcSSc or dcSSc regarding, the cumulative number of organ systems involved, number of involved organs 1 to 7, although as shown in our publications [2] the diffuse subtype presented more severe disease.

**Patient Consent**

Not applicable (there are no patient data)

**Disclosure of Interest**

None Declared

**References**

1. van den Hoogen F, Khanna D, Fransen J, et al. 2013 classification criteria for systemic sclerosis: an American college of rheumatology/European league against rheumatism collaborative initiative. Ann Rheum Dis. 2013 Nov;72(11):1747-55.
2. Foeldvari I, Klotsche J, Kasapcopur O, et al. Differences Sustained Between Diffuse and Limited Forms of Juvenile Systemic Sclerosis in an Expanded International Cohort. Arthritis Care Res (Hoboken). 2022 Oct;74(10):1575-1584.

### Scleroderma and related syndromes

#### P353 Juvenile localized scleroderma: experience of a pediatric rheumatology unit

##### M Ines Nunes Marques, Inês Madureira, Cristina Henriques, Marta Conde, Margarida P. Ramos

###### Pediatric Rheumatology Unit , Hospital Dona Estefânia, ULS São José, Lisbon, Portugal

####### **Correspondence:** M Ines Nunes Marques

*Pediatric Rheumatology 2024*, **22(2)**: PReS24-ABS-1787

**Introduction:** Juvenile localized scleroderma (LS), also called morphea, is a rare disease characterized by inflammation of the skin and subdermal tissues, triggering fibrosis that can lead leading to functional and aesthetic sequelae. The Pediatric Rheumatology European Society classifies LS into five subtypes: linear (most frequent), circumscribed, generalized, pansclerotic, and a mixed subtype which includes a combination of two or more subtypes.

Between 20-70% of LS patients have extracutaneous involvement (musculoskeletal, neurological, ocular and oral). Approximately 10% of these patients have an additional autoimmune (AI) disease.

**Objectives:** Characterization of demographics, clinical and complementary findings, treatment and outcome in children with LS, followed in a tertiary reference center for pediatric rheumatic diseases.

**Methods:** A single center retrospective analysis of children diagnosed with LS from 2008 to April 2024.

**Results:** Of the 18 patients, 16 were female, with a median age at diagnosis of 7 years (5-18y), and a median time to diagnosis of 2.3y (0.15-15y).

The most common form of LS was linear scleroderma (9/18; 6 with head and neck involvement, 2 head and limbs and 1 only the limbs), followed by circumscribed (5/18), mixed (3/18) and generalized morphea (1/18).

Half had family history of AI diseases and 4/18 had previous diagnosis of other AI diseases.

Regarding extracutaneous manifestations: neurological manifestations were present in 4 patients, all with Linear Head Scleroderma (2 preceding the soft-tissue lesions); 4/18 had limited articular mobility; and 2/18 had Raynaud Phenomenon.

At diagnosis, 12/17 had positive ANA (³1:80), 2/17 had positive anti-Scl70 (without evidence of any systemic involvement) and 1/17 had anti-centromere antibody. Confirmatory skin biopsy was performed in 13/17 patients.

Regarding treatment, 2/18 achieved remission exclusively with topical treatment (corticoid, tacrolimus).All other patients required systemic immunosuppression and were initially treated with methotrexate (MTX) 12 /16 in combination with glucocorticoids. Five needed repeat pulses of methylprednisolone due to disease flare, and two switched from MTX to mycophenolate mofetil (MMF) due to persistently active disease. Follow-up time ranged from 1 to 14y, with clinical stability at this date.

**Conclusion:** Our findings are in accordance with LS literature, regarding female predominance, positive personal and family AI history and linear scleroderma being the most common subtype of LS. The preferred DMARD was MTX, with remissionin all but two patients who had refractory cutaneous and/or extracutaneous disease; recent studies provide further support for the use of MMF in LS. Studies indicate that pediatric-onset LS has a poorer prognosis than adult-onset LS. Close monitoring of the disease is particularly important in the first 2 years after discontinuation of treatment when disease relapses occur more frequently.

**Patient Consent**

Not applicable (there are no patient data)

**Disclosure of Interest**

None Declared

### Services and pathways of care

#### P358 Exploring vaccination coverage and attitudes among children and adults with rheumatic diseases on biologic therapies: understanding cocooning strategies, under-vaccination factors and predictors of favorable attitudes

##### Charikleia Kariniotaki^1, 2^, George Bertsias^2, 3, 4^, Emmanouil Galanakis^1, 2^, Chrysoula Perdikogianni^1, 2^

###### ^1^Department of Pediatrics, University Hospital of Heraklion; ^2^Medical School, University of Crete; ^3^Department of Rheumatology and Clinical Immunology, University Hospital of Heraklion; ^4^Institute of Molecular Biology and Biotechnology-FORTH, Heraklion, Greece

####### **Correspondence:** Charikleia Kariniotaki

*Pediatric Rheumatology 2024*, **22(2)**: PReS24-ABS-1158

**Introduction:** In patients receiving therapy with biologic agents, infections pose a substantial risk of morbidity and mortality, which can be mitigated by vaccination of both the patients themselves and their close contacts. However, a notable gap exists in comprehending the adequacy of immunization among individuals receiving biologic agents, as published data on this matter are scarce, while no studies have been published regarding the vaccination status of their close contacts.

**Objectives:** Τo evaluate the vaccination coverage of children and adults with rheumatic diseases who are treated with biologics, as well as their household contacts, in a cohort of patients followed in different clinical settings in Heraklion, Crete. Second, to determine the reasons for inadequate vaccination, highlight possible correlations of demographic, clinical and behavioral parameters with vaccination status, as well as to identify factors related to the favorable attitude of patients toward vaccination.

**Methods:** A cross-sectional observational study was conducted in adult and pediatric patients treated with biologic agents and followed in different clinical settings in Heraklion, Crete, from October 2022 to January 2023. Face-to-face or telephone interviews were conducted using pre-structured questionnaires and further data were collected from medical files and the electronic prescription system. Analysis was performed considering the compliance of each individual to the age–appropriate recommended types and doses per vaccine examined, according to the National Immunization Program of Greece.

**Results:** Among 446 adult patients examined, vaccination rates were as follows: COVID-19 - 83%, influenza - 73.8%, PCV - 64.5% and PPSV - 29.6%, while Tdap vaccination stood at 4%. Among 26 pediatric patients, coverage rates with the vaccines of the basic schedule exceeded 96%, but rates for the vaccines administered usually at adolescence were lower (Tdap 47.8%, HPV 42.1%, MenACWY 66.7%). COVID-19 vaccination stood at 38.5%. Regarding the additional vaccines recommended due to treatment-induced immunosuppression, annual influenza vaccination was complete in 69.2% of pediatric patients, while only 19.2% had received the PPSV vaccine. Household contacts demonstrated low vaccination rates across most vaccines, except for COVID-19. Female gender (p<0.007) and increased age (p<0.001) were associated with favorable attitudes and higher vaccination coverage in adults, while in pediatric patients there were no statistically significant associations. Finally, the primary reason for not being vaccinated was the lack of recommendation from their treating physician.

**Conclusion:** The results of the present study indicate that receipt of a biologic agent may be underestimated and not always taken into account among indications for vaccination of the patients and their close contacts. Appropriate actions focusing on improving information about the indications and benefits of vaccines, strengthening the importance of treating physicians’ recommendations and promoting knowledge among physicians, patients and their close contacts should be encouraged.

**Patient Consent**

Not applicable (there are no patient data)

**Disclosure of Interest**

None Declared

### Services and pathways of care

#### P359 Evaluation of the impact of an advanced nurse practitioner/physiotherapy led clinic on waiting list reduction and access to timely to care in a paediatric rheumatology service

##### Norma O'keeffe, Kelly Robinson, Clodagh Lowry

###### Rheumatology, CHI Temple Street, Dublin, Ireland

####### **Correspondence:** Norma O'keeffe

*Pediatric Rheumatology 2024*, **22(2)**: PReS24-ABS-1697

**Introduction:** Paediatric Rheumatology involves caring for children with inflammatory and non-inflammatory musculoskeletal disease. Children referred with non-inflammatory pain often experience impaired function and mobility, which impacts their physical, psychological, social and educational performance.

In Ireland, hospital waiting lists are very long with patients waiting an unacceptably longtime for care which can result in delayed treatment and suboptimal outcomes for newly referred patients (1). In 2021, our Rheumatology Service had 318 new patient referrals awaiting an initial review with some waiting over 4 years.

**Objectives:** Implement changes to streamline care, enhance efficiency and improve patient outcomes by targeting 3 key areas to reduce waiting lists and times:

1. Assessment of current activity including waiting lists, capacity etc.
2. Update care pathway to reflect the extended scope of practice of advanced nurse practitioners (ANP) and specialist physiotherapists.
3. Adopt a new model of care that schedules these non-inflammatory referrals to an ANP/Physiotherapist led clinic rather than a consultant clinic.

**Methods:** After stakeholder engagement meetings and analysing patient referrals, we offered reviews in an ANP/Physiotherapist-led clinic for new referrals categorized as non-inflammatory.

We implemented plan-do-study-act (PDSA) cycles to facilitate change, focusing on continuous process improvement. By utilizing clinic data to monitor adjustments, we achieved shorter waiting lists and enhanced patient outcomes.

**Results:** Since its establishment of an ANP/ Physiotherapy-led clinic in October 2022, wait times for new rheumatology referrals have reduced from four to two years, with the wait for non-inflammatory referrals reduced to one year. 145 patients have been seen in the clinic in 18 months; 14 required a follow-up review with the consultant, 44 were discharged to their GP, and the remainder were scheduled for a follow-up in an ANP and/or physiotherapy clinic.

**Conclusion:** By triaging new referrals where non-inflammatory conditions are reviewed in an ANP/ Physio-led clinic, we demonstrated an effective use of resources that enhances patient care, streamlines our referral process, while significantly reducing waiting times from four to two years and improving patient outcomes, highlighting our ongoing commitment to delivering quality healthcare to our patients.

**Patient Consent**

Not applicable (there are no patient data)

**Disclosure of Interest**

None Declared

### Services and pathways of care

#### P360 Empowering coping strategies for JSLE: the role of care-professionals in promoting well-being

##### Jana Mattei, Martina Kadoke, Heidi Rummel, Lea Höfel

###### Kinderklinik Garmisch-Partenkirchen gGmbH, Garmisch-Partenkirchen, Germany

####### **Correspondence:** Jana Mattei

*Pediatric Rheumatology 2024*, **22(2)**: PReS24-ABS-1171

**Introduction:** Systemic Lupus Erythematosus (SLE) is an autoimmune chronic inflammatory multisystemic disease. The main psychosocial symptoms are pain, anxiety, insecurity, fatigue and sleep disorders, which, in addition to other symptoms, can trigger further stress reactions.

**Objectives:** Promoting individual well-being and improving the quality of life are important for children and adolescents with jSLE, as this has impact on disease progression and thus on further development. Coping strategies can reduce or prevent stress reactions and improve self-efficacy.

Various therapeutic concepts in pediatric rheumatology (e.g. the “Garmischer Therapiekonzept”) have proven multiprofessional collaboration to be fundamental for successful treatment.[i]

The aim was to identify disease management strategies specifically attributed to the nursing sector, to categorize them in order to specify issues for future development.

**Methods:** A structured and an integrative review was carried out including the PubMed and LIVIVO databases. The conducted data have been analyzed and stratified using a model defining five categories. In addition we screened for studies demonstrating positive effects of coping strategies provided by care-givers on the course of the disease.

**Results:** A total of 3280 hits were checked for relevance, quality and suitability.

Results of 19 implemented studies show a variety of coping strategies and prove the assumption that individual coping strategies tailored to the needs of the patient are crucial for success. 5 studies have defined the influence of different dimensions. Both the number of categories and the content varied, although overlaps could be identified. om these findings, an overarching structure was derived:

1. Cognitive
2. Emotional
3. Motor/sensory
4. Social
5. Self-esteem regulating

In each of these categories there are measures that can be implemented specifically by nursing staff. In this way, they can complement and support the therapies of the other professional groups.

In addition, 16 studies suggest that coping strategies are tailored to improve specific disease symptoms, with most strategies described for pain.

**Conclusion:** The results of the literature search make it clear that there are a number of possible influences on coping with the disease and that nurses must be aware of the dimensions that need to be taken into account.

Nurses should offer patients strategies from different categories. Increased knowledge of different strategies expands the individual range of coping options.

The classification of coping strategies into five categories also shows that collaboration in multi-professional teams is fundamental to ensure holistic therapy and to fully utilize the potential of coping strategies in jSLE.

**Patient Consent**

Not applicable (there are no patient data)

**Disclosure of Interest**

J. Mattei Employee with: low, M. Kadoke Employee with: low, H. Rummel Employee with: low, L. Höfel Employee with: low

**Reference**

1. Haas, J.-P. (2021) Das Garmischer Modell als Beispiel für ein interdisziplinäres Therapiekonzept. In: Kinder- und Jugendrheuma, Chronische Schmerzen bei jungen Menschen, Wir können was tun! Ein Ratgeber für Eltern, Angehörige und Patienten, 3. Auflage, Kinderklinik Garmisch-Partenkirchen gGmbH (Hrsg.), 81-82

### Services and pathways of care

#### P361 Paediatric rheumatology education on a global level – where are we now? A survey study across the world

##### Sonia Melo Gomes^1, 2^ on behalf of PReS-EMERGE Global Affairs Subcommittee, Erdal Sag^3^ on behalf of PReS-EMERGE Global Affairs Subcommittee, Reiko Yatabe^4^ on behalf of PReS-EMERGE Global Affairs Subcommittee, Vignesh Pandiarajan^5^ on behalf of PReS-EMERGE Global Affairs Subcommittee, Lujain Alahmadi^6^ on behalf of PReS-EMERGE Global Affairs Subcommittee, Jordi Anton^7^, Marija Jelusic^8^ and PReS-EMERGE Global Affairs Subcommittee

###### ^1^Rheumatology, Great Ormond Street Hospital; ^2^Rheumatology, Great Ormond Street Hospital, London, United Kingdom; ^3^Rheumatology, Hacettepe University, Ankara, Türkiye; ^4^Rheumatology, Tokyo Metropolitan Children’s Medical Center, Tokyo, Japan; ^5^Rheumatology, Postgraduate Institute of Medical Education and Research, Chandigarh, India; ^6^Rheumatology, King Saud Bin Abdulaziz University for Health Sciences, Riyadh, Saudi Arabia; ^7^Rheumatology, Hospital Sant Joan de Déu, Barcelona, Spain; ^8^Rheumatology; University of Zagreb School of Medicine, Zagreb, Croatia

####### **Correspondence:** Sonia Melo Gomes

*Pediatric Rheumatology 2024*, **22(2)**: PReS24-ABS-1437

**Introduction:** Paediatric rheumatology (paed-rheum) is a relatively young subspecialty and practices regarding education and access to training vary widely around the world.

**Objectives:** To understand how training and access to the subspecialty varies in different countries and uncover unmet needs in training and continuous education.

**Methods:** An online survey with 39 questions was distributed through official PReS/EMERGE social media, mailing lists and by country representatives of the EMERGE Global Affairs Subcommittee (Dec23-Apr24). Descriptive statistics were performed using MSforms software and excel.

**Results:** 503 responses were received from 72 countries (339-F/164-M; 232(46%) <40y); 294 participants were certified specialists (58.4%) and 108 (21.5%) fellows/trainees, mostly from a paediatric background (445; 88.5%). Paed-rheum was recognised as a subspecialty in the country of 76.5% of responders (385) and only 57% (287) had a formal training program; 186 (37%) trained abroad, but for 299 (60%) funding was a main limitation.

Of the 238 (47.3%) in training/finished in the last 5y, 118 (49.6%) were very satisfied, but lack of research projects and technical procedures (13/40 each) were limitations; clinical overload (10/51) and lack of protected time for learning (12/51) were the most common causes for dissatisfaction. In terms of time for research during training, 161/238 had either no time or <25%; only 33/238 thought they had enough time for research; patient overload (139/238) and lack of funding (87/238) were the most frequently identified limitations. Overall, participants identified ultrasound skills (325; 64.6%), training in image interpretation (305; 60.6%) and more time for research (300; 59.6%) as the main ways to improve training.

For continuous professional development, case discussion with experts (275; 54.8%), practical training (221; 44%) and congresses (208;41.4%) were the most valued, but many do not have access to departmental (225; 45%) or alternative sources of funding (217; 43%) for their educational needs. Social media was used by most responders (79%), including for professional development (163; 32.4%), whilst 92 (18.2%) have separate personal and professional accounts.

**Conclusion:** Paed-rheum is still not officially recognised as a subspecialty in the countries of 23.5% of the responders and many more do not have access to a formal training program, highlighting asymmetries even within Europe. However, biases may exist as responses in this study reflect personal opinions. Lack of funding is a general concern, affecting training abroad, research opportunities and continuous development. Social media is becoming increasingly popular for professional development and could be used to tackle disparities. This study also underlines several ways in which training could be improved. Efforts to promote harmonization in paed-rheum training and continuous education should be encouraged and developed.

**Patient Consent**

Not applicable (there are no patient data)

**Disclosure of Interest**

None Declared

### Services and pathways of care

#### P362 Challenges of pediatric rheumatology at the resource-constrained himalayan regions– lone and unseen efforts

##### Dharmagat Bhattarai ^1^, Aaqib Z. Banday^2^, Pratap K. Patra^3^, Phub Tenzin^4^

###### ^1^Pediatric Immunology & Rheumatology, Advanced Centre for Immunology & Rheumatology; Om Hospital and Research Centre, Kathmandu, Nepal; ^2^Pediatrics, Government Medical College, Srinagar; ^3^Pediatrics, All India Institute of Medical Sciences, Patna, India; ^4^Pediatrics, Jigme Dorji Wangchuk National Referral Hospital, Thimphu, Bhutan

####### **Correspondence:** Dharmagat Bhattarai

*Pediatric Rheumatology 2024*, **22(2)**: PReS24-ABS-1701

**Introduction:** Pediatric rheumatological diseases (PRDs) including immune dysregulation disorders (IDDs) are increasingly being diagnosed with accuracy in resource-limited Himalayan regions due to the availability of a single subspecialist.

**Objectives:** To describe the profile of patients diagnosed with PRDs/IDDs in Nepal and the surrounding Himalayan regions during 2020-2024

**Methods:** Records of all patients diagnosed with PRDs/IDDs at a tertiary care center in Nepal (including children referred from surrounding Himalayan regions like Bhutan, and North-East India) from August 2020 to February 2024 were analyzed. The pediatric rheumatologist, [lead author (DB)] has examined and diagnosed all cases. Diseases were diagnosed based on internationally acclaimed criteria and genetic analyses.

**Results:** A total of 1175 children with PRDs (M: F = 1.4:1) and 114 children with IDDs were diagnosed during the study period. Genetic analysis was done on 69 patients with monogenic rheumatic/immune dysregulation disorders. Most patients had come after multiple admissions (1-94 times) before patient met the only pediatric rheumatologist in the country. Most children were misdiagnosed as tuberculosis, infections, septic arthritis, allergy, dermatitis, muscular dystrophy, malnutrition, or asthma. About 1/3^rd^ of children were wrongly commenced on steroids without evidence. The diagnostic profile of the patients includes various common autoimmune and autoinflammatory disorders to rare IDDs (e.g., *ARPC1B* deficiency). Monogenic autoinflammatory disorders were diagnosed in 44 patients. Some patients succumbed before the scope of diagnosis. Stem cell transplantation is done in 4 children. Commonly diagnosed PRDs included chronic arthritis, connective tissue disorders, vasculitis, autoinflammatory diseases, and reactive diseases.

**Conclusion:** With a single subspecialist, our is the first report of proven PRDs in Nepal. Skepticism about the existence of PRDs and lack of referrals may lead to the missing of these serious illnesses. Logistic constraints coupled with a lack of awareness of IEIs accounted for missed diagnoses and poor outcomes in resource-limited settings.

**Trial registration identifying number:** Not applicable

**Date of birth::** 12.08.1981

**Patient Consent**

Yes, I received consent

**Disclosure of Interest**

None Declared

### Services and pathways of care

#### P363 Quality improvement lessons in pediatric rheumatology program in Qatar

##### Farah Shaya ^1^, Buthaina Al Adba^1^, Sharon Bout-Tabaku^1, 2^

###### ^1^Pediatrics, Sidra Medicine; ^2^Pediatric, Weill Cornell Medicine-Qatar, Doha , Qatar

####### **Correspondence:** Farah Shaya

*Pediatric Rheumatology 2024*, **22(2)**: PReS24-ABS-1715

**Introduction:** Children with Juvenile Idiopathic Arthritis (JIA) have better disease outcomes with current medications available, yet only 50-70% reach clinically inactive or low disease activity. Quality improvement (QI) processes are dynamic ways to identify gaps, implement goals and impact variability, to improve outcomes. Organizations worldwide have identified QI goals for JIA that are important and feasible in their settings. Our practice opened in 2018, as the sole provider of pediatric rheumatology services in Qatar and we identified areas of QI.

**Objectives:** We describe four QI goals: toxicity monitoring for disease-modifying antirheumatic drugs (DMARD’s), tuberculosis screening prior to starting biologics, joint injection safety, and uveitis screening.

**Methods:** We built measures retrieved from the electronic medical record (EMR). From 2018 we screened for drug toxicity among patients receiving methotrexate or leflunomide within 3-4 month of receiving methotrexate. From September 2021 to 2022, we added new QI goals that could not be captured by the EMR. We engaged nursing staff to monitor tuberculosis screening prior to starting biologics by ensuring completion of QuantiFERON gold or PPD testing, and/or chest x-ray. We developed a joint injection tracker to capture the number of patients having a procedure within 2 weeks of the referral, and to monitor long and short-term side effects. For uveitis screening and monitoring, we measured the percent of eligible JIA patients with up to date screenings over the previous 6 months as a baseline. In January 2022, we implemented a monthly combined clinic Ophthalmology and Rheumatology clinic. Measures were reviewed quarterly.

**Results:** Between 2019 and 2024, laboratory monitoring for DMARD’s showed that 96% of children receiving methotrexate or leflunomide, were screened for toxicity every 3-4 months. However, during a COVID 19 surge, in the first quarter of 2021, 83% were screened. Between 2021 and 2022, 100% of patients had tuberculosis screening prior to starting biologics. Timely performance of joint injections was variable with a median of 53% done within 2 weeks in the year of 2022. In 2023, the median of timely performed joint injections was 77%. From December 2021 to April 2024, among 63 patients we measured a total of 6% experiencing long-term side effects and 10% experiencing short-term side effects. Prior to the combined clinics, 96% of patients had up to date eye screening visits. After starting combined clinics, adding patient specific diagnosis, and ANA, and HLA B27 results, there was an increase in the eye screening over four quarters (Jun-2022 to Mar-2023), reaching a median of 98%. After a change in provider availability, the eye screening visits dropped to a median of 96% over the last 4 quarters.

**Conclusion:** QI projects can be successful and should be started early for patient safety. Most of our goals were successful and were maintained at over 90% except for the timeliness of joint injection procedures. The EMR captured one of our QI goals whereas other goals were not easily extractable from the EMR. The successful QI measures required the engagement of nursing staff to maintain the workflow. Finally, the implementation of a monthly, combined clinic is an effective way of ensuring timely uveitis screening and decreases the burden of total number of clinic appointments. Sustainability is crucial and finding ways to automate cumbersome workflows are needed.

**Patient Consent**

Not applicable (there are no patient data)

**Disclosure of Interest**

None Declared

**Reference**

1. Bingham CA,et al, Pediatric Rheumatology Care and Outcomes Improvement Network. Pediatric Rheumatology Care and Outcomes Improvement Network's Quality Measure Set to Improve Care of Children With Juvenile Idiopathic Arthritis. Arthritis Care Res (Hoboken). 2023 Dec;75(12):2442-2452.

### Services and pathways of care

#### P364 Changing perspectives of pediatric rheumatic diseases through community-directed interventions in resource-limited settings

##### Dharmagat Bhattarai ^1^, Aaqib Z. Banday^2^, Pratap K. Patra^3^, Asmita Neupane^4^

###### ^1^Pediatric Immunology & Rheumatology, Advanced Centre for Immunology & Rheumatology; Om Hospital and Research Centre, Kathmandu, Nepal; ^2^Pediatrics, Government Medical College, Srinagar; ^3^Pediatrics, All India Institute of Medical Sciences, Patna, India; ^4^Statistics, Advanced Centre for Immunology & Rheumatology, Kathmandu, Nepal

####### **Correspondence:** Dharmagat Bhattarai

*Pediatric Rheumatology 2024*, **22(2)**: PReS24-ABS-1704

**Introduction:** Pediatric Rheumatic diseases (PRDs) remain undiagnosed in resource-limited settings. With the availability of subspecialists, perspectives have changed in recent times.

**Objectives:** To study the effect of various community-directed interventions (CDIs) on the diagnosis of PDDs.

**Methods:** From August 2020 to December 2021, the baseline diagnosis rate of PRDs was assessed in Nepal. Web-based and physical surveys were conducted to assess the various factors affecting PRDs. CDIs were implemented from January 2022 to May 2023. Applied interventions (number) included immunology-oriented national health camps (6), social media promotions (38), local language articles (9), audio-visual clips (17), interviews (9), talks (3), physicians’ class (18), society registration (4), and society activities (many). The rate of diagnosis of PRDs was reassessed. Post-intervention web-based surveys were conducted to assess the change in awareness in the second phase.

**Results:** Lead author DB has revolutionized awareness efforts for PRDs including immune dysregulation disorders. Nepalese patient societies like NIAPIDS, NePOPII, HAEi, and Lupus Nepal have helped with the single doctor’s movement. One hundred and fourteen patients (M: F=1:1.4) were diagnosed with PRDs from August 2020 to December 2021. Genetic diagnosis was established in 12 patients. Disease-modifying drugs were given in around 62% of cases. Out of 18 needful patients, biologics was initiated in 2 patients only. Hematopoietic stem cell transplantation (HSCT) could not be expedited for any patients. Baseline awareness regarding PRDs among the public and physicians was 2.1% and 14.7%, respectively.

From January 2022 to February 2024, 1175 patients (M: F=1:1.5) with PRDs were diagnosed. Genetic confirmation was established in 147. More than 90% of children could be started with disease-modifying agents. Biologics started in 32 children. Four children underwent HSCT. Post-interventional awareness regarding PRDs among the public and physicians was 7.1% and 39.2% respectively. In this period, we also diagnosed some rare and newly identified autoimmune and autoinflammatory disorders. Advocacy for patients’ rights with the government began in the interventional period.

**Conclusion:** Notable increments in the diagnosis of PRDs and awareness among physicians were observed after CDIs. The study points to the need for further interventions for awareness of IEIs/IDDs. Logistic constraints coupled with a lack of awareness of IEIs among laity and pediatricians accounted for missed diagnoses.

**Trial registration identifying number:** Not applicable

**Date of birth::** août 12, Y

**Patient Consent**

Yes, I received consent

**Disclosure of Interest**

None Declared

### Services and pathways of care

#### P365

##### **Advancing pediatric rheumatology ambulatory care: introducing the reumpedic patient-centered model**

###### Joanna Wojtowicz

####### Pediatric Rheumatology, Reumapedic, Warsaw, Poland

*Pediatric Rheumatology 2024*, **22(2)**: PReS24-ABS-1736

**Introduction:** Delayed diagnosis and patient non-compliance pose significant challenges in treating pediatric rheumatic diseases. However, properly organized pediatric rheumatology ambulatory care can effectively address these obstacles. REUMPAPEDIC, an outpatient clinic in Warsaw, Poland, has developed a comprehensive and modern patient-centered system of care for pediatric rheumatic patients.

**Objectives:** This study aims to present the REUMPAPEDIC model of ambulatory care in pediatric rheumatology, emphasizing its modern strategies for quick diagnosis and improved patient adherence.

**Methods:** Through a detailed analysis of REUMPAPEDIC's outpatient care model, we explore its organizational strategies, interdisciplinary collaborations, and patient engagement efforts, taking into account local/national conditions.

**Results:** Despite public financing ensuring high-quality specialist care for children with rheumatic diseases in Poland, long waiting times for specialist care, including the initial visit, remain a challenge. Privetely-funded REUMAPEDIC provides comprehensive care for children with suspected and diagnosed rheumatic diseases, handling approximately 200 patient visits (+ online meetings) per month encompassing diagnosis, treatment, and holistic care aspects such as infections, vaccinations, and mental well-being. Swift diagnosis is facilitated through streamlined processes, with rapid access to specialized care (typically within a week for a pediatric rheumatologist visit). Thorough differential diagnosis is enabled by appropriate visit length and a post-visit structured analysis process. Tailored treatment plans, educational initiatives, and accessible online communication channels enhance patient adherence. Collaboration between pediatric rheumatologist and other consultants, coupled with access to high-quality laboratory and imaging diagnostics, is assured. Patient feedback is actively solicited.

**Conclusion:** The REUMPAPEDIC modern model of ambulatory care in pediatric rheumatology represents a significant advancement in addressing the challenges of delayed diagnosis and patient non-adherence in Poland. By prioritizing quick diagnosis and promoting patient adherence through interdisciplinary collaborations and patient engagement efforts, Reumpapedic exemplifies patient-centered approaches in optimizing outcomes for children with rheumatic diseases.

**Patient Consent**

Not applicable (there are no patient data)

**Disclosure of Interest**

None Declared

### Services and pathways of care

#### P366 Paediatric rheumatic diseases and vaccinations: a promising alliance

##### Maria Cristina Maggio^1^, Carla Gilotta^1^, Antonia Cantavenera^1^, Santi Cancila^1^, Rosario Bonadia^1^, Anna Di Salvo^1^, Seyed Milad Mousavì^1^, Clotilde Alizzi^2^, Giovanni Corsello^1^

###### ^1^PROMISE "G. D'Alessandro", University of Palermo, ^2^UOC Pediatria generale, Children Hospital "G. Di Cristina", ARNAS Palermo, Palermo, Italy

####### **Correspondence:** Maria Cristina Maggio

*Pediatric Rheumatology 2024*, **22(2)**: PReS24-ABS-1599

**Introduction:** Pediatric rheumatic diseases and autoinflammatory diseases (AID) are chronic conditions, often burdened by comorbidities and reduced quality of life. Immunosuppressive drugs, DMARDS (methotrexate), glucocorticoids and biological drugs can limit the immune response to vaccines and, also, contraindicate their administration, particularly in the case of live vaccines. Recent studies, however, documented the safety of the latter in patients taking low doses of glucocorticoids and methotrexate, highlighting the appropriate timing for vaccine dose. Guidelines propose to guarantee a variable interval, based on the type of treatment, for the administration of inactive vaccines to promote a better immunological response and to guarantee clinical remission, the optimal condition to carry out the vaccine. For patients receiving immunosuppressive therapy, an additional booster dose is suggested, without any modification of the therapeutic scheme, at least 3 months later.

**Objectives:** We supported the importance of vaccinations for patients with rheumatic diseases or AID and we promoted anti-flu and anti-SARS-CoV-2 vaccinations.

**Methods:** We analyzed a series of 103 patients with rheumatological pathologies and AID, in follow-up at the "Pediatric Rheumatology" unit of the P.O. “G. Di Cristina” of Palermo, and we divided them into 4 groups, evaluating their adherence to the seasonal flu and SARS-CoV-2 vaccinations: 1) 56 patients with juvenile idiopathic arthritis (JIA); 2) 29 patients with AID (FMF, TRAPS, MVK, CAPS, etc); 3) 14 patients with other rheumatological pathologies (SLE, recurrent uveitis, vasculitis, Behçet's disease); 4) 4 patients with MIS-C, Kawasaki disease.

**Results:** The vaccination campaign did not obtain the expected results: 15 patients (14.6%) received both vaccinations; 49 patients (47.6%) were vaccinated against COVID-19; 29 patients (28.2%) against seasonal flu and 40 patients (38.8%) have not been vaccinated.

Patients in group 1 and group 4 showed low compliance with both vaccinations. Some patients were not vaccinated against SARS-CoV-2 because contracted the infection.

Patients in group 2 and group 3 adhered to the anti-flu and anti-SARS-CoV-2 vaccinations in a low percentage because the fear of recurrence of the underlying pathology influenced the parents' choice.

**Conclusion:** It is necessary to support families in the decision to join the vaccination campaigns, with exhaustive information that can make known the benefits of vaccination in terms of quality of life and tolerability. The mistrust and fear which, in most cases, represent the primum movens of the failure to carry out the anti-flu and anti-SARS-CoV-2 vaccines, are not currently supported by studies which support the risks of reactivations or exacerbation of the disease.

In this regard, it is important to promote adequate vaccination coverage, not only for patients, but also for the entire family unit. We need to sensitize families to make a conscious choice and to eliminate misinformation should represent a primary objective to prevent, protect, treat!

**Patient Consent**

Yes, I received consent

**Disclosure of Interest**

None Declared

### Spondyloarthritis (SpA) and enthesitis related arthritis (ERA)

#### P367 Outcome of spondyloarthritis/ enthesitis related arthritis treated with sulfasalazine: experience from a single center

##### Jahnavi Shrivastava ^1, 2^, Anu Maheshwari^2^, Deonath Mahto^2^

###### ^1^Pediatrics, Lady Hardinge Medical College, New Delhi; ^2^Pediatrics, Lady Hardinge Medical College, Delhi, India

####### **Correspondence:** Jahnavi Shrivastava

*Pediatric Rheumatology 2024*, **22(2)**: PReS24-ABS-1365

**Introduction:** The 2019 American College of Rheumatology (ACR)/ Arthritis Foundation Guidelines for the management of Juvenile Idiopathic arthritis (JIA) strongly recommend the use of anti-tumor necrosis factor (TNF) agents in those patients with active sacroiliitis [1]. However, the use of these drugs in low and middle income countries is limited because of logistical issues involving availability, high costs and a high burden of tuberculosis [2]. This study was planned to assess the effectiveness of conventional drugs, namely sulfasalazine, in the treatment of spondyloarthritis/Enthesitis related arthritis (SSA/ERA).

**Objectives:** To evaluate the effectiveness of sulfasalazine in the primary treatment of newly diagnosed SSA/ERA patients.

**Methods:** This prospective cohort study was conducted over a period of four years from 2019 to 2023 with due approval from the institutional ethics committee. 112 patients diagnosed with SSA/ERA according to the ILAR criteria [3] were enrolled into the study after taking consent from the parent/guardians. Patients with chronic renal, hepatic and cardiac disease were excluded. Demographic parameters, HLA-B27, JADAS-27 scores [4] and radiological assessment of sacroiliitis were recorded at enrolment. Patients with MRI-proven active sacroiliitis (n=100) were started on sulfasalazine at a dose of 30-50 mg/kg/day. Over a follow up period of six months, they were assessed for disease activity scores and radiological improvement. Those with persistent moderate/high disease activity (JADAS-27 score>=6) were shifted to biological therapy.

**Results:** Of the 112 patients enrolled, 75 (66.96%) were males. The median age of the study population was 9 years [IQR: 7.8-16.9 years]. 79 patients (70.5%) were HLA-B27 positive. 100 patients had evidence of sacroiliitis on MRI, rest were excluded from the analysis. Out of this 85 patients had bilateral involvement while the remaining 15% had unilateral involvement at diagnosis. Median JADAS-27 scores at baseline were 14.00 [IQR: 6.00-20.25]**.** At 6 months of treatment, 51 patients had moderate/high disease activity scores and were shifted to etanercept (51%). The remaining 49 patients showed good response to sulfasalazine and were continued on the same regimen.

**Conclusion:** While biologicals based therapy is currently the recommended standard of care for SSA/ERA patients, conventional therapy with sulfasalazine must not be written off as ineffective. A good trial of sulfasalazine for up to six months is warranted, especially in settings where availability or costs are limiting factors, along with those situations where biological therapy is otherwise contraindicated.

**Date of birth::** juin 09, Y

**Patient Consent**

Yes, I received consent

**Disclosure of Interest**

None Declare

**References**

1. Ringold S, Angeles‐Han ST, Beukelman T, Lovell D, Cuello CA, Becker ML, et al. 2019 American College of Rheumatology/Arthritis Foundation Guideline for the Treatment of Juvenile Idiopathic Arthritis: Therapeutic Approaches for Non‐Systemic Polyarthritis, Sacroiliitis, and Enthesitis. Arthritis Care Res. 2019;71:717-734.
2. Caporali R, Pallavicini FB, Filippini M, Gorla R, Marchesoni A, Favalli EG, *et al*. Treatment of rheumatoid arthritis with anti-TNF-alpha agents: a reappraisal. Autoimmun Rev. 2009;8:274-280.
3. Petty RE, Southwood TR, Manners P, Baum J, Glass DN, Goldenberg J, et al. International League of Associations for Rheumatology. International League of Associations for Rheumatology classification of juvenile idiopathic arthritis: second revision, Edmonton, 2001. J Rheumatol. 2004;31:390-392.
4. Consolaro A, Ruperto N, Bazso A, Pistorio A, Magni-Manzoni S, Filocamo G, *et al*. Paediatric Rheumatology International Trials Organisation. Development and validation of a composite disease activity score for juvenile idiopathic arthritis. Arthritis Rheum. 2009;61:658-666.

### Spondyloarthritis (SpA) and enthesitis related arthritis (ERA)

#### P368 Evaluation of disease severity and treatment responses in patients with enthesitis-related arthritis

##### Sema Aydın ^1^, Zeynep Balık^2^, Yağmur Bayındır^2^, Dilara Ünal^2^, Emil Aliyev^2^, Veysel Çam^2^, Hülya Ercan Emreol^2^, Erdal Sağ^2^, Ezgi Deniz Batu ^2^, Halide Özge Başaran^2^, Yelda Bilginer^2^, Seza Özen^2^

###### ^1^Department of Pediatrics; ^2^Department of Pediatric Rheumatology, Hacettepe University, Ankara, Türkiye

####### **Correspondence:** Sema Aydın

*Pediatric Rheumatology 2024*, **22(2)**: PReS24-ABS-1756

**Introduction:** Enthesitis-Related Arthritis (ERA) is a chronic inflammatory arthritis classified as a type of Juvenile Idiopathic Arthritis(JIA), constituting 15-20% of all cases(1). Sacroiliac joints are often involved (2).

**Objectives:** The objective of this study is to assess the disease severity and treatment responses in ERA patients.

**Methods:** This is a single-center retrospective cohort study evaluating ERA patients (aged 0-18 years) diagnosed between 2009-2023.The demographic and disease characteristics of patients, disease activity scores were recorded at the initiation of treatment, 12th weeks and current visits.

**Results:** Of the 118 patients, 72% were male and 28% were female. The median age at diagnosis was 13,3 (5,4-17,6) years. In the family history, 10,2% had ankylosing spondylitis and 2,5% had inflammatory bowel disease. As for comorbidities, 12,7% had Familial Mediterranean Fever (FMF), 4,2% had chronic recurrent nonbacterial osteomyelitis (CRMO), 3,4% had Crohn's disease and 1,7% had both FMF and Crohn's disease. HLA-B27 was positive in 51,7% of all cases, in 40% of FMF, in 40% of CRMO and in 25% of Crohn's patients.

At diagnosis, axial involvement and enthesitis were present in 55,9% and 49,2%, respectively. ESR was elevated in 44,1% and CRP in 83,1% of patients. At onset, median values for the Bath Ankylosing Spondylitis Dissease Activity Index (BASDAI) were 4 (0,8-8), for the Juvenile Arthritis Disease Activity Score (JADAS) were 14 (3,7-26,7) and for the Juvenile Spondyloarthritis Disease Activity Index (JSpADA) were 3 (1-7). The scores at diagnosis were found to be correlated in the repeated measures ANOVA analysis.

All patients were on Nonsteroidal Anti-Inflammatory Drugs(NSAIDs), while 46,6% sulfasalazine, 70,3% methotrexate, 64,4% anti-TNF agents, 39,8% (short-course) systemic steroids, 17,8% intra-articular steroids and 2 patients received secukinumab. The median values for BASDAI, JADAS, and JSpADA at the 3rd month were 1,6 (0-4,9), 5 (0-14,6), 1,5 (0-5) for patients taking NSAIDs (n=26); 1,4 (0-5,7), 4 (0-19), 1 (0-5) for those taking sulfasalazine (n=33); and 2 (0-6,4), 6 (0-24,1), 1,8 (0-7) for patients receiving methotrexate (n=79), respectively. For patients receiving anti-TNF agents, median activity scores at the 3rd month were all zero.

In current evaluations, ongoing treatments were as follows: NSAIDs 8,5%, sulfasalazine 4,2%, methotrexate 2,5% and anti-TNF agents in 68,4%.

**Conclusion:** The three acitivty scores had a good correlation. Although more patients achieved remission with anti-TNF agents, a group of patients responded to DMARDs.

**Date of birth::** décembre 2

**Patient Consent**

No, I have not received consent

**Disclosure of Interest**

None Declare

**References**

1. Petty RE, Southwood TR, Manners P, Baum J, Glass DN, Goldenberg J, et al. International League of Associations for Rheumatology classification of juvenile idiopathic arthritis: second revision, Edmonton, 2001. J Rheumatol. 2004;31(2):390-2.
2. Shen CC, Yeh KW, Ou LS, Yao TC, Chen LC, Huang JL. Clinical features of children with juvenile idiopathic arthritis using the ILAR classification criteria: a community-based cohort study in Taiwan. J Microbiol Immunol Infect. 2013;46(4):288-94.

### Spondyloarthritis (SpA) and enthesitis related arthritis (ERA)

#### P369 Unmet needs in Juvenile spondyloarthritis in the real world

##### Maria Martha Katsicas, Luciana Vasconcellos, Janet Manrique, Juan Portigliatti, Emilia Puentes, Luciana Lancioni, Giselle Villarreal

###### Rheumatology, Hospital JP Garrahan, CABA, Argentina

####### **Correspondence:** Maria Martha Katsicas

*Pediatric Rheumatology 2024*, **22(2)**: PReS24-ABS-1570

**Introduction:** Juvenile spondyloarthritis is a group of diseases that includes enthesitis -related arthritis (ERA). Early recognition and treatment, as well as the effectiveness of biologic agents could help prevent development of ankylosing spondylitis over the course of the disease. However, despite treatment with TNF inhibitors, there are patients that show partial or no response, especially in the axial component.

**Objectives:** To assess the effectiveness of anti- TNF treatment and identify unmet needs in a cohort of patients with ERA

**Methods:** We conducted a retrospective review of prospectively collected data. We included patients with ERA (according to ILAR criteria) who were continuously treated with anti-TNF agents for ≥ 12 months. Demographic, clinical, biochemical and imaging variables were investigated. MRI of the sacroiliac joints (SIJ) was performed before starting anti-TNF therapy (baseline) and during the follow up (> 12 months after anti-TNF treatment). The SIJ were examined using T1-weighted images, T2 fat-suppressed and STIR. The Spondyloarthritis Research Consortium of Canada sacroiliac joint score (SPARCC-SIS) was determined by two pediatric radiologists. Treatment with TNF-blockers and exposure time were recorded. Outcome measures included: pain score (0-10), wellbeing according to the patient using a visual analogue scale (VASp, 0-10), disease activity according to the physician (VASphy, 0-10), JADAS-10 and JSpADA. Functional capacity was assessed by CHAQ. Inactive disease was evaluated based on Wallace criteria. Inactive disease based on JspADA index (inactive score=0) and SPARCCSIS score ≥2 was used as a surrogate for an MRI-sacroiliitis positive /active. Statistical análisis included: descriptive statistics and comparisons using Wilcoxon Signed Rank test

**Results:** Seventeen (53% male) patients fulfilled the inclusion criteria. The median age at onset of the disease was 7(range:2-14) The median time to start TNF-blocker therapy was 3 years (0.5-9) and disease duration was 8 years (range:2-14).Clinical features included : arthritis 100%, tarsitis 45%, lower back pain 35% , hip arthritis 29%, enthesitis 18%, uveítis 12% and dactylitis 12%. Morning stiffness had a median duration of 15 min (range:0-60). The MRI SPARCC-SIS was ≥2 in all patients. HLA-B27 was positive in 29%. The biologic treatment consisted of adalimumab for 9 patients (3 of then switched to Secukinumab) and etanercept for 8 patients (one of them switched to adalimumab). The median exposure to anti –TNF therapy was 36 months (range:12-108). The Outcome measures before and after TNF blocker treatment were: pain Score (0-10) median (range) 3(0-7.5) vs 0.5(0-5) p=0.046; VASp 3.25(0-9.5) vs 0 (0-6) p=0.016; VASphy 3.25(0-7.5) vs 0.5(0-6) p=0.0006; SPARCC-SIS 30±17.35 vs 12±7.61 p=0.0084; JADAS-10 12 (0.5-28) vs 1.2 (0-10.2) =:0.009; JSpADA3(0.5-6)vs1(0-3)p=0.0096. CHAQ≥0.5 8(47%) vs 1(6%) p:ns. Inactive disease rates according to Wallace at last visit = 10 (58%) patients . SPARCC-SIS Median±SD 15±10.2. Inactive disease according to JSpA index =5 (29%) patients . SPARCC-SIS 6±7.82

**Conclusion:** Anti-TNF blockers demonstrated effectiveness, however, there is a subset of patients who remain active despite treatment.. Furthermore, patients with inactive disease still exhibited evidence of inflammatory SIJ according to SPARCC score. Future studies are needed to understand the need for early escalation treatment to prevent structural damage.

**Patient Consent**

Not applicable (there are no patient data)

**Disclosure of Interest**

None Declared

### Spondyloarthritis (SpA) and enthesitis related arthritis (ERA)

#### P370 Temporal trends in and associations with nonsteroidal anti-inflammatory drug (nsaid) prescription in adult and pediatric patients with inflammatory bowel disease

##### Adam Mayer^1^, Rui Xiao^2^, Andrew Grossman^3^, Meenakshi Bewtra^4^, Michael George^5^, Pamela Weiss^1^

###### ^1^Pediatric Rheumatology; ^2^Biostatistics; ^3^Pediatric Gastroenterology, The Children's Hospital of Philadelphia; ^4^Gastroenterology; ^5^Rheumatology, University of Pennsylvania, Philadelphia, United States

####### **Correspondence:** Adam Mayer

*Pediatric Rheumatology 2024*, **22(2)**: PReS24-ABS-1712

**Introduction:** The role of nonsteroidal anti-inflammatory drugs (NSAIDs) in the management of patients with inflammatory bowel disease (IBD) and IBD-associated arthritis is unclear in light of mixed data on intestinal safety, and United States national prescribing patterns in patients with IBD are unknown.

**Objectives:** The objectives of this study are to determine real-world use of prescription NSAIDs in patients with IBD across the age spectrum, including factors associated with NSAID prescription and how NSAID prescribing patterns have changed over time.

**Methods:** This is a retrospective cohort study from 2000-2022 in Optum’s de-identified Clinformatics® Data Mart Database. Children and adults with IBD and available pharmacy claims data were enrolled. NSAID and opioid prescriptions per calendar year were assessed. Descriptive statistics were used to assess differences in characteristics between adult vs. pediatric patients. Wilcoxon-Cruzick test of trend and generalized estimating equation models were used to evaluate trends in NSAID and opioid prescribing and assess characteristics associated with NSAID use.

**Results:** 361,025 patients met inclusion criteria of which 12,930 (3.6%) were <18 years old. 99,895 (27.7%) patients had at least one prescription NSAID during the study period. Adults were more likely than children to have 1 NSAID (28.1% vs. 14.9%, p<0.01) or opioid prescription (53.5% vs. 37.2%, p<0.01). They were also more likely to have ³1 diagnosis code for inflammatory arthritis, osteoarthritis, other joint pain not otherwise specified (NOS), or chronic pain (p<0.01 for all) irrespective of NSAID prescription. There was a significant decreasing trend in the proportion of patients prescribed NSAIDs over time (p<0.01). In the multivariable model, opioid prescription (OR 2.12, 95% CI 2.10-2.14), or a diagnostic code for inflammatory arthritis (OR 1.23, 95% CI 1.21-1.25), osteoarthritis (OR 1.56, 95% CI 1.54-1.58), or joint pain NOS (OR 1.59, 95% CI 1.57-1.61) had strong independent associations with NSAID prescription, while age <18 (OR 0.54, 95% CI 0.51-0.57) or 80 years (OR 0.69, 95% CI 0.67-0.71) at date of first IBD code were associated with significantly lower odds of NSAID prescription.

**Conclusion:** NSAID prescription in patients of all ages with IBD is common but fill patterns decreased over time. Pediatric patients were least likely to receive NSAIDs even though the comorbidity and side effect profile may be more favorable in this population.

**Patient Consent**

Not applicable (there are no patient data)

**Disclosure of Interest**

A. Mayer: None Declared, R. Xiao: None Declared, A. Grossman: None Declared, M. Bewtra Grant / Research Support with: Research funding from J&J, GlaxoSmithKline, Takeda and Iterative Health, Consultant with: Consulting fees from J&J, Pfizer and AbbVie, M. George Grant / Research Support with: Research contract from Janssen and GSK, Consultant with: Consulting fees from Pfizer, P. Weiss Consultant with: Site investigator for Pfizer and Abbvie Clinical Trials (Payment to institution), Consulting fees: Pfizer (payment to institution)

### Spondyloarthritis (SpA) and enthesitis related arthritis (ERA)

#### P371 Diagnostic and prognostic values of hla-b27 testing

##### Jean Jacques De Bruycker^1^, Raphael Kraus^1^, Marie-Paule Morin^1^, Julie Barsalou^1^, Fabien Touzot^1^, Elie Haddad^1^, Bilade Cherqaoui ^2^

###### ^1^Immuno-Rheumatology, CHU Sainte Justine, Montreal, Canada; ^2^Paediatrics, CHU Ambroise Pare, Boulogne-Billancourt, France

####### **Correspondence:** Bilade Cherqaoui

*Pediatric Rheumatology 2024*, **22(2)**: PReS24-ABS-1319

**Introduction:** The HLA-B27 allele is associated with enthesitis-related juvenile idiopathic arthritis (ERA), or juvenile spondyloarthritis (jSpA), leading to a broad prescription for HLA-B27 testing, which diagnostic and prognostic values are not well evaluated.

**Objectives:** We thus aimed to estimate the diagnostic and prognostic values of HLA-B27 testing in a large cohort of children followed in a paediatric centre.

**Methods:** In that purpose, we constituted a retrospective cohort of children tested for the HLA-B27 and followed at Sainte-Justine university paediatric hospital between 2018 and 2023. The prevalence of HLA-B27 according to the prescribing department and the final diagnosis were evaluated. Among patients with ERA/jSpA (ILAR criteria) and/or related pathology, the remission rate, the relapse rate and biotherapy use were notified according to HLA-B27 status.

**Results:** Only 39/397 [9.8%] of the tested children were HLA-B27(+), a rate comparable to its prevalence in Canadian healthy population. HLA-B27 prevalence was only increased when the test was prescribed in ophthalmology department (11/56 [19.6%], p=0.01). The most frequent diagnosis in this cohort was that of nonspecific arthralgia (127/397 [31.9%]), associated with a low level of HLA-B27(+) (7/127 [5.5%]). The prevalence of HLA-B27 was increased in patients with: psoriatic arthritis (3/10 [30%], p=0.04), JSpA (8/33 [24.2%], p=0.01), acute anterior uveitis (AAU) (12/61 [19.7%], p=0.02). In these patients with HLA-B27-related disorders, the remission rate, relapse rate, and use of methotrexate/biotherapies did not vary according to HLA-B27(+) status.

**Conclusion:** HLA-B27 testing does not seem to be a good diagnostic biomarker; its prescription should be restricted to the clearest phenotypes, especially AAU. HLA-B27 status does not appear to predict the prognosis of HLA-B27-associated pathologies, and therefore cannot be used to guide management.

**Patient Consent**

Yes, I received consent

**Disclosure of Interest**

None Declared

### Spondyloarthritis (SpA) and enthesitis related arthritis (ERA)

#### P372 Temporomandibular joint involvement in pediatric patients with inflammatory bowel disease-related arthritis

##### Ana K. Leos-Leija^1^, Carlos Valera-Ribera ^2^, Jordi Anton-Lopez^3^, Joan Calzada-Hernandez^3^, Juan M. Mosquera-Angarita^3^, Estibaliz Iglesias-Jimenez^3^, Andrea Zacarias-Crovato^3^, Violeta Bitterman^3^, Judith Sanchez^3^, Sonia Carriqui^3^, Nolvia Castillo^3^, Adaia Valls^4^, Emili Inarejos^5^

###### ^1^Pediatric Rheumatology, Hospital Regional Materno Infantil, Guadalupe, Mexico, ^2^Rheumatology, Doctor Peset University Hospital, Valencia, ^3^Pediatric Rheumatology, ^4^Maxillofacial surgery, ^5^Radiology, Hospitat Sant Joan de Déu, Barcelona, Spain

####### **Correspondence:** Carlos Valera-Ribera

*Pediatric Rheumatology 2024*, **22(2)**: PReS24-ABS-1205

**Introduction:** Arthritis is the most common extra-intestinal manifestation in patients with inflammatory bowel disease (IBD). The temporomandibular joint (TMJ) is frequently affected in children with juvenile idiopathic arthritis (JIA), and there are very few reports of its involvement in patients with IBD-related arthritis.

**Objectives:** To describe the demographic, clinical characteristics, and differences between pediatric patients with IBD-related arthritis with and without TMJ involvement.

**Methods:** We designed an observational retrospective study, enclosing a cohort of children with IBD-related arthritis, being followed for a maximum of 11 years (2012-2023). Demographic and clinical data were collected from clinical records. The variables and their association with TMJ involvement were evaluated. The statistical analysis was performed using SPSS 17.0 program.

**Results:** Twenty patients were included, 11 females (55%). The median age at the time of diagnosis was 7.5 years (IQR 6.75). Sixteen patients were diagnosed with Crohn’s disease, 3 of ulcerative colitis and 1 had indeterminate colitis. The median fecal calprotectin at diagnosis of the IBD was 2002mg/kg (IQR: 3773). The most common phenotype of Crohn’s disease was inflammatory (65%), followed by stenotizing (15%) and 2 patients with unknown subtypes. Nine patients (45%) developed gastrointestinal symptoms, whilst 2 patients (10%) debuted with gastrointestinal and articular symptoms concomitantly. Of our sample, 4 patients had suffered from TMJ arthritis. Three patients had unilateral involvement, and 1 patient had both TMJs. We did not find any of the studied variables associated with TMJ arthritis. Regarding the treatment, there were no statistically significant differences between the need for biologic treatment between the two groups (p=0.2), nor the number of biologic drugs used (p=0.148). The most used immunosuppressant in both groups was azathioprine (n=12), followed by methotrexate (n=9). The most used biological drug was adalimumab (n=17), followed by infliximab (n=5). During the following period, 75% of patients with TMJ affectation required intra-articular infiltration with glucocorticoids, whereas only 37.5% of patients with no TMJ affectation required it (p=0.217). Moreover, patients with TMJ arthritis had nearly double the number of infiltrations performed on them, although it did not reach statistical significance (p=0.8).

**Conclusion:** IBD-related arthritis can also affect the TMJ. We did not find any demographic or clinical data that may increase the risk of developing it. These patients can have a higher need for intra-articular glucocorticoid infiltration than other IBD-related arthritis patients.

**Patient Consent**

Yes, I received consent

**Disclosure of Interest**

None Declared

**References**

1. Rogler G, Singh A, Kavanaugh A, Rubin DT. Extraintestinal Manifestations of Inflammatory Bowel Disease: Current Concepts, Treatment, and Implications for Disease Management. Gastroenterology. octubre de 2021;161(4):1118-32.
2. Yıldız M, Haşlak F, Adroviç A, Şahin S, Barut K, Kasapçopur Ö. Juvenile spondyloartropathies. Eur J Rheumatol. 2021;9(1):42-49.

### Systemic JIA

#### P373 Still's disease-associated lung disease across lifespan: a multicentric study

##### Augusto Silva^1^, Carolina O. Matos^1^, Catarina Silva^2^, Diana B. Raimundo^3^, Helena Sousa^4^, Helena Assunção^5^, Inês Santos^6^, Joana Silva-Dinis^7^, João Lagoas^8^, João Nascimento^9^, João Oliveira^10^, Maria J. Simões^10^, Marta Cabral^11^, Pedro Abreu^12^, Pedro M. Teixeira^13^, Rodrigo Rei^14^, Susana Almeida^15^, Vanessa Fraga^16^, Ana F. Mourão^17^, Liliana Saraiva^18^, Patrícia C. Reis^19^, Raquel Campanilho-Marques^1^, Filipa Oliveira-Ramos^1^

###### ^1^Rheumatology, Centro Hospitalar Lisboa Norte, Lisbon; ^2^Rheumatology, Unidade Local de Saúde de Gaia/Espinho, Gaia; ^3^Rheumatology, Unidade Local de Saúde de Loures-Odivelas, Loures; ^4^Peadiatric, Hospital de Vila Franca de Xira, Vila Franca de Xira; ^5^Rheumatology, Unidade Local de Saúde de Trás-os-Montes e Alto Douro, Vila Real; ^6^Rheumatology, Unidade Local de Saúde de São João, Porto; ^7^Rheumatology, Unidade Local de Saúde de São José, Lisbon; ^8^Rheumatology, Unidade Local de Saúde do Tâmega e Sousa, Penafiel; ^9^Peadiatric; ^10^Rheumatology, Unidade Local de Saúde de Coimbra, Coimbra; ^11^Peadiatric, Hospital Professor Doutor Fernando Fonseca, Amadora; ^12^Rheumatology, Unidade Local de Saúde de Castelo Branco, Castelo Branco; ^13^Rheumatology, Unidade Local de Saúde da Região de Aveiro, Aveiro; ^14^Rheumatology, Hospital de Faro, Faro; ^15^Rheumatology, Unidade Local de Saúde do Alto Minho, Ponte de Lima; ^16^Rheumatology, Unidade Local de Saúde de Almada Seixal, Almada; ^17^Rheumatology, Centro Hospitalar Lisboa Ocidental, Lisbon; ^18^Rheumatology, Unidade Local de Saúde de Viseu Dão-Lafões, Viseu; ^19^Peadiatric, Centro Hospitalar Lisboa Norte, Lisbon, Portugal

####### **Correspondence:** Augusto Silva

*Pediatric Rheumatology 2024*, **22(2)**: PReS24-ABS-1593

**Introduction:** Still’s disease (SD) is understood as a life course entity encompassing systemic-onset juvenile idiopathic arthritis (SoJIA) and adult-onset Still’s disease (AOSD). SD-associated lung disease (LD) is an emerging severe complication that is apparently increasing in frequency.

**Objectives:** To describe and compare SoJIA and AOSD in a multicentric population emphasizing pulmonary involvement.

**Methods:** Clinical records of patients classified as SoJIA or AOSD, from 18 Portuguese centers, were reviewed. Patients with clinical or chest imaging objective findings were considered to have LD, including interstitial lung disease, pulmonary alveolar proteinosis and pulmonary arterial hypertension. Previous/concomitant known LD related to other causes was excluded. Data on demographic variables and clinical features were presented as frequencies and mean ± standard deviation for categorical and continuous variables, respectively. Linear regression was performed to assess the independent association of relevant covariables.

**Results:** We collected data from 175 patients, 104 with SoJIA, 67 with AOSD, 4 unknown age of symptoms onset. The mean age of symptoms onset was 8.2 years for SoJIA and 38.5 years for AOSD patients. LD developed in 14 patients (8%), 7 with SoJIA (6.7% of the total), and 7 with AOSD.

At SD onset 99% of the patients had fever, 89.1% arthritis, 81.3% rash, 43.5% odynophagia, 43% myalgia, 40.6% adenomegaly, 37.1% hepatomegaly/splenomegaly with no significant differences between SoJIA and AOSD except for lower reports of odynophagia and adenomegaly in SoJIA. Similar results were obtained in the subgroup of paediatric and adult patients that developed LD.

LD presented with tiredness in 57.1%, dyspnea in 42.9% and clubbed fingers in 15.4% of the cases. Low peripheral oxygen saturation was more frequent in AOSD than SoJIA (57.1% vs 14.3%).

The average time between SD symptoms onset and diagnosis of LD was 6.1 years for SoJIA and 7.3 years for AOSD, with a mean age at LD diagnosis of 9.0 and 39.2 years for SoJIA and AOSD, respectively. At LD diagnosis, all patients with AOSD and 57.1% with SoJIA had active SD. The most common radiological findings were ground glass (42.9%), peripheral consolidation (35.7%) and septal thickening (21.4%), while pulmonary hypertension was found in 21.4% of the patients, with similar frequencies in both subgroups.

Patients with LD had more pleuritis (42.9% vs 10.9%; p=0.024) and a higher frequency of macrophage activation syndrome (MAS) (35.7% vs 9.4%; p<0.001) independent of age of SD-onset. Half of the patients, all adults, had eosinophilia at LD diagnosis. After LD diagnosis, patients were treated with glucocorticoids (69.2%), tocilizumab (21.4%) and anakinra (7.1%), in similar proportions in both subgroups. After a mean of 1.8 years of follow-up after LD diagnosis, 6 patients (42.9%) needed intensive care (3 with SoJIA), 4 (28.6%) died (1 with SoJIA) and 9 (64.3%) had improvement or stabilization of the LD (5 with SoJIA and 4 with AOSD).

**Conclusion:** LD is a significant and severe complication in patients with SD, affecting both paediatric and adult populations. Despite similar symptoms at onset in SoJIA and AOSD patients, those with LD may face a more severe prognosis, including higher rates of pleuritis and MAS.

**Date of birth::** mars 14, Y

**Patient Consent**

Not applicable (there are no patient data)

**Disclosure of Interest**

None Declared

### Systemic JIA

#### P374 Predictors of remission in systemic juvenile idiopathic arthritis / still’s disease in the biologic era - a real-life experience consistent with the “window of opportunity”

##### Sarah Abu Rumeileh^1^, Maria Vincenza Mastrolia^2, 3^, Audrey Laurent^4^, Marine Fouillet-Desjonqueres^4^, Franck Zekre^4^, Ilaria Pagnini^2^, Edoardo Marrani^2^, Ilaria Maccora^2, 3^, Alexandre Belot^4^, Gabriele Simonini^2, 3^

###### ^1^Pediatric Department, Rheumatology Unit, AOU Pisana, Santa Chiara Hospital, Pisa; ^2^Rheumatology Unit, Meyer Children Hospital IRCCS; ^3^NEUROFARBA Department, University of Florence, Florence, Italy; ^4^Nephrology and Rheumatology Unit, Hospices Civils de Lyon, Hôpital Femme-Mère-Enfant, Lyon, France

####### **Correspondence:** Sarah Abu Rumeileh

*Pediatric Rheumatology 2024*, **22(2)**: PReS24-ABS-1637

**Introduction:** Among the various subtypes of juvenile idiopathic arthritis (JIA), systemic JIA (sJIA) / Still’s disease, stands out as a distinct clinical entity because of its peculiar pathogenesis attributable to the spectrum of autoinflammatory disorders. The evidence about the role of epidemiological and clinical factors as predictors of different disease trajectories in sJIA patients remains limited, especially because most studies dated back to the pre-biological era [1].

**Objectives:** To investigate potential predictors of remission and relapse in sJIA, in a real-life clinical setting.

**Methods:** An observational bicentric cohort study was conducted including patients diagnosed with sJIA between January 2017 and December 2022 at Meyer Children’s Hospital IRCCS (Florence, Italy), or at Hospices Civils de Lyon (France). Data on demographics, clinical and laboratory features, treatment approaches, and disease evolution were collected through retrospective chart reviews.

**Results:** Of the 64 sJIA patients included, 57.8% exhibited a monophasic course, and 42.2% had a non-monophasic course. 46.9% of patients (30/64) were females. The median age at diagnosis was 6.5 years (IQR 3-12), with a median time from symptom onset to the diagnosis of 23 days (IQR 14-32.5). Patients were followed up for a median of 22 months (IQR 12-38.8) and 60/64 (93.7%) achieved remission on medication and 35/64 (54.7%) remission off medication. The time from first symptom to diagnosis (Hazard Ratio (HR): 0.991) and interleukin 1 (IL1) inhibitors treatment failure (HR: 0.236) resulted predictors of a longer time to achieve remission on therapy. Clinical inactive disease at month 3 (HR: 3.506) predicted a shorter interval of time to remission off medication while anti-IL1 failure (HR: 0.153) was found to be a predictor of longer time to achieve remission off medication. The presence of rash three months after onset (HR: 5.763) resulted significantly associated with a shorter time to relapse, while the male gender resulted a protective factor (HR: 0.247). IL1 inhibitors non-responder patients (15/42, 35.7%) presented a lower age (p=0.040) and a higher frequency of polyarthritis at onset (p=0.029), a non-monophasic disease course (p<0.001), a higher number of relapses (p=0.010), and a longer time to achieve remission on therapy (p<0.001). Non-responder patients reported a longer time interval in starting anti-IL1 treatment (median 56 days vs 27 days), although this result did not reach a statistically significant (p=0.098) value.

**Conclusion:** A diagnostic and therapeutic delay predicts a longer time to reach remission in sJIA patients, and seems to affect the response to IL1 inhibition, according to the “window of opportunity” hypothesis in sJIA treatment. A failure to IL1 inhibitors predicts a longer time to reach remission both on and off medications and is associated with an early polyarticular onset and non-monophasic disease course.

**Patient Consent**

Yes, I received consent

**Disclosure of Interest**

None Declared

**Reference**

1. Singh-Grewal D, Schneider R, Bayer N, *et al.* Predictors of disease course and remission in systemic juvenile idiopathic arthritis: significance of early clinical and laboratory features. *Arthritis Rheum*. 2006;54:1595–601.

### Systemic JIA

#### P375 Identifying potential disease course and response predicting biomarkers in systemic juvenile idiopathic arthritis using a mixed-effects modeling approach

##### Remco Erkens ^1, 2^, Arjen Leek^1, 2^, Julia Drylewicz^2^, Dörte Hamann^2^, Gerda Den Engelsman^1^, Sytze De Roock^1, 2^, Dieneke Schonenberg-Meinema^3^, Merlijn Van den Berg^3^, Mariken Gruppen^3^, Wineke Armbrust^4^, Elizabeth Legger^4^, Sylvia Kamphuis^5^, Marleen Verkaaik^5^, Ellen Schatorjé^6^, Esther Hoppenreijs^6^, Thomas Vogl^7^, Johannes Roth^7^, Joost Swart^1^, Marc Jansen^1^, Jorg Van Loosdregt^2^, Bas Vastert^1, 2^

###### ^1^Division of Pediatric Rheumatology and Immunology, Wilhelmina Children’s Hospital; ^2^Center for Translational Immunology, University Medical Center Utrecht, Utrecht; ^3^Division of Pediatric Rheumatology and Immunology, Amsterdam University Medical Center, Amsterdam; ^4^Division of Pediatric Rheumatology and Immunology, University Medical Center Groningen, Groningen; ^5^Division of Pediatric Rheumatology and Immunology, Erasmus Medical Center, Rotterdam; ^6^Division of Pediatric Rheumatology and Immunology, University Medical Center Radboud, Nijmegen, Netherlands; ^7^Institute of Immunology, University of Münster, Münster, Germany

####### **Correspondence:** Remco Erkens

*Pediatric Rheumatology 2024*, **22(2)**: PReS24-ABS-1687

**Introduction:** Systemic Juvenile Idiopathic Arthritis (sJIA) is a distinct but heterogeneous subtype of JIA, with many patients following a monophasic course, while others develop a refractory disease course[1]. The introduction of targeted biological therapy has revolutionized treatment and improved outcome. Unfortunately, still a significant number of sJIA patients show incomplete response to therapy and often need to try multiple and sequential biological treatments and/or still need adjunctive glucocorticoids to control their disease[2-3]. Treatment-refractory sJIA patients more often face severe and life-threatening complications including macrophage activation syndrome, sJIA associated lung disease, and destructive arthritis[1]. Currently no reliable biomarker is able to predict which patient will follow a monophasic disease course in sJIA and can safely taper and stop therapy and which patients will have a more refractory disease course. Non-linear mixed-effects models enable to simultaneously describe longitudinal repeated datapoints of biomarkers over time, allowing for inter- and intra-patient variation irrespective of missing data and aliening visits. This approach will help identify potential biomarker profiles that differentiate patient subgroups that can be later on used in a Cox Proportional Hazards Model with Time-Dependent Covariates to reliably predict outcome and disease course.

**Objectives:** Building a prediction algorithm using real life inflammatory biomarkers over time, that can aid in the clinical decision making of which patients can safely taper and stop therapy, and which sJIA patient is at risk for refractory disease.

**Methods:** We used clinical and laboratory data from the Dutch multicenter ‘Early Stop of targeted Treatment in children with Systemic JIA’ (ESTIS) trail with 68 new-onset therapy naïve sJIA patients starting with anakinra as first-line treatment. All Hb, CRP, ESR, ferritin, AST, ALT, triglycerides, D-dimer, thrombocytes, leucocytes, neutrophils, eosinophils, monocytes, CXCL10, CXCL13, Galectin-9, IL-6, IL-18, IL-10 and S100A8/9 datapoints were extracted and modeled over time. Clinical disease parameters and biomarker measurements over time were modeled using a mixed effect models approach, including fixed and random intercept and slope, as well as adjusting for the outcome and disease course for the intercept and slope when it significantly improved the model.

**Results:** Based solely on the cytokine levels before start of treatment we are unable to predict which patient will have a monophasic disease course in the first year of the disease course. The trajectory of IL-18 over time however was significantly different in monophasic patients vs non-monophasic patients. IL-18 over time, are able to *early* (< 2 months of disease) differentiate between sJIA patients that will achieve disease remission without medication and sJIA with a refractory disease course at T=12 months after the start of treatment. We are currently implementing additional clinical parameters and biomarkers to further improve our model resulting in increased sensitivity and selectivity.

**Conclusion:** Modeling inflammatory biomarkers over time with non-linear mixed effects is a promising tool that can aid in identifying biomarkers discriminating patient groups with either a monophasic or refractory disease course. Predictive algorithms could directly translate into clinical practice: early taper and stop of treatment in monophasic sJIA patients, while adjusting therapy early in the disease course in patients with a high risk for refractory disease.

**Patient Consent**

Yes, I received consent

**Disclosure of Interest**

R. Erkens: None Declared, A. Leek: None Declared, J. Drylewicz: None Declared, D. Hamann: None Declared, G. Den Engelsman: None Declared, S. De Roock: None Declared, D. Schonenberg-Meinema: None Declared, M. Van den Berg: None Declared, M. Gruppen: None Declared, W. Armbrust: None Declared, E. Legger: None Declared, S. Kamphuis: None Declared, M. Verkaaik: None Declared, E. Schatorjé: None Declared, E. Hoppenreijs: None Declared, T. Vogl: None Declared, J. Roth: None Declared, J. Swart: None Declared, M. Jansen: None Declared, J. Van Loosdregt: None Declared, B. Vastert Grant / Research Support with: SOBI, Consultant with: SOBI and Novartis

**References**

1. PMID: 34635293
2. PMID: 30848528
3. PMID: 32402087

### Systemic JIA

#### P376 Interstitial lung disease in systemic juvenile idiopathic arthritis: a nationwide study

##### Dilara Ünal ^1^, Elif K. Könte^2^, Elif Çelikel^3^, Hatice K. Zora^4^, Metin K. Gürgöze^5^, Veysel Çam^1^, Yağmur Bayındır^1^, Hülya E. Emreol^1^, Özge Başaran^1^, Kenan Barut^2^, Yelda Bilginer^1^, Özgür Kasapçopur^2^, Seza Özen^1^

###### ^1^Department of Pediatric Rheumatology, Hacettepe University, Ankara; ^2^Department of Pediatric Rheumatology, İstanbul University, Cerrahpaşa Faculty of Medicine, İstanbul; ^3^Department of Pediatric Rheumatology, Ankara Cıty Hospital, Ankara; ^4^Department of Pediatric Rheumatology, Uludağ University, Bursa; ^5^Department of Pediatric Rheumatology, Fırat University, Elazığ, Türkiye

####### **Correspondence:** Dilara Ünal

*Pediatric Rheumatology 2024*, **22(2)**: PReS24-ABS-1727

**Introduction:** Interstitial lung disease (ILD) is becoming a major concern in systemic juvenile arthritis (sJIA). Several previous studies showed the severe course and fatal outcomes in these patients.

**Objectives:** To describe the features of sJIA patients with ILD from Turkey.

**Methods:** In the present retrospective cohort study, 18 patients younger than 18-years-old with sJIA and ILD were included. The diagnosis of sJIA was made according to the 2004 ILAR criteria. ILD was diagnosed with chest computed tomography with the exclusion of other possible reasons. Macrophage activation syndrome (MAS) was diagnosed with HLH-2004 and 2016 EULAR/ACR/PRINTO Classification Criteria.

**Results:** The onset age of sJIA ranged from 2 years to 16 years. Eight of the patients were female. The time interval between the diagnosis of sJIA and the development of ILD ranged from 2 months to 8 years. Ninety four percent (17/18) of the patients had a history of MAS, twelve of them had a MAS episode concomitantly with ILD diagnosis. Cough was the most common symptom in 94% (17/18), followed by dyspnea (83%) and clubbing (83%). Thirteen patients (72%) had been treated with anakinra and six out of these 13 patients were discontinued because of having diagnosed with sJIA-ILD and were switched to other drugs such as JAK-inhibitors. Three of the patients stopped treatment because they were in remission. Three of the ILD patients were positive for HLA DRB1*15. Adverse reactions to anti-IL-6 agent-had been observed in two patients. Only one patient (5,5%) had hyper-eosinophilia. Eight had ground-glass opacity on radiographic examination. Two patients (11%) developed pulmonary arterial hypertension. Six (33,3%) patients had sJIA-ILD remission clinically and radiologically. Mortality was seen in only one patient.

**Conclusion:** ILD is a severe life-threatening complication of sJIA that may affect children of different ages with different time intervals. Further studies are needed to define predictors, and treatment options, for preventing and treating ILD in sJIA.

**Date of birth::** janvier 19

**Patient Consent**

Yes, I received consent

**Disclosure of Interest**

None Declared

### Systemic JIA

#### P377 The safety and efficacy of simultaneous vaccination with pcv13 and HIB in children with systemic Juvenile idiopathic arthritis

##### Olga Lomakina^1^, Ekaterina Alexeeva^1, 2^, Tatyana Dvoryakovskaya^1, 2^, Anna Fetisova^1^, Elizaveta Krekhova^1^, Meyri Shingarova^1, 2^, Ivan Kriulin^1^, Tatyana Kriulina^1, 2^, Irina Tsulukiya^1^, Natalia Lisitsina^3^, Anastasia Zhuzhula^4^, Maria Kokina^1^, Mikhail Kostik^5^

###### ^1^Rheumatology, National Medical Research Center for Children's Health; ^2^Pediatric, I.M. Sechenov First Moscow State Medical University (Sechenov University); ^3^Vaccination; ^4^Laboratory, National Medical Research Center for Children's Health, Moscow; ^5^Pediatric, Saint Petersburg State Pediatric Medical University, Saint-Petersburg, Russian Federation

####### **Correspondence:** Olga Lomakina

*Pediatric Rheumatology 2024*, **22(2)**: PReS24-ABS-1275

**Introduction:** Systemic juvenile idiopathic arthritis (SJIA) is the most severe form of JIA, with a rapidly progressive course and poor prognosis. The risk of infections, such as pneumococcal and hemophilus influenzae, can lead to skipping immunosuppressive therapy and exacerbation of the disease. Vaccination is the main tool to prevent these infections.

**Objectives:** To evaluate safety and efficacy of simultaneous vaccination with PCV13 and Hib in children with systemic juvenile idiopathic arthritis.

**Methods:** The study included 100 patients: 55 girls and 45 boys with sJIA aged 2 to 17.5 years. Patients were not vaccinated PCV13 and Hib before. Pneumococcal conjugate (PCV13) and Hib vaccines were administered (0.5 ml each) concurrently subcutaneously into the deltoid area. Anti-SP IgG, anti Hib, hsCRP and S100 protein were evaluated before and 3 weeks and 6 months after the vaccination by the ELISA. Seroprotection was established for anti-pneumococcal IgG ≥ 7 U/ml and for anti-Hib IgG ≥ 1.07 ug/ml. We assessed the dynamics of clinical parameters (systemic manifestations, joints with active arthritis) 3 weeks, 6 months after vaccination (compared to baseline). The upper limit of the reference interval for hsCRP was considered (according to the manufacturer's instructions) a value of 8.2 mg/L, for S100 – 2.9 μg/ml. hsCRP and S100 were considered as predictors of disease activity. Evaluating of vaccine safety was recorded during 3 weeks after administration. Analysis carried out rate of infectious diseases, duration of infectious disease (week), rate of courses of antibacterial therapy 6 months before and 6 months after the vaccination.

**Results:** Patients received biologic treatment (91%): tocilizumab-56%, canakiumab-30%, etanercept-2%, adalimumab-3% and 34% of the patients received methotrexate as monotherapy - 9.0% and in the combination with biologics – 44%. The average age of the patients was 10.3 (2.0, 17.5) years. The significant raise in the anti-SP IgG, antibodies Hib levels in three weeks after vaccination was detected. By the 6 month after vaccination the abovementioned indicators remained at the same level without clinical and laboratorial (S100 and hsCRP) flare. Before vaccination anti-SP IgG, Me (SD) U/ml 95% - 61 (58) [50, 73], protective titer anti-SP IgG, n (%) - 90 (90%) [82%, 95%], antibodies Hib, Me (SD) U/ml 95% CI - 1.49 (1.57), protective titer to anti-HIb, n (%) 95% CI - 40 (40%) 30%, 50%, S-100, Me (SD) U/ml 95% CI - 7 (8) 4.9, 8.1, hsCRP, Me (SD) U/ml 95% CI 2.2 (5.4) 1.1, 3.3, systemic manifestations, n (%) 95% CI 12 (12%)6.6%, 20%, joints with active arthritis, Me (SD) 95% CI - 0.54 (2.11) 0.12, 0.96; 3 weeks after vaccination: 124 (88) [107, 142], 95 (95%)[88%, 98%], 3.95 (3.39) 3.3, 4.6 95 (95%)88%, 98%, 5.2 (5.8) [3.2, 5.3], 2.3 (4.3) 1.4, 3.1, 4 (4.0%)1.3%, 11%, 0.40 (1.75) 0.06, 0.75, 6 months after - 116 (76)[ 100, 131], 99 (99%)[94%, 100%], 3.90 (3.28) 3.3, 4.6, 92 (92%) 84%, 96%, 1.59 (2.99) 1.0, 2.2, 1.58 (2.90) [1.3, 1.9], 2 (2.0%)0.35%, 7.7%,0.150 (0.869) -0.02, 0.32, respectively. The level of antibodies significantly increased 3 weeks and 6 months after vaccination. The clinical activity of the disease and the concentration of S-100 and hsCRP did not significantly increase.

The main AEs were systemic (4%), hyperthermia at the injection site (8%), and pain at the injection site (13%). The rate of infectious diseases 6 months before vaccination - 4.0 (3.8; 4.3), duration of infectious disease (week) 9.6 (9.0; 10), number of courses of antibacterial therapy 2.7 (2.5; 2.9) significantly decreased 6 months after vaccination and was 1.8 (1.6; 2.0); 2.8 (2.4; 3.1) and 0.8 (0.7, 1.0), respectively.

**Conclusion:** Simultaneous vaccination PCV13 and Hib of children with sJIA was safe and effective and could be recommended children with sJIA.

**Patient Consent**

Not applicable (there are no patient data)

**Disclosure of Interest**

None Declared

### Systemic JIA

#### P378 Worldwide evaluation of clinical practice strategies (clips) for lung involvement in still's disease within the Jir-clips network: a cost action

##### Maurine Jouret^1^, Francisca Aguiar^2^, Charlotte Girard^3^, Yulia Vyzhga^4, 5^, Filipa Oliveira-Ramos^6^, Cristina Costa Lana^7^, Rahma Guedri^8, 9^, Alain Lefèvre-Utile^10, 11^, Djohra Hadef^12^, Juan Manuel Mosquera Angarita ^13^, Ozen Seza ^14^, Sezgin Sahin^15^, Soad S. Hashad^16^, Dirk Foell^17^, Karima Daghor- Abbaci^18^, Sophie Georgin-Lavialle^19^, Katerina Theodoropoulou ^20, 21^ and JIR-CLIPS Working Group

###### ^1^Hôpital Femme-Mère-Enfants, Pediatric Nephrology, Rheumatology and Dermatology Unit, Hospices Civils de Lyon, Lyon, France; ^2^Adult and Pediatric Rheumatology Unit, ULS São JoãoYoung, Porto, Portugal; ^3^Department of Medicine, University of Geneva, Geneva, Switzerland; ^4^National irogov Memorial Medical University, Vinnytsya, Ukraine; ^5^The Hospital for sick children “SickKids”, Toronto, Canada; ^6^Pediatric Rheumatology Unit, Hospital Universitário Santa Maria, Faculdade de Medicina da Universidade de Lisboa, Lisbonne, Portugal; ^7^Medical School of Medicine, Federal University of Minas Gerais State (UFMG), Hospital das Clínicas, Belo Horizonte, Brazil; ^8^Pediatric rheumatology unit, Béchir Hamza Children’s Hospital; ^9^Faculty of Medicine of Tunis & University of Tunis El Manar, Tunis, Tunisia; ^10^Département femme-mère-enfant, Centre Hospitalier Universitaire Vaudois; ^11^Faculté de biologie et de médecine de l’Université de Lausanne (UNIL), Lausanne, Switzerland; ^12^Faculty of Medicine, Batna 2 University, Batna, Algeria; ^13^Pediatric Rheumatology Unit, Sant Joan de Déu Children's Hospital , Barcelona, Spain; ^14^Department of Pediatrics, Hacettepe University, Ankara; ^15^Department of Pediatric Rheumatology, University Cerrahpasa, Istanbul, Türkiye; ^16^University of Tripoli , Tripoli children hospital, Tripoli, Libya; ^17^Department of Pediatrics, University of Münster, Munster, Germany; ^18^CHU Babeloued, Algiers, Algeria; ^19^, Internal Medicine Department, Tenon Hospital, APHP, Paris, France; ^20^Department of Woman, Mother, Child, Unit of Pediatric Immunology, Allergology and Rheumatology, Lausanne University Hospital; ^21^Lausanne University Hospital and University of Lausanne, Lausanne, Switzerland

####### **Correspondence:** Maurine Jouret

*Pediatric Rheumatology 2024*, **22(2)**: PReS24-ABS-1452

**Introduction:** Recent literature has documented a subset of patients with severe Still’s Disease who are prone to develop lung disease (LD). This condition is described in both systemic juvenile idiopathic arthritis (sJIA) and adult-onset Still's disease (AOSD). It may manifest as interstitial lung disease, pulmonary arterial hypertension, and/or alveolar proteinosis. The management of this complication remains challenging for physicians as it is often refractory to conventional treatment.

**Objectives:** This study aims to outline global clinical practices for Still’s associated lung disease through the Clinical Practice Strategies (CliPS) network and compare them with existing recommendations.

**Methods:** This study is part of the CLiPS project, a work dedicated to capture clinical practice strategies worldwide by disseminating questionnaires to physicians about five diseases, including Still’s disease. The project, funded as a COST (European Cooperation for Science and Technology) action, has been distributing the questionnaires since September 2022 through the JIRcohort network and national societies.

**Results:** On April 14, 2024, 337 physicians responded to the Still’s disease questionnaire. Fifty-eight physicians (17%) from 5 continents reported following patients with lung involvement. Most respondents were pediatricians (86%) but adults rheumatologists (n=8/58) also reported encountering this condition. Acute digital clubbing, history of recurrent MAS and adverse events to cytokine-targeted biotherapies were identified as key clinical features to evoke LD. Radiography and thoracic computed tomography were performed by the majority of physicians (92% and 78% respectively). Bronchoalveolar lavage was carried out systematically by 40% of respondents. Cardiac echocardiography was performed either always (78%, 39 out of 50 respondents) or sometimes (22% of the respondents) to rule out pulmonary hypertension. Pulmonary biopsy was not deemed necessary to confirm lung disease. In fact, 71% of physicians performed biopsies occasionally, while the remainder never found the need for this invasive procedure. HLADRB1*15 was used by 65% of physicians to support LD diagnosis. Fifty percent of participants could use interferon signature to aid in the management of these patients while IL-18 cytokine assay is performed by a few clinicians (8/24 participants, 33%). Thirty-seven percent of the respondents chose to continue the same treatment with additional therapeutics, while 31% decided based on the effectiveness of biological treatment to control systemic activity, and 17% chose to stop biologics. JAK inhibitors were the medication most frequently chosen as additional treatment (n=25), followed by cyclosporine (n=18) and mycophenolate mofetil (n=14).

**Conclusion:** This study highlights variations in medical approaches regarding screening, follow-up and treatments for Still's Disease-LD. There is a need to define real-life clinical practice strategies that are tailored to the specific contexts of different regions worldwide to enhance the management of these patients.

**Patient Consent**

Not applicable (there are no patient data)

**Disclosure of Interest**

None Declared

### Systemic JIA

#### P379 Phenotypic and functional characterization of innate lymphoid cells in systemic Juvenile idiopathic arthritis patients

##### Linda Quatrini^1^, Cecilia Ciancaglini^2^, Ivan Caiello^3^, Silvia Santopolo^1^, Valentina Matteo^3^, Elena Loricchio^3^, Manuela Pardeo^3^, Claudia Bracaglia^3^, Nicola Tumino^1^, Paola Vacca^1^, Lorenzo Moretta^2^, Fabrizio De Benedetti^3^, Giusi Prencipe^3^

###### ^1^Innate Lymphoid cell Unit; ^2^Tumor Immunology Unit; ^3^Rheumatology Unit, Bambino Gesù Children's Hospital, Rome, Italy

####### **Correspondence:** Giusi Prencipe

*Pediatric Rheumatology 2024*, **22(2)**: PReS24-ABS-1487

**Introduction:** Systemic Juvenile Idiopathic Arthritis (sJIA) is an autoinflammatory disease characterized by fever, rash, lymphadenopathy, hepatosplenomegaly and serositis. Macrophage activation syndrome (MAS) is a potentially life-threatening complication of sJIA. Innate immune mechanisms and overproduction of inflammatory cytokines, including interleukin-1 (IL-1), IL-6, and IL-18, play a central role in the pathogenesis of sJIA. Likewise, the expansion and prolonged activation of macrophages and CD8+ T cells, along with excessive interferon-gamma (IFN-γ) production, are the main drivers of MAS manifestations.

**Objectives:** Innate Lymphoid Cells (ILCs), comprising Natural Killer (NK) cells and helper-ILCs (hILCs), represent the innate cellular source of IFN-γ. Peripheral blood hILCs encompass ILC1s, ILC2s, and ILCPs, with ILC1s producing IFN-γ and ILC2s producing IL-13. This study aimed to understand the potential role of these cells in sJIA pathogenesis/progression through phenotypic and functional characterization of ILCs in sJIA patients.

**Methods:** Peripheral ILCs from children with inactive sJIA under IL-1 inhibitors treatment (n=34) were analyzed by flow cytometry and compared to those of 23 healthy children.

**Results:** In inactive sJIA patients, circulating NK cells were significantly reduced, with a higher proportion of CD56bright cells, compared to healthy controls. The frequency and number of hILCs was comparable between the two groups, with hILCs correlating with therapy duration. In sJIA patients, the composition of hILC subsets showed increased ILC1 frequency and decreased ILC2 and ILCP frequencies compared to controls. While the frequency of ILC1 positively correlated (p<0.05, r=0.4), the frequency of ILC2 negatively correlated (p=0.01, r=0.45) with IL-18 plasma levels, indicating a shift in hILC subset proportions associated with a disease activity marker related to the FN-γ pathway. To assess the functional capacity of NK cells and hILCs, peripheral blood mononuclear cells were stimulated with PMA/ionomycin or with IL-18/IL-12, and intracellular IFN-γ levels were measured by flow cytometry. While NK cells from sJIA patients exhibited comparable IFN-γ production to controls under various stimulations, hILCs displayed lower IFN-γ production, indicative of intrinsic functional impairment. Notably, IL-13 levels remained unaffected, suggesting a specific defect in IFN-γ production by hILCs in inactive sJIA patients.

**Conclusion:** Inactive sJIA patients display a lower frequency of NK cells, and an increased frequency of ILC1s as compared to healthy donors. Despite this alteration, hILCs have an intrinsic defect in IFN-γ production. Altogether, these findings suggest an alteration in group 1 ILC subset composition and function in sJIA patients, despite the absence of clinical disease activity

**Patient Consent**

Yes, I received consent

**Disclosure of Interest**

None Declared

### Systemic JIA

#### P380 The combination of clinical manifestations in the onset of disease in patients with systemic juvenile idiopathic arthritis receiving biological agents in the russian federation: federal register data

##### Maria Botova^1^, Ekaterina Alexeeva^1, 2^, Tatiana Dvoryakovskaya^1, 2^, Tatiana Kriulina^1, 2^, Aleksandra Chomakhidze^1^, Olga Lomakina^1^, Anna Fetisova^1^, Ivan Kriulin^1^, Elizaveta Krekhova^1, 2^, Irina Tsulukiya^1^, Natalia Kondrateva^1^, Meiri Shingarova^1, 2^, Maria Kokina^1, 2^

###### ^1^Rheumatology, National Medical Research Center for Children’s Health; ^2^Sechenov First Moscow State Medical University (Sechenov University), Moscow, Russian Federation

####### **Correspondence:** Ekaterina Alexeeva

*Pediatric Rheumatology 2024*, **22(2)**: PReS24-ABS-1408

**Introduction:** Systemic juvenile idiopathic arthritis (sJIA) is the rarest variant of juvenile idiopathic arthritis, characterized by severe course, frequent exacerbations, the development of life-threatening extra-articular manifestations and complications, which requires the use of expensive medications and frequent hospitalizations of patients. sJIA has to be diagnosed at an early stage. One of the problems of early diagnostics is absence of arthritis at disease onset in some patients. Arthritis may be a non-prominent and/or a delayed manifestation. Since morbidity and mortality may develop early in sJIA patients, a strict adherence to the ILAR criteria may lead to an unacceptable delay in diagnosis. In the Russian Federation (RF), the provision of medicines to patients with sJIA is carried out at the expense of the federal budget, in this regard: the Federal Register (FR) of sJIA was created in 2018.

**Objectives:** To analyze clinical manifestations at the onset of sJIA in RF according to the data from the FR.

**Methods:** Retrospective analysis of medical data of patients receiving biologic agents with sJIA included in the FR of the RF. The analysis included patients receiving biologics: tocilizumab, canakinumab, etanercept, adalimumab.

**Results:** The total number of patients in the FR is 2002, among them – 927 receiving biologics. Almost all children had febrile fever 908 (98%); 748 (80,7%) – rash; 562 (60,6%) – hepatomegaly and/or splenomegaly; 383 (41,3 %) - lymphadenopathy, 198 (21,4 %) – serositis. Active arthritis at the onset of the disease developed in 708 (76,4%) children. Also, we analyzed the combinations of clinical symptoms at the onset of sJIA. Most often observed fever, rash, arthritis –140/927 (15,1%); fever, rash, arthritis, hepatomegaly, splenomegaly, lymphadenopathy – in 92/927 (9,9%); fever, rash, arthritis, hepatomegaly and/or splenomegaly – 91/927 (9,8%); fever and rash – in 58/927 (6,3%); fever and arthritis – in 44/927 (4,7%); fever, rash, arthritis, hepatomegaly, splenomegaly, lymphadenopathy, pericarditis - in 42/927 (4,5%); fever, rash, arthritis, hepatomegaly and splenomegaly, lymphadenopathy, pericarditis, pleurisy, ascites – in 11/927 (1,2%) and other combinations – in 449/927 (48,5%).

**Conclusion:** We are seeing cases where patients clearly suffer sJIA, but there is no arthritis. We should be aware that even in the absence of arthritis, highly suggestive clinical manifestations of sJIA shouldn’t rule out its diagnosis.

**Patient Consent**

Yes, I received consent

**Disclosure of Interest**

None Declared

### Systemic JIA

#### P381 Interleukin-18 as a promising cytokine for differentiation between systemic Juvenile idiopathic arthritis and other prolonged fever diseases

##### Saroch Pattaratiansakul, Butsabong Lerkvaleekul, Soamarat Vilaiyuk

###### Pediatric Department, Rheumatology Division, Faculty of Medicine Ramathibodi Hospital, Mahidol University, Bangkok, Thailand

####### **Correspondence:** Soamarat Vilaiyuk

*Pediatric Rheumatology 2024*, **22(2)**: PReS24-ABS-1166

**Introduction:** Prolonged fever in children causes by several differential diagnoses, including infections, autoimmune conditions, and malignancies. The approach to these patients relies on clinical features and laboratory investigations. Previous studies tried to identify biomarkers that distinguish the cause of prolonged fever but it is still uncertain. Systemic juvenile idiopathic arthritis (SJIA) typically manifests with prolonged and high-grade fever, necessitating the exclusion of other potential causes such as infection, malignancy, or other autoimmune diseases like Kawasaki disease (KD) before confirming the diagnosis of SJIA. Interleukin (IL)-18, a proinflammatory cytokine primarily produced by macrophages and dendritic cells, plays significant roles in autoimmune diseases, particularly SJIA, and macrophage activation syndrome. Previous literature revealed that IL-18 is also involved in malignancies and infectious diseases. However, its utility in differentiating the causes of prolonged fever in children remains unclear.

**Objectives:** This study aims to determine whether IL-18 levels can distinguish between SJIA and other causes of prolonged fever, including infection, malignancy, and KD.

**Methods:** This is a cross-sectional study design. Patients with prolonged fever (temperature > 38°C) persisting for more than seven days were recruited. Baseline characteristics, comprising clinical and laboratory data, along with diagnosis, were collected. Patients were categorized into two groups: 1) SJIA based on International League of Associations for Rheumatology criteria and 2) non-SJIA (comprising other causes of prolonged fever). Blood samples were collected upon the patients' initial hospital presentation, and serum was extracted and stored at -80°C until analysis. IL-18 levels were quantified using the enzyme-linked immunosorbent assay (ELISA) technique. Group comparisons were conducted using the Chi-square and Mann-Whitney U tests. The receiver operating characteristic (ROC) analysis for IL-18 levels was generated to determine the optimal cut-off value for differentiating SJIA from other causes of prolonged fever.

**Results:** Among 62 patients, 22 were diagnosed with SJIA, while 40 had other diseases (infection 25%, malignancy 27.5%, KD 30%, and others 17.5%). The median ages (interquartile range [IQR]) were 8.4 (5.8-11.6) years for SJIA patients and 4.0 (1.3-7.8) years for those with other prolonged fever diseases. The median [IQR] levels of inflammatory markers, including erythrocyte sedimentation rate (68.0 [30-94] mm/h vs. 69 [22.5-93.5] mm/h, P-value = 0.90), C-reactive protein (72.1 [41.4-170.2] mg/L vs. 71.0 [29.3-131.8] mg/L, P-value = 1.0), and ferritin (1,121.5 [314.1-21,430.2] ng/mL vs. 404.8 [156.7-1,175.6] ng/mL, P-value = 0.37), did not show statistical differences between the two groups. However, the median [IQR] levels of serum IL-18 were significantly higher in SJIA patients (7,672.3 [1,175.4-52,085.0] pg/mL) compared to patients with other prolonged fever diseases (437.5 [225.7-1,169.2] pg/mL), with a P-value < 0.01. Furthermore, the ROC analysis for differentiating between SJIA and other prolonged fever diseases demonstrated a diagnostic sensitivity of 76.2% and specificity of 90.0% at IL-18 cut-off values ≥1,346.4 pg/mL.

**Conclusion:** IL-18 levels may serve as a biomarker aiding in the differentiation between SJIA and other causes of prolonged fever, whereas other basic inflammatory markers did not show the same ability.

**Patient Consent**

Not applicable (there are no patient data)

**Disclosure of Interest**

None Declared

### Systemic JIA

#### P382 The place of jak inhibitors in systemic juvenile idiopathic arthritis with lung disease (SJIA-LD): french experience

##### Gaëlle Côte^1^, Pierre Quartier^2, 3^, Alexandre Belot^4^, Isabelle Melki^5^, Véronique Hentgen^6^, Etienne Merlin^1^

###### ^1^Department of Pediatrics, CHU Estaing, Clermont Ferrand; ^2^Pediatric Immuno-Hematology and Rheumatology Unit, RAISE, Hopital Universitaire Necker-Enfants Malades; ^3^Université Paris-Cité, Paris; ^4^Pediatric Rheumatology, Nephrology and Dermatology Department, Hospices civils de Lyon, Lyon; ^5^General Pediatrics-Infectious Diseases and Internal Medicine Department, Hopital Robert Debré, Paris; ^6^Department of Pediatrics, Versailles Hospital, Le Chesnay, France

####### **Correspondence:** Gaëlle Côte

*Pediatric Rheumatology 2024*, **22(2)**: PReS24-ABS-1212

**Introduction:** A new form of systemic juvenile idiopathic arthritis (SJIA) with associated lung disease (SJIA-LD) has recently been described. Multiple lines of treatment have failed to yield satisfactory results for this disorder.

**Objectives:** JAK inhibitors (JAKi) have recently been approved for the treatment of JIA, but clinical evidence of their efficacy in SJIA-LD is still weak. Here we describe and assess real-life experience of SJIA-LD treatment with JAKi in France.

**Methods:** This is a retrospective study based on information gathered from patients’ medical records.

**Results:** Eight patients with SJIA-LD were identified in French pediatric rheumatology centers. All received at least one JAKi (baricitinib, ruxolitinib, tofacitinib). Complete disease control was obtained in four patients. Steroids were tapered in four patients and stopped in two. Three patients presented an episode of MAS shortly after the introduction of JAKi. Two patients had other serious side effects (viral reactivation (EBV, BK virus), cytopenia). At last follow-up, one patient had died from severe MAS, two patients had undergone hematopoietic stem cell transplantation, four were in complete response upon JAKi, and one in partial response with JAKis.

**Conclusion:** JAKi offer another therapeutic option for patients with SJIA-LD that may be worth starting early in the course of severe SJIA, both to prevent lung involvement and to reduce the need for prolonged high-dose steroids. However, the risk of triggering MAS and/or severe side effects must be considered.

**Patient Consent**

Not applicable (there are no patient data)

**Disclosure of Interest**

None Declared

### Systemic JIA

#### P383 Allogeneic hematopoietic stem cell transplantation for severe refractory systemic Juvenile idiopathic arthritis: a retrospective report of 3 patients

##### Muriel Schmutz^1^, Jade Cognard^2^, Véronique Hentgen^3^, Christine Pietrement ^4^, Paul Bastard^1^, Martin Castelle^1^, Marwa Chbihi^1^, Laurye-Anne Eveillard^1^, Romain Levy^1^, Anne Welfringer^5^, Laureline Berteloot^6^, Alice Hadchouel^7^, Pierre Quartier^1^, Benedicte Neven^1^, Despina Moshous^1^, Benjamin Fournier^1^, Marie Louise Frémond^1^

###### ^1^Department of Pediatric Immunology-Hematology and Rheumatology, Necker Hopital, Paris; ^2^Department of General Pediatrics, CHU Reims, Reims; ^3^Department of General Pediatrics, André Mignot Hopital; ^4^Department of General Pediatrics, CHU Reims; ^5^Department of Pediatric Dermatology; ^6^Department of Pediatric Radiology; ^7^Department of Pediatric Pulmonology, Necker Hopital, Paris, France

####### **Correspondence:** Muriel Schmutz

*Pediatric Rheumatology 2024*, **22(2)**: PReS24-ABS-1239

**Introduction:** Severe forms of systemic juvenile idiopathic arthritis (SJIA), also called pediatric-onset Still’s disease are associated with two major life-threatening complications: macrophage activation syndrome (MAS) and severe lung disease (LD) (1-3). These patients are usually resistant to conventional synthetic (cs) Disease-Modifying Antirheumatic Drugs (DMARDs), biologic (b) DMARDs, and Janus kinase inhibitors. These last years, allogeneic HSCT has been reported to achieve durable clinical remission in some patients with severe Still’s disease (4,5).

**Objectives:** We report 3 children with severe Still’s disease that underwent allogeneic HSCT.

**Methods:** We conducted a retrospective, observational, single-center study in a tertiary pediatric immunology care center (Necker Hospital, Paris, France).

**Results:** We report 3 patients with refractory SJIA complicated by recurrent MAS and interstitial lung disease. They all had elevated serum rate of IL-18 and had a disease refractory to cs DMARDs, bDMARDs and Janus kinase inhibitors. Two of them developed a drug-induced hypersensitivity -like syndrom after few months of IL-1 inhibitors and carried the HLA-DRB1*15 haplotype. The median age at conditioning regimen initiation was 3.5 years. Donors were matched sibling donor (MSD) for the first and the third patient, and matched unrelated donor (MUD) for the second patient. They developed post graft infections and graft-versus-host disease (acute and chronic). The second and third patients showed respectively severe post-transplant inflammatory complications with skin and lung involvement (diffuse alveolar hemorrhage), possibly related to their pre-transplant disease. Lastly the third patient presented a severe thrombotic microangiopathy. At a median follow-up of 14 months (from 8 to 22) after transplantation, 2 out of 3 patients were in complete remission with full donor chimerism and were off immunosuppressive treatment. The third patient is in partial remission suffering from polyarthritis despite corticosteroid and infliximab but improved her pre-graft pulmonary involvement.

**Conclusion:** Allogeneic HSCT is an effective salvage therapy in patients with refractory SJIA, however the risk of post-transplant severe immune-mediated inflammation on previously affected organs, such the skin and the lungs deserve peculiar attention.

**Patient Consent**

Yes, I received consent

**Disclosure of Interest**

None Declared

**References**

1. Minoia F, et al, Arthritis Rheumatol. 2014
2. Schulert GS, et al, Arthritis Rheumatol. 2019
3. Saper VE, et al, Ann Rheum Dis. 2019
4. Grom AA, et al, Pediatr Rheumatol Online J. 2024
5. M F Silva J, et al, Blood Adv. 2018

### Systemic JIA

#### P384 Unveiling major challenges and unmet needs in the therapeutic approach to systemic Juvenile idiopathic arthritis: the patient perspective

##### Francesco Baldo ^1^, Luciana Pereira^2^, Remco Erkens^3^, Claudia Bracaglia^4^, Dirk Foell^5^, Marco Gattorno^6^, Maria Jelusic^7, 8^, Sebastiaan J. Vastert^3^, Rashmi Sinha^2^, Francesca Minoia^9^ and PReS MAS/sJIA Working Party and the systemic JIA Foundation.

###### ^1^ASST-G.Pini-CTO, Milano, Italy; ^2^Systemic JIA Foundation., Cincinnati, United States; ^3^Wilhelmina Children's Hospital, utrecht, Netherlands; ^4^Ospedale pediatrico Bambin Gesù, Roma, Italy; ^5^Pädiatrische Rheumatologie und Immunologie Universitätsklinikum, Klinik für Kinder- und Jugendmedizin, Munster, Germany; ^6^IRCCS Istituto Giannina GasliniIRCCS Istituto Giannina Gaslini, Genova, Italy; ^7^ Department of Paediatric Rheumatology, Immunology and Alergology, University Hospital Centre (UHC) ; ^8^ University of Zagreb, School of Medicine, Zagreb, Croatia; ^9^IRCCS CA' Granda Ospedale Maggiore Policlinico, Milano, Italy

####### **Correspondence:** Francesco Baldo

*Pediatric Rheumatology 2024*, **22(2)**: PReS24-ABS-1530

**Introduction:** Despite continuous improvements in the therapeutic options for children with systemic juvenile idiopathic arthritis (sJIA), access to medications significantly differs among centres and countries. Furthermore, major concerns still exist in the management of refractory disease trajectories, seriously impacting patients’ quality of life.

**Objectives:** To capture the major challenges and unmet needs in the treatment of sJIA from the patient perspective.

**Methods:** In the context of METAPHOR project, a PReS/PRINTO initiative aimed to optimize therapeutic approaches to sJIA and macrophage activation syndrome (MAS), an international survey addressed to patients with sJIA was performed, exploring challenges and concerns regarding treatment. The survey was developed by a core team, constituted of 2 senior physicians and 2 patient representatives, and further refined in a focus group by 8 sJIA parents and 1 young adult patient. The survey was then forwarded via the sJIA Foundation to all their members.

**Results:** A total of 139 replies were collected, mainly from United States (64%) and Europe (19%). Almost all participants (94%) were parents or legal guardians. Patient ages were all similarly represented, with a median sJIA duration of 3-5 years. Almost half of patients experienced MAS (44%), and 23% had multiple episodes. At survey time, 38% of patients had an active disease, while 55% and 17% had an inactive disease with and without medications, respectively; 2 patients died. Nearly half patients received treatments for 1-5 years, and 23% for over 6 years. From patient perspective, poor therapy compliance at home is mainly affected by daily injections (90%) and prolonged treatment (51%). During hospitalization, missing daily activities (65%) and difficulties in venous access (47%), further negatively impact on patient compliance. Major concerns regarding steroids include mood changes (64%), weight gain (58%), and growth delay (61%). Starting a new treatment, the majority of parents were scared about the possibility of treatment failure (82%), side effects (77%) and the increased risk of infections (51%). The key treatment goals of the sJIA treatment from patient perspective were complete symptom control (79%), improved quality of life (72%), and reduced steroid exposure (53%). Notably, 54% of patients faced difficulties in accessing necessary medications, primarily due to issues with private insurance or drug unavailability in their country.

**Conclusion:** Treatment of sJIA still represent a challenge for patients. Addressing the patient perspective, including an improvement in the tolerability of home treatment and in the access to medication, a reduction of steroid exposure and less interference with daily life, is crucial for enhancing the quality of care for patients with sJIA.

**Date of birth::** avril 21,

**Patient Consent**

Not applicable (there are no patient data)

**Disclosure of Interest**

None Declared

### Systemic JIA

#### P385 Analysis of protein biomarkers for the prediction of anti-il-1 treatment response in the carra first-line options for systemic Juvenile idiopathic arthritis treatment (Frost) study

##### Michael Matt ^1^, Mariana C. Marques^2^, Lexi Auld^1^, Michael Ombrello^3^, George Tomlinson^4^, Yukiko Kimura^5^, Grant Schulert^1^ and the CARRA FROST Investigators

###### ^1^Rheumatology , Cincinnati Children's Hospital Medical Center, Cincinnati, OH; ^2^NIAMS, NIH, Bethesda, MD, United States; ^3^Translational Genetics and Genomics Section, NIAMS, NIH, Bethesda, MD, United States; ^4^Biostatistics Division, University of Toronto, Toronto, Canada; ^5^Rheumatology, JM Sanzari Children's Hospital, Hackensack Meridian School of Medicine, Hackensack, NJ, United States

####### **Correspondence:** Michael Matt

*Pediatric Rheumatology 2024*, **22(2)**: PReS24-ABS-1306

**Introduction:** IL-1 inhibitors are both widely used and highly effective as first-line therapy for children with systemic juvenile idiopathic arthritis (sJIA); however, the mechanisms underlying response or non-response to these treatments are not well understood.

**Objectives:** We sought to characterize inflammatory cytokine and protein levels in responders and non-responders to IL-1 blockade in a real-world cohort of new-onset sJIA patients in the Childhood Arthritis and Rheumatology Research Alliance (CARRA) Registry.

**Methods:** We identified all patients in the FROST study who were started on anti-IL-1 therapy and had available serum or plasma. IL-1 responders were classified as those patients who fulfilled the Wallace criteria of clinically inactive disease (CID) or modified CID at 6 months, with no change of therapy. If all other criteria for CID were met but inflammatory markers were not obtained, patients were classified as modified CID. Children who did not fulfill these requirements were characterized as non-responders. Cytokine and inflammatory protein levels were measured using a custom Luminex panel. Expression levels were compared using a student t-test or paired t-test.

**Results:** We identified 27 patients with existing baseline biosamples who were started on anti-IL-1 therapy. Of these, 16 were responders and 11 were non-responders. At baseline, median IL-18 levels were higher in non-responders (4381.82 pg/mL, range 10.34- 6615.8) when compared to responders (2877.22 pg/mL, range 304.44-6683.95), but this difference was not significant. There was a non-significant trend towards increased median CXCL9 levels in non-responders (305.05 pg/mL, range 106.72-846.81) compared to responders (256.63 pg/mL, range 106.72-872.55). These differences were reflected in the baseline IL-18/CXCL9 ratio, which revealed a trend towards higher values in responders (12.19) than non-responders (9.18) (p =0.27). Receiver operating curve analysis of this ratio revealed an AUC of 0.60. An IL-18/CXCL9 ratio of less than 3.1 was associated with a 94% specificity but only 27% sensitivity for non-response to anti-IL-1 therapy. Non-responders at baseline had significantly decreased median levels of CCL25 (80.23 pg/mL, range 24.4-130.07 vs 105.81 pg/mL, range 33.06 -151.63, p =0.045) compared to responders. Changes in inflammatory protein levels were also seen over time with anti-IL-1 treatment. In responders, significant decreases were observed in CD163, IFNγ, IL-17a, CD25, GDF-15, IL-23, and IL-18 at 6 months when compared to baseline. At 6 months, non-responders had significantly decreased IL-18 and IL-12 p70 when compared to non-responders at baseline.

**Conclusion:** For children with new-onset sJIA in the FROST cohort, an IL-18/CXCL9 ratio of <3.1 showed high specificity but low sensitivity for non-response to anti-IL-1 therapy. Responders to IL-1 treatment demonstrated significant reductions in numerous inflammatory protein levels. These findings support the hypothesis that subgroups of sJIA patients with different immunologic phenotypes exist, and that immune phenotyping could assist in predicting anti-IL-1 non-response.

**Date of birth::** mai 06, YY

**Patient Consent**

Not applicable (there are no patient data)

**Disclosure of Interest**

None Declared

### Systemic JIA

#### P386 Intestinal barrier biomarkers in adolescents with Juvenile idiopathic arthritis

##### Sophie Hecquet^1^, Marion Thomas^1^, Alice Combier^1^, Julien Wipff^1^, Anne Cauvet^2^, Gertrude Touanga^1^, Frank Verhoeven^3^, Céline Demougeot^4^, Pierre Quartier^5^, Jérôme Avouac^1^, Yannick Allanore^1^

###### ^1^Rheumatology, Cochin Hospital, ^2^Institut Cochin, Paris, ^3^Rheumatology, CHU Minjoz, ^4^ Equipe TAI-IT, UMR 1098 INSERM EFS RIGHT , Besançon, ^5^Rheumatology, (Hôpital Necker, Paris, France

####### **Correspondence:** Sophie Hecquet

*Pediatric Rheumatology 2024*, **22(2)**: PReS24-ABS-1566

**Introduction:** Recent evidence suggests the existence of increased intestinal permeability (IP) and bacterial translocation (BT) in patients with juvenile idiopathic arthritis (JIA). Similar observations have been reported in adult patients with rheumatoid arthritis (RA) and spondyloarthritis. In adults, serum zonulin, a physiological modulator of intestinal epithelial tight junctions, has been identified as an element involved in this hyperpermeability. This modification of the intestinal barrier is thought to lead to BT, for which certain markers, lipopolysaccharide (LPS) and soluble CD14 (sCD14), are increased in RA patients. The aim of this study was to investigate IP, BT and intestinal barrier integrity in JIA patients in transition.

**Objectives:** The aim of this study was to investigate IP, BT and intestinal barrier integrity in JIA patients in transition.

**Methods:** Clinical data and sera from a single centre retrospective cohort of consecutive JIA patients transitioning to adult rheumatology were studied. IP, BT and intestinal integrity were assessed by measuring serum concentrations (ELISA) of zonulin, soluble CD14 and iFABP. These intestinal markers were also measured in the serum of healthy volunteers. Differences between the two groups and correlations were assessed using Student's t-test and Pearson's test or Mann-Whitney and Spearman's tests, depending on the distribution of the subgroups studied.

**Results:** We identified 120 JIA patients (19±5 years) and 40 controls (48.7±9.2 years). The mean age at diagnosis of JIA was 9 (±6) years. there were 18 (15%) oligoarticular JIA, 17 (14%) extensive oligoarticular JIA, 17 (14%) enthesitis-related arthritis (ERA), 16 (13%) systemic JIA, 14 (12%) FR+ polyarticular JIA and 14 (12%) FR- polyarticular JIA.

Concentrations of zonulin (2.5 [1.4-4.6] vs 3.4 [2.2-4.6] ng/ml, p=0.07), iFABP (396 [201-856] vs 637 [290-1235] ng/ml, p=0.07) and soluble CD14 (2275 [1759-2725] vs 2296 [1766-3249] ng/ml, p=0.49) were not different between the JIA and control groups.

The concentration of zonulin was higher in the systemic JIA group compared to all other forms of JIA (5.7 vs 1.8 ng/ml, p=0.01). There was no difference in iFABP and soluble CD14 levels between oligo- and polyarticular or systemic JIA and controls.

There was no correlation between zonulin concentration and CRP or IL-6 in either the all JIA group (r=-0.06, p=0.75) or the systemic JIA group (r=0.4, p=0.39).

There was no difference in zonulin concentration between JIA patients with or without NSAIDs (3.3 [1-5.9] vs 1.7 [1.3-7.2] ng/ml, p=0.9) and those with or without corticosteroids (2.7 [0.4-9.8] vs 1.7 [1.3-7.2] ng/ml, p=0.74). Finally, zonulin levels did not differ between JIA patients on biotherapies and those on csDMARDs (2.1 [0.8-3.3] vs 1.4 [1 .3-7.2] ng/ml, p=0.41), nor between those whose disease had progressed for more or less than 5 years (4 [1.5-9.1] vs 2 [1-4.4] ng/ml, p=0.06).

**Conclusion:** Our findings support data in the literature suggesting an involvement of the intestinal barrier in chronic inflammatory rheumatism. This is the first study in adult JIA patients to demonstrate an increase in markers of intestinal hyperpermeability in systemic forms This increase in IP is not associated with an increase in BT, as recently shown in spondyloarthritis (7), and is not correlated with systemic inflammation. These data may pave the way for new therapies modulating intestinal barrier.

**Date of birth::** février 22

**Patient Consent**

Yes, I received consent

**Disclosure of Interest**

None Declared

### Systemic JIA

#### P387 Worldwide assessment of clinical practice strategies (clips) in still's disease treatment through the jir-clips network: a cost action

##### Francisca Aguiar^1, 2^, Sofia Azevedo^3, 4^, Charlotte Girard‑Guyonvarc’h^5^, Yulia Vyzhga^6^, Filipa O. Ramos^7, 8^, Maurine Jouret^9^, Sohad Hashad^10^, Alain Lefèvre-Utile^11^, Djohra Hadef^12^, Juan Manuel Mosquera Angarita^13^, Karima Daghor-Abbaci^14^, Konstantinos Pateras^15^, Joanne May^16^, Rahma Rahma Guedri^17^, Raquel Faria^18^, Sinisa Savic^19^, Sezgin Sahin^20^, Cristina Lanna^21^, Seza Ozen^22^, Dirk Föll^23^, Michaël Hofer^24, 25, 26^, Sophie Georgin-Lavialle^27, 28^, Katerina Theodoropoulou^24, 29^ on behalf of JIR-CliPS Network

###### ^1^Young Adult and Pediatric Rheumatology Unit, ULS São João; ^2^Faculty of Medicine of Porto Medicine, Porto; ^3^Rheumatology Department, ULS da Região de Aveiro - Hospital Infante D. Pedro; ^4^Egas Moniz Health Alliance, Aveiro, Portugal; ^5^3 Division of Rheumatology, Department of Internal Medicine Specialties, Hôpitaux Universitaires de Genève, Genève, Switzerland; ^6^Pediatric Rheumatology, The Hospital for Sick Children (SickKids) , Toronto, Canada; ^7^ Pediatric Rheumatology Unit, Rheumatology and Pediatric Department, University Hospital Santa Maria; ^8^Instituto de Medicina Molecular, Faculty of Medicine of Lisbon University, Lisboa, Portugal; ^9^ Pediatric Nephrology, Rheumatology, Dermatology Unit,, Hospices Civiles de Lyon, Lyon, France; ^10^Tripoli Children´s Hospital, Tripoli, Libya; ^11^General Pediatrics and Pediatric Emergency Department, Jean Verdier Hospital, Bondy, France; ^12^Pediatrics Department, University of Batna 2, Batna, Algeria; ^13^Hospital Sant Joan de Déu, Esplugues de Llobregat, Spain; ^14^Internal Medicine Department, University of Algiers 1, Algiers, Algeria; ^15^School of Medicine, Volos, Greece, ^16^Children's Hospital for Wales, Cardiff, United Kingdom; ^17^Department of Pediatrics, Béchir Hamza Children’s Hospital of Tunis, Tunes, Tunisia; ^18^ULS Santo António, Porto, Portugal; ^19^Leeds Institute of Rheumatic and Muskuloskeletal Medicine, Leeds, United Kingdom; ^20^Pediatric Rheumatology, Istanbul University Cerrahpasa, Istanbul, Türkiye; ^21^Locomotor Apparatus Department, , Faculdade de Medicina da Universidade Federal de Minas Gerais, Belo Horizonte, Brazil; ^22^ Pediatric Rheumatology, Hacettepe University, Ankara, Türkiye; ^23^Rheumatology and Immunology, University Children's Hospital Münster, Münster, Germany; ^24^Pediatric Immuno-Rheumatology , Lausanne University Hospital, Lausanne; ^25^Pediatric Immuno-Rheumatology , Genève University Hospital, Genève; ^26^Pediatric Immuno-Rheumatology , Hôpital Riviera-Chablais, Rennaz, Switzerland; ^27^Internal Medicine Department, Hôpital Tenon, Sorbonne Université, Assistance Publique-Hôpitaux de Paris; ^28^French National Reference Center of Autoinflammatory Diseases and Inflammatory Amyloidosis, Paris, France; ^29^Immunobiology Deparment, University of Lausanne, Lausanne, Switzerland

####### **Correspondence:** Francisca Aguiar

*Pediatric Rheumatology 2024*, **22(2)**: PReS24-ABS-1643

**Introduction:** Despite the existence of evidence-based or consensus-based recommendations for treatment of Still’s disease, their implementation in real-world settings is challenging.

**Objectives:** To gather real-life clinical practice strategies (CliPS) from physicians worldwide who treat Still’s disease, aiming to create appropriate management plans that support physicians in decision-making processes.

**Methods:** This research was conducted as part of the CliPS project, funded by COST (European Cooperation for Science and Technology), using online questionnaires distributed since September 2022. The study focused on analyzing treatment options for first, second and third lines according to the phenotypic presentation of the disease (mainly systemic or mainly articular). Participants’ choices were also compared based on country income levels.

**Results:** By April 2024, 317 physicians had responded the survey. Most were pediatric specialists (66%, n=210) 19% were adult specialists (n=61) and 15% were both pediatric and adult specialists (n=46). Respondents were from 55 different countries across Europe, Asia, Africa, South America, North America and Oceania. A preliminary analysis of 190 responses revealed distinct treatment strategies based on patient phenotype. For patients with mainly articular symptoms, the most popular first-line treatment strategy was methotrexate, with or without steroids and/or NSAIDs. In cases of non-response, anti-IL-6 and anti-IL-1 agents were chosen as second and third-line treatments, respectively. Conversely, for patients with predominantly systemic features, initiating biologics as a first-line treatment was preferred, with anti-IL-1 agents being more popular than anti-IL-6 agents. In cases of non-response, switching within IL-1 inhibitors or introducing IL-6 inhibitors were the most popular second and third-line treatments, respectively. revealed differences based on country income levels. Physicians in high-income countries tend to prioritize biologics (anti-IL-1 > anti-IL-6 agents) as the first option for treatment induction regardless of patient phenotype while physicians in medium-income countries more frequently opted for methotrexate and steroids for articular and systemic disease presentations, respectively.

**Conclusion:** This study underscores the diverse approaches used by physicians globally, highlighting variations based on patient phenotype and country income levels, which emphasize the need for tailored management plans to optimize patient outcomes and address the challenges in implementing evidence-based recommendations.

**Patient Consent**

Not applicable (there are no patient data)

**Disclosure of Interest**

None Declared

### Systemic JIA

#### P388 The plasma metabolome of systemic JIA is different to non-systemic JIA and non-JIA controls, and partially explained by chronic inflammation

##### Jooa Kwon^1, 2^, Toby Mansell^1, 2^, Boris Novakovic^1, 2^, Melanie R. Neeland^2^, Richard Saffery^2, 3^, Jane Munro^1, 2, 4^, Justine A. Ellis^5^

###### ^1^Paediatrics, University of Melbourne; ^2^Murdoch Children’s Research Institute, Melbourne, Australia; ^3^Paediatrics, Murdoch Children’s Research Institute; ^4^Royal Children's Hospital; ^5^Northern Health Research Development and Governance Unit, Melbourne, Australia

####### **Correspondence:** Jooa Kwon

*Pediatric Rheumatology 2024*, **22(2)**: PReS24-ABS-1364

**Introduction:** Diagnosing systemic Juvenile Idiopathic Arthritis (sJIA) with clinical features alone is challenging, as symptoms are shared between subtypes and other inflammatory diseases. Characterising a molecular signature may therefore be useful for subtype classification in JIA.

**Objectives:** To characterise the plasma metabolome of JIA patients and non-JIA controls, and to determine the extent to which the differences are driven by chronic inflammation measured by glycoprotein acetyls (GlycA).

**Methods:** Nuclear magnetic resonance (NMR) metabolomics of plasma of 73 children with JIA and 18 age- and sex- matched controls was assessed cross-sectionally. Associations between 71 metabolomic biomarkers and JIA, four JIA subtypes, and inflammation (measured by GlycA) were assessed using multivariable linear regression models.

**Results:** Three biomarkers were different between the control and JIA group, with acetate reduced in JIA (mean difference -0.98 standard deviations, [95% confidence interval -1.49, -0.47], *P*_*adj*_ =0.015), while docosahexaenoic acid (DHA) (1.01 [0.47, 1.55], *P*_*adj*_ =0.015) and GlycA (0.91, [0.36, 1.46], *P*_*adj*_ =0.041) were elevated in JIA. Subtype analysis revealed that systemic JIA (sJIA) samples accounted for these changes, with no significant metabolic differences identified in oligoarticular and polyarticular JIA (oJIA and pJIA) relative to controls. A total of 24 of 71 biomarkers were significantly different (*P*_*adj*_ <0.05) in sJIA compared to controls including acetate, DHA and GlycA. No evidence was found that medication status of patients with sJIA (n=20) affected metabolic biomarkers. Of the 24 biomarkers, 6 (polyunsaturated fatty acids (PUFA), sphingomyelins, low density lipoprotein triglycerides (LDL-TG), acetate, histidine, and Omega-6 fatty acids) were significantly associated with levels of the inflammatory marker GlycA.

**Conclusion:** The variation of plasma NMR metabolome of sJIA is the most pronounced relative to non-JIA controls and other JIA subtypes, which show limited evidence of plasma metabolomic disruption. Only a small number of metabolomic profile differences in sJIA were associated with levels of GlycA, indicating a complex relationship between metabolic disruption and chronic inflammation in sJIA patients.

**Trial registration identifying number:** HREC no. 27127Q

**Date of birth::** décembre 2

**Patient Consent**

Yes, I received consent

**Disclosure of Interest**

J. Kwon: None Declared, T. Mansell Employee with: The authors report no competing interests, B. Novakovic Grant / Research Support with: The authors report no competing interests, M. Neeland Grant / Research Support with: The authors report no competing interests, R. Saffery Grant / Research Support with: The authors report no competing interests, J. Munro Grant / Research Support with: The authors report no competing interests, J. Ellis Grant / Research Support with: The authors report no competing interests

### Systemic JIA

#### 389 Distinct expression profile of inflammasome associated genes in systemic juvenile idiopathic arthritis

##### Masaki Shimizu, Shuya Kaneko, Asami Shimbo, Maho Hatano, Futaba Miyaoka, Yuko Hayashi, Hitoshi Irabu, Yuko Akutsu, Masaaki Mori

###### Department of Pediatrics and Developmental Biology, Tokyo Medical and Dental University, Tokyo, Japan

####### **Correspondence:** Masaki Shimizu

*Pediatric Rheumatology 2024*, **22(2)**: PReS24-ABS-1622

**Introduction:** Systemic juvenile idiopathic arthritis (s-JIA) is clinically characterized by arthritis and other systemic features including spiking fever, salmon colored skin rash, hepatosplenomegaly, generalized lymphadenopathy, and polyserositis. The disease course of s-JIA is variable and is divided to three courses, a monocyclic course, multicyclic course and persistent course with chronic arthritis. Although the pathogenesis of s-JIA is still unknown, continual activation of innate immunity and overproduction of innate immune proinflammatory cytokines, including interleukin (IL)-1, IL-6 and IL-18 play an important role in the pathogenesis of s-JIA.

**Objectives:** This study was aimed to clarify the activation profile of inflammasome associated genes in s-JIA

**Methods:** Thirteen patients with s-JIA, 4 patients with macrophage activation syndrome associated with s-JIA and 10 healthy controls (HCs) were enrolled. Whole blood samples were collected at the time of diagnosis of s-JIA or MAS. mRNA were purified and mRNA expressions of inflammasome associated genes were analyzed with Human Inflammasomes RT^2^ Profiler™ PCR Array (Qiagen Sciences).

**Results:** mRNA expressions of 7 genes (NLRC4, AIM2, CASP5, NAIP, TNFSF4, MAPK11, PYDC1) were significantly elevated in 17 s-JIA patients including 4 MAS patients compared to HCs. mRNA expressions of NFKB1A and IRF1 were significantly elevated in MAS patients compared to those of s-JIA patients. mRNA expressions in s-JIA patients were clustered into two distinct groups, PARX7 and CD40LG dominant group and CASP5, CXCL1, NLRP6, MEFV, NFKB1A, NLRP12, CXCL2, NLRC4, CFLAR, AIM2, MYD88 and IRF2 dominant group.

**Conclusion:** Activation of NLRC4 and AIM2 inflammasomes might play an important role in the pathogenesis of s-JIA. Distinct expression profile of inflammasome associated genes in s-JIA might indicate the heterogeneity of s-JIA.

**Trial registration identifying number:** n/a

**Patient Consent**

Not applicable (there are no patient data)

**Disclosure of Interest**

None Declared

### Systemic JIA

#### P390 Juvenile arthritis with systemic onset. the role of interleukin profile in differential diagnosis

##### Valeriya Yussupova on behalf of Elena Zholobova, Seda Kurbanova, Anzhelika Nargizyan, Elena Afonina, Marita Nickolaeva, Saniya Valieva

###### Rheumatology, Sechenov university, Moscow, Russian Federation

####### **Correspondence:** Valeriya Yussupova

*Pediatric Rheumatology 2024*, **22(2)**: PReS24-ABS-1791

**Introduction:** Juvenile rheumatoid arthritis is one of the most common chronic diseases of childhood. One of the most severe subtypes is the systemic form, characterized by fever lasting at least three consecutive days within 2 weeks, arthritis and one or more of the following symptoms: rash, generalized lymphadenopathy, hepato- and/or splenomegaly, serositis. Currently, sJIA is more associated with autoinflammatory diseases due to pathogenetic features. Considering the peculiarities of pathogenesis and the absence of a characteristic characteristic only of sJIA, the study of the interleukin profile is of great interest.

**Objectives:** determining the role of the interleukin profile in children with sJIA and in patients with fever of unknown origin. Identification of the most specific cytokine for the development of sJIA and/or MAS in sJIA. Comparison of the results of the study of the interleukin profile with the severity of the patient’s condition and clinical picture.

**Methods:** The study included 32 patients from the group of fevers of unknown origin, 13 patients were diagnosed with sJIA during the study according to ILAR criteria, 5 patients with an already verified diagnosis of sJIA were in the acute stage, 14 patients were given other diagnoses. Age group from 1 year to 18 years. Patients with the following symptoms were included in the study: fever, rash, lymphadenopathy, arthritis. An inflammatory reaction was observed in the blood: increased C-reactive protein, accelerated ESR, leukocytosis, neutrophilia, some had anemia and thrombocytosis.

**Results:** The highest values were observed when assessing the levels of IL-6, IL-1 and IL-18 types. A relationship has been identified between increased IL-18 levels and sJIA disease

**Conclusion:** It was found that in comparison with other cytokines, namely IL 1, 2, 4, 6, 8, 10, 12 types, as well as TNF-alpha, interferons alpha and gamma, an increase in the level of IL-18 is most specific for sJIA. In the future, this interleukin may be used as a biomarker. Unfortunately, it is not yet possible to compare the severity of the patient’s condition with the level of IL-18, as well as clinical picture data due to the small sample of patients.

**Patient Consent**

Yes, I received consent

**Disclosure of Interest**

None Declared

**References**

1. Sikora KA, Grom AA. Update on the pathogenesis and treatment of systemic idiopathic arthritis. Curr Opin Pediatr. 2011 Dec;23(6):640-6. doi: 10.1097/MOP.0b013e32834cba24. PMID: 22045308; PMCID: PMC3315376.
2. Tayal V, Kalra BS. Cytokines and anti-cytokines as therapeutics--an update. Eur J Pharmacol. 2008 Jan 28;579(1-3):1-12. doi: 10.1016/j.ejphar.2007.10.049. Epub 2007 Oct 25. PMID: 18021769.
3. Yasin S, Fall N, Brown RA, Henderlight M, Canna SW, Girard-Guyonvarc'h C, Gabay C, Grom AA, Schulert GS. IL-18 as a biomarker linking systemic juvenile idiopathic arthritis and macrophage activation syndrome. Rheumatology (Oxford). 2020 Feb 1;59(2):361-366. doi: 10.1093/rheumatology/kez282. PMID: 31326996; PMCID: PMC7571484.
4. Xia Y, Cui P, Li Q, Liang F, Li C, Yang J. Extremely elevated IL-18 levels may help distinguish systemic-onset juvenile idiopathic arthritis from other febrile diseases. Braz J Med Biol Res. 2017 Feb 16;50(2):e5958. doi: 10.1590/1414-431X20165958. PMID: 28225869; PMCID: PMC5343556.

### Systemic JIA

#### P391 Systemic onset juvenileidiopathic arthritis associated with agammaglubolinemia

##### Reza Shiari^1^, Niloofar Shashaani^1^, Vadood Javadi Parvaneh^1^, Khosro Rahmani^1^, Azadeh Zeinab Mirzaee^2^, Mehrnoush Yeganeh^1^, Fatemeh Freshteh Mehregan^3^

###### ^1^Pediatric Rheumatology, Mofid Children Hospital; ^2^Pediatric Rheumatology, Masih Daneshvari Hospital; ^3^Pediatric Rheumatology, Loghman, Tehran, Iran, Islamic Republic Of

####### **Correspondence:** Niloofar Shashaani

*Pediatric Rheumatology 2024*, **22(2)**: PReS24-ABS-1771

**Introduction:** Systemic onset Juvenile Idiopathic Arthritis (SJIA) is a type of juvenile idiopathic arthritis that is presented in children younger than 16 years with fever for at least 3 consecutive days for 2 weeks. Arthritis, generalized lymphadenopathy, erythematous rash, splenomegaly, hepatomegaly, or serositis are other manifestation of SJIA [1]. SJIA has some auto-inflammatory features [2].

**Objectives:** A significant increase is seen in myeloid cells in the blood samples of patients with SJIA [3]. Also, inflammatory markers and cytokines are elevated in SJIA[4]. Genetic abnormalities in the context of SJIA causes disturbance in immunity factors (such as interleukins of 1,6,10, and and induce SJIA [5].

**Methods:** One-year-old boy was referred to hospital for pain and swelling in both knees, lack of height and weight growth, nocturnal fever, and illness during the night. During the day there were no presentations of illness or fever. On physical examination, neurodevelopmental disorder was found but there were no manifestations of serositis, splenomegaly, or pericarditis. Laboratory study was done and an iron deficiency anemia accompanying minor beta thalassemia was found. Laboratory findings are seen in Table 1. Bone scan and bone marrow aspiration were done and both were normal. Systemic onset juvenile arthritis was diagnosed based on history, physical, and laboratory exams.

**Results:** The patient had a history of recurrent acute otitis media with infiltration. So, an immune deficiency work-up was performed on him and his immunoglobulin level was low (Table 1). Then, a genetic test was done on him and autosomal recessive agammaglobulinemia was reported (Table 2). SJIA treatment and IVIG (for treatment of autosomal recessive agammaglobulinemia) was administered to him and finally the patient was discharged in a good condition.

**Conclusion:** As a conclusion, in children with fever and arthritis, SJIA is one of the differential diagnosis and physicians should be aware about the manifestations of immune deficiency such as recurrent infections. In these cases, performing immunologic and genetic tests are needed because the patients may have an underlying genetic disorder cause immune deficiency, such as our case.

**Date of birth::** décembre 3

**Patient Consent**

Not applicable (there are no patient data)

**Disclosure of Interest**

None Declared

**References**

1. Eisenstein EM, Berkun Y. Diagnosis and classification of juvenile idiopathic arthritis. Journal of autoimmunity. 2014; 48: 31-3.
2. De Benedetti F, Brunner HI, Ruperto N, Kenwright A, Wright S, et al. Randomized trial of tocilizumab in systemic juvenile idiopathic arthritis. New England Journal of Medicine. 2012; 367:2385-95.
3. Ruperto N, Brunner HI, Quartier P, Constantin T, Wulffraat N, et al. Two randomized trials of canakinumab in systemic juvenile idiopathic arthritis. New England Journal of Medicine. 2012; 367: 2396-406.
4. Macaubas C, Nguyen K, Deshpande C, Phillips C, Peck A, et al. Distribution of circulating cells in systemic juvenile idiopathic arthritis across disease activity states. Clinical immunology. 2010;134: 206-16.
5. Hinze CH, Fall N, Thornton S, Mo JQ, Aronow BJ, et al. Immature cell populations and an erythropoiesis gene-expression signature in systemic juvenile idiopathic arthritis: Implications for pathogenesis. Arthritis research & therapy. 2010; 12: 1-13.
6. Kessel C, Holzinger D, Foell D. Phagocyte-derived S100 proteins in autoinflammation: putative role in pathogenesis and usefulness as biomarkers. Clinical immunology. 2013; 147: 229-41.

### Systemic lupus erythematosus and antiphospholipid syndrome

#### P393 NGAL, tweak and mcp-1 urine levels as possible biomarkers for disease activity in juvenile systemic lupus erythematosus patients

##### Frederico Rajão Martins ^1^, Rafaela Nicolau^2^, Tiago Beirão^3^, Daniela Santos Oliveira^4, 5^, Sara Ganhão^6^, Francisca Aguiar^6, 7^, Mariana Rodrigues^6, 7^, Cristina Ferreras^8^, Sandra Martins^9, 10^, Rui Farinha^9, 10^, Iva Brito^6, 7^

###### ^1^Rheumatology, University Hospital Centre of Algarve, Faro; ^2^Rheumatology, University Hospital Centre of Tondela- Viseu, Viseu;, ^3^Rheumatology, University Hospital Centre of Vila Nova de Gaia / Espinho, Vila Nova de Gaia; ^4^Rheumatology, University Hospital Centre of São João; ^5^Center for Health Technology and Services Research (CINTESIS), Faculty of Medicine, University of Porto; ^6^Paediatric and Young Adult Rheumatology Unit, University Hospital Centre of São João; ^7^Faculty of Medicine, University of Porto; ^8^Pediatrics; ^9^Pathology, University Hospital Centre of São João; ^10^EPIUnit/ITR - Institute of Public Health and Laboratory for Integrative and Translational Research on Public Health, University of Porto, Porto, Portugal

####### **Correspondence:** Frederico Rajão Martins

*Pediatric Rheumatology 2024*, **22(2)**: PReS24-ABS-1099

**Introduction:** Lupus nephritis (LN) is a serious renal manifestation of systemic lupus erythematosus (SLE), constituting the leading cause of chronic kidney disease in these patients. Absence of reliable serum or urine biomarkers for early diagnosis of LN is troublesome in clinical practice. Urinary cytokines such as TNF-like weak inducer of apoptosis (TWEAK), monocyte chemoattractant protein-1 (MCP-1) and neutrophil gelatinase-associated lipocalin (NGAL) may play a role as early indicators of renal involvement in SLE.

**Objectives:** To describe clinical and laboratorial associations with urine TWEAK, MCP-1 and NGAL levels in juvenile lupus erythematosus (jSLE) patients.

**Methods:** Patients with a definite SLE diagnosis before the age of 21 years-old were consecutively recruited. A randomly selected control group was assembled. Sociodemographic and clinical variables were collected, urine and blood samples were obtained. T-Test, Mann-Whitney-U, Fisher’s exact test and Chi-Square were used. Spearman correlation determined variable association. A p-value <0.05 was considered as significant.

**Results:** Seventy-two patients were analyzed, equally divided in patient and control groups. Both were predominantly female (97.2% and 61.1%, respectively), with similar mean age (24.2±7.7 and 18±5.8 years). Lupus nephritis was frequent (n=15, 41.7%), mainly class IV (n=9, 60%). There were significant differences between groups related to the presence of lymphopenia (60% vs 24%, p<0.001), proteinuria (0.29±0.4 vs 0.03±0.1 g/dL, p<0.001), NGAL (45.4±13.4 vs 26.8±22.5 ng/ml, p<0.001) and TWEAK urine levels (1013.1±475.0 vs 68.6±64.7 pg/ml, p<0.001). Concerning NGAL, higher levels were observed in older patients (ρ =0.31, p=0.01), female sex (z=2.74, p<0.01), proteinuria (ρ=0.30, p=0.03) and anemia (z=2.14, p=0.03). In jSLE patients, NGAL levels were lower in those with higher C3 levels (ρ=-0.5, p<0.001). TWEAK levels were higher in females (z=.03, p=0.04) and associated with proteinuria (ρ=0.41, p<0.01). MCP-1 levels were higher in anti-P-ribossome antibody positive patients (z=2.06, p=0.04), but without other clinical significant associations. In the control group, NGAL levels were higher in patients with leukocyturia (z=0.5, p<0.01) and erythrocyturia (z=0.46, p<0.01) and MCP-1 levels positively correlated with serum creatinine levels (ρ=0.48, p=0.01). None of these urine biomarkers were associated with other urine parameters in jSLE patients.

**Conclusion:** NGAL and TWEAK urine levels positively correlated with urine protein levels in our sample, which may represent a surrogate marker for renal disease activity in jSLE patients. More studies are needed with larger sample size and measurements in blood samples to establish more consistent data concerning their role as possible biomarkers in jSLE patients.

**Patient Consent**

Not applicable (there are no patient data)

**Disclosure of Interest**

None Declared

### Systemic lupus erythematosus and antiphospholipid syndrome

#### P394 Evaluation of the relationship between serum matrix metalloproteinases and their tissue inhibitor levels and capillaroscopy findings in Juvenile systemic lupus erythematosus

##### Ufuk Furkan Özdemir^1^, Özlem Akgün^2^, Selen Duygu Arık^2^, Gülşah Kavrul Kayaalp^2^, Figen Çakmak^2^, Nuray Aktay Ayaz^2^

###### ^1^Division of Pediatrics; ^2^Division of Pediatric Rheumatology, Istanbul University Istanbul Faculty of Medicine, Istanbul, Türkiye

####### **Correspondence:** Özlem Akgün

*Pediatric Rheumatology 2024*, **22(2)**: PReS24-ABS-1738

**Introduction:** Increasing evidence in juvenile-onset systemic lupus erythematosus (jSLE) indicates the presence of microvascular abnormalities. Matrix metalloproteinases (MMPs) such as MMP-2 and MMP-9, and tissue inhibitors of metalloproteinases (TIMPs) including TIMP-1 and TIMP-2, are well-established proteins associated with microvascular damage. Nailfold capillaroscopy examination is a visualization method that can be used to evaluate microvascular damage in jSLE.

**Objectives:** In this study, serum levels of MMP-2, MMP-9, TIMP-1, TIMP-2, and capillaroscopy findings were examined, aiming to evaluate the potential utility of these biomarkers and findings as diagnostic, disease activity, treatment management and prognostic indicators in jSLE.

**Methods:** This cross-sectional and single-center study was conducted at the Department of Pediatric Rheumatology, Istanbul University Istanbul Faculty of Medicine, involving 30 patients diagnosed with jSLE. They were compared with 32 healthy volunteers of similar age, gender, and body mass index distribution. Blood samples were collected from both patient and control groups, and the levels of MMP-2, MMP-9, TIMP-1, and TIMP-2 were determined using the Enzyme-Linked Immunosorbent Assay method. Additionally, capillaroscopy examination was performed on individuals using a videocapillaroscope. The capillaroscopy reading and evaluation were conducted using the classification parameters of the EULAR Working Group on Microcirculation in Rheumatic Diseases.

**Results:** Comparing biomarker levels between the patient and control groups, MMP-9 levels were found to be significantly higher in the patient group than in the control group (p=0.019). For the cut-off point of 2550 ng/L for MMP-9, the sensitivity was 80%, specificity was 53.13%.Comparing capillaroscopy findings between the patient and control groups, the following parameters were found to be significantly higher in the patient group: apical loop width (p=0.026), number of dilated capillaries (p=0.026), number of cross capillaries (p=0.002), number of capillary tortuosity (p=0.042), number of abnormal vessels (p=0.001), and percentage of tortuosity (p=0.049). Cut-off points >16.5 μm for apical loop width (p=0.017), >0.25 for the number of cross capillaries (p=0.002), and >0.38 for the number of abnormal vessels (p=0.001) were statistically significant under the Receiver Operating Characteristics (ROC) curve. Among these variables, the number of cross capillaries had the highest sensitivity, and the number of abnormal vessels had the highest specificity.Examining the relationship between biomarkers and capillaroscopy findings, positive correlations were found between TIMP-1 and apical loop width (r=0.269, p=0.040), number of capillary tortuosity (r=0.369, p=0.004), and presence of tortuosity (r=0.342, p=0.008). Similarly, positive correlations were observed between TIMP-2 and apical loop width (r=0.280, p=0.031), number of cross capillaries (r=0.280, p=0.032), number of capillary tortuosity (r=0.300, p=0.021), and presence of tortuosity (r=0.326, p=0.012).

**Conclusion:** The serum level of MMP-9 and capillaroscopy findings demonstrate microvascular involvement in jSLE. These markers could potentially be utilized for disease diagnosis, prediction of organ involvement, assessment of prognosis, and planning of treatment strategies.

**Patient Consent**

Not applicable (there are no patient data)

**Disclosure of Interest**

None Declared

### Systemic lupus erythematosus and antiphospholipid syndrome

#### P395 Attainment of childhood lupus low disease activity state is associated with improvements in health-related quality of life

##### Lowena Lindsay^1^, Chandni Sarker^2^, Sarah Dyball^3^, Andrea Jorgensen^4^, Michael Beresford^1, 2^, Eve Smith^1, 2^

###### ^1^Alder Hey Children's NHS Foundation Trust Hospital; ^2^Institute of Life Course and Medical Sciences, University of Liverpool, Liverpool; ^3^Division of Musculoskeletal & Dermatological Sciences, University of Manchester, Manchester; ^4^Department of Health Data Science, Institute of Population Health, University of Liverpool, Liverpool, United Kingdom

####### **Correspondence:** Lowena Lindsay

*Pediatric Rheumatology 2024*, **22(2)**: PReS24-ABS-1423

**Introduction:** Childhood-onset systemic lupus erythematosus (cSLE) is associated with poorer health-related quality of life (HRQoL)^1^. In adult-onset SLE, attainment of the treat-to-target (T2T) goal Lupus Low Disease Activity State (LLDAS) has been associated with a significant improvement in HRQoL^2^.

**Objectives:** To determine if attainment of the T2T goal of childhood Lupus Low Disease Activity State (cLLDAS)^3^ is associated with improvements in HRQoL**.**

**Methods:** The analysis included UK JSLE Cohort Study participants diagnosed with cSLE by age 18 years, meeting ≥4 ACR 1997 criteria, and assessed ≥2 times for HRQoL using the Short Form 36-Item (SF-36) survey. The SF-36 provides scores for eight health-related domains, including Emotional Wellbeing, Energy/Fatigue, General Health, Pain, Physical Functioning, Role Limitations due to Emotional Problems, Role Limitations due to Physical Health and Social Functioning. These scores are summarised and normalised against healthy population data producing Physical and Mental Component Scores respectively (PCS and MCS). Clinically derived, real world patient data was used to determine if the cLLDAS target was met at each visit. Markov multi-state modelling assessed the longitudinal impact of cLLDAS attainment on the eight SF-36 HRQoL domain scores, and the PCS and MCS scores.

**Results:** The analysis included 241 patients, median age 13 years (IQR 11.3-14.7), with 85.1% being female. Markov multi-state models indicated that maintenance of cLLDAS between visits was associated with a statistically significant improvement in General Health (HR 2.80, CI 1.16, 6.78) and MCS (HR 2.62, CI 1.04, 6.62). Persistent non-attainment of cLLDAS between visits was associated with poorer Emotional Wellbeing (HR 0.80, CI 0.65, 0.98), Role Limitations due to Emotional Problems [HR 0.71, CI 0.56, 0.91) and MCS scores (HR 0.78, CI 0.62, 0.97) (all *p*<0.05). Patients who were not in cLLDAS but subsequently attained cLLDAS demonstrated a statistically significant improvement in Emotional Wellbeing (HR 1.68, CI 1.10, 2.56), Physical Functioning (HR 1.68, CI 1.02, 2.77), Role Limitations due to Physical Health (HR 2.21, CI 1.23, 3.97) scores and PCS (HR 1.64, CI 1.07, 2.53). Notably, three SF-36 domains were not impacted by either cLLDAS attainment or cLLDAS non-attainment, including: Energy/Fatigue, Social Functioning, and Pain.

**Conclusion:** Attainment and maintenance of cLLDAS over time was shown to have a positive impact on key domains of HRQoL in patients with cSLE. These data support the implementation of cLLDAS as a target to help control disease activity and contribute to improvements in HRQoL. Clearly, however, not all aspects of HRQoL can be improved by attainment of cLLDAS alone; highlighting a need to collaborate with the multidisciplinary team to monitor and address broader goals like fatigue and pain.

**Patient Consent**

Yes, I received consent

**Disclosure of Interest**

None Declared

**References**

1. Brunner H et al. DOI: 10.3899/jrheum.081164
2. Golder V et al. DOI:10.1186/s13075-017-1256-6
3. Smith EMD et al. DOI: 10.1016/j.clim.2023.109296

### Systemic lupus erythematosus and antiphospholipid syndrome

#### P396 How do the clinical, laboratory, treatment features, and outcomes in pediatric patients with lupus nephritis progress over the last 30 years?

##### Deniz Gezgin Yildirim^1^, Nihal Karacayır^1^, Hakan Kısaoglu^2^, Aydan Yekedüz Bülbül^3^, Pınar Garipcin^3^, Hülya Nalçacıoglu^4^, Mukaddes Kalyoncu^2^, Hakan Poyrazoglu^3^, Sevcan A. Bakkaloglu^1^

###### ^1^Pediatric Rheumatology, Gazi University, Ankara; ^2^Pediatric Rheumatology, Karadeniz Teknik University, Trabzon; ^3^Pediatric Rheumatology, Erciyes University, Kayseri; ^4^Pediatric Nephrology, Ondokuz Mayıs University, Samsun, Türkiye

####### **Correspondence:** Deniz Gezgin Yildirim

*Pediatric Rheumatology 2024*, **22(2)**: PReS24-ABS-1500

**Introduction:** Management of the systemic lupus erythematosus (SLE) through new treatment options has improved lupus nephritis (LN) prognosis (1,2).

**Objectives:** The aim of this study was to compare the changes in the demographic, laboratory and treatment characteristics, prognosis, and outcomes of pediatric LN patients over 30 years.

**Methods:** We retrospectively reviewed the medical records of 103 pediatric-onset LN patients. Patients were divided into two subgroups according to the years of LN diagnosis. Group 1 consisted of patients diagnosed with LN between the years of 1993 to 2005, and group 2 consisted of patients diagnosed with LN between the years of 2006 to 2023.

**Results:** The mean age at diagnosis of SLE, age at diagnosis of LN, time to LN development, and mean delay time to diagnosis were significantly higher in group 1 (p<0.001, p<0.001, p=0.049, and p=0.004, respectively). Baseline SLEDAI scores and anti-phospholipid antibody positivity were found to be higher in patients with group 1 (p= 0.040 and p=0.025, respectively). Azathiopurine in maintenance phase was given more frequently in group 1 (p=0.016), while rituximab was more frequently used in group 2 (p=0.042). In both groups, the majority of the patients had proliferative nephritis (class III and/or class IV) (53.5% in group 1 vs. 68% in group 2). Complete renal remission was significantly more common in group 2 (p=0.005), while end-stage kidney disease (ESKD) and death were significantly more common in group 1 (p=0.005 and p=0.001, respectively). Proteinuria and SLEDAI scores at the first visit were independent risk factors for progression to ESKD (p=0.034 and p=0.024).

**Conclusion:** High disease activity scores and proteinuria levels may be the signs of the development of ESKD. Over the last decades, new treatment options such as rituximab have provided better disease outcomes in patients with juvenile-onset LN.

**Date of birth::** janvier 02

**Patient Consent**

Yes, I received consent

**Disclosure of Interest**

None Declared

**References**

1. Smith EMD, Lythgoe H, Midgley A, Beresford MW, Hedrich CM. Juvenile-onset systemic lupus erythematosus: update on clinical presentation, pathophysiology and treatment options. *Clin Immunol* 2019;209:108274.
2. Xipell M, Lledó GM, Egan AC, Tamirou F, Del Castillo CS, Rovira J, et al. From systemic lupus erythematosus to lupus nephritis: The evolving road to targeted therapies. *Autoimmun Rev* 2023;3:103404.

### Systemic lupus erythematosus and antiphospholipid syndrome

#### P397 Assessment of damage in juvenile-onset systemic lupus erythematosus: a single center study

##### Maria Kaleda, Irina Nikishina

###### Pediatrics, V. A. Nasonova Research Institute of Rheumatology, Moscow, Russian Federation

####### **Correspondence:** Irina Nikishina

*Pediatric Rheumatology 2024*, **22(2)**: PReS24-ABS-1118

**Introduction:** Previous studies have shown that approximately 40-65% of patients (pts) with juvenile-onset systemic lupus erythematosus (jSLE) have damage to at least one organ. But data about factors associated with organ damage in jSLE and types of damage currently remain limited.

**Objectives:** To study the frequency of organ damage in pts with jSLE and its relationship with clinical, immunological manifestations, and ongoing therapy.

**Methods:** The retrospective study included all pts with jSLE who undergoing in-patient treatment in our pediatric department during 2022-2023 yy. Diagnosis of SLE was based on SLICC criteria, 2012 y. The organ system damage was measured by the Pediatric Systemic Lupus International Collaborating Clinics/American College of Rheumatology Damage Index (Ped-SDI). Risk factors for damage included demographic data; clinical and immunological manifestations at diagnosis; previous corticosteroid, immunosuppressive, and antimalarial therapies; disease activity assessment (SELENA-SLEDAI was used).

**Results:** Overall of 71 pts (63 girls, 8 boys) were included. The median age at the onset was 12.5 y [10.2; 14.0]. Median disease activity by SLEDAI at the time of jSLE verification was 14 [9;19]. The median age at the time of assessment was 16.0 [14.0;17.65], the duration of the disease – 3.75 years [1.95; 5.6]. 32 patients (45.1%) had SDI ≥ 1, an average of 2.1 years after onset, the minimum time from the onset to the detection of damage was 4 months, the maximum – 5 years. The median SDI score was 2 [1;2], 40.6% had SDI=1, 43.75% - SDI=2, 12.5% – SDI=3, 3.15% – SDI=4. The most frequent type of organ damage was involvement of the musculoskeletal system (62.5% of cases), followed by ocular (53.1% of cases) and neurological (28.1% of cases) disorders. Pts with organ damage were statistically significant more likely to have Coombs’ positive hemolytic anemia (p=0.04), positive antiphospholipid antibodies (p=0.036), received a higher initial dose of GC (Me [IQR] 45 mg/day [33.75;60] with damage, 20 mg/day [10;33.75] without damage, p=0.027), more often received mycophenolate mofetil (p=0.05), but them were less often prescribed hydroxychloroquine (p<0.0001). There were no differences in the frequency of biologics prescribing between the groups with/without damage. Median disease activity by SLEDAI at the time of jSLE verification was 16 [12; 23.25] in pts with organ damage and 12 [9;17] in pts without damage (without statistical difference).

**Conclusion:** We found evidence of cumulative organ damage, as measured by the SDI, in 45.1% of pts with jSLE. In our study was found that pts with Coombs positive hemolytic anemia, positive antiphospholipid antibodies, received a higher initial dose of GC, but less often received hydroxychloroquine are at higher risk to developing organ damage. A statistically significant more frequent administration of mycophenolate mofetil in the group of pts with damage, most likely due to a relatively higher activity of the disease. The most frequent type of organ damage was involvement of the musculoskeletal system and ocular disorders.

**Patient Consent**

Yes, I received consent

**Disclosure of Interest**

None Declared

### Systemic lupus erythematosus and antiphospholipid syndrome

#### P398 Proteomic analysis to identify novel biomarkers in juvenile systemic lupus erythematosus

##### Claudia Simu ^1, 2^, Medeea Badii^3, 4^, Iulia Szabo^5, 6^, Orsi Gaal^3, 4^, Cristina Pamfil^5, 6^, Maria Magdalena Tamas^5, 6^, Laura Damian^6^, Mihaela Sparchez^1, 2^, Tania Crisan^3, 4^, Leo Joosten^3, 4^, Simona Rednic^5, 6^

###### ^1^Department of Mother and Child, “Iuliu Hatieganu” University of Medicine and Pharmacy; ^2^2nd Pediatric Clinic, Emergency Clinical Hospital for Children; ^3^Department of Medical Genetics, “Iuliu Hatieganu” University of Medicine and Pharmacy , Cluj-Napoca, Romania; ^4^Department of Internal Medicine, Radboud Institute for Molecular Life Sciences (RIMLS), Nijmegen, Netherlands; ^5^Department of Rheumatology, “Iuliu Hatieganu” University of Medicine and Pharmacy; ^6^Department of Rheumatology, County Emergency Hospital, Cluj-Napoca, Romania

####### **Correspondence:** Claudia Simu

*Pediatric Rheumatology 2024*, **22(2)**: PReS24-ABS-1329

**Introduction:** Juvenile-onset systemic lupus erythematosus (jSLE) is an autoimmune disease characterized by its heterogeneity and unpredictable course, often marked by substantial damage and disability. Compared to individuals who develop the disease in adulthood, jSLE patients typically experience increased disease activity and damage, requiring more aggressive treatment approaches. While numerous autoantibodies are linked to disease expression, there is a lack of age-specific validation for these potential diagnostic biomarkers.

**Objectives:** This study aimed to identify novel biomarkers that may be used in disease activity in jSLE.

**Methods:** Proximity extension immunoassay (PEA, Olink) was used to assess the serum levels of 92 inflammation proteins in patients with jSLE (n = 19) and age-matched healthy controls (HCs; n = 9). SLE Disease Activity Index (SLEDAI) was assessed in SLE patients to characterize the disease status of all patients. To evaluate the contribution of molecular profiles to the course of disease, we also assessed the association of serum vitamin D levels with differentially expressed proteins.

**Results:** Several circulating proteins related to inflammation were altered in the serum of jSLE patients in relation to HCs. This analysis differentiated two clusters presenting low-inflammatory and high-inflammatory proteomic profiles. Eight upregulated, high-inflammatory proteins were identified in jSLE patients. CXCL6 și CST5 were expressed at significantly higher levels in jSLE group compared to HCs, whereas TNF, HGF, FGF-5, CD244, MMP-10, TNFRSF9 were upregulated, but did not reach statistical significance at p<0.05. Described abnormalities included changes in the expression of IFN-inducible chemokines, alterations in B cell receptor signaling, and shifts in the expression of cytokines associated with leukocyte, neutrophil, and macrophage trafficking. Patients with jSLE exhibited significantly elevated levels of disease activity (SLEDAI score), alongside with renal and hematologic manifestations both at diagnosis and during flares. However, no significant correlation was found with a specific protein profile. Pearson’s correlation analysis between inflammatory proteins in the juvenile SLE cohort and vitamin D levels revealed a significant positive correlation between CX3CL1 and serum vitamin D levels.

**Conclusion:** Through this highly sensitive proteomic analysis, we discovered several new candidate proteins that reveal distinctive molecular patterns in jSLE patients with increased disease activity. This observed proteomic signature underscores the necessity for tailored, age-specific treatment approaches in SLE.

**Date of birth::** septembre

**Patient Consent**

Yes, I received consent

**Disclosure of Interest**

None Declared

**References**

1. Jingquan, H.; Donger, T.; Dongzhou, L.; Xiaoping, H.; Chiyu, M.; Fengping, Z.; Zhipeng, Z.; Yumei, C.; Jie, D.; Lin, K.; Lianghong, Y.; Qianjin, L.; Yong, D. Serum proteome and metabolome uncover novel biomarkers for the assessment of disease activity and diagnosing of systemic lupus erythematosus. Clinical Immunology. 2023(251):109330.
2. Su, K.Y.C.; Reynolds, J.A.; Reed, R. et al. Proteomic analysis identifies subgroups of patients with active systemic lupus erythematosus. Clin Proteom. 2023(20):29.
3. Xie, T.; Dong, J.; Zhou, X. et al. Proteomics analysis of lysine crotonylation and 2-hydroxyisobutyrylation reveals significant features of systemic lupus erythematosus. Clin Rheumatol. 2022(41):3851–3858.

### Systemic lupus erythematosus and antiphospholipid syndrome

#### P399 Systemic inflammatory index and clinical features in pediatric systemic lupus erythematosus: a 10-year retrospective study

##### Esra Baskın^1^, Meraj A. Siddiqui ^2^, Halah O. G. Farhan^2^, Huda T. S. Azzawi^2^

###### ^1^Department of Pediatrics Rheumatology; ^2^Department of Pediatrics, Baskent University Faculty of Medicine, Ankara, Türkiye

####### **Correspondence:** Meraj A. Siddiqui

*Pediatric Rheumatology 2024*, **22(2)**: PReS24-ABS-1798

**Introduction:** Pediatric systemic inflammatory diseases, such as systemic lupus erythematosus (SLE), present significant diagnostic and therapeutic challenges. Recent research has highlighted the potential of the Systemic Inflammatory Index (SII) as a valuable marker for assessing inflammation and disease activity. Studies have shown promise in reflecting the inflammatory burden in various conditions, including autoimmune diseases. However, its application and significance in pediatric SLE, especially in relation to specific organ involvement, remain underexplored.

**Objectives:** To evaluate the clinical features, organ involvement, laboratory and inflammatory markers, biopsy results, and treatment modalities in pediatric patients with SLE, providing a detailed correlation of inflammatory markers with clinical outcomes. This study is the first to compare the SII among different organ involvements in pediatric SLE patients.

**Methods:** This retrospective study was conducted at the Pediatric Rheumatology Department from 2013 to 2023. A total of 104 pediatric patients diagnosed with SLE were included, without discrimination based on gender. Data collected comprised demographic information, clinical features, laboratory markers, and inflammatory markers. Patients were divided into groups based on organ involvement, specifically skin, renal, and CNS involvement, and further categorized into renal involvement with joint involvement, renal involvement with skin, and renal involvement with CNS to compare inflammatory markers (NLR, PLR, and SII) across these groups.

**Results:** This study included 85 (81.7%) female patients and 19 (18.3%) male patients, with a mean age of 17.2 ± 4.7 years at diagnosis. Joint involvement was reported in 78 (75.0%) patients. Organ involvement included skin involvement in 60 (57.7%) patients, renal involvement in 44 (42.3%) patients, and CNS involvement in 9 (8.6%) patients. Inflammatory markers indicated a mean NLR of 2.70, a PLR of 181.25, and an SII of 806.00. Statistical comparisons of these markers across organ involvements indicated no significant differences in PLR values (p > 0.14). NLR analyses showed some variance but were generally non-significant (p > 0.05). Joint involvement had a mean NLR of 2.14 (CI 1.78 to 2.49) compared to 3.66 (CI 1.85 to 5.47) in those without, with a statistically significant difference (p = 0.0370). For SII, renal involvement had a mean of 1092.81 (CI 551.17 to 1634.45) compared to 601.13 (CI 458.51 to 743.76) in those without, with a statistically significant difference (p = 0.0442). Among the further categorized renal groups, none showed statistically significant differences in SII (p > 0.05).

**Conclusion:** This study underscores the significant inflammatory burden in pediatric systemic inflammatory diseases, evidenced by elevated NLR, PLR, and SII levels. The statistically significant higher SII in patients with renal involvement and lower NLR in patients with joint involvement highlight the potential of these markers in reflecting specific organ involvement. The detailed correlation between clinical features, biopsy results, and inflammatory markers provides valuable insights for diagnosis and management.

**Patient Consent**

Not applicable (there are no patient data)

**Disclosure of Interest**

None Declared

### Systemic lupus erythematosus and antiphospholipid syndrome

#### P400 Evaluation of hydroxychloroquine cardiotoxicity in patients with childhood-onset systemic lupus erythematosus

##### Seher Sener^1^, Yusuf Z. Sener^2^, Ezgi D. Batu^1^, Ilker Ertugrul^3^, Ozge Basaran^1^, Yelda Bilginer^1^, Tevfik Karagoz^1^, Seza Ozen^1^

###### ^1^Pediatric Rheumatology, ^2^Internal Medicine, ^3^Pediatric Cardiology, Hacettepe University, Ankara, Türkiye

####### **Correspondence:** Seher Sener

*Pediatric Rheumatology 2024*, **22(2)**: PReS24-ABS-1041

**Introduction:** Hydroxychloroquine, the mainstay of treatment, is recommended for all patients with systemic lupus erythematosus (SLE). Studies conducted in adults have suggested that hydroxychloroquine may have cardiotoxic effects, such as cardiac conduction disorders and myocardial hypertrophy.

**Objectives:** In this study, we aimed to evaluate the effects of hydroxychloroquine on cardiac functions and left ventricular mass in patients with childhood-onset SLE (cSLE).

**Methods:** Fifty cSLE patients treated with hydroxychloroquine were included in the study. These patients were evaluated by echocardiography (ECHO) during their routine outpatient controls. All patients had negative disease activity markers and were clinically in remission at the ECHO visit.

**Results:** The median (min-max) age at diagnosis and the current median age of the patients were 11.5 (3-16) and 17.5 (12-21) years, respectively (F/M=3.2). As comorbidities; six patients (12%) had hypertension, five (10%) had obesity, three (6%) had antiphospholipid antibody syndrome (AFAS), two (4%) had autoimmune hepatitis, and one (2%) had immunodeficiency. All patients were on hydroxychloroquine, %56 (n=28) and on low-dose corticosteroids (5-10 mg/day), and 52% (n=26) were on other disease modifying anti-rheumatic drugs (DMARDs). The median hydroxychloroquine exposure time of the patients was 7.1 (5.2-9.5) years, and the median cumulative hydroxychloroquine dose was 784.8 (509.5-3437.6) grams. No correlation was detected between the parameters of left ventricular ejection fraction, left ventricular mass index, geometry and the cumulative hydroxychloroquine dose (p=0.245, p=0.094, and p=0.146, respectively). In addition, no significant correlation was found between the cumulative dose of hydroxychloroquine and diastolic cardiac parameters (all p>0.005). When we compared the patients who received less than the median cumulative dose of hydroxychloroquine (low dose group) and patients who received more (high dose group), there were again no significant differences according to the ECHO parameters (all p>0.005).

**Conclusion:** Our findings revealed that chronic hydroxychloroquine use in cSLE patients did not adversely increase left ventricular mass or impair left ventricular systolic and diastolic functions. However, it is beneficial for patients using chronic hydroxychloroquine to be evaluated with ECHO at regular intervals to monitor cardiotoxicity.

**Date of birth::** 09.10.1989

**Patient Consent**

Yes, I received consent

**Disclosure of Interest**

None Declared

### Systemic lupus erythematosus and antiphospholipid syndrome

#### P401 Prevalence of herpes zoster in pediatric and young adult patients with systemic lupus erythematosus in japan: using the Japanese large claims data

##### Ryoko Sakai ^1, 2^, Yui Saito^1^, Tomohiro Kawabe^2^, Takako Miyamae^2^

###### ^1^Department of Public Health and Epidemiology, Meiji Pharmaceutical University, ^2^Division of Rheumatology, Department of Internal Medicine, Tokyo Women’s Medical University School of Medicine, Tokyo, Japan

####### **Correspondence:** Ryoko Sakai

*Pediatric Rheumatology 2024*, **22(2)**: PReS24-ABS-1164

**Introduction:** It has been reported that prevalence and incidence of herpes zoster (HZ) in patients with systemic lupus erythematosus (SLE) was higher than general population. In Japan, chickenpox vaccination has been done regularly for children aged 12-36 months since 2014. However, the prevalence of HZ in pediatric and young adult (AYA) patients with SLE have has yet to be known.

**Objectives:** To describe the change in the prevalence of HZ and characteristics of patients with HZ in the patients with SLE using the large Japanese claims data.

**Methods:** We used health insurance reimbursement data of patients aged from 0 to 30 years old from Jan. 2015 to Dec.2020 provided from JMDC. We defined patients who had at least one ICD 10 code for SLE (M321, M329) and prescription of oral corticosteroids (CS) or other medications approved for SLE as patients with SLE. Patients with SLE were divided into pediatric patients under 16 years and AYA patients aged 16 to 30 years old. To define patients who developed HZ, we used ICD10 codes for HZ (B02.x) and prescription of antiviral drugs. We describe the prevalence of HZ, the proportions of prescriptions medications for SLE in each year from 2015 to 2020, and the characteristics of patients who had developed HZ.

**Results:** The number of pediatric SLE patents was 767 (female percentage: 56%), that of the AYA patients was 1,828 (female percentage: 75%). About 70 to 80 percent of patients were treated with oral CS. The proportion of patients treated with high doses of oral CS (prednisolone equivalent dose>30mg/day) has decreased recently in both pediatric and AYA patients (33% in 2015, 28% in 2020 for pediatric patients, 23% in 2015, 15% in 2020 in AYA patients). The most frequently prescribed medications other than CS were mycophenolate mofetil (MMF) in pediatric patients and tacrolimus in AYA patients. The prevalence of HZ in both groups has decreased recently (5.2% in 2015, 1.2% in 2020 in pediatric patients, and 2.8% in 2015, 2.4% in 2020 in AYA patients). It was observed that patients who developed HZ were more likely treated with immunosuppressants such as azathioprine and MMF than those who did not.

**Conclusion:** We showed a change in HZ prevalence and medications use in pediatric and AYA patients with SLE. The prevalence of HZ in both groups has decreased recently (1.2% in 2020 in pediatric patients, 2.4% in 2020 in AYA patients). To prevent the occurrence of HZ under treatment, further investigations using more extensive data will be needed to identify the risk factors of HZ in the future.

**Patient Consent**

Not applicable (there are no patient data)

**Disclosure of Interest**

None Declared

**Reference**

1. Lupus Sci Med., 2022;9(1):e000574.

### Systemic lupus erythematosus and antiphospholipid syndrome

#### P402 Insights into pediatric antiphospholipid syndrome: results from a multicenter Turkish study

##### Selcan Demir^1^, Tutku Doğan Kuzuca^1^, Hatice M. Kacmaz^2^, Nergis Akay^3^, Sıla Atamyıldız Uçar^4^, Veysel Cam^5^, Deniz Gezgin Yıldırım^6^, Nesibe G. Kocamaz^7^, Fatih Haslak^8^, Seyda Doğantan^9^, Merve C. Polat^10^, Halil Kazanasmaz^11^, Raziye B. Taskın^12^, Seher Sener^13^, Gulsah Kılbas^14^, Burcu Bozkaya Yücel^15^, Sezgin Sahin^3^, Selcuk Yüksel^16^, Guzide Aksu^12^, Mukaddes Kalyoncu^11^, Banu Celikel Acar^10^, Semanur Ozdel^7^, Kubra Ozturk^8^, Sevcan Bakkaloglu^6^, Betül Sözeri^4^, Ayse Balat^2^, Ozgur Kasapcopur^3^, Seza Ozen^5^

###### ^1^Department of Pediatric Rheumatology, Eskisehir Osmangazi University, Eskisehir; ^2^Department of Pediatric Rheumatology, Gaziantep University Faculty of Medicine, Gaziantep; ^3^Department of Pediatric Rheumatology, Istanbul University Cerrahpasa, ^4^Department of Pediatric Rheumatology, University of Health Sciences; Ümraniye Training and Research Hospital, Istanbul; ^5^Department of Pediatric Rheumatology, Hacettepe University; ^6^Department of Pediatric Rheumatology, Gazi University; ^7^Department of Pediatric Rheumatology, Ankara Etlik City Hospital, Ankara; ^8^Department of Pediatric Rheumatology, İstanbul Medeniyet University Prof. Dr. Süleyman Yalçın City Hospital; ^9^Department of Pediatric Rheumatology, Basaksehir Cam and Sakura City Hospital, Istanbul, ^10^Department of Pediatric Rheumatology, Ankara Bilkent City Hospital, Ankara; ^11^Department of Pediatric Rheumatology, Karadeniz Technical University, Trabzon; ^12^Department of Pediatric Rheumatology, Ege University, Izmir; ^13^Department of Pediatric Rheumatology, Adana City Hospital, Adana; ^14^Department of Pediatric Rheumatology, Pamukkale University, Denizli; ^15^Department of Pediatric Rheumatology, Samsun Training and Research Hospital, Samsun; ^16^Department of Pediatric Rheumatology, Canakkale Onsekiz Mart University, Canakkale, Türkiye

####### **Correspondence:** Selcan Demir

*Pediatric Rheumatology 2024*, **22(2)**: PReS24-ABS-1729

**Introduction:** Antiphospholipid syndrome (APS) is a systemic autoimmune disease characterized by thrombotic events and/or pregnancy morbidity in the presence of persistently positive antiphospholipid antibodies (aPLs), namely anticardiolipin (acl), beta2 glycoprotein I (B2GPI) and lupus anticoagulant (LA). The presentation and progression of APS in children often exhibit distinct differences compared to the adult form of the disease.

**Objectives:** To describe the frequency of thrombotic and non-thrombotic clinical manifestations, laboratory findings, treatment approaches, and prognosis in patients with pediatric APS.

**Methods:** This retrospective study included pediatric patients diagnosed with APS according to the updated Sapporo criteria or the 2023 ACR-EULAR APS criteria.

**Results:** Sixty-five pediatric APS patients from 15 centers in Turkey were included in the study. Among them, 51 (78.5%) were female and 14 (21.5%) were male. The median age at the time of diagnosis was 13.1 years (0.3-17 years). Of these patients,15 (23.1%) were diagnosed with primary APS, while 50 (76.9%) had an underlying autoimmune disease, predominantly SLE (98%) and undifferentiated connective tissue disease (UCTD) (2%). Thrombotic manifestations included venous thrombosis in 36 patients (55.3%), arterial thrombosis in 14 patients (24.6%), small-vessel thrombosis in 7 patients (10.7%), and mixed arterial and venous thrombosis in 4 patients (6.1%). Three patients (4.6%) presented with catastrophic APS (CAPS). Non-thrombotic manifestations were also significant, with hematologic disorders occurring in 42 patients (64.6%). Among these, 37 patients (56.9%) had thrombocytopenia, 20 (30.8%) had autoimmune hemolytic anemia, and 1 (1.5%) patient had microangiopathic hemolytic anemia. Additionally, 23 patients (28.2%) had non-thrombotic neurologic disorders, and 6 patients (9.2%) had cardiac valve disease. Laboratory findings showed the presence of aCLs in 52 patients (80%), anti-β2GPI in 36 patients (55%), and LA in 40 patients (61.5%) at the time of diagnosis. All patients with venous thrombosis received long-term anticoagulation therapy. Among patients with arterial thrombosis, 12% received no treatment, 44% received antiaggregation therapy, and 44% received anticoagulation therapy with or without concomitant antiaggregation therapy. 50 patients (76.9%) received at least one immunosuppressive drug for APS manifestations, including glucocorticoid therapy (oral or intravenous) for all 50 patients, Rituximab for 9.2%, CYC for 26.2%, and MMF for 10.8% of those patients. During the follow-up, 4 patients (6.1%) experienced recurrent thrombosis, and 2 patients (4.6%) died due to thrombotic events.

**Conclusion:** Despite its rarity in children, APS can manifest severely and lead to significant morbidity and mortality. While classification criteria have been primarily designed for adults, there is a clear indication for the future development of pediatric-specific criteria. Comprehensive understanding of its clinical features, treatment options, and outcomes is crucial for effective management.

**Date of birth::** septembre

**Patient Consent**

No, I have not received consent

**Disclosure of Interest**

None Declared

### Systemic lupus erythematosus and antiphospholipid syndrome

#### P403 Childhood-onset systemic lupus erythematosus patients living in eartquake area are at risk for higher disease activity and development of major organ involvement

##### Hatice Melisa Kaçmaz ^1^, Sezgin Şahin^2^, Oya Köker^3^, Nergis Akay^2^, Dilara Ünal^4^, Zeynep Özaslan^5^, Yasemin Demir Yiğit^6^, Zelal Aydın^7^, Ozan A. Akın^8^, Rabia M. Kışla^9^, Aydan Yekedüz Bülbül^10^, Seher Şener^11^, Nesibe G. Kocamaz^12^, Ahmet Girgeç^1^, Nihal Şahin^5^, Fatih Haşlak^7^, Selcan Demir^8^, Semanur Özdel^12^, Erdal Sağ^4^, Ayşenur Paç Kısaarslan^10^, Metin Kaya Gürgöze^6^, Özgür Kasapçopur^2^, Ayşe Balat^1^

###### ^1^Paediatric Rheumatology , Gaziantep University Faculty of Medicine, Gaziantep; ^2^Paediatric Rheumatology , İstanbul University - Cerrahpaşa Faculty of Medicine; ^3^Paediatric Rheumatology , Marmara University Faculty of Medicine, İstanbul; ^4^Paediatric Rheumatology , Hacettepe University Faculty of Medicine, Ankara; ^5^Paediatric Rheumatology , Kocaeli University, Faculty of Medicine, Kocaeli; ^6^Paediatric Rheumatology , Fırat University Faculty of Medicine, Elazığ; ^7^Paediatric Rheumatology , İstanbul Medeniyet University Prof. Dr. Süleyman Yalçın City Hospital, İstanbul; ^8^Paediatric Rheumatology , Eskişehir Osmangazi Faculty of Medicine, Eskişehir; ^9^Paediatric Rheumatology , Adana Çukurova University Faculty of Medicine, Adana; ^10^Paediatric Rheumatology , Erciyes University Faculty of Medicine, Kayseri; ^11^Paediatric Rheumatology , Adana City Hospital, Adana; ^12^Paediatric Rheumatology , Ankara Etlik City Hospital, Ankara, Türkiye

####### **Correspondence:** Hatice Melisa Kaçmaz

*Pediatric Rheumatology 2024*, **22(2)**: PReS24-ABS-1654

**Introduction:** A devastating earthquake with a magnitude of 7.8 on the Richter scale struck southeast region of Turkiye on February 6, 2023. Widespread damage occurred in 11 cities, covering an area of about 350,000 km2, approximately the size of a European country. Approximately 15% of the Turkish population was affected by this earthquake, making it the deadliest disaster in Turkiye in the last millennium. There are only two studies in the literature that evaluate the role of emotional trauma, stress, sun exposure and activity in triggering lupus flares following the earthquake. Additionally, these studies have been conducted on a very limited number of systemic lupus erythematosus (SLE) patients.

**Objectives:** We aimed to compare the frequency of clinical and serologic activity flares in childhood onset SLE (cSLE) patients living in the earthquake region and cSLE patients living away from the epicenter at two time points (T1: within 45 days after the earthquake; T2: from 45^th^ day to the last visit) following the earthquake) in Turkiye.

**Methods:** cSLE patients who have been followed up in 12 pediatric rheumatology clinics and were living in the earthquake region (Gaziantep, Hatay, Adıyaman, Şanlıurfa, Adana, Osmaniye, Diyarbakır, Malatya, Elazığ) were included in our study. cSLE patients living outside the epicenter have been enrolled as control patients. The demographic, clinical, laboratory and treatment characteristics of cSLE patients before and after the earthquake were retrospectively recorded from patient files. Besides, all the patients living in the earthquake zone were interviewed regarding accommodation status and duration (prefabricated container, tent, house), post-earthquake medication supply, access to hospitals, hospitalizations, and changes in treatment.

**Results:** A total of 142 cSLE patients were included in this multicenter study from 12 pediatric rheumatology clinics: Sixty-one patients living in the earthquake zone (Group 1) and 81 patients living away from the earthquake's epicenter (Group 2). Within 45 days after the earthquake, higher rates of fever(p=0.012), constitutional manifestations (p=0.001), malar rash (p<0.001) were observed in cSLE patients living in the earthquake region compared to those living away from epicenter.Dose escalation of corticosteroid (p<0.001) and change in immunosuppressive medication (p=0.004) were more frequently required in Group 1 compared to Group 2. From the 45^th^ day of earthquake until the last visit, proteinuria (p<0.001), kidney involvement (p<0.001), class III/IV lupus nephritis (p<0.001), and hospitalization (p<0.001) were more frequently developed in Group 1 than in Group 2.

**Conclusion:** During the early period after the earthquake, children with SLE who lived in the affected area were at a higher risk of developing fever, constitutional manifestations, malar rash, and hypocomplementemia. These patients also required more frequent increases in corticosteroid doses and changes in immunosuppressive therapies. Longer follow-ups of these patients revealed that major organ involvement and hospitalization were more frequently pronounced in the late post-earthquake period.However, more extensive studies are needed to explore the underlying reasons responsible for this unfavourable course of cSLE after the earthquake.

**Date of birth::** 01.01.1984

**Patient Consent**

Not applicable (there are no patient data)

**Disclosure of Interest**

None Declared

**References**

1. Chou CT, Hwang CM. Changes in the clinical and laboratory features of lupus patients after the big earthquake in Taiwan. Lupus. 2002;11:109-13.
2. Wallace DJ, Metzger AL . Can an earthquake cause flares of rheumatoid arthritis or lupus nephritis? Arthritis Rheum 1994; 37: 1826–1828.

### Systemic lupus erythematosus and antiphospholipid syndrome

#### P404 The relationship between disease perception, disease course, and quality of life in childhood systemic lupus erythematosus

##### Nihal Şahin^1^, Kübra Uçak ^1^, Sıla Atamyıldız Uçar^2^, Hafize E. Sönmez^1^, Betül Sözeri^2^

###### ^1^Pediatric Rheumatology, Kocaeli University, Kocaeli, ^2^Pediatric rheumatology, Health Sciences University, Istanbul Umraniye Training and Research Hospital, İstanbul, Türkiye

####### **Correspondence:** Kübra Uçak

*Pediatric Rheumatology 2024*, **22(2)**: PReS24-ABS-1197

**Introduction:** Systemic lupus erythematosus (SLE) is a chronic autoimmune disease characterized by recurrent flares affecting various organs such as skin, joints, and kidneys. One of the treatment goals for SLE is to preserve and improve patients' quality of life. Patients' perceptions of their disease and it’s treatment can influence their quality of life.

**Objectives:** This study aims to evaluate disease perceptions in childhood SLE patients and their interaction with patient-parent quality of life.

**Methods:** Patients diagnosed with childhood SLE according to the SLICC 2012 classification were included between January 2023 and November 2023. Disease perception was assessed using the Brief Illness Perception Questionnaire, while quality of life was evaluated using PedsQL in patients and WHOQOL-BREF in parents.

**Results:** The study included 32 patients and 32 parents. Of the patients, 25 were female (78.1%), and 7 were male (21.9%), with a female-to-male ratio of 3.6. The mean age was 17.7±2.2 years, the median age at diagnosis was 13.9 years (range: 1.23 to 17.25 years), and the median disease duration was 4.2 years (range: 0.25 to 13.42 years). The most common organ involvement was musculoskeletal involvement in 21 patients (65.6%), followed by skin involvement in 19 patients (59.4%), and kidney and hematological involvement in 16 patients each (50%). No patient had neuropsychiatric involvement. The median SLEDAI score at the last visit was 2 (range: 0-18). The mean score of the Brief Illness Perception Questionnaire was 36±10.3. When categorized, 20 patients (62.5%) had low threat perception, 9 (28.1%) had moderate, and 3 (9.4%) had high threat perception. There was a significant negative correlation between patients' disease perception and quality of life (r=0.576, p=<0.001). However, there was no correlation between any subscale of parental quality of life and patients' disease perception (p=>0.05). The number of patients with a PedsQL total score below the cut-off value (<70) was 18 (56.3%), while 14 parents (43.8%) had a WHOQOL-BREF general health score below the cut-off value (<60).

**Conclusion:** Although disease perception in childhood SLE was not associated with disease activity, it was negatively correlated with patient quality of life. According to these results, approaches aimed at improving patients' perceptions of SLE and its treatment may be beneficial for enhancing their quality of life.

**Date of birth::** avril 09,

**Patient Consent**

Yes, I received consent

**Disclosure of Interest**

None Declared

**Reference**

1. Dr. Hafize Emine Sönmez

### Systemic lupus erythematosus and antiphospholipid syndrome

#### P405 Long term neurodevelopmental outcome of children, born to mothers with antiphospholipid syndrome

##### Mojca Zajc Avramovic ^1, 2^, Tadej Avcin^2, 3^, Aleš Ambrožič^4^, Vesna Fabjan^5^, Mateja Sever^6^

###### ^1^Pediatric clinic, University Medical Center Ljubljana, ^2^Medical faculty, University of Ljubljana, ^3^Pediatric clinic, ^4^Department for rheumatology, ^5^Maternity hospital, University Medical Center Ljubljana, ^6^University Medical Center Ljubljana, Ljubljana, Slovenia

####### **Correspondence:** Mojca Zajc Avramovic

*Pediatric Rheumatology 2024*, **22(2)**: PReS24-ABS-1734

**Introduction:** Children of mothers with antiphospholipid syndrome (APS) are generally considered healthy, but recent studies have shown that they are prone to cognitive abnormalities.

**Objectives:** The aim of this study was to assess long-term neurodevelopmental outcomes in a Slovenian cohort of children born to mothers with APS.

**Methods:** Consecutive children of mothers with APS, referred to the University Medical Centre Ljubljana for immunologic evaluation between 2001 and 2023 were included. Demographic data, perinatal data and data related to the mother's disease were recorded from electronic medical records. Antiphospholipid antibodies (aPL) were checked at birth and at follow-up. Neurologic and cognitive development was assessed at every visit. In addition, the children were evaluated by a psychologist using general health questionnaire, the Child Behavior Check List and age appropriate Wechsler Intelligence Scale.

**Results:** 108 children (56 boys) were included. The mean follow-up time was 4.5 years (3months – 20 years). Ninety-two percent of mothers had obstetric APS, while 8 % had thrombotic APS. Eleven percent of mothers also had systemic lupus erythematosus and 2% had Sjogren syndrome. During pregnancy 94.4 % of mothers were taking Aspirin and 87.2 % were also taking low molecular weight heparine. At birth 35.1% of newborns had positive aPL – out of these 57.9% had only aCL IgG, 36.8 % had only antibGPI IgG and 5.2% were positive for both aCL and antibGPI. Lupus anticoagulant was not assessed at birth. None of the children developed SLE, APS or thrombosis. Neurodevelopmental abnormality was detected in 36 % of all patients, in 50.1% of those followed for at least 3 years and in 54 % of those followed for at least 6 years. Neurodevelopmental abnormalities are shown in the Picture 1. The mean age of 37 children further assessed by psychologist was 4.8 years, 51,4 % were girls and 48,6 % boys. Seventy-two percent were born full-term, 25 % were born near-term (34-37 weeks of gestation, while one child was an extreme premature baby (born before 29 weeks of gestation), Out of these 37 children,13.5 %, exhibited significant behavioural and emotional abnormalities (emotional problems, interpersonal relations, sleeping difficulty, distancing, pervasive developmental problem, internalisation), while 33.3 % had significantly dicrepant cognitive profile – there was a discrepance between verbal intelligence quotient (VIQ) and performance intelligence quotient (PIQ) In 21.2% children VIQ>PIQ, in 12.1 % children PIQ>VIQ. Elements of ADHD were noted in 36.3%, one third of which was pretem. Speech/verbal abnormalities were detected in 21,2 % and 6,1 % children had problems with graphomotorics. Sixteen percent were independently reffered to psychologist and 48,6 % were followed in special neuro-development outpatient.

**Conclusion:** Children, born to mothers with APS are at risk of nerodevelopmental abnormalities, when they reach school-age, in more than 50 % of cases. Close follow-up and early interventions would be beneficial.

**Patient Consent**

Yes, I received consent

**Disclosure of Interest**

None Declared

### Systemic lupus erythematosus and antiphospholipid syndrome

#### P406 Comparison of clinical differences between juvenile- and adult-onset systemic lupus erythematosus in russian patients: retrospective study

##### Elena Aseeva, Sergei Soloviev, Irina Nikishina, Maria Kaleda

###### V. A. Nasonova Research Institute of Rheumatology, Moscow, Russian Federation

####### **Correspondence:** Irina Nikishina

*Pediatric Rheumatology 2024*, **22(2)**: PReS24-ABS-1706

**Introduction:** The differences in clinical features of different age-onset systemic lupus erythematosus (SLE) patients (pts) deserve more attention from rheumatologists. Juvenile onset of SLE (jSLE) results in more aggressive disease and worse outcomes, and is currently considered as a separate phenotype.

**Objectives:** To compare clinical, laboratory parameters and complications in adult- onset SLE (aSLE) and jSLE.

**Methods:** 400 pts with SLE (F/M 363:37, mean age 34,2±11.5 years, mean disease duration 106,3±91,9.0 months) were included in retrospective single-center study. Diagnosis of SLE was based on SLICC criteria 2012. Patients were divided into 2 groups: the 1^st^ - jSLE (100 pts, 25%), the 2^nd^ - aSLE (300 pts, 75%). The clinical, laboratory and immunological parameters, SLEDAI 2K, SDI were assessed.

**Results:** The chronological relationship between the onset of the disease and previous insolation was more often in jSLE in contrast to aSLE (53% vs. 8%, OR = 14.2; 95% CI 7.94–25.6; p = 0.0001). Pts with jSLE more often had at onset lupus nephritis (26% vs. 13%, p = 0.002), hematological disorders (14% vs. 5%, p = 0.0001) and the central nervous system involvement (6% vs 0%, p<0.0001). Clinical manifestations included more frequent detection of photosensitivity (45% vs. 9%, OR = 8.62; 95% CI 4.91–15.1; p < 0.0001), classic malar (butterfly) rash (48% vs. 29%, OR = 2.22; 95% CI 1.4–3.54; p = 0.0001), generalized erythematous rash (25% vs. 10%, OR = 3.0; 95% CI 1.66–5.41; p = 0.0001), mucosal lesions (47% vs. 28%, OR = 2.24; 95% DI 1.41–3.58; p = 0.0001). We observed the development of irreversible organ lesions in 72% of patients with jSLE compared with 50% of pts with aSLE and a higher SDI (2.3±1.5 vs. 1.5±1.2 points, p = 0.01).

**Conclusion:** The following differences were identified in pts with jSLE compared with aSLE: previous insolation as a trigger for the development of the disease; more frequent predisposition to the development of lupus nephritis and central nervous system involvement, even at disease onset; significant number of patients (up to 72%) with irreversible organ damages and higher damage index.

**Patient Consent**

Yes, I received consent

**Disclosure of Interest**

None Declared

### Systemic lupus erythematosus and antiphospholipid syndrome

#### P407 Juvenile lupus-like with negative serology: a clinical dilemma and diagnostic challenge

##### Alhanouf Alsharif ^1^, Abdulaziz Almutairi^2^, Emtenan Basahl^1^, Jameela Kari^3^, Abdulaziz Alshathri^4^, Mohammed Shalaby^3^, Mohammed Nashawi^5^

###### ^1^Faculty of Medicine, King AbdulAziz University, Jeddah, ^2^Pediatric Rheumatology, King Saud Medical City, Riyadh , ^3^Pediatric Nephrology, King AbdulAziz University, Jeddah, ^4^Pediatric Nephrology, King Saud Medical City, Riyadh , ^5^Pediatric Rheumatology, King AbdulAziz University, Jeddah, Saudi Arabia

####### **Correspondence:** Alhanouf Alsharif

*Pediatric Rheumatology 2024*, **22(2)**: PReS24-ABS-1103

**Introduction:** Systemic lupus erythematosus (SLE) is an autoimmune disorder that can affect various organs. Juvenile-onset SLE (jSLE) may be more severe than the adult-onset form, but the diagnosis and classification remain challenging due to the complex nature of the condition and its resemblance to other conditions. Antinuclear antibodies (ANA) are the immunological hallmark of SLE, but their limited specificity poses challenges. The 2019 EULAR/ACR criteria introduced a weighted multi-criteria system for classifying SLE, using ANA as an entry criterion. However, seronegative SLE, in which a patient's clinical features and laboratory values are consistent with SLE but their ANA serology test is negative, is a rare subtype of SLE that has been reported in several cases worldwide.

**Objectives:** To evaluate the clinical presentation, treatment responses, and outcomes of seronegative jSLE in two pediatric cases, aiming to highlight the implications of seronegativity on diagnosis and management, and to advocate for revised diagnostic criteria that accommodate seronegative manifestations.

**Methods:** This case series analyzed two pediatric cases of seronegative jSLE. Each patient was assessed through comprehensive clinical evaluations, laboratory tests, and imaging studies. Treatment regimens included various immunosuppressives, and the patients were monitored to document clinical responses and any changes in ANA status.

**Results:** The first patient, a 13-year-old girl, presented with headache, generalized edema, hypertension, nephrotic-range proteinuria, and seizures. She was diagnosed with lupus nephritis based on the renal biopsy revealing combined diffuse and membranous lupus nephritis of class IV and V, and an MRI brain scan showing evidence of lupus cerebritis. Despite these severe presentations, her ANA tests repeatedly returned negative. Whole exome sequencing was performed to rule out monogenic causes, which also returned negative. However, the patient responded well to treatment and showed significant improvement. The second patient, an 11-year-old girl was admitted with acute kidney injury with headache, vomiting, and hypertension. The diagnosis was supported by renal biopsy, which showed features resembling Class IV lupus nephritis. Similar to the first case, her ANA and other antibodies related to SLE were negative, and whole exome sequencing did not reveal any genetic abnormalities. Her management involved intensive treatment with high-dose prednisolone, cyclophosphamide, and rituximab. Despite these aggressive interventions, she progressed to chronic kidney disease stage V within four months of diagnosis.

**Conclusion:** These cases underscore the limitations of relying on ANA positivity as a key diagnostic criterion for SLE. The presence of lupus-like symptoms in ANA-negative patients calls for a re-evaluation of current diagnostic protocols using a more inclusive set of criteria that does not centralize immunological serology, thereby enabling accurate diagnosis even in its absence to prevent misdiagnosis and delayed treatment.

**Date of birth::** mars 11, Y

**Patient Consent**

Yes, I received consent

**Disclosure of Interest**

None Declared

### Systemic lupus erythematosus and antiphospholipid syndrome

#### P408 Preliminary results: a ten-year review of pediatric lupus - clinical characterization, therapeutic approaches, and outcomes in an intensive care unit

##### Ana Luiza G. Cunha, Matheus S. França, Leonardo M. M. de Castro, Thaynara A. M. Araújo, Isabela T. B. Matias

###### Hospital Infantil João Paulo II, Belo Horizonte, Brazil

####### **Correspondence:** Ana Luiza G. Cunha

*Pediatric Rheumatology 2024*, **22(2)**: PReS24-ABS-1361

**Introduction:** Patients with systemic lupus erythematosus (SLE) often require intensive care unit (ICU) admission, a circustance associated with a poor prognosis. There are limited studies focusing on pediatric populations in this context.1,2

**Objectives:** To investigate the causes of ICU admission, therapeutic interventions, and mortality rates in juvenile systemic lupus erythematosus (jSLE).

**Methods:** Retrospective observational study from 2014 to 2023 at a tertiary pediatric center. Patients were identified using the ICD-10 code M32.1 and conformance with SLE classification criteria was verified, using SLICC 2012 and EULAR/ACR 2019 criteria. Data collected included SLEDAI-2K and Pediatric Index of Mortality 3 (PIM-3) scores at the time of ICU admission, indications for ICU transfer, treatments administered, and mortality outcomes.

**Results:** The study included 45 patients with a total of 129 hospital admissions. Among them,18 patients (31.8%) required ICU care ate least once, accounting for a total of 29 ICU admissions. These patients exhibited significantly prolonged median hospitalalization (44 days, IQR 21-92) and more frequent hospital admissions (median 3.5, IQR 1-6) compared to non-ICU patients (median 6 days, IQR 2-17; median 2.3 admissions, IQR 1-3). There were no significant differences observed in gender distribution (85% vs. 77.8%) or age (mean 10.6 years, standard deviation [SD] 3.6 vs. SD 2.3) between the groups.

Most ICU admissions resulted from active jSLE (86.2%), including autoimmune hemolytic anemia (27.6%), lupus nephritis (24.1%), neurological manifestations of lupus (13.8%), and macrophage activation syndrome (6.9%); with the remainder due to infectious diseases (13.8%). There were four deaths (13.8%) during the study period, two due to sepsis, one refractory autoimmune hemolytic anemia, and one stroke. Comparing survivors and non-survivors, there were no significant differences in SLEDAI-2K scores (20.32, SD 15.2 vs. 22.75, SD 14.75, p=0.768) or PIM-3 scores (-4.07, SD 0.84 vs. -1.32, SD 3.58, p=0.223). Therapeutic approach in the ICU setting included methylprednisolone (82.7%), antibiotics (72.4%), cyclophosphamide (37.9%), intravenous immunoglobulin (34.5%), and plasmapheresis (27.5%). Despite high antibiotic use, only four patients had positive cultures. ICU admissions for infections (p=0.08), requiring mechanical ventilation (p=0.052), or necessitating plasmapheresis (p=0.052), showed a trend toward higher mortality, but the small number of deaths limits the assessment of these outcomes.

**Conclusion:** This decade-long observational study delineates the clinical management and outcomes of jSLE in ICU settings. The uniform administration of aggressive therapies across the majority jSLE ICU patients reflects the critical nature of their health status.

**Patient Consent**

Not applicable (there are no patient data)

**Disclosure of Interest**

None Declared

**References**

1. Aragón CC, Ruiz-Ordoñez I, Quintana JH, et al. Clinical characterization, outcomes, and prognosis in patients with systemic lupus erythematosus admitted to the intensive care unit. Lupus. 2020 Aug;29(9):1133-1139.
2. Al-Mayouf SM, Fallatah R, Al-Twajery M, et al. Outcome of children with systemic rheumatic diseases admitted to pediatric intensive care unit: An experience of a tertiary hospital. Int J Pediatr Adolesc Med. 2019 Dec;6(4):142-145.

### Systemic lupus erythematosus and antiphospholipid syndrome

#### P409 Delay in diagnostic, referral and treatment in pediatric patients with systemic lupus erythematosus: a systematic review

##### Fernando García-Rodríguez^1^, Marcela Álvarez^2^, Ana V. Villarreal-Treviño^1^, Virginia Ramírez-Nova^3^, María F. Ramírez-Flores^4^, Adolfo Hernandez-Garduno^5^, Alfonso Gastelum-Strozzi^6^, Ingris Peláez-Ballestas^7^ and Latin American Group for the Study of Lupus (GLADEL)

###### ^1^Departamento de Pediatría, Universidad Autónoma de Nuevo León, Monterrey, Mexico; ^2^Sección de Reumatología infantil, Hospital de Niños Ricardo Gutiérrez, Buenos Aires, Argentina; ^3^Servicio de Pediatria, Hospital General de México, "Dr. Eduardo Liceaga"; ^4^Programa de Estudios Combinados en Medicina (PECEM), Facultad de Medicina, Universidad Nacional Autónoma de México; ^5^AHS Health Consulting; ^6^Instituto de Ciencias Aplicadas y Tecnología, Universidad Nacional Autónoma de México; ^7^Rheumatology Unit, Hospital General de México, "Dr. Eduardo Liceaga", Mexico city, Mexico

####### **Correspondence:** Fernando García-Rodríguez

*Pediatric Rheumatology 2024*, **22(2)**: PReS24-ABS-1269

**Introduction:** Juvenile systemic lupus erythematosus [jSLE] comprises about 20% of all SLE cases. The delay of diagnosis, referral, and treatment of patients with jSLE is one of the most important determinants that impact the outcomes.

**Objectives:** To systematically review the available evidence regarding the delay of diagnosis, referral, and treatment of patients with jSLE.

**Methods:** Electronic searches were conducted in Scopus, PubMed, and Web of Science for studies published up to July 15^th^, 2023. Inclusion criteria were observational studies, including patients with jSLE who reported delays in diagnosis, referral, or treatment. The project followed the PRISMA guidelines and was divided into phases including 1) the consensus of a group of experts (pediatric rheumatologists, pediatrician, general practitioner, methodologist, anthropologist) on the keywords for an advanced query string search; 2) the screening of titles and abstracts by peer review; 3) the full-text review and data collection in a predesigned format; 4) the synthesis of the information focused on identifying the variables related to delay in care; 5) the critical appraisal and assessment of risk of bias of selected studies using the JBI Checklist [1]. To ensure consistency, reported variables of interest in years were converted into months.

**Results:** After the initial search, 309 pediatric studies were screened, and 10 met the inclusion criteria. Nine were retrospective studies, 4 from Europe, 3 from Asia, and 3 from the Americas. The median JBI quality score was 6 (interquartile range [IQR] 5 – 7).

Six studies reported the time to diagnosis of jSLE; one categorized the delay in diagnosis, and another reported the time from jSLE onset to lupus nephritis (LN) diagnosis. None of the articles presented a definition of jSLE diagnostic delay, but one categorizes this delay according to adult-onset SLE literature.

The estimated mean delay in diagnosis was 3.7 months (6 studies, 568 patients). One study reported that diagnosis was delayed more than 3 months in 62.6% of the 1555 patients included. The delay in treatment was reported in 2 studies, which stated that therapy started at diagnosis. Only one study reported a referral delay (1.4 months, IQR 7 days - 3.6 months).

The barriers identified for timely diagnosis were male gender, low anti-nuclear antibodies [ANA] titers, mild manifestations, living in a state with a low density of pediatric rheumatologists, meeting criteria for discoid rash, younger age of onset, and low income.

**Conclusion:** Despite the number of studies reporting patients with jSLE, there is a lack of information on the delay of care in these patients. As a result, there is no consensus on defining the delay in diagnosis, referral, or treatment in jSLE patients. Despite the limited evidence, it is evident that delay of care is a significant issue that needs to be urgently addressed.

**Trial registration identifying number:** This protocol was registered with the International Prospective Register of Systematic Reviews (PROSPERO CRD42023456508).

**Date of birth::** mars 27, Y

**Patient Consent**

Not applicable (there are no patient data)

**Disclosure of Interest**

None Declared

**Reference**

1. Moola S, Munn Z, Tufanaru C, Aromataris E, Sears K, Sfetcu R, Currie M, Qureshi R, Mattis P, Lisy K, Mu P-F. Chapter 7: Systematic reviews of etiology and risk. In: Aromataris E, Munn Z (Editors). Joanna Briggs Institute Reviewer's Manual. The Joanna Briggs Institute, 2017. Available from https://reviewersmanual.joannabriggs.org

### Systemic lupus erythematosus and antiphospholipid syndrome

#### P410 Effect of immunomodulatory therapies in patients with antiphospholipid syndrome

##### Patricia Morán Álvarez ^1^, Veronica Garcia-Garcia^2^, Aliuska Palomeque^2^, Maria Jesus Garcia Villanueva^2^, Emiliano Marasco^1^, Fabrizio De Benedetti^1^, Claudia Bracaglia^1^

###### ^1^Ospedale Pediatrico Bambino Gesù, Rome, Italy, ^2^Hospital Universitario Ramon y Cajal, Madrid, Spain

####### **Correspondence:** Patricia Morán Álvarez

*Pediatric Rheumatology 2024*, **22(2)**: PReS24-ABS-1359

**Introduction:** Antiphospolipid Syndrome (APS) is an autoimmune disease characterized by thrombotic and/or obstetric events in the presence of antiphospholipid antibodies (aPL), (IgG/IgM anticardiolipin (aCL), IgG/IgM β2-glycoprotein I (aβ2GPI) and/or lupus anticoagulant (LA). Recent data suggests that aPL levels may decrease over time due to the natural history of the disease or to the treatments. Therefore, monitoring of aPL levels may represent a strategy to evaluate disease activity and response to therapies.

**Objectives:** To investigate the effect of immunomodulatory (IMM) therapies in aPL titers in patients with APS.

**Methods:** We carried out a descriptive, observational, cross-sectional study of adults and pediatric population with a diagnosis of APS. aPL testing was carried out in all patients from diagnosis every 3-4 months for at least 2 years. Laboratory parameters, clinical and demographic data was retrieved and analyzed.

**Results:** Forty-six patients, 13 children and 34 adults with a diagnosis of primary APS were included. The median age at disease onset was 10 years (range: 6 months – 16 years) and 46 (range: 19-82), respectively. Thirty-six patients presented with at least one thrombotic event (TE) (21 arterial and 20 venous). Twenty-three and 27 patients received antiaggregant and/or anticoagulant therapy, respectively. A total of 36 (77%) patients developed at least one non-TE. Eleven (23%) patients were positive for only 1 aPL subtype, 21 (47%) for 2 aPL subtypes and 13 (28%) for all 3 subtypes, showing the highest frequency IgG aCL (55%) and IgG aβ2GP (53%). Lower rates were identified for IgM aCL (46%) and IgM aB2GP (42%) and LA (38%). Eighteen (38%) had ANA positivity and 4 (8%) ENA. Adult population showed significantly higher rates of positive IgM and ANA (p <0.05). Regarding the treatment, 10 (77%) children received at least one IMM: 10 mycophenolate, 3 rituximab and 2 cyclophosphamide (CYC) compared to only one (3%) adult, who was treated with CYC (p=0.001). At the end of follow-up, children were negative for aPL with a higher frequency compared to adult population (67% vs 12%, p=0.001]. Finally, a stepwise regression analysis demonstrated IMM as independent factor for aPL negative [OR 16.6, 95% CI (2.6-102.83) p=0.002].

**Conclusion:** patients under IMM treatment showed higher rates of negative aPL at the end of follow-up. An immunosuppressive strategy should be considered in the treatment of APS. However, further studies may be necessary to confirm our data and to investigate the role of IMM as a potential therapeutic strategy in the management of APS.

**Date of birth::** octobre 15

**Patient Consent**

No, I have not received consent

**Disclosure of Interest**

None Declared

### Systemic lupus erythematosus and antiphospholipid syndrome

#### P411 Evaluation of antiphospholipid antibody positivity in patients with systemic lupus erythematosus

##### Hülya Ercan Emreol^1^, Ozan A. Akın^2^, Dilara Ünal^1^, Veysel Çam^1^, Emil Aliyev^1^, Yağmur Bayındır^1^, Selcan Demir^2^, Ezgi D. Batu ^1^, Halide Ö. Başaran^1^, Seza Özen^1^, Yelda Bilginer^1^

###### ^1^Department of Paediatric Rheumatology , Hacettepe University Faculty of Medicine, Ankara; ^2^Department of Paediatric Rheumatology , Eskişehir Osmangazi University Faculty of Medicine, Eskişehir, Türkiye

####### **Correspondence:** Hülya Ercan Emreol

*Pediatric Rheumatology 2024*, **22(2)**: PReS24-ABS-1720

**Introduction:** Antiphospholipid syndrome (APLS) is characterised by thromboembolic events in the presence of antiphospholipid antibodies (APLA). The clinical presentation of isolated autoantibody positivity is unknown, non-pathogenic transient elevations of APLA are known in the paediatric age group.

**Objectives:** In our study; we aimed to evaluate the relationship between isolated APLA positivity and the clinical features of SLE patients together with the laboratory parameters which were not included in the antiphospholipid syndrome classification criteria, during their 5-yr follow up. We also aimed to summarise the course of autoantibody positivity, other laboratory parameters and thromboembolic conditions during admission and follow-up of patients who developed secondary APLS.

**Methods:** Our study included 122 patients from two centres who were followed up between 2005 and 2023. Others features of the patients at the disease onset and upto 5-yr follow up were evaluated retrospectively.

**Results:** 51 (41.8%) of 122 patients had APLA positivity at least once, during thier follow-up without having APLS diagnosis. 70.5% (n=36), 21.5% (n=11), and 7.8% (n=4) of the patients had one, two, and three APLA positivity, respectively. APLA positivity at the time of diagnosis was present in 26.2% (n=32) of the patients while 27.4% (n=31) of them had in the 1st year, 21.7% (n=22) of them had in the 2nd year, 13.8% (n=13) of them had in the 3rd year, 11.6% (n=10) of them had in the 4th year and, 19.1% (n=13) of them had in the 5th year. The mean duration of neutralization of APLA positivity was 5.58 (±3.8) months. During the period of APLA positivity, 50.9% (n=26) of the patients had C3 deficiency and 56.8% (n=29) had C4 deficiency. Nine patients had a diagnosis of APLS during follow up. The mean age of patients who developed APLS was 15 (±2.7) years, which was higher than the mean age of patients who did not develop APLS. At the time of APLS diagnosis of these 9 patients, 66.6% (n=6) of them had aCL IgG, 77.7% (n=7) of them had aCL IgM, 77.7% (n=7) of them had B2GPI IgG, 66.6% (n=6) of them had B3GPI IgM, and 77.7% (n=7) of them had LA positivity were detected. APLAs became negative on average 16.5 months in 6 out of 9 patients. Although the mean age of patients with isolated APLA positivity was higher than that of patients with secondary APLS, there was no statistically significant (p= 0.1). There was no statistical difference between the two groups in terms of C3, C4 and sedimentation rates, whereas CRP value was slightly higher in the group with isolated APLA positivity (11.2 mg/L (±1.3) vs 5.3 mg/L (±1.7) p=0.1).

**Conclusion:** SLE is a complex autoimmune disease characterized by different clinical manifestations and a large number of autoantibodies that help to discriminate the clinical phenotype of patients. The data on the clinical presentation of APLA positivity in juvenile SLE is very limited and further multicentre studies are needed to highlight the importance of this specific subgroup.

**Date of birth::** février 08

**Patient Consent**

Not applicable (there are no patient data)

**Disclosure of Interest**

None Declared

### Systemic lupus erythematosus and antiphospholipid syndrome

#### P412 Serum interferon-alpha levels in Japanese childhood-onset systemic lupus erythematosus: associations with disease activity and clinical characteristics

##### Asami Shimbo, Maho Hatano, Futaba Miyaoka, Shuya Kaneko, Yuko Hayashi, Hitoshi Irabu, Yuko Akutsu, Masaaki Mori, Masaki Shimizu

###### Department of Pediatrics and Developmental Biology, Graduate School of Medical and Dental Sciences, Tokyo Medical and Dental University, Tokyo, Japan

####### **Correspondence:** Asami Shimbo

*Pediatric Rheumatology 2024*, **22(2)**: PReS24-ABS-1733

**Introduction:** Type I interferons (IFN-I) play a significant role in systemic lupus erythematosus (SLE) pathogenesis. Various methods, including serum IFNα levels, interferon-stimulated genes (ISGs), and IFNα2 measurement by Single-Molecule Array (Simoa), have been explored to assess IFN-I due to observed low serum IFNα levels in SLE. However, ethnic variations in IFN-I activity are reported in SLE, with higher levels observed in Asian populations compared to Caucasian populations.

**Objectives:** To evaluate serum IFNα levels in Japanese childhood-onset systemic lupus erythematosus (cSLE) patients and investigate their association with disease activity and clinical characteristics.

**Methods:** Serum IFNα levels were measured in 40 samples from 24 cSLE patients and 10 age-matched healthy controls (HC) using the Human IFNα All Subtype ELISA Kit (PBL Assay Science). All patients were of Japanese ethnicity and met the 2019 EULAR/ACR classification criteria for systemic lupus erythematosus. Disease activity was determined by Systemic Lupus Erythematosus Disease Activity Index-2000 (SLEDAI-2K), and inactive disease was defined by Lupus Low Disease Activity State (LLDAS) or Disease Outcomes in Rheumatology (DORIS) criteria. Flare-ups were defined as instances requiring either treatment escalation or addition. Clinical data were retrospectively collected from medical records. Correlations were assessed using Spearman's correlation, and statistical significance between the two groups was determined using the Mann-Whitney test.

**Results:** The median serum IFNα levels were 20.53 pg/mL in cSLE and 1.7 pg/mL in HC, significantly higher in cSLE (p < 0.0001). A cutoff value of > 4.69 pg/mL effectively distinguished cSLE from HC (sensitivity 92.5%, specificity 100%). IFNα showed a significant correlation with SLEDAI-2K (r=0.56, p=0.0002). Patients with rash ( p= 0.0008), mucosal ulcers ( p= 0.0206), fever ( p= 0.0003), and proteinuria ( p= 0.0412) had significantly elevated IFNα levels, which correlated with low leukocyte count, lymphopenia, anemia, thrombocytopenia, hyperferritinemia, and hypocomplementemia. Even in the inactive disease group, IFNα exceeded the cutoff of 4.69 pg/mL in 75% of patients. IFNα levels during inactive disease correlated with the number of flare-ups throughout the disease course, suggesting its potential as a more sensitive indicator of disease activity than SLEDAI-2K.

**Conclusion:** Serum IFNα levels are significantly associated with increased disease activity and specific clinical manifestations in Japanese patients with cSLE, highlighting its potential utility as a sensitive biomarker for disease monitoring and management.

**Patient Consent**

Yes, I received consent

**Disclosure of Interest**

None Declared

### Systemic lupus erythematosus and antiphospholipid syndrome

#### P413 Cardiac valvular involvement in childhood-onset systemic lupus erythematosus: a case series and a concise review of the literature

##### Reima Bakry^1^, Shahad Alansari^2^

###### ^1^Pediatric Rheumatology , Maternity and Childre Specialized Hospital , Jeddah; ^2^Pediatric Rheumatology , Hera General Hospital, Makkah, Saudi Arabia

####### **Correspondence:** Reima Bakry

*Pediatric Rheumatology 2024*, **22(2)**: PReS24-ABS-1668

**Introduction:** Cardiac valvular disease is a significant concern in childhood-onset systemic lupus erythematosus (cSLE), an autoimmune condition characterized by systemic inflammation. SLE can affect the heart, leading to various complications, including inflammation and damage to the heart valves, namely mitral and aortic valves. The present of cardiac involvement may carry a poor prognostic risk and may associated with higher disease activity and major organ involvement. In this series, we will review clinical manifestations, echocardiographic findings, autoimmune workup, and medications used and correlate them with disease activity and outcomes.

**Objectives:** This report aims to illuminate the cardiac valvular involvement in childhood-onset systemic lupus erythematosus(cSLE), and a brief literature review

**Methods:** Four consecutive children with systemic lupus erythematosus (SLE) who fulfilled the Systemic Lupus International Collaborating Clinics (SLICC) criteria were evaluated retrospectively. Demographic data, clinical manifestations, SLE disease activity index (SLEDAI), laboratory, radiological, and echocardiographic findings, medications, and the outcome on the last visit were reviewed.

**Results:** Four patients (three females) were analyzed. The median of disease onset was (11.5) years (IQR1.5), and the median age of diagnosis was (12) years (IQR 1.75). All patients had cardiac valvular disease, affecting mainly the mitral valve; only one patient had Libman sacks endocarditis. Three patients (75%) had major organ involvement other than cardiac, including the renal, respiratory, haematological and central nervous system, accounting for (75%), (50%), (75%), and (50%), respectively. All patients have positive anti-nuclear antibodies (ANA), anti-smith antibodies, double-strand DNA antibodies (dsDNA), low C3 and C4 levels, and negative anti-SSA and anti-SSB antibodies. Antiphospholipid (APL) antibodies were sent for three patients; all of them had positive for one or more (two had lupus anticoagulant (LA), one had anti-cardiolipin (ACL), and one had anti-beta 2 glycoprotein 1 (B2GPI)) antibodies. All patients were treated aggressively either with corticosteroids, conventional, biological disease-modifying antirheumatic drugs or a combination with variable responses. The median SLEDAI score was (37, IQR 23.5) which indicates severe disease activity. Unfortunately, there were two (50%) deaths due to pulmonary hemorrhage.

**Conclusion:** Although limited data are available on cardiac valvular disease in childhood-onset systemic lupus erythematosus (cSLE), our research aligns with the published findings. Understanding the link between cardiac involvement and cSLE can help with early diagnosis and appropriate management, potentially leading to better patient outcomes.

**Patient Consent**

No, I have not received consent

**Disclosure of Interest**

None Declared

### Systemic lupus erythematosus and antiphospholipid syndrome

#### P414 Cardiac and pulmonary manifestations in juvenile systemic lupus erythematousus: data from a single centre cohort at great ormond street hospital for children

##### Inga Z. Turtsevich, Sandrine Compeyrot-Lacassagne, Muthana Al Obaidi, Elena Moraitis

###### Paediatric Rheumatology, Great Ormond Street Hospital NHS Foundation Trust, London, United Kingdom

####### **Correspondence:** Inga Z. Turtsevich

*Pediatric Rheumatology 2024*, **22(2)**: PReS24-ABS-1794

**Introduction:** Systemic lupus erythematosus (SLE) is more severe in children compared to adults, associated with greater damage over time. A number of studies have focused on the cardiac and pulmonary involvement in adult SLE, with only a few studies investigating the paediatric cohorts.

**Objectives:** To describe the demographic, clinical and laboratory characteristics of patients with juvenile systemic lupus erythematosus (jSLE) with cardiac and pulmonary manifestations.

**Methods:** A retrospective study of a cohort of paediatric patients with jSLE from the largest paediatric rheumatology department in the UK between January 2000 and March 2021. Demographics, clinical and laboratory characteristics and specifically data on pulmonary and cardiac involvement were collected at diagnosis and follow-up.

**Results:** A total of 70 patients were included in the study with a mean age of 14.3±2.9 years and disease duration of 4.5±3.5 years, predominantly females (82.6%). The cohort was multi-ethnic, with a preponderance of Black (34.3%) followed by Asian (21.4%) and Caucasian participants (12.8%). Thirty-one participants had either cardiac or pulmonary manifestation. The majority of them (54.8%) were found to have cardiac manifestations at the diagnosis with pericardial effusion being the most frequent (52.9%) followed by valvulopathy (47.1%) and pericarditis (17.6%). Four participants had pulmonary manifestations of JSLE: half had pleural effusion, one had interstitial lung disease and one had mediastinal lymphadenopathy.

Ten patients (32.3%) had mixed cardiac and pulmonary manifestations, with pericardial, pleural effusion, and valvulopathy, with abnormal lung function tests being the most common pathologies found in this subgroup, associated with significant morbidity.

The prevalence of cardiac and/or pulmonary involvement was higher among the Black African ethnicity (43.3%), followed by Asian (16.7%) and white British background (10%).

All patients received steroids, and rituximab was given to 70.6% in the cardiac manifestation group, 75% in the pulmonary manifestation group, and 66.7% in the mixed subgroup. No deaths were reported.

**Conclusion:** Our data are consistent with previous reports in paediatric and adult populations. Prospective studies are needed to characterise the natural history of acute cardiac/pulmonary manifestations of jSLE, and to identify risk factors for poor outcomes.

**Patient Consent**

Not applicable (there are no patient data)

**Disclosure of Interest**

None Declared

**References**

1. Al-Adhoubi NK, Bystrom J. Systemic lupus erythematosus and diffuse alveolar hemorrhage, etiology and novel treatment strategies. Lupus. 2020;29(4):355-63.
2. Harrison MJ, Zuhlke LJ, Lewandowski LB, Scott C. Pediatric systemic lupus erythematosus patients in South Africa have high prevalence and severity of cardiac and vascular manifestations. Pediatr Rheumatol Online J. 2019;17(1):76.
3. Alamoudi OS, Attar SM. Pulmonary manifestations in systemic lupus erythematosus: association with disease activity. Respirology. 2015;20(3):474-80.
4. Chang JC, Xiao R, Mercer-Rosa L, Knight AM, Weiss PF. Child-onset systemic lupus erythematosus is associated with a higher incidence of myopericardial manifestations compared to adult-onset disease. Lupus. 2018;27(13):2146-54.

### Systemic lupus erythematosus and antiphospholipid syndrome

#### P415 First case report of omani child with prkcd mutation with monogenic Systemic Lupus Erythematosus (SLE)

##### Amani Al-Ghadani^1^, Ruqaiya Al Jashmi^2^, Safiya Al Abrawi^2^

###### ^1^Pediatrics Residency Training Program, Oman Medical Specialty Board; ^2^Child Health, Royal Hospital, Muscat, Oman

####### **Correspondence:** Amani Al-Ghadani

*Pediatric Rheumatology 2024*, **22(2)**: PReS24-ABS-1105

**Introduction:** SLE is characterized by autoantibodies targeting various cellular components (1). Genetic factors have been implicated in the pathogenesis of juvenile-onset SLE (2). Protein kinase C delta plays a crucial role in regulating the lymphocytes and autoreactive B cells. Case reports have demonstrated that mutations in PRKCD are associated with juvenile-onset SLE (3). There is a limited literature describing patients with early-onset SLE with PRKCD mutations. This case report describes two siblings with early-onset and severe SLE.

**Objectives:** This study aims to provide a detailed description of the clinical characteristics of a young child with early onset SLE who carry mutation in the PRKCD gene.

**Methods:** We reviewed the clinical presentation and disease progression using the medical records of two siblings who presented with juvenile-onset SLE, and utilized Whole Exome Sequencing (WES) to identify mutations in the genes associated with the disease.

**Results:** An 8-year-old girl was born to a first cousins presented at the age of 13 months with hepatosplenomegaly and discoid rash. Laboratory tests revealed elevated ESR, low complements, positive ANA, anti-dsDNA and ENA. She was started on steroids, MMF, and hydroxychloroquine. In 2020-2023 she experienced severe and frequent flares, she was treated with rituximab and Belimumab. WES showed homozygous non-coding variant of uncertain significance in the PRKCD gene (c.629-125G>T). The family history is significant for an elder brother who presented at the age of 2 years with hepatosplenomegaly and lymphadenopathy. Initial evaluation was negative. WES was negative, it was done outside Oman and possibly PRCKD gene was overlooked. He suffered from severe lupus nephritis with worsening renal function. He developed end stage renal disease required hemodialysis. Unfortunately, he passed away in 2020 in a local hospital with pulmonary edema and sepsis.

**Conclusion:** PRKCD is a crucial protein involved in the development and function of B-cells and programmed cell death. Research using animals has shown that a deficiency in PRKCD can lead to an increased risk of autoimmune diseases. In humans, a lack of PRKCD has been identified as a genetic cause of juvenile-onset SLE and has been linked to autoimmune lymphoproliferative syndrome and recurrent infections. Subsequent case reports have demonstrated a wide range of presentations and phenotypes (4). Further studies are needed to aid in the management of patients with such complex and severe presentations.

**Date of birth::** février 07

**Patient Consent**

Yes, I received consent

**Disclosure of Interest**

None Declared

**References**

1. Namjou B, Kilpatrick J, Harley JB. Genetics of clinical expression in SLE. Autoimmunity. 2007 Jan 7;40(8):602–12
2. Belot A, Kasher PR, Trotter EW, Foray A, Debaud A, Rice GI, et al. Protein Kinase Cδ Deficiency Causes Mendelian Systemic Lupus Erythematosus With B Cell‐Defective Apoptosis and Hyperproliferation. Arthritis Rheum. 2013 Aug 26;65(8):2161–71
3. Batu ED. Monogenic systemic lupus erythematosus: insights in pathophysiology. Rheumatol Int. 2018 Oct 15;38(10):1763–75.
4. Jefferson L, Ramanan AV, Jolles S, Bernatoniene J, Mathieu AL, Belot A, et al. Phenotypic Variability in PRKCD: a Review of the Literature. J Clin Immunol. 2023 Nov 5;43(8):1692–705.

### Systemic lupus erythematosus and antiphospholipid syndrome

#### P416

##### **Spectrum of extrarenal systemic manifestations in juvenile systemic lupus erythematosus with and without renal involvement**

###### Basil Fathalla^1, 2^

####### ^1^Allergy, Immunology and Rheumatology , Children's Hospital of Michigan, Detroit, ^2^College of Medicine , Central Michigan University, Mt Pleasant , United States

*Pediatric Rheumatology 2024*, **22(2)**: PReS24-ABS-1207

**Introduction:** Extrarenal systemic manifestations in juvenile Systemic Lupus Erythematosus (jSLE) can precede renal disease and be life-threatening yet there are no guidelines for management of these manifestations.

**Objectives:** To highlight impact of extrarenal manifestations in a cohort of jSLE from one tertiary center.

**Methods:** A retrospective review since the year 2020 – present time after IRB approval.

**Results:** We report 29 patients with jSLE (10 males and 19 females). One patient has a 47,XXY karyotype. Patients are African American (19), Caucasian (7), and others (3). Mean age at diagnosis was 12.3 years (range 7 – 16) and mean age at time of last visit was 14.5 years (range 10 – 18). Six patients had a first degree relative with SLE and two siblings have a *DNASE1LE* pathogenic variant. Patients fulfilled the 2019 EULAR/ACR classification for SLE. All had extrarenal manifestations whereas 20 patients had renal disease: Twelve patients had renal involvement at disease onset with three patients requiring repeated biopsies. Two patients had acute kidney injury but did not have renal biopsies due to unstable clinical status. Six patients had renal disease at a median of three years after SLE onset. Renal biopsies showed class II lupus nephritis (n=3), class III (n=6), class IV (n=3), class V (n=5), class III+V (n=1), class IV+V (n=1) and EXT2 positive focal segmental glomerulosclerosis (FSGS) (n=1).Extrarenal manifestations included musculoskeletal (n=15), hematologic (n=12), mucocutaneous (n=9), pulmonary (n=8), cardiac (n=7), gastrointestinal/liver (n=6), ophthalmologic (n=3), Sjogren (n=3), and CNS involvement (n=2). Life threatening manifestations included pulmonary hemorrhage (alone or as pulmonary-renal syndrome), cardiac arrythmia, and hematologic manifestations including macrophage activation syndrome. Atypical initial presentations included life-threatening pulmonary hemorrhage with aplastic anemia, thrombotic thrombocytopenia purpura with active COVID 19 infection, and EXT2 positive FSGS progressing to renal failure. Cardiac disease included: pericarditis, coronary ectasia, atrial flutter, premature ventricle contractures, and others. All patients received systemic steroids in addition to mycophenolate mofetil, Plaquenil, IVIG, plasma exchange, anakinra, cyclophosphamide, rituximab and belimumab. Two patients have end stage renal disease. Most had hypertension and cushingoid features, growth delay (n=4, but one had primary growth hormone deficiency requiring growth hormone),avascular necrosis and compression fracture (n=2), steroid induced diabetes (n=2, one had diabetic ketoacidosis), and posterior reversable encephalopathy syndrome (n=1).

**Conclusion:** Immune suppressive treatments used for jSLE extrarenal manifestations can mitigate natural history and lead to more subtle markers of future renal involvement. Life threatening extrarenal manifestations dominated the disease course with need to escalate therapy in many of our cases.

**Patient Consent**

Not applicable (there are no patient data)

**Disclosure of Interest**

None Declared

### Systemic lupus erythematosus and antiphospholipid syndrome

#### P417 Autoantibodies in children with systemic lupus erythematosus

##### Rinat Raupov^1, 2^, Eugenia Bondarenko^2^, Elvira Kalashnikova^2^, Alina Mochalova ^2^, Mikhail Kostik^2^

###### ^1^H. Turner National Medical Research Center for Children’s Orthopedics and Trauma Surgery; ^2^Saint-Petersburg State Pediatric Medical University, Saint-Petersburg, Russian Federation

####### **Correspondence:** Mikhail Kostik

*Pediatric Rheumatology 2024*, **22(2)**: PReS24-ABS-1234

**Introduction:** systemic lupus erythematosus (SLE) is a heterogeneous autoimmune disease that affects various systems, and is characterized by an unpredictable course of the disease. SLE is characterized by the production of multiple autoantibodies, which are associated with the symptoms of the disease, and in some cases, have a prognostic role.

**Objectives:** to study the spectrum of autoantibodies and their associations with clinical and laboratory manifestations of SLE in children

**Methods:** 155 children (132 girls and 23 boys) are included in the study. Autoantibodies against dsDNA, Sm, RNP, SSA, SSB, MCV, RNP, nucleosomes, histones and their association with clinical and laboratory manifestations were analyzed.

**Results:** The mean age of the patients was 12.9 (10.3-15.0) years. Patients who had anti-dsDNA were significantly more likely to have skin lesions (86% vs 68%, p=0.023), hepatomegaly (24% vs 6%, p=0.031), fever (65% vs 35%, p=0.004), thrombocytopenia (42% vs 17%, p=0.010). The presence of anti-Sm antibodies was associated with Raynaud's phenomenon (41% vs 17%, p=0.014), livedo (36%vs 10%, p=0.003) and leukopenia (64% vs 39%, p=0.042), and the presence of SSA antibodies (26% vs 6%, p=0.009) and SSB antibodies (55% vs 5%, p<0.001) with dry eye syndrome. Patients with RNP antibodies were significantly more likely to have anemia (93% vs 58%, p=0.015), and patients with antibodies to nucleosomes had fever (75% vs 52%, p=0.033) and anemia (86% vs 54%, p=0.003). The presence of antihistone antibodies was associated with hypocomplementemia (85% vs 49%, p=0.027), anemia (85% vs 58%, p=0.023), the presence of antibodies to beta-2 glycoproteins with cutaneous vasculitis (33% vs 11%, p=0.011).

**Conclusion:** The evaluation of autoantibodies in children with SLE revealed associations with clinical and laboratory manifestations, which emphasizes the importance of their assessment in the diagnosis and follow-up of patients with SLE.

**Patient Consent**

Not applicable (there are no patient data)

**Disclosure of Interest**

None Declared

### Systemic lupus erythematosus and antiphospholipid syndrome

#### P418 Systemic lupus erythematosus in a monocentric pediatric population. need for a multi-disciplinary long-term follow-up database

##### Celine Boeykens ^1^, Katrien De Vusser^2^, Lien De Somer^3^, Carine Wouters^3^

###### ^1^KULeuven; ^2^Nephrology; ^3^Pediatric rheumatology, UZleuven, Leuven, Belgium

####### **Correspondence:** Celine Boeykens

*Pediatric Rheumatology 2024*, **22(2)**: PReS24-ABS-1065

**Introduction:** Childhood onset systemic lupus erythematosus (cSLE) is a chronic, multisystemic auto-immune disease with a wide range of clinical manifestations. As of today, there are no long-term follow-up studies nor studies describing the clinical characteristics and outcomes of Belgian cSLE patients.

**Objectives:** The aim of the present study was to describe the demographic, clinical, immunological and treatment characteristics in a Belgian cSLE cohort and to compare these results with 35 similar cohort studies in the literature.

**Methods:** We examined the medical records of patients who received the diagnosis of cSLE in the university hospital UZ Leuven between January 1^st^ 1999 and July 1^st^ 2023. Demographic, clinical, immunological and treatment data were extracted from our medical record database.

**Results:** A total of 33 children (26 girls), who met at least 4 of the ACR19 classification criteria, were included in the study. The mean age at diagnosis and average duration of follow-up were 11.9 and 8.3 years, respectively. 30.3% had a family history of auto-immune diseases. Leukopenia (69.7%) and arthritis/arthralgia (78.8%) were the most common presenting clinical manifestations. The frequency of lupus nephritis was remarkably low(33.3%). The frequency of the most common autoantibodies was as follows: anti-dsDNA (90.9%), anti-sm (46.9%), anti-U1-RNP (43.8%) and anti-SSA (25.0%). 9.1% developed an antiphospholipid syndrome. Mycophenolate mofetil was the most prescribed immunosuppressant (72.7%). The mean SLEDAI score at diagnosis was 16.1. In our cohort, as well as two other cohorts, an increase in the incidence of cSLE diagnoses over time was observed.

**Conclusion:** Over 23 years, our patients experienced a generally positive outcome: a flare rate of 1.7 per patient and a mortality rate of 0%. Nevertheless, the atypical presentation in 1/3 of the children and the persistent burden of treatment complications, such as weight gain and steroid myopathy, remain areas of significant concern. Moreover, the increasing incidence of cSLE diagnoses is remarkable.

**Trial registration identifying number:** /

**Date of birth::** 10.02.2000

**Patient Consent**

Not applicable (there are no patient data)

**Disclosure of Interest**

None Declared

**Reference**

1. Reference number for the Ethics Committee: MP026088 - PMID reference numbers of the 35 cohort studies: 1: 36371201, 2: 30957986, 3: 23806205, 4: 36226607, 5: 35134124, 6: 35037500, 7: 34479243, 8: 32631203, 9: 30648229, 10: 30071768, 11: 29915860, 12: 29690965, 13: 29233038, 14: 27771618, 15: 26223297, 16: 25926055, 17: 25758881, 18: 24505121, 19: 23966304, 20: 24435032, 21: 24003079, 22: 22294381, 23: 21294427, 24: 20659970, 25: 21199476, 26: 20734169, 27: 19301009, 28: 18539716, 29: 18346514, 30: 18344923, 31: 18092438, 32: 17668644, 33: 17402372, 34: 15940546, 35: 15479574

### Systemic lupus erythematosus and antiphospholipid syndrome

#### P419 The many faces of paediatric cns lupus and sjögren’s – a case series

##### Heba Mansour^1^, George Aldersley^2^, Phil Riley^2^, Dipak Ram^1^, Sajida Rasul^2^

###### ^1^Department of Paediatric Neurology; ^2^Department of Paediatric Rheumatology , Royal Manchester Children's Hospital, Manchester, United Kingdom

####### **Correspondence:** Heba Mansour

*Pediatric Rheumatology 2024*, **22(2)**: PReS24-ABS-1557

**Introduction:** Central nervous system (CNS) manifestations of juvenile-onset SLE (jSLE) and Sjögren’s are highly variable. Presentations include headache, seizures, movement disorders, and a variety of psychiatric manifestations from mood disorders to psychosis. There also exists a significant overlap with aquaporin-4 related neuromyelitis optica spectrum disorder. We describe four patients each with differing presentations, their approach to diagnosis and management, and their progress with treatment. In particular, we highlight the importance of multidisciplinary team working.

**Objectives:** To highlight the complexity in diagnosis and management of CNS jSLE and Sjögren’s through describing our experience in this case series.

**Results:** All patients met criteria for diagnosis after thorough multidisciplinary discussion and were initially treated with steroids and cyclophosphamide +/- rituximab.

Patient 1: presented age eleven with persistent vomiting, weight loss and progressive lower limb weakness. MR brain was suggestive of area postrema syndrome. Aquaporin 4-IgG was positive, as was dsDNA. She subsequently suffered cerebral venous sinus thrombosis. At six-year follow-up, she can walk for ten minutes without weakness.

Patient 2: presented age seven with hemiparesis and parotid swelling. MR brain revealed multiple old infarcts and an acute infarct in the right basal ganglia alongside optic neuritis. Aquaporin 4-IgG was positive, as were Sjögren-related antibodies. At seven-year follow up, she has no neurological deficit.

Patient 3: presented age eleven with intermittent right hemichorea. An initial MR brain showed foci of possible ischaemia or inflammation, but her symptoms had self-resolved. Two years subsequently, she developed intermittent brief hemisensory disturbance and vertigo. A repeat MR brain revealed scattered white matter hyperintensities. High ESR and positive ANA were highly indicative of CNS lupus. At three-month follow up post-initiation of treatment her symptoms have improved.

Patient 4: presented age fifteen with florid psychosis and catatonia having experienced a six-month prodrome of declining mood and behaviour. SSA-A and ANA were positive. Other organic causes were excluded. MR brain showed generalised atrophy. At three-month follow up post-initiation of treatment, her mental state has improved.

**Conclusion:** We demonstrate the varied CNS presentations and outcomes of jSLE and Sjögren’s. Each patient required a tailored approach to rehabilitation and close multidisciplinary team working. We recommend using ACR/Eular and SLICC criteria, with a consensus between rheumatology and neurology to make such diagnoses. We also recommend that all patients with jSLE and Sjögren’s are tested for aquaporin-4 antibodies given the prognostic implications.

**Patient Consent**

Yes, I received consent

**Disclosure of Interest**

None Declared

### Systemic lupus erythematosus and antiphospholipid syndrome

#### P420 Utility of pan-immune-inflammation value as a predictor of the prognosis of childhood lupus

##### Ali Alasmari^1^, Haifa Aldakhil^2^, Alhanouf Al-Saleem^1^, Sulaiman M. AlMayouf^1, 3^

###### ^1^Pediatric Rheumatology, ^2^Department of Biostatistics, Epidemiology and Scientific Computing, King Faisal Specialist Hospital and Research Centre, ^3^College of Medicine, Alfaisal University, Riyadh, Saudi Arabia

####### **Correspondence:** Ali Alasmari

*Pediatric Rheumatology 2024*, **22(2)**: PReS24-ABS-1569

**Introduction:** Systemic lupus erythematosus (SLE) is a chronic inflammatory multisystemic disease. Monitoring disease course and activity is important for effective management and outcome.

**Objectives:** To assess whether the pan-immune inflammation value (PIIV) at diagnosis could predict organ involvement and disease activity in childhood SLE (cSLE) patients.

**Methods:** This is an observational retrospective multicenter study that comprised cSLE patients seen and followed at the participating centers between January 2010 and December 2022. All patients met the EULAR/ACR-19 criteria, were immunosuppressive drug-naïve at the time of SLE diagnosis and had a minimal follow-up period of 12 months. The data included clinical and laboratory findings and disease activity using the SLEDAI. A receiver operating characteristic (ROC) curve was employed to determine the optimal cut-off value of PIIV and the predictive power of PIIV for disease activity, organ involvement, and treatment response.

**Results:** A total of 125 patients (104 female) with a median age of 16 (IQR 5.6) years, a median age at disease onset of 10.9 (IQR 3) years, and a median disease duration of 4.8 (IQR 5.3) years were included. The most frequent involved organs at diagnosis were hematological (89.6%), musculoskeletal (68.8%), mucocutaneous (63.2%), and renal (58.4%). However, at one year follow-up visit, the most frequent involved organs were renal (40.0%), hematological (39.2%), musculoskeletal (15.2%), and mucocutaneous (10.4%). The median PIIV at diagnosis was 139 (IQR 229.6), while the median SLEDAI was 12 (IQR 6.5) and 3.5 (IQR 7.0) at diagnosis and 12 months, respectively. An optimal cut-off of PIIV of 250 was found to be a predicative for disease activity, with a sensitivity of 45% and a specificity of 86%. The study revealed that the PIIV successfully predicted four systems in our cohort of patients.

**Conclusion:** Our work suggests the PIIV might be a reasonable predictor for organ involvement and disease activity in newly diagnosed cSLE, though further research, particularly larger studies, is required to validate these findings, especially regarding organ involvement.

**Patient Consent**

No, I have not received consent

**Disclosure of Interest**

None Declared

### Systemic lupus erythematosus and antiphospholipid syndrome

#### P421 Evaluation of general characteristics in juvenile- onset systemic lupus erythematosus patients: “a single center study”

##### Gülcan Özomay Baykal, Betül Sözeri

###### Pediatric Rheumatology, Umraniye Training and Research Hospital, Istanbul, Türkiye

####### **Correspondence:** Gülcan Özomay Baykal

*Pediatric Rheumatology 2024*, **22(2)**: PReS24-ABS-1679

**Introduction:** Juvenile-onset systemic lupus erythematosus (jSLE), while uncommon, represents a critical multisystem autoimmune/inflammatory condition that holds the potential to target any organ, leading to severe impairment, disability, or even mortality. This disease is characterized by its emergence before the individual reaches 18 years old, and it accounts for about 15–20% of all systemic lupus erythematosus (SLE) cases.

**Objectives:** The aim of this study is to define the sociodemographic, clinical and laboratory aspects of the jSLE patients.

**Methods:** Data from consecutive jSLE patients treated at Ümraniye Training and Research Hospital’s pediatric rheumatology unit were retrospectively collected. The investigation spanned from January 2017 to February 2024 and focused on patients diagnosed with the criteria: 1. fulfilling at least four of the American College of Rheumatology (ACR) criteria for SLE classification; 2. being under 18 years of age at the onset of the disease.

**Results:** The study ultimately included 69 patients diagnosed with jSLE; fifty-five were female and fourteen were male. The average age at the time of diagnosis was 13.05 years, spanning from 2 to 17.5 years. The average duration of follow-up for these patients was 3.5 years, with a range from 3 to 7 years. Clinically, at the initial consultation, musculoskeletal symptoms, arthralgia, were the most frequently reported. The predominant clinical signs included renal disease, malar rash, and hematological issues. A significant portion of the patient group exhibited critical organ involvement, either through renal (37 patients) or hematological issues (36 patients); notably, 23 patients (33%) presented with complications in both renal and hematological domains. In total, fifty-three patients (76.8%) showed involvement of at least one major organ system, be it CNS, renal, or hematological. The discovery of a strong link between renal involvement and SLE Disease Activity Index (SLEDAI) scores at both the time of diagnosis (p=0) and during the most recent visit (p= 0.0038) indicates a distinct difference in SLEDAI-2K scores among lupus patients with and without renal complications.

**Conclusion:** This investigation reveals a favorable disease trajectory for jSLE in patients, marked by lower baseline disease activity, damage scores, and less frequent kidney and CNS disease.

**Date of birth::** novembre 2

**Patient Consent**

Not applicable (there are no patient data)

**Disclosure of Interest**

None Declared

**References**

1. Brunner HI, Gladman DD, Ibañez D, Urowitz MD, Silverman ED. Difference in disease features between childhood-onset and adultonset systemic lupus erythematosus. Arthritis Rheum. 2008;58(2):556-62. 10.1002/art.23204
2. Hedrich CM, Smith EMD, Beresford MW. Juvenile-onset systemic lupus erythematosus (jSLE) - pathophysiological concepts and treatment options. Best Pract Res Clin Rheumatol. 2017;31(4):488-504. 10.1016/j.berh.2018.02.001
3. Smith EMD, Lythgoe H, Hedrich CM. Vasculitis in juvenile-onset systemic lupus erythematosus. Front Pediatr. 2019;7:149. 10.3389/fped.2019.00149
4. Smith EMD, Lythgoe H, Midgley A, Beresford MW, Hedrich CM. Juvenile-onset systemic lupus erythematosus: update on clinical presentation, pathophysiology and treatment options. Clin Immunol. 2019;209:108274. 10.1016/j.clim.2019.108274
5. Tsokos GC. Systemic lupus erythematosus. N Engl J Med. 2011;365(22):2110-21. 10.1056/NEJMra1100359

### Systemic lupus erythematosus and antiphospholipid syndrome

#### P422 A complete heart block in infant lupus erythematosus, : a case report

##### Awatif A. Abushhaiwia^1^, Abdelmoneim A. Benarous ^2^

###### ^1^Paediatric Rheumatology , Faculty of Medicine , university of Tripoli, Tripoli Children's Hospital; ^2^Cardiology department , Tripoli Children's Hospital , Tripoli , Libya

####### **Correspondence:** Awatif A. Abushhaiwia

*Pediatric Rheumatology 2024*, **22(2)**: PReS24-ABS-1024

**Introduction:** Neonatal lupus erythematosus (NLE) is a rare acquired autoimmune disease of newborns caused by the transfer of maternal antibodies against autoantigen type A (Ro/SSA) or B (La/SSB) to the fetus.The irreversible and complete heart block (CHB) due to NLE causes mortality in 15%–30% and two‐thirds of them require permanent pacing. Here. We report a rare case of infant with complete CHB with high maternal Ro/SSA and La/SSB titre with unfavourable outcome.

**Objectives:** Highlights the challenges of diagnosing and managing CHBs in low resource settings.

**Methods:** a case report

**Results:** A 4-month-old infant female was referred to our centre and was immediately admitted to paediatric PICU because of cardiogenic shock with severe bradycardia with HR 32 b\m. She was born to a 24-year primigravida mother at 37 weeks of gestation. Delivered by emergency (LSCS) with birth weight 3.7kgm because the mother was on regular antenatal checkups and had a history of joint pain, fatigability &thrombocytopenia. Although the mother had a high index of suspicion of SLE at that time, they didn’t investigate her about it. She was admitted to NICU due to severe bradycardia, poor peripheral perfusion& cyanosis. chestx-ray showed marked cardiomegaly&left lung collapse.ECG showed complete heart block, a heart rate of 32 beats/min and findings suggestive of complete heart block. Her echocardiography showed 5 mm ostium secundum atrial septum defect (OS‐ASD) 7 mm, small PDA, mild LV hypertrophy, dilated ascending aorta, mild MR, mild TR & severe pericardial effusion, dissecting aortic arch. she was critical ill, pale, cyanotic, and dehydration. Her heart rate was 32 bpm, absent peripheral pulses, her temperature was 35 c^0^, BP 50\30, weighed 4.5kg and she had respiratory rate 40 b\m) .Blood pressure was 50/30 mm of Hg. other systems were non‐ significant. She was connected to mechanical ventilation, started on dobutamine IV, methypredinsolne 25mg IV twice\day, furosemide 2mg\kg\day IV, aldactone tab by NGT once per day. Cardiac neonatal lupus was confirmed with positive maternal anti-Ro/SSA was >100 U\ml and anti-La/SSB was 69U\ ml, and the baby test of anti-Ro/SSA was positive with high titter 54.50 U\ml, anti-La/SSB was 34.7 U\ml. The rest of the investigations of the baby (CBC, CRP, ESR, TORCH, blood culture, TSH, free T4, serology test for COVID-19) were within normal limits. Therefore. she was an indication of a pacemaker implantation, but unfortunately, she died before that.

**Conclusion:** CHB may be associated with a high morbidity and mortality. In this case, infant presented with a complete heart block and dissecting aortic arch , which required a pacemaker for treatment. But unfortunately she died before inserted the pacemaker

**Patient Consent**

Yes, I received consent

**Disclosure of Interest**

None Declared

**Reference**

1. Sara De Carolis^1^, Cristina Garufi^2^, Ester Garufi^3^_,_ Autoimmune Congenital Heart Block: A Review of Biomarkers and Management of Pregnancy, Frontiers in Pediatrics , 1 Volume 8 | Article 607515, December 2020

### Systemic lupus erythematosus and antiphospholipid syndrome

#### P423 Monogenic systemic lupus erythematosus in combination with primary immunodeficiency - features of the disease and therapeutic tactics

##### Maria Shalygina ^1^, Nina Nikishina^1^, Tatiana Popkova^1^, Irina Nikishina^1^, Maria Kaleda^1^, Svetlana Rodionovskaya^2^

###### ^1^V.A.Nasonova Research Institute of Rheumatology; ^2^Federal Research and Clinical Center for Children and Adolescents, FMBA of Russia, Moscow, Russian Federation

####### **Correspondence:** Irina Nikishina

*Pediatric Rheumatology 2024*, **22(2)**: PReS24-ABS-1113

**Introduction:** Monogenic lupus is a form of systemic lupus erythematosus (SLE) that occurs in patients with a single gene defect. Type I interferon (IFN) is a important pathogenic factor in SLE, including in some monogenic forms. This fact allows us to classify monogenic lupus as type 1 interferonopathy. Monogenic lupus is often combined with primary immunodeficiency (PID)

**Objectives:** to present a case juvenile(j) SLE with PID

**Methods:** Case report

**Results:** Patient 20 y.o. with jSLE. Debut at 6 y.o with arthritis, fever, erythematous rash, ↑ESR. jArthritis was diagnosed, therapy - sulfasalazine, cyclosporineA (CsA),sequentially. 4 y. after the debut, thrombotic microangiopathy developed: PLT 26 10^9^/l, WBC 2,8 10^9^/k, Hb 76g/l, gastrointestinal hemorrhage, hemorrhagic rash, proteinuria. Therapy - plasma, thromboconcentrate, hemotransfusions, systemic glucocorticoids(GC) with a positive effect. At the age of 11, photosensitization, ANA(Hep-2) 1/10240(n<1/160), anti dsDNA>200 IU/ml(n 0-20), diagnosed SLE. Azathioprine(AZA), hydroxychloroquine 200mg/day, GC max dose 20mg/day are prescribed. Over the next 7 years exacerbations against the background of viral infections, hormone resistance, delayed physical development(height 135cm, weight 39kg), high activity of inflammation (CRP,ESR), autoimmunity (↑anti dsDNA,Sm/RNP). At the age of 17 y.o she started biologics therapy – belimumab(BEL) with positive results during 2 y. After, she'd flare, associated with herpes: mucocutaneous involvement, thrombovasculitis, arthritis, АNA (Hep-2) 1/5120, anti dsDNA 612 IU/ml(n0-100), anti RNP-70 175U/ml (n0-25), anti C1q 51U/ml (n0-10), ↓С3,С4. SELENA-SLEDAI-18. BEL, AZA discontinued, she started Rituximab(RTM), mycophenolate mofetil(MMF) with positive effect - SELENA-SLEDAI after 12mo. of therapy – 6, RTM summary 1.5g, however depletion of CD19+ wasn't completed. According to atypical course of disease we investigated the interferon profile: CD169+ 1209.6 (range <7.0). A complete sequencing of the exome was performed at the age of 20 y.o.: a mutation of p.Gln426Ter, previously not described in the literature, was detected in a heterozygous state in a gene SYK, associated with immunodeficiency in combination with systemic inflammation

**Conclusion:** Features of the disease: debut, delayed physical development, recurrence of SLE against the background of infections, high laboratory and immunological activity of inflammation, insufficient response to therapy allowed discussing the diagnosis of monogenic SLE in combination with autoinflammatory diseases from the group of interferonopathies and PID. The mutation in the SYK gene explains the atypical course of the disease with recurrent inflammation against the background of viral infections. Therapy remain an open question, the expediency of prescribing an IFN type 1 inhibitor or JAK kinase blockers in case of insufficient RTM effect is being discussed.

**Date of birth::** 24.01.1997

**Patient Consent**

Yes, I received consent

**Disclosure of Interest**

None Declared

### Systemic lupus erythematosus and antiphospholipid syndrome

#### P424 Trex1 as the cause of monogenic systemic lupus erythematosus in a 10-year-old girl: case report

##### Leosirlay Rojas-Gomez^1, 2^, Adriana Díaz-Maldonado ^1, 2, 3, 4^, Maria Fernanda Reina-Ávila^1, 2, 3^

###### ^1^Pediatric Rheumatology Program, Universidad El Bosque, School of Medicine, ^2^Member of the Colombian Association of Rheumatology, ^3^Pediatric Rheumatology, HOMI, Fundación Hospital Pediatrico La Misericordia, ^4^CARE FOR KIDS, Bogotá, Colombia

####### **Correspondence:** Leosirlay Rojas-Gomez

*Pediatric Rheumatology 2024*, **22(2)**: PReS24-ABS-1266

**Introduction:** Childhood-onset systemic lupus erythematosus (cSLE) is a chronic, autoimmune, multisystem and potentially fatal disease that occurs in children under 18 years of age. From the pathophysiological perspective, early presentation can lead to a stronger genetic relationship and in fact in recent years different genetic variants have been identified that can cause phenotypes similar to SLE and these are known as monogenic SLE. The three prime repair exonuclease 1 (TREX1) has been one of those most frequently associated with the onset of the disease and is estimated to be around 2% of patients with SLE.

**Objectives:** Here we report the case of a 10-year-old patient with a diagnosis of cSLE but with atypical clinical presentation, severe manifestations of the disease and with failure to the usual management therapy, so it was suggested that her autoimmunity was associated with some immune disorder. Sequencing of a panel of genes related to autoinflammation and immunodeficiencies was performed, with a report of a variant in the TREX1 gene c.590C>T,

p.Ala197Val, in heterozygosity, of uncertain significance based on the American College classification of Medical Genetics and genomic criteria, but compatible with the patient's clinical phenotype, so monogenic SLE was diagnosed due to a variant in TREX1.

**Conclusion:** Monogenic SLE is a multisystemic and highly severe pathology that usually manifests itself atypically when compared to classic SLE; TREX1 variants represent up to 2% of all SLE in the general population, so it is necessary to think about those children with the onset of the disease before the age of 5 years, in whom the form of presentation is atypical, severe and there is no response to conventional treatment, and in this way be able to establish a timely molecular diagnosis to carry out targeted therapy and thus avoid progression of organ damage derived from the pathology.

**Patient Consent**

Yes, I received consent

**Disclosure of Interest**

None Declared

**References**

1. Omarjee O, Picard C, Frachette C, Moreews M, Rieux-Laucat F, Soulas-Sprauel P, et al. Monogenic lupus: Dissecting heterogeneity. Vol. 18, Autoimmunity Reviews. Elsevier B.V.; 2019.
2. Charras A, Smith & E, Hedrich CM. Systemic Lupus Erythematosus in Children and Young People. Available from: 10.1007/s11926-021-00985-0
3. Lythgoe H, LJ M, Hedrich CM, Aringer M. Classification of systemic lupus erythematosus in children and adults. Clinical Immunology. 2022 Jan 1;234.

### Systemic lupus erythematosus and antiphospholipid syndrome

#### P425 Drug induced lupus by ruxolitinib in a patient with graft versus host disease myositis

##### Elli Athanasopoulou^1^, Eleni D. Ioannidou^2^, Christina Economopoulou^2^, Kyveli Chiotopoulou^1^, Katerina Kourtesi^3^, Evgenios Goussetis^2^, Lampros Fotis^3^

###### ^1^Department of Pediatrics, ATTIKON General Hospital, National and Kapodistrian University of Athens; ^2^ BMT Unit, Aghia Sofia Children’s Hospital; ^3^Department of Pediatrics, Division of Pediatric Rheumatology, Attikon General Hospital, National and Kapodistrian University of Athens, Athens, Greece

####### **Correspondence:** Lampros Fotis

*Pediatric Rheumatology 2024*, **22(2)**: PReS24-ABS-1270

**Introduction:** Drug-induced lupus (DIL) is a rare autoimmune phenomenon. Here, we report the case of an adolescent with a history of acute myeloid leukemia (AML) who developed myositis following allogeneic bone marrow transplantation (BMT) and subsequently experienced DIL due to ruxolitinib therapy.

**Objectives:** To elucidate the diagnostic challenges and therapeutic implications of DIL in a pediatric patient with post-BMT following ruxolitinib administration.

**Methods:** A comprehensive review of the patient's medical records, clinical presentations, imaging studies, treatment regimens, disease course, and autoimmune serologies, was conducted. Pertinent literature regarding drug-induced lupus and ruxolitinib-related adverse effects in pediatric BMT recipients was also reviewed.

**Results:** A 15.5-year-old female, previously diagnosed with AML at age 4, underwent allogeneic BMT followed by immunosuppressive therapy. Six years post-BMT, she presented with symptoms suggestive of myositis, including fatigue, muscle pain, diplopia, eyelid droop, and blurred vision. Initially, her treatment regimen consisted of corticosteroids, intravenous immunoglobulin, mycophenolate mofetil (MMF), and rituximab. After a drug-free period of three months, she experienced a relapse, prompting initiation of ruxolitinib at a dose of 10 mg twice daily, leading to the cessation of corticosteroids and achieving clinical stability.

However, after 20 months of ruxolitinib treatment, she developed leucopenia, Coombs-positive anemia, thrombocytopenia, deep vein thrombosis, arthritis, and elevated inflammatory markers. Immunological laboratory testing revealed elevated levels of ANA (>1:2560), pANCA (1:1280), anti-histone, anti-dsDNA, anti-CCP, and anti-cardiolipin antibodies (IgG & IgM). The diagnosis of DIL secondary to ruxolitinib was confirmed. Consequently, ruxolitinib was discontinued, and treatment with prednisone, hydroxychloroquine, and rituximab was initiated.

Three months after completing the second cycle of rituximab, while on a low dose of prednisone, she experienced a relapse of myositis. Tacrolimus at a dose of 0.5mg twice daily was commenced, allowing complete cessation of prednisone, and she has since remained stable on tacrolimus monotherapy.

**Conclusion:** As ruxolitinib has not previously been reported as a drug that induces lupus, this case represents the first such report in the literature. The association of ruxolitinib with autoimmune dysregulation necessitates careful monitoring of autoimmune markers and management of potential complications. In addition to autoimmune concerns, ruxolitinib has been linked to other adverse events, including anemia, neutropenia, thrombocytopenia, infections (such as HZV reactivation), and neoplasms. These diverse adverse events underscore the importance of comprehensive monitoring and personalized management strategies tailored to each patient.

**Patient Consent**

Yes, I received consent

**Disclosure of Interest**

None Declared

**References**

1. Fan S, Huo WX, Yang Y et al. Efficacy and safety of ruxolitinib in steroid-refractory graft-versus-host disease: A meta-analysis. *Front Immunol*. 2022;13:954268.
2. Hoisnard L, Lebrun-Vignes B, Maury S, et al. Adverse events associated with JAK inhibitors in 126,815 reports from the WHO pharmacovigilance database. *Sci Rep*. 2022;12(1):7140.

### Systemic lupus erythematosus and antiphospholipid syndrome

#### P426 Clinical, laboratory and immunological features in patients with Juvenile -onset systemic lupus erythematosus

##### Adisa Čengić^1^, Velma Selmanović^1^, Danka M. Pokrajac^2^, Delfina Skender^3^, Ahmed Mulač^1^

###### ^1^Allergology, Rheumatology And Clinical Immunology; ^2^Nephrology, Pediatric clinic,Clinical Center University of Sarajevo, Sarajevo, ^3^Rheumatology and Immunology, Pediatric Clinic, University Clinical Hospital , Mostar, Bosnia and Herzegovina

####### **Correspondence:** Adisa Čengić

*Pediatric Rheumatology 2024*, **22(2)**: PReS24-ABS-1344

**Introduction:** Juvenile-onset systemic lupus erythematosus (jSLE) also known as pediatric lupus (pSLE) or childhood SLE (cSLE) is a rare chronic autoimmune disease with multisystemic involvement that can cause significant damage, disability and death. jSLE fulfils the definition of a rare disease in Europe and has a more severe disease presentation than lupus in adults, with a higher incidence of major organ involvement, more aggressive disease course and higher medication burden.

**Objectives:** To determine the presenting clinical and laboratory characteristics of patients diagnosed with jSLE in the last 18 years in our department.

**Methods:** Medical records of 18 jSLE patients admitted toour department from July 2007 to March 2024 were retrospectively reviewed. Five patients fulfilled the 1997 ACR classification criteria and 13 patients fulfilled 2012 SLICC classification criteria. We followed at disease presentation: constitutional symptoms (fatigue, fever, arthralgia, myalgia and weight changes), skin and mucosal changes,arthritis, renal and CNS disease, macrophage activation syndrome, secondary antiphospholipid syndrome. Laboratory:CRP,ESR,WBC, platelets count,DCT,ANA,anti-DsDNA,antiSm, antiphospholipid antibodies, C3, C4.

**Results:** Eighteen children (15 girls and 3 boys) were diagnosed as having jSLE. Mean age at disease presentation was 12 years. Youngest child was two years old and later, he was diagnosed as having monogenic SLE ( DNASE1L3). Arthralgia was the most common complaint at presentation (72%), followed by fever (44%). 22% of patients had both fever and arthralgia. The most common clinical manifestation of lupus were: synovitis ( 67%) folowed by renal disease (66%), leukopenia (50%), oral ulcers and thrombocytopenia( 38%), malar rash (33%), serositis ( 22%), neurologic simptoms(16%).The renal biopsy was performed in 13 cases; 4 patients (22%) had stage 2 nephritis, one had stage 3 and 3 (16%) had stage IV nephritis. Two patients had normal pathohistological findings on kidney biopsy. Sedimentation rate was increased in all children as well as ANA. Anti-dsDNA were significantly elevated in16(88%), anti-Smith in 9 (50%), antiphospholipid antibodies in 12 (66%). Low C3 was observed in 14 (77%) and C4 in 15 (83%) patients.

**Conclusion:** Juvenile –onset systemic lupus is a challenging disease, in terms of diagnosis and treatment. Disease caries greater aggressiveness compared with lupus diagnosed during adulthood. All cases of fever of unknown origin, especially if concomitant with joint pain, should undergo evaluation for jSLE, as the most common presenting symptom in our cohort were fever, arthritis and arthralgia.

**Patient Consent**

Not applicable (there are no patient data)

**Disclosure of Interest**

None Declared

**Reference**

1. Charras A, Smith E, Hedrich CM. Systemic Lupus Erythematosus in Children and Young People. Curr Rheumatol Rep. 2021;23(3):20.

### Systemic lupus erythematosus and antiphospholipid syndrome

#### P427 Management of childhood-onset systemic lupus erythematosus over the last two decades in Spain

##### Alina Boteanu ^1^, Juan J. Bethencourt^2^, Joan Calzada-Hernández^3^, Daniel Clemente^4^, Juan C. Nieto-González^5^, Laura Luque^6^, Covadonga Lopez^6^, Inmaculada Calvo^7^

###### ^1^Pediatric Rheumatology Department, Ramón y Cajal University Hospital, Madrid; ^2^Pediatric Rheumatology Department, Canarias University Hospital, Santa Cruz de Tenerife; ^3^Pediatric Rheumatology Department, Hospital Sant Joan de Déu, Barcelona; ^4^Pediatric Rheumatology Department, Niño Jesús Children University Hospital; ^5^Rheumatology Department, Gregorio Marañón General University Hospital; ^6^Medical Department, GSK, Madrid; ^7^Pediatric Rheumatology Department, La Fe University and Politecnic Hospital, Valencia, Spain

####### **Correspondence:** Alina Boteanu

*Pediatric Rheumatology 2024*, **22(2)**: PReS24-ABS-1375

**Introduction:** Childhood-onset systemic lupus erythematosus (cSLE) is a more severe disease than adult-onset SLE (1), but its management is mostly based on studies in adults, with little evidence in children.

**Objectives:** Explore the evolution of cSLE management in Spain over the last 20 years, considering recent recommendations and studies.

**Methods:** A review of cSLE publications by Spanish authors was performed.

**Results:** cSLE management in Spain has followed adult and cSLE recommendations during the last two decades. In the early 2000s, the 1997 American College of Rheumatology (ACR) classification criteria were the most accepted. Hydroxychloroquine (HCQ) and glucocorticoids (GCs) were the cornerstone of treatment but did not control disease activity and achieve remission in all patients (2).

Over the following decades, there was an increasing concern about GC’s long-term effects such as the risk of infections and other comorbidities. By then, international experts recommended the general use of HCQ and GC-sparing agents such as immunosuppressants (IS) (3,4). Additionally, new classification criteria for SLE had been published by European League Against Rheumatism/ACR (2019), which have been applied also in cSLE (5).

Currently, there is no consensus document for the management of cSLE in Spain other than book chapters focused on cSLE by the Spanish Society of Rheumatology (*Manual*, 2019) and by the Spanish Society of Paediatric Rheumatology (*Protocol*, 2020) (6,7). Both highlight the lack of cSLE classification criteria developed for children and establish remission, organ damage reduction and improvement of survival as the main treatment goals, thus recommending early initiation of GC-sparing agents such as conventional IS or belimumab (BEL).

Recent advances in cSLE management include the application of the treat-to-target (T2T) approach (8) and the approval of intravenous BEL for patients ≥5 years, as a result of the PLUTO study (9), while the first results with subcutaneous BEL in cSLE were promising (10). However, cSLE management remains a challenge due to the lack of evidence and guidelines.

**Conclusion:** Substantial changes have occurred in cSLE management, with the principles of T2T contributing to this evolution, whilst the use of HCQ and GC-sparing strategies such as IS and BEL has increased in clinical practice. However, evidence is still limited, highlighting the need for new studies, real-world evidence and consensus documents.

**Patient Consent**

Not applicable (there are no patient data)

**Funding**

GSK

**Disclosure of Interest**

A. Boteanu Speaker Bureau with: Novartis, GSK, J. Bethencourt Speaker Bureau with: GSK, Abbvie, J. Calzada-Hernández Consultant with: Alexion, Speaker Bureau with: Alexion, Pfizer and Gebro, D. Clemente Grant / Research Support with: AbbVie and Pfizer, Speaker Bureau with: Novartis, AbbVie, Roche, Pfizer, GSK and Sobi, J. Nieto-González Grant / Research Support with: Nordic Pharma, Consultant with: Galápagos, Abbvie, GSK, Janssen and MSD, Speaker Bureau with: MSD, Pfizer, Abbvie, UCB pharma, Janssen, Lilly, Roche, Nordic Pharma, Gebro, Novartis, Biogen, Amgen and Sandoz., L. Luque Employee with: GSK, C. Lopez Employee with: GSK, I. Calvo Consultant with: Mereo Byopharma, Amgen, Novartis, Sobi, GSK, Abbvie, Speaker Bureau with: Pfizer, Abbvie, Novartis, Sobi, Mereo Byopharma, GSK

**References**

1. Torrente V. Clin Exp Rheumatol. 2017 Nov-Dec;35(6):1047-1055.
2. Stichweh D. An Pediatr (Barc). 2005 Oct;63(4):321-9.
3. Hollander MC. Arthritis Care Res (Hoboken). 2013 Sep;65(9):1416-23.
4. Groot N. Ann Rheum Dis. 2017;76(11):1788-1796.
5. Levinsky Y. Rheumatology (Oxford). 2021;60(11):5142-5148.
6. López-Robledillo JC. Madrid: Ergon; 2019. 107-116
7. Boteanu A. Protoc diagn ter pediatr. 2020;2:115-128.
8. Brunner HI. Ann Rheum Dis. 2020;79(10):1340-1348.
9. Smith EMD. Ann Rheum Dis. 2023;2(6):788-798.
10. Brunner H. Arthritis Rheumatol. 2023;75(suppl 9)

### Systemic lupus erythematosus and antiphospholipid syndrome

#### P428 Emerging systemic complications in pediatric subacute cutaneous lupus: a case of unexpected gastrointestinal vasculitis

##### Brenda De Jesus Fortuna^1^, Jorge Armando G. Chapa^2^

###### ^1^Pediatric Rheumatology, UANL, Monterrey, Mexico; ^2^Rheumatology, University of Washington, Seattle, United States

####### **Correspondence:** Brenda De Jesus Fortuna

*Pediatric Rheumatology 2024*, **22(2)**: PReS24-ABS-1222

**Introduction:** Subacute Cutaneous Lupus Erythematosus (SCLE) in children is an uncommon condition usually characterized by distinct morphological rashes and minimal systemic involvement. However, arthralgia or arthritis can occur in 50-70% of cases.

**Objectives:** This study aims to document and analyze the clinical manifestations, diagnosis, and treatment of gastrointestinal vasculitis in a child diagnosed with SCLE.

**Methods:** This case study reviews the clinical presentation, diagnostic approach, and therapeutic interventions for a 9-year-old patient who developed gastrointestinal vasculitis following a diagnosis of SCLE.

**Results:** A 9-year-old child initially presented in 2022 with a well-defined erythematous annular plaque without scaling on the right maxillary region extending to the ear. Laboratory findings showed an ANA titer of 1:160 with a centriole pattern and positive anti-Ro. There were no systemic symptoms initially. Treatment with hydroxychloroquine and topical steroids was initiated, leading to an excellent clinical response. In January 2024, the patient developed moderate epigastric pain and hematemesis. An endoscopy revealed chronic nonspecific duodenitis, and chronic lymphoid colitis. Histopathology confirmed vasculitis in the esophagus, stomach, duodenum, and large intestine. Consequently, treatment with intravenous methylprednisolone boluses followed by monthly cyclophosphamide starting in April 2024. This case underscores the rarity and complexity of SCLE in pediatric patients, particularly when progressing to severe systemic manifestations like gastrointestinal vasculitis. While SCLE is predominantly noted for its skin manifestations, systemic complications can occur, albeit rarely. Anti-Ro antibodies, strongly associated with subacute cutaneous forms, were present in our patient, reinforcing the diagnosis of SCLE. It is important to note that approximately 15% of patients with SCLE are known to progress to systemic lupus. The therapeutic response to topical and systemic corticosteroids, along with hydroxychloroquine, was favorable in our patient. However, the emergence of gastrointestinal vasculitis as a complication signifies a severe progression of the disease. Gastrointestinal vasculitis is among the most devastating complications of systemic lupus erythematosus (SLE), with a prevalence of 0.2% to 9.7% among all SLE patients and and a mortality rate as high as 50%. The management strategy emphasizes the need for aggressive treatment and close monitoring, reflecting the high stakes involved in controlling the disease's progression in pediatric patients.

**Conclusion:** Subacute cutaneous lupus erythematosus is exceedingly rare in children. The presentation of gastrointestinal vasculitis, which is extremely rare. Continuous monitoring of this patient will be carried out in conjunction with pediatric rheumatology to manage any recurrence or evolution of the disease.

**Patient Consent**

Yes, I received consent

**Disclosure of Interest**

None Declared

**References**

1. 10.1111/pde.12007
2. 10.1186/1546-0096-13-1
3. 10.1016/j.jtauto.2021.100106
4. 10.1016/j.abd.2022.09.005

### Systemic lupus erythematosus and antiphospholipid syndrome

#### P429 Efficacy of abatacept in systemic lupus erythematosus arthritis: a case report

##### Maria Francesca Gicchino, Alma N. Olivieri

###### University of the Study of Campania L.Vanvitelli, Naples, Italy

####### **Correspondence:** Maria Francesca Gicchino

*Pediatric Rheumatology 2024*, **22(2)**: PReS24-ABS-1324

**Introduction:** Arthritis in systemic lupus erythematosus(SLE)is episodic and self-limited in most patients. However,in some cases, refractory joint problems may be poorly controlled byNSAIDs and other treatments.

In SLE patients with isolated, intermittent joint symptoms, short courses of NSAIDs should be used as the first-line treatment. If joint symptoms are more severe, a combination of low-dose corticosteroids(≤10mg/day) and antimalarial drugs is recommended. If joint symptoms persist, treatment indications depend on the other organs affected. In joint forms that are refractory to treatment or corticodependent, methotrexate should be proposed initially, in combination with antimalarial drugs. In cases of treatment failure or intolerance, mycophenolate mofetil or even azathioprine may be considered as an alternative treatment. As a last resort, after having weighed up the individual benefit-risk ratio, leflunomide, belimumab, rituximab or abatacept may be considered.

**Objectives:** To describe a case of refractory arthritis in a young patient with sistemic lupus erythematous successfully treated with Abatacept.

**Methods:** A 14-year-old girl was admitted to our Department for arthritis and fever since three months.

On admission to our department, the patient had a low grade fever, lymphadenopathy in the neck and bilateral inguinal areas, and markedly swollen and slightly tender fingers and wrists, elbows, ankles. Blood tests revealed anemia, (hemoglobin 10.2 g/dL ), acute inflammatory response (CRP 3 mg/dL, erythrocyte sedimentation rate [ESR] 48 mm/h), hypergammaglobulinemia (IgG 2,395 mg/dL), decreased levels of complements (C3 60 mg/dL, C4 8 mg/dL), and the presence of autoantibodies (ANA x320 speckled pattern, IgG anti-DNA antibody 15 U/mL). Antibodies to extractable nuclear antigens such as Sm, U1- RNP, Ro/SS-A, and La/SS-B were all negative. Echocardiogram showed a small amount of pericardial effusion.

**Results:** According to 2019 EULAR/PRES classification criteria Systemic lupus erythematous was diagnosed. Was started treatment with methylprednisoloen 1 gram per day for three consecutive days then prednisone, 50 mg per day and mycophenolate mofetil 2000 mg per day. Thanks to treatment patient clinical conditions initially improved.

Because of a gradual worsening of arthritis during steroids decalage, methotrexate 15 mg subcoutaneously once a week was added to treatment. After six months due to insufficient effectiveness, methotrexate was replaced by abatacept 500mg intravenously once per month. Thanks to this treatment joint swelling markedly improved. Corticosteroid dose was reduced successfully to 10mg/day after three months of abatacept treatment

**Conclusion:** In SLE patients with sever arthritis, refractory to treatment with methotrexate or immunosuppressant drugs and/or corticodependent, Abatacept should be considered.

**Patient Consent**

Yes, I received consent

**Disclosure of Interest**

None Declared

### Systemic lupus erythematosus and antiphospholipid syndrome

#### P430 Protein kinase cδ deficiency as a rising contributor to fatal monogenic lupus

##### Sulaiman M Al Mayouf^1, 2^, Safiya Al Abrawi^3^, Mohammed Assiri ^1^, Amani Al Ghadani^3^, Alhanouf Al-Saleem^1^

###### ^1^Pediatric Rheumatology, King Faisal Specialist Hospital & Research Center; ^2^College of Medicine, Alfaisal University, Riyadh, Saudi Arabia; ^3^Department of Child Health, Pediatric Rheumatology Unit, Royal Hospital, Muscat, Oman

####### **Correspondence:** Mohammed Assiri

*Pediatric Rheumatology 2024*, **22(2)**: PReS24-ABS-1564

**Introduction:** Monogenic lupus, a rare form of the disease, arises from single gene variants, dictating its development. Patients exhibit diverse clinical symptoms due to multi-organ involvement. Cerain genetic variants are well described as contributors while others are not rarely reported.

**Objectives:** To describe the phenotypic, genetic, and outcome characteristics of patients with monogenic lupus and Protein Kinase Cδ Deficiency (PRKCD).

**Methods:** The medical records of all children with monogenic lupus and PRKCD followed at two institutions (Saudi Arabia and Oman) were retrospectively reviewed for demographic, clinical, and genetic data, and outcomes at the last follow-up visit.

**Results:** Six (three males) children from two unrelated consanguineous Arab families were identified. Three of them had proven genetic variants. All patients presented before the age of 3-years with fever and multi-organ involvement, including cutaneous, musculoskeletal, cardiopulmonary, neurological, and renal features. Also, all had recurrent infection, two of them had BCGitis. All patients had high ANA, ds-DNA, and APL, with variable other autoantibodies positivity. They also, had low C_3_/C_4_, low C1q and CH_50_ (four patients). All patients were treated with corticosteroids and required sequential immunosuppressive drugs. Five patients received biologic agents. Three patients maintained a low disease activity on intensive treatment while three patients died due to severe disease and serious infections.

**Conclusion:** Our findings support the genetic evidence of PRKCD association with autosomal-recessive monogenic lupus. Furthermore, PRKCD may contribute to fatal disease.

**Patient Consent**

Yes, I received consent

**Disclosure of Interest**

None Declared

### Systemic lupus erythematosus and antiphospholipid syndrome

#### P431 A monogenic lupus family with multiple members of c1q deficiency

##### Aysima Atilgan Lulecioglu^1^, Sezgin Sahin^2^, Esma Aslan^2^, Semih Gulle^3^, Esra Firat Senturk^4^, Serdal Ugurlu^4^, Serkan Belkaya^1^, Ozgur Kasapcopur^2^

###### ^1^Department of Molecular Biology and Genetics, Bilkent University, Ankara; ^2^Pediatric Rheumatology, Istanbul University-Cerrahpasa, Cerrahpasa Medical Faculty, Istanbul; ^3^Division of Rheumatology, Department of Internal Medicine, Batman Training and Research Hospital, Batman; ^4^Division of Rheumatology, Department of Internal Medicine, Istanbul University-Cerrahpasa, Cerrahpasa Medical Faculty, Istanbul, Türkiye

####### **Correspondence:** Esma Aslan

*Pediatric Rheumatology 2024*, **22(2)**: PReS24-ABS-1789

**Introduction:** Systemic lupus erythematosus (SLE) is an autoimmune multifactorial disease. Patients with a family member with SLE and those with an early-onset disease should be genetically assessed for a Mendelian inheritance pattern[1-2].

**Objectives: Case 1:** A 4-year-old girl presented with resistant fever, alopecia, malar and discoid rash, and arthritis. ANA, anti-Smith, and anti-SSA were positive, anti-dsDNA was negative, and complement C3-C4 was normal. The patient was diagnosed with early-onset SLE and received pulse methylprednisolone (MPZ), hydroxychloroquine (HCQ), mycophenolate mofetil (MMF), and methotrexate (MTX). There are 4 adult patients diagnosed with SLE in the patient's family. Therefore, whole exome sequencing (WES) was conducted. A mutation in the C1q gene was detected in 5 individuals.

**Case 2:** A 21-year-old girl with malar rash, photosensitivity, and alopecia. ANA, anti-Smith, and anti-RNP were positive. Considering the possibility of SLE, the patient was started on HCQ. A Schirmer test was performed on the positive patient; the result was 3/3. A salivary gland biopsy was suggested to the patient but was declined.

**Case 3:** A 20-year-old girl with malar rash, photosensitivity, oral ulcers, and arthritis complaints was found to have positive ANA and anti-Smith in the tests and low serum complement levels. Considering the possibility of SLE, the patient was started on HCQ treatment. The patient's skin and joint symptoms improved with HCQ.

**Case 4:** A 15-year-old girl with malar rash, oral ulcers, and alopecia was found to have leukopenia, positive ANA, anti dsDNA, anti-Smith, anti-SSA, and anti-SSB, with low serum complement. The patient was diagnosed with SLE and started on HCQ and azathioprine. Due to arthritis and proteinuria, MPZ and MMF were added. A renal biopsy was planned but was declined. The patient became pregnant at the age of 28. In the third trimester, proteinuria increased up to 7.5 g/day; delivery was performed via cesarean section at 36 weeks. Postpartum follow-up showed a gradual decrease in proteinuria to below 1 g/day.

**Case 5:** A 13-year-old girl with malar rash, alopecia, and arthritis. Positive ANA and anti-Smith were detected. The patient had proteinuria. A renal biopsy was performed, revealing Class III lupus nephritis (LN). The patient was treated with MPZ, HCQ, and cyclophosphamide. At the age of 21, there was an increase in proteinuria, leading to another renal biopsy that showed Class IV LN and received rituximab. At the age of 25, the patient passed away due to sepsis.

**Conclusion:** C1q deficiency can manifest clinically across a broad spectrum, ranging from classic cutaneous involvement to a Sjögren-like phenotype.

**Date of birth::** avril 23,

**Patient Consent**

Yes, I received consent

**Disclosure of Interest**

None Declared

**References**

1. Afzali P, Isaeian A, Sadeghi P, et al. Complement deficiency in pediatric-onset systemic lupus erythematosus. J Lab Physicians. 2018;10(2):232-36.
2. Haşlak F, Kılıç Könte E, Aslan E, et al. Type I Interferonopathies in Childhood. Balkan Med J. 2023;40(3):165-74.

### Systemic lupus erythematosus and antiphospholipid syndrome

#### P432 Clinical characteristics at debut of juvenile systemic lupus erythematosus in hospitals and services of the social welfare institute: cohort 2018-2022

##### Najat Hijazi^1^, Carolina Aguilera^1^, Adrian Denis^1^, Zoilo Morel^1, 2^, Malena Arce^1, 3^, Carolina Estigarribia^1^, Monica Charotti^4^, Natalia Cabrera^1, 2^ on behalf of Paediatric RheumatologY Study Group (PY Study Group)

###### ^1^Paediatric Department, Instituto de Previsión Social, ^2^Sociedad Paraguaya de Reumatología, ^3^Sociedad Paraguaya de Pediatría, ^4^Central Laboratory , Instituto de Previsión Social, Asunción, Paraguay

####### **Correspondence:** Natalia Cabrera

*Pediatric Rheumatology 2024*, **22(2)**: PReS24-ABS-1436

**Introduction:** Systemic lupus erythematosus (SLE) is an autoimmune disease with a broad and variable clinical and laboratory spectrum (1,2). The unpredictable course and increased severity of juvenile SLE (jSLE) make description from the onset of the disease essential. Paraguay has a fragmented health system. The social security (IPS) has a system of hospitals and services distributed in the whole country and covers the proportion of the population linked to employment in the formal economy (3).

**Objectives:** To describe the clinical characteristics present at the onset of jSLE in the Instituto de Previsión Social (IPS): 2018-2022 cohort.

**Methods:** Descriptive, cross-sectional, retrospective observational study.

IPS covers 22% of the country's population (approx. 1,500,000 people), and implements health information systems for its hospitals.

We initially scanned the electronic medical records database using the ICD-10 M32 code and then, in selected cases (<16 years at debut, between 2017 and 2022) we searched the electronic laboratory, outpatient and inpatient records.

**Results:** A total of 41 patients diagnosed with jSLE between 01/01/2018 and 31/12/2022 were included, of which data on debut characteristics were collected for 33 patients (80.5%). Follow-up details were not available in 8 cases (19.5%).

The majority were female (n=35, 85.0%), mean age at diagnosis was 13.0 ± 2.3 years. ANA positive was found in 100% of the population at the time of debut. It is mentioned that 7 patients (17.1%) were diagnosed with early-onset jSLE (sex ratio = 0.75, M/F= 3/4, average age at debut was 8.3 and minimum 5 years). All with early-onset jSLE patients are still undergoing regular follow-up and symptomatic treatment.

58.5% came from the Central Area and Asuncion. Regarding follow-up: 59.0% with paediatricians, 19.5% lost sight of, 12.2% with adult rheumatologists. There were 4 deceased patients (9.75%). Among the clinical manifestations, haematological manifestations were the most frequent in 78.7%, followed by articular manifestations in 45.5% and cutaneous manifestations in 42.4%. Regarding specific autoantibodies, positive anti-DNA was found in 51.5% (n=17) of patients, while anti-Sm was found in 3% (n=1) of patients at debut.

**Conclusion:** In this cohort all patients had ANA 1/80 at debut. Haematological manifestations were the most frequent, followed by cutaneous and articular manifestations. Renal and neurological involvement were the least frequent. Institutional definitions are needed to code these cases and to better understand the details of the clinical manifestations and evolution of patients with jSLE in IPS hospitals (Paraguay).

**Date of birth::** janvier 16

**Patient Consent**

Not applicable (there are no patient data)

**Disclosure of Interest**

None Declared

**Reference**

1. Smith EMD et al. Clinical Immunology, 2019, 2. Massias JS et al. Lupus 2020, 3. Mancuello Alum et al. Rev. Salud Pública Parag, 2011

### Systemic lupus erythematosus and antiphospholipid syndrome

#### P433 Juvenile systemic lupus erythematosus revealing a family autoimmunity : a case report in bouaké

##### Aissata Doucoure Traore ^1^, Kan E. J. Koffi^1^, Christ Ziahy R. M. Koffi^2^, Joe C. Yao^1^, Ehaulier S. C. Kouakou^1^, Weu M. Tia^2^, Felix J. C. Daboiko^1^

###### ^1^Rhumatologie; ^2^Nephrologie, Universite Alassane Ouattara (BOUAKE), Bouake, Côte d'Ivoire

####### **Correspondence:** Aissata Doucoure Traore

*Pediatric Rheumatology 2024*, **22(2)**: PReS24-ABS-1084

**Introduction:** Juvenile Systemic Lupus erythematosus (jSLE) is a rare but severe multisystem autoimmune, inflammatory disease. It is defined by disease onset before the age of 18. Compared to adult-onset SLE, jSLE is more aggressive, with higher disease activity and medication burden. It is multi-factorial disease unknown etiology which of genetic factors play a critical role in the pathogenesis. We present a case report of a 15-year-old female diagnosed with jSLE, revealing a family autoimmunity, with a focus on the potential genetic predisposition.

**Objectives:** We present a case report of a 15-year-old female diagnosed with jSLE, revealing a family autoimmunity, with a focus on the potential genetic predisposition.

**Methods:** case presentation

**Results:** It is a 15-year-old girl, one of three children, from a non-consanguineous marriage. She was admitted to rheumatology department for polyarthritis of the large joints with malar rash that had been present for 6 months. There was medical history of fever, photosensitivity and flare-ups of inflammatory arthralgia affecting the large and small joints had begum since the age of 7. On interrogation, family investigations had found similar symptoms in the mother and father. On examination, the child had a malar erythema, the erythematous-scaly and purplish plaques on the face, palms, a recent alopecia, synovitis of the knees, ankles and wrists with asthenia and weight loss. Blood tests showed a normal erythrocyte sedimentation rate (ESR) of 120mm/h, C reactive protein of 40 mg/l, anemia 7g/dl and antinuclear antibodies (ANA), anti-native DNA, Anti-Sm were positive. other test was normal ( ferritinemia, proteinuria, ECBU, renal function, chest x-ray, hand and foot x-ray, and ECG). The diagnosis of jSLE retained on the basis of the EULAR-ACR 2019. She was put on systemic corticosteroid therapy, hydroxychloroquine and topical corticosteroids for the skin lesions. Immunology analysis revealed Sharp syndrome with ANA, anti-RNP positive in the mother and ANA in the father.

**Conclusion:** Understanding the genetic basis of jSLE will have significant implications for prognosis, treatment, and genetic counseling. In fact, immunology test, genetic analysis and screening family should be considered in jSLE. this case is an example.

**Date of birth::** avril 26,

**Patient Consent**

Yes, I received consent

**Disclosure of Interest**

None Declared

**References**

1. Charras, E. Smith, M.D.,and C.M. Hedrich. Systemic Lupus Erythematosus in Children and Young People. Curr Rheumatol Rep. 2021 ; 23(3) : 20
2. Ashournia, P. Sadeghi, N. Rezaei and M-H. Moradinejad. Prevalence of Family History of Autoimmune Disorders in Juvenile Systemic Lupus Erythematosus. Maedica (Bucur). 2018 Mar ; 13(1) : 21– 24

### Systemic lupus erythematosus and antiphospholipid syndrome

#### P434 Multiple clinical presentations of systemic lupus erythematosus: a long way to diagnosis - a clinical case

##### Sania Valieva, Maria Kapranova, Seda Kurbanova, Anna Sologub, Evgeniya Korobyants, Angelica Nargizyan, Marina Dzis, Agunik Marandgyan

###### Rheumatology, Morozov Children’s City Clinical Hospital, Moscow, Russian Federation

####### **Correspondence:** Maria Kapranova

*Pediatric Rheumatology 2024*, **22(2)**: PReS24-ABS-1292

**Introduction:** Systemic lupus erythematosus (SLE) is a multifactorial disease characterized by the variability of the clinical picture due to the production of organ-specific autoantibodies.

**Objectives:** The purpose of this study is to demonstrate a variety of clinical features of SLE.

**Methods:** Case report

**Results:** 15-year-old boy was admitted to the Children’s Hospital with complaints of periorbital oedema, normocytic normochromic anaemia. The preliminary diagnosis was angioedema, differential diagnosis was made with the violation of the integrity of the bones of the facial skull.

Anamnesis revealed prolonged lymphadenopathy, unsuccessful use of iron supplements, episodes of fever and weight loss. Laboratory tests in the department revealed haemolytic Coombs-positive anaemia, inflammatory activity (increased erythrocyte sedimentation rate); minimal proteinuria. According to the CT scan of the brain, there is no intracranial haemorrhage, no violation of the integrity of the bones of the skull.

Pleural ultrasound: there were signs of homogeneous fluid component with divergence of pleural layers. The patient was consulted by gastroenterologist, otolaryngologist, ophthalmologist, haematologist - no specific pathology was found. The teenager had oedema syndrome, polyserositis phenomena (ascites, pleurisy) and centrifugal erythema in the form of "butterfly", pain syndrome in the head area, exudative changes of knee joints. The child was examined by a rheumatologist and a neurologist, and additional tests were performed.

According to the results of the CT scan of the brain with IV contrast: image of thrombosis of superficial veins over the cerebellum, direct, left transverse, left sigmoid sinus with transition to the temporomandibular joint up to the level of the C1 vertebra, hypodense zone of the left temporal lobe were found out. Laboratory data included thrombocytopenia, immunological activity, hypocomplementemia. ENA profile blood test results showed high titer of antinuclear factor, antibodies to 2-helix DNA. Ultrasound of knee joints - signs of synovitis. Diagnosis: "Systemic lupus erythematosus, high activity, moderate severity". Complication of the disease: "Thrombosis of the superficial veins above the cerebellum, direct, left transverse, left sigmoid sinus". For pathogenetic purposes, pulse therapy with methylprednisolone, hydroxychloroquine and mycophenolate therapy, anticoagulant therapy, as well as biological therapy by rituximab were performed. On the background of treatment there was positive dynamic in boy’s condition with, clinical and haematological remission was achieved, too.

**Conclusion:** The diagnosis of systemic lupus erythematosus requires a multidisciplinary approach and is often challenging for clinicians. It is of paramount importance to be aware of any symptoms that deviate from the typical disease presentation and to be alert to the development of systemic disease.

**Date of birth::** janvier 07

**Patient Consent**

Yes, I received consent

**Disclosure of Interest**

None Declared

### Systemic lupus erythematosus and antiphospholipid syndrome

#### P435 Tuberculosis in sle patients: twist of fate or significant correlation

##### Shray Thakur^1^, Nandini Kaundal^1^, Sehar Asotra^1^, Avinash Sharma^2^

###### ^1^Dr. RPGMC Tanda, Kangra, India; ^2^Pediatrics, Dr. RPGMC Tanda, Kangra, India

####### **Correspondence:** Shray Thakur

*Pediatric Rheumatology 2024*, **22(2)**: PReS24-ABS-1298

**Introduction:** Systemic Lupus Erythematosus (SLE) is an autoimmune disease characterized by multisystemic inflammation and circulating autoantibodies. This causes an immunocompromised state leading to morbidity and mortality due to increased susceptibility to infections. Of particular interest is Tuberculosis (TB) superimposed on rheumatic diseases like SLE esp. in a TB-endemic country like India, as it complicates the diagnosis and treatment.

Here we report 2 female patients of SLE with TB.

**Objectives:** To see any correlation between SLE and TB infection.

**Methods: Case 1:-** 18-year female, presented with pain abdomen for 8 days, localized to left upper quadrant, insidious in onset, intermittent in nature, prominent during the evening hours.

Fever for 3 days, max. documented upto 103°F, exhibiting diurnal variation, single spike/day, primarily occurring at night, associated with chills and rigor.

She was diagnosed with SLE in 2019 when she presented with fever, malar rash, joint pain and oral ulcers but no evidence of nephritis, was started on tapering doses of prednisolone and hydroxychloroquine (HCQ). Currently she is on 15mg prednisolone everyday. History of anti-tuberculosis treatment (ATT) intake by grandmother 5 years ago.

On examination, respiratory rate was 24 breaths/min with marked pallor and lymphadenopathy. Urine culture showed *E. coli* so nitrofurantoin was initiated. Subsequently, thickening of the caecal wall was revealed on Ultrasound (USG), raising suspicion of TB. Sputum CB-NAAT (Cartridge-Based Nucleic Acid Amplification Test) along with Computed Tomography (CT) abdomen, helped form diagnosis of pulmonary and extrapulmonary TB.

4 drug ATT was initiated and patient was discharged once her fever subsided. Oral prednisolone and HCQ were continued. Patient is doing well on follow-up.

**Case 2:-** 17-year girl, presented with fever for 7 days, insidious in onset, max. documented upto 104°F, 2-3 spikes/day, showing diurnal variation.

Loose stools for 3 days, 2 to 3 episodes/day, associated with tenesmus, mild abdominal pain. Cough for 3 days, insidious in onset, non-productive.

Patient is a known case of SLE since 2018 with class 4 lupus nephritis, was started on pulse methylprednisolone therapy, followed by inj. cyclophosphamide.

On examination, respiratory rate was 22 breaths/min. Pitting edema was present on left lower limb, malar rash sparing the nasolabial fold and abdominal striae.

Blood investigations revealed anemia, raised Erythrocyte Sedimentation Rate and neutrophil count. Blood culture revealed methicillin sensitive *S. aureus.* Fever persisted despite appropriate antimicrobial therapy. Deep vein thrombosis of left lower limb was confirmed by USG Doppler, low molecular weight heparin started at 1mg/kg/day. Sputum CBNAAT revealed rifampicin sensitive MTB. 4 drug ATT was started and the patient improved significantly, remaining afebrile for 5 days, and was then discharged.

**Results:** Various studies show that there is a direct relation between rheumatic diseases and TB with the incidence being much higher in these patients than in the general population. Making a diagnosis of TB with SLE is complicated by the overlapping clinical manifestations and increased risk of extrapulmonary TB and so gets delayed. These are exacerbated by high doses of corticosteroids and immunosuppressive agents, and hence these should be used cautiously.

**Conclusion:** A clinician should always consider TB as a possible diagnosis in patients of SLE esp. in countries with high TB burden. Latent Tuberculosis Infection screening along with preventive treatment for SLE patients can be an effective means to reduce TB incidence and improve patient outcome.

**Date of birth:** mai 12, YY

**Patient Consent**

Yes, I received consent

**Disclosure of Interest**

None Declared

**References**

1. Arenas Miras Mdel M, Hidalgo Tenorio C, Jimenez Alonso J. Tuberculosis in patients with systemic lupus erythematosus: Spain's situation. Reumatol Clin. 2013 Nov-Dec;9(6):369-72.
2. Hamijoyo L, Sahiratmadja E, Ghassani NG, Darmawan G, Susandi E, van Crevel R, Hill PC, Alisjahbana B. Tuberculosis Among Patients With Systemic Lupus Erythematosus in Indonesia: A Cohort Study. Open Forum Infect Dis. 2022 Apr 12;9(7).
3. Xiao X, Da G, Xie X, Liu X, Zhang L, Zhou B, Li H, Li P, Yang H, Chen H, Fei Y, Tsokos GC, Zhao L, Zhang X. Tuberculosis in patients with systemic lupus erythematosus-a 37-year longitudinal survey-based study. J Intern Med. 2021 Jul;290(1):101-115.
4. Chatterjee R, Pattanaik SS, Misra DP, Agarwal V, Lawrence A, Misra R, Aggarwal A. Tuberculosis remains a leading contributor to morbidity due to serious infections in Indian patients of SLE. Clin Rheumatol. 2023 Aug;42(8):2079-2090.
5. Al-Arbi KMS, Magula NP, Mody GM. Tuberculosis remains a major burden in systemic lupus erythematosus patients in Durban, South Africa. Front Med (Lausanne). 2023 Mar 1;10:1118390.
6. Wu Q, Liu Y, Wang W, Zhang Y, Liu K, Chen SH, Chen B. Incidence and prevalence of tuberculosis in systemic lupus erythematosus patients: A systematic review and meta-analysis. Front Immunol. 2022 Jul 22;13:938406.
7. Bhattacharya PK, Jamil M, Roy A, Talukdar KK. SLE and Tuberculosis: A Case Series and Review of Literature. J Clin Diagn Res. 2017 Feb;11(2):OR01-OR03.
8. Chu AD, Polesky AH, Bhatia G, Bush TM. Active and latent tuberculosis in patients with systemic lupus erythematosus living in the United States. J Clin Rheumatol. 2009 Aug;15(5):226-9.

### Systemic lupus erythematosus and antiphospholipid syndrome

#### P436 Baricitinib monotherapy in a patient with refractory juvenile systemic lupus erythematosus: a case report

##### Inga Z. Turtsevich^1^, Lea Solman^2^, Muthana Al Obaidi^1^, Charalampia Papadopoulou^1^, Sandrine Compeyrot-Lacassagne^1^, Paul A. Brogan^1, 3^, Despina Eleftheriou^1,3^, Elena Moraitis^1^

###### ^1^Paediatric Rheumatology; ^2^Paediatric Dermatology, Great Ormond Street Hospital NHS Foundation Trust; ^3^Infection, Immunity and Inflammation, University College London Great Ormond Street Institute of Child Health, London, United Kingdom

####### **Correspondence:** Inga Z. Turtsevich

*Pediatric Rheumatology 2024*, **22(2)**: PReS24-ABS-1719

**Introduction:** Type I interferons (IFN) are involved in systemic lupus erythematosus (SLE) pathogenesis. Janus kinase inhibitors (JAK) displayed efficacy in several autoimmune conditions including SLE in adults, and in cases of interferon-mediated inflammation in children. However, juvenile SLE (jSLE) studies on efficacy and safety of JAK inhibitors are lacking.

**Objectives:** To describe a case of JSLE refractory to standard treatment, treated with Baricitinib, a JAK1/2 inhibitor.

**Methods:** Case report

**Results:** A 15-year-old female was diagnosed with jSLE at the age of 9 years old, with constitutional symptoms, severe cutaneous involvement, polyarthritis, pulmonary hypertension, alopecia, fatigue, positive ANA, anti-Ro/La, anti-Sm, anti-RNP and antiphospholipid antibodies. Treatment with glucocorticoids, hydroxychloroquine and cyclophosphamide was initiated, followed by azathioprine maintenance treatment. From the age of 10 years to 15 years, the patient remained on prednisolone at doses 0.5-1 mg/kg body weight, had four courses of rituximab, azathioprine was switched to mycophenolate mofetil (MMF), and methotrexate (MTX) was added in. Intravenous immunoglobulin (IVIG) was added in at the age of 12 years. Prolonged high doses of steroids required to control the disease led to osteoporosis and Kienbock disease. Interferon gene assay revealed upregulation of type I interferon genes. At the age of 15 years, Baricitinib was introduced initially at a dose of 4 mg daily and subsequently increased to twice a day, and MMF and MTX were discontinued. The initiation of Baricitinib led to resolution of arthritis and cutaneous features, decrease in SLEDAI and normalisation of IFN signature. Subsequently, prednisolone was tapered, and IVIG discontinued. At 4 years follow up the patient remains in remission on baricitinib and no side effects reported.

**Conclusion:** Baricitinib monotherapy in this case of SLE refractory to standard treatment and steroid dependent led to clinical improvement accompanied by downregulation of IFN gene responses. Further prospective studies are warranted to elucidate long-term efficacy of Baricitinib in jSLE.

**Patient Consent**

Yes, I received consent

**Disclosure of Interest**

None Declared

**References**

1. Ma L, Peng L, Zhao J, Bai W, Jiang N, Zhang S, et al. Efficacy and safety of Janus kinase inhibitors in systemic and cutaneous lupus erythematosus: A systematic review and meta-analysis. Autoimmun Rev. 2023 Dec 1;22(12):103440
2. Petri M, Bruce IN, Dörner T, Tanaka Y, Morand EF, Kalunian KC, et al. Baricitinib for systemic lupus erythematosus: a double-blind, randomised, placebo-controlled, phase 3 trial (SLE-BRAVE-II). Lancet [Internet]. 2023 Mar 25 [cited 2024 May 15];401(10381):1011–9. Available from: https://pubmed.ncbi.nlm.nih.gov/36848919/

### Systemic lupus erythematosus and antiphospholipid syndrome

#### P437 Uncommon presentations of childhood systemic lupus erythematosus

##### Diksha Katoch, Avinash Sharma, Sandesh Guleria

###### Paediatrics, Dr Rajendra Prasad Medical college, Kangra, India

####### **Correspondence:** Diksha Katoch

*Pediatric Rheumatology 2024*, **22(2)**: PReS24-ABS-1747

**Introduction:** Systemic Lupus erythematosus (SLE) is a multisystemic autoimmune disease with varying clinical presentation from mild mucocutaneous involvement to multiorgan involvement. Nearly every organ system can be involved in SLE. Thus, diagnosis can be challenging while dealing with atypical presentations and management is dictated by organ system involvement.

**Objectives:** To broaden the horizon of initial presentations of children with SLE.

**Methods:** Case records of children diagnosed with SLE and registered in pediatric rheumatology clinic, department of pediatrics, Dr. Rajendra Prasad Government Medical College, Tanda, between July 2017 to July 2023, were reviewed.

**Results:** We describe three patients between 7 to 17 years admitted to a our medical college with unusual presentations which were subsequently evaluated and diagnosed as pediatric SLE. 6 years old female, initially presented as a case of nephrotic syndrome. Initial investigation were normal and patient received treatment for 12 weeks. After 8months, again presented with complaints of recurrent nasal bleeds, petechiae and ecchymosis over abdomen and lower limbs. No other stigmata was present. On investigations found to have thrombocytopenia with platelet counts 10,000/ml, direct coomb test negative, ANA positive(1:640,homogenous), low C3 levels. Patient was started on oral prednisolone and hydroxychloroquine. Within 4 weeks of treatment platelet counts increased to 45000/ml.

17 years old female adolescent presented with malar rash and abnormal body movements for 1.5 months, calf pain for 15days. On examination, malar rash, livedo reticularis, non-scarring alopecia was present. Lab investigations revealed anemia, direct coomb test positive, ANA positive(1:640, speckled), low C3 and C4 levels, activated partial thromboplastin time was positive and dilute Russell viper venom time test was positive. Patient was diagnosed as case of SLE with Antiphospholipid syndrome and was started on oral prednisolone, hydroxychloroquine and Mycophenolate.

15yrs old male adolescent presented with complaint of pain and swelling in multiple peripheral small and large joints for 4 months with signs of inflammation present. Initially possibility of polyarticular Juvenile Idiopathic arthritis kept. Investigations revealed mild anemia, urine examination showed non-nephrotic proteinuria and hematuria, ANA positive(1:640, speckled), dsDNA positive, low C3 and C4. Patient was started on methotrexate and hydroxychloroquine after which patient improved symptomatically.

**Conclusion:** SLE can have unconventional presentation. Thus a great degree of suspicion is required for accurate diagnosis, as delay can lead to disease progression and poor outcome.

**Date of birth:** février 16

**Patient Consent**

Yes, I received consent

**Disclosure of Interest**

None Declared

### Systemic lupus erythematosus and antiphospholipid syndrome

#### P438 Diffuse alveolar haemorrhage as initial manifestation of systematic lupus erythematosus in a teenage girl

##### Maria Tsinti, Stefania Andriakopoulou, Elena Tsitsami

###### Pediatric Rheumatology Unit First Department of Pediatrics, University of Athens, Children's Hospital "Agia Sofia", Athens, Greece

####### **Correspondence:** Maria Tsinti

*Pediatric Rheumatology 2024*, **22(2)**: PReS24-ABS-1749

**Introduction:** Introduction: Diffuse alveolar hemorrhage (DAH) is an acute, rare, life-threatening manifestation of systemic lupus erythematosus (SLE). Early recognition and detection of possible underlying pulmonary infection is critical.

**Objectives:** Objectives: Τo report the clinical presentation of DAH and treatment response in a child with SLE.

**Methods:** case report

**Results:** Results: A 14-year-old Caucasian female patient with a history of one month fatigue and weight loss presented to the emergency department due to recurrent fever and symptomatic anemia. Recent medical history revealed three febrile episodes with associated cough the past 25 days including a confirmed diagnosis of Influenza A. Laboratory findings on admission included significant anemia (hemoglobin 7.2 g/dl), leukopenia (WBC 3140 x 103/μl), high ESR (126 mm), microscopic hematuria and positive direct Coombs test. Chest X-ray and CT scan showed alveolar pattern and crazy paving appearance, respectively; findings consistent with infection and/or diffuse alveolar hemorrhage. Broad-spectrum antibiotics were initiated. Immunologic blood tests showed ANA titer 1:1280, anti-dsDNA 195 IU/ml, C3 26 mg/dl, C4 3 mg/dl. On the diagnosis of SLE pulse methylprednisolone 1 gr for 3 days, hydroxychloroquine and mycophenolate mofetil were initiated. Patient’s condition, laboratory and CT scan findings were significantly improved in the following week. However, on the 18th day of hospitalization, the patient developed mild renal impairment (BUN 121mg/dl, creatinine 1.18 mg/dl), while otherwise stable. Three days later, she developed fever, hemoptysis, hemodynamic instability, hypoxemia and drop in hemoglobin (from 10.8 to 8.5 g/ dl). Chest-X-ray showed bilateral pulmonary infiltrates. The child was admitted to ICU and received HFNC oxygen therapy, iv piperacillin/tazobactam plus teicoplanin and voriconazole as well as 3 days methylprednisolone pulse. Bronchoscopy was performed. BAL findings were suggestive of DAH and pulmonary infection (95,8% neutrophils, presence of Aspergillus spp. DNA). On the 4th day of antibiotic treatment iv cyclophosphamide was administered (6 fortnightly doses totally). She also received a 6 weeks-course of pos voriconazole. Renal biopsy was performed when patient stabilized and revealed class III focal lupus nephritis. The girl received maintenance therapy with hydroxychloroquine and mycophenolate mofetil and has achieved remission for 17 months. In the 12th month of remission COVID-19 infection manifested as mild febrile upper respiratory infection and did not trigger disease relapse.

**Conclusion:** Conclusion: DAH is a severe condition associated with SLE and should be suspected in patients with dyspnea, sudden drop in hemoglobin and bilateral pulmonary infiltrates. DAH is associated with active nephritis in patients with SLE and it is often suggestive of underlying pulmonary infection..

**Patient Consent**

Yes, I received consent

**Disclosure of Interest**

None Declared

### Treatment

#### P444 Preliminary report on the prospective study on MMR vaccine in children with rheumatic diseases treated with dmards and/or biologics

##### Masa Bizjak ^1, 2^, Foteini Dasoula^3^, Barbora Balaziova^4^, Amra Adrovic^5^, Amit Ziv^6, 7^, Merav Heshin-Bekenstein ^7, 8^, Despoina Maritsi^3^, Tomas Dallos^4^, Ozgur Kasapcopur^5^, Yosef Uziel^6, 7^, Natasa Toplak^1, 2^ and PReS Vaccination Working Party

###### ^1^Department of Allergology, Rheumatology and Clinical Immunology, University Children's Hospital, University Medical Centre Ljubljana; ^2^Medical Faculty, University of Ljubljana , Ljubljana , Slovenia; ^3^Second Department of Pediatrics, P.&A. Kyriakou Children’s Hospital, National and Kapodestrian University of Athens, Athens, Greece; ^4^Department of Pediatrics, Comenius University Medical School in Bratislava, National Institute of Children’s Diseases, Bratislava, Slovakia; ^5^Department of Pediatric Rheumatology, Istanbul University-Cerrahpasa, Istanbul, Türkiye; ^6^Pediatric Rheumatology Unit, Department of Pediatrics, Meir Medical Center, Kfar Saba; ^7^Tel Aviv University; ^8^Pediatric Rheumatology Service, Dana-Dwek Children’s Hospital, Tel Aviv Sourasky Medical Center, Tel Aviv, Israel

####### **Correspondence:** Masa Bizjak

*Pediatric Rheumatology 2024*, **22(2)**: PReS24-ABS-1497

**Introduction:** According to the EULAR/PReS recommendations, measles-mumps-rubella (MMR) booster vaccine can be considered in children with rheumatic diseases (RD) on specified biologic disease-modifying antirheumatic drugs (bDMARDs), but the level of evidence for this approach is low (1).

**Objectives:** To prospectively evaluate safety and long-term immunogenicity of the MMR vaccine in children with RD treated with immunosuppressive therapy.

**Methods:** This is an ongoing multinational, multicentre prospective study. Patients with RD on conventional synthetic (cs), biologic (b) or targeted synthetic (ts) DMARDs with stable disease were included if they were scheduled to receive the 2^nd^ dose of MMR vaccine according to their National Immunisation Programme. In case of favourable safety profile of the 2^nd^ dose of the vaccine in the first 50 included patients, the 1^st^ dose would be considered in areas with measles outbreaks. Controls were patients without therapy and healthy children. Infections with vaccine or wild-type viruses and adverse events (AE) were monitored after vaccination and disease activity before and after vaccination. Protective antibodies were measured before and at predetermined time points after vaccination.

**Results:** By the end of April 2024, 4 patients received the 1^st^ dose and 67 patients the 2^nd^ dose of MMR vaccine while treated with csDMARDs and/or bDMARDs. Two patients who received the 1^st^ dose were on bDMARDs and csDMARDs, one on bDMARD and one on csDMARD. Among patients who received the 2^nd^ dose, 26 were on bDMARDs and csDMARDs, 20 each on bDMARDs/csDMARDs and 1 on tsDMARD. Majority (89%) had juvenile idiopathic arthritis. Median age at diagnosis was 2.8 yrs (range 0.6-12.3 yrs), at 1^st^ dose of MMR vaccine 1.2 yrs (range 0.6-4.4 yrs) and at 2^nd^ dose 8.3 yrs (range 2.5-15.8 yrs). There were no vaccine strain infections, serious AE or disease flares after vaccination. Twenty eight percent of patients reported mild AE (fever, fatigue, arthralgia, myalgia, headache, cough). Protective antibodies against measles and mumps were positive in 75% of patients before 2^nd^ dose, in 95% at 2-3 months, in 94% for measles and 100% for mumps at 12-18 months after vaccination.

**Conclusion:** The 2^nd^ dose of MMR vaccine was safe and mostly immunogenic in children with RD treated with immunosuppressive therapy. Our results strengthen the updated EULAR\PReS recommendations to give booster MMR vaccine to children with RD treated with bDMARDs. Furthermore, four patients received the 1^st^ dose of MMR vaccine due to the epidemiological situation and it was safe in all of them.

**Patient Consent**

Not applicable (there are no patient data)

**Disclosure of Interest**

None Declared

**Reference**

1. Jansen MHA, Rondaan C, Legger GE, et al. Ann Rheum Dis 2023;82:35–47.

### Treatment

#### P445 Olokizumab, a monoclonal antibody against IL-6, in Polyarticular-course Juvenile Idiopathic Arthritis (PCJIA): results of 24 weeks of the phase 2 open-label clinical trial

##### Ekaterina Alexeeva^1^, Tatiana Dvoryakovskaya^1^, Elena Zholobova^2^, Daria Bukhanova^3^, Alina Egorova^3^, Sergey Grishin^3^, Mikhail Samsonov^3^, Mikhail Kostik^4^, Irina Nikishina^5^

###### ^1^Federal State Autonomous Institution “National Medical Research Center of Children's Health” of the Ministry of Health of the Russian Federation; ^2^First Moscow State Medical University n.a. I.M.Sechenov; ^3^R-Pharm, Moscow; ^4^Pediatric Department, Saint-Petersburg State Pediatric Medical University, Saint Petersburg; ^5^Pediatric Department, V. A. Nasonova Research Institute of Rheumatology, Moscow, Russian Federation

####### **Correspondence:** Irina Nikishina

*Pediatric Rheumatology 2024*, **22(2)**: PReS24-ABS-1718

**Introduction:** Olokizumab (OKZ) is a direct interleukin-6-inhibitor for treatment of rheumatoid arthritis and is being investigated in the open-label Phase 2 trial in adolescents with polyarticular-course juvenile idiopathic arthritis (pcJIA).

**Objectives:** The aim of this study is to assess the pharmacokinetics (PK), effectiveness and safety of OKZ in patients with pcJIA who had inadequate response to methotrexate (MTX).

**Methods:** Adolescent (12-17 years old) patients with active pcJIA received OKZ in a dose of 64 mg every 4 weeks (q4w) subcutaneously (SC) in open regimen during the main 24-weeks part of the study. The following outcomes were analyzed: JIA American College of Rheumatology 30/50/70% responses (ACR pedi 30/50/70 responses), Juvenile Arthritis Disease Activity Score-71 (JADAS-71), its components, C-reactive protein (CRP), and Childhood Health Assessment Questionnaire (CHAQ), PK parameters. The statistical analysis utilized the method of descriptive statistics. The significance of change from baseline was tested using the Wilcoxon signed-rank test.

**Results:** Totally 16 patients: 9 [56.3%] girls and 7 [43.8%] boys were enrolled; median (25%; 75%) age at inclusion was 14.0 (13.0; 16.5) years, at pcJIA onset 11.8 (5.4; 13.7) years, and pcJIA duration was 3.4 (0.4; 8.9). Concomitant MTX received 12 (75.0%) patients, and 9 (56.3%) patients experienced bDMARDs treatment earlier, of them 8 had ≥2 previous bDMARDS. 43.7% patients were RF-positive and ANA was found in 56.3%.

By 24 weeks of treatment, all studied JIA outcomes significantly (p<0.01) decreased from the baseline: ΔAJC = - 66.7%, ΔJADAS71= - 61.5%, ΔCHAQ= -66.7% (p<0.05), ΔPVAS= -80.8%, ΔMDVAS= -61.8%, ΔESR= -75.0%, ΔCRP= -81.8%. JIA ACR 30/50/70 response was achieved in 80%, 73.3%, 46.7% of patients, respectively. No pcJIA flares were registered. In 5 (33,3%) patients inactive disease status was achieved at week 24. Median T_max_ was 168 hours, AUC_(0-24)_ - 32309.0 μg/ml, C_max_ 13.3 μg/ml. Treatment-emergent adverse events (AEs) were reported in 12 (75%) patients. The most frequent AEs were ordinary infections, in 6 (37.5%) patients, all resolved without complications. No Grade 3 or 4 AEs, serious AEs or deaths were reported. There was 1 case of Grade 2 neutropenia, which resolved spontaneously. One patient discontinued treatment at Week 12 due to psoriasis de novo. In one patient local injection reaction (6.2%) was observed.

**Conclusion:** OKZ in patients with pcJIA was associated with improvement in disease activity and functional health status. Safety was expected for this class of agents, but it seems OKZ has advantages regarding the risk of developing neutropenia.

**Patient Consent**

Not applicable (there are no patient data)

**Disclosure of Interest**

E. Alexeeva Speaker Bureau with: Roche, Novartis, Johnson & Johnson, T. Dvoryakovskaya Speaker Bureau with: Novartis, Johnson & Johnson, E. Zholobova Grant / Research Support with: Pfizer, Novartis, Speaker Bureau with: Abbvie, Pfizer, Roche, Novartis, D. Bukhanova Employee with: R-Pharm, A. Egorova Employee with: R-Pharm, S. Grishin Employee with: R-Pharm, M. Samsonov Employee with: R-Pharm, M. Kostik: None Declared, I. Nikishina Speaker Bureau with: Pfizer, Novartis, R-Pharm, Ipsen, Janssen

### Treatment

#### P446 Belimumab in the treatment of systemic lupus erythematosus with juvenile onset: results of a single-center retrospective study

##### Maria Kaleda, Irina Nikishina, Anna Shapovalenko, Tamara Pachkoria

###### Pediatrics, V. A. Nasonova Research Institute of Rheumatology, Moscow, Russian Federation

####### **Correspondence:** Irina Nikishina

*Pediatric Rheumatology 2024*, **22(2)**: PReS24-ABS-1119

**Introduction:** The treatment of systemic lupus erythematosus with juvenile onset (jSLE) remains a difficult task, taking into account the more aggressive course of the disease, requiring the appointment of various therapy regimens, including mainly a combination of high doses of glucocorticoids (GC) with immunosuppressive drugs, which on the one hand improves control by the course of the disease, but on the other hand leads to an increase in serious adverse effects from therapy. Modern therapy capabilities have improved significantly with the advent of the belimumab (BEL) - first and alone registered biologics for children with SLE.

**Objectives:** based on an open single-center retrospective study, to analyze the efficacy and safety of BEL in children with SLE.

**Methods:** The study included all patients (pts) with jSLE who were observed in the pediatric department of V. A. Nasonova Research Institute of Rheumatology and received at least 1 infusion of BEL. Diagnosis of SLE was based on SLICC criteria, 2012 year. The efficacy of therapy was evaluated among all pts who received BEL for 6 months or more, and safety in all who received at least 1 infusion.

**Results:** The study included 31 pts, 24 girls/7 boys. The median (Me) age at onset of jSLE was 12.6 [10.18; 13.5] years, the Me duration of the disease at the time of initiation of BEL therapy was 2.15 [0.9; 4.4] years. The Me activity on the SLEDAI at the time of diagnosis verification was 12 [9;17.5], at the time of start of BEL – 8 [6;12], 35,5% pts had severe activity, 51.6% - moderate, 12.9% - mild activity. The dose of GC per os at start of BEL was 15 [10; 21.25] mg/day, 32.26% of pts received a high dose of GC, 54.84% - moderate dose, 12.9% - low dose. 9 pts had SDI≥1, Me 1 [1;2]. After 6 months of therapy, the Me disease activity according to SLEDAI was 4 [2;6], the dose of GC per os was reduced to 10 [8.25; 17.5] mg/day. In 15 pts, a decrease in antiDNA was recorded (57.7% of those who initially had elevated values of antiDNA), in 9 - the level of complement was normalized (50% of pts who had hypocomplementemia at start). After 12 months of therapy, the Me SLEDAI was 4 [2;4] (p=0.034), the dose of GC per os was 5 [5; 8,125] mg/day (p=0.012). 5 pts completed therapy within 12 months or more: 1 patient - remission, 4 pts - secondary inefficiency. BEL was well tolerated, with the exception of three cases of serious adverse reactions (9.7%): prolonged diarrheal syndrome (after the 1^st^ infusion), Lyell's syndrome (after the 2^nd^ infusion), infusion reaction (during the 2^nd^ infusion). During the therapy of BEL no new damage were recorded; SDI decreased in 2 pts.

**Conclusion:** BEL in pts with jSLE demonstrated high efficacy with a decrease in the activity of the disease according to SLEDAI, normalization of antiDNA and complement, the possibility of a significant reduction the dose of GC, the absence of progression of the SDI with a good safety profile in the vast majority of pts.

**Patient Consent**

Yes, I received consent

**Disclosure of Interest**

None Declared

### Treatment

#### P447 Overcoming methotrexate intolerance in a pediatric cohort of patients with rheumatic diseases - successful application of a large interval oral split-dose regimen

##### Fabian Speth^1^, Sarah-Jolan Bremer^1^, Julia Pagel^1^, Anja Fröhlich^1^, Susan Farmand^1^, Kleimeyer Christian^1^, Müller Ingo^2^, Ania C. Muntau^3^

###### ^1^Division of Pediatric Rheumatology; ^2^Clinic of Pediatric Hematology and Oncology; ^3^Director of the department, Department of Pediatrics, University Hospital Hamburg-Eppendorf , Hamburg, Germany

####### **Correspondence:** Fabian Speth

*Pediatric Rheumatology 2024*, **22(2)**: PReS24-ABS-1482

**Introduction:** Methotrexate (MTX) is the most commonly used disease-modifying antirheumatic drug to treat rheumatic diseases in children. In up to half of the patients, the conventional once-weekly dosage will lead to MTX intolerance (1).

**Objectives:** To investigate whether an existing MTX intolerance can be overcome by switching patients from once-weekly dosage to a large interval oral split-dose (LIOS) MTX regimen.

**Methods:** The clinical data of 26 pediatric patients with rheumatic diseases were analyzed retrospectively. Ethical approval and informed consent of all patients have been obtained. All included patients wanted to stop their conventional once-weekly dosage MTX therapy prematurely in the course of the disease due to intolerance that did not respond to at least two of the following countermeasures: switching MTX from oral to subcutaneous administration or vice versa, use of antiemetics, or covert dosing by the parents. The patients were switched to the LIOS MTX regimen as follows: the pre-existing once-weekly MTX dose of 10-15mg/m^2^/week (maximum 20mg) was divided into two reduced oral doses of 50% each (maximum 10mg) at three and four-day intervals. To compare side effects before and after changing the administration regimen, we used the Methotrexate Intolerance Severity Score (MISS) (2), collected data on mental stress of parents during the drug administration procedure and analyzed laboratory parameters.

**Results:** All patients were able to continue MTX therapy for at least one year after switching to the LIOS MTX regimen. Average MISS values decreased significantly from 12.3 to 2.8 (p <0.0001) and thus far below the threshold of 6 points that define MTX intolerance. In line with this, parental mental stress during the drug administration improved in 24/26 cases (92 %). No relevant changes were observed for laboratory parameters or disease activity after switching to the LIOS MTX regimen.

**Conclusion:** Switching patients with MTX intolerance to the LIOS MTX regimen enables long-term continuation of treatment with significantly reduced side effects. We suggest that the positive observations of this first pilot study on three and four-day intervals oral split dose MTX therapy in children with rheumatic diseases should be further investigated in a prospective randomized controlled trial.

**Patient Consent**

Yes, I received consent

**Disclosure of Interest**

None Declared

**References**

1. Hügle B, Van Dijkhuizen EHP. MTX intolerance in children and adolescents with juvenile idiopathic arthritis. Rheumatol (United Kingdom). 2020;59(7):1482–8.
2. Bulatović M, Heijstek MW, Verkaaik M, Van Dijkhuizen EHP, Armbrust W, Hoppenreijs EPA, et al. High prevalence of methotrexate intolerance in juvenile idiopathic arthritis: Development and validation of a methotrexate intolerance severity score. Arthritis Rheum. 2011;63(7):2007–13.

### Treatment

#### P448 Safety, effectiveness and immunogenicity of varicella vaccination in children with juvenile idiopathic arthritis treated with biologic therapy

##### Masa Bizjak^1, 2^, Andreja Natasa Kopitar^3^, Alojz Ihan^3^, Miroslav Petrovec^3^, Tadej Avcin^1, 2^, Natasa Toplak^1, 2^

###### ^1^Department of Allergology, Rheumatology and Clinical Immunology, University Children's Hospital, University Medical Centre Ljubljana; ^2^Medical Faculty, University of Ljubljana; ^3^Institute of Microbiology and Immunology, Faculty of Medicine, University of Ljubljana, Ljubljana, Slovenia

####### **Correspondence:** Masa Bizjak

*Pediatric Rheumatology 2024*, **22(2)**: PReS24-ABS-1526

**Introduction:** Children with juvenile idiopathic arthritis (JIA) on immunosuppressive therapy are at risk for severe varicella. The most effective method for protection against varicella is vaccination, but data on live-attenuated vaccines in patients on biologic disease-modifying antirheumatic drugs (bDMARDs) are scarce.

**Objectives:** To prospectively evaluate safety, effectiveness and long-term immunogenicity of varicella vaccination in children with JIA, treated with bDMARDs.

**Methods:** This is an ongoing prospective case-control study. Varicella zoster virus (VZV)-naÏve patients with stable JIA on bDMARDs and normal values of lymphocyte populations, who were at risk for contracting varicella, were vaccinated against VZV. Adverse events (AE), disease activity and effectiveness were monitored after vaccination. VZV-specific humoral and cellular immunity were measured at predetermined time points after vaccination by LIAISON and intracellular cytokine staining, respectively. In age-matched healthy controls (HC; healthy children after varicella infection or VZV vaccination), immune response was measured once after infection/vaccination.

**Results:** To date, 16 patients were vaccinated against VZV while on bDMARDs; 13/16 received the 1^st^ and the 2^nd^ dose, 2/16 the 2^nd^ dose and 1/16 the 3^rd^ dose of VZV vaccine on bDMARDs (12 on TNFα-inhibitors, 3 on IL6-inhibitors, 1 on IL1-inhibitor; 11 were concomitantly on csDMARDs). Fifty-two HC after VZV vaccination and 52 HC after varicella infection were included. There were no serious AE or vaccine-strain infections after vaccination. Two patients had an increase in disease activity within 3 months after the 2^nd^ dose. Six patients (37%) and 11 HC (21%) reported mild AE, including injection site reactions, fever, rash and arthralgia (p=0.2). Four patients had mild breakthrough varicella 4 months – 4.5 years after vaccination (mean follow-up time 8.9 ± 3.3 years). Further 6 patients had a documented contact with varicella but did not contract it. No HC got varicella after vaccination (mean follow-up time 2.6 ± 1.5 years). After 2^nd^ vaccination 12/15 patients developed VZV-specific humoral immunity and 9/13 patients VZV-specific cellular immunity, which persisted longer than humoral. Anti-VZV IgG levels were significantly higher in vaccinated HC compared to patients for up to 1.5 years and comparable at 2-3 years after vaccination. Compared to HC after varicella infection, patients had significantly lower levels of anti-VZV IgG during the 3 years after vaccination.

**Conclusion:** Vaccination against varicella was safe, but not always effective or immunogenic in children with JIA, treated with bDMARDs. However, it was effective in preventing severe disease in all vaccinated patients. VZV-specific cellular immunity could offer an additional insight into the immunogenicity of the vaccine.

**Patient Consent**

Not applicable (there are no patient data)

**Disclosure of Interest**

None Declared

### Treatment

#### P449 Autologous Hematopoietic Stem Cell Transplantation (AHSCT) for treatment-refractory autoimmune diseases in children

##### Özlem Satirer^1^, joerg Henes^2^, Michaela Döring^3^, Till Lesk^4^, Susanne M Benseler^5, 6^, Jasmin Kümmerle-Deschner^1^

###### ^1^Division of Paediatric Rheumatology, Department of Paediatrics and Autoinflammation reference Center Tuebingen (arcT); ^2^Centre for Interdisciplinary Clinical Immunology, Rheumatology and Auto-inflammatory Diseases and Department of Internal Medicine; ^3^Department I - General Pediatrics’, Hematology/Oncology; ^4^University Hospital Tuebingen, Tübingen, Germany; ^5^Rheumatology, Department of Pediatrics, Alberta Children's Hospital (ACH), ACH Research Institute, University of Calgary, Calgary, Canada; ^6^Childrens Health Ireland, Dublin, Ireland

####### **Correspondence:** Özlem Satirer

*Pediatric Rheumatology 2024*, **22(2)**: PReS24-ABS-1070

**Introduction:** Autoimmune diseases constitute a broad spectrum of conditions characterized by immune system dysfunction, leading to inflammation and affecting multiple organs. Despite advancements in treatments such as biologics and targeted synthetic drugs, certain patients continue to grapple with severe forms of autoimmune disease that resist conventional therapies. In such cases, autologous hematopoietic stem cell transplantation (AHSCT) has emerged as a viable alternative, having been employed for over two decades to address treatment-resistant autoimmune diseases.

**Objectives:** To evaluate the long-term effectiveness and safety of autologous hematopoietic stem cell transplantation (AHSCT) for severe, refractory autoimmune diseases in paediatric patients.

**Methods:** A single centre study of consecutive children and adolescents with refractory autoimmune diseases undergoing AHSCT was performed. Demographics, clinical, laboratory features, pre-AHSCT medications, disease activity and functional status were captured. The primary outcome was progression free survival, secondary outcomes included overall survival, disease-specific treatment responses, disease activity at the last follow-up and AHSCT safety.

**Results:** The study included seven patients: 2 systemic sclerosis, 1 pansclerotic morphea, 1 eosinophilic fasciitis, 1 JDM and 2 sJIA patients. These were 4 females, 3 males with a median age at AHSCT of 10 years (7-19), median follow-up post-AHSCT of 17 years. Median progression free survival and overall survival was 4.2 years (95% CI: 0.98-8.3) and 17 years (95% CI: 11.8-22.1), respectively.Progression free survival rates at 1 and 2 years post-AHSCT were 100% and 77%, respectively. All children survived. All patients are in clinical remission, of whom only 4 require ongoing immunotherapy. Safety: Three patients experienced infections most commonly HHV6; one developed a systemic inflammatory response syndrome (SIRS); two developed new onset secondary autoimmune diseases and one was found to have a breast fibroadenoma. Treatment toxicity: one cyclophosphamide-associated transient renal failure and one patient with amenorrhea/infertility.

**Conclusion:** AHSCT was an effective and safe approach for children and adolescents with treatment-refractory autoimmune diseases. The indication and timing of transplantation requires a careful consideration and a multidisciplinary approach.

**Patient Consent**

Yes, I received consent

**Disclosure of Interest**

None Declared

**References**

1. Alexander, T., et al., *[Autologous hematopoietic stem cell transplantation for autoimmune diseases : Current indications and mode of action, a review on behalf of the EBMT Autoimmune Diseases Working Party (ADWP)].* Z Rheumatol, 2020. 79(5): p. 419-428.
2. Alexander, T., R. Greco, and J.A. Snowden, *Hematopoietic Stem Cell Transplantation for Autoimmune Disease.* Annu Rev Med, 2021. **72**: p. 215-228.

### Treatment

#### P450 Tofacitinib treatment of different variants of JIA : single center experience of real clinical practice

##### Svetlana Arsenyeva, Irina Nikishina, Valeria Matkava, Olga Kostareva, Aliya Arefieva, Maria Kaleda, Svetlana Salugina, Evgeny Fedorov, Anna Shapovalenko

###### Paediatric, V.A. Nasonova Research Institute of Rheumatology, Moscow, Russian Federation

####### **Correspondence:** Svetlana Arsenyeva

*Pediatric Rheumatology 2024*, **22(2)**: PReS24-ABS-1565

**Introduction:** Despite of wide spectrum of Biologics for the treatment of Juvenile Idiopathic Arthritis (JIA), some subtypes have resistant course which required drug design and development. Unique mechanism of Janus-kinases suggests that tofacitinib (TOFA) can be a useful option for the treatment of various subtypes of JIA.

**Objectives:** To evaluate efficacy and safety of TOFA in children with different subtypes of JIA.

**Methods:** We analyzed data base of 133 patients (pts) who were treated by TOFA in our clinic since 2018 to 2024. All cases of «off-label» TOFA use were approved by Local Ethic Committee.

**Results:** The prospective analysis includes data of 133 pts (45/33% boys; 88/67% girls). Among this cohort we identified 100 pts with different subtypes of JIA. The data includes pts with systemic JIA – 8/8%, RF-positive polyarticular subtype of JIA – 7/7%, enthesitis-related JIA – 9/9%, psoriatic JIA – 8/8%. The largest number of pts had RF-negative polyarticular subtype of JIA – 68/68% (9 was combined with uveitis, 3-with Blau syndrome, 1 – with Chron’s disease, 7 – with chronic recurrent multifocal osteomyelitis (CRMO), 1 – with Down’s syndrome, 2 – with camptodactyly-arthropathy-coxa vara-pericarditis syndrome (CACP), 2 – with type 1 diabetes mellitus). The dosage of TOFA was up to 5 mg twice a day. All JIA pts received methotrexate. The mean duration of TOFA therapy is 10.1 months (from 1 to 36). The majority of TOFA administration was as a first-line treatment (38/38%) and 2-nd line – 34/34%, in 3-d line – 14/14%, 4-th line – 8/8%, 5-th line – 5/5%, 6-th line – 1/1%. Approximately all patient had a good response on TOFA therapy with relief of active arthritis and uveitis, significant reduction of psoriasis skin lesions. Also, we noticed regression of sacroiliitis and coxitis activity by MRI under TOFA therapy. Drug tolerance was good in 98/98% pts. In 1 pt TOFA therapy was withdrawn due to adverse event presented by extended maculopapular skin rash which appeared 3 days after TOFA initiation and 1 pt had allergic conjunctivitis. 13 more withdrawals were due to primary inefficacy (4 pts in 1-st line, 5 – in 2-d line, 3 – in 3-d line, 1- in 4-th line, 2 pt received TOFA as 5-th line therapy).

**Conclusion:** Tofacitinib is highly-efficient and well-tolerated option for management of different subtypes of JIA including refractory forms. Also we noted good response in pts with active uveitis and psoriasis. The further study of the therapeutic potential of JAK-kinase inhibitors in pediatric RD is needed.

**Patient Consent**

Not applicable (there are no patient data)

**Disclosure of Interest**

None Declared

### Treatment

#### P451 Safety of intravenous use of anakinra in pediatric inflammatory conditions: an expansion of the previous case series

##### Marta Klanjscek ^1^, Matteo Trevisan^2^, Serena Pastore^3^, Manuela Pardeo^2^, Alberto Tommasini^3^, Roberta Caorsi^4^, Sarah Abu Rumeileh^5^, Manuela Cortesi^6^, Marco Cattalini^6, 7^, Gabriele Simonini^8, 9, 10^, Marco Gattorno^4^, Fabrizio De Benedetti^2^, Claudia Bracaglia^2^, Andrea Taddio^1, 3^

###### ^1^University of Trieste, Trieste, ^2^Division of Rheumatology, IRCCS Ospedale Pediatrico Bambino Gesù, Roma, ^3^IRCCS Burlo Garofolo, Trieste, ^4^Center for Autoinflammatory Diseases and Immunodeficiencies, IRCCS Giannina Gaslini, Genova, ^5^Reumatology Unit, IRCCS Anna Meyer, Firenze, ^6^Pediatric Clinic, Spedali Civili, ^7^University of Brescia, Brescia, ^8^Rheumatology Unit, IRCCS Anna Meyer, ^9^ERN ReCONNET Center, ^10^NEUROFARBA Department, University of Florence, Firenze, Italy

####### **Correspondence:** Serena Pastore

*Pediatric Rheumatology 2024*, **22(2)**: PReS24-ABS-1486

**Introduction:** Anakinra is a recombinant human interleukin-1 (IL-1) receptor antagonist. It is primarily used by subcutaneous injection in the treatment of several autoinflammatory conditions such as Still disease, familial mediterranean fever and cryopyrin-associated periodic syndromes (CAPS). Intravenous anakinra is used in clinical practice, especially for Macrophage Activation Syndrome (MAS)/Hemophagocytic Lymphohistiocytosis (HLH), despite this being an off-label route of administration. In acute and life-threatening diseases, the subcutaneous route is often problematic, absorption may be unreliable in patients with critical illness, peripheral oedema or anasarca and multiple painful injections are needed to achieve the high doses required. Intravenous administration of anakinra enables a higher and faster maximal plasma concentration to be achieved, compared with subcutaneous delivery. Some authors have already reported its effectiveness via intravenous administration in some specific pediatric clinical settings such as MAS/HLH.

**Objectives:** To collect a greater number of pediatric patients treated by intravenous (IV) anakinra from various Centers to expand our previous research in order to assess its safety.

**Methods:** We retrospectively collect into a database the details of intravenous off-label administration of anakinra in pediatric patients from January 1^st^, 2017, to February 29^th^, 2024. Our study involved IRCCS Maternal and Child Health Institute Burlo Garofolo (Trieste, Italy), IRCCS Ospedale Pediatrico Bambino Gesù (Roma, Italy), IRCCS Giannina Gaslini (Genova, Italy), IRCCS Anna Meyer (Firenze, Italy) and Spedali Civili (Brescia, Italy). Collected data include quantitative characteristics related to drug administration (administered dose, treatment duration, co-treatments), information regarding the hospital setting (intensive care unit admission (ICU)), findings about the clinical response, side effects, their nature and the patient’s outcome (death or alive).

**Results:** Our case series includes 113 patients with different underlying clinical conditions. 21 (18.6%) patients were hospitalized in hematology-oncology division. 59 patients (52.2%) were admitted in ICU. The main diagnoses include MIS-C (30.9%), HLH (21.2%), and MAS (10.6%). The dosage of IV anakinra ranged from 2 to 20 mg/kg/day and the treatment duration from 1 to 80 days. 97 patients (85.8%) experienced an improvement in their underlying condition as assessed by the healthcare provider. Side effects were observed in 10 out of 113 treated children (8.8%), in most cases they were represented by transient elevation of hepatic or pancreatic enzymes (6.2%). Two patients (1.7%) showed a maculopapular rash 20 minutes after the infusion, which resolved upon discontinuation of the medication. In another case (0.01%) there was an immediate reaction with hypotension and vomiting, which resolved after administration of antihistamine. 16 patients (14.1%) experienced an unfavorable outcome due to the progressive and untreatable nature of their underlying clinical condition.

**Conclusion:** The IV use of anakinra in pediatrics could represent a more manageable and effective alternative in the treatment of some life-threatening acute clinical conditions, ensuring the possibility to administer a high dosage of the drug and its absorption even in critical settings. The IV administration of anakinra at a dosage of 2-20mg/kg/day has proven to be safe in our case series. Side effects demonstrate to be transient or pharmacologically manageable. The recorded death cases involved patients who were already in serious condition prior to the administration of anakinra.

**Patient Consent**

Yes, I received consent

**Disclosure of Interest**

None Declared

### Treatment

#### P452 Infectious side effects in patients with JIA treated with rituximab

##### Natalia Kondrateva^1^, Ekaterina Alexeeva ^1, 2^, Tatyana Dvoryakovskaya^1, 2^, Olga Lomakina^1^, Anna Fetisova^1^, Ksenia Isaeva^3^, Aleksandra Chomakhidze^1^, Kristina Chibisova^1^, Ivan Kriulin^1^, Elizaveta Krekhova^1, 2^, Meiri Shingarova^1, 2^, Maria Botova^1^, Tatiana Kriulina^1, 2^, Irina Tsulukiya^1^

###### ^1^Rheumatology, National Medical Research Center of Children's Health, ^2^Sechenov First Moscow State Medical University, ^3^National Medical Research Center of Children's Health, Moscow, Russian Federation

####### **Correspondence:** Ekaterina Alexeeva

*Pediatric Rheumatology 2024*, **22(2)**: PReS24-ABS-1404

**Introduction:** Juvenile idiopathic arthritis (JIA) – is the most common rheumatological disorder in children [1]. Most of patients with JIA respond on monotherapy with DMARDs or their combination with iTNF, IL-6, IL-1, IL-17 blockers and other biologics or JAK inhibitors [2]. Some patients are refractory to all conventional therapies. Rituximab (RTX) is anti-CD20 monoclonal antibody is used like «last-ditch effort» to manage the disease activity in refractory cases. Data of rituximab safety are based on oncology or adult rheumatology studies. It is known that infections are one of the most common side effects of RTX treatment.

**Objectives:** To evaluate quantity and pattern of infectious side effects in children with refractory JIA treated with rituximab.

**Methods:** Monocentric retrospective cohort study of 102 children with established JIA diagnosis according to ILAR criteria who was treated with RTX was performed. Every infectious case was collected and graduated according to Common Terminology Criteria for Adverse Events (CTCAE) v5.0. Also data about neutrophil count, blood immunoglobulin level and other immunosuppressive therapy beside RTX were added.

**Results:** Observational time was 282 patient-years (PY). 162 (57,4 per 100 py) infectious events were registered: grade 3 – 24 cases (8,5 per 100 py); grade 4 – 6 (2 per 100 py), grade 5 – 1 case (interstitial SARS-CoV-2-pneumonia). The most common were pneumonia (45 cases/16 per 100 py, in 11/45 (24%) pneumonia was caused by opportunistic pathogens *- Pneumocystis jirovecii* or/and *Aspergillus spp.*), ENT infections (32 cases/11,3 per 100 py), UTI (22 cases/7,8 per 100 py), less common – URIs (15 cases/5,3 per 100 py), GI infections (10 cases/3,5 per 100 py), skin infections (8 cases/2,8 per 100 py), stomatitis (9 cases/3,2 per 100 py), herpes infections (8 cases/2,8 per 100 py), mucous candidiasis (5 cases/1,8 per 100 py), genital infections (3 cases/1,1 per 100 py), sepsis (3 cases/1,1 per 100 py), 1 case of lung tuberculosis and 1 – purulent gonitis. In 25/102 (24,5%) patients no infection event occurred. More often infectious events were observed during first year after first rituximab infusion (p<0,001). Frequency of infections events inverse correlated with immunoglobulin levels (Ig M – р = 0,01; Ig G – p = 0,009; Ig A – p =0,0007) and directly correlated with dose of oral prednisolone >0,5 mg/kg (p=0,013) and the number of concomitantly used immunosuppressive agents (p<0,001). There was no correlation between neutrophil blood count and infectious events frequency (p>0,05).

**Conclusion:** There are certain infections events which clinician should take account of in JIA patient treated with rituximab. Risk of infection event is higher if patient takes oral prednisone more than 0,5 mg/kg/day or other immunosuppressive drugs beside RTX; if patient has low Ig level.

**Date of birth::** juillet 19

**Patient Consent**

Yes, I received consent

**Disclosure of Interest**

None Declared

**References**

Zaripova LN, Midgley A, Christmas SE, Beresford MW, Baildam EM, Oldershaw RA (2021) Juvenile idiopathic arthritis: from aetiopathogenesis to therapeutic approaches. Pediatr Rheumatol 19:135
2.Onel KB, Horton DB, Lovell DJ, et al (2022) 2021 American College of Rheumatology Guideline for the Treatment of Juvenile Idiopathic Arthritis: Therapeutic Approaches for Oligoarthritis, Temporomandibular Joint Arthritis, and Systemic Juvenile Idiopathic Arthritis. Arthritis Care & Research 74:521–537

### Treatment

#### P453 Tocilizumab experience in juvenile scleroderma at a single tertiary center in Saudi Arabia

##### Munira Al Marri, Deena Alwakeel, Abdulrahman Asiri, Abdulaziz Alrowais

###### Pediatrics, Prince Sultan Military Medical City, Riyadh, Saudi Arabia

####### **Correspondence:** Munira Al Marri

*Pediatric Rheumatology 2024*, **22(2)**: PReS24-ABS-1327

**Introduction:** Scleroderma is an autoimmune disease that may result in changes to the skin, blood vessels, muscles, and internal organs. It can be either Localized scleroderma (LS) or Systemic Sclerosis (SSc). Early diagnosis and effective treatment are crucial to improve the long-term outcome. Tocilizumab (TCZ) is an anti-IL-6 receptor-α antibody which approved for the treatment of inflammatory disorders, including rheumatoid arthritis, systemic and polyarticular juvenile idiopathic arthritis, and giant cell arteritis. The use of TCZ in other pediatric rheumatic diseases as scleroderma is not yet well studied although there are several reports and case series with promising results in this matter. The current analysis is from a single center in Saudi Arabia to investigate the long-term efficacy and safety of TCZ in patients with SSc and LS.

**Objectives:** This study aims to evaluate the efficacy and safety of tocilizumab (TCZ) as a treatment option for juvenile LS and SSc.

**Methods:** This is a retrospective chart review for all the patients who receive TCZ for scleroderma in a single center at Prince Sultan Military Medical City in Saudi Arabia. Overall, six patients were included in the study (3 Females and 3 males with a mean age of 9 years (range 6-12 years). The modified Rodnan skin score (mRSS), carbon-monoxide diffusion capacity (DLCO), thorax high-resolution tomography (HRCT), patient global assessment (PGA), and Juvenile Systemic Sclerosis Severity (J4S) score were used in 3 patients with SSc as endpoints. The Localized Scleroderma Cutaneous Assessment Tool (LoSCAT) and physician global assessment of activity and damage (PGA-A and PGA-D) were used in 3 patients with LS patients as endpoints to evaluate TCZ treatment response at 12 months post-treatment.

**Results:** Six patients with scleroderma were treated with TCZ, in addition to conventional treatment. The median age at the disease onset is 8.3 years (5–11.5) and at the diagnosis is 9 years (6-12). Three JSS patients with interstitial lung disease were treated with TCZ with a median treatment duration of 19 months (14-24). All 3 patients had radiologically confirmed improvement in thorax HRCT at 12 months post-treatment. They had decreased PGA (mean pre-treatment PGA 5 vs. 2.6 post-treatment PGA at 12 months). They had increased DLCO (mean pre-treatment DLCO 47% vs.12 months post-treatment DLCO 72.6%). In all 3 patients, mRSS and the J4S decreased: 33.3 vs.14.3 and 10 vs. 4.3, respectively. Three LS patients were treated with TCZ with a median treatment duration of 22 months (6 -48). They had decreased PGA-A and PGA-D with mean pre-treatment PGA-A 6.3 vs 1.5 in 12 months post-treatment while the mean pre-treatment PGA-D 6 vs. 4 in 12 months post-treatment. They had decreased in LoSSI and LoSDI: 25 vs. 2 and 20 vs.16 respectively. All 6 patients tolerated well TCZ treatment without side effects.

**Conclusion:** Tocilizumab is considered an effective treatment for patients unresponsive to conventional treatment in systemic sclerosis and refractory severe localized scleroderma. Long-term studies with a bigger number of patients are needed to provide more relevant data.

**Patient Consent**

Not applicable (there are no patient data)

**Disclosure of Interest**

None Declared

**References**

1. Adrovic, A., Yildiz, M., Haslak, F. *et al.*Tocilizumab therapy in juvenile systemic sclerosis: a retrospective single-center pilot study. *Rheumatol Int* 41, 121–128 (2021).
2. Lythgoe H, Baildam E, Beresford MW, Cleary G, McCann LJ, Pain CE (2018) Tocilizumab as a potential therapeutic option for children with severe, refractory juvenile localized scleroderma. Rheumatology 57:398–401.
3. Martini G, Campus S, Raffeiner B, Boscarol G, Meneghel A, Zulian F (2017) Tocilizumab in two children with pansclerotic morphea: a hopeful therapy for refractory cases? Clin Expert Rheumatol 35:211–213.

### Treatment

#### P454 Combination therapy with palovarotene and tofacitinib in patients with fibrodysplasia ossificans progressiva: hopeful promising for a hopeless disease

##### Irina Nikishina^1^, Valeria Matkava^1^, Svetlana Arsenyeva^1^, Aliya Arefieva^1^, Leonid Blank^2^, Emil Gasymov^2^

###### ^1^Paediatric, ^2^Radiology, V.A. Nasonova Research Institute of Rheumatology, Moscow, Russian Federation

####### **Correspondence:** Irina Nikishina

*Pediatric Rheumatology 2024*, **22(2)**: PReS24-ABS-1505

**Introduction:** Fibrodysplasia ossificans progressiva (FOP) is ultra-rare severe genetic disorder characterized by progressive heterotopic ossification (HO) and certain rheumatological symptoms such as synovitis, sacroiliitis, spondylitis. Current treatment options for FOP are limited to symptomatic management. In initial stage of new bone formation inflammation plays important role so our previous experience shown good results of tofacitinib (TOFA) use in FOP patients^1^. New promising therapeutic agent for HO prevention is palovarotene (PALO), a selective retinoic acid receptor gamma agonist. It affects the processes of chondrogenesis and it is hoped that the effect of PALO will complement the anti-inflammatory effect of TOFA.

**Objectives:** To describe the first experience of combination therapy with PALO added to TOFA in severe course of FOP in open study based on real clinical practice.

**Methods:** In 10 patients (pts) with severe course of FOP who previously received TOFA in standard dose regimen, PALO was added to therapy. All pts signed informed consent and met eligibility criteria(genetically verified FOP;female>8y.o.,male>10y.o.). PALO was taken according official instructions–in chronic or flare-up regimens. Low-dose whole-body CT(WBCT) was done for evaluate HO volume.

**Results:** All 10 pts have classic FOP phenotype with malformed great toes (10/100%), shortened thumbs (8/80%), abnormalities of cervical spine, peripheral osteochondromas and multiple HO in 10/100% pts. Diagnosis was confirmed by genetic test (ACVR1) in 10/100% pts and revealed typical (p.Arg206His) in 9 pts and 1 ultra-rare(p.Gly356Asp). The median age was 13,2 y.o. (min–10.2;max–17.9). 7 pts were < 14 years. Me height was 155 cm(min-129; max–180). Me weight was 45 kg(min-25;max–65). By present time 7 pts received PALO (combinated with TOFA), for other 3 pts PALO have just been administered. The median therapy duration is 9 months (from 5 to 12 mo). 3/7 pts used only chronic regimen(3-5 mg per day depends on weight) and 4/7 pts used additionally flare-up regimen(12.5-20 mg per day for 4 weeks). All pts had different intensity adverse reactions connected with skin and depended on the PALO dosage. 3 pts on chronic regimen had mild skin reactions such as dry skin, dry lip and short-duration pruritus. Other 4 pts had more severe skin damage(on flare-up regimen) such as rash, erythema and skin exfoliation with significant pruritus. When we reduce the dosage of PALO all skin symptoms relieved. There were no any serious adverse events. It difficult to evaluate efficacy due to short period of treatment. For assessment of HO volume we performed WBCT in all pts. In 2 pts it was done with 1-year period and HO progression were not revealed.

**Conclusion:** Palovarotene is well-tolerated option for treatment of FOP pts. Furthermore, safety profile and oral administration make palovarotene an attractive candidate for long-term treatment. Сomplementary effect of palovarotene and TOFA seems to be new strategy but need further investigations.

**Patient Consent**

Not applicable (there are no patient data)

**Disclosure of Interest**

None Declared

**Reference**

1. Nikishina,I et al.. Successful experience of tofacitinib treatment in patients with Fibrodysplasia Ossificans Progressiva. Pediatr Rheumatol Online J. 2023 Aug 29;21(1):92. doi: 10.1186/s12969-023-00856-1. PMID: 37644581; PMCID: PMC10464034.

### Treatment

#### P455 Safety and efficacy of combination therapy with adalimumab and anakinra in two patients with overlapping inflammatory diseases

##### Simone Coslovich, Serena Pastore, Daniela Chicco, Matteo Bramuzzo, Sara Lega, Alberto Tommasini, Andrea Taddio

###### IRCCS Burlo Garofolo, Trieste, Italy

####### **Correspondence:** Serena Pastore

*Pediatric Rheumatology 2024*, **22(2)**: PReS24-ABS-1783

**Introduction:** Chronic inflammatory diseases can be associated with other simultaneous inflammatory conditions that worsen the overall clinical situation making therapeutic management challenging.For example, 3 to 6% of Familial Mediterranean fever (FMF) cases may be associated with Juvenile idiopathic arthritis (JIA) and uveitis^1-2,^ while recurrent pericarditis can be an extra-intestinal manifestation of ulcerative colitis (UC)^3^.It may happen that every inflammatory condition proceeds independently one from each other, requiring specific therapy.Biologic drugs are increasingly used in the treatment of these conditions and there are limited data regarding a combination therapy.

**Objectives:** To describe the safety and efficacy of combination therapy with adalimumab and anakinra in two patients with overlapping inflammatory diseases

**Methods:** Case reports

**Results:** A 15-year-old girl on adalimumab therapy (40 mg every 15 days) for UC was diagnosed with recurrent pericarditis.Corticosteroids proved to be the only effective treatment, with each attempt to discontinue the medication resulting in pericarditis relapse.To achieve better disease control and minimize the risk of side effects, the decision was made to start treatment with anakinra (100 mg daily).Six months after starting anakinra, the patient did not experience any further episodes of pericarditis.An 11-year-old boy with JIA-associated uveitis on adalimumab therapy was diagnosed with FMF, after an history of arthralgias, chest pain, abdominal pain and persistently elevated indices of inflammation. The patient showed poor response and tolerance to colchicine, so we decided to initiate therapy with anakinra (100 mg daily) with good control of disease.

In both cases, the combination therapy was well-tolerated with no documented side effects at follow-up of 4 months for both cases.

**Conclusion:** We describe our experience of combined use of adalimumab and anakinra.There are limited studies concerning combination therapy of IL-1 and TNFalpha inhibitors.One study combined these two drug classes (in adults with rheumatoid arthritis) with no added benefit and an increased incidence of infections^4.^

In our cases combination therapy proved to be effective and lacking of short-term immunosuppressive effects, in addition allowed to achieve better disease control.

**Patient Consent**

Yes, I received consent

**Disclosure of Interest**

None Declared

**References**

1. 1Özçakar ZB et al. Familial Mediterranean fever-associated diseases in children. QJM. 2017 May 1;110(5):287-290
2. 2Yildiz M et al. Evaluation of co-existing diseases in children with familial Mediterranean fever. Rheumatol Int. 2020 Jan;40(1):57-64
3. 3Mitchell NE et al. Heart Under Attack: Cardiac Manifestations of Inflammatory Bowel Disease. Inflamm Bowel Dis. 2018 Oct 12;24(11):2322-2326
4. 4Genovese MC et al. Combination therapy with etanercept and anakinra in the treatment of patients with rheumatoid arthritis who have been treated unsuccessfully with methotrexate. Arthritis Rheum. 2004 May;50(5):1412-9

### Treatment

#### P456 Efficacy of combined bmards therapy in treatment-resistant juvenile idiopathic arthritis (jia) and dermatomyositis (dm): a case series

##### Agnieszka Gazda^1^, Beata M. Kołodziejczyk^1^, Piotr Gietka^2^, Olga Krasowicz-Towalska^1^, Joanna Królikowska^1^, Dominika Labochka-Chyżyńska^1^, Iryna Naishtetik ^1^, Iwona Witkowska^1^, Khrystyna Rybak^1^

###### ^1^Developmental Age Rheumatology Clinic, ^2^ Developmental Age Rheumatology Clinic, National Institute of Geriatrics, Rheumatology and Rehabilitation, Warsaw, Poland

####### **Correspondence:** Agnieszka Gazda

*Pediatric Rheumatology 2024*, **22(2)**: PReS24-ABS-1240

**Introduction:** Management of severe JIA and DM challenges clinicians, especially in the cases with resistance to standard therapies. This study explores the outcomes of combination treatment using two biological drugs.

**Objectives:** The aim of the study was to assess the effectiveness and safety of combined treatment with two biological drugs.

**Methods:** We retrospectively reviewed 2 pediatric patients diagnosed with severe JIA and 1 patient with DM unresponsive to conventional treatments, including DMARDs and bMARDs. The combinations employed were adalimumab (ADA) with tofacitinib in two patients and secukinumab with tofacitinib in one patient. The effects of these combinations were monitored by clinical and disease activity markers assessment.

**Results:** A 15-year-old girl with enthesitis-related arthritis JIA. The onset in the age of 3 years- inflammation of multiple joints, the course of the disease with treatment-resistant inflammation of the right knee joint, right ankle and right foot. In the treatment MTX, GCs oral and i.a.,CsA, sulpha, etanercept, tocilizumab, ADA were used. Isotope synovectomy and surgical synovectomy of the right knee joint were performed. On MTX and ADA therapy, acute anterior uveitis of the right eye occurred for the first time. Tofacitinib was added to MTX and ADA treatment. A significant reduction in inflammatory changes in the anterior uvea of the right eye and inflamed joints was observed.

A 13-year-old boy with with enthesitis-related arthritis JIA diagnosed at the age of 5 months.The onset and course of JIA with treatment-resistant inflammation of peripheral polyarthritis, then with bilateral sacroiliitis, arthritis of the cervical and thoracic spine and with persistently elevated inflammatory parameters. In the treatment were used: MTX, GKS oral and i.a.,etanercept, tocilizumab, ADA, secukinumab. Despite treatment modifications, a high disease activity persisted. Tofacitinib was added to the treatment with secukinumab, MTX and prednisone. Joints' pain and morning stiffness were reduced, inflammation of many joints still persists, in laboratory tests moderately elevated inflammatory parameters, and ultrasound examinations of the joints show active inflammatory lesions.

A 12-year-old girl with juvenile DM, diagnosed at the age of 6. In the treatment of DM were used: GCs oral and i.v, HCQ, MTX, IVIG, CsA - in combination with MTX, MMF, aza, ADA. Despite treatment modifications, there was no improvement in the skin lesions, muscle weakness and calcinosis with myocardial calcinosis appered. Combined treatment: tofacitinib, ADA, and zolendronic acid was used. A slight improvement in skin lesions and muscle strength was achieved.

**Conclusion:** The combination therapy was well tolerated in all patiens. No serious adverse effects, no serious infections, no episodes of thrombosis were reported during the observation period (average follow-up time- 11 months).In 2 patients, transient, mild anemia occurred, and a single neutropenia in 1 patient- 1.31 x 10^3/uL. Moreover, no leuko-, lympho- or neutropenia was observed. Liver and kidney function parameters were normal in all patients.Dual biological therapy may offer an alternative for patients with severe JIA and DM who do not respond adequately to standard treatment.

**Patient Consent**

Yes, I received consent

**Disclosure of Interest**

None Declared

### Treatment

#### P457 An experience with the use of biologic dmards in rheumatic diseases at a tertiary hospital in Cape Town, South Africa

##### Hanna Kassa^1^, Claire Butters ^1^, Helen Crichton^1^, Kate Webb^1^

###### ^1^Paediatrics, University of Cape Town, Cape Town, South Africa

####### **Correspondence:** Claire Butters

*Pediatric Rheumatology 2024*, **22(2)**: PReS24-ABS-1764

**Introduction:** Biologic disease modifying anti-rheumatic drugs (bDMARDs) have revolutionized the treatment of pediatric rheumatic diseases (PRD) including juvenile idiopathic arthritis ( JIA), offering improved control of symptoms and enhanced long-term outcomes. Access to these drugs is extremely limited across Africa due to concerns about affordability and safety, despite scarce data describing the use of bDMARDs in children from Africa.

**Objectives:** To describe use of bDMARDs in children with PRD in a tertiary hospital in South Africa.

**Methods:** A retrospective cross-sectional folder review of children on continuous bDMARD therapy at the state funded Red Cross War Memorial Children’s Hospital in Cape Town between January 2020 and July 2023 was performed. Demographics, details of disease and treatment outcomes were analysed.

**Results:** Forty patients received bDMARDs. Twelve (30%) were male, and the median duration from diagnosis to start of biologic was 408 days, with the median age at the start of biologic at 7,64 years. Disease profile: 9/40 (23%) - systemic JIA; 6/40 (15%) - polyarticular RF- JIA; 5 (13%) - isolated uveitis; 4 (10%) - ERA, 4 (10%) - undifferentiated JIA; 3 (8%) – psoriatic JIA; 3 (8%) - oligoarticular persistent JIA; 1 (3%) - polyarticular RF+ JIA; 1 (3%) - oligoarticular extended JIA. Four children had other diagnoses, including a misdiagnosis of mucopolysaccharidosis, 2 with undefined autoinflammation and one with polyarteritis nodosum.

Before receiving a biologic, the following DMARDs were used: 17 (43%) oral steroids, 17 (43%) oral and IV steroids and 2 (5%) intra-articular steroids only. Thirty-seven (93%) received methotrexate, and 19 (47%) received additional non biological DMARDs. The median number of active joints before biologic, 3 months after and 6 months after were 4 (n=31; IQR 0, 7), 0 (n=29; IQR 0, 2) and 0 (n=31; IQR 0, 1) respectively. The median cJADAS score before receiving a biologic, 3 months after and 6 months after was 4.3 (n=19; IQR 3; 5.3), 0.5 (n=18; IQR 0; 2) and 0.3 (n=17; IQR 0; 1.3) respectively.

Seven patients required switching to a second bDMARD, and 5 switched to a third bDMARD. The bDMARDs received included adalimumab (n=26; 65%), tocilizumab (n=13, 33%), etanercept (n=4; 10%), infliximab (n=4; 10%), secukinumab (n=2; 5%), rituximab (n=1; 3%), anakinra (n=1; 3%) and golimumab (n=1; 3%). The reasons for switching included disease flare, bDMARD failure, and bDMARD side effects. These side effects included infections, liver injury, MAS, rash and hypersensitivity.

**Conclusion:** Children in Africa with PRD deserve access to bDMARD therapy. This is the only data documenting the routine and appropriate use of these drugs in this setting. This provides a resource to advocate for improved access, decreased cost and increased awareness among doctors, funders and policy makers on the continent.

**Date of birth::** mai 07, YY

**Patient Consent**

Not applicable (there are no patient data)

**Disclosure of Interest**

None Declared

### Treatment

#### P458 The drug rash with eosinophilia and systemic symptoms (Dress) syndrome in a 16-year-old patient with idiopathic Juvenile arthritis (JIA) triggered by salazopyrine

##### Martin L. Boesen^1^, Anne E. Christensen^1^, Peter Toftedal^1^

###### ^1^H C Andersen Children Hospital, Odense University Hospital, Odense, Denmark

####### **Correspondence:** Martin L. Boesen

*Pediatric Rheumatology 2024*, **22(2)**: PReS24-ABS-1595

**Introduction:** DRESS syndrome is a rare but serious complication to a variety of drugs. Symptoms of DRESS syndrome includes fever, cutaneous and mucosal involvement, lymphadenopathy, visceral organ involvement especially liver but also renal, cardiac and pulmonary involvement. The syndrome is characterized by hematologic abnormalities (eosinophilia, mononucleosis-like atypical lymphocytes), and viral reactivation. The symptoms develop 2-8 weeks after exposure to the drug.

**Objectives:** A case of DRESS syndrome in a 16-year-old girl diagnosed with JIA triggered by Salazopyrine is presented to increase the awareness of the syndrome.

**Methods:** Case-report

**Results:** Salazopyrine was prescribed to a 16-year-old girl with JIA, due to former adverse reaction to methotrexate (nausea). Symptoms of DRESS syndrome arose 28 days after initializing Salazopyrine. Initial symptoms consisted of mild fever and maculo-papular rash primarily located in the face mimicking a mild virus infection. She developed edema of the face and on the extremities. Salazopyrine treatment was suspended 6 days after the initial symptoms in suspicion of drug reaction, but 6 weeks after initial Salazopyrine treatment she was admitted to hospital with a generalized maculopapular rash, oral blisters, lymphadenopathy, and generalized edema. The skin was warm, highly red and itching. Tonsils were red and swollen with lymphadenopathy located on the neck, in the axilla- and the inguinal regions. Mild gastrointestinal involvement in form of colitis was observed. She presented with leukocytosis (21,9x10^9^/L), atypical lymphocytosis, eosinophilia (3.17x10^9^/L) and increased alanine aminotransferase (ALT) 191 U/L and gamma-glutamyl transferase (GGT) 122 U/L. Reactivation of herpes virus infection was not seen. A chest radiograph showed minimal pleural effusion with no other pulmonary involvement.

At time of admittance treatment with prednisolone 1 mg/kg was initiated. Despite this, eosinophilia peaked at 8.61x10^9^/L 3 days later. After 9 days of prednisolone treatment the rash faded, edema degraded, and her well-being improved. Only 3 days later ALT rose 5-fold to 1600 U/L along with worsening of edema and rash. High-dose intravenous immunoglobulin (IVIG) (2 g/kg/day for 3 consecutive days) was initiated resulting in a response in symptoms and liver enzymes.

**Conclusion:** DRESS syndrome should be supposed when a child presents with fever, maculopapular eruption, lymphadenopathy, eosinophilia, and visceral involvement. Immediately suspension of the drug is essential. This case shows a seldom but classic example of DRESS syndrome as an adverse drug reaction to Salazopyrine.

**Patient Consent**

Yes, I received consent

**Disclosure of Interest**

None Declared

### Treatment

#### P459 Treatment of juvenile idiopathic arthritis with monoclonal antibody in child with concommitant alpha-1-antitripsin deficiency, hashimoto thyroiditis and nonalcoholic fatty liver disease

##### Velma Selmanovic^1^, Adisa Cengic^2^, Samra Rahmanovic^3^, Snijezana Hasanbegovic^4^, Delfina Skender^5^, Amila Hadzimuratovic^1^

###### ^1^Allergology, Rheumatology and Clinical Immunology, Children's Hospital university Clinical Center Sarajevo, ^2^Allergology, Rheumatology and Clinical Immunology, Children's Hospital University Clinical Center, ^3^Gastroenterohepatology, ^4^Endocrinology, Children's hospital University Clinical Center Sarajevo, Sarajevo, ^5^Rheumatollogy and Immunology, Children's Hospital University Clinical Center Mostar, Mostar, Bosnia and Herzegovina

####### **Correspondence:** Velma Selmanovic

*Pediatric Rheumatology 2024*, **22(2)**: PReS24-ABS-1609

**Introduction:** Juvenile idiopathic arthritis (JIA) is one of most common chronic diseases in childhood. It is extremely rarely accompanied with alpha-1-antitrypsin deficiency (AAD). AAD is the most common genetic cause of pediatric liver disease and the most frequent inherited indication for liver transplantation in children. Individuals who are homozygous for the mutant Z allele (PiZZ) are at risk for the development of liver disease. Alpha-1-antitripsin is a serine proteinase inhibitor, synthesized primarily in hepatocytes, with primary function to protect normal body tissue from damage by nonspecific neutrophil proteolytic enzymes, such as neutrophile elastase (can attack lung elastin resulting in emphysema).

**Objectives:** to decribe clinical characteristics, disease history and treatment response in 13y girl with JIA and AAD (PiZZ) genotype.

**Methods:** case report

**Results:** Girl,13y, March 2022, presented with extensive symmetrical polyarthritis. Arthritis onsed was 1,5mo earlier while COVID-19. Her BMI was 25,2 (overweigh).Laboratory showed slightly raised ESR, normal CBC and CRP, raised AST, ALT, hypergammaglobulinemia,raised IgG, ANA+, HLA-B27 and RF-negative, stool calprotectin twice negative, autoimmune hepatitis antibodies negative. History: transitory neonatal jaundice, age 3y transitory elevation of transaminases; at age 5y diagnosed as profound Hashimoto thyroiditis (HT) treated accordingly. Usuall treatement with ibuprofen resulted in mild hipertransaminasemia. One low dose of metil-prednisolon also resulted in raised transamnases. Clear evidence of drug induced liver injury. Extensive gastroenterology work up detected low level of alpha-1-antitripsin enzyme, PiZZ genotype, and liver biopsy performed in June 2022 revealed nonalkoholic fatty liver disease (NAFLD). Autoimmune hepatitis was excluded. Etanercept was successfully introduced in November 2022. She is followed up for 18mo till now : no signs of active arthritis or liver injury.

**Conclusion:** we presented successfull treatement of JIA with etanercept in a child with concomitant AAD, Hasimoto thyroiditis, NAFLD. Multidisciplinary team approach is required to successful treatment of such challenging aptient : rheumatologist, gastroenterohepatologist, endocrinologist and clinical pharmacologist. There is potential association of AAD, JIA and HT involving both autoimmunity and neutrophilic inflammatory process.

**Patient Consent**

Yes, I received consent

**Disclosure of Interest**

None Declared

### Treatment

#### P460 Zoledronic acid: an effective treatment in chronic non-bacterial osteomyelitis

##### Kyveli Chiotopoulou^1^, Katerina Kourtesi^2^, Spyridon Proutzos^3^, Eftuxia Kostoula^1^, Elli Athanasopoulou^1^, Erato Atsali^2^, Olympia Papakonstantinou^3^, Lampros Fotis^2^

###### ^1^Department of Pediatrics; ^2^Department of Pediatrics, Division of Pediatric Rheumatology; ^3^Department of Radiology, ATTIKON General Hospital, National and Kapodistrian University of Athens, Athens, Greece

####### **Correspondence:** Lampros Fotis

*Pediatric Rheumatology 2024*, **22(2)**: PReS24-ABS-1268

**Introduction:** The treatment goal in chronic non-bacterial osteomyelitis (CNO) is to relieve pain and prevent disease progression, aiming to mitigate permanent damage in the affected bones. First-line treatment often consists of non-steroidal anti-inflammatory drugs (NSAIDs), either alone or in combination with steroid therapy. According to consensus treatment plans, second and third-line medications include disease-modifying antirheumatic drugs (DMARDs), bisphosphonates, and biologic DMARDs. Zoledronic Acid (ZA) is a third-generation bisphosphonate and is considered an acceptable treatment option.

**Objectives:** To delineate the clinical profile and prognostic outcomes of pediatric patients diagnosed with CNO, particularly those exhibiting partial or inadequate response to other therapies, and subsequently managed with ZA.

**Methods:** The medical records of children with CNO from May 2017 to March 2024 were retrospectively reviewed. The diagnosis of CNO for all children was based on the 'Bristol criteria.' Descriptive statistics were utilized to describe the population and assess the treatment effects of ZA. The dosing regimen for ZA was 0.0125-0.025-0.05 mg/kg at 0-3-6 months, respectively, followed by 0.05 mg/kg thereafter.

**Results:** Forty-three children (22 females and 21 males) with a mean age of 10.5 years (6-16 years) participated in the study. Whole-body MRI (WBMRI) at the time of diagnosis revealed multiple bone lesions in all but 5 patients (mean number: 8 lesions). Six children presented with pauci-focal disease (2-4 lesions), while 32 patients (74.4%) had multifocal bone involvement. Thirty-eight patients received treatment for CNO, while 5 were lost to follow-up. Nine patients underwent treatment with ZA.

Three patients received ZA, as 2^nd^ line treatment following NSAIDs, while for 6 it was selected as a third-line treatment. The average treatment period of ZA was 13.6 months (median: 6) with an average of 2.2 infusions (median: 2) of ZA. At diagnosis, 8 out of 9 patients reported bone pain. At the final visit, 2 patients reported no pain, and 6 reported a significant reduction in pain. Following treatment with ZA, etanercept was administered concomitantly in 2 cases, and adalimumab in 1 case, as patients had vertebral fractures, while 1 patient continued therapy with Ustekinumab to treat concomitant psoriasis. All 9 patients received a 3-day course of methylprednisolone following the infusion to minimize flu-like symptoms. The treatment response was also noteworthy. In most cases, follow-up WB-MRI one year post-diagnosis is pending.

**Conclusion:** In this study, patients exhibited a positive response to treatment with ZA, as evidenced by the reduction in pain reported at their last visit. Thus, the use of ZA appears to enhance the quality of life for these patients, representing a significant advancement in the treatment of CNO, with minimal adverse events.

**Trial registration identifying number:**

**Patient Consent**

Yes, I received consent

**Disclosure of Interest**

None Declared

**References**

1. Sergi CM, Miller E, Demellawy DE et al. Chronic recurrent multifocal osteomyelitis. A narrative and Pictorial Review. Frontiers in Immunology. 2022 Aug 22;13. doi: 10.3389/fimmu.2022.959575
2. González Vázquez E, Martín Pedraz L, et al. Is zoledronate a safe and effective treatment option in chronic nonbacterial osteomyelitis? Anales de Pediatría (English Edition). 2023 Feb;98(2):142–5. doi: 10.1016/j.anpede.2022.09.011

### Treatment

#### P461 A rare phenotype of histiocytosis lymphadenopathy plus syndrome: new therapeutic approach

##### A. Amodio^1^, C. Grella^1^, M. Lupia^2^, S. Scianguetta^1^, I. Tartaglione^1^, M. Casale^1^, F. Rossi^1^, A. Zanfardino^1^, D. Rana^1^, A.N. Olivieri^1^, S. Perrotta^1^, D. Roberti^1^, M.F. Gicchino^1^

###### ^1^University of the Study of Campania L.Vanvitelli, Naples, ^2^IRCCS Istituto Giannina Gaslini, Genoa, Italy

####### **Correspondence:** A. Amodio

*Pediatric Rheumatology 2024*, **22(2)**: PReS24-ABS-1313

**Introduction:** "Histiocytosis Lymphadenopathy plus” syndromes are a group of autosomal recessive disorders characterized by lympho-hyperproliferation, histiocytes infiltration and overexpression of inflammatory cytokines. They are caused by LOF mutations of the *SLC29A3* gene encoding the human Equilibrative Nucleoside transporter 3 (ENT3), a member of a group of transmembrane carriers. Lack of ENT3 leads to an impairment in macrophage functions, and a disruption of histocyte proliferation and functions, of mitochondrial activity, and lysosomal nucleoside buildup, also causing an over activation of the inflammasome pathway.

**Objectives:** To describe a case of 16-year-old patient with a new mutation of the SLC29A3 gene.

**Methods:** Patient is currently cared for her disease by our Pediatric Hematology Unit, she is a 16-year-old girl homozygous carrier of a new mutation of the *SLC29A3* gene (NM_018344: c.914C>G).

Since young age, she manifested a pleomorphic clinical picture with hematological, autoimmune, cardiorespiratory and metabolic involvement.

She has a severe transfusion-dependent hypo regenerative anemia with secondary hemosiderosis; dilated cardiomyopathy treated with beta blockers; chronic latent respiratory failure upon physical effort and associated reduced alveolar-capillary diffusion capacity; insulin-dependent diabetes mellitus currently treated with insulin pump therapy; autoimmune polyarticular arthritis treated with oral low dose prednisone and subcutaneous methotrexate; macular degeneration Stargardt-like treated with Lutein and polyunsaturated fatty acids.

These symptoms showed fluctuation among clinical and laboratory improvements and flare ups.

We assessed peripheral INF signature and both innate- and adaptive- cytokines levels.

In view of the results described below, to modulate the inflammatory pathways involved in the disease, a Jak inhibitor was administered since January 2024. Based on the selectivity and current regulatory approvals, Tofacitinib, a JAK1/3 and partial JAK2 inhibitor, was selected. Patient started with a low dose (5 mg), increased to the full daily dose (10 mg), after 4 weeks. Meanwhile she continued her baseline therapy, and she started minor decalage prednisone (from 5 mg/die to 2.5 mg/die).

**Results:** Cytokine testing on peripheral blood and bone marrow revealed an important rise of IL-2R, IL-6, IL-18, and sFAS ligand in both compartments, whereas peripheral interferon signature was negative. However, the most interesting finding, was related to IL-10 levels, highly elevated in bone marrow compartment (22 pg/mL) and unmeasurable in peripheral blood, implying for a compartmental restriction of the immune de-regulation. Therapy with Tofacitinib caused a remarkable sustained improvement in metabolic and hematologic features. Indeed, glycemic control improved from needing insulin dose that never dropped below 1 U/kg/day to half of it (0.5 U/kg/day) with an on-treatment glycemic normal range that went from 70 to 90%. Moreover, patient showed a marked average decrease in hemoglobin consumption that was reduced by approximately 4 times, going from 0.28 g/dL/day to 0.08 g/dL/day.

**Conclusion:** Targeted therapies must consider target and off-target selectivity, mostly in ultrarare, “hard-to treat and understand” complex diseases. In our report, use of Tofacitinib improved patient's metabolic and hematological features likely due to an immune modulation of a compartmental deregulation. A long-term assessment of benefits and tolerability will be required.

Taken together, our report suggests that selective JAK inhibitors could represent a possible treatment for the underlying inflammatory mechanisms of this rare disorder.

**Patient Consent**

Yes, I received consent

**Disclosure of Interest**

None Declared

### Treatment

#### P462 Biologic therapies for Juvenile idiopathic arthritis in costa rica: a 10-year experience

##### Constanza Chacon-Gonzalez ^1^, Gabriela Ivankovich-Escoto^2^, Oscar Porras-Madrigal^2^

###### ^1^Pediatrics, ^2^Immunology and Rheumatology, Hospital Nacional de Niños "Dr. Carlos Sáenz Herrera", San Jose, Costa Rica

####### **Correspondence:** Constanza Chacon-Gonzalez

*Pediatric Rheumatology 2024*, **22(2)**: PReS24-ABS-1533

**Introduction:** Nowadays biologic drugs are a mainstay in the treatment of juvenile idiopathic arthritis (JIA). An opportune use of these drugs early in the diagnosis allows controlling disease activity, avoiding long-term sequeale, and improving the quality of life of patients. We describe the main characteristics of our cohort of JIA patients treated with biologics.

**Objectives:** To describe the main characteristics of patients and biological treatments received during the last 10 years (2014–2024) in the pediatric population with JIA in a single center in Costa Rica.

**Methods:** A retrospective descriptive analytical study of patients diagnosed with JIA, as proposed by ILAR, receiving biological treatment from 1 January 2014 to April 2024. Data was recorded from electronic medical records of patients received biological treatment. Statistical analysis performed.

**Results:** A total of 38 patients were included in the cohort, 28 (74%) were female, 10 (26%) were male. Mean age of diagnosis was 7.1 years (SD±3.39 years). The distribution of JIA subtypes was: polyarticular FR negative 39% (15 patients), polyarticular FR positive 21% (8 patients), oligoarticular 13% (5 patients), systemic 10.5% (4 patients), enthesitis-related 10.5% (4 patients), psoriatic 2.5% (1 patient), and unspecified 2.5% (1 patient).

Patients with positive antinuclear antibodies (ANA) represent 21% (8 patients). Only 3 (7.9%) patients had uveitis, and only 1 of the 3 (33%) patients with uveitis had a positive ANA.

All patients had received methotrexate either orally or subcutaneously prior to starting biologic therapy. All patients continued on methotrexate if using an anti-TNF biologic.

A total of 43 biologics were prescribed. Etanercept represented 51% of prescriptions, tocilizumab 37%, and adalimumab 12%.

Six patients (16%) required a change in biologic therapy.

No adverse effects or deaths related to the use of biologic treatment were recorded.

**Conclusion:** Many clinical features in this cohort coincide with the literature. However, unlike most countries, the most common subtype was polyarticular JIA. It is uncommon to see uveitis and ANA positivity in the Costa Rican population.

The biological treatment most often used was etanercept, particularly in non-systemic JIA, while tocilizumab was used in all patients with systemic JIA and in many non-systemic JIA that failed to respond to etanercept. Adalimumab was most often used in enthesitis-related JIA.

Biological treatments appear to be safe, as no adverse effects or deaths were reported in our cohort.

**Trial registration identifying number:** CEC-HNN-005-2024

**Date of birth::** octobre 29

**Patient Consent**

Not applicable (there are no patient data)

**Disclosure of Interest**

None Declared

### Treatment

#### P463 The national Arthritis Research Coalition (ARC) Biobank- the first 100 paediatric patients to date

##### Karolina Warciak ^1^, Emma O'Brien^2^, Gerry Wilson^3^, Orla G. Killeen^1^

###### ^1^Rheumatology, National Centre for Paediatric Rheumatology, CHI at Crumlin; ^2^Research Centre, National Children's Research Centre, CHI at Crumlin; ^3^Centre for Arthritis Research, Centre for Arthritis Research, Conway Institute, University College Dublin, Dublin, Ireland

####### **Correspondence:** Karolina Warciak

*Pediatric Rheumatology 2024*, **22(2)**: PReS24-ABS-1651

**Introduction:** Major advances in research have included molecular studies on the pathogenesis of common rheumatic diseases, the discovery and validation of prognostic biomarkers, and clinical studies on novel therapies. These advances have relied on the recruitment of highly characterised patient populations and by the linked biorepositories of blood and synovial tissue.

The ARC Biobank was successfully set up in 2016 funded in partnership from charitable sources and through Arthritis Ireland and coordinated via the Rheumatology research centre UCD. The Paediatric arm (CHI @Crumlin) is one of 6 disease / patient specific groups enrolled in Ireland’s first national biobank for rheumatic diseases.

**Objectives:** To recruit cohorts of patients with common rheumatic disease and collect biological samples (DNA, RNA, serum) that will underpin clinical research in Rheumatology across Ireland.

**Methods:** Patients were recruited from Rheumatology outpatients clinics. Inclusion criteria: All patients with a diagnosis of a childhood inflammatory arthritis or a childhood onset autoinflammatory disorder (AID).

Participants were assessed at four timepoints: Baseline, Visit 2, Visit 3 and Visit 4, when they attended for routine clinic visits over the course of one to three years. (A minimum of two months between the first three visits and a minimum of five months between Visit 3 and Visit 4).

Clinical measurements such as clinical history and examination and all medications were obtained and relevant laboratory parameters were documented on each patient at each visit, along with sample collection if there was a clinical indication.

**Results:** Over 100 children have been successfully recruited as of March 2024. This includes Juvenile Idiopathic Arthritis (JIA=40), Chronic Nonbacterial Osteomyelitis (CNO=24), Down syndrome Associated Arthritis (DA =21), Systemic onset Juvenile Idiopathic Arthritis (SoJIA =10), Behcet’s Disease =3, Periodic Fever, Aphthous Stomatitis, Pharyngitis, Adenitis (PFAPA =1) Syndrome and Undifferentiated AID=1.

**Conclusion:** It is anticipated that the outcomes from this study will help to increase national involvement in clinical trials of novel therapeutic agents by establishing clinical cohorts of patients with common rheumatic diseases. This can advance patient treatment and improve longterm outcomes.

**Patient Consent**

Not applicable (there are no patient data)

**Disclosure of Interest**

None Declared

**References**

1. A toddler with an unusually severe polyarticular arthritis and a lung involvement: a case report. BMC Pediatr. 2022 Nov 4;22(1):639.
2. Copa Syndrome: a Novel Autosomal Dominant Immune Dysregulatory Disease. J Clin Immunol. 2016 May;36(4):377-387. Epub 2016 Apr 5.
3. SIGLEC1 (CD169) as a potential diagnostical screening marker for monogenic interferonopathies. Orak B, Ngoumou G, Ebstein F, Zieba B, Goetzke CC, Knierim E, Kaindl AM, Panzer A, Theophil M, Berns M, Krüger E, Meisel C, Unterwalder N, Kallinich T. Pediatr Allergy Immunol. 2021 Apr;32(3):621-625.
4. Baricitinib in therapy of COPA syndrome in a 15-year-old girl. 2020 Feb;7(Suppl1):S78-S81.

### Treatment

#### P465 Successful treatment of sapho syndrome in adolescent with secukinumab

##### Rinat Raupov^1, 2^, Segei Vissarionov^1^, Gumru Babaeva^1^, Mikhail Kostik ^2^

###### ^1^H. Turner National Medical Research Center for Children’s Orthopedics and Trauma Surgery; ^2^Saint-Petersburg State Pediatric Medical University, Saint-Petersburg, Russian Federation

####### **Correspondence:** Mikhail Kostik

*Pediatric Rheumatology 2024*, **22(2)**: PReS24-ABS-1230

**Introduction:** SAPHO (Synovitis–acne–pustulosis–hyperostosis–osteitis) is a heterogenous, more autoinflammatory, than autoimmune disease, that is characterized by specific skin and joints involvement. The etiology of SAPHO syndrome is not clear. Clavicle hyperostosis and multifocal CRMO are characteristic for children and adolescents, while adults more often have anterior chest wall and axial skeleton involvement. Skin disease can manifest before, after or with osteoarticular involvement. There are no consensus-based treatment guidelines of SAPHO syndrome. NSAIDS, bisphosphonates and TNF inhibitors have shown efficacy. Treatment with IL-17 blockers has been effective in adults with SAPHO syndrome in several publications.

**Objectives:** to describe the successful treatment adolescent with SAPHO syndrome with secukinumab.

**Results:** 15 years old boy has complained of periodic left hip pain for 2 years. Then left clavicle edema and pain have occurred. He was examined by pediatric rheumatologist. The posture is sluggish, limping, edema of the left clavicle, pain on palpation, restriction of movements in the cervical spine, pain on full flexion in the left hip, limitation of maximal flexion in the right elbow, acne on the forehead, face, chest and back. ESR and CRP were elevated, HLA-B27 was positive. The X-ray demonstrated hyperostosis of the cortical layer of the diaphysis of the left clavicle (Fig.1,A). The right hip and the left elbow synovitis were on ultrasound. Odontoid process C2 trabecular edema and C2 contour changes, initial signs of C2-C5 spondylodiscitis were observed on MRI (Fig.1, B). Sacroiliac joint MRI showed cyst-like changes of the left iliac bone (Fig.1, C). SAPHO syndrome was diagnosed based on the left clavicle hyperostosis, axial skeleton abnormalities, synovitis and skin acne. Initial treatment with NSAIDs didn’t show results. Secukinumab was started. The skin rash improvement, no joint complaints, laboratory inflammatory markers normalization were observed in 6 months.

**Conclusion:** IL-17 inhibitor could be used for treatment in children with SAPHO syndrome. Consensus-based guidelines for diagnosis and treatment of SAPHO syndrome is needed.

**Patient Consent**

Not applicable (there are no patient data)

**Disclosure of Interest**

None Declared

### Treatment

#### P466 Efficacy and safety of zoledronic acid for chronic non-bacterial osteomyelitis with vertebral involvement

##### Marco Francesco Natale ^1^, Virginia Messia^1^, Manuela Pardeo^1^, Silvia Federici^1^, Camilla Celani^1^, Fabrizio De Benedetti^1^, Antonella Insalaco^1^

###### ^1^Rheumatology, Bambino Gesù Children's Hospital, Rome, Italy

####### **Correspondence:** Marco Francesco Natale

*Pediatric Rheumatology 2024*, **22(2)**: PReS24-ABS-1473

**Introduction:** Chronic non-bacterial osteomyelitis (CNO) is an auto-inflammatory bone disorder presenting with a wide spectrum of clinical manifestations. It usually presents with recurrent bone pain in one or multiple sites and is often associated with arthritis, inflammatory bowel disease and/or skin involvement. Vertebral involvement in CNO is frequent (one third of cases) and represents a diagnostic challenge and an important cause of morbidity and long-term severe sequelae.

**Objectives:** To describe seven pediatric cases of CNO with vertebral involvement treated with zoledronic acid.

**Methods:** We describe seven pediatric patients affected by CNO with vertebral involvement. All patients were treated with zoledronic acid with a therapeutic scheme of four infusions, one every three months, with a dose of 0.025mg/kg each. T-test was used to compare groups and p-value <0.05 was considered statistically significant

**Results:** We report seven pediatric patients referred to our rheumatology department with a diagnosis of CNO. All patients presented vertebral involvement, four skin involvement including psoriasis or folliculitis and two lung involvement as inflammatory nodules. Increase of inflammatory markers was detected in four patients with a median value of C-reactive protein (CRP) of 3.68 mg/dl (0.8-8). In each patient we started zoledronic acid treatment in addition to a TNF inhibitor (4 patients with etanercept and 3 with adalimumab) and methotrexate. Baseline MRI total detected lesions were 7.71 (mean, SD 2.870) and spinal lesions were 2.86 (mean, SD 1.57). After one year the total lesions dropped to 4.28 (mean, p value 0.01) and vertebral lesions to 1.71 (mean, p value 0.007). Moreover after one year CRP was normal in all patients. Bone pain was completely resolved in 85% of cases and the skin manifestations disappeared in 3/4 patients. Just in one patient we detected persistent osteoarticular pain and nail psoriasis.Whole-body MRI of this patient showed an improvement of both total and spinal bone lesions. In three children we detected adverse events after the first infusion: mild asymptomatic hypocalcemia and fever lasting less than 48 hours. No vertebral fractures occurred during the course of treatment

**Conclusion:** Vertebral involvement is frequently described in patients with CNO and represents an important cause of morbidity and long-term sequelae. There is no consensus on the therapeutic approach to these patients. We describe seven patients treated with zoledronic acid in addition to TNF inhibitor and methotrexate. We observed a significant clinical and radiological improvement. No serious adverse events were identified and no vertebral fractures occurred after start of treatment. Further study with larger cohorts and control groups would be helpful to confirm these data.

**Date of birth::** février 17

**Patient Consent**

Yes, I received consent

**Disclosure of Interest**

None Declared

### Treat-to-target

#### P468 Analysis of treatment adherence in patients with juvenile arthritis

##### Semyon Zhukov^1, 2^, Victor Malievsky^1, 3^

###### ^1^BSMU, ^2^Rheumatology, ^3^RCCH, Ufa, Russian Federation

####### **Correspondence:** Semyon Zhukov

*Pediatric Rheumatology 2024*, **22(2)**: PReS24-ABS-1336

**Introduction:** Individual adherence is one of the important factors contributing to the effectiveness of treatment. Using an anonymous questionnaire, a survey was conducted of patients with juvenile arthritis (JA) in the cardio-rheumatology department of the Republican Children's Clinical Hospital in Ufa.

**Objectives:** Assessing adherence to therapy in patients with juvenile arthritis.

**Methods:** The simplest and shortest questionnaire is the validated Morisky-Green test. The test (MMAS-4) contains 4 questions that determine whether the patient forgets to take the drug, observes the time of taking the drug, skips an extra dose with good self-sensitivity or with poor self-sensitivity. The Morisky-Green Questionnaire Extended (MMAS-8) contains 8 questions that also determine how often the patient takes this drug, if he has missed a dose in the last 2 weeks, and whether the patient takes the drug on a particular day. The interview states that the patient was provided with information about the pass. The answer to each question is scored from 0 to 1 point. A survey of 120 children aged 4 to 18 years was conducted to observe traditional traditions using a Morisky-Green marker.

**Results:** 110 respondents (91.7%) are quite fully informed about the disease, have a good command of the methods of monitoring and treating their disease, and 10 patients (8.3%) know little about the disease, its measurements and continuation of treatment. The vast majority of patients - 116 people (96.7%) were informed about the drugs used to treat JA. 87 respondents (72.5%) received information about morbidity from their attending physician. Scoring on the Morisky-Green scale showed that 101 respondents (84.0%) adhered to monarchical rule, 12 patients (10.0%) were not sufficiently adherent and were at risk of developing non-adherence, 7 patients (6.0%) did not adhere to the relationship. Regarding drugs that directly affect the prognosis of the disease and life, 114 families of patients with JA (95.0%) are ready for long-term or lifelong therapy, 6 families (5.0%) doubt or are not ready for long-term use of drugs. Analysis of the questionnaire showed that 95 parents (79.2%) noted their reluctance to take medications for their child, and 25 respondents (20.8%) did not note this. 4 respondents (3.3%) rely on their memory when taking medications regularly, 72 patients (60.0%) use written or printed instructions related to the need to take medications, 8 respondents (6.7%) use the help of relatives for reminders about the need to take medication. take medications, a third of patients (36 people, 30.0%) used electronic means, programs for smartphones. At the same time, 106 respondents (88.3%) kept a self-observation diary, which may be due to the high level of information provided by parents when visiting children with JA. At the next stage, activities were carried out that could facilitate compliance with all medical standards. The majority of respondents (96 people, 80.0%) believe that the use of special applications for a smartphone helps a child comply with medication regimen; 18 patients (15.0%) doubt the effectiveness of such programs and only 6 people (5.0%) 0 %) consider such measures to be useless.

**Conclusion:** The study established a high level of adherence of patients with JA (100 people, 84.0%) according to the Morisky-Green scale MMAS-8

**Patient Consent**

Yes, I received consent

**Disclosure of Interest**

None Declared

**Reference**

1. The most effective measures aimed at increasing patient adherence to treatment, according to respondents, are educational activities (including health schools) aimed at increasing awareness of patients and their families about the disease, methods of its treatment, preventive measures and increasing the availability of medical care.

### Uveitis

#### P469 Proposal of the multinational interdisciplinary working group for uveitis in childhood (MIWGUC) how to wean dmards after reaching remission in jia associated anterior uveitis and idiopathic anterior uveitis in childhood

##### Ivan Foeldvari^1^, Ameenat Lola Solebo^2^, Regitze Bangsgaard^3^, Joke de Boer^4^, Joan Calzada-Hernandez^5^, Jesús Díaz-Cascajosa^5^, Tamas Constantin^6^, Mia Glerup^7^, Helene Ingels^8^, Stephanie Mata^9^, Ellen Nordal^10^, Harry Petrushkin ^11^, Rotraud Saurenmann^12^, Gabriele Simonini^13^, Joost Swart^4^, Jan Titz^9^, Jordi Anton^5^

###### ^1^Hamburg Centre for Pediatric and Adolescence Rheumatology, Hamburg, Germany; ^2^Great Ormond Street Hospital NHS Foundation Trust, London, United Kingdom; ^3^Department of Ophthalmology, Copenhagen University Hospital Glostrup/Rigshospitalet, Copenhagen, Denmark; ^4^Department of ophthalmology, University Medical Center Utrecht, Utrecht, Netherlands, ^5^Hospital de Sant Joan de Déu, Barcelona, Spain; ^6^Semmelweis University, Budapest, Hungary; ^7^Dept of Pediatric and Adolecent Medicine, Aarhus University Hospital, Aarhus; ^8^Department of Paediatrics and Adolescent medicine Copenhagen Paediatric Rheumatology; Copenhagen University Hospital; Rigshospitalet, Copenhagen, Denmark; ^9^Patients Representative, Hamburg, Germany; ^10^Department of Paediatrics, University Hospital of North Norway and UiT The Arctic University of Norway, Tromsø, Norway; ^11^Moorfields Eye Hospital NHS Foundation Trust, London, United Kingdom; ^12^Department of Paediatrics, Winterthur, Switzerland; ^13^Rheumatology Unit,ERN-ReCONNET center, Meyer Children's Hospital IRCCS, Firenze, Italy

####### **Correspondence:** Ivan Foeldvari

*Pediatric Rheumatology 2024*, **22(2)**: PReS24-ABS-1666

**Introduction:** 10 to 20% of patients with non-systemic juvenile idiopathic arthritis (JIA) develop uveitis (JIA-U) of which the majority need a disease modifying agent (DMARD) to achieve disease control. The published guidance from the SHARE (Single Hub and Access point for paediatric Rheumatology in Europe) and the MIWGUC (Multinational Interdisciplinary Working Group for Uveitis in Childhood) groups have ensured an evidence-based, standardized approach to DMARD commencement, and definition of disease remission. We sought to develop a much needed guidance on tapering or discontinuing DMARDs for JIA-U and non-JIA childhood idiopathic chronic anterior uveitis (CAU).

**Objectives:** To develop a much needed standardized concept of weaning dmards after reaching remission

**Methods:** A RAND/UCLA appropriateness method (RAM) consensus exercise undertaken through MIWGUC, involving pediatric rheumatologists (n=10), pediatric uveitis specialists (n=5) and patient representatives (n=2), in January 2024. The RAM comprised: a rapid scoping review, presentation of review findings to attendees, open discussion to develop statements, and expert scoring of statements with a threshold of ≥80% agreement across the group to define consensus.

**Results:** The statements which reached scores indicating consensus were: use of a standardised tapering schedule should be limited to those patients without any significant structural ocular damage; 24 months of disease remission are needed before DMARD tapering can begin; the definition of remission is inactive disease off topical therapy; children undergoing tapering should be examined for uveitis every 3 months at least applying the proposed MIWGUC assessment protocoll. Statements on discontinuation schedules involving dosage tapering, dosing interval extension and/or simple stopping were also developed for each of the most commonly used therapeutic interventions for JIA-U and CAU:

- methotrexate (MTX) monotherapy (interval extension from weekly to every 2 weeks then every 3 weeks or dose reduction from >15 to <10mg/m2/week)
- biologics (adalimumab, interval extension from x1/2 weeks to x1/5 weeks, infliximab, interval extension by 1-2 weeks and tocilizumab, interval extension by 1 – 2 weeks) monotherapy or
- biologic in combination with MTX, with consensus on the biologic agent being stopped first when used in combination. There was an additional consensus statement on an overall discontinuation window of 18 months for biologic therapies.

**Conclusion:** We present consensus-based recommendations for standardized medication tapering approaches for children with JIA-uveitis and idiopathic chronic anterior uveitis. The standardization of care enabled by these recommendations should support future multi-centre observational research on long term outcomes following medication tapering, and on the predictors of long-term disease remission for affected children and for adults with childhood onset disease.

**Patient Consent**

Not applicable (there are no patient data)

**Disclosure of Interest**

None Declared

### Uveitis

#### P470 Predictors of recurrence in pediatric patients with non-infectious uveitis undergoing adalimumab tapering: an international multicenter study

##### Achille Marino ^1^, Maria Vittoria Cicinelli^2, 3^, Elisabetta Miserocchi^2, 3^, Francesco Baldo^1^, Stefania Costi^1^, Cecilia Chighizola^1, 4^, Roberto Caporali^1, 4^, Francesco Pichi^5, 6^

###### ^1^Unit of Pediatric Rheumatology, ASST G. Pini-CTO; ^2^School of Medicine, Vita-Salute San Raffaele University; ^3^Department of Ophthalmology, IRCCS San Raffaele Scientific Institute; ^4^Department of Clinical Sciences and Community Health, Research Center for Pediatric and Adult Rheumatic Diseases (RECAP.RD), University of Milan, Milan, Italy; ^5^Eye Institute, Cleveland Clinic Abu Dhabi, Abu Dhabi, United Arab Emirates; ^6^Cleveland Clinic Lerner College of Medicine, Case Western Reserve University, Cleveland, United States

####### **Correspondence:** Francesco Baldo

*Pediatric Rheumatology 2024*, **22(2)**: PReS24-ABS-1185

**Introduction:** Adalimumab (ADA) is frequently administered to pediatric patients with non-infectious uveitis (NIU). Given safety considerations and cost implications, exploring the possibility of tapering/withdrawing ADA in patients experiencing a prolonged period of persistent remission has been suggested.

**Objectives:** To evaluate factors associated with the risk of NIU relapse in patients undergoing ADA tapering.

**Methods:** A multicenter retrospective cohort study was conducted. Patients diagnosed with NIU, with or without an associated systemic disease, before age 18 and treated with ADA were included. All patients underwent ADA tapering due to NIU inactivity. ADA tapering consisted of progressive injection spacing decided by the treating clinician.

**Results:** The cohort comprised 114 patients (57% female) with NIU treated with ADA. Demographic and clinical characteristics of the cohort are detailed in Table 1. Fifty-three patients (46%) experienced NIU recurrence after a median of 30 weeks (IQR 15-58 weeks) from the onset of ADA tapering. The interval between ADA injections was increased by 1 week every month (n =1), 1 week every 2 months (n =2), 1 week every 3 months (n = 50), 2 weeks every 4 months (n = 1), 1 week every 4 months (n=4),1week every 5 months(n=5), 4 weeks every 6 months(n=1), 2 weeks every 6 months(n=2),1 week every 6 months (n = 24), 1 week every 12 months (n = 24). Considering the heterogeneity in the distribution of patients across the speed of tapering, ADA tapering was classified into two main groups based on the rate of drug reduction: faster (fast_t, comprising tapering speeds from 1 week every month to 2 weeks every 4 months, N = 54) and slower (slow_t, encompassing tapering speeds from 1 week every 4 months to 1 week every 12 months, N = 60). An association between the speed class and the incidence of uveitis relapse was observed, with 56% of recurrences in the fast_t group compared to 38% in the slow_t group (p = 0.06). A multivariate Cox regression analysis was conducted to identify independent predictors of the recurrence rate. In the multivariate model, being Caucasian was associated with more than a two-fold increase in the risk of developing uveitis relapse (HR 2.33; 95% CI 1.12-4.85; p = 0.02). Furthermore, the adjusted analysis showed that a slower ADA tapering is associated with a 50% lower risk of recurrence than a faster tapering (HR 0.49; 95% CI 0.26-0.95; p = 0.03).

**Conclusion:** About half of the cohort experienced a NIU relapse after the initiation of ADA tapering. Caucasian race and fast tapering were associated with a higher risk of recurrences. Therefore, a strict follow-up for these patients should be advisable, and a gradual ADA taper is recommended.

**Date of birth::** septembre

**Patient Consent**

Yes, I received consent

**Disclosure of Interest**

None Declared

**Table 1 (abstract PReS24-ABS-1185).** General features of the cohort

	Overall (***N***=114)	No recurrences (***N***=61 pts)	Recurrences (***N***=53 pts)	***p***
**Age, years, mean (sd)**	12 (3.9)	12 (4)	12 (3.8)	0.81
**Ethnicity**				0.03
Caucasian	74 (65%)	34 (55%)	40 (75%)	
Non-Caucasian	40 (35%)	27 (45%)	13 (25%)	
**Diagnosis**				0.51
Idiopathic Uveitis	46 (40%)	22 (36%)	24 (45%)	
Juvenile idiopathic arthritis	52 (46%)	28 (46%)	24 (45%)	
Other	16 (14%)	11 (18%)	5 (10%)	
**Inactivity before adalimumab taper onset, weeks, mean (sd)**	163 (174)	189 (200)	133 (132)	0.72

### Uveitis

#### P471 Ophthalmological complications after 35 years of Juvenile idiopathic arthritis - results from the South Swedish juvenile idiopathic arthritis cohort

##### Ola Rauer ^1, 2^, Alma Dahlberg^2, 3, 4^, Angelika Skarin^1, 2^, Elisabet Berthold^2, 5^, Robin Kahn^2, 3, 4^, Helena Tydén^5, 6^

###### ^1^Department of Ophtalmology, Clinical Sciences, Lund University, Lund; ^2^Skane University Hospital, Lund and Malmö; ^3^Department of Pediatrics, Clinical Sciences, Lund University; ^4^Wallenberg Center for Molecular Medicine, Lund University; ^5^Department of Rheumatology, Clinical Sciences, Lund University; ^6^Skane University Hospital, Lund, Sweden

####### **Correspondence:** Helena Tydén

*Pediatric Rheumatology 2024*, **22(2)**: PReS24-ABS-1761

**Introduction:** Active uveitis in adults with JIA has been reported from retrospective studies, but data from cross-sectional clinical investigations of population-based cohorts are scarce(1-4)

**Objectives:** To investigate the presence of active uveitis and its complications of glaucoma, cataract, and visual impairment in adults with JIA and up to 44 years of disease duration from a population-based, validated JIA cohort from southern Sweden.

**Methods:** Individuals diagnosed with JIA between 1980-1993 were recruited from a pre-existing population-based JIA cohort in southern Sweden and examined by an ophthalmologist and a rheumatologist. Presence of active or previous uveitis, glaucoma, cataract, and visual impairment, as well as general disease activity was recorded.

**Results:** Adults with JIA (n=100, 78% female, mean age 42 years, mean disease duration 35 years, 51% ANA positive, 49% oligoarthritis) were included.

Of these, 15/100 patients and 27/30 eyes had a history of uveitis (66% female, 73% ANA positive, 60 % oligoarthritis).

Active anterior uveitis was found in 3/27 and flare in 8/27 eyes, where 5/27 eyes were on topical corticoid steroid drop treatment.

Cataract was found in 13/27 uveitis eyes and in 22/200 eyes in the whole cohort. Glaucoma caused by uveitis was found in 7/27 uveitis eyes.

Severe visual impairment (< 0,1) (Snellen <20/200) caused by uveitis was found in 3/27 eyes. In the whole cohort 1/200 eyes had moderate visual impairment (0,1-<0,3) (Snellen 20/200-<20/70) while the remaining 196/200 eyes had mild or no visual impairment (≥0,3) (Snellen ≥20/70).

**Conclusion:** After a median follow up time of 35 years, 60% of the JIA patients with uveitis had one or more complications to their eye disease and approximately one of five had ongoing topical steroid treatment. Severe visual impairment was uncommon in uveitic eyes. No new undiagnosed uveitis was found.

**Patient Consent**

Not applicable (there are no patient data)

**Disclosure of Interest**

None Declared

**References**

1. Berthold E et al. Arthritis Res Ther. 2019;21(1):218.
2. Rypdal V et al. Ophthalmology. 2021;128(4):598-608.
3. Kotaniemi K et al. Ann Rheum Dis. 2005;64(6):871-4.
4. Skarin A et al. Pediatr Rheumatol Online J. 2022;20(1):47.

### Uveitis

#### P472 Long-term outcome of pediatric chronic anterior uveitis from a single centre in North India

##### Sujata Sawhney^1^, Prajakta Dekate^1^, Amit Khosla^2^, Manjari Agarwal^1^

###### ^1^Pediatric Rheumatology, ^2^Ophthalmology, Sir GangaRam Hospital, Delhi, India

####### **Correspondence:** Sujata Sawhney

*Pediatric Rheumatology 2024*, **22(2)**: PReS24-ABS-1438

**Introduction:** We run a paediatric uvea-rheumatology clinic for > 20 years and evaluated long-term visual acuity(VA) in patients with non-infectious Chronic anterior uveitis (CAU) .

**Objectives:** To assess long-term outcome of isolated CAU or CAU with JIA and study factors impacting final VA.

**Methods:** An ambispective analysis of the study population was done. Minimum 5-year follow-up. VA>/= 6/9 good outcome/gr 1, VA </=6/12 gr 2/adverse outcome. SPSS version 28, Chi square and Fisher/s exact test used to assess factors impacting VA in JIA uveitis.

**Results:** *Whole cohort:* 42 children(34F, 8M) with CAU,≥5 years followup. Age at presentation:12-223 months (mo), median 37.5mo. 9 isolated CAU, 33 JIA associated CAU(19 OJIA,10 PJIA,3 EOJIA,1 PsJIA). 1 had uveitis prior to arthritis. ANA positive:34(81%). 9(21.4%) had isolated CAU, 13(31%) JIA uveitis at screening, 20(47.6%) developed during follow-up(60-311 mo). 11(26%) patients and total 14 eyes(17.7%) underwent cataract surgery, of whom 10 eyes had cataract at presentation,2(5%) glaucoma surgery, and 1(2%) BSK removal. Male sex(p=0.031),complicated uveitis at presentation(p=0.031) and use of Anti-TNF therapy (p=0.013) associated with adverse VA. Initial vision did not predict the final visual outcome neither in the whole cohort nor in the Isolated uveitis or Uveitis associated with JIA subgroup.

*Isolated CAU:* Median diagnosis age: 66mo(IQR 77.5). 7(77.8%) female. Symptoms:4 redness,1 pain, 6 diminution of vision. Initial visit:7 (77.8%) both eye involved, 2 unilateral. Of 16 eyes:56% Grade(Gr)1 VA,44% Gr2. Final visit: 83% Gr 1,17% Gr 2. Complications: Initial-Posterior synechiae(PS) and cataract(44% each), glaucoma(13%),BSK(6%). Final-(PS) and BSK 22%. Cataract 17%; glaucoma nil. Treatment: Mtx and ADA for all; 2(22%) Golimumab; 1(11%) Infliximab, MMF, Tofacitinib each. 1 given 3 Anti-TNF over 123mo; 1 given ADA for 8y then Golimumab. 59.3% on Anti TNF achieved 6/6-6/9 versus 6.7% without. No difference in VA between isolated CAU or JIA associated CAU (p=0.406).

*JIA with Uveitis:* 33 patients, median uveitis diagnosis 56mo(IQR 26.5),median 26mo b/w arthritis and uveitis. 27 female(82%), 28(85%) ANA+ve. Pre-uveitis:16 Mtx, 5 Etanercept. Initial visit: 22(67%) had both eyes involved. T/t:All received topical/systemic steroids+Mtx; ADA 18(55%), MMF 6(18%). Isolated uveitis flare in 23(70%), arthritis-associated 5(15%). At last FU: Mtx 31(94%), ADA 13(39%) Tofacitinib 4(12%), MMF and Actemra 2(6%) each; 1(3%) Golimumab. Initial: 22(67%) both eyes and Last FU: 29(2%) had both eyes involved. First visit VA:40 eyes(73%) Gr 1, 15(27%)Gd 2. Last visit: 50 (86%)Gr1,8(14%)Gr2. Initial: PS 16%, BSK15%, cataract 9%, no glaucoma. Last: PS10%, BSK 7%, cataract 12%, new glaucoma 2%. Factors that impacted adverse VA were male sex (p=0.02), complicated uveitis at presentation (p=0.046).

**Conclusion:** 42 children had CAU with a median follow-up of 108 months. At last visit, VA >6/9 was observed in 30(71%) and <6/12 in 12(29%) respectively. Anti-TNF was used in 27(64%). Male sex and complicated uveitis at presentation adversely impacted VA in uveitis associated with JIA and in the whole cohort.

**Patient Consent**

Not applicable (there are no patient data)

**Disclosure of Interest**

None Declared

### Uveitis

#### P473 Efficacy of tocilizumab versus anti-tnf switching in refractory Juvenile idiopathic arthritis-associated uveitis

##### Gisella Beatrice Beretta ^1, 2^, Chiara Mapelli^3^, Marco Nassisi^3^, Federica Lucioni^1, 2^, Elisabetta Lo Iudice^1, 2^, Gaia Leone^3^, Stefano Lanni^1^, Martina Rossano^1^, Giovanni Filocamo^1^, Francesca Serena Minoia^1^

###### ^1^Pediatric Immuno-Rheumatology Unit, Fondazione IRCCS Ca' Granda Ospedale Maggiore Policlinico, ^2^University of Milan, ^3^Ophthalmology Unit, Fondazione IRCCS Ca' Granda Ospedale Maggiore Policlinico, Milan, Italy

####### **Correspondence:** Gisella Beatrice Beretta

*Pediatric Rheumatology 2024*, **22(2)**: PReS24-ABS-1453

**Introduction**

Juvenile idiopathic arthritis-associated uveitis (JIA-U) can often be refractory not only to methotrexate (MTX) but also to first-line biological disease modifying antirheumatic drug (bDMARD). The optimal choice of therapy following the failure of initial anti-TNF treatment remains uncertain.

**Objectives**

Assessing therapeutic strategies in a cohort of JIA-U patients resistant to methotrexate and first-line bDMARDs to determine the optimal treatment approach.

**Methods**

Clinical records of patients with JIA-U followed at our tertiary Pediatric Rheumatology centre were retrospectively reviewed, collecting data regarding clinical features, treatment and outcomes. Differences between patients managed with a single bDMARD and those requiring multiple biologic agents were evaluated using χ2 or unpaired T-tests, as appropriate.

**Results**

Data from 65 JIA-U patients treated with MTX (83.1% female) were analyzed, with a mean follow-up of 8.41 years and a mean age at uveitis onset of 5.28 years. Among these patients, 48 required at least one bDMARD for uveitis management, while 17 (26.2%) needed multiple bDMARDs (9 patients required two bDMARDs, while 8 patients necessitated 3 or more bDMARDs).

Adalimumab and infliximab served as first-line agents in 41 and 7 patients, respectively. Following the failure of initial anti-TNF treatment, 11 patients transitioned to a new anti-TNFa (infliximab or adalimumab), while 6 switched to tocilizumab. Uveitis control was more frequently achieved by switching from adalimumab to tocilizumab compared to infliximab (p=0.044). Additionally, tocilizumab proved effective in other 4 patients unresponsive to anti-TNF switching. Golimumab and Abatacept were employed as third-line biologics in 3 and 4 patients, respectively. However, Golimumab did not lead to remission of uveitis in any patient, whereas only two patients receiving Abatacept achieved remission.

Children requiring multiple bDMARDs for uveitis had a more frequent polyarticular course (p=0.03) and a higher prevalence of systemic steroid use (p<0.001) compared to those responsive to first-line bDMARDs. Despite a similar frequency of ocular damage at onset, patients needing multiple bDMARSs exhibited a higher percentage of ocular damage at the last visit (58.8% vs 27.6%, p=0.02).

**Conclusion**

Managing refractory JIA-U can be challenging, particularly in patients failing first-line anti-TNF. Switching to tocilizumab may represent a relevant therapeutic option, although these results need to be confirmed in larger cohort studies.

**Patient Consent**

Yes, I received consent

**Disclosure of Interest**

None Declared

### Uveitis

#### P474 Late onset juvenile idiopathic arthritis-associated uveitis: preliminary data from a single-center study

##### Stefania Costi^1^, Claudia Iannone^2^, Elisabetta Miserocchi^3^, Francesco Baldo^1^, Achille Marino^1^, Cecilia B. Chighizola^1, 4^, Roberto Caporali^1, 4^

###### ^1^Pediatric Rheumatology, ASST-Pini-CTO; ^2^ Rheumatology, Università degli Studi di Milano; ^3^Ophtalmology, Università Vita e Salute San Raffaele; ^4^Università degli Studi di Milano , Milan, Italy

####### **Correspondence:** Stefania Costi

*Pediatric Rheumatology 2024*, **22(2)**: PReS24-ABS-1506

**Introduction:** Uveitis is the most fearful extra-articular complication in juvenile idiopathic arthritis (JIA) and a significant cause of disability. In most cases, it occurs simultaneously or after the diagnosis of arthritis, usually in the first four years after the onset of the joint disease.

**Objectives:** The study aims to evaluate and describe cases of late-onset uveitis.

**Methods:** We retrospectively reviewed medical records of patients followed in our Center suffering from JIA-associated uveitis from 1985 to 2023.

**Results:** 114 patients suffering from JIA complicated by uveitis were included in the study (F 78%; n=89) with a mean follow-up time of 21 years (SD ± 10.9) from the joint or ocular disease onset. 99 patients (87%) were classified into the oligoarticular JIA subgroup, 12 (11%) in the polyarticular FR negative category, 2 (2%) in the psoriatic form, and 1 (1%) presented a form of acute anterior uveitis HLA B27 correlated with the diagnosis of enthesitis-arthritis. ANA antibodies were positive in 111 patients (97%). In 108 patients (95%) joint disease preceded ocular involvement, while in only 6 patients (5%) uveitis preceded arthritis. In the majority of cases, uveitis manifested itself in the first 4 years from diagnosis of the joint disease with a median arthritis-uveitis time of 12 months (IQR 37); however, in 25 patients (25/108; 23%), uveitis appeared after the first 4 years of the disease. In the latter subgroup, all patients had disease onset before 5 years of life and were classified as oligoarticular JIA ANA positive, except for 4 patients diagnosed with polyarticular JIA FR negative, ANA positive.

19/25 patients (76%) were female. The median time between the onset of arthritis and uveitis was 7 years (IQR 3.5; range 4.5-23), with a mean number of visits equal to 19 (SD ±7.9) before the detection of uveitis. At the time of uveitis onset, 10 patients were on methotrexate monotherapy and 2 on methotrexate in combination with etanercept.

In patients with uveitis appearing in the first 5 years of life, the onset of ocular disease occurred within 4 years from the onset of arthritis, unlike patients with uveitis onset in the first 5 years of life in whom uveitis could manifest after many years from the onset of the joint disease (p= 0.011). Furthermore, a negative correlation was found between age at onset of arthritis and age at onset of uveitis (r= -0.2; p=0.036).

**Conclusion:** The greatest risk of uveitis occurs in the first years of the disease after the onset of the joint disease. However, a non-negligible portion of patients with risk factors for uveitis appear even after many years of disease; therefore, long-term follow-up seems to be the most prudent approach in this subgroup of patients.

**Date of birth::** octobre 10

**Patient Consent**

Yes, I received consent

**Disclosure of Interest**

None Declared

### Uveitis

#### P475 Clinical Characteristics And Risk Factors Of Severe Disease In Pediatric Non-Infectious Uveitis: A Single Center Study

##### Lutfiye Koru^1^, Fehim Esen^2^, Ozlem Turkyılmaz^2^, Feray Kaya^1^, Elif Kucuk^1^, Zelal Aydın^1^, Fatih Haslak^1^, Kubra Ozturk^1^

###### ^1^Pediatric Rheumatology, ^2^Ophthalmology, Istanbul Medeniyet Universty, Istanbul, Türkiye

####### **Correspondence:** Lutfiye Koru

*Pediatric Rheumatology 2024*, **22(2)**: PReS24-ABS-1538

**Introduction:** Pediatric non-infectious uveitis is a rare condition characterized by inflammation of the uveal components of the eye. Untreated, it may cause blindness and is sometimes linked to rheumatologic diseases like juvenile idiopathic arthritis (JIA).

**Objectives:** In our study, we aimed to present the demographic, clinical, laboratory and treatment data of patients with non-infectious uveitis. We also aimed to evaluate the effect of extraocular involvement on the clinic and prognosis, as well as to evaluate the risk factors for the development of complications and the need for biological treatment.

**Methods:** Patients diagnosed with non-infectious uveitis in childhood and followed up in our tertiary care center for at least 1 year were included in the study. Demographic data including age, gender, age at diagnosis, uveitis in first-degree relatives and rheumatologic diseases were obtained retrospectively from medical records. The presence of complications or the need for biologic therapy was considered as a composite outcome and patients with this composite outcome were considered to have severe disease.

**Results:** The study included 123 patients (Female: n=59 (40.7%)). The mean age at diagnosis was 14.89 ± 4.86 years. Uveitis was symptomatic in 104 patients (71.7%). Approximately one quarter of the patients had at least one rheumatic disease (n=35, 24.1%), the most common being JIA. Thirty-three patients (22.8%) were anti-nuclear antibody (ANA) positive. Biologic agents were needed in 60 (41.4%) patients. Complications developed in 9.7% (n=14) of patients. Early age (aOR, 0.875; 95% C.I.: 0.795- 0.915, p=0.007)and female gender (aOR, 2.99; 95% C.I.: 1.439- 6.248, p=0.003 was found to be an independent risk factor for the need for biological treatment. Additionally, Behcet's disease was found to be a indipendent risk factor for uveitis-related complication (aOR, 0.071; 95% C.I.: 0.014- 0.362, p=0.001).

**Conclusion:** Patients with uveitis, particularly those with an early onset of the disease, female patients, and those with Behçet's disease, should be closely monitored for the necessity of biologic therapy and potential complications.

**Date of birth::** octobre 15

**Patient Consent**

Yes, I received consent

**Disclosure of Interest**

None Declared

**References**

1. Kump, L. I., Cervantes-Castañeda, R. A., Androudi, S. N., & Foster, C. S. (2005). *Analysis of pediatric uveitis cases at a tertiary referral center*. Ophthalmology, 112(7).
2. Sen, E. S., & Ramanan, A. v. (2020). *Juvenile idiopathic arthritis-associated uveitis.* Clinical Immunology (Orlando, Fla.), 211.
3. Ozdel, S., Baglan, E., Gungor, T., Yazılıtas, F., Cakıcı, E. K., Ozdal, P. C., & Bulbul, M. (2021). *Comparison of pediatric patients with noninfectious idiopathic uveitis and noninfectious uveitis associated with an underlying systemic disease: from a referral center in Turkey.* Postgraduate Medicine, 133(4), 444–448.

### Uveitis

#### P476 Uveitis onset in juvenile idiopathic arthritis: do we need to reconsider factors driving the ophthalmology screening?

##### Ilaria Maccora^1, 2^, Carolina Burgassi^3^, Edoardo Marrani^1^, Maria Vincenza Mastrolia^1, 2^, Ilaria Pagnini^1^, Gabriele Simonini^1, 2^

###### ^1^Rheumatology Unit, Meyer Children's Hospital IRCCS, ^2^Neurofarba department; ^3^University of Florence, Florence, Italy

####### **Correspondence:** Gabriele Simonini

*Pediatric Rheumatology 2024*, **22(2)**: PReS24-ABS-1363

**Introduction:** Juvenile Idiopathic Arthritis (JIA) is the most common chronic rheumatic disease in childhood, and it is burdened by uveitis (U) in the 20-24% of cases. In most of the cases U have an onset within the first years from JIA onset, and only a minimal part later. Based on this, an ophthalmology screening was built.

**Objectives:** To describe a cohort of children with JIA associated U (JIA-U) and to report the factors associated with a later U onset.

**Methods:** In a retrospective cohort study, we enrolled all patients currently followed in our Rheumatology Unit, with a diagnosis of JIA according to ILAR criteria and U history. Demographics, clinical, laboratory and treatment data were collected. The main outcomes were U onset after 4 years of JIA and the time lapse between arthritis and U onset.

**Results:** We identified 82 children with JIA-U (68 female (82.9%), 75 ANA positive (91.5%)), with a median age at onset of arthritis of 28 months(m) (IQR 23-51) and U onset of 48 m (IQR 31-88), and a median follow-up of 88 m (IQR 46-128). 78 children had anterior-U (95.1%), 51 oligoarticular-JIA (62.2%), 23 polyarticular-JIA (28%), 6 psoriatic JIA (7.3%) and 2 ERA (2.4%). The median time between arthritis and U onset was 5.5 m (IQR 0-29), with 10 children with U onset before arthritis (12.2%), 16 after 3 years of JIA (19.5%) and 10 after 4 years (12.2%). JIA-U showed a median ESR at onset of 36 mm/h (IQR 26-60) and a CRP at onset of 1.41 mg/dl (IQR 0.38-3.45).

35 JIA received at least a systemic treatment (ST) any time before U onset (42.7%). At U onset 32 were on ST (24 methotrexate, 4 etanercept and 3 adalimumab) (39%), 54 had active arthritis (65.9%) and 7 developed U after drug withdrawal (8.5%). We observed significant differences in the time between arthritis and U onset, regarding the biologic sex (9 m (female) vs 0.5 m (male), p 0.024), to be on ST at U onset (18 m vs 1 m, p <0.001), to have active arthritis at U onset (29 m vs 3 m, p 0.001), to have stopped the ST (50 m vs 4 m, p<0.001), to have received a ST any time (21m m vs 1 m p<0.001 (e^-7^)). Furthemore, we observed significant differences regarding U onset before/after 4 years of JIA and to be on ST at U onset (χ_2_ 5.85 p0.016) regardless of the specific ST, the arthritis activity (χ_2_6.58 p 0.01), the withdral of ST (χ_2_15.66 p0.001 (e-10^5^)), to have received a ST any time before U (χ_2_8.18 p0.004), the ESR value at onset (p0.033) and CRP value at onset (p 0.002).

Considering the significant variables identified, performing logistic regression, the only variables associated to U onset after 4 years of JIA were the arthritis status (OR 0.169, CI 0.029-0.985), the ST status at U onset (OR 8.71 CI 1.19-63.5) and the withdrawal of ST (OR 15.70 CI 2.02-121.59).

**Conclusion:** As evaluated in our retrospective study, we observed that timing of U onset in JIA may be influenced by the ST status, to have withdrawn a ST and the arthritis status. If these findings will be duplicated in a larger cohort, the ophthalmology screening in high-risk patients should be intensified after drug withdrawal, in case of active arthritis without ST. Conversely, receiving a ST seems to be protective for the development of U during the disease course.

**Patient Consent**

Yes, I received consent

**Disclosure of Interest**

None Declared

### Uveitis

#### P477 Non-infectious uveitis in pediatric rheumatology: long-term follow-up at tertiary centers

##### Nergis Akay^1^, Umit Gul^1^, Oya Koker^2^, Mustafa Asim Erol^3^, Mehmet Yildiz^1^, Elif Kilic Konte^1^, Ebru Altinok^2^, Aybuke Gunalp^1^, Esma Aslan^1^, Fatih Haslak^1^, Amra Adrovic^1^, Sezgin Sahin^1^, Kenan Barut^1^, Didar Ucar^3^, Ilknur Tugal Tutkun^4, 5^, Ozgur Kasapcopur^1^

###### ^1^Department of Pediatric Rheumatology, Istanbul University-Cerrahpasa, Cerrahpasa Medical School; ^2^Department of Pediatric Rheumatology, Marmara University-Pendik Training and Research Hospital; ^3^Department of Ophthalmology, Istanbul University-Cerrahpasa, Cerrahpasa Medical School; ^4^Department of Ophthalmology, Istanbul University, Istanbul Medical School; ^5^Department of Ophthalmology, Uveitis Outpatient Clinic, Eye Protection Foundation Bayrampasa Eye Hospital, Istanbul, Türkiye

####### **Correspondence:** Nergis Akay

*Pediatric Rheumatology 2024*, **22(2)**: PReS24-ABS-1414

**Introduction:** Non-infectious uveitis is mostly idiopathic in the pediatric population and may also occur in association with systemic diseases.

**Objectives:** Our study aimed to assess the characteristics and outcome of children with idiopathic uveitis and systemic disease-related uveitis (SD-U) as well as the categories of etiologies associated with SD-U.

**Methods:** Patients with non-infectious uveitis before the age of 18 years and followed up for at least 3 months in 2 tertiary centers of pediatric rheumatology and ophthalmology departments were included in the study. Demographics, etiology, clinical features, laboratory data, and treatments were evaluated retrospectively and compared based on the etiology and the use of biologic disease-modifying antirheumatic drugs (bDMARD).

**Results:** Out of 244 patients (131 with idiopathic uveitis and 113 with SD-U), 141 (57.8%) were female. The mean age at diagnosis was 8.7 (±3.9) years, and the mean follow-up was 45.6 (±39.2) months. We observed that uveitis was mostly anterior (n=140, 57.4%), chronic (n=122, 67.4%), and bilateral (n=146, 59.8%). The mean CRP and ESR values were 5.27 (±10.05) mg/L and 16.91 (±12.54) mm/h. ANA positivity was observed in 48.8% of all uveitis patients. Among the 113 SD-U patients, uveitis occurred in 47 (41%) at diagnosis of the SD, in 5 (4.4%) patients before diagnosis, and in 61 (54%) after diagnosis. Patients with SD-U showed a higher prevalence of female predominance, younger age at diagnosis, bilateral involvement, chronic course, increased ESR value, and ANA positivity compared to patients with idiopathic uveitis. However, the idiopathic uveitis group observed a higher prevalence of intermediate uveitis and acute course (p < 0.05). Out of all the patients, 47 (19.2%) had been treated with non-biologic DMARD (nbDMARD) monotherapy, 62 (25.4%) with bDMARD monotherapy, and 89 (36.5%) treated with both treatment regimens. Uveitis-related complications occurred in 103 (42.2%) patients with the most common being posterior synechiae (n=60, 24.6%). Among the ocular complications, band keratopathy was observed more commonly in the SD-U group than in those with idiopathic uveitis (p<0.05). Ocular surgery was required for 7 patients (5.3%) in idiopathic uveitis and 14 patients (12.4%) in SD-U group.

**Conclusion:** Our study showed a higher frequency of ocular complications including surgery and bDMARD usage in the SD-U group compared to those with idiopathic uveitis.

**Date of birth::** janvier 12

**Patient Consent**

Yes, I received consent

**Disclosure of Interest**

None Declared

**References**

1. Tuğal-Tutkun İ. An overview of pediatric uveitis. Turkish Archives of Pediatrics. 2023;58(4):363.
2. Gentile P, Ragusa E, Bolletta E, De Simone L, Gozzi F, Cappella M, et al. Epidemiology of pediatric uveitis. Ocular Immunology and Inflammation. 2023;31(10):2050-9.
3. Nguyen AT, Koné-Paut I, Dusser P. Diagnosis and Management of Non-Infectious Uveitis in Pediatric Patients. Pediatric Drugs. 2023:1-17.
4. Shivpuri A, Turtsevich I, Solebo AL, Compeyrot-Lacassagne S. Pediatric uveitis: Role of the pediatrician. Frontiers in Pediatrics. 2022;10:874711.
5. Maleki A, Anesi SD, Look-Why S, Manhapra A, Foster CS. Pediatric uveitis: a comprehensive review. Survey of ophthalmology. 2022;67(2):510-29.

### Uveitis

#### P478 Acute ocular involvement as early presenting sign of Juvenile behcet's disease

##### Camilla Sembenini^1^, Francesca Tirelli^1^, Andrea Leo^1^, Alessandra Meneghel^1^, Francesco Zulian^1^, Maria Elisabetta Zannin^2^

###### ^1^Departement for Women and Child’s Health, University Hospital of Padua, Italy, Pediatric Rheumatology Unit; ^2^Departement for Women and Child’s Health, University Hospital of Padua, Italy, Ophtalmology Unit, Padua, Italy

####### **Correspondence:** Camilla Sembenini

*Pediatric Rheumatology 2024*, **22(2)**: PReS24-ABS-1769

**Introduction:** Juvenile Behçet's disease (jBD) is a quite rare condition and can be a challenging diagnosis as the early clinical picture may be incomplete. Sight-threatening ocular manifestations are common but often are misdiagnosed at the disease onset or appear later in the disease course.

**Objectives:** In this study, we aimed to report a series of cases of jBD with acute ocular symptoms as presenting signs of the disease.

**Methods:** Retrospective analysis of a cohort of jBD patients presenting with acute ocular manifestations as only signs of the disease. Demographic, ophthalmological and systemic data at presentation and during follow-up were collected and analyzed.

**Results:** Eleven patients, 8 males(72.7%), mean age at onset 8.5 years (range: 5-13 y) have been consecutively diagnosed at our center between 2015 and 2023, for HLA B51-positive acute noninfectious uveitis. Ocular involvement included 6 (54.5%) panuveitis, all but one bilateral, 3 (27,2%) bilateral anterior uveitis (AU), 1 (9%) bilateral intermediate uveitis and 1(9%) bilateral central retinal vasculitis. In all cases, uveitis was syntomatic and acute at onset, with a relapsing course. Topical treatment was immediately started after uveitis diagnosis, with a median delay from the onset of ocular syntoms of 24 days (range 5-45). Only 4 patients (36,3%, 2 M and 2 F) developed extra-ocular jBD systemic signs or symptoms during the F/U: 4 oral aphthosis, 1 genital ulcerations and 1 sensoryneural hearing loss. No one had vascular or mucocutaneous involvement. As for treatment, 10/11 patients (91%) required systemic treatment with DMARDs to control ocular inflammation, including two patients treated with colchicine and 8 with methotrexate (MTX). Due to frequent uveitis relapses, 3/4 children with systemic symptoms needed combined treatment with MTX and biologics, specifically canakinumab for a girl with retinal vasculitis and adalimumab (ADA) in the other two cases, while only 1/7 patient in the ‘non-systemic’ group required ADA. All patients responded to systemic treatment, without progressive visual impairment.

**Conclusion:** Bilateral acute ocular symptoms may represent the early presenting signs of jBD and are often the only manifestation of the disease. A careful differential diagnosis with similar acute conditions such as acute uveitis in HLA-B27+ patients or infections should be performed. In these cases, the search for HLA-B51 is crucial. Since systemic symptoms correlate with a severe disease course, a second line therapy with csDMARDs or bDMARDs and a close rheumatological follow up are advisable.

**Patient Consent**

Yes, I received consent

**Disclosure of Interest**

None Declared

### Uveitis

#### P479 Are any differences in features and outcomes between Juvenile idiopathic arthritis-associated uveitis and chronic idiopathic uveitis?

##### Alexandr Iakovlev, Ekaterina Gaidar, Tatiana Nikitina, Mikhail Kostik

###### Saint-Petersburg Pediatric State Medical University, Saint-Petersburg, Russian Federation

####### **Correspondence:** Mikhail Kostik

*Pediatric Rheumatology 2024*, **22(2)**: PReS24-ABS-1180

**Introduction:** Chronic idiopathic uveitis (CIU) and uveitis associated with juvenile idiopathic arthritis (JIA) are vision-threatening conditions with similar mechanisms, but while JIA-associated uveitis has multiple treatment options including biologic and non-biologic DMARDs, in contrast to limited CIU poses a challenge due to a lack of specific treatments.

**Objectives:** The aim of the study was to compare features and outcomes of juvenile idiopathic arthritis (JIA)-associated uveitis and chronic idiopathic uveitis.Начало формы

**Methods:** A retrospective cohort study included the data of 111 patients (<18 years) with JIA-associated uveitis and 39 patients with CIU from January 2012 to May 2023. Analysis focused on demographic and clinical characteristics, treatments, and outcomes.

**Results:** The onset of arthritis was 5.7 (2.7; 9.3) years. Uveitis onset before arthritis was diagnosed in 32 (29%), simultaneously in 19 (18%), and after arthritis in 57 (53%) patients. We observed a lot of similarities (onset age, ANA and HLAB27-positivity, ESR) between groups, with slight female predominance (67% vs 60%, p=0.086) in JIA-associated uveitis, and predominance of anterior uveitis (OR=3.0 (95%CI: 1.4; 6.4), p=0.004), higher CRP 2.67 mg/l (1.0; 17.0) mg/l vs 0.98 mg/l (95%CI: 0.34; 1.7), p=0.031) in the same group. The frequency of symptomatic (red eye), unilateral and complicated form of uveitis at first observation was equal in both groups. The number of patients treated with methotrexate (87% vs 90%, p=0.599) and biologics (69% vs 74%, p=0.557) was the same with similar remission rate on methotrexate (67% vs 55%, p=0.233) and on biologics (90% vs 96%, p=0.369) in JIA-associated uveitis and CIU, respectively. The time before methotrexate was shorter in JIA-associated uveitis, the probality to receive methotrexate was higher (HR=1.54 (95%CI:1.04; 2.28), p=0.033), the remission achieved slower and flares were rarely with similar time before first flare on methotrexate. In patient failed to methotrexate biologics were prescribed in two years in both groups with similar outcomes (table).

**Conclusion:** The treatment of chronic idiopathic uveitis often features a more conservative approach with a delayed start of systemic therapy, which may affect long-term outcomes. Furthermore, the introduction of methotrexate in the chronic idiopathic uveitis group leads to a relatively rapid establishment of disease control.

**Patient Consent**

Not applicable (there are no patient data)

**Disclosure of Interest**

None Declared

**Table 1 (abstract PReS24-ABS-1180).** See description

Parameter, Me (25%;75)	JIA-associated uveitis, *n*=111 (%)	CIA, *n*= 39 (%)	*p*
Type of uveitis, n (%)			
- Anterior^*^	82 (73)	19 (49)	**0.032**
- Parsplanitis	4 (4)	2 (5)	
- Posterior	4 (4)	4 (10)	
- Panuveitis	21 (19)	14 (36)	
Time before methotrexate, years	1.6 (0.1; 2.3)	2.7 (0.4; 4.2)	**0.005**
Time before remission on methotrexate, years	0.75 (0.17; 0.42)	0.2 (0.1; 0.3)	0.07
Flares on methotrexate, n (%)	27/58 (47)	12/16 (75)	**0.044**
Time before flare, years	1.0 (0.75; 1.7)	1.2 (0.3; 1.4)	0.3
Time before biologics since uveitis onset, years	2.7 (0.7; 4.0)	4.0 (1.1; 6.2)	**0.03**
Time before remission on biologics, years	0.3 (0.1; 0.6)	0.2 (0.1; 0.3)	0.080
Flare on biologics, n (%)	22/55 (40)	11/24 (46)	0.629
Time before flare on biologics, years	1.8 (1.0; 2.7)	1.2 (0.3; 1.9)	0.100

### Uveitis

#### P480 Features of the course of uveitis in the pediatric rheumatologist practice

##### Iryna I. Shenchenko, Olena A. Oshlianska

###### Department of pediatrics, pediatric infectious diseases, immunology and allergology, Shupic National Medical Academy of postgraduate education, Kiyv, Ukraine

####### **Correspondence:** Olena A. Oshlianska

*Pediatric Rheumatology 2024*, **22(2)**: PReS24-ABS-1193

**Introduction:** Inflammatory lesions of the organ negatively affect the child's body. The annual incidence of uveitis in Ukraine is 12.4 cases per 100000 population. The exact total number and structure of autoimmune uveitis remains unknown.

**Objectives:** To analyze the clinical manifestations, structure and features of the course of isolated idiopathic uveitis and uveitis in children with rheumatic diseases.

**Methods:** A prospective observational monocentric study was conducted at the pediatric rheumatology center of the Institute of Pediatrics, Obstetrics and Gynecology of the National Academy of Medical Sciences of Ukraine over the period 2022-24. For diagnosis, the IUSG criteria (2008) were applied. Infectious and other causes of uveitis were excluded. Out of the total 97 children with uveitis, 43 children had autoimmune uveitis.

**Results:** A total number of 43 patients with uveitis were included in the study. The average age at the time of diagnosis was 10.4±years (3-17). The time from the appearance of complaints to the diagnosis was 5.5 months on average (1-12 months). In general, girls predominated among all patients with non-infectious uveitis; the ratio of boys to girls was 1:2. In half, the process was one-sided. Anterior uveitis was observed in 81 (83.5%), middle uveitis in 13 (13.4%), posterior uveitis in 3 (3.1%), none of the patients had pan uveitis. 14 (14.4%) patients had isolated idiopathic uveitis, 24 (24.7%) JIA-associated, 1 with systemic lupus erythematous , 3 with systemic vasculitis, 1 with undifferentiated connective tissue disease. JIA-associated uveitis was observed in 3 case with entesitis-arthritis, 4 with polyarthritis, the remaining cases were oligoarthritic in our center. HLA B27-positivity was observed in 9 (37.5%), ANA in 11 (45.8%), more often in JIA-associated uveitis (p=0.03). Cyclic course was noted in 9 (64.2%) patients with isolated uveitis and in 6 (25.0%) with JIA-associated. Recurrent course was observed more often (p=0.049) in uveitis associated with JIA and other rheumatic diseases. DMARDs were prescribed for 3 (21.4%) isolated uveitis, 19 (79.2%) for JIA-associated, and in all cases uveitis in cases with other rheumatic diseases. bDMARDs were used in 7 (29.1%) cases of JIA-associated uveitis, in 2 (14.2%) cases of isolated uveitis, and in 3 (60.0%) cases of other rheumatic diseases (due to the general activity of the disease). 3 (12.5%) cases with uveitis had complications in the form of cataracts, 7 (29.3%) - corneal dystrophy. Complete loss of vision was not observed among the examined patients, which did not correspond to the data of studies of previous years in Ukraine (12 per 10,000 population of blindness) and could indicate the improvement of diagnostics and treatment in recent decades.

**Conclusion:** Isolated uveitis do not have a more favorable course than those associated with rheumatic diseases. As a rule, with idiopathic uveitis, the etiology of the disease is not always established, which complicates the treatment process and increases the frequency of relapses.

**Patient Consent**

Not applicable (there are no patient data)

**Disclosure of Interest**

None Declared

**References**

1. Збітнева СВ. Захворюваність населення України на хвороби ока та його придаткового апарату. Вісн. соц. гігієни та орган. охорони здоров’я України. 2010;3:14–8.
2. Zbytneva SV. [Morbidity in Ukraine on eye disease and its adnexa]. Bulletin of Social Hygiene and Health Protection Organization of Ukraine. 2010;3:14–8. Ukrainian.

### Uveitis

#### P481 Evaluation of the timing and management of uveitis onset in Juvenile idiopathic arthritis patients

##### Sıla Atamyıldız Uçar ^1^, Betül Sözeri^1^

###### ^1^Pediatric Rheumatology, Umraniye Training and Research Hospital, Istanbul, Türkiye

####### **Correspondence:** Sıla Atamyıldız Uçar

*Pediatric Rheumatology 2024*, **22(2)**: PReS24-ABS-1760

**Introduction:** Juvenile idiopathic arthritis (JIA) is the most common chronic arthritis in children, and uveitis is its most prevalent extraarticular manifestation. Risk factors for uveitis include positive antinuclear antibodies (ANA), oligoarticular JIA type, early onset of JIA. Most cases of the JIA- associated uveitis develop within the first four years following the diagnosis of JIA.

**Objectives:** To evaluate the timing of uveitis development onset and under which treatment uveitis develops in JIA cases.

**Methods:** This cross-sectional retrospective study included a total of 115 patients with JIA and uveitis who were followed up in the pediatric rheumatology department of Umraniye Training and Research Hospital. Patients who had uveitis findings in their initial ophthalmologic examination were excluded. A total of 74 patients who first presented with arthritis with a normal initial ophthalmologic examination and later developed uveitis were included in the study. We evaluated the timing of uveitis onset and the treatments the patients were receiving at the time of their initial uveitis development.

**Results:** Among 74 JIA uveitis cases, 52 (70.3%) were female. The median age at the onset of symptoms was 2.79 years (IQR 1.8, 7.3), the median age at JIA diagnosis was 3.2 years (IQR 1.9, 10.2) and the median age at the onset of uveitis was 6.2 years (IQR 4.3, 10.25). The median follow-up duration was 64.5 months (IQR 42, 74). According to JIA subgroups, 61 (82.4%) were oligoarticular, 9 (12.2%) were enthesitis-related arthritis, and 4 (5.4 %) were diagnosed with psoriatic arthritis. The ANA positivity rate in the entire group was found to be 67.9%. The median time from JIA diagnosis to the development of uveitis was 23 months (IQR 11, 40).

All patients received DMARDs, only methotrexate, and 56 (75.7%) received biological DMARDs. The median age at DMARD initiation was 4.25 years (IQR 1.94, 7.37) and the median age at biologic agent initiation was 6.4 years (IQR 4.2, 9.4).

Forty-two (56.7%) patients developed uveitis while on methotrexate therapy and the median time from JIA diagnosis to uveitis onset was 15.5 months (IQR 6.7, 35.7). The median time for uveitis development under methotrexate was 13.5 months (IQR 12.2, 26.2). Eight of these patients were on methotrexate tapering, with a median tapering duration of 4.5 months (IQR 4, 9.5). Seventeen patients were in the methotrexate cessation period, with a median drug-free period of 11 months (IQR 4.5, 30).

Nineteen patients (34%) needed biologic treatment for arthritis symptoms, with etanercept as the first choice. 17 of these patients developed uveitis while under etanercept therapy, 14 of whom were also on concomitant methotrexate therapy. The median time of methotrexate was 26 months (IQR 16, 38) and concomitant etanercept treatment was 9 months (IQR 5, 14). Two patients were switched to infliximab and adalimumab due to unresponsiveness to etanercept for arthritis symptoms and later developed uveitis. Both patients were boys with HLA B27 positive enthesitis-related arthritis. 37 (66%) patients needed biologic treatment for uveitis findings, with 91% receiving adalimumab and 9% receiving infliximab as the first choice.

**Conclusion:** This study highlights the importance of monitoring uveitis development in JIA patients and identifies the timing and treatments under which uveitis is likely to develop. The findings emphasize the need for regular ophthalmologic examinations, even after periods of treatment discontinuation.

**Date of birth::** avril 25,

**Patient Consent**

Yes, I received consent

**Disclosure of Interest**

None Declared

### Uveitis

#### P482 Evolving treatment strategies and outcomes in Juvenile idiopathic arthritis and associated uveitis: a ten-year retrospective study

##### Esra Baskın^1^, Meraj A. Siddiqui ^2^, Sirel G. Güngör^3^, Yonca Aydın Akova^3^

###### ^1^Department of Pediatric Rheumatology, ^2^Department of Pediatrics, ^3^Department of Ophthalmology, Başkent University Medical Faculty, Ankara, Türkiye

####### **Correspondence:** Meraj A. Siddiqui

*Pediatric Rheumatology 2024*, **22(2)**: PReS24-ABS-1567

**Introduction:** Juvenile Idiopathic Arthritis (JIA) is the most prevalent pediatric rheumatic disease, characterized by joint inflammation and diverse clinical manifestations. Associated uveitis, a serious complication of JIA, can lead to significant morbidity and potentially sight-threatening conditions such as cataracts, glaucoma, and band keratopathy, ultimately resulting in vision loss. Understanding the interaction and management of JIA with uveitis is crucial for improving patient outcomes.

**Objectives:** This study aims to assess the prevalence of JIA-associated uveitis and evaluate the effectiveness of various management strategies to enhance patient care and outcomes.

**Methods:** This single-center retrospective study included pediatric patients aged 2-18 years diagnosed with JIA and its associated complication, uveitis, at Department of Pediatric Rheumatology, during the years 2013 to 2023. Patients were retrospectively evaluated for demographic and clinical features. Additionally, the study assessed the changes in treatment modalities over the decade, examining shifts in pharmacological strategies, and protocol adjustments based on current guidelines.

**Results:** The study included 80 patients (Male/Female: 28/52) with a mean age of 8.77 ± 4.19 years. The most common subtype was oligoarticular JIA (49 patients, 61.25%), followed by polyarticular JIA (18 patients, 22.5%), systemic JIA (8 patients, 10%), and enthesitis-related and psoriatic JIA (5 patients, 6.25%). Chronic uveitis was observed in 42 patients (52.5%). Initially, treatment primarily involved DMARDs and systemic steroids, administered to 90.0% and 85.0% of the patients, respectively. Due to significant side effects, low efficacy, and intolerance to DMARDs and systemic steroids, newer biologic agents (BAs) such as anti-TNFα, most commonly adalimumab, followed by infliximab and golimumab, have become increasingly important. In our cohort, 48 patients (60.0%) adopted these anti-TNFα drugs over time. Adalimumab, used in conjunction with methotrexate, demonstrated superior effectiveness. For patients with systemic JIA, anti-IL-1 therapies such as Anakinra and Canakinumab were the treatments of choice. Concurrently, the long-term use of systemic steroids decreased drastically, as did the ophthalmic-related complications associated with these drugs. Patients resistant to anti-TNF therapy required switching to Abatacept for 2 (2.5%) and Tocilizumab for 2 (2.5%) of the patients. Notably, complications related to BAs were less frequent compared to those associated with DMARDs and systemic steroids.

**Conclusion:** This study highlights the evolving landscape of treatment modalities for JIA and associated uveitis, underscoring the importance of adapting therapeutic strategies based on patient responses and emerging research. The shift towards BAs reflects a significant advancement in managing side effects and improving patient outcomes. Continued research and a multidisciplinary approach are essential to refining treatment protocols and enhancing the quality of life for affected children.

**Patient Consent**

Not applicable (there are no patient data)

**Disclosure of Interest**

None Declared

### Uveitis

#### P483 Is there a relationship between the CDMARDS and bdmards used and uveitis?

##### Sinan Işık^1^, Selen Duygu Arik^2^, Özlem Akgün^2^, Gülşah Kavrul Kayaalp^2^, Şeyma Türkmen^3^, Betül Sözeri^3^, Nuray Aktay Ayaz^2^

###### ^1^Department of Pediatrics; ^2^Department of Pediatric Rheumatology, Istanbul University Istanbul Faculty of Medicine; ^3^Department of Pediatric Rheumatology, Umraniye Training and Research Hospital, Istanbul, Türkiye

####### **Correspondence:** Selen Duygu Arik

*Pediatric Rheumatology 2024*, **22(2)**: PReS24-ABS-1677

**Introduction:** Uveitis is a prevalent complication of juvenile idiopathic arthritis (JIA), leading to significant ocular morbidity. While biological therapies are vital in managing uveitis and minimizing complications, some patients may still develop it despite conventional and biological treatments.

**Objectives:** In this study, it was aimed to evaluate the frequency of uveitis development among JIA patients receiving biological treatment, explore the impact of biological agent selection on uveitis occurrence in these individuals, and analyze treatment patterns and responses following the onset of uveitis. Additionally, we aimed to investigate the frequency of uveitis development in patients undergoing methotrexate treatment or after discontinuation of methotrexate, and assess the treatment patterns and response to therapy in these patients as well.

**Methods:** In this multicenter retrospective study, the records of 2385 patients followed-up with a diagnosis of JIA were examined. Among them, 101 patients initially diagnosed with JIA without uveitis, but later developed uveitis during the follow-up period, were further included in the analysis. This patients were categorized based on their uveitis development: under methotrexate treatment (group 1), after discontinuation of methotrexate treatment (group 2), and under biological treatment (group 3). Each group of patients underwent evaluation for their demographic and clinical characteristics, treatments received before the onset of uveitis, treatments initiated for uveitis, and their responses to these treatments, including the necessity for a switch in biological agents.

**Results:** The uveitis frequency among all reviewed JIA patients was 6.3%. In Group 1 (55 patients developing uveitis during methotrexate treatment), adalimumab was initiated in 40 patients, tocilizumab in 1, and infliximab in 1. Among those starting adalimumab, 36 achieved remission, with 3 switching to infliximab. The patient initially on infliximab achieved remission after switching to adalimumab. Remission was observed in the patient treated with tocilizumab. In Group 2 (15 patients developing uveitis after discontinuing methotrexate), adalimumab was initiated in 8 patients, infliximab in 2, and 5 resumed methotrexate. Two patients receiving adalimumab required a switch to infliximab, while one patient on infliximab needed to switch to adalimumab. Patients who resumed methotrexate treatment experienced remission. In Group 3 (31 patients developing uveitis during biological treatment), 30 were under etanercept treatment and 1 were under infliximab. When the entire cohort of JIA patients treated with biologics due to joint symptoms was evaluated, it was observed that out of 365 patients using etanercept, uveitis developed in 30 individuals, in 1 out of 39 patients using infliximab, while no cases of uveitis were observed among the 285 patients using adalimumab. Among patients who developed uveitis on etanercept, 27 switched to adalimumab, 1 to tocilizumab, and 1 to infliximab, resulting in remission for 21 patients following the change in medication. In the case of the patient who developed uveitis while on infliximab, remission was achieved after initiating adalimumab.

**Conclusion:** Although etanercept effectively treats arthritis in JIA, it's not the preferred choice for managing JIA-associated uveitis. Our study, in line with previous reports, documents uveitis development during etanercept treatment. We also noted specific treatment initiation and response patterns post-uveitis development. Further extensive and prospective randomized controlled trials are needed to assess the impact of biological therapies on uveitis.

**Date of birth::** octobre 30

**Patient Consent**

Not applicable (there are no patient data)

**Disclosure of Interest**

None Declared

### Uveitis

#### P484 Non-infectious ocular pathology in a newly started pediatric rheumatology unit

##### Estefania Pardo Campo ^1^, Stefani Burger^1^, Sara Murias Loza^2^, Paloma Rozas Reyes^3^, Carmen Costales ^3^, Norma Callejas ^1^, Paula Alvarez Peñalba^1^, Pablo Gonzalez del Pozo^1^, Julian Rodriguez^4^

###### ^1^Rheumatology; ^2^Pediatrics; ^3^Ophthalmology; ^4^Hospital Universitario Central de Asturias, Oviedo, Spain

####### **Correspondence:** Estefania Pardo Campo

*Pediatric Rheumatology 2024*, **22(2)**: PReS24-ABS-1251

**Introduction:** Inflammatory ocular pathology frequently accompanies systemic autoimmune diseases, but it can occur in the absence of other clinical manifestations. The best known is uveitis associated with juvenile idiopathic arthritis (JIA) as it is the extra-articular manifestation, without forgetting that there are other rheumatic entities that cause ocular involvement.

**Objectives:** To analyse the frequency and characteristics of patients diagnosed with non-infectious ocular pathology either associated or not with JIA, in a cohort of patients followed in the Pediatric Rheumatology Unit of the Central University Hospital of Asturias (HUCA).

**Methods:** Retrospective, descriptive, observational study including pediatric patients diagnosed with non-infectious ocular pathology (with/without JIA) under follow-up by the HUCA Pediatric Rheumatology Unit from October 2020 to March 2024.

**Results:** Twenty-four patients were included, 18 (70.8%) women.

There were 22 patients diagnosed with any type of uveitis (17 anterior uveitis, 1 intermediate uveitis, 1 posterior uveitis, 3 panuveitis), 12 uveitis associated with JIA.

Patients with uveitis not associated with JIA were 10, of which 6 uveitis were idiopathic (3 associated with HLA B27, 1 idiopathic granulomatous, 1 pars planitis, 1 pure idiopathic). The other 4 patients were; 2 autoinflammatory origin ( Blau Syndrome and early onset sarcoidosis, both granulomatous), 1 paraneoplastic (lymphoma), 1 orbital pseudotumor.

3 patients developed ocular involvement in the form of nodular sclerosis (1) and episcleritis (2).

The median age at last follow-up was 8 years (range 2-18). The mean age at onset of JIA was 4.7 years, with a median of 2 (range 0.5-15).

Of the 10 patients with JIA-associated uveitis, 100% had ANA+ and 3 of them had ocular involvement as a form of JIA onset. At the last follow-up visit, of the 24 patients, 70.8% (17) were undergoing biological treatment and 62.5% (15) were on methotrexate (MTX). Of the patients, 58.3% (14) had received systemic corticosteroid therapy at some time during their evolution.

**Conclusion:**

Non-infectious ocular pathology in paediatric rheumatology consultations encompasses a wide range of entities beyond JIA-associated uveitis. It should not be forgotten that uveitis may be the presentation of JIA. The early and targeted introduction of biological treatments has been a revolution in inflammatory diseases that are difficult to control, including uveitis. Multidisciplinary management between paediatric rheumatology and ophthalmology is essential for early diagnosis and close follow-up.

**Patient Consent**

Not applicable (there are no patient data)

**Disclosure of Interest**

None Declared

**References**

1. Maleki A, Patel PD, Foster CS. Juvenile idiopathic arthritis and its associated uveitis. Expert Rev Clin Immunol. 2023 Jul-Dec;19(9):1157-1169. doi: 10.1080/1744666X.2023.2231154. Epub 2023 Jul 4. PMID: 37401872.
2. Paroli MP, Del Giudice E, Giovannetti F, Caccavale R, Paroli M. Management Strategies of Juvenile Idiopathic Arthritis-Associated Chronic Anterior Uveitis: Current Perspectives. Clin Ophthalmol. 2022 May 28;16:1665-1673. doi: 10.2147/OPTH.S342717. PMID: 35663189; PMCID: PMC9159812.

### Uveitis

#### P485 Refractory panuveitis a silent cause for blindness

##### Awatif A. Abushhaiwia, Yusra AElfawires

###### Paediatric Rheumatology , Tripoli Children's Hospital, faculty of medicine, university of Tripoli, Tripoli , Libya

####### **Correspondence:** Awatif A. Abushhaiwia

*Pediatric Rheumatology 2024*, **22(2)**: PReS24-ABS-1047

**Introduction:** Panuveitis, also known as Diffuse uveitis, is the inflammation of all uveal componentsof the eye with no site of predominant inflammation. (“Panuveitis - EyeWiki”). In many cases the cause is unknown, but it may occur in association with other systemic and eye diseases.Treatment options include non-specific such as steroids, immune suppressants and,biological agents as well as, specific for the underlying cause. And it is well known to be a major cause for blindness and morbidity.

**Objectives:** To highlight the importance of screening and early diagnosis for suspected cases as well as, introduction of proper management plan according to severity and cause. Minimizing the risk ofblindness and eye morbidity.

**Methods:** A retrospective study for two patients’ files.

**Results: Patient #1**.**A** six-year-old boy, born to non-consanguineous parent and no family history of note. Presented with skin nodules -scalp, hands, and feet. His skin biopsy showed evidence of rheumatoid nodules Later he developed red eyes, with evidence of bilateral granulomatous uveitis.After which he was diagnosed to have sarcoidosis.Unfortunately, he was referred to Rheumatology clinic after three years with evidence of refractory bilateral panuveitis in addition to macular oedema and synechia formation,his systemic review is unremarkable.His all blood tests/serology &images were normal/neg.Due to issues regarding biological medication availability, he received infliximab total 6 doses- after receiving pulses of methylprednisolone infusion, oral steroids and MTX injections with history of intermittent courses of oral and local steroids showing no improvement.Currently he is following at a Rheumatology/Ophthalmology joint clinic and has been shifted to Emgevita (adalimumab) SC 20mg on 9^th^ of April 2024 hoping for better control.

**Patient #2.A** ten-year-old girl, born to non-consanguineous parents.Her family history and past medical history are unremarkable. She presented with red eyes, blurred vision, and photophobia for four months. She was diagnosed to have isolated bilateral panuveitis and started with local and systemic steroids.Initially she showed some response to medication for one year but, on attempts for tapering steroids she shows relapse and flaring of symptoms.Her blood tests/serology &images were all normal/neg.Eye examination: Visual acuity was 0.7 and 0.8 in the right & left eye respectively. IOP was 12 in both eyes. Slit lamp bio microscopy of both eyes revealed 3+ anterior chambers (AC) cells.The patient has 2+ vitreous cells in both eyes. Synechia in right.OCT revealed panuveitis & optic oedema.When referred to Rheumatology clinic she started MTX inj in addition to local and systemic steroids.Due to lack of biological medication after her last relapse she was started on MMF 600mg/m^2^/day.

**Conclusion:** Blindness and eye morbidities are the result of refractory panuveitis. Rasing awarenessabout the importance of early access to proper medical service and biological medication plays amajor role in the outcome.

**Patient Consent**

Yes, I received consent

**Disclosure of Interest**

None Declared

### Uveitis

#### P486 Observation of patients with blau and vogta-koyanagi-harada syndrome

##### Akhenbekova Aida^1^, Gulsara Rakhym^2^

###### ^1^Department of Children Diseases; ^2^Department of Rheumatology, Kazakh National Medical University named after S.D.Asfendiyarov, Almaty, Kazakhstan

####### **Correspondence:** Akhenbekova Aida

*Pediatric Rheumatology 2024*, **22(2)**: PReS24-ABS-1684

**Introduction:** Uveitis in children is a serious pathology in pediatrics that has a polyetiological nature and is often accompanied by autoimmune and infectious diseases. The importance of early diagnosis of uveitis is associated with the risk of developing complications such as cataracts, glaucoma, and retinal detachment. A long stage of differential diagnosis reduces therapeutic options, which is most important for autoimmune uveitis of a genetic nature. In this study, we present a clinical observation of a child with Vogt-Koyanagi-Harada syndrome (VKH), which is rarely encountered in pediatric practice.

**Objectives:** To describe the clinical observation of a 5-year-old child with VKH syndrome during the use of combination therapy of adalimumab and methotrexate.

**Methods:** Clinical observation included ophthalmological and general clinical examination, determination of antinuclear factor, and ultrasound examination of the abdominal cavity.

**Results:** The first symptoms appeared at the age of 3 in the form of red eyes and photophobia. Based on a positive result for HSV, Mucopurulent conjunctivitis of infectious origin was diagnosed. The child received long-term treatment. Systemic skin manifestations: depigmented spots on the face, body, upper and lower extremities, graying of hair and eyelashes, appeared later. Confluent spots of vitiligo reached a large area of ​​​​damage in the shoulders, abdomen, torso, and forearms. In 1.5 years, when a sharp decrease in vision, band-like degeneration of the cornea, clouding in the superficial and middle layers of the eye, the progression of glaucoma, iris atrophy, and synechia were recorded, Vogt-Koyanagi-Harada syndrome was diagnosed. VIS OD = 0.005, VIS OS = 0.01. Systemic glucocorticoid therapy was carried out at a rate of 15 mg per kilogram of weight with transfer to oral administration of 1 mg per kilogram of weight with gradual withdrawal of the drug. There was a violation of compliance with therapy; initiation of therapy with adalimumab was started 2.5 years from the onset of the disease in combination with methotrexate.

Clinical observation over the next 1.5 years showed that combination therapy with methotrexate and a TNF inhibitor provided clinical improvement in ocular symptoms and relief of the inflammatory process. The dynamics of the skin syndrome and the partial restoration of melanin in the patient’s skin and hair were interesting. However, the child continued to have eccentric vision and corneal opacities. To restore visual function in one eye, corneal transplantation was successfully performed, increasing visual acuity to 0.1.

**Conclusion:** Therapy with a TNF inhibitor and methotrexate has shown effectiveness in a child with VKH syndrome. Early diagnosis and adequate immune-mediated treatment of a patient with autoimmune uveitis can prevent complications and invalidization. The patient is under observation and a corneal transplant is planned in the second eye.

**Patient Consent**

Yes, I received consent

**Disclosure of Interest**

None Declared

### Uveitis

#### P487 Characteristics of patients with uveitis associated with JIA in a center in Nicaragua

##### Karla V. Jirón Mendiola

###### Pediatrics, Hospital Vivian Pellas, MANAGUA, Nicaragua

*Pediatric Rheumatology 2024*, **22(2)**: PReS24-ABS-1709

**Introduction:** Juvenile idiopathic arthritis (JIA) is the most common chronic rheumatic disease in childhood and is of unknown etiology. The percentage of uveitis is higher among children with extended oligoarticular JIA. Several risk factors for presenting uveitis in JIA have been reported: JIA subtype, female sex, early age of onset of arthritis, and positive antinuclear antibodies (ANA). These factors are reflected in the follow-up guidelines of the American Academy of Pediatrics, where the ANA result, the subtype of JIA, and the age of onset guide the recommended frequency of follow-up for uveitis in children with juvenile idiopathic arthritis.

Among children with JIA-associated uveitis, the following risk factors have been associated with an increased risk of complications and/or disease severity: complications at the onset of disease presentation, male sex, disease severity, cellularity ≥ 1 + in the anterior chamber at the presentation of the disease, flare ≥ 1 + in the anterior chamber, ANA positive, short duration between the diagnosis of arthritis and uveitis and the presentation of uveitis prior to arthritis. In Nicaragua, especially in our center, the majority of children who have attended present complications such as total loss of vision, this due to a late referral or due to their phenotype, which is why the study was created to describe the characteristics of the patients

**Objectives:** To describe the characteristics of the patients with uveitis asociated to JIA in a center of Nicaragua

**Methods:** 15 patientes with JIA were includen in the study

**Conclusion:** The majority of patients were female, had extended oligoarticular juvenile idiopathic arthritis, in addition to antinuclear antibodies, all had elevated acute phase reactants, such as ESR and CRP, they also came from distant communities and had been previously treated with non-specialized ophthalmological therapies. found cataracts, glaucoma and band keratopathy mainly.

**Date of birth::** février 23

**Patient Consent**

Yes, I received consent

**Disclosure of Interest**

None Declared

### Vasculitides

#### P488 Cogan syndrome in children: clinical characteristics predicting prognosis and therapeutic regimens

##### Jianghong Deng^1^, Caifeng Li^1^ on behalf of Xue Yuan, MD, PhD; Kuang Weiying, MD, Zhang junmei, MD, Tan Xiaohua, MD, Li Chao, MD, Li Shipeng, MD, Liu Xuanyi, MD, PhD

###### ^1^Beijing children's hospital, Beijing, China

####### **Correspondence:** Caifeng Li

*Pediatric Rheumatology 2024*, **22(2)**: PReS24-ABS-1108

**Introduction:** Cogan syndrome (CS) is a rare chronic inflammatory disease characterized by general, ocular, and auditory-vestibular symptoms. Due to the rarity of CS, it is often overlooked and misdiagnosed especially in Children.

**Objectives:** This article aims to summarize the clinical presentations of CS patients at a tertiary hospital and review the clinical features, diagnosis, treatment, and prognosis of pediatric Cogan syndrome reported in the literature. The goal is to enhance the understanding of this rare disease among pediatricians and explore the relevant factors influencing the prognosis of CS.

**Methods:** A retrospective study was conducted in which patients who met the diagnostic criteria for CS with age of onset <18 years were enrolled. A comprehensive review of the literature on pediatric CS was performed by searching for relevant articles. The disease characteristics, course, clinical manifestations, treatment approaches, and follow-up prognosis of this condition was analyzed. Clinical manifestations frequently found in CS were analyzed to fit a predictive model for positive response to treatment with the method of eXtreme Gradiet Boosting (XGboost) algorithm and Random Forest (RF)method. Model discrimination, calibrationcurve analysis were also performed for validation.

**Results:** A total of 39 pediatric patients with Cogan syndrome were included in this study, comprising 3 patients admitted to our hospital and 36 cases reported in the literature. The cohort consisted of 20 males and 19 females, with a male-to-female ratio of 1.05:1. The median age of onset was 11 years (range: 3-17 years), and the median age at diagnosis was 12 years (range: 4-21 years). Among the 39 patients, all had varying degrees of ocular symptoms (100%), Thirty-six patients had auditory symptoms (92.3%), Systemic symptoms were observed in 21 patients (53.8%). Cardiovascular symptoms were present in 7 patients (17.9%). Steroid pulse therapy followed by maintenance treatment with adequate doses of steroids, immunosuppressants (methotrexate, switched to azathioprine in case 3 due to poor response), and biologics (infliximab) were administered in all three patients.

A total of 152 articles published between 1954 and 2023 were searched, and research subjects were selected based on the inclusion and exclusion criteria. Ultimately, data from 36 patients in 28 articles were included for analysis . The main clinical manifestations, course, and outcomes of pediatric Cogan syndrome reported in the literature are summarized in Table 3. Thirty-one patients had regular follow-up (79.5%) with a median follow-up duration of 2 years and 8 months after immunosuppressants and steroid treatment. Five patients achieved complete remission (12.8%), 12 patients showed improvement (30.8%). The clinical manifestations frequently found in CS of the 39 patients were analyzed using the XGBoost algorithm. 10 variables with a SHARP value>0.3 were selected in the predictive model for positive response to treatment.

**Conclusion:** Early diagnosis and intervention are crucial for the prognosis of Cogan syndrome in children. Timely administration of immunosuppressive agents, including methotrexate, can significantly improve outcomes. The XGBoost model demonstrates high efficacy in predicting treatment improvement and identifying relevant factors.

**Trial registration identifying number:** none

**Patient Consent**

Yes, I received consent

**Disclosure of Interest**

None Declared

### Vasculitides

#### P489 Journey into the genetic realm: investigating childhood vasculopathy as a monogenic vasculitis through a comprehensive systematic review

##### Shahad A. Alansari^1^, Reima Bakry^2^, Fatimah Alkhars^3^, Wafaa Abdulghafaar^4^, Sulaiman Almayouf^4, 5^

###### ^1^Pediatric Rheumatology, Heraa General Hospital, Makkah; ^2^Pediatric Rheumatology, Maternity and Children Specialized Hospital, Jeddah; ^3^Pediatric Rheumatology, Maternity and Children Hospital, Al-Hassa; ^4^Pediatric Rheumatology, King Faisal Specialist Hospital and Research Center; ^5^Alfaisal University, College of Medicine, Riyadh, Saudi Arabia

####### **Correspondence:** Shahad A. Alansari

*Pediatric Rheumatology 2024*, **22(2)**: PReS24-ABS-1218

**Introduction:** Monogenic vasculitis is a rare form of vasculitis that has recently been used to describe the deficiency of adenosine deaminase 2 (DADA2) and STING-associated vasculopathy of infancy (SAVI). Unlike sporadic systemic vasculitis, monogenic vasculitis is caused by specific genetic mutations that disrupt the immune system's normal function. In this review, we will discuss the clinical manifestations, genetic basis, and the response to available treatment options not only concerning vasculitis in systemic autoinflammatory syndromes but also vasculitis in inborn error of immunity (IEI) in general.

**Objectives:** To review the literature on the genotypic and phenotypic features of childhood vasculopathy, supporting the proposal of monogenic vasculitis as a new entity.

**Methods:** This is a comprehensive systematic review of the literature conducted by electronic searches via PubMed and Google Scholar for relevant studies using the appropriate MeSH terms related to the clinical features of systemic vasculitis and associated genetic variants. The search was limited to articles from January 1990 till March 2024. Authors independently reviewed the searched literature to identify eligible studies. Citations from selected articles were also checked for additional eligible studies.

**Results:** Of the 239 initially reviewed studies, only 85 articles met the eligible entry criteria. Of those, 219 patients (117 females) had a genetically proven diagnosis and were selected for analysis. One hundred ten patients with deficiency of adenosine deaminase 2 (ADA2), twenty patients with Haploinsufficiency of A20 (TNFAIP3), thirty-two patients with STING-associated vasculopathy with onset in infancy (TMEM173), four patients with Activated Phosphoinositide 3-Kinase Delta Syndrome (PIK3CD), twenty-two patients with NF-kB pathway abnormalities (NFKB1, RELA), two with familial Mediterranean fever (MEFV) and three with Blau syndrome (NOD2). Twenty-six patients had other rare inborn error of immunity affected genes (1= STAT1, 1= IL-2Rγ, 1= LRBA, 1= FIBN 1, 1= XIAP, 2= TYK2, 3= CBL, 4= WAS, 1= AICDA, 1= IKZF1, 1=COPA, 1=RAG1, 1=LIG4, 1=NLRC3, 2= DOCK8, 1= FOXP3, 1= KDM5B, 1= Ch 22q11, 1=SLC27A6). For the reported cases, the median of disease onset was (5.3) years (IQR: 3.9-12); consanguinity and positive family history were detected in 39 patients and 81 patients (17%) and (36.9%), respectively. Recurrent fever, central nervous system (CNS), and mucocutaneous manifestations were the most reported clinical manifestations, accounting for (58.9%), (46.1%) and (74.8%), respectively. Complete blood count abnormality and elevated inflammatory markers were reported in (76.2%) and (47.4)%, respectively. All patients were treated aggressively either with corticosteroids, conventional, biological disease-modifying antirheumatic drugs or a combination with variable responses. Twelve patients underwent haematopoietic stem cell transplantation. There were fifteen deaths.

**Conclusion:** This study shows that various inherited disorders may contribute to childhood vasculitis. Strong genetic evidence and familial clustering may enable us to broaden the proposed monogenic vasculitis beyond DADA2 and SAVI.

**Patient Consent**

Not applicable (there are no patient data)

**Disclosure of Interest**

None Declared

### Vasculitides

#### P490 Evaluation of pediatric immunoglobulin a vasculitis with gastrointestinal tract involvement: normal vs abnormal abdominal ultrasound

##### Merve Cansu Polat^1^, Zahide Ekici Tekin^1^, Cüneyt Karagöl^1^, Banu Çelikel Acar^1^

###### ^1^Pediatric Rheumatology, Ankara Bilkent City Hospital, Ankara, Türkiye

####### **Correspondence:** Merve Cansu Polat

*Pediatric Rheumatology 2024*, **22(2)**: PReS24-ABS-1262

**Introduction:** Gastrointestinal (GI) tract involvement is observed in 50-75% of patients with immunoglobulin A vasculitis (IgAV). Abdominal ultrasound (US) is the most commonly used diagnostic tool to assess the severity and complications of GI inflammation in symptomatic patients [1].

**Objectives:** The aim of this study was to compare the clinical, demographic and laboratory data of IgAV patients with GI symptoms with and without normal abdominal US.

**Methods:** A total of 187 IgAV patients with GI tract involvement who were followed up for at least 3 months were included in the study. Clinical, laboratory, and radiological data of the patients were analyzed from electronic file records. The pediatric vasculitis activity score (PVAS) was used to evaluate disease activity.

**Results:** Abdominal US was normal in 69 (36.9%) patients at the onset of symptoms. In the control US of these patients, which was performed a mean of 2.6±1.3 days after the first US, 26 (37.6%) patients had abnormal findings suggestive of GI involvement, such as intestinal wall thickening and edema. Patients were divided into 2 groups as those with a normal initial and control abdominal US (Group 1, n=43) and at least one pathological US (Group 2, n=144). Gender (*p*=1), age at diagnosis (*p*=0.9) and duration of GI symptom development (*p*=0.32) were similar in both groups. The duration of hospitalization was longer in group 2 (*p*=0.001). There was no difference between the symptoms on admission and GIS symptoms, articular, renal, and scrotal involvement during follow-up. Massive GI tract hemorrhage was observed in 7 (16.3%) patients in Group 1 and 27 (18.8%) patients in Group 2 (*p*=0.82) (Table 1). All patients in Group 1 and 97.9% in Group 2 received steroid treatment. Pulse steroid was more commonly prescribed in Group 2, whereas patients in Group 1 were treated with a 2 mg/kg/day steroid (*p*=0.001). The mean duration of steroid usage was 44.9±47.1 days in Group 1 and 55±52.8 days in Group 2 (*p*=0.002). Cyclophosphamide was initiated in one (2.3%) patient in Group 1 due to massive GI tract hemorrhage. In Group 2, 14 (9.7%) patients received cyclophosphamide, 10 (6.9%) received intravenous immunoglobulin, and 2 (1.4%) underwent plasmapheresis. At the time of diagnosis, the median PVAS was 3 (IQR:2-3) in both groups (*p*=0.58).

**Conclusion:** GI complications are one of the most important causes of morbidity and mortality in the early stages of the disease. In some patients with severe GI tract involvement, intestinal wall abnormalities may not be demonstrated on US [2,3]. Therefore, treatment should not be delayed in patients with GIS complaints, even if abdominal US is normal.

**Date of birth::** juin 09, Y

**Patient Consent**

Not applicable (there are no patient data)

**Disclosure of Interest**

None Declared

**References**

1. Ebert EC. Gastrointestinal manifestations of Henoch-Schonlein Purpura. Dig Dis Sci. 2008;53(8):2011-9. doi: 10.1007/s10620-007-0147-0.
2. Rubino C, Monacelli C, Marrani E et al. Gastrointestinal involvement in IgA vasculitis: a single-center 11-year study on a cohort of 118 children. Clin Rheumatol. 2021;40(12):5041-5046. doi: 10.1007/s10067-021-05863-9.
3. Li Y, Zhang X, Liu H et al. Severe gastrointestinal involvement in pediatric IgA vasculitis: a retrospective single-center cohort study in China. Front Pediatr. 2023;11:1194214. doi: 10.3389/fped.2023.1194214.

### Vasculitides

#### P492 The exploration of the relationship between disease activity and neutrophil extracellular traps (NETOSIS) findings in pediatric immunoglobulin a vasculitis (IGAV) diagnosed patients

##### Vafa Guliyeva^1^, Fatma G. Demirkan^1^, Erdem Bektaş^2^, Rabia Deniz^3^, Ahmet Gül^2^, Nuray Aktay Ayaz^1^

###### ^1^Department of Pediatric Rheumatology, ^2^Department of Internal Medicine, Istanbul University Istanbul Faculty of Medicine, ^3^Department of Genetics, Istanbul University, Aziz Sancar Experimental Medicine Research Institute, istanbul, Türkiye

####### **Correspondence:** Vafa Guliyeva

*Pediatric Rheumatology 2024*, **22(2)**: PReS24-ABS-1631

**Introduction:** IgA vasculitis leads to a skin rash, arthritis, and affects the gastrointestinal and renal systems. Neutrophil extracellular traps (NETs) are believed to play a role in autoimmune conditions such as IgA vasculitis.

**Objectives:** We aimed to investigate the relationship between NETosis findings and disease activity in IgA vasculitis

**Methods:** A total of 33 patients under the age of 18 newly diagnosed with IgA vasculitis, along with 26 healthy individuals, were included in our study.Levels of cf-DNA, NE, MPO and cit-H3, which are NETosis markers, were measured in serum and urine samples from patients who were followed cross-sectionally and longitudinally during active and remission periods.s.

**Results:** Fifteen of 33 (44.1%) of the patient group were male, the mean age was 7.97 ± 3.03 years. 11 of the healthy control group were male (42%), the mean age was 9.36 ± 3.63 years. There was no significant difference in gender and age between the two groups. Serum cf-DNA level was significantly higher in the active patient group than in the control and inactive patient groups (p=0.04; p=0.04, respectively). Serum MPO-DNA levels were higher in the control group, but no difference was observed between the disease groups. The MPO-DNA value measured in urine was significantly lower than the control group in the inactive phase (p = 0.009). Urine cit-H3 values were significantly higher in both periods of the disease compared to the control group (p = 0.01 and p = 0.03, respectively). Serum and urine NE levels showed similar results. The AUC value for serum cf-DNA was 0.654 (95% confidence interval (CI) 0.509-0.798, p=0.046), the cutoff value was 935 ng/ml with 93% sensitivity and 72% specificity.

**Conclusion:** Our study shows that NETosis in IgAV may be linked to disease activity, helping in monitoring. It may serve as a diagnostic and prognostic marker for disease management.

**Patient Consent**

Yes, I received consent

**Disclosure of Interest**

None Declared

**References**

1. Jennette JC, Falk RJ, Bacon PA, Basu N, Cid MC, Ferrario F, ve diğerleri. 2012 revize edilmiş Uluslararası Chapel Hill Konsensus Konferansı Vaskülitlerin Adlandırılması. Artrit Rheum. 2013;65(1):1-11.
2. Gardner-Medwin JM, Dolezalova P, Cummins C, Southwood TR. Farklı etnik kökene sahip çocuklarda Henoch-Schönlein purpurası, Kawasaki hastalığı ve nadir vaskülitlerin görülme sıklığı. Lancet. 2002;360(9341):1197-202.
3. Kessenbrock K, Krumbholz M, Schönermarck U, Back W, Gross WL, Werb Z, et al. Otoimmün küçük damar vaskülitinde netleşen nötrofiller. Nat Med. 2009;15(6):623-5.
4. Safi R, Kallas R, Bardawil T, Mehanna CJ, Abbas O, Hamam R, ve diğerleri. Behçet hastalığında nötrofiller, hücre dışı nötrofil tuzaklarının salınımını artırarak vaskülite katkıda bulunur. J Dermatol Sci. 2018;92(2):143-50.
5. Khandpur R, Carmona-Rivera C, Vivekanandan-Giri A, Gizinski A, Yalavarthi S, Knight JS, ve diğerleri. NET'ler sitrulinlenmiş otoantijenlerin kaynağıdır ve romatoid artritte inflamatuar yanıtları uyarır. Sci Transl Med. 2013;5(178):178ra40.
6. Chen XQ, Tu L, Zou JS, Zhu SQ, Zhao YJ, Qin YH. Nötrofil Hücre Dışı Tuzaklarının IgA Vaskülitiyle İlişkili Hastalık Aktivitesine Katılımı. Ön İmmunol. 2021;12:668974.

### Vasculitides

#### P493 Tocilizumab in childhood-onset takayasu arteritis: the experience of a federal pediatric rheumatology centre in Russia

##### Vera Podzolkova^1^, Galina Lyskina^1^, Nadezhda Podchernyaeva^1^, Olga Shpitonkova^1^, Yulia Kostina^2^, Valentina Seraya^2^, Maria Nikolaeva^2^, Anstasia Ivina^1^, Daria Dementyeva^3^

###### ^1^Department of Children's Diseases, Sechenov's University, ^2^Department of Pediatrics Rheumatology №1, Clinic of Children’s diseases Sechcenov’s Centre of Maternity and Childhood, ^3^Faculty of General Medicine, N.I. Pirogov Russian National Research Medical University, Moscow, Russian Federation

####### **Correspondence:** Vera Podzolkova

*Pediatric Rheumatology 2024*, **22(2)**: PReS24-ABS-1148

**Introduction:** Takayasu arteritis (TA) in children is characterised by an aggressive course with frequent relapses and the involvement of the abdominal aorta, renal arteries with the development of renovascular hypertension. Tocilizumab (TCZ), an IL-6 receptor inhibitor, is one of the drugs for second-line therapy of TA according to the recommendations of EULAR 2018 and ACR 2021; however, there is insufficient data on its efficacy in paediatric TA in the world literature.

**Objectives:** To evaluate the efficacy and tolerance of TCZ in children with TA observed in the federal pediatric rheumatology centre.

**Methods:** A total of 56 children with confirmed diagnosis of TA according to EULAR/PRINTO/PReS criteria of 2010 were observed in our centre from February 2001 to March 2024. Among the children, girls predominated (male/female ratio – 1:5.2), the median age of disease onset was 11.5 [9.5; 14] years, the median delay of diagnosis was 10 [3; 22.5] months. At the time of TA confirmation, the median ITAS.A was 13 [10; 15.5], and the median number of affected vessels was 4 [3; 6.5]. As first-line therapy, 53 patients received a combination of glucocorticoids with a median prednisolone equivalent dose of 1 [0.63; 1] mg/kg/day with non-biological disease-modifying drugs such as methotrexate and cyclophosphamide, and three patients also received TCZ at a rate of 8-10 mg/kg/month to this combination. The relapse-free survival rate on first-line therapy at 18 months follow-up was 52.5%.

**Results:** A total of 16 children received TCZ in our centre: in 13 children it was prescribed as a second-line therapy, in 3 children as a first-line therapy due to disease activity. The median follow-up after initiation of TCZ was 22.5 [12; 28] months. TCZ was prescribed for intravenous administration at the rate of 8-10 mg/kg/month in combination with glucocorticoids and non-biological disease-modifying drugs. After 12 months follow-up after initiation of TCZ the median glucocorticoids dose decreased from 0.43 [0.29; 0.5] mg/kg/day to 0.19 [0.16; 0.21] mg/kg/day; the median of ITAS.A decreased from 7 [5; 10] to 0 [0; 0]. There were no side effects to TCZ. Among the complications of therapy, we can note 1 case of postoperative neck phlegmon in one child. There was no activity of TA at the end of follow-up in our patients. After 21 months of TCZ use, one patient had an increase in the ITAS.A to 4 points when the glucocorticoid dose was reduced below 0.06 mg/kg/day. When the glucocorticoid dose was increased to the previous effective dose, the symptoms of activity resolved in 6 months. Five patients underwent surgical interventions for critical arterial stenoses. Another patient had a severe relapse of the disease with the transient ischemic attack, myocardial infarction, progression of renal artery stenoses and increasing renovascular hypertension against the background of problems with TCZ receipt for several months. The patient was restored to TCZ therapy and underwent surgical treatment of left renal artery stenosis; however, the child died of early postoperative complications.

**Conclusion:** TCZ proved to be an effective and safe drug to control the activity of TA and to maintain remission. Attempts to withdraw glucocorticoids may be associated with a high risk of disease recurrence according to our data. TCZ is prescribed for vital indications and cannot be discontinued without the approval of the treating rheumatologist or without adding other immunosuppressants to the treatment.

**Patient Consent**

Not applicable (there are no patient data)

**Disclosure of Interest**

None Declared

### Vasculitides

#### P494 Predictive factors of bowel involvement in pediatric patients with henoch - schoenlein purpura

##### Flora Fedele^1^, Maria Tardi^2^, Martina Peluso^1^, Andrea Catzola^2^, Andrea Uva^2^, Luigi Martemucci^2^, Francesca Orlando^2^

###### ^1^Department of Translational Medical Science, Section of Pediatrics, University of Naples “Federico II”; ^2^Pediatric Immuno-rheumatology Unit, Department of General and Emergency Pediatrics, Santobono-Pausilipon Children's Hospital, Naples, Italy

####### **Correspondence:** Flora Fedele

*Pediatric Rheumatology 2024*, **22(2)**: PReS24-ABS-1607

**Introduction:** Henoch-Schoenlein purpura (HSP) is an acute systemic small vessel vasculitis characterized by palpable purpura, arthritis, gastrointestinal and renal manifestations. Gastrointestinal symptoms may occur with the disease onset or following purpura onset, usually within a month. The presence of a severe abdominal involvement requires corticosteroid therapy, which may reduce the risk of complications if early administered.

**Objectives:** Identify any predictive factors (clinical, laboratory and instrumental) of gastrointestinal involvement in order to practice more careful monitoring and early therapeutic intervention in HSP patients.

**Methods:** A retrospective and prospective observational study was conducted on a population of pediatric patients with HSP, afferent to the Complex Operative Unit of General Pediatrics and Immunorheumatology of the AORN Santobono Pausilipon of Naples between January 2019 and September 2023. The diagnosis was made according to the 2008 Ankara criteria. Demographic data, clinical features, gastrointestinal involvement, laboratory and instrumental data were collected at T0, defined as the first assessment of the patient. All patients with gastrointestinal involvement at T0 were excluded for the analysis of predictive factors. Subsequently, the enrolled patients were divided into two groups based on gastrointestinal symptoms: absent gastrointestinal involvement (Group 1) and development of symptoms within 4 weeks (Group 2).

**Results:** Eighty-four patients were enrolled (median age at diagnosis: 5.9 years; M/F: 41/43) at T0. Male gender was associated with a higher number of clinical manifestations at onset (2.3 vs. 1.9; p=0.04). In addition, a higher percentage of males (39% vs 23.3%) presented with abdominal pain as a clinical manifestation, although without statistical significance (p=0.1). Seventy-two children were finally included in the study (median age at diagnosis: 5.2 years, range: 4.1-7.3 years; M/F: 34/38), of whom 51 patients (70.8%) were belonging to Group 1 and 21 (29.2%) to Group 2. Patients in Group 1 showed a higher age at diagnosis than those in Group 2 (5.75 years vs 4.2 years; p =0.005). Abdominal ultrasound was found to be pathological in 40% of Group 1 and 90% of Group 2 with a statistically significant difference (p<0.001). Differently, there was no statistically significant difference in the positive result of the occult test between the two groups (50% vs 68.8%; p=0.2)

**Conclusion:** As reported in literature, a positive fecal occult blood test occurred in more than half of patients, however it is not a predictive factor for gastrointestinal involvement. Instead, early age at onset and abdominal pathological ultrasound are risk factors for gastrointestinal involvement in HSP. Further investigations are needed to define the role of an early intestinal ultrasound to find patients requiring early treatments.

**Date of birth::** octobre 31

**Patient Consent**

Yes, I received consent

**Disclosure of Interest**

None Declared

### Vasculitides

#### P495 Performance Of The Eular/Printo/Pres (Ankara 2008) classification criteria for IGA vasculitis in adults

##### Yagmur Bayindir ^1^, Peter C. Grayson^2^, Katherine B. Gribbons^3^, Selcan Demir^4^, Alexandra Audemard-Verger^5^, Cristina Ponte^6^, Joanna C. Robson^7^, Ravi Suppiah^8^, Raashid A. Luqmani^9^, Richard A. Watts^10^, Peter A. Merkel^11^, Seza Ozen^1^

###### ^1^Hacettepe University Children’s Hospital, Ankara, Türkiye, ^2^National Institute of Arthritis and Musculoskeletal and Skin Diseases, Bethesda, Maryland, ^3^Brigham and Women’s Hospital, Boston, MA, United States, ^4^Eskişehir Osmangazi University Children’s Hospital, Eskişehir, Türkiye, ^5^Centre Hospitalier Régional Universitaire de Tours, Tours, France, ^6^Universidade de Lisboa, Lisboa, Portugal, ^7^University of the West of England Bristol, Bristol, United Kingdom, ^8^Auckland District Health Board, Auckland, New Zealand, ^9^University of Oxford, Oxford, ^10^University of East Anglia, Norwich, United Kingdom, ^11^University of Pennsylvania, Philadelphia, Pennsylvania, United States

####### **Correspondence:** Yagmur Bayindir

*Pediatric Rheumatology 2024*, **22(2)**: PReS24-ABS-1702

**Introduction:** The 1990 American College of Rheumatology Classification Criteria for Henoch Schönlein purpura (HSP), now IgA vasculitis (IgAV), relies on non-specific features and does not address the importance of IgA in diagnosis and pathogenesis of the disease. The 2008 EULAR/Pediatric Rheumatology International Trials Organization/Pediatric Rheumatology European Society validated classification criteria (known as the Ankara criteria) for HSP/ IgAV. The Ankara criteria have a high sensitivity and specificity in children.

**Objectives:** The aim of this study was to examine the application of the Ankara criteria to adults with IgA vasculitis (IgAV).

**Methods:** This analysis utilized data from the Diagnostic and Classification Criteria for Primary Systemic Vasculitis (DCVAS) study from patients i) with a diagnosis of IgA vasculitis; and ii) a set of comparators with polyarteritis nodosa, ANCA-associated vasculitis, cryoglobulinemic vasculitis, or a different small-vessel vasculitis. Only patients for whom the investigator was “very confident” with the diagnosis were included in the analysis. For this analysis the Ankara 2008 Criteria items were revised by adding ANCA and/or cryoglobulin positivity as an exclusion criteria, then skin involvement plus one of the following four criteria including abdominal pain, a biopsy showing IgA deposition, arthritis or arthralgia, and renal involvement (any hematuria and/or proteinuria) were required.

**Results:** The data set consisted of 178 cases of IgAV and 1705 comparators (96 PAN, 1442 AAV, 41 cryoglobulinemic vasculitis, and 126 other small-vasculitis vasculitis). When the Ankara criteria were tested in the data set, the sensitivity for classifying patients with IgAV was 91% (95% CI 86.9% to 95.1%) and the specificity was 73.4% (95% CI 71.3% to 75.5%). When the revised Ankara criteria were tested in the data set, the sensitivity for classifying patients with IgAV was 76.4% (95% CI 69.8% to 82.2%) and the specificity was 94.5% (95% CI 93.4% to 95.1%).

**Conclusion:** The EULAR endorsed Ankara 2008 criteria with added exclusion criteria have good specificity and moderate sensitivity for IgAV. Use of the Ankara criteria in adult patients could help with harmonization of future clinical research projects.

**Date of birth::** mai 29, YY

**Patient Consent**

Yes, I received consent

**Disclosure of Interest**

None Declared

### Vasculitides

#### P496 Comprehensive evaluation of children with behçet's disease

##### Zuhal Karalı^1^, Yasin Karalı^2^, Sukru Cekic^3^, Sara Sebnem Kilic ^2^

###### ^1^Bursa City Hospital, ^2^Pediatric Immunology and Rheumatology, ^3^Bursa Uludag University, Bursa, Türkiye

####### **Correspondence:** Sara Sebnem Kilic

*Pediatric Rheumatology 2024*, **22(2)**: PReS24-ABS-1019

**Introduction:** Behçet disease is a rare vasculitic disorder that is characterized by recurrent oral aphthous ulcers, genital ulcers, and uveitis.

**Objectives:** Our study aims to investigate children with Behcet's disease's clinical manifestations and laboratory characteristics and assess treatment responses.

**Methods:** A retrospective analysis was performed on electronic medical records of 50 children aged between 0 and 18 years who have been followed with Behcet's disease, encompassing demographics, clinical presentations, and laboratory and immunological profiles.

**Results:** Our investigation comprised a cohort of 24 (48%) males and 26 (52%) females. The mean age at the onset of initial symptoms was 9.02±4.71 years, whereas the average age at diagnosis was 11.12±4.24 years (10.75±4.55 for males and 12.35±3.65 for females), with an average diagnostic delay of 2.08 years. Regarding family medical histories, Behcet's disease was documented in the families of 20 patients (40%), with Familial Mediterranean Fever (FMF) emerging as the second most prevalent rheumatological ailment, affecting the families of 11 patients (22%). Oral aphthous ulcers were prevalent in 48 patients (96%), with an average frequency of 7.94±3.8 cases per year. Genital ulcers were present in 17 patients (34%), while signs of uveitis were noted in 9 (18%). Gastrointestinal involvement was identified in 17 patients (34%), while seven patients (18%) exhibited neurological involvement, and five patients (10%) had vascular involvement, which was more prevalent in females. Pathergy positivity was identified in only four patients. Laboratory analyses revealed that 39 patients (78%) were HLA-B51 positive. Mutations in the MEFV gene were found in 5 out of 16 patients (31.2%) undergoing genetic mutation analysis. In immunological assessments, 11 out of 34 patients (32.3%) exhibited a decrease in at least one serum immunoglobulin level relative to their age, yet none necessitated immunoglobulin replacement therapy. Colchicine emerged as the most frequently prescribed medication for treatment (n = 45, 82%), with 4 receiving biological agent therapy. Throughout the follow-up period, clinical improvement was observed in 32 patients (64%). Remarkably, 5 of 8 patients treated for uveitis completely recovered using biological agents.

**Conclusion:** Although Behçet's disease may cause vascular or organ involvement, it is a disease with a good prognosis and low morbidity in children due to high awareness in our country. Throughout the timeline of our study, no patient died, and the morbidity was at an acceptable level.

**Patient Consent**

Not applicable (there are no patient data)

**Disclosure of Interest**

None Declared

**References**

1. Mendes D, Correia M, Barbedo M, Vaio T, Mota M, Gonçalves O, Valente J. Behcet’s disease--a contemporary review. J Autoimmun. 2009 May-Jun;32(3-4):178-88. doi: 10.1016/j.jaut.2009.02.011. Epub 2009 Mar 26. PMID: 19324519.
2. Kone-Paut I. Behcet's disease in children, an overview. Pedia Rheumatol J. (2016) 14:10. doi: 10.1186/s12969-016-0070-z
3. Pain CE. Juvenile-onset Behcet’s syndrome and mimics. Clin Immunol. (2020) 214:108381. doi: 10.1016/j.clim.2020.108381

### Vasculitides

#### P497 Evidence-based personalized angiocorrection of microthromboangiopathy, capillary hematomas, giant capillaries and pathological neoangiogenesis

##### Ulyana Lushchyk ^1^, Viktor V. Novytskyy^2^, Viktor V. Novytskyy^1^, Diaa N. Moamar^3^, Ivanna I. Legka^3^, Serhiy O. Sazchenko^3^

###### ^1^Veritas Research Center, ^2^Institute of Mathematics of NAS of Ukraine, ^3^Clinic of Vascular Innovations, Kyiv, Ukraine

####### **Correspondence:** Ulyana Lushchyk

*Pediatric Rheumatology 2024*, **22(2)**: PReS24-ABS-1107

**Introduction:** Diseases of the rheumatological spectrum remain completely unstudied and require an in-depth study of hemodynamic pathological patterns in the diagnostic protocol of the vascular arteriovenous status of the regional limbic reservoir of the upper and lower extremities in vivo, both by the method of ultrasound at the macroangiological level and by the method of optical capillaroscopy at the microangiological level. It has been revealed that patients of the rheumatological spectrum have specific patterns of changes in the capillary shape in the nail bed, which are highly informative and differentiated in systemic scleroderma and lupus erythematosus and today belong to the nosologically specific patterns of giant capillaries, capillary hematomas, avascular zones and pathological neoangiogenesis in the transitional knee and less studied.

Pathological rearrangements became the object of our research to find hemodynamically based angiocorrective algorithms for restoring an adequate level of blood supply, in particular, to the hands and feet of patients.

**Objectives:** For 4 years, we have carried out combined angioresearch using the methods of ultrasound-angioscanning and dopplerography with the assessment of the vascular status of main and peripheral arteries and veins on an evidence-based basis.

**Methods:** The vascular status of the regional limbic reservoir in the upper and lower extremities has been studied at the macro, peripheral, and micro levels.

The calibre and capillary length, the presence of arteriolo-venular shunts, hemodynamic patterns of arteriolospasm, adequacy of blood filling of all capillary segments and blood flow, pulse wave, sludge phenomenon, microthromboangiopathy, pathological neoangiogenesis, venular stasis and transformation of venular segments, perivascular edema, various patterns of deviation of the shape of capillaries from the ideal shape in the form of a classic hairpin are the main modelling criteria [1, 2].

**Results:** Angiocorrection demonstrates an effective influence on pathologically changed capillaries of vascular preparations both alone and in a combination of personalized therapeutic mixtures, and with individual mathematical modelling of hemodynamic changes, it has been effective for almost all patients.

**Conclusion:** Thus, angiocorrection of hemodynamic patterns in a particular patient is extremely important in the treatment process. Modelling of the identified pathological hemodynamic patterns that are specific for Raynaud's syndrome, systemic scleroderma, systemic lupus erythematosus and oncocapillaries requires further investigation.

**Patient Consent**

Yes, I received consent

**Disclosure of Interest**

None Declared

**References**

1. Lushchyk U: Device for the vascular screening: State Patent of Ukraine. No. 85052. 11.11.2013.
2. Lushchyk UB, Novytskyy VV et al. (2021). Vascular Screening of PathoNeoAngioOncogenesis. (Analytical Approach to an Early Diagnosis of Pathological ArterioVenous Angiotransformations at the Micro- and Macrovascular Levels). Journal of Blood Disorders, Symptoms & Treatments- 2021- Vol. 3.

### Vasculitides

#### P498 Infliximab in the treatment of childhood primary angiitis of the CNS: a case series

##### Sarah Abu Rumeileh ^1^, Ilaria Pagnini^2^, Ilaria Maccora^2, 3^, Edoardo Marrani^2^, Maria Vincenza Mastrolia^2, 3^, Valerio Maniscalco^2^, Gabriele Simonini^2, 3^

###### ^1^Pediatric Department, Rheumatology Unit, AOU Pisana, Santa Chiara Hospital, Pisa; ^2^Rheumatology Unit, Meyer Children Hospital IRCCS, ^3^NEUROFARBA Department, University of Florence, Florence, Italy

####### **Correspondence:** Sarah Abu Rumeileh

*Pediatric Rheumatology 2024*, **22(2)**: PReS24-ABS-1318

**Introduction:** Childhood Primary angiitis of the CNS (cPACNS) is a rare and severe form of vasculitis that can cause pediatric stroke. Currently, there are no treatment guidelines available, and recommendations are based on evidence from limited open-label cohort studies and case series [1].

**Objectives:** To describe the efficacy and safety of infliximab in the treatment of cPACNS.

**Methods:** A single-center observational study was performed in cPACNS receiving infliximab between December 2011 and March 2024.

**Results:** Four patients followed at the Rheumatology Unit of Meyer Children’s Hospital in Florence, were included. Median age at diagnosis of 1.9 years (interquartile range (IQR) 2.7-11.6 years), three males. For all patients, the location and distribution of acute ischemic lesions were identified on brain magnetic resonance imaging (MRI). All patients at diagnosis received glucocorticoids and acetylsalicylic acid. Infliximab (10 mg/kg/4 weeks for patients 1-3; 5 mg/kg/4 weeks for patient 4) was introduced once documented a radiological and clinical progression in three cases; while in one patient, it was started at disease onset due to severe vascular involvement on imaging (median time to introduction: 18.6 months, IQR 0.75-19.3 months). Vascular lesions at the onset and progression of symptoms were detected by magnetic resonance angiography (MRA) and by digital subtraction angiography. The median follow-up was 6.1 years (IQR 1.7-7.8 years). Infliximab infusions are ongoing in two patients, while two patients discontinued therapy after more than 2 years. At the last evaluation, all patients showed stable radiological findings (MRA and magnetic resonance spectroscopy) and normal neurological outcome, and only one patient had residual neurological deficits (patient 4). This patient, a 2-year-old child initially diagnosed with Moya-Moya disease, exhibited a severe disease course; however, after starting infliximab, no clinical and radiological progression has been shown. None of the patients experienced adverse events.

**Conclusion:** Infliximab resulted effective and safe in our case series. Multicenter randomized and controlled studies are required in order to define the optimal treatment approach for cPACNS.

**Patient Consent**

Yes, I received consent

**Disclosure of Interest**

None Declared

**Reference**

1. Beelen J, Benseler SM, Dropol A, et al. Strategies for treatment of childhood primary angiitis of the central nervous system. *Neurol Neuroimmunol Neuroinflamm*. 2019;6(4):e567.

### Vasculitides

#### P499 Diverse management approaches for atypical skin manifestations in igav – preliminary results from the juvenile inflammatory rheumatism (JIR)-clips network

##### Nastasia Kifer^1^, Marija Jelusic^1^, Teresa Giani^2^, Aušra Šnipaitienė^3^, Margarita Ganeva^4^, Francois Hofer^5^, Michael Hofer^6^, Tilmann Kallinich^7, 8^, Daiva Gorczyca^8^ and JIR‐CLiPS network

###### ^1^Department of Pediatrics, University Hospital Centre Zagreb, School of Medicine University of Zagreb, Zagreb, Croatia; ^2^Department of Pediatrics, AOU Meyer IRCCS, Florence, Italy; ^3^Department of Pediatrics, Lithuanian University of Health Sciences, Kaunas, Lithuania; ^4^Department of Pediatric Rheumatology, Medical University Sofia, Sofia, Bulgaria; ^5^Fondation Rhumatismes-Enfants-Suisse, Lausanne; ^6^Department of Pediatrics, Hôpital Riviera-Chablais, Rennaz, Switzerland; ^7^Charité Universitätsmedizin Berlin; ^8^Deutsches Rheuma-Forschungszentrum, an institute of the Leibniz Association, Berlin, Germany

####### **Correspondence:** Nastasia Kifer

*Pediatric Rheumatology 2024*, **22(2)**: PReS24-ABS-1374

**Introduction:** Skin manifestations of IgA vasculitis (IgAV) can range from mild to severe. The severity and duration of cutaneous symptoms have been associated with disease prognosis and different treatment strategies.

**Objectives:** The JIR-CliPS network aims to gather real-life clinical practice strategies (CliPS) used by physicians worldwide to present variations in the diagnosis and management of IgAV, particularly in atypical skin manifestations.

**Methods:** Since September 2022, an online questionnaire has been distributed in English using different channels. Diagnostic and therapy pathways of IgAV with the atypical skin manifestation were analyzed based on different clinical scenarios. The project is funded as a COST (European Cooperation for Science and Technology) action CA21168.

**Results:** Between September 2022 and February 2024, 238 questionnaires were distributed, and 80 responses from physicians from 34 countries were received. Sixty-six percent of respondents had over 10 years of experience with IgAV, predominantly pediatric rheumatologists (88%). Most respondents (79%) often encountered patients with a typical IgAV rash lasting less than four weeks and rarely atypical rashes (45%). Lesions resembling erythema multiforme/target-like lesions (68%), ulcerations (66%), necrotic lesions (61%), and subcutaneous nodules (59%) were identified as atypical for IgAV. Skin biopsies were most frequently considered for recurrent atypical rashes (77%) and atypical rashes lasting over four weeks (65%). Dermatologists performed biopsies in 86% of cases. In scenarios where a typical rash persisted for less than four weeks without other clinical features of IgAV, a majority of respondents (88%) wouldn't initiate treatment. However, oral prednisolone (<2 mg/kg body weight/day) was recomended if the rash was atypical, or typical and persisted for more than four weeks (36% and 58%, respectively). For recurrent rashes, 48% favored low to moderate doses of oral prednisolone. Colchicine emerged as the second choice treatment for recurrent rashes and typical rashes lasting over four weeks (24% and 11%, respectively).

**Conclusion:** Our findings highlight that decisions regarding diagnostic approach and therapy for IgAV vary based on the duration and characteristics of the rash. Low-dose glucocorticoids are frequently chosen for persistent or atypical skin presentations.

**Date of birth::** août 13, Y

**Patient Consent**

Not applicable (there are no patient data)

**Disclosure of Interest**

None Declared

**Reference**

1. Sestan M, Kifer N, Sozeri B, et al. Clinical features, treatment and outcome of pediatric patients with severe cutaneous manifestations in IgA vasculitis: Multicenter international study. Semin Arthritis Rheum. 2023;61:152209.

### Vasculitides

#### P500 Thrombosis in children with behcet disease: a monocentric study

##### Kenza Bouayed^1, 2^, Kawtar El Ouassifi^1^, Ghita Benbrahim Ansari^1, 2^, Asmaa Sakhi^1, 2^, Hanan Aboufaris^1^, Siham Salam^2, 3^

###### ^1^Pediatric Rheumatology, Hôpital Mère-Enfant A. Harouchi, CHU Ibn Rochd; ^2^Faculty of Medicine and Pharmacy, Hassan 2 University; ^3^Pediatric Radiology, Hôpital Mère-Enfant A. Harouchi, CHU Ibn Rochd, Casablanca, Morocco

####### **Correspondence:** Kenza Bouayed

*Pediatric Rheumatology 2024*, **22(2)**: PReS24-ABS-1155

**Introduction:** Behçet's disease “BD” is a systemic vasculitis known for its high risk of thrombosis, which is a serious and potentially fatal complication. In Pediatric BD, thrombosis is very rare.

**Objectives:** The aim of our study is to report cases of children with BD disease complicated by thrombosis and describe their characteristics.

**Methods:** Thirty-six BD patients followed in a Pediatric Rheumatology Department between January 2009 and April 2024 were retrospectively assessed.

**Results:** Thrombosis was found in 6 male patients (16.66 %), mean age was 10, 45 years (5.7-13). It was inaugural in 5 patients. Three patients had cerebral sinus venous thrombosis, 3 patients had deep venous thrombosis, at mesenteric veins (1 case), Budd Chiari syndrome complicated with cardiac thrombosis (1 case) and internal jugular vein associated with lower lobar artery aneurysm (1 case). The mean erythrocyte sedimentation rate was 30mm/1^st^ h, and the mean C-reactive protein was 37.23 mg/L. All patients were treated with low-molecular-weight heparin and anti-vitamin K anticoagulation combined with aggressive immunosuppression using boluses of MP, relayed by oral corticosteroids combined with Azathioprine. In addition, one patient was treated with Cyclophosphamide, 2 received Adalimumab and the third is undergoing pre-biotherapy screening for Adalimumab. Outcome was favorable in 5 cases despite a case of recurrent thrombosis, we deplore one case of death.

**Conclusion:** Thrombosis is a very serious condition that can reveal a vasculitis such as Behçet's disease. Male gender and high inflammatory parameters may increase the risk of thrombosis in BD. All our patients are male and present with an inflammatory syndrome. Etiological diagnosis is essential, because in addition to anticoagulant therapy, etiological treatment is fundamental to stopping the vascular inflammatory process that is the cornestone of thrombosis genesis. The etiological problem arises primarily in inaugural forms, which is why any thrombosis in children must be investigated for evidence of BD, and if the patient is known, the family must be encouraged to consult as soon as any unusual symptoms appear.

**Patient Consent**

Not applicable (there are no patient data)

**Disclosure of Interest**

None Declared

**References**

1. Guzelkucuk Z. Children With Behçet Disease-associated Thrombosis: A Single-Center Experience. J Pediatr Hematol Oncol. 2021 Jan;43(1):e15-e18.
2. Güngörer V. J Clin Factors Associated with the Development of Thrombosis in Pediatric Behçet Disease. Rheumatol. 2023 Jun 1;29(4):e19-e24.

### Vasculitides

#### P501 Childhood takayasu arteritis case series: single center experience

##### Büşra Başer Taşkın, Bengisu Menentoğlu, Ayşenur Doğru, Selen D. Arık, Gülşah Kavrul Kayaalp, Özlem Akgün, Nuray Aktay Ayaz

###### Pediatric Rheumatology, Istanbul University Istanbul Faculty of Medicine, İstanbul, Türkiye

####### **Correspondence:** Büşra Başer Taşkın

*Pediatric Rheumatology 2024*, **22(2)**: PReS24-ABS-1759

**Introduction:** Takayasu arteritis, the third most common systemic vasculitis in children, is a chronic granulomatous vasculitis that affects the aorta and its major branches. It is very rare in the pediatric population, with an estimated incidence of 1-2.6 per 1,000,000.

**Objectives:** The aim of this study is to share our clinical experiences with the demographic, clinical, and angiographic characteristics of patients diagnosed with Takayasu arteritis. Additionally, we aim to detail the diagnosis, treatment, and follow-up processes for this very rare condition.

**Methods:** The single-centered retrospective study included 6 patients diagnosed with Takayasu arteritis according to the 2008 Ankara EULAR/PRINTO/PRES (European League Against Rheumatism/Pediatric Rheumatology International Trials Organization/European Pediatric Rheumatology Association) criteria and followed up at the Pediatric Rheumatology Clinic. The medical files of the patients are retrospectively reviewed.

**Results:** Of the 6 patients with Takayasu arteritis evaluated retrospectively in our clinic, 5 (83%) were female, with a median age at diagnosis of 14.5 years (range: 8-17 years). The most common presenting symptoms were fatigue and weight loss, each reported by 50% of patients. Physical examination revealed heart murmurs in all patients (100%), hypertension in 5 patients (83%), and weakened pulses in 4 patients (67%). Laboratory investigations at the time of diagnosis showed a mean CRP of 101 mg/L (range: 11-268) and an ESR of 72 mm/hour (range: 8-120). All patients were classified as type 5 based on the angiographic classification of Takayasu arteritis. Renal arteries were involved in all cases, followed by the celiac truncus branches in five patients, carotis communis artery and abdominal aorta in four patients each.

All patients received corticosteroid therapy. Methotrexate (66.7%) and cyclophosphamide (33.3%) were the most commonly preferred non-biologic anti-rheumatic agents. Tocilizumab (66.7%) and etanercept (33.3%) were the most frequently used biologic agents. During follow-up, one patient underwent percutaneous transluminal angioplasty (PTA) due to total occlusion of abdominal aorta.

**Conclusion:** Takayasu arteritis is a rare and challenging condition that often requires a high level of suspicion due to its subtle onset and nonspecific symptoms, which can mimic other inflammatory conditions. The clinical presentation can vary widely depending on the location and size of the affected vessels. In children with unexplained hypertension, weight loss, fatigue, and elevated acute phase reactants, Takayasu arteritis should be strongly considered. Our case series highlights the importance of comprehensive physical examinations and advanced imaging techniques for early and accurate diagnosis. Effective management of active disease involves the use of corticosteroids and immunosuppressive agents. There is a significant need for international studies to identify reliable biomarkers for assessing disease activity and treatment efficacy, which will help in defining long-term outcomes for pediatric Takayasu arteritis more clearly.

**Date of birth::** juillet 12

**Patient Consent**

Yes, I received consent

**Disclosure of Interest**

None Declared

### Vasculitides

#### 502 Analysis of worldwide data of diagnostic approaches for kawasaki disease through the Juvenile Inflammatory Rheumatism (JIR)-clips network

##### Jurgita Marčiulynaitė^1^, Aušra Šnipaitienė^1^, Teresa Giani^2^, Margarita Ganeva^3^, Zeynep Balik^4^, Francois Hofer^5^, Michael Hofer^6^, Tilmann Kallinich^7, 8^, Daiva Gorczyca^7^ and JIR‐CliPS network

###### ^1^Department of Pediatrics, Lithuanian University of Health Sciences, Kaunas, Lithuania; ^2^Department of Pediatrics, AOU Meyer IRCCS, Florence, Italy; ^3^Department of Pediatric Rheumatology, Medical University Sofia, Sofia, Bulgaria; ^4^Division of Pediatric Rheumatology, Department of Pediatrics, Hacettepe University, Ankara, Türkiye; ^5^Fondation Rhumatismes-Enfants-Suisse, Lausanne; ^6^Department of Pediatrics, Hôpital Riviera-Chablais, Rennaz, Switzerland; ^7^Charité Universitätsmedizin Berlin; ^8^Deutsches Rheuma-Forschungszentrum, An Institute of the Leibniz Association, Berlin, Germany

####### **Correspondence:** Jurgita Marčiulynaitė

*Pediatric Rheumatology 2024*, **22(2)**: PReS24-ABS-1137

**Introduction:** Kawasaki disease (KD) is currently considered as the leading cause of acquired heart disease in childhood, and the KD diagnostic approach is constantly evolving.

**Objectives:** The JIR-CliPS network aims to provide a library of real-life clinical practice strategies (CliPS) used by physicians worldwide for different juvenile inflammatory diseases. The study aims to investigate the worldwide variations in the characteristics of complete and incomplete KD.

**Methods:** From September 2022, an English-language survey with 35 specific questions on KD was distributed worldwide. The questions included demographic data, diagnostics, and treatment approaches. Diagnostic pathways for complete and incomplete KD were analyzed based on different clinical scenarios. The chi-square test was applied to assess differences among the most representative countries. The project is funded as a COST (European Cooperation for Science and Technology) action (CA21168).

**Results:** A total of 144 questionnaires, received between September 2022 and February 2024, were analyzed. Physicians from 47 countries participated. The best-represented countries (answered more than ten questionnaires) were Brazil, France, Spain, and Turkey. 85% of respondents were pediatric subspecialists, with a predominance of pediatric rheumatologists (67.5%). Half of the participants had over 10 years of experience in the field. The majority of respondents (67.4%) consider complete KD in patients presenting with at least 4 clinical criteria for KD along with a fever for at least 5 days, while approximately 30% diagnose complete KD also in patients with a fever lasting 3-4 days. Among participants from the most represented countries, respondents from France most likely diagnose a complete KD before day 5 of fever (p = 0.0056). Eighty-seven percent of participants agreed that fever is mandatory for diagnosing incomplete KD. In a febrile child with coronary artery lesions (CALs), more than half of respondents (58%) diagnose incomplete KD in the presence of at least one additional clinical criterion. When CALs are present without other clinical criteria, respondents from Europe are more likely to diagnose KD (p = 0.045). The most important factors suggesting a KD diagnosis in ambiguous cases include C reactive protein greater than 30 mg/L (70.1%), thrombocytosis after the seventh day of fever (64.5%), infant age (62.3%), and erythrocyte sedimentation rate greater than 40 mm/h (61.3%).

**Conclusion:** Differences defining complete and incomplete KD were observed within the JIR-CliPS network. Interim analysis suggests that in Europe physicians consider complete KD also in children before the fifth day of fever. CALs are the key feature confirming KD diagnosis by incomplete presentation, particularly in Europe. Further distribution of questionnaires is warranted to better understand the reasons behind varying diagnostic approaches.

**Patient Consent**

Not applicable (there are no patient data)

**Disclosure of Interest**

None Declared

### Vasculitides

#### 503 Hemodynamic patterns of microcirculation VST-disorders (vascular screening technology) in the superficial and deep layers of the microcirculatory bed in patients with rheumatological spectrum

##### Ulyana Lushchyk ^1^, Viktor V. Novytskyy^2^, Viktor V. Novytskyy^1^, Diaa N. Moamar^3^, Serhiy O. Sazchenko^3^, Ivanna I. Legka^1^

###### ^1^Veritas Research Center; ^2^Instutute of Mathmatics of NAS of Ukraine; ^3^Clinic of Vascular Innovations, Kyiv, Ukraine

####### **Correspondence:** Ulyana Lushchyk

*Pediatric Rheumatology 2024*, **22(2)**: PReS24-ABS-1201

**Introduction:** During the last decade, specific patterns of changes in the capillary shape in the nail bed in patients with a rheumatological spectrum have been recorded, such as a violation of the arteriolo-venular balance in Raynaud's syndrome, systemic scleroderma, lupus erythematosus, etc.

**Objectives:** During 2004-2024, we have conducted microcirculation study in 1,846 patients with a clinical picture of Raynaud's syndrome, 127 patients with confirmed diagnoses of systemic scleroderma, and 94 systemic lupus erythematosus.

**Methods:** Microvascular study and assessment of specific pathological patterns in patients are based on the method of large-format smart optical capillaroscopy with visualization of the capillaries of the superficial and deep layers of the microcirculation in the nail bed of fingers and toes [1,2].

**Results:** It has been established that the clinical picture of Raynaud's syndrome is manifested by hemodynamic patterns at the level of microcirculatory changes. 68% of patients with a clinical diagnosis of Raynaud's syndrome has dominant venular stasis with various hemodynamic patterns of venulectasia, pathological venular network, microthromboangiopathy.

All patients with a confirmed diagnosis of systemic scleroderma and systemic lupus erythematosus have specific patterns that clearly identified stasis in the transitional knee, pathological neoangiogenesis in the projection of the transitional knee, capillary hematomas, giant capillaries, subdecompensated perivascular edema, post-Covid occlusive microthromboangiopathy [1, 2].

Hypothermia in the anamnesis and post-Covid vascular syndrome significantly worsens the picture of microcirculation and deepens the clinical picture of the course of rheumatological pathology with increasing complaints not only of skin color change, but also of numbness in the limbs, pain in the small joints of the hands, and cold "allergy". Patterns of pathological neoangio-oncogenesis with a high risk of developing oncopathology have been found in 17% of all examined patients.

An ultrasound examination of the pathology in the main arteries in limbs in systemic lupus erythematosus has revealed pathological "boot" patterns with a violation of the elastic-tonic properties of the arterial wall during hemodynamic overload with systolic pulse pressure.

**Conclusion:** The rheumatological spectrum of diseases is characterized by specific hemodynamic patterns of structural pathological transformations in the microcirculatory channel.

Mathematical modeling has revealed the violation of blood supply to the skin and small joints as the most dependent on the state of blood flow and the most sensitive to the deficiency of blood supply according to the residual principle.

**Patient Consent**

Yes, I received consent

**Disclosure of Interest**

None Declared

**References**

2. Lushchyk U: Device for the vascular screening: State Patent of Ukraine. No. 85052. 11.11.2013.
3. Lushchyk UB, Novytskyy VV, LushchykNG, BabiyIP, Alexeyeva TS: The Up-to-date Potential of an Integrated Functional Estimation of the Arteriovenous Balance in the Closed Vascular System on the Macro- and Microlevel (popular functional angiology: from main arteries through capillaries to main veins). Kyiv: Istyna; 2006:120.

### Vasculitides

#### 504 Analysis of worldwide data of initial therapy for kawasaki disease through the juvenile inflammatory rheumatism (JIR)-clips network

##### Margarita Ganeva^1^, Zeynep Balik ^2^, Jurgita Marčiulynaitė^3^, Francois Hofer^4^, Michael Hofer^5, 6^, Konstantinos Pateras^7, 8^, Tilmann Kallinich^9, 10^, Daiva Gorczyca^9^ and JIR‐CLiPS network

###### ^1^Department of Pediatric Rheumatology, Medical University Sofia, Sofia, Bulgaria; ^2^Department of Pediatric Rheumatology, Hacettepe University, Ankara, Türkiye; ^3^Department of Pediatrics, Lithuanian University of Health Sciences, Kaunas, Lithuania; ^4^Fondation Rhumatismes-Enfants-Suisse, Lausanne; ^5^Department of Pediatrics, Centre Hospitalier Universitaire Vaudois (CHUV), Geneva; ^6^Pediatric Immuno-Rheumatology Unit of Western Switzerland, Lausanne University Hospital, Lausanne, Switzerland; ^7^Department of Biostatistics, University Medical Center Utrecht (UMCU), Utrecht, Netherlands; ^8^Department of Public Health, University of Thessaly (UTH), Thessaly, Greece; ^9^Charité Universitätsmedizin Berlin, ^10^Deutsches Rheuma-Forschungszentrum, an institute of the Leibniz Association, Berlin, Germany

####### **Correspondence:** Zeynep Balik

*Pediatric Rheumatology 2024*, **22(2)**: PReS24-ABS-1216

**Introduction:** Kawasaki disease (KD) is the leading cause of childhood-acquired heart disease. Despite great improvement in the management of KD, unmet needs remain.

**Objectives:** Our network aims to gather real-life clinical practice strategies (CliPS) used by physicians worldwide for different KD disease scenarios. Here we will focus on characterization of high-risk patients for coronary artery lesions (CAL) and evaluation of the initial and intensified treatment of KD, especially in cases with complications such as coronary artery aneurysms (CAA), cardiac shock or macrophage activation syndrome (MAS).

**Methods:** An online questionnaire has been distributed in English using different channels since September 2022. The project is funded as a COST (European Cooperation for Science and Technology) action CA21168.

**Results:** Between September 2022 and February 2024, we sent out 238 questionnaires and received responses from physicians from 47 countries. The majority of physicians were from Turkey (14%), Brazil (12%), New Zealand (7.5%), and Spain (7.5%). Pediatric rheumatology (67.5%) was the most common subspecialty. Almost all respondents (96%) are involved in both inpatient and outpatient care. Half of the respondents had over 10 years of experience with KD. Most respondents (78%) treatedcomplete and incomplete KD similarly. Of those, 98% used intravenous immunoglobulin (IVIG) of dose 2g/kg as initial therapy, often combined with acetylsalicylic acid (ASA) (87%). The preferred ASA dosage ranged between 30-50 mg/kg/day (85%). Respondents consider age under one year, initial Z score ³2, disease duration over 14 days, and C-reactive protein greater than 100 mg/L as the most important risk factors for CAL. In high-risk patients, treatment was intensified with various steroid regimens, with 45% preferring intravenous steroids, 34% oral steroids. The most common intravenous steroid regimen was 10-30 mg/kg/day of methylprednisolone for three days (50%), followed by a one-day course (29%). In cases of KD complications such as early onset of CAA, cardiac shock, or MAS, 83% of respondents augment initial IVIG and ASA therapy with a pulse steroid therapy. Among these, 57% preferred three-days course of methylprednisolone 10-30 mg/kg/day intravenously, followed by tapering over 2-3 weeks. For preexisting MAS, 30% of respondents used anakinra, 12.5% infliximab, and 12% cyclosporine.

**Conclusion:** There are differences in KD treatment strategies in real-life clinical practices. The majority of respondents used steroids for intensification of treatment in high-risk patients with an intravenous regimen being the most preferred. Future studies are necessary to identify the most effective strategies applicable across different healthcare systems.

**Date of birth::** septembre

**Patient Consent**

Not applicable (there are no patient data)

**Disclosure of Interest**

None Declared

### Vasculitides

#### 505 Clinical case: facial nerve neuritis as a rare manifestation of kawasaki disease

##### Seda Kurbanova, Angelina Bryk, Sania Valieva, Anna Sologub

###### Rheumatology, Morozov Children’s City Clinical Hospital, Moscow, Russian Federation

####### **Correspondence:** Seda Kurbanova

*Pediatric Rheumatology 2024*, **22(2)**: PReS24-ABS-1300

**Introduction:** Kawasaki disease (KD) is an acute systemic condition characterized by predominant involvement of small and medium-sized arteries. Patients may exhibit clinical manifestations not included in the diagnostic criteria. Facial nerve involvement is uncommon, with the majority of the existing literature focusing on individual case reports.

**Objectives:** In this report, we outline a clinical case involving a patient diagnosed with KD and facial nerve palsy (FNP).

**Results:** A boy, 5-month-old, was brought to the local pediatrician with complaints of fever up to 38.3В°C, conjunctivitis, fine-spotted rash on the trunk and limbs, and skin peeling of the fingers and toes. The presumptive diagnosis was enteroviral infection, thus antibiotic therapy (ABT) with amoxicillin was initiated. An allergic reaction was noted, which was managed with intramuscular administration of dexamethasone and taking chloropyramine. ABT was switched to cefixime, with a temporary improvement in the patient's condition followed by a subsequent rise in temperature. On the 16th day, complaints of facial asymmetry appeared. The child was referred to the pediatric neurology department of the regional children's hospital with ineffective therapy. On the 24th day, they independently sought care at the State Budgetary Healthcare Institution "Morozov Children's City Clinical Hospital" of the Moscow City Health Department. A comprehensive examination was conducted. Echocardiography revealed a giant aneurysm in the proximal and middle segments of the right coronary artery (CA) and a large one in the left CA extending to the left main CA. The diagnosis was confirmed on the 30th day: Incomplete KD with CA involvement. The patient was transferred to the rheumatology department, and pathogenetic therapy was started: intravenous immunoglobulin (IVIG) at a dose of 2 g/kg, acetylsalicylic acid at 2.5 mg/kg/day, and subcutaneous dalteparin sodium at 125 IU/kg/day. By the 34th day, there was a persistently high level of inflammatory biomarkers with slight echocardiographic dynamics. A repeat dose of IVIG at 2 g/kg was administered. By the 37th day, after two courses of IVIG the child's condition was considered to be immunoresistant. Infliximab (anti-TNF-a) was prescribed off-label at a dose of 5 mg/kg. By the 41st day, there was positive clinical and laboratory progress, with a reduction in perivascular adhesions in the right CA observed instrumentally. The signs of FNP in the patient were resolved.

**Conclusion:** FNP in KD is a rare but serious complication. Early detection and appropriate treatment of CA aneurysms in KD can significantly improve the prognosis. Considering this, it is necessary to include KD in the list of conditions for differential diagnosis that may cause FNP and to perform an echocardiogram when a patient has a history of fever for more than 5 days.

**Patient Consent**

Yes, I received consent

**Disclosure of Interest**

None Declared

### Vasculitides

#### 506 Clinical case: administration of infliximab in a patient with refractory kawasaki disease

##### Seda Kurbanova, Daria Pykhtina, Sania Valieva, Angelica Nargizyan

###### Department of Rheumatology, Morozov Children’s City Clinical Hospital, Moscow, Russian Federation

####### **Correspondence:** Seda Kurbanova

*Pediatric Rheumatology 2024*, **22(2)**: PReS24-ABS-1315

**Introduction:** The treatment of Kawasaki disease involves the administration of intravenous immunoglobulin (IVIG) at a dose of 2 g/kg as a single continuous infusion. Most patients respond quickly to IVIG administration, however, 10-20% do not respond to this therapy and experience recurrent fever within 36-48 hours after treatment.

**Objectives:** In this report we demonstrated clinical observation of a complete form of Kawasaki disease with resistance to IVIG therapy and liver involvement.

**Methods:** Case report

**Results:** A 9-year-old boy was transferred from the infectious clinical hospital to the rheumatology department with a tentative diagnosis of "Cryptogenic Hepatitis. Kawasaki Disease?". A thorough assessment in the infectious disease department was confirmed the absence of infectious diseases. On the 7th day of the illness, IVIG therapy was administered at a dose of 2 g/kg; however, the febrile fever persisted. Laboratory tests showed ALT–160 IU/L, AST-87 IU/L, bilirubin-138 μmol/L, CRP-77 mg/mL. Upon arrival at the rheumatology ward: prolonged fever exceeding 12 days, scleritis, cheilitis, a rash on the torso, swelling in the hands, feet, lymph node enlargement on the left side of the neck, skin jaundice. The blood count results showed LEU 12.77x10^9/L, PLT 632x10^9/L, ESR 97 mm/h (by Westergren method). Biochemical blood analysis revealed albumin 26 g/L (normal range up to 35-52 g/L), ALT-78 IU/L, AST-95 IU/L, bilirubin-124 μmol/L, CRP-50 mg/L (normal range up to 5 mg/L). According to the echocardiogram data (12th day of illness), there were signs of coronary arteritis, dilation of the coronary arteries. Liver elastometry showed the absence of fibrosis, elastosis. Magnetic resonance cholangiopancreatography revealed liver enlargement without structural, ductal abnormalities. The diagnosis of "Complete Kawasaki disease with coronary artery involvement" was confirmed. On the 12th day of the illness, a second round of IVIG therapy was administered at a dosage of 2 g/kg. Nevertheless, the fever persisted, inflammatory biomarkers continued to show elevated levels, and there was a negative trend observed in the echocardiogram findings. The lack of response to two rounds of IVIG therapy led to the identification of a refractory form of Kawasaki disease. A decision was made to initiate pulse therapy with glucocorticoids (10-15 mg/kg No.5). After therapy, the boy experienced a recurrence of fever. On the 21st day of the illness, Infliximab was initiated at a dose of 5 mg/kg off-label. By the second day following the infusion, a favorable progression was noted, characterized by enhanced general health, decreased fever, restoration of normal values in laboratory biomarkers.

**Conclusion:** This clinical observation demonstrates a positive effect of Infliximab treatment in a patient with Kawasaki disease resistant to the previous three courses of immunomodulatory therapy and rare manifestation of this disease as a liver involvement.

**Patient Consent**

Yes, I received consent

**Disclosure of Interest**

None Declared

### Vasculitides

#### 507 Assesment of thiols as markers of oxidative stress in patients with IGA vasculitis

##### Mario Sestan^1^, Martina Held^1^, Daniel Turudic^1^, Ana Kozmar^2^, Nastasia Kifer^1^, Sasa Srsen^3^, Alenka Gagro^4^, Marijan Frkovic^1^, Marija Jelusic^1^

###### ^1^University Hospital Centre Zagreb, Department of Paediatrics, University of Zagreb School of Medicine; ^2^Department of Laboratory Diagnostics, University of Zagreb School of Medicine, University Hospital Centre Zagreb, Zagreb; ^3^Department of Paediatrics, University of Split School of Medicine, University Hospital Centre Split, Split; ^4^Children’s Hospital Zagreb, Josip Juraj Strossmayer University of Osijek, Medical Faculty Osijek, Zagreb, Croatia

####### **Correspondence:** Mario Sestan

*Pediatric Rheumatology 2024*, **22(2)**: PReS24-ABS-1419

**Introduction:** IgA vasculitis (IgAV) is the most common systemic vasculitis in childhood, with complex and still not fully known pathogenesis. There is growing interest in studying biomarkers of oxidative stress. Thiols are a group of molecules found in all cells and are important antioxidants protecting the cell from the action of free radicals. The role of thiols is not known in IgAV.

**Objectives:** We investigated whether thiols can serve as biomarkers in IgAV: do they indicate active disease and whether there is association between different phenotypes of the disease and dynamic thiol homeostatic status.

**Methods:** We have measured thiol levels in serum and urine samples using a colorimetric method for the detection of free sulfhydryl groups. Measurement was performed at the onset of IgAV and after a six-month interval and once in the control group.

**Results:** Our cohort consisted of 84 patients with IgAV, of whom there were 45 girls, with a median (IQR) age of 6.38 (4.38 - 7.92) years and 49 controls. Musculoskeletal manifestations were present in 76 patients (90.5%), gastrointestinal manifestations in 34 patients (40.5%), and nephritis developed in 26 patients (30.9%). In 32 patients (38.1%), the rash occurred diffusely throughout the body. Median (IQR) serum thiol levels were not statistically significantly different between patients with IgAV and control group at the disease onset (65.50 (31.63 − 94.31) μM vs. 62.77 (28.46 − 86.06) μM, p = 0.75) nor after 6 months of follow up (65.50 (33.81 − 97.20) μM vs. 62.77 (28.46 − 86.06) μM, p = 0.75). However, there was a statistically significant difference in urinary thiol levels between patients with IgAV and the control group at dIsease onset (15.30 (9.98 − 37.23) μM vs. 27.36 (14.93 − 43.1) μM, Welch t-test on log-transformed data p = 0.048). After 6 months of follow-up, this difference was not significant. No statistically significant difference was found in serum or urinary thiol levels between patients with IgAV who developed nephritis, gastrointestinal manifestations, or generalized rash. Upon subsequent investigation, the values outside of 2+/-SD (outliers and extremes) proved unreliable and were removed.

**Conclusion:** Assessing thiols in plasma and urine offers an indirect measure of antioxidant defense. In our cohort of patients with IgAV, the determination of thiol levels showed limited value in determining disease activity and differentiating patients with different disease phenotypes. SUPPORT: Croatian Science Foundation project IP-2019-04-8822

**Patient Consent**

Yes, I received consent

**Disclosure of Interest**

None Declared

### Vasculitides

#### 508 Unravelling the enigma: anca-negative eosinophilic Granulomatosis with Polyangiitis (EGPA)

##### Hafsa Tufail, Emily Tan, Orla Killeen, Emma MacDermott, Kathy Gallagher

###### Rheumatology, CHI at Crumlin, Dublin, Ireland

####### **Correspondence:** Hafsa Tufail

*Pediatric Rheumatology 2024*, **22(2)**: PReS24-ABS-1475

**Introduction:** Eosinophilic granulomatosis with polyangiitis (EGPA), previously known as Churg-Strauss Syndrome, is a small to medium vessel vasculitis, recognized as one of the anti-neutrophil cytoplasmic antibody (ANCA) – associated vasculitis. It is extremely rare in children and associated with significant morbidity and mortality.

**Objectives:** To report the pulmonary and extrapulmonary clinical manifestations of EGPA in a 13-year-old boy.

**Methods:** This 13-year-old boy presented to the emergency department with a 2-week history of intermittent vomiting. His past medical history included allergic rhinitis and absence seizures for which he was taking ethosuximide. On further questioning, he reported a dry irritating cough for 1 month and weight loss of 2 kg over 2 months. On examination, he was haemodynamically stable. He was pale with a dry cough and rhinitis. Cardiovascular, respiratory, and abdominal examinations were otherwise unremarkable.

Initial bloods showed significantly elevated peripheral eosinophil counts of 16.94×/L (0.1-0.8) with an ESR of 19 mm/hr (1-13) along with normal haemoglobin and platelets. ANCA was negative. His chest x-ray showed prominent hilar markings which looked vascular in nature. Echocardiogram was normal. He was extensively screened for infections including parasites. Bone marrow showed a degree of eosinophilia. In view of the vomiting, a CT head was performed which showed no intracranial pathology however, mucosal thickening was noted in the maxillary antra bilaterally. Pulmonary function tests showed a markedly raised FeNo indicative of significant airway inflammation. Subsequently, a high-resolution CT showed predominantly peripheral patchy airspace/ground glass changes throughout the periphery of both lungs with small additional nodules and prominent thick-walled bronchi.

The patient proceeded to a lung biopsy which showed striking eosinophilia with infiltration and destruction of airways, venulitis and possible capillaritis with patchy eosinophilic consolidation, abundant fresh haemorrhage and no granulomas. A bronchoalveolar lavage contained a striking number of apoptotic cells in addition to viable eosinophils. Oesophageal biopsy confirmed eosinophilic oesophagitis. A nasal biopsy showed mild eosinophilic infiltrate without any granulomas. Based on the clinical, radiological and histopathological features, a diagnosis of ANCA-negative EGPA was confirmed.

**Results:** He received a pulse of intravenous methylprednisolone followed by tapering oral prednisolone. In view of the multi-organ involvement, he was commenced on Rituximab and cyclophosphamide.

**Conclusion:** This case highlights the pulmonary and extrapulmonary manifestations of EGPA. Unexplained peripheral eosinophilia with even mild symptoms of asthma or rhinitis should prompt clinicians to investigate for EGPA. The diagnosis is often challenging and requires a multidisciplinary team approach. Early aggressive immunosuppression is recommended to prevent life-threatening complications.

**Patient Consent**

Yes, I received consent

**Disclosure of Interest**

None Declared

**References**

1. Emmi, G., Bettiol, A., Gelain, E. *et al.* Evidence-Based Guideline for the diagnosis and management of eosinophilic granulomatosis with polyangiitis. *Nat Rev Rheumatol* **19**, 378–393 (2023). 10.1038/s41584-023-00958-w
2. Groh M, Pagnoux C, Baldini C, *et al.* Eosinophilic granulomatosis with polyangiitis (Churg-Strauss) (EGPA) Consensus Task Force recommendations for evaluation and management. *Eur J Intern Med*. 2015;26(7):545-553. doi:10.1016/j.ejim.2015.04.022

### Vasculitides

#### 509 Renal artery involvement in a patient with takayasu arteritis: case report

##### Nathalie Yepes^1^, on behalf of GRINPED, María D. P. Gómez^2^, José F. Gomez^1^, Jorge A. Endo^3^

###### ^1^Pediatrics; ^2^Pediatric Rheumathology, ^3^Pediatric Neprhology, Universidad Libre, Seccional Cali, Cali, Colombia

####### **Correspondence:** Nathalie Yepes

*Pediatric Rheumatology 2024*, **22(2)**: PReS24-ABS-1020

**Introduction:** Takayasu arteritis (TAK) is an idiopathic granulomatous disease of large vessels that predominantly affects the aorta, main arterial branches and the lungs. The prevalence is higher in adults than in children, with an incidence of 1.1 per million people. Its etiology remains poorly understood.

**Objectives:** We present the case of a pediatric patient with loss of left renal mass due to a decrease in the caliber of the ipsilateral renal artery months after the debut of Takayasu Arteritis as an abdominal aortic aneurysm is presented. The patient meets TAK criteria according to EULAR/PRINTO/PReS classification based on angiographic abnormality, arterial hypertension and elevated acute phase reactants.

**Results:** TAK presents systemic involvement and cardiovascular alterations (75-85%), hypertension in more than 80% of cases, with renal artery stenosis in almost 50% of patients. The general treatment of TAK is aimed at controlling vascular inflammation and preventing irreversible organ damage

**Conclusion:** Despite an early diagnosis with timely and aggressive treatment, follow-up in Takayasu disease is essential given that its progression can continue to occur. Strategies must be established to ensure adherence to follow-up.

**Date of birth::** novembre 0

**Patient Consent**

Yes, I received consent

**Disclosure of Interest**

None Declared

**References**

1. Aeschlimann FA, Yeung RSM, Laxer RM. An Update on Childhood-Onset Takayasu Arteritis. Vol. 10, Frontiers in Pediatrics. Frontiers Media S.A.; 2022.
2. Aeschlimann FA, Twilt M, Yeung RSM. Childhood-onset Takayasu Arteritis. Eur J Rheumatol [Internet]. 2020 Feb 3;7(1):58–66. Available from: https://eurjrheumatol.org//en/childhood-onset-takayasu-arteritis-133296
3. Gamboa P. Takayasu’s arteritis. Vol. 27, Revista Colombiana de Cardiologia. Elsevier B.V.; 2020. p. 428–33

### Vasculitides

#### 510 ANCA-associated vasculitis with central nervous system involvement: two case studies

##### Najat Hijazi^1^, Belen Legal^1^, Ana Recalde^2^, Horacio Lezcano^3, 4^, Zunilda Gonzalez^3^, Luis Celias^3, 4^, Analy Barreto^5^, Sara Pozzi^5^, Marlene Martinez-Pico^1^, Lujan Estigarribia^5^, Avelina Troche^1^, Cynthia Florentin^1^, Natalia Cabrera ^1, 6^ on behalf of Paediatric RheumatologY Study Group (PY Study Group)

###### ^1^Paediatric Department, Instituto de Previsión Social; ^2^Department of Physiology, Universidad Catolica "Nuestra Señora de la Asunción"; ^3^Department of Pathology, Instituto de Previsión Social; ^4^Sociedad Paraguaya de Anatomia Patológica y Citología; ^5^Department of Ophthalmology, Instituto de Previsión Social; ^6^Sociedad Paraguaya de Reumatología, Asunción, Paraguay

####### **Correspondence:** Judith Mura

*Pediatric Rheumatology 2024*, **22(2)**: PReS24-ABS-1439

**Introduction:** Paediatric anti-neutrophil cytoplasmic antibody (ANCA)-associated vasculitides (AAV) are a rare group of diseases in children, posing diagnostic challenges. (1) There are no previous studies in Paraguay on paediatric AAV.

**Objectives:** To describe 2 cases of AAV with signs of ocular disease at onset, as a manifestation of central nervous system (SNC) involvement.

**Methods:** Case report

**Results:** Two 11-year-old previously healthy female pediatric patients presented with ocular symptoms. Patient A had persistent bilateral conjunctival injection, acute visual acuity decline, and purpuric lesions on the lower limbs. Patient B had left ocular proptosis progressing over a year.

Patient A was diagnosed with central retinal artery occlusion (CRAO) and total retinal detachment in the right eye, along with multi-organ involvement indicating microscopic polyangiitis. Laboratory tests revealed multi-organ involvement with elevated inflammatory markers (CRP: >118.6 mg/dL, fibrinogen= 634 mg/dL), normal C3 and C4 levels, negative ANA and anti-DNA antibodies, microhematuria (10-12/field), proteinuria (30 mg/dL), and positive ANCA myeloperoxidase (MPO) (>300). Skin biopsy showed residual leukocytoclastic vasculitis with thrombosis. Renal biopsy revealed focal segmental necrotizing glomerulonephritis consistent with microscopic polyangiitis. Despite corticosteroid and cyclophosphamide therapy, her visual acuity did not improve (in right eye).

Patient B initially showed improvement with oral corticosteroid treatment for orbital inflammation but relapsed later. Positive ANCA MCO and ANA (1/320) were obtained. she was admitted in the ER due to vomiting, altered consciousness, and right-sided hemiparesis. Cranial CT revealed a neoproliferative lesion in the left parietal region, leading to biopsy showing an intraorbital hypervascularized, solid elastic mass with bleeding. During surgery, left reactive unresponsive mydriasis and convulsive event were noted. Brain MRI revealed an acute ischemic vascular event with hemorrhagic transformation in the territory of the left middle cerebral artery, causing mass effect and midline shift, necessitating decompressive craniotomy and intensive care for 9 days. Biopsy of the orbital lesion showed chronic nonspecific lymphocytic infiltrate.

Both patients responded well to treatment including methylprednisolone, cyclophosphamide (6 doses) as induction treatment, and rituximab as maintenance.

**Conclusion:** The rarity of paediatrics AAV needs a high index of suspicion based on clinical, physical examination, and laboratory analysis. Effective intervention by the medical team may be crucial, particularly in severe complications such as CRAO, for mmediate corticosteroid administration in or life-threatening events, such as haemorrhagic stroke necessitating decompressive craniotomy.

**Date of birth::** janvier 16

Patient Consent

Yes, I received consent

**Disclosure of Interest**

None Declared

**Reference**

1. Mahi, Sl et al. Pediatr Nephrol, 2023

### Vasculitides

#### 511 Severe abdominal complication in a child with hypocomplementemic atypical IGA vasculitis successfully treated with sodium mycophenolate

##### Andrea Uva ^1^, Andrea Catzola^1^, Francesca Orlando^1^, Roberta Gigante^2^, Chiara Tornincasa^1^, Simona Errichiello^1^, Pasquale D'Avino^1^, Luigi Martemucci^1^, Maria Tardi^1^

###### ^1^Pediatric Immuno-rheumatology Unit, Santobono-Pausilipon Children's Hospital; ^2^Department of Translational Medical Sciences, Section of Pediatrics, Federico II University, Naples, Italy

####### **Correspondence:** Andrea Uva

*Pediatric Rheumatology 2024*, **22(2)**: PReS24-ABS-1763

**Introduction:** IgA vasculitis (IgAV) is the most common childhood primary systemic vasculitis affecting skin, joints, kidneys and intestine. The pathogenesis remains unclear, although serum IgA seems to play a pivotal role as well as complement activation at the tissue level. Indeed, serum complement levels tend to be normal and its reduction in patient with IgAV is not clearly associated with a more severe disease course

**Objectives:** We describe the case of a child with IgAV and C4 reduction serum levels complicated by severe abdominal course.

**Methods:** Serum immunoglobulins and the complement system were measured with nephelometry; anti-C1q antibodies were detected using the CE-IVD (buhlmann lab) kit.

**Results:** A 7 y.o.girl was admitted to our hospital following a week of palpable purpuric non-itching rash, cutaneous swelling in different body areas and arthralgia; her family medical record was negative. Exams showed increased IgA values with reduction of C4 (0.08 g/L), normal hemoglobin and coagulation parameters; no increase of CRP (3 mg/L) or ESR. ANA, ENA, antidsDNA and ANCA were negative. A presumptive diagnosis of IgAV was made and she underwent an ibuprofen course. During the hospitalization her clinical condition worsened. She started complaining abdominal pain, so an US abdomen was performed showing no signs of intussusception but a huge presence of fluid and a severe thickening and swelling of large and small intestinal loops. She was treated with glucocorticoids (2 mg/kg) but she developed severe rectal bleeding needing a blood transfusion. She started IV immunoglobulin (2 g/kg) on top of corticosteroids with poor response. Her exams showed a marked reduction of C4 fraction (0.04 g/L) so a skin biopsy was performed resulting in leukocytoclastic vasculitis. Moreover, anti-C1q antibodies were detected: 21.5 U/ml (positive values>15). As her condition worsened, three IV methylprednisolone pulses were done with mild response. Due to the persistent rectal bleeding with no signs of US enhancement at several abdomen evaluations and progressive increase of urine proteinuria (700 mg/24 h), she was started with sodium mycophenolate (30 mg/kg) with complete response. A renal biopsy has been scheduled but not already done according to nephrologists.

**Conclusion:** Our case report suggests that patients with severe IgAV could have an impairment of the complement pathway. AntiC1q-antibodies in IgAV could have a role in the pathogenesis of complications and they should be performed when hypocomplementemia has been detected. As for the treatment, sodium mycophenolate over mofetil mycophenolate could be a promising option for gastrointestinal complication in these patients considering its higher tolerability intestinal profile.

**Patient Consent**

Yes, I received consent

**Disclosure of Interest**

None Declared

### Vasculitides

#### 512 Case report: takayasu arteritis in a tertiary hospital in Northeast Mexico

##### Sol Jiménez^1^, Luis C. Jimenez^2^, Rosa E. Vela^1^, Felix J. Dragustinovis^2^

###### ^1^Pediatric Rheumatology, ^2^Pediatrics, Instituto Mexicano del Seguro Social , Monterrey, Mexico

####### **Correspondence:** Sol Jiménez

*Pediatric Rheumatology 2024*, **22(2)**: PReS24-ABS-1431

**Introduction:** Takayasu arteritis, a granulomatous large vessel vasculitis, affects the aorta and its main branches, considered a rare vasculitis with low incidence in adults (1.11 per million person-years), and no accurate pediatric incidence data worldwide available. It’s considered of etiology unknow and multifactorial.

Clinical manifestations will be according to the stage of the disease, acute, stenotic, and fibrotic. First with constitutional features such as fever, weight loss, malaise, fatigue, that could delay diagnosis and treatment. The most common symptoms in children are hypertension, elevated phase reactants and constitutional features.

**Objectives:** Present the case of a pediatric patient with Takayasu artetitis in Mexico.

**Methods:** 11-year-old female was admitted to a tertiary hospital in Northeast Mexico, with 8-month history of fatigue and palpitations, diagnosed and treated as iron deficiency anemia, without improvement. Her symptoms continued, addingv headache, abdominal pain, vomiting, tachycardia, and dyspnea. No past medical history significant, no contact with tuberculosis.

Clinical examination revealed pallor, bruits in carotid, supra/subclavian and abdominal areas, jugular engorgement, hyperdynamic precordium, multifocal systolic murmur, perophereal pulses present with decreased in popliteal, pedis and radial, and high intensity pulses in the upper right extremity. Blood pressure was 161/96 mmHg and 130/76 in her right arm and right leg respectively.

**Results:**

Laboratory tests showed elevated acute phase reactants, microcytic anemia, mild elevated platelet count, renal and liver function test were normal, negative tuberculosis test, serology and cultures for bacteria and virus were negative.

Angiotomography showed severe stenosis in the left common carotid artery, left subclavian artery, right subclavian artery, origin celiac trunk, origin of superior mesenteric artery and origin left renal artery, dilated left ventricle.

Diagnosis of Takayasu arteritis was established, fulfilling criteria of angiographic abnormalities, pulse deficit, blood pressure discrepancies, bruits in major arteries, hypertension, and elevated acute phase reactants, dilated left ventricle with concentric hypertrophy and severe systolic dysfunction, considered a type V.

Treatment started with induction to remission, methylprednisolone pulses for three days, followed with prednisone 1mgkgday, and intravenous cyclophosphamide, to date 4 doses.

**Conclusion:** Takayasu arteritis is a complex disease with nonspecific symptoms at onset, which predisposes to long-term complications. Its classified according to anatomical involvement, considering type IV and V the most common in pediatric patients.

The objetive of the treatment is to avoid complications, considering first line treatment glucocorticoids and immunosuppression, the second one more common in children, considering the use of cyclophosphamide in the presence of extensive disease. Recently, the use of biological therapy such as TNF, IL6, and JAK inhibitors is being considered.

**Patient Consent**

Yes, I received consent

**Disclosure of Interest**

None Declared

**References**

1. Aeschlimann FA, Yeung RSM and Laxer RM (2022) An Update on Childhood-Onset Takayasu Arteritis.Front. Pediatr. 10:872313.doi: 10.3389/fped.2022.872313
2. Aeschlimann FA, et al. Presentation and Disease Course of Childhood-Onset Versus Adult-Onset Takayasu Arteritis. Arthritis Rheumatol. 2019 Feb;71(2):315-323. doi: 10.1002/art.40690. Epub 2018 Dec 29. Arthritis Rheumatol. 2020 Jun;72(6):1035
3. Mendiola K, Portillo AC, Galicia A, García JA, Maldonado R, Faugier E.Type III Takayasu's arteritis in a pediatric patient. Case report and review of the literature. Reumatol Clin. 2012 Jul-Aug;8(4):216-9. doi: 10.1016/j.reuma.2011.11.008. Epub 2012 Mar 31.

### Vasculitides

#### 513 Coronavirus OC43-related vasculitis: a case series

##### Maria Cristina Maggio ^1^, Carlotta Magno^1^, Filippo Rimi^1^, Chiara Barberi^1^, Ciro Borreano^1^, Giovanna Centonze^1^, Chiara Cannata^1^, Clotilde Alizzi^2^, Giovanni Corsello^1^

###### ^1^PROMISE "G. D'Alessandro", University of Palermo; ^2^UOC Pediatria generale, Children Hospital "G. Di Cristina", ARNAS Palermo, Palermo, Italy

####### **Correspondence:** Maria Cristina Maggio

*Pediatric Rheumatology 2024*, **22(2)**: PReS24-ABS-1608

**Introduction:** Between human Coronaviruses the OC43 strain, linked to the famous “Russian flu” (1889-95), is associated with clinical manifestations resembling seasonal colds.

**Objectives:** Our study aims to highlight a possible correlation between some rare forms of vasculitis and Coronavirus OC43 infection, hypothesizing the pathogenetic role of an immune cross-reactivity with SARS-CoV-2.

**Methods:** We report two clinical cases of vasculitis, different in onset and presentation pictures, but sharing the same possible trigger: the Coronavirus OC43 infection.

Patient 1: a 2-year-old female, showed edema and vasculitis purplish red, swollen raised and painful skin manifestations (up to 2 cm in diameter), on the right auricle and left ankle, with bilateral hemorrhagic conjunctivitis, pharyngitis and enanthema. 3 days before, she had loss of appetite, vomiting, cough, rhinitis without fever.

Blood examinations showed: neutrophilic leukocytosis (12,830/15,360), lymphocytopenia (600), thrombocytosis (731,400); normal coagulation and D-Dimer; increase of CRP (0.98 mg/dL), ESR (29), fecal occult blood positivity. Autoantibodies, C3, C4 were normal. Echocardiogram and chest X-ray excluded cardiac involvement and pleural/pericardial effusion. Abdominal ultrasound documented a mild ascites. PCR on pharyngeal and ocular swabs demonstrated the human Coronavirus OC43.

The diagnostic hypothesis of “acute hemorrhagic edema of infancy” (AHEI) or “Seidlmayer's purpura” recognized a more severe form of the disease for the intestinal involvement, ascites; bilateral hemorrhagic conjunctivitis, not previously described in association with AHEI.

Intravenous methylprednisolone (2 mg/kg/day) allowed the progressive resolution of the clinical picture.

Patient 2: a 3-year-old female was admitted simultaneously with the patient 1, for the appearance of pain and functional impotence of both ankles, swollen with purpuric lesions (up to 0,5 cm in diameter), extending to the lower limbs.

Blood tests, including coagulation study, urinalysis and SOF, were normal, except for increased PCR values (8.49 mg/dL).

Autoimmunity was negative; C3 was increased (185 mg/dL). Ultrasound confirmed the inflammatory involvement of ankles, tibio-tarsal joints, peroneal tendons. PCR on pharyngeal swab was positive for the human Coronavirus OC43. She was treated with Ibuprofen, with gradual regression of pain, swelling and hemorrhagic manifestations.

**Results:** SARS-CoV-2 as other Coronavirus can trigger vasculitis through the direct cytopathic invasion of the endothelium or, indirectly, through an autoimmune pathway.

The 2 forms of vasculitis, although different, were triggered by Coronavirus OC43.

**Conclusion:** Are we facing a mutated, more aggressive OC43 strain? Considering the mechanisms of cross-reactivity, not always protective, between different strains of human Coronavirus, could immunity developed against SARS-CoV-2 play a role in the increased virulence demonstrated by OC43?

There are mechanisms of cross-reactivity between different Coronavirus strains (e.g., Sars-COV-2 and OC43) that could explain more severe disease pictures in some patients and are therefore worthy of further studies.

**Patient Consent**

Yes, I received consent

**Disclosure of Interest**

None Declared

**References**

1. Maggio MC, et al. Idiopathic Seidlmayer's Purpura: A Case Report. Case Rep Dermatol. 2014;6(2):150-3. doi: 10.1159/000362754.
2. Batu ED, et al. The Characteristics of Patients With COVID-19-Associated Pediatric Vasculitis: An International, Multicenter Study. Arthritis Rheumatol. 2023;75(4):499-506. doi: 10.1002/art.42411.
3. Maggio MC et al. Ten-month follow-up of patients with covid-19 temporally related multi-system inflammatory syndrome in children: the experience of the children hospital of Palermo. Ital J Pediatr. 2023. doi: 10.1186/s13052-023-01416-9.

### Vasculitides

#### 514 Angiography-positive non-progressive CNS vasculitis in a postpartum adolescent

##### Camilo A. Vargas-Rincon

###### Pediatrics, Universidad del Valle, Cali, Colombia

*Pediatric Rheumatology 2024*, **22(2)**: PReS24-ABS-1554

**Introduction:** Angiography-positive non-progressive CNS Vasculitis **(NP-cPACNS)** affects medium-large sized vessels and the diagnosis is confirmed on angiography. It is a monophasic disease. Children with NP-cPACNS typically present arterial ischemic stroke. Prompt and accurate diagnosis of CNS vasculitis is essential to prevent irreversible brain damage, and to secure precise treatment decisions. An adequate diagnostic approach must be made to differentiate from vasoconstriction syndromes. There are no reports of isolated CNS vasculitis in a postpartum adolescent.

**Objectives:** Describe a case of isolated CNS vasculitis in a postpartum adolescent. Raise awareness among non-rheumatological treating physicians of the importance of other diagnostic considerations in cases of acute neurological deficit.

**Methods:** This is a case report, a review of the clinical history was carried out, obtaining the clinical, laboratory and imaging data of the patient. Follow-up was carried out for 6 months by rheumatology and pediatric neurology.

**Results:** 15-year-old patient, originally from Venezuela. G1. Pregnancy and childbirth without complications. No significant history, no headache episodes during pregnancy, no migraine, no hypertensive disorder of pregnancy, nor medications frequently associated with vasoconstriction. On the 7th day postpartum she presented paresthesia and weakness in the right hemibody, dysarthria and facial paralysis.

Without alteration of the state of consciousness. Brain CT without masses, edema or hemorrhages. MRI angiography with hyperintense lesion on FLAIR in the left putamen nucleus and left corona radiata with diffusion restriction. Angiographic window with alteration of the continuity of the a. left middle brain. 6-vessel angiography with multiple stenotic areas in the M2 segment of the a. left middle cerebral, with post-stenotic dilations, which generate delay in parenchymal arterial time.

Blood counts, serum electrolytes, and liver and renal function tests and cerebrospinal fluid normal. Negative APS profile. Normal echocardiogram. Hemoglobinopathies negative screening.

Management was started with pulse steroids of 30 mg/kg/day and maintenance of prednisolone at 2 mg/kg/day. Aspirin 100mg/day. Calcium + vitamin D supplementation. She presented progressive recovery of the neurological deficit, imaging control in 3 months without evidence of new vascular lesions or ischemic events in other areas.

**Conclusion:** Inflammatory diseases of the CNS should be taken into account in all pediatric patients with acute neurological deficit, vasoconstriction syndromes are not always the explanation for a postpartum ischemic event. Establishing a work team with neurology, radiology and rheumatology is essential for an accurate diagnosis and treatment.

**Patient Consent**

Yes, I received consent

**Disclosure of Interest**

None Declared

**References**

1. Aneesh B. Singhal and Richard A. Bernstein. Postpartum Angiopathy and Other Cerebral Vasoconstriction Syndromes Neurocrit. Care 2005;3:91–97
2. Hubert de Boysson, et al. Primary angiitis of the CNS and reversible cerebral vasoconstriction syndrome. Neurology. 2018;00:e1-e11. doi: 10.1212/WNL.0000000000006367

### Vasculitides

#### 515 Diagnostic and treatment dilemma of a suspected case of primary angiitis of the central nervous system, in a patient with post-transplant lymphoproliferative disease

##### Karolina Warciak^1^, Marah Shaikh Yousef^1^, Kathy Gallagher^2^, Emma J. MacDermott^1^, Orla Killeen^1^, Peter McCarthy^3^

###### ^1^Rheumatology, National Centre for Paediatric Rheumatology, CHI-Crumlin, Dublin; ^2^Rheumatology, National Centre for Paediatric Rheumatology, CHI-Crumlin, Ireland; ^3^Haematology and Oncology, CHI -Crumlin, Dublin, Ireland

####### **Correspondence:** Karolina Warciak

*Pediatric Rheumatology 2024*, **22(2)**: PReS24-ABS-1603

**Introduction:** We present a case of suspected Primary Angiitis of the Central Nervous system (PACNS), in a patient with Ebstein Barr Virus (EBV) driven Post Transplant Lymphoproliferative Disease (PTLD). We aim to highlight the importance of multi-disciplinary (MDT) approach to diagnosis and management.

**Objectives:** PACNS is a rare paediatric disease, now better recognised as a cause of cerebrovascular accident in childhood^1,2^. There are no standardised treatment protocols. Many reports and studies show a better response with Mycophenolate Mofetil (MMF) maintenance therapy, after an initial cyclophosphamide and glucocorticoid therapy^2,3,4^.

**Methods:** 15-year-old female presented with left-sided limp and facial droop and weakness of left upper limbs eight days post commencement of PTLB treatment. This is on a complex background of renal failure secondary to congenital urogenital malformation, peritoneal dialysis in 2020 and haemodialysis until 2022. First renal transplant in 2021 failed immediately intraoperatively. Second renal transplant was successful in 2022, with MMF and Tacrolimus maintenance. MMF was discontinued in October 2023 due to PTLD diagnosis.

Magnetic Resonance Imaging (MRI) showed multifocal acute infarcts in the right middle cerebral artery territory. Patient was treated with high dose intravenous Methylprednisolone followed by oral Prednisolone. Over the following two weeks patient had two more left-sided neurological events. Repeat imaging showed new areas of infarction relying on blood supply from collateral vessels consistent with PACNS, with no evidence of systemic vasculitis. A brain biopsy was deemed too high risk at present.

**Results:** As a result of increasing concern for further cerebrovascular injury, she underwent cerebral by-pass surgery. Six weeks post-surgery, patient had left-sided hand tremor, with new areas of acute ischaemia on MRI of brain with contrast.

She has now completed her full treatment, of two doses of Rituximab and six doses of Cyclophosphamide. Remains on oral Prednisolone.

We now face a treatment dilemma, as maintenance options are limited due to the risk of EBV reactivation and PTLB recurrence with additional immunosuppression, especially with MMF.

**Conclusion:** PACNS is an exceptionally rare condition that can lead to permanent neurological damage and often proves fatal. Patients with co-morbidities pose greater challenges. Prompt diagnosis, aggressive treatment and comprehensive MDT approach is paramount.

**Patient Consent**

Yes, I received consent

**Disclosure of Interest**

None Declared

**References**

1. Romero FVP, Rincón ADC, Maldonado ASD. Childhood primary vasculitis of the central nervous system: Case report and literature review. Revista Colombiana de Reumatología (English Edition). 2018 Oct;25(4):301–6.
2. Zinovia-Maria Kefalopoulou, Stamatis-Nick Liossis, Sagona T, Dimitra Veltsista, Petros Zampakis, Pantelis Kraniotis, et al. An ischemic stroke as the presenting manifestation of rapidly progressive primary angiitis of central nervous system in a 17-year-old boy. Journal of Neuroimmunology. 2020 Apr 1;341:577190–0.
3. Sen ES, Leone V, Abinun M, Forsyth R, Ramesh V, Friswell M, et al. Treatment of primary angiitis of the central nervous system in childhood with mycophenolate mofetil. Rheumatology. 2010 Jan 25;49(4):806–11.
4. Beelen J, Benseler SM, Dropol A, Ghali B, Marinka Twilt. Strategies for treatment of childhood primary angiitis of the central nervous system. Neuroimmunology and Neuroinflammation. 2019 May 3;6(4):e567–7

### JIA (oligo, poly, psoriatic)

#### P522 A case of turner syndrome associated with Juvenile idiopathic arthritis

##### Azadeh Z. Mirzaee^1^, Reza Shiari^2^, Shabnam H. Ghotbabadi^3^, Niloofar Shashaani^2^, Pooneh Tabibi^2^

###### ^1^Pediatrics, Pediatric Respiratory Disease Research Center (PRDRC), Masih Daneshvari Hospital; ^2^Pediatrics, Shahid Beheshti University of Medical Sciences, Tehran; ^3^Pediatrics, Shiraz Medical University, Namazi Hospital, Shiraz, Iran, Islamic Republic Of

####### **Correspondence:** Azadeh Z. Mirzaee

*Pediatric Rheumatology 2024*, **22(2)**: PReS24-ABS-1510

**Introduction:** Juvenile Idiopathic Arthritis (JIA) is an autoimmune, inflammatory joint disease. It is the most common rheumatic disease in children and one of the more common chronic illnesses of childhood. Turner’s Syndrome (TS) is a condition characterized by complete or partial monosomy of the X chromosome and defined by combination of phenotypic features. Half of the patients with Turner’s syndrome have a 45X hromosome complement. Turner syndrome occurs in approximately 1 in 2000 to 1 in 2500 live female births.

**Objectives:** Recent studies have suggested that there is a higher incidence of Autoimmune Diseases (AD) in people with Turner syndrome. In this paper, we report a 4-year-old Iranian girl with Turner syndrome who was complicated with JIA.

**Methods:** The patient is a 4-year-old Iranian girl born after an uneventful pregnancy and normal delivery. She was the third child of non-consanguineous, phenotypically normal parents. At the age of 2 years dysmorphic features were noticed, She also had failure to thrive, ambiguous genitalia and developmental delay. She was diagnosed with Turner syndrome via genetic study which revealed mixed gonadal dysplasia compatible with 45XO/46XY, compatible with abnormal chromosomal complements with mosaicism.

At the age of 4 years, she was referred to Mofid Children Hospital complaining of progressive left knee swelling and pain for the past two months and inability to walk. She didn’t have any history of trauma to the affected knee. In physical examination, the appearance of left knee was abnormal with swelling, tenderness and mild warmness. The Ballottement test of the left knee was positive and she also had reduced range of motion on it.

**Results:** the patient fulfilled the International League of Associations for Rheumatology (ILAR) criteria for a diagnosis of oligoarticular JIA. Her ophthalmology examination was normal and there were no evidence of uveitis oriridocyclitis. She was treated with intra-articular injection of Triamcinolon and oral Naproxen. The patient was also referred to Endocrinologist because of poor weight gain and short stature.

**Conclusion:** The underlying immunopathogenic mechanism of this association remains partially unexplained.

Studies have suggested a higher responsibility of the X chromo-some abnormalities. For instance, the long arm of the X chromosome hosts a MHC-locus, so the loss of that region in Turner syndrome may cause a deficiency in immune regulation.

**Date of birth::** décembre 3

**Patient Consent**

Yes, I received consent

**Disclosure of Interest**

None Declared

### JIA (oligo, poly, psoriatic)

#### P526 Application of omeract synovitis ultrasound scoring system in children with JIA in Nicaragua

##### Karla Vanessa Jiron Mendiola

###### Pediatrics, Hospital Vivian Pellas, Managua, Nicaragua

*Pediatric Rheumatology 2024*, **22(2)**: PReS24-ABS-1605

**Introduction:** US has the great advantage that it can be performed in the same consultation, which facilitates immediate comparison with the patient's clinical and examination data in cases of suspected or doubtful diagnosis. Consequently, it is essential to facilitate teaching regulated by a competitive curriculum in rheumatological US, to have a mid- or high-range ultrasound machine, and to fully understand the adjustment parameters.

MRI may not be as accessible in consultation as US, but it is a very useful imaging technique for both diagnosis and patient follow-up.

Juvenile idiopathic arthritis continues to be a very broad field of research, mainly in populations in which timely detection and approach is already being carried out, however we have noted that ultrasound findings, mainly subclinical degree of synovite, can contribute to timely diagnosis mainly by OMERACT score. .

**Objectives:** Describe ultrasound findings in children with JIA in Nicaragua

**Methods:** Patients with inflammatory joint pain and JIA were detected, independent of classification, and evaluation was performed by a musculoskeletal radiologist using the OMERACT pediatric ultrasound (US) synovitis definitions and scoring system.

**Conclusion:** We found that the omeract score performed by a musculoskeletal radiologist is of great support in an evaluation and diagnosis of JIA, especially in subclinical cases.

**Date of birth::** février 16

**Patient Consent**

Yes, I received consent

**Disclosure of Interest**

None Declared

### Scleroderma and related syndromes

#### 520 Delay in diagnosis of mixed connective tissue disease in an a adolescent libyan girl

##### Awatif A. Abushhaiwia^1^, Angelo Ravelli^2^, Nairuz B. M. Abushhiwa^3^, Abdelmoneim A. Benarous ^4^

###### ^1^ Paediatric Rheumatology, ripoli Children's Hospital, Faculty Of Medicine, University Of Tripoli, Tripoli , Libya; ^2^Division of Paediatric Rheumatology and Scientific Institute for Research and Health Care, IRCCS Istituto Giannina Gaslini, Genoa, Italy; ^3^Pathology department, Faculty Of Medicine, University Of Tripoli; ^4^Cardiology department, Tripoli Children's Hospital, Tripoli , Libya

####### **Correspondence:** Awatif A. Abushhaiwia

*Pediatric Rheumatology 2024*, **22(2)**: PReS24-ABS-1054

**Introduction:** MCTD is an extremely rare multisystem disease with overlapping features of rheumatoid arthritis (RA), SSc, SLE, and dermatomyositis, along with the presence of anti-U1-ribonucleoprotein (RNP) antibodies in high titers

**Objectives:** We report a rare case of MCTD. Identified in an adolescent girl, misdiagnosed since childhood.

**Methods:** A case report is described

**Results:** A 21-year-old Libyan girl, born to consanguineous parents, has no family history of note. She came to our attention with skin tightness over the last 2 years associated with contracture of her fingers of both hands (deformity of her fingers), poor appetite, loss of weight of about 22 kg, fatigue, Reynaud’s phenomenon, oral ulcers, muscle pain, She also experienced shortness of breath, coughing, and joints pain for the last 2 years. She had a rough clinical course of disease that required her several admissions and was misdiagnosed ; the last one was in 2022 because of huge hepatosplenomegaly and ascities for bone marrow that were normal.During the whole of these years, but no one has noticed she has a connective tissue disease.On physical examination she was short for her age, underweight, delayed puberty, Bp = 101\74, tightness of her skin of face (mouth fish appearance), both upper and lower extremities, trunk and back, deformity of her fingers of both hands, huge hepatosplenomegally no ascities.Laboratory tests showed leukocytosis microcytic hypochromic anemia, ESR high elevated 137mm\hr, CRP was positive, GPT 46 little bit elevated but GOT 106 was high elevated,high elevated CPK = 2489 U/L, LDH = 465 U/L, urea = 13 mg/dl, creatinine = 0.4 mg/dl, normal TSH, free T4. Urine analysis demonstrated protein+++, very high urine for protein \creatinine ratio =6% Immunoprofile: ANA was very elevated (1:5120 titter), positive RF was 31 U\ml, and antidsDNA abs were negative. U1-snRNPab positive with high titter 53 (N <5); anti-SMab was negative; she was positive for SAA (RO 52, 60) with a high titter >200 U\ml but antiSSB La abs, Scl70, Jo1, APL abs were negative and normal complement. An echocardiography finding was mild pulmonary hypertension. Renal biopsy revealed vascular nephropathy associated with segmental and focal Hayalin disease of scleroderma. Endoscopy revealed severe oesophageal candidiasis and no oesophageal varices. She was being treated with prednisolone 25 mg, then tapered to 10 mg, MM 500 mg twice daily, Enalapril tab 2.5mg once per day ,furosemide tab 20mg once per day, aldoctone tab 25 mg, and plaquenil tab 100 mg once per day. Fluconazol capsule, 150 mg, once a day for 2 weeks. Recently added MTX 10 mg/wk. The disease was partially under control with these medications.

**Conclusion:** This report emphasizes the importance of educating practitioners about MCTD. Early recognition is key as a delay in diagnosis can result in potentially serious complications.

**Acknowledgments**

The authors would like to thank Professor Angelo Ravelli for his invaluable help.

**Patient Consent**

Yes, I received consent

**Disclosure of Interest**

None Declared

### Scleroderma and related syndromes

#### 521 Morphea in two cases of children; libyan experience

##### Awatif A. Abushhaiwia^1^, Nairuz B. M. Abushhiwa^2^

###### ^1^Paediatric Rheumatology, Tripoli Children's Hospital, Faculty Of Medicine, University Of Tripoli; ^2^Pathology, Faculty Of Medicine, University Of Tripoli, Tripoli , Libya

####### **Correspondence:** Awatif A. Abushhaiwia

*Pediatric Rheumatology 2024*, **22(2)**: PReS24-ABS-1068

**Introduction:** Morphea (jLS) is a rare pediatric disease, a group of autoimmune diseases in which there is deposition of collagen in the skin and sometimes other organs as well. Skin sclerosis may lead to significant contractures or growth retardation, with a risk of severe disfigurement and functional impairment. Scleroderma treatment is targeted at controlling inflammation and managing specific problems. Early diagnosis can significantly improve outcomes.

**Objectives:** To describe two cases of scleroderma

**Methods:** Retrospective review of medical records of two patients with localized scleroderma

**Results: Pateint#1 is** A 10-year-old Libyan girl presented with a one-year history of morphea of the right arm, forearm, and left thigh. Her condition started as a single erythematous indurated lesion over the right forearm and has a history of vitiligo for a year; her mother as well has had viltigo for 10 years. A deep-punch biopsy of the initial lesion demonstrated histological findings consistent with LS (morphea). There were no other systems involved. On examination, she has only cutenous findings: three deep circumscribed morphea lesions over her right arm (3 cm x 12 cm), forearm (7 cm x 4 cm), left thigh (6 cm x 4 cm), and vitligo lesions.Other systemic examinations, including musculoskeletal, were unremarkable.All labratory tests were within the normal range icluding ANA, RF were negative,due to this negative ANA value no other extractable nuclear antigens were tested .After starting oral prednisone and methotrexate therapy (15 mg subcutaneously weekly), she developed a new lesion on her right upper lip. then added mycophenolate mofetil (MMF) 500mg twice a day for the past two months, which showed partial improvement.

**Pateint#2** is a 12-year-old Libyan boy who presented with a 3-year history of inability to walk and getting up from a sitting position. A physical examination revealed tightness in the majority of the skin of his body, including the neck and back, trunk, and upper and lower extremities. He had lower limb atrophy and extreme wasting of thigh and calf muscles, as well as a contracture of the knees, ankles, and fingers of his hands. His investigations revealed negative rheumatoid factor, positive antinuclear antibody (ANA), mild microcytic hypochromic anemia, normal ESR, and CRP. He’s on daily prednisone that was tapered gradually, calcium, Vit D, iron supplements, weekly methotrexate, and daily folic acid.

**Conclusion:** JLS is rare in children and has a high risk of misdiagnosis, significant disfigurement, functional impairment, and emotional impact. While systemic treatment with methotrexate, corticosteroids, and mycophenolate mofetil is available and often effective, we need further studies of treatment effects or to develop new medications for this disease.

**Patient Consent**

Yes, I received consent

**Disclosure of Interest**

None Declared

### Scleroderma and related syndromes

#### 523 Severe scoliosis secondary to Juvenile localised scleroderma

##### Emily Willis^1^, Eleanor Heaf^2^, Clare E. Pain^2^

###### ^1^Paediatric and Adolescent Rheumatology, Royal Manchester Children's Hospital , Manchester; ^2^Paediatric and Adolescent Rheumatology , Alder Hey Children's Hospital, Liverpool, United Kingdom

####### **Correspondence:** Emily Willis

*Pediatric Rheumatology 2024*, **22(2)**: PReS24-ABS-1112

**Introduction:** We present the case of an 8 year old girl with juvenile localised scleroderma (JLS) and scoliosis. Whilst extracutaneous manifestations (ECM) are common in JLS^1^ , scoliosis is not widely reported ^2,3^. Diagnostic delay is recognised as a barrier to timely care ^4^. In addition, waiting times for specialist paediatric care are currently very high in the UK, with over 400,000 children awaiting consultant led treatment, 17,991 of these waiting over a year.^5^

**Objectives:** Highlight the importance of prompt diagnosis and management of scleroderma to prevent complications. Raise awareness of scoliosis as a complication of JLS.

**Methods:** Case report from medical records.

**Results:** Skin changes were first noticed at the age of 5 years. The child was seen by a dermatologist at age 6 years and diagnosed with morphoea. She was referred to paediatric dermatology for consideration of systemic immunotherapy. First appointment with paediatric dermatologist was 2 years later. She was then referred to paediatric rheumatology. First review with paediatric rheumatology took place 3 years after the onset of skin changes. There were extensive skin changes consistent with JLS. Scoliosis was present, Cobb angle 33°. At first visit, modified Localised Skin Severity Index (mLoSSI) 10, Localised Scleroderma Damage Index (LoSDI) 18, physician global assessment (PGA) activity 40, PGA damage 60. Systemic treatment was commenced with IV methylprednisolone and subcutaneous methotrexate. MRI spine confirmed scoliosis but no other abnormality. Following review by spinal and plastic surgeons, it was concluded that the scoliosis was secondary to JLS causing a soft tissue tether. Skin is improving on treatment and surgery is planned. Recent LoSCAT: LoSSI 1, LoSDI 24, PGA activity 5, PGA damage 55.

**Conclusion:** This case highlights a rare but significant complication of JLS, contributed to by delayed initiation of systemic treatment. Early recognition of JLS and referral to a specialist centre is vital to prevent complications. In the UK, delayed access to specialist care due to rising waiting lists has been highlighted by RCPCH as a significant problem.

**Patient Consent**

Yes, I received consent

**Disclosure of Interest**

None Declared

**References**

1. Li S, Higgins G, Chen M, et al. Extracutaneous Involvement Is Common and Associated with Prolonged Disease Activity and Greater Impact in Juvenile Localized Scleroderma. Rheumatology (Oxford) 2021 Dec 1;60(12):5724-5733. doi: 10.1093/rheumatology/keab238
2. Burke S. Scoliosis and morphoea. Spine 1986 Jan-Feb;11(1):71-2. doi:10.1097/00007632-198601000-00021
3. Dave J N et al. Linear scleroderma with bilateral lesions, contractures and kyphoscoliosis. Indian J Dermatol Venereol Leprol 1997 Jan-Feb;63:66-68.
4. Stubbs, L.A. *et al.* Barriers to care in juvenile localized and systemic scleroderma: an exploratory survey study of caregivers’ perspectives. Pediatr Rheumatol J Online 2023 Apr 25;21(1):39.doi: 10.1186/s12969-023-00819-6.
5. https://www.rcpch.ac.uk/news-events/news/record-high-over-400000-children-waiting-treatment-amidst-child-health-crisis

### Scleroderma and related syndromes

#### 516 When the pieces of the puzzle come together: a challenging case of overlap syndrome

##### Aikaterini Koryllou, Raffaella Carlomagno, Caroline Schnider, Katerina Theodoropoulou

###### Pediatric Immunology-Rheumatology and Allergology Unit, Department of Pediatrics, Lausanne University Hospital and University of Lausanne, Lausanne, Switzerland

####### **Correspondence:** Aikaterini Koryllou

*Pediatric Rheumatology 2024*, **22(2)**: PReS24-ABS-1805

**Introduction:** Connective tissue disorders in children can overlap in various ways, making diagnosis challenging. Patients may present with complex clinical features of more than one disease without satisfying the diagnostic criteria and fulfilling simultaneously the diagnostic criteria of more diseases.

**Objectives:** We describe the case of an overlap connective tissue disease in a young patient initially diagnosed as juvenile systemic sclerosis (SSc).

**Methods:** Case report

**Results:** A 15-year-old girl originally from Sri-Lanka, was admitted with a 3-month history of sclerodactyly, digital pitting scars, skin sclerosis, fatigue, dysphagia and weight loss, and a Raynaud’s phenomenon during the last 3 years. She further developed multiple ulcerative lesions of fingers/forearms/thighs. Skin histopathology showed superficial and deep perivascular lymphoplasmacytic infiltration, direct immunofluorescence revealed no C3, IgA, IgG, and IgM deposition. Autoantibody screening revealed positive ANA, anti-Scl-70, anti-SSA, anti-Sm and anti-RNP antibodies. Anti dsDNA and myositis antibodies panel were negative. Chest CT scan revealed signs of interstitial lung disease (ILD) and multiple adenopathies. An excisional biopsy of an affected lymph node was suggestive of Kikuchi-Fujimoto disease. Capillaroscopy revealed enlarged capillaries, increased tortuosity and avascular areas. A whole-body MRI showed features of proximal inflammatory polymyositis of lower limbs. She was diagnosed with SSc and treated with oral steroids, mycophenolate mofetil and calcium channel blocker with poor response, particularly on the cutaneous features. Antifibrotic agent, antiplatelet and tocilizumab were then added without response. Her features seeming overlapping with juvenile systemic lupus erythematosus and dermatomyositis, she was then treated as an overlapping disease. Tocilizumab was stopped and she was started on hydroxychloroquine, monthly intravenous immunoglobulins, rituximab and tadalafil. She progressively improved with resolution of skin sclerosis and ulcers, remission of joint stiffness and stability of ILD.

**Conclusion:** This case highlights the importance of increasing awareness of overlap syndromes in young patients. The association should be recognized early for prompt initiation of appropriate treatment that can prevent important morbidity and mortality.

**Patient Consent**

Yes, I received consent

**Disclosure of Interest**

None Declared

### Systemic lupus erythematosus and antiphospholipid syndrome

#### 524 Efficacy and safety of cyclic steroid pulses with sequential therapeutic schemes of belimumab and rituximab in severe childhood lupus: case report

##### Fatimah Alkhars^1^, Shahad A. Alansari^2^, Suliman M. Al-Mayouf^3^

###### ^1^Pediatric, Maternity and Child hospital, Alhassa; ^2^Pediatric, Hera General Hospital, Makkah; ^3^Pediatric, King Faisal Specialist Hospital & Research Center, Al-Riyadh, Saudi Arabia

####### **Correspondence:** Fatimah Alkhars

*Pediatric Rheumatology 2024*, **22(2)**: PReS24-ABS-1210

**Introduction:** Childhood-onset systemic lupus erythematosus (cSLE) is a systemic autoimmune disease with a complex genetic and immunological background. Sever cSLE can be challenging, and the lack of solid evidence in the pediatric population makes it more difficult. Compared to adult SLE, cSLE tends to have a more aggressive and active course as the disease progresses. Patients with cSLE often require higher doses and combined immunosuppressant medications. As a result, severe cSLE can be challenging, and the lack of solid evidence in the pediatric population makes it more difficult.

**Objectives:** This report describes our experience treating severe cSLE with cyclic steroid pulses and sequential therapeutic schemes of Belimumab and Rituximab. Such a treatment regimen has not previously been reported.

**Methods:** We report a 12-year-old female with severe SLE and lupus nephritis which was refractory to conventional treatment and had steroid-related complications successfully treated with cyclic steroid pulses as well as sequential therapeutic schemes of Belimumab and Rituximab.

**Conclusion:** Our findings suggest that the RTX and BLM combination therapy appears to be safe and successful in achieving a clinically significant response in cSLE, thus representing a valid option for the treatment of severe and refractory SLE. Additionally, intermittent (cyclic) intravenous methylprednisolone pulse therapy with low-dose oral prednisone therapy is an excellent option in severe cSLE, and it can be the future standard of care.

**Patient Consent**

Yes, I received consent

**Disclosure of Interest**

None Declared

**References**

1. Trindade V, Carneiro-Sampaio M,1 Bonfa E, Silva C. An Update on the Management of Childhood-Onset Systemic Lupus erythematosus. Paediatr Drugs. 2021;23(4):331–347.
2. Chen F, Zheng Y, Chen X, Wen Z, Xu Y, Yang J, et al. Belimumab in childhood systemic lupus erythematosus: A review of available data. Front. Immunol. 2022;13:940416.
3. Pan L, Liu J, Liu C, Guo L, Punaro M, Yang S. Childhood-onset systemic lupus erythematosus: characteristics and the prospect of glucocorticoid pulse therapy. Front. Immunol. 2023;14:1128754.
4. Elshaer R, Jaber S, Odeh S, Arbili L, Al-Mayouf S. Safety and efficacy of biologics in childhood systemic lupus erythematosus: a critical systematic review. Clin Rheumatol. 2024;43:863–877 2024.
5. Petricca L, Gigante M, Paglionico A, Costanzi S, Vischini G, Mario C, et al. Rituximab followed by belimumab controls severe lupus nephritis and bullous pemphigoid in systemic lupus erythematosus refractory to several combination therapies. Front Med. 2020;7:553075.
6. Porta S, Danza A, Saavedra M, Carlomagno A, Goizueta M, Vivero F, et al. Glucocorticoids in systemic lupus erythematosus. ten questions and some issues. J Clin Med. 2020;9(9):2709.
7. Sciascia S, Radin M, Roccatello D, Sanna G, Bertolaccini ML. Recent advances in the management of systemic lupus erythematosus. F1000Res. 2018;29;7:F1000.
8. Atisha‐Fregoso Y, Malkiel S, Harris K, Byron M, Ding L, Kanaparthi S, et al. Phase II randomized trial of rituximab plus cyclophosphamide followed by belimumab for the treatment of lupus nephritis. Arthritis Rheumatol. 2021;73(1):121–131.

### Systemic lupus erythematosus and antiphospholipid syndrome

#### 527 Juvenile lupus peritonitis: navigating the challenges of diagnosis and treatment in an unusual debut presentation – a case study

##### Napapas Yothakol

###### Department of Pediatrics, Somdech Phra Pinklao Hospital, Bangkok, Thailand

*Pediatric Rheumatology 2024*, **22(2)**: PReS24-ABS-1440

**Introduction:** Lupus peritonitis is a rare manifestation of systemic lupus erythematosus (SLE) characterized by peritoneal inflammation. It affects less than 5% of abdominal manifestations in SLE cases. Early identification and appropriate management are crucial for optimizing outcomes and minimizing long-term morbidity.

**Objectives:** To comprehensively explore clinical features and therapeutic interventions for lupus peritonitis, a relatively uncommon manifestation in SLE patients.

**Methods:** Case report

**Results:** A previously healthy 14-year-old girl presented with 3-month history of fatigue, low grade fever, intermittent diarrhea and significant weight loss. Notably, no abnormal skin lesion, edema, joint pain or neuropsychiatric symptoms were reported. She denied family history of tuberculosis or recent illnesses. Physical examination revealed a body temperature of 37.9 °C, tachycardia, markedly pale conjunctivae, palpable bilateral cervical lymphadenopathy. No hepatosplenomegaly was observed without abdominal tenderness.

Laboratory tests revealed microcytic anemia (hemoglobin: 2.6 g/dL) without evidence of hemolysis, normal white cell count and differentiation, and low platelet count (14 x 10^3^ u/L) without clumping. Renal and liver function were within normal limit, except for mild hypoalbuminemia without proteinuria. Erythrocyte sedimentation rate was notably elevated (>140 mm/hr), while C-reactive protein remained normal. Chest x-ray showed mild cardiomegaly with a small left pleural effusion. Positve ANA (>1:320) with a coarse-speckled nucleolar pattern, low C3 (49 mg/dL) and C4 (9.4 mg/dL) led to a diagnosis of SLE. Treatment with corticosteroid and anti-malarial agent was initiated after excluding infection.

Following 7 days of prednisolone, she developed rapid-onset abdominal pain and significant distension. Abdominal computed tomography scan revealed abundant ascites and diffuse enteritis without hepatosplenomegaly. Ascitic fluid analysis suggested inflammatory serositis due to a low serum ascites albumin gradient with low protein level. Culture were unremarkable, leading to diagnosis of lupus peritonitis. Pulse methylprednisolone was given for 3 consecutive days, followed by intravenous administration at a dosage of 60 mg/day with empirical antibiotics. The clinical symptoms were gradually improved. Follow-up ultrasonography 10 days post-treatment showed nearly resolved ascites. Subsequently, treatment was transitioned to oral prednisolone, with azathioprine as a steroid sparing agent.

**Conclusion:** Diagnosing lupus peritonitis, though rare, requires thorough evaluation involving clinical assessment, laboratory examinations, and imaging. Prompt recognition and intervention are essential to address peritoneal inflammation and prevent complications in affected individuals.

**Patient Consent**

Yes, I received consent

**Disclosure of Interest**

None Declared

### Systemic lupus erythematosus and antiphospholipid syndrome

#### 529 Posterior reversible encephalopathy syndrome as a rare presentation of systemic lupus erythematosus: a case report

##### Surya Prakash Joshi^1^, Vijay Shrees^1^, Prasanna Karki^1^

###### ^1^Maharajgunj Medical Campus, Kathmandu, Nepal

####### **Correspondence:** Surya Prakash Joshi

*Pediatric Rheumatology 2024*, **22(2)**: PReS24-ABS-1822

**Introduction:** Posterior Reversible Encephalopathy Syndrome (PRES) is a rare clinical-radiological entity characterized by the rapid onset of neurological symptoms accompanied by typical radiological signs of vasogenic edema predominantly in the posterior brain region. Numerous causes can contribute to PRES, including hypertension, renal failure, pre-eclampsia, and immunosuppression. Systemic lupus erythematosus (SLE) is a multisystem autoimmune disorder involving several immune dysfunctions and can affect the central nervous system. Although rare, cases of PRES have been documented in individuals diagnosed with SLE.

**Objectives:** We here describe a case of an 18-year-old female with posterior reversible encephalopathy syndrome as a rare presentation of Systemic Lupus Erythematosus

**Methods:** Case report

**Results:** 18-year-old female who was newly diagnosed with SLE and experienced generalized tonic and clonic seizures during the course of treatment inpatient in the hospital. On further evaluation for the cause of the seizure, the presence of PRES was found on the MRI Brain. The patient was managed with antiepileptic medication and kept on regular BP monitoring. For the management of SLE, due to infertility risk the patient was discharged on mycophenolate mofetil.

**Conclusion:** PRES should be kept in consideration while making a differential diagnosis for patients experiencing seizures, changes in mental state, and vision disturbances consistent with brain imaging, even though it is uncommon in SLE. Prompt recognition and management of PRES are essential to prevent potential neurological sequelae. Further studies are needed to elucidate the underlying mechanisms and optimize treatment strategies for PRES in the context of SLE.

**Trial registration identifying number:** none

**Date of birth::** juillet 23

**Patient Consent**

Yes, I received consent

**Disclosure of Interest**

None Declared

### Systemic lupus erythematosus and antiphospholipid syndrome

#### 517 Belimumab to the rescue: managing complex comorbidities in pediatric systemic lupus erythematosus

##### Angela Mauro^1^, Federica Bona^1, 2^, Alfredo Diana^1, 3^, Valentina Ansuini^1^, Martina Sandini^1^, Giulia Poretti^2^, Alessandra Cecchini^2^, Luca Bernardo^1^

###### ^1^Casa Pediatrica, Ospedale Fatebenefratelli; ^2^Pediatria, Università degli studi di Milano - Ospedale Buzzi, Milano; ^3^Pediatria, Università degli studi di Napoli Federico II, Napoli, Italy

####### **Correspondence:** Angela Mauro

*Pediatric Rheumatology 2024*, **22(2)**: PReS24-ABS-1690

**Introduction:** Systemic Lupus Erythematosus (SLE) is a chronic autoimmune disease that can involve any organ system, leading to significant morbidity and mortality. Only a few drugs are approved for use in children while the use of multiple immunosuppressants on an “off-label” basis is common.

**Objectives:** A 9-year-old girl was admitted for skin lesions, xerosis, alopecia and left knee arthritis. Blood tests showed positivity for ANA, anti-Ro, anti-SSA, anti-Sm, anti-RNP, anti-RIBP antibodies and rheumatoid factor; hypergammaglobulinemia and a significant increase of IgG and hypertransaminasemia were also found. Abdominal ultrasound highlighted hepatic steatosis. SLE was diagnosed based on 2012 SLICC diagnostic criteria and on 2019 new diagnostic criteria (non-scarring alopecia, arthritis, anti-Sm positive), SLEIDAI score:10. Furthermore, secondary Sjogren syndrome was diagnosed based on the positivity of ANA, anti-SSA and rheumatoid factor, along with deficiency in tear secretion and ultrasound hypoecoic spots of the salivary glands. The Quantiferon tested positive, and a latent tuberculosis was also hypothesized but not treated as we opted for a follow-up with an infectious disease specialist. We therefore started oral hydroxycloroquine and prednisone associated with cortisone cream to be applied on the skin lesions. Mofetil mycophenolate was added to the therapy after the worsening of the clinical symptoms, with a good response and an improvement of clinical presentation. Two months later, the patient was admitted for worsening of the symptoms with vasculitis of the palms and of the soles, worsening of alopecia and knee arthritis associated with asthenia and dizziness. Blood tests found persistent positivity of the autoantibodies. According to the clinical and laboratory presentation monoclonal antibody Belimumab was “off-label” added to the therapy, with good response. Two weeks later the patient was re-admitted for the second administration of Belimumab. The child was in good general condition, with a xerotic skin without lesions, cushingoid facies but improving alopecia. Blood tests found antibodies anti-U1RNP, anti-SSA, anti-SSB, anti-Saccharomyces Cerevisiae IgG positive. Anti-PR3, anti-MPO, anti-Sm, anti-Scl70 negative. Normal IgM and only slightly increased IgG. Rheumatoid factor was negative. Chest x-ray, abdominal ultrasound and Schirmer test, Mantoux and Quantiferon were normal. Salivary gland ultrasound highlighted a slightly heterogeneous appearance of parotids and submandibular glands without focal lesions. Therefore, the second administration of Belimumab was performed. The patient was discharged and is still undergoing rheumatological follow-up.

**Conclusion:** Managing SLE is complex, and the presence of comorbidities further complicates the treatment of this patient. For this reason, the optimal control of the disease has not yet been achieved.

**Patient Consent**

Yes, I received consent

**Disclosure of Interest**

None Declared

### Treatment

#### 525 Hydrogen gas inhalation treatment for coronary artery lesions in Kawasaki disease mice model

##### Ho-Chang Kuo ^1^, Wen-Ling Shih^1, 2^

###### ^1^Pediatrics, kawasaki disease center , Kaohsiung; ^2^National Pingtung University of Science and Technology, Pintone, Taiwan, Province of China

####### **Correspondence:** Ho-Chang Kuo

*Pediatric Rheumatology 2024*, **22(2)**: PReS24-ABS-1043

**Introduction:** Kawasaki Disease (KD) is a syndrome primarily affecting young children, typically under the age of five and is characterized by the development of acute vasculitis. Through extensive research conducted on both murine and human subjects, it has been demonstrated that the heightened levels of reactive oxygen species (ROS) play a pivotal role in the development of KD especial the coronary artery lesions (CAL).

**Objectives:** Hydrogen gas exhibits potent antioxidant properties that effectively regulate ROS production and the inflammatory response for Kawasaki disease.

**Methods:** We used lactobacillus casei cell wall extract (LCWE)-induced vasculitis in mice as an animal model of KD and treated by hydrogen gas inhalation.

**Results:** We observed significant dilatation and higher Z score of LCA in D21 and D28 in mice after LCWE treatment compared to the control group (p<0.05) and significant resolution of LCA diameter (p<0.005) and Z score (p<0.005) after treatment with inhaled hydrogen gas. We further demonstrated that higher serum IL-6 expression in mice after LCWE treatment (p<0.005) and IL-6 significantly decreased after inhaled hydrogen gas therapy (p<0.0005).

**Conclusion:** From literature review this is the first report that hydrogen gas inhalation demonstrated effective for the treatment of coronary artery dilatation in the KD murin model.

**Patient Consent**

Not applicable (there are no patient data)

**Disclosure of Interest**

None Declared

### Autoinflammatory diseases

#### 519 Comorbidity of periodic disease with hemorrhagic vaculitis. Clinical case

##### Anna Sologub, Sania Valieva, Seda Kurbanova

###### Department of Rheumatology, Morozov Children's City Clinical Hospital, Moscow, Russian Federation

####### **Correspondence:** Anna Sologub

*Pediatric Rheumatology 2024*, **22(2)**: PReS24-ABS-1117

**Introduction:** Periodic disease (Family Mediterranean fever) is a autoinflammatory disease, manifested by periodically occurring unmotivated attacks of fever in combination with serositis with a significant increase in the level of acute phase markers for 12 -72 hours, as well as other symptoms.

**Objectives:** In FMF, there is comorbidity with a number of other inflammatory diseases, and therefore most patients suffer from misdiagnoses or untimely diagnoses and associated improper treatment.

**Methods:** Case report

**Results:** Boy G., 9 years old, with complaints of acute abdominal pain, hemorrhagic rash on the lower extremities, pasty stools, fever up to 38.4C. On the 3rd day of illness, due to an increase in symptoms, he was hospitalized in an infectious diseases hospital. In connection with the suspicion of acute surgical pathology, a diagnostic laparoscopy was performed with collection of abdominal fluid for microflora growth - acute surgical pathology was excluded. Hemogram - ESR acceleration to 73 mm/h. In the biochemical blood test - an increase in the level of C-reactive protein to 197.9 mg/l. Dynamics of increased hemorrhagic rash in the area of the knee and elbow joints, along the dorsum of the feet. Hemogram - ESR up to 73mm/hour. In OAM - proteinuria 0.733 g/l. In the blood test - CRP up to 286 mg/l. A diagnosis was made - Hemorrhagic vasculitis, mixed form. Antibacterial, anticoagulant, and GCS therapy were prescribed. Condition in dynamics: symptoms of polyneuropathy. pANCA/AT to MPO, cANCA/AT to PR3 - the result is negative. On the 26th day of illness, a genetic examination for MEFV was performed - a pathogenic variant was discovered. In the TAM, proteinuria remains at 1.0 g/l, erythrocyturia 25 in the field of view. On the 32nd day of the disease, he was transferred to the rheumatology department with a diagnosis of “MEFV periodic disease in exon 10 in heterozygote c.2177T (p. Val726Ala: p.V726A.” According to clinical recommendations, Colchicine therapy was started at a dose of 0.5 mg/day and increased to 1 mg/day. On the 41st day of illness, a nephrobiopsy was performed - the child has IgA nephropathy with a picture of focal necrotizing glomerulonephritis with 6% segmental crescents. The child was diagnosed with “MEFV periodic disease in exon 10 in heterozygote p.2177T (p.Val726Ala: p.V726A). Hemorrhagic vasculitis, mixed form with kidney damage - IgA nephropathy (nephrobiopsy dated December 20, 2023).” During treatment with Colchicine, positive dynamics were observed in the form of relief of abdominal pain syndrome, episodes of fever, and rash. According to laboratory data, inflammatory activity decreased.

**Conclusion:** FMF is quite rare. An obstacle in the diagnosis and treatment of patients with FMF is the low awareness of doctors about this disease, despite the high risk of occurrence in ethnic groups, as well as comorbidity with other inflammatory diseases.

**Patient Consent**

Yes, I received consent

**Disclosure of Interest**

None Declared

### New diseases

#### 528 Ehler danlos syndrome- first case report in Oman

##### Sara Al Balushi^1^, Safiya Al Abrawi^2^, Ismail Al Rashdi^3^, Halima Al Hashmi^4^, Rovana Yousef^2^

###### ^1^General Pediatrics, Oman Medical Speciality Board; ^2^Ped Rheumatology; ^3^Genetics, Royal Hospital, Muscat; ^4^Ped Cardiology, Royal Hospital, Msucat, Oman

####### **Correspondence:** Sara Al Balushi

*Pediatric Rheumatology 2024*, **22(2)**: PReS24-ABS-1817

**Introduction:** Ehlers Danlos syndrome (EDS) is a group of inherited connective tissue disorders that affects collagen formation and function (2). It is characterized by skin hyperelasticity, hypermobility of joints, atrophic scarring, and fragility of blood vessels (1). EDS affects 1 in 5000 births and its equal in both sexes (4). Identifying the gene encoding the collagen or proteins interacting with it is important to identify the subtype of EDS which will guide the management (1). According to the 2017 international classification for ehlers danlos syndromes, there are 13 subtypes of the syndrome for which all has genetic mutation except for the hypermobile subtype (3). There are no case reports or studies found about EDS in Oman. This case report describes a child with signs and symptoms suggestive of EDS.

**Objectives:** this study aims to provide a clinical characteristics of a child presenting with pic of Ehler Danlos Syndrome

**Methods:** We reviewed the clinical presentation of a patient who presented with history suggestive of Ehler Danlos Syndrome.

**Results:** 5 years old boy who is born to first degree cousins was referred from polyclinic with history of delayed wound healing, easily bruising and he required very frequent suturing even with minor trauma, which started at age of 1 year, upon taking further history he is found to have skin hyperextensibility and joints hypermobility which are noted by the parents. Examination revealed multiple scars and atrophied skin lesions on previous trauma areas on his both elbows and knees (Fig 3), multiple bruises also noted in his bilateral lower limbs, has hyperextensible skin (Fig 2) and hypermobile joints, Beighton hypermobility score 9/9. Whole exome sequencing test sent by genetic team and still pending, baseline ECHO done which was normal.

**Conclusion:** Ehlers Danlos syndrome is a rare connective tissue disorder that affects the collagen metabolism which leads to skin hyperextensibility and joints hypermobility. Patients also can have complications involving cardiovascular system (such as aneurysms and mitral valve prolapse), gastrointestinal system (hernias and gastrointestinal diverticulosis), and ocular defects. Proper history and examination are crucial for early recognition of the signs and symptoms of EDS as these patients may present with only delayed wound healing and abnormal scarring.

**Patient Consent**

Yes, I received consent

**Disclosure of Interest**

None Declared

**References**

1. Tyler Miklovic; Vanessa C. Sieg, Ehler-Danlos Syndrome, May 29, 2023.
2. Brandon E Tapasakand David J Malis, The Mystery of Ehlers-Danlos Syndrome: An Autobiographical Case Report, 2022 Jan 25.
3. Am J Med Genet C Semin Med Genet, The 2017 international classification of the Ehlers–Danlos syndromes, 2017 Mar;175(1):8-26.
4. M Rosita, I R N Alima, E I Auerkar, Genetics of ehlers-danlos syndroms, 1943 (2021) 012092
5. Pragati Kaurani, Nikhil Marwah, Mayank Kaurani, and Narendra Padiyar, Ehler Danlos Syndrome- a case report, 2014 Mar; 8(3): 256–258.

### Systemic lupus erythematosus and antiphospholipid syndrome

#### 518 Nonsuicidal self-injury and systemic lupus erythematosus in an adolescent: a challenging diagnosis

##### Angela Mauro^1^, Alfredo Diana^2^, Marc Lorenzo Garcia^3^, Valentina Ansuini^1^, Martina Sandini^1^, Federica Bona^4^, Francesca Gaudenzi^4^, Francesca Gaboardi^4^, Roberta Sodero^4^, Gaia Bossalini^4^, Vania Giacomet^3^, Luca Bernardo^1^

###### ^1^Casa Pediatrica, Ospedale Fatebenefratelli, Milan; ^2^Pediatria, Università degli studi di Napoli Federico II, Naples; ^3^Pediatria ad indirizzo infettivologico, Ospedale Luigi Sacco; ^4^Pediatria, Università degli studi di Milano - Ospedale Buzzi, Milan, Italy

####### **Correspondence:** Angela Mauro

*Pediatric Rheumatology 2024*, **22(2)**: PReS24-ABS-1695

**Introduction:** Neuropsychiatric lupus, characterized by psychiatric and neurologic manifestations, poses diagnostic challenges in systemic lupus erythematosus (SLE).

**Objectives:** A 13-year-old Caucasian girl came to our ER with bullous, pruritic lesions on her left forearm and local edema; the lesions started appearing 2 weeks before admission after a self-harm cut on her wrist. The lesions were previously evaluated by a dermatologist and treated as impetigo with Amoxi-clavulanate for 2 days without success. Upon arrival the patient was assuming Clarithromycin for 2 days without clinical improvement. Familiar history was positive for autoimmune diseases. Upon admission we switched to Clindamycin suspecting bullous impetigo, but the patient showed 2 new purpuric lesions on the hands with positive Nikolskys’ sign.

Lab exams showed normal blood count and IgA-G-M levels, with slightly elevated IgE levels (464 kU/L). ANA and AntiSm Antibodies were positive while SSA, SSB, U1RNP, Jo1, Scl70, Beta2glycoprotein, MPO and PR3 antibodies resulted negative even on further evaluations; CRP, ESR, Quantiferon, LAC, were negative. Due to the appearance of new lesions only when the patient was left alone without supervision, suspecting cryothermic lesions from deodorant and dry shampoo we removed all possible sources of harm and witnessed an improvement of the existing lesions without new ones appearing. The Brain MRI, Fundus oculi and EEG resulted negative, while a neuropsychiatric consult suggested an emotional disorder with depressive traits. The positivity for AntiSm led us to a rheumatologic consult, were the possibility of Lupus induced Psychosis was first hypothesized.

The biopsy of the lesion was not conclusive, as the hyperkeratosis, achantosis and slight increase of dermal thickness with linear deposits of IgM and C1q on basal membrane were suggestive but not specific for cutaneous SLE lesions.

Per the new EULAR/ACR criteria, the patient had 6 points derived from the AntiSm Antibody positivity, but we were uncertain about the possibility of considering the emotional disorder, the self-injury and the cutaneous lesions in the score allowing us to reach the minimum score of 10 points (2 for each domain). Nevertheless, discussed the case with our rheumatologist specialist, we started high-dose hydroxychloroquine and corticosteroids and are strictly monitoring the patient.

**Conclusion:** We showcase a very challenging patient with an uncertain diagnosis of SLE. The self-injury lesions, which could be a manifestation of lupus induced psychosis, may be, as described in literature, the inducing factor for cutaneous manifestation and therefore allow us to fully comply with the minimum requirements for SLE diagnosis, but further evaluations are required to assess the clinical response of the patient psychosis to corticosteroids and better define the diagnosis.

**Patient Consent**

Yes, I received consent

**Disclosure of Interest**

None Declared

